# Supplementary material for: Single-cell transcriptomics reveal how root tissues adapt to soil stress
Source: Nature. 2025 Apr 30;642(8068):721–9. doi: 10.1038/s41586-025-08941-z (PMC12176638; doi:10.1038/s41586-025-08941-z)
Supplement: Supplementary file 1 — Reporting Summary [file 41586_2025_8941_MOESM1_ESM.pdf]

nature portfolio

Corresponding author(s): Bipin Pandey, Malcolm Bennett

Last updated by author(s): Mar 21, 2025

Reporting Summary

Nature Portfolio wishes to improve the reproducibility of the work that we publish. This form provides structure for consistency and transparency in reporting. For further information on Nature Portfolio policies, see our Editorial Policies and the Editorial Policy Checklist.

Statistics

For all statistical analyses, confirm that the following items are present in the figure legend, table legend, main text, or Methods section.

n/a	Confirmed	
☐	☒	The exact sample size (*n*) for each experimental group/condition, given as a discrete number and unit of measurement
☐	☒	A statement on whether measurements were taken from distinct samples or whether the same sample was measured repeatedly
☐	☒	The statistical test(s) used AND whether they are one- or two-sided<br>*Only common tests should be described solely by name; describe more complex techniques in the Methods section.*
☐	☒	A description of all covariates tested
☐	☒	A description of any assumptions or corrections, such as tests of normality and adjustment for multiple comparisons
☐	☒	A full description of the statistical parameters including central tendency (e.g. means) or other basic estimates (e.g. regression coefficient) AND variation (e.g. standard deviation) or associated estimates of uncertainty (e.g. confidence intervals)
☐	☒	For null hypothesis testing, the test statistic (e.g. *F*, *t*, *r*) with confidence intervals, effect sizes, degrees of freedom and *P* value noted<br>*Give P values as exact values whenever suitable.*
☒	☐	For Bayesian analysis, information on the choice of priors and Markov chain Monte Carlo settings
☒	☐	For hierarchical and complex designs, identification of the appropriate level for tests and full reporting of outcomes
☐	☒	Estimates of effect sizes (e.g. Cohen's *d*, Pearson's *r*), indicating how they were calculated

Our web collection on statistics for biologists contains articles on many of the points above.

Software and code

Policy information about availability of computer code

Data collection	For confocal microscopy data collection, we used Zen 2009 (version 6.0.0.303).For sequencing data collection, we mainly used the NovaSeq 6000, NovaSeq X and Nextseq 500 platforms.For spatial transcriptomics experiments, we used the Molecular Cartography platform developed by Resolve Biosciences.
Data analysis	For the analysis of bulk RNA-seq, we used STAR aligner_2.6.1b, UMI-Tools_1.1.2 and HTSeq-Count_2.0.0, and Deseq2_1.36.0.<br>For the analysis of scRNA-seq data, we used CellRanger mkfastq (v3.1.0, 10X Genomics), R version 4.2.0 (2022-04-22), R package COPILOT (PMID: 36181683) , together with major packages:<br>circlize (0.4.15), ComplexHeatmap (2.14.0), ggplot2 (3.4.2), ggrepel (0.9.3), cowplot (1.1.1), RColorBrewer (1.1-3), tidyr (1.3.0), dplyr (1.1.2), Seurat (3.1.5), scran (1.26.0), SingleCellExperiment (1.20.0), muscat (1.12.0), limma (3.54.0), gprofiler2 (0.2.2).<br><br>For the analysis of spatial transcriptomic data, we used ImageJ 1.52n.<br>For the analysis of confocal images, we used MorphoGraphX 2.0 r1-32-gf272c434.<br><br>Code availability:<br>We mainly adapted codes published in Hsu et al., 2022 (https://doi.org:10.1016/j.xpro.2022.101729), Stuart et al., 2019 (https://doi.org:10.1016/j.cell.2019.05.031), Crowell et al., 2020 (https://doi.org:10.1038/s41467-020-19894-4), and Kolberg et al., 2020 (https://doi.org:10.12688/f1000research.24956.2) for our scRNA-seq analysis. The adapted codes for analysing the scRNA-seq data are available at GitHub: https://github.com/zhumy09/scRNA-seq-for-rice

For manuscripts utilizing custom algorithms or software that are central to the research but not yet described in published literature, software must be made available to editors and reviewers. We strongly encourage code deposition in a community repository (e.g. GitHub). See the Nature Portfolio guidelines for submitting code & software for further information.

Data

Policy information about availability of data

All manuscripts must include a data availability statement. This statement should provide the following information, where applicable:

- Accession codes, unique identifiers, or web links for publicly available datasets
- A description of any restrictions on data availability
- For clinical datasets or third party data, please ensure that the statement adheres to our policy

All information supporting the conclusions are provided with the paper. scRNA-seq data for Xkitaake and Kitaake roots grown under gel and soil conditions is available at NCBI BioProject PRJNA640389 (GSE251706). scRNA-seq from Zhang et al., 2021 (PMID: 33824350) is available at NCBI BioProject PRJNA706435 and PRJNA706099.Bulk RNA-seq data for developmental stage annotation is available at NCBI BioProject PRJNA1082669 (GSE260671). Bulk RNA-seq data for protoplasting induced genes is available at NCBI BioProject PRJNA1194134 (GSE283509). Bulk RNA-seq data for Xkitaake roots grown under compacted and non-compacted soil conditions are available at NCBI BioProject PRJNA1193632 (GSE283428). Raw data for Spatial transcriptomics (Molecular Cartography) is provided in Supplementary data 4 (gel), 6 (non-compacted soils), 8 (compacted soils). Source Data for Main Figures and Extended Data Figures are provided in Supplementary Data 11 as separated excel files. Gene accession number information is available in Supplementary Table 14. Supplementary tables are provided with this manuscript. Supplementary data 1-11 are available on the Nature Figshare platform: https://doi.org/10.6084/m9.figshare.25146260. The processed scRNA-seq for gel-grown rice roots is now publicly accessible through a user-friendly platform hosted on Shiny: https://rice-singlecell.shinyapps.io/orvex_app/

Research involving human participants, their data, or biological material

Policy information about studies with human participants or human data. See also policy information about sex, gender (identity/presentation), and sexual orientation and race, ethnicity and racism.

Reporting on sex and gender	NA
Reporting on race, ethnicity, or other socially relevant groupings	NA
Population characteristics	NA
Recruitment	NA
Ethics oversight	NA

Note that full information on the approval of the study protocol must also be provided in the manuscript.

Field-specific reporting

Please select the one below that is the best fit for your research. If you are not sure, read the appropriate sections before making your selection.

☒ Life sciences ☐ Behavioural & social sciences ☐ Ecological, evolutionary & environmental sciences

For a reference copy of the document with all sections, see nature.com/documents/nr-reporting-summary-flat.pdf

Life sciences study design

All studies must disclose on these points even when the disclosure is negative.

Sample size	Sample sizes are based on the standard practice in plant biology research and the objectives of the experiments. For scRNA-seq, we included over 20,000 high-quality cells for each experimental conditions, which is a similar setup with recent scRNA-seq study in Arabidopsis roots (PMID: 36996230) and in rice roots (PMID: 33824350). Also this number of cells ensure a relatively unbiased capture of cells from different cell types. For spatial transcriptomic experiments, more than three rice root transverse sections with good mRNA detection were included for each experimental conditions. The reproducible patterns of gene detection support major experimental conclusions. For confocal imaging, over 3 root sections with reproducible cell phenotype or fluorescent dye staining patterns were included for each experimental conditions.
Data exclusions	For scRNA-seq, the low-quality cells which had low UMI counts or high protoplasting-inducible gene expression were excluded. For spatial transcriptomic experiment, the root sections with low mRNA detection were excluded. For confocal imaging, the root sections with obvious damages were excluded.
Replication	All results represented in the manuscript were replicated for at least three time. The number of cells and roots were specified in either the figure legends or the supplementary tables.
Randomization	The root sections used for scRNA-seq and spatial transcriptomics were harvested in at least three different experimental rounds. The root sections used for confocal imaging came from different roots of multiple experimental rounds. The culture conditions were kept consistent for different experimental set-up. All roots of a given genotype were taken from a single allele (Wild type or a single mutant).

Blinding	The cell annotation for scRNA-seq data was performed with unbiased processing codes. The procedures and relevant parameters of analyzing the spatial transcriptomic data and confocal images were kept consistent for different experimental conditions.

Reporting for specific materials, systems and methods

We require information from authors about some types of materials, experimental systems and methods used in many studies. Here, indicate whether each material, system or method listed is relevant to your study. If you are not sure if a list item applies to your research, read the appropriate section before selecting a response.

Materials & experimental systems

n/a	Involved in the study	
☒	☐	Antibodies
☒	☐	Eukaryotic cell lines
☒	☐	Palaeontology and archaeology
☒	☐	Animals and other organisms
☒	☐	Clinical data
☒	☐	Dual use research of concern
☐	☒	Plants

Methods

n/a	Involved in the study	
☒	☐	ChIP-seq
☒	☐	Flow cytometry
☒	☐	MRI-based neuroimaging

Dual use research of concern

Policy information about dual use research of concern

Hazards

Could the accidental, deliberate or reckless misuse of agents or technologies generated in the work, or the application of information presented in the manuscript, pose a threat to:

No	Yes	
☒	☐	Public health
☒	☐	National security
☒	☐	Crops and/or livestock
☒	☐	Ecosystems
☒	☐	Any other significant area

Experiments of concern

Does the work involve any of these experiments of concern:

No	Yes	
☒	☐	Demonstrate how to render a vaccine ineffective
☒	☐	Confer resistance to therapeutically useful antibiotics or antiviral agents
☒	☐	Enhance the virulence of a pathogen or render a nonpathogen virulent
☒	☐	Increase transmissibility of a pathogen
☒	☐	Alter the host range of a pathogen
☒	☐	Enable evasion of diagnostic/detection modalities
☒	☐	Enable the weaponization of a biological agent or toxin
☒	☐	Any other potentially harmful combination of experiments and agents

Plants

Seed stocks	We used Rice transgenic line XKitaake, which was shared by Dr. Pamela C. Ronald (UC Davis) and mhz5, aba1, aba2 and proCSLD1-VENUS-N7 seeds, which were shared by Prof. Jinsong Zhang (CAS, China) and Dr. Guoqiang Huang (SJTU,China).
Novel plant genotypes	NA
Authentication	This transgenic lines has been published in PMID: 25841037 DOI: 10.1105/tpc.15.00080 (mhz5);PMID: 31775618 DOI: 10.1186/s12864-019-6262-4 (XKitaake); PMID: 35858424 DOI: 10.1073/pnas.2201072119 (aba1, aba2); PMID: 38653244 DOI: 10.1016/j.cub.2024.03.064 (proCSLD1-VENUS-N7)
