# Supplementary material for: Single-cell transcriptomics reveal how root tissues adapt to soil stress
Source: Nature. 2025 Apr 30;642(8068):721–9. doi: 10.1038/s41586-025-08941-z (PMC12176638; doi:10.1038/s41586-025-08941-z)

tz1 Summary

Processed by COPILOT

Summary Analysis

Parameters

Iteration of Filtering	1
Mitochondrial Expression Threshold	5 %
Top High Quality Cell Filtered	1 %
Doublet Removed	Yes

Cell Stats

Estimated Number of High Quality Cell	18,526
High Quality Cell	22.99 %
Total UMI Counts in High Quality Cell	39,498,225
UMI Counts in High Quality Cell	65.81 %
Median UMI Counts per High Quality Cell	1,551
Median Genes per High Quality Cell	813
Total Genes Detected in High Quality Cell	30,597
Cell above Mitochondrial Expression Threshold	13.1 %
Estimated Doublet Rate in High Quality Cell	13.75 %

Sequencing Stats

Number of Reads Processed	445,233,609
Reads Pseudoaligned	81.5 %
Reads on Whitelist	80.68 %
Total UMI Counts	60,020,312
Sequencing Technology	10xv2
Species	Oryza sativa
Transcriptome	MSU7

Sample Stats

UMI Counts Histogram

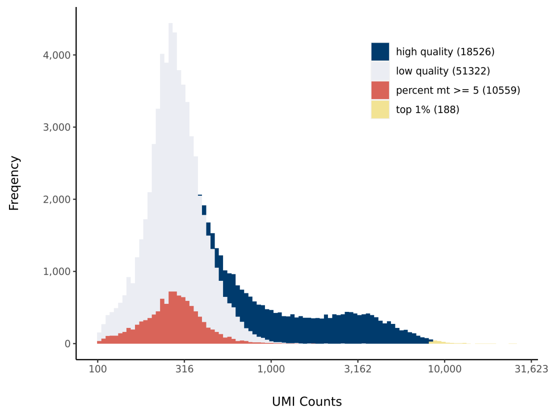

Number of Genes Histogram

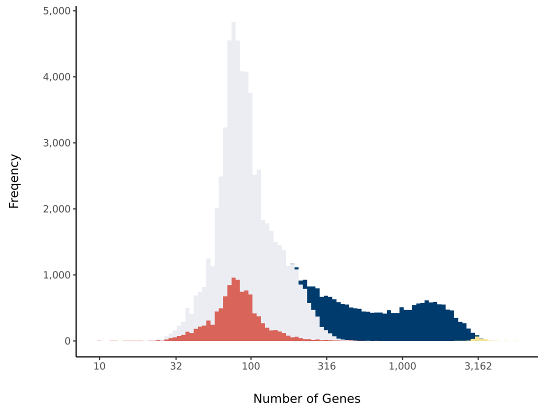

Barcode Rank Plot

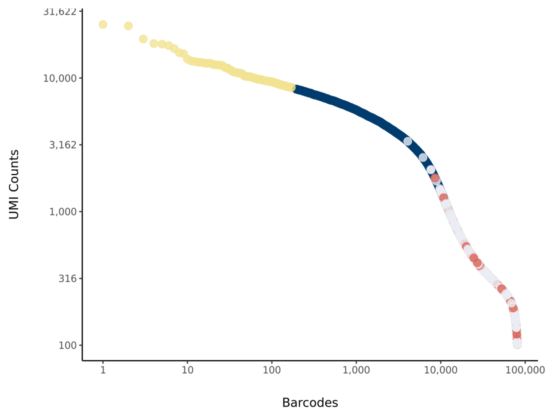

tz2 Summary

Processed by COPILOT

Summary Analysis

Parameters

Iteration of Filtering	1
Mitochondrial Expression Threshold	5 %
Top High Quality Cell Filtered	1 %
Doublet Removed	Yes

Cell Stats

Estimated Number of High Quality Cell	19,604
High Quality Cell	23.49 %
Total UMI Counts in High Quality Cell	340,202,445
UMI Counts in High Quality Cell	75.17 %
Median UMI Counts per High Quality Cell	12,262.5
Median Genes per High Quality Cell	3,436
Total Genes Detected in High Quality Cell	34,838
Cell above Mitochondrial Expression Threshold	12.83 %
Estimated Doublet Rate in High Quality Cell	14.54 %

Sequencing Stats

Number of Reads Processed	824,342,115
Reads Pseudoaligned	88 %
Reads on Whitelist	94.89 %
Total UMI Counts	452,555,775
Sequencing Technology	10xv3
Species	Oryza sativa
Transcriptome	MSU7

Sample Stats

UMI Counts Histogram

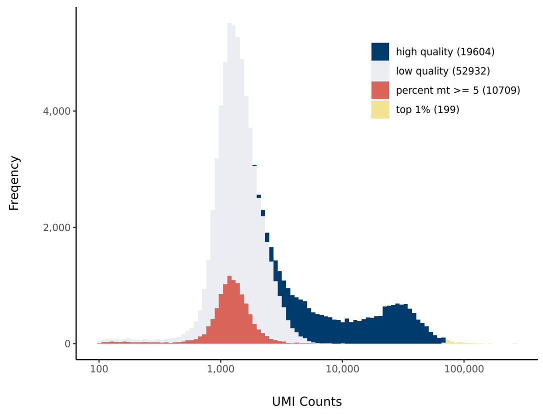

Number of Genes Histogram

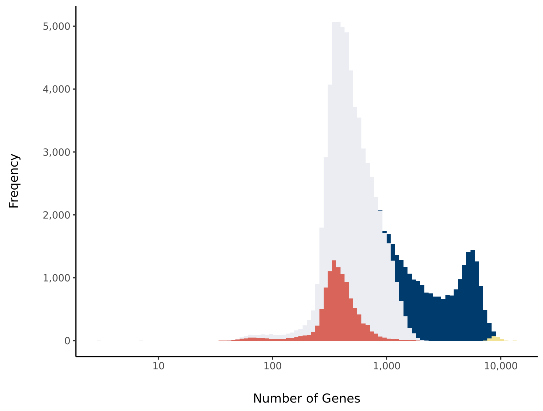

Barcode Rank Plot

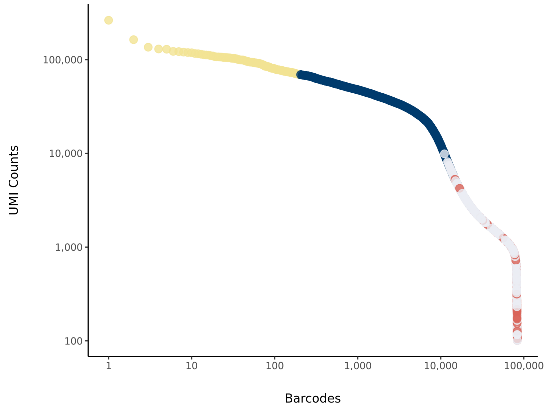

sc_7 Summary

Processed by COPILOT

Summary | Analysis

Parameters

Iteration of Filtering	1
Mitochondrial Expression Threshold	5 %
Top High Quality Cell Filtered	1 %
Doublet Removed	Yes

Cell Stats

Estimated Number of High Quality Cell	3,117
High Quality Cell	60.44 %
Total UMI Counts in High Quality Cell	74,956,511
UMI Counts in High Quality Cell	93.88 %
Median UMI Counts per High Quality Cell	22,013
Median Genes per High Quality Cell	4,234
Total Genes Detected in High Quality Cell	29,813
Cell above Mitochondrial Expression Threshold	4.6 %
Estimated Doublet Rate in High Quality Cell	2.42 %

Sequencing Stats

Number of Reads Processed	230,816,429
Reads Pseudoaligned	84.3 %
Reads on Whitelist	93.36 %
Total UMI Counts	79,845,109
Sequencing Technology	10xv3
Species	Oryza sativa
Transcriptome	MSU7

Sample Stats

Sample	sc_7
Name	Rice 1cm untreated
Source	Benfey lab
Genotype	X.Kitaake
Transgene	NA
Treatment	Untreated
Age	2_day
Timepoint	NA
Rep	2
Target Cells	10,000
Date	2019-12-18
Seq Run	Nolan_6131

UMI Counts Histogram

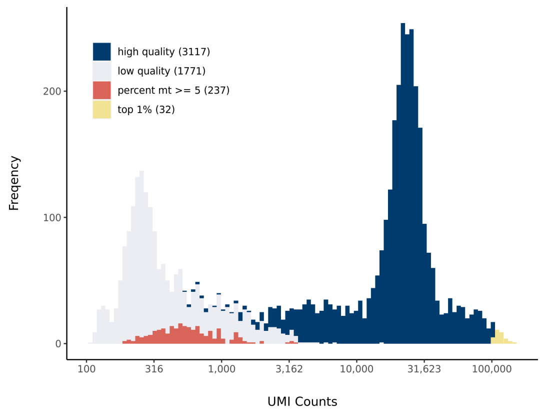

Number of Genes Histogram

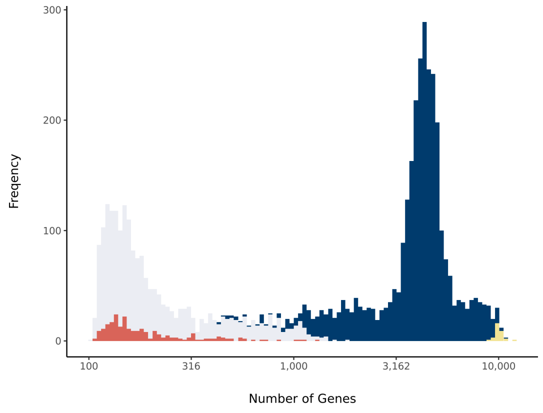

Barcode Rank Plot

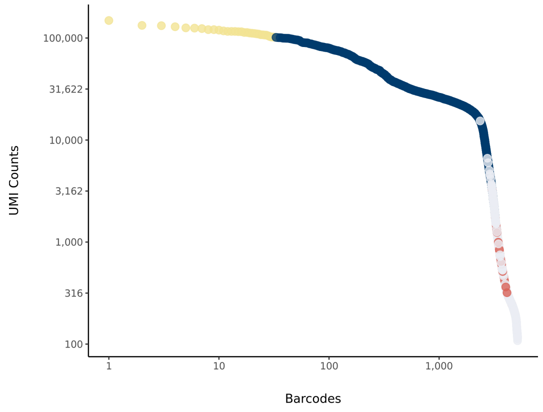

sc_108 Summary

Processed by COPILOT

Summary Analysis

Parameters

Iteration of Filtering	1
Mitochondrial Expression Threshold	5 %
Top High Quality Cell Filtered	1 %
Doublet Removed	Yes

Cell Stats

Estimated Number of High Quality Cell	5,200
High Quality Cell	5.35 %
Total UMI Counts in High Quality Cell	90,168,086
UMI Counts in High Quality Cell	51.16 %
Median UMI Counts per High Quality Cell	12,265.5
Median Genes per High Quality Cell	3,212
Total Genes Detected in High Quality Cell	30,224
Cell above Mitochondrial Expression Threshold	10.68 %
Estimated Doublet Rate in High Quality Cell	3.95 %

Sequencing Stats

Number of Reads Processed	308,624,433
Reads Pseudoaligned	84.2 %
Reads on Whitelist	86.66 %
Total UMI Counts	176,250,933
Sequencing Technology	10xv3
Species	Oryza sativa
Transcriptome	MSU7

Sample Stats

UMI Counts Histogram

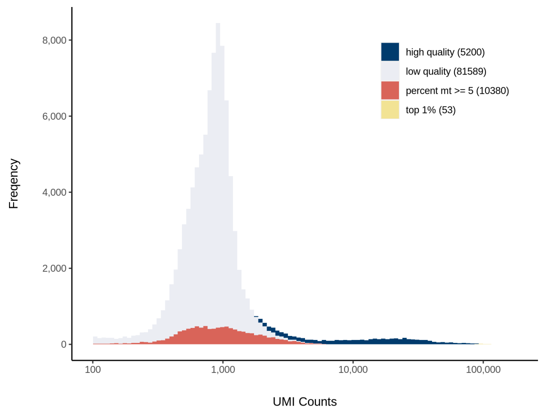

Number of Genes Histogram

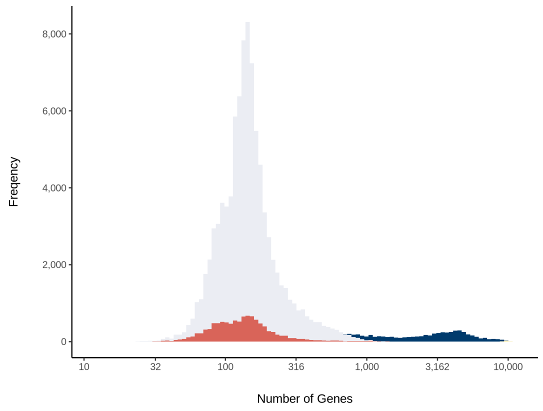

Barcode Rank Plot

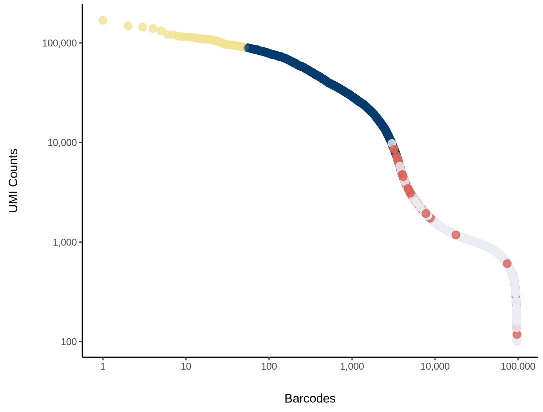

sc_109 Summary

Processed by COPILOT

Summary Analysis

Parameters

Iteration of Filtering	1
Mitochondrial Expression Threshold	5 %
Top High Quality Cell Filtered	1 %
Doublet Removed	Yes

Cell Stats

Estimated Number of High Quality Cell	4,665
High Quality Cell	4.98 %
Total UMI Counts in High Quality Cell	115,826,697
UMI Counts in High Quality Cell	64.74 %
Median UMI Counts per High Quality Cell	10,096
Median Genes per High Quality Cell	2,808
Total Genes Detected in High Quality Cell	31,061
Cell above Mitochondrial Expression Threshold	8.14 %
Estimated Doublet Rate in High Quality Cell	3.56 %

Sequencing Stats

Number of Reads Processed	351,974,854
Reads Pseudoaligned	86.8 %
Reads on Whitelist	88.32 %
Total UMI Counts	178,897,997
Sequencing Technology	10xv3
Species	Oryza sativa
Transcriptome	MSU7

Sample Stats

UMI Counts Histogram

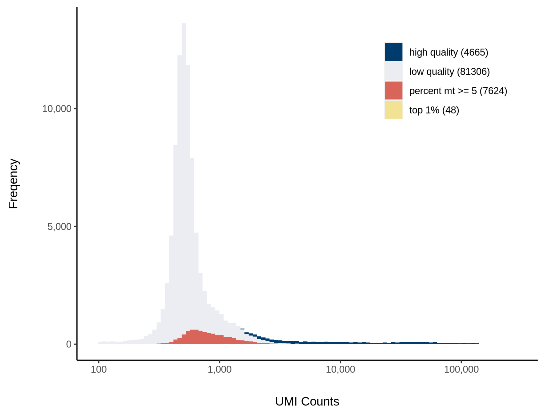

Number of Genes Histogram

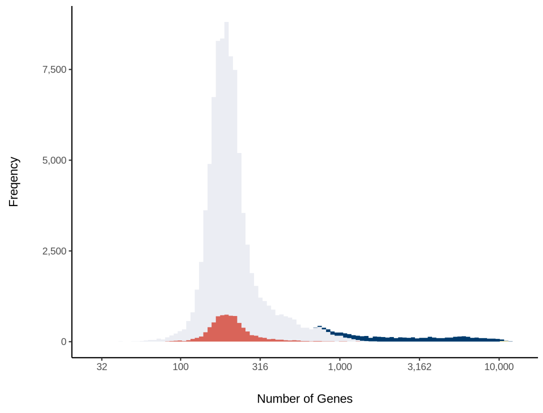

Barcode Rank Plot

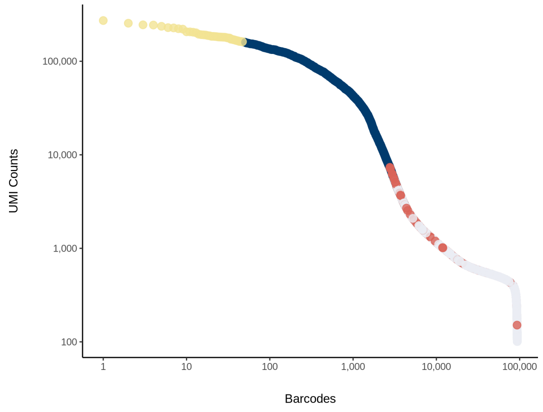

sc_115 Summary

Processed by COPILOT

Summary | Analysis

Parameters

Iteration of Filtering	1
Mitochondrial Expression Threshold	5 %
Top High Quality Cell Filtered	1 %
Doublet Removed	Yes

Cell Stats

Estimated Number of High Quality Cell	3,474
High Quality Cell	4.03 %
Total UMI Counts in High Quality Cell	72,685,831
UMI Counts in High Quality Cell	33.91 %
Median UMI Counts per High Quality Cell	10,507
Median Genes per High Quality Cell	2,689.5
Total Genes Detected in High Quality Cell	31,479
Cell above Mitochondrial Expression Threshold	6.35 %
Estimated Doublet Rate in High Quality Cell	2.68 %

Sequencing Stats

Number of Reads Processed	483,682,795
Reads Pseudoaligned	84 %
Reads on Whitelist	95.23 %
Total UMI Counts	214,373,707
Sequencing Technology	10xv3
Species	Oryza sativa
Transcriptome	MSU7

Sample Stats

UMI Counts Histogram

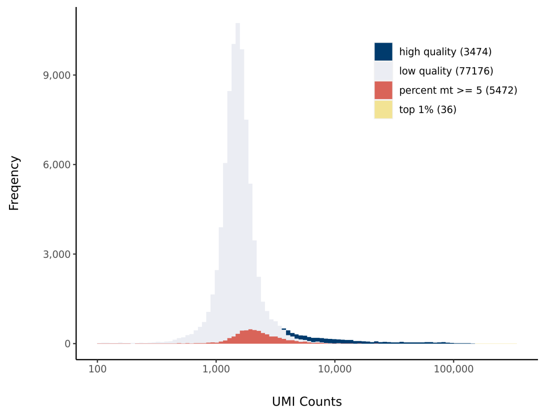

Number of Genes Histogram

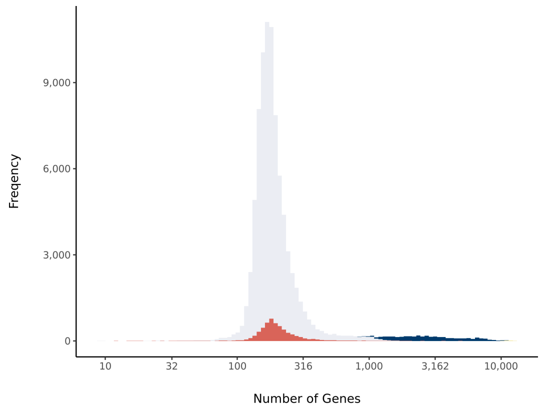

Barcode Rank Plot

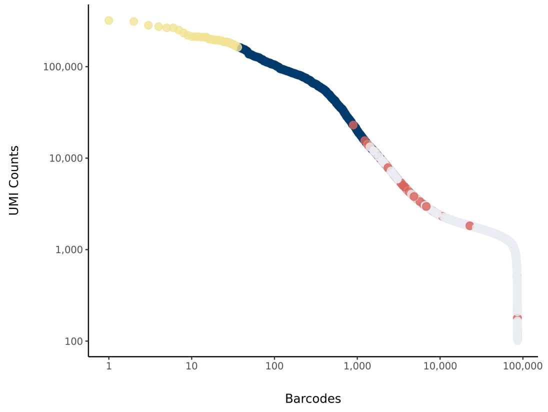

sc_116 Summary

Processed by COPILOT

Summary | Analysis

Parameters

Iteration of Filtering	1
Mitochondrial Expression Threshold	5 %
Top High Quality Cell Filtered	1 %
Doublet Removed	Yes

Cell Stats

Estimated Number of High Quality Cell	5,410
High Quality Cell	6.3 %
Total UMI Counts in High Quality Cell	110,100,592
UMI Counts in High Quality Cell	41.03 %
Median UMI Counts per High Quality Cell	13,388
Median Genes per High Quality Cell	3,376
Total Genes Detected in High Quality Cell	32,851
Cell above Mitochondrial Expression Threshold	8.18 %
Estimated Doublet Rate in High Quality Cell	4.1 %

Sequencing Stats

Number of Reads Processed	477,710,327
Reads Pseudoaligned	85.6 %
Reads on Whitelist	95.12 %
Total UMI Counts	268,341,154
Sequencing Technology	10xv3
Species	Oryza sativa
Transcriptome	MSU7

Sample Stats

UMI Counts Histogram

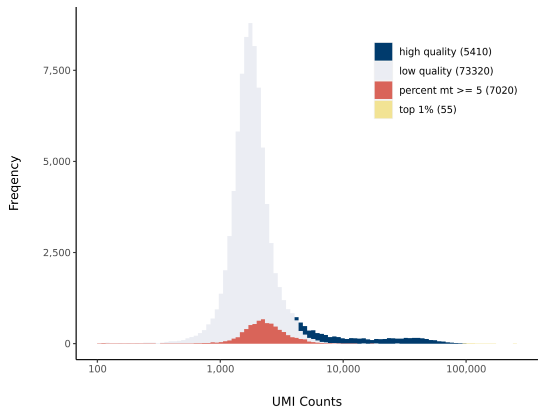

Number of Genes Histogram

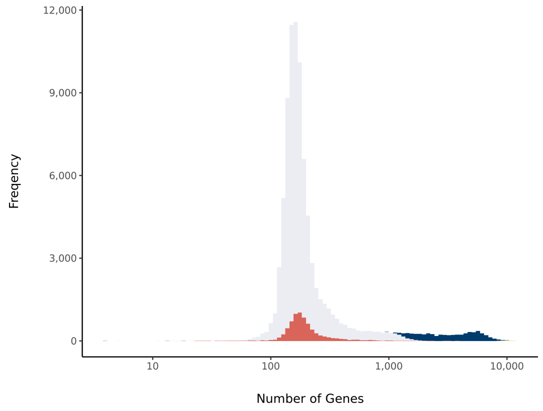

Barcode Rank Plot

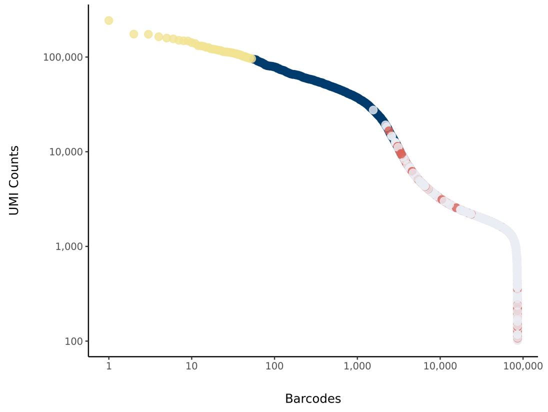

sc_192 Summary

Processed by COPILOT

Summary | Analysis

Parameters

Iteration of Filtering	1
Mitochondrial Expression Threshold	5 %
Top High Quality Cell Filtered	1 %
Doublet Removed	Yes

Cell Stats

Estimated Number of High Quality Cell	7,021
High Quality Cell	9.14 %
Total UMI Counts in High Quality Cell	144,773,020
UMI Counts in High Quality Cell	73.57 %
Median UMI Counts per High Quality Cell	18,745
Median Genes per High Quality Cell	4,052
Total Genes Detected in High Quality Cell	32,336
Cell above Mitochondrial Expression Threshold	5.24 %
Estimated Doublet Rate in High Quality Cell	5.29 %

Sequencing Stats

Number of Reads Processed	525,577,848
Reads Pseudoaligned	87.2 %
Reads on Whitelist	95.18 %
Total UMI Counts	196,774,926
Sequencing Technology	10xv3
Species	Oryza sativa
Transcriptome	MSU7

Sample Stats

Sample	sc_192
Name	X. kitaake box
Source	Benfey Lab
Genotype	X. kitaake box
Transgene	NA
Treatment	NA
Age	4 day
Timepoint	NA
Rep	NA
Target Cells	10,000
Date	2022-06-14
Seq Run	NA

UMI Counts Histogram

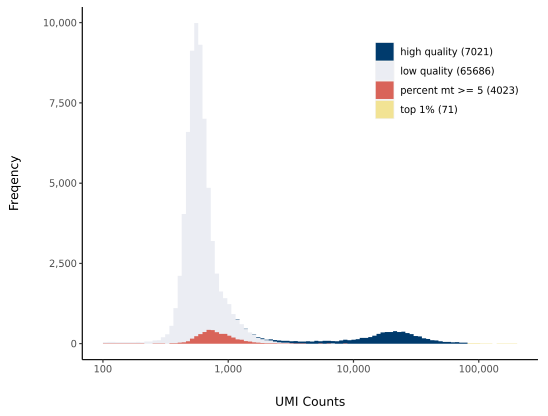

Number of Genes Histogram

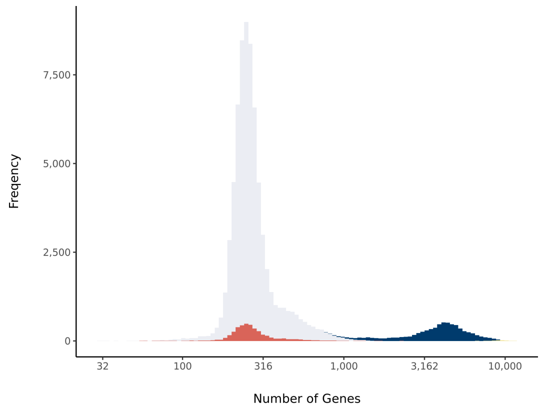

Barcode Rank Plot

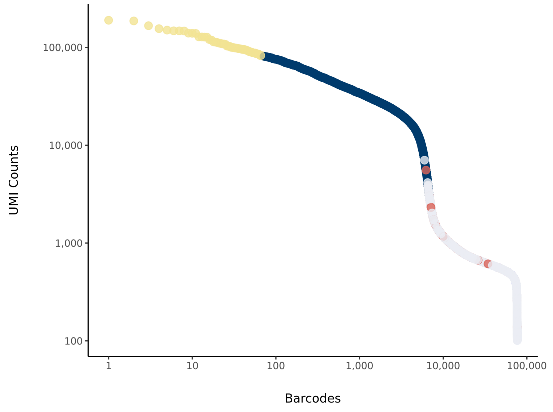

sc_193 Summary

Processed by COPILOT

Summary Analysis

Parameters

Iteration of Filtering	1
Mitochondrial Expression Threshold	5 %
Top High Quality Cell Filtered	1 %
Doublet Removed	Yes

Cell Stats

Estimated Number of High Quality Cell	5,528
High Quality Cell	7.15 %
Total UMI Counts in High Quality Cell	122,376,071
UMI Counts in High Quality Cell	67.96 %
Median UMI Counts per High Quality Cell	20,248
Median Genes per High Quality Cell	4,174
Total Genes Detected in High Quality Cell	32,078
Cell above Mitochondrial Expression Threshold	5.25 %
Estimated Doublet Rate in High Quality Cell	4.19 %

Sequencing Stats

Number of Reads Processed	534,162,746
Reads Pseudoaligned	86.3 %
Reads on Whitelist	94.23 %
Total UMI Counts	180,070,886
Sequencing Technology	10xv3
Species	Oryza sativa
Transcriptome	MSU7

Sample Stats

Sample	sc_193
Name	X. kitaake box
Source	Benfey Lab
Genotype	X. kitaake box
Transgene	NA
Treatment	NA
Age	4 day
Timepoint	NA
Rep	NA
Target Cells	10,000
Date	2022-06-14
Seq Run	NA

UMI Counts Histogram

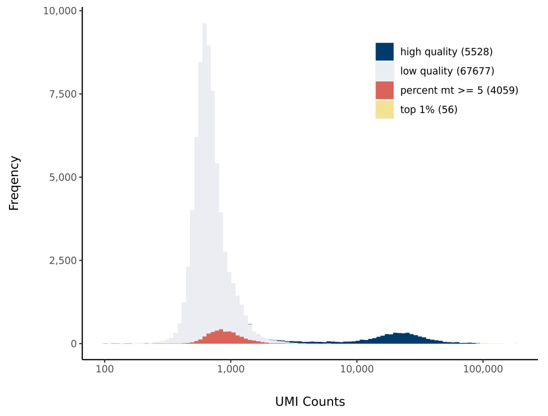

Number of Genes Histogram

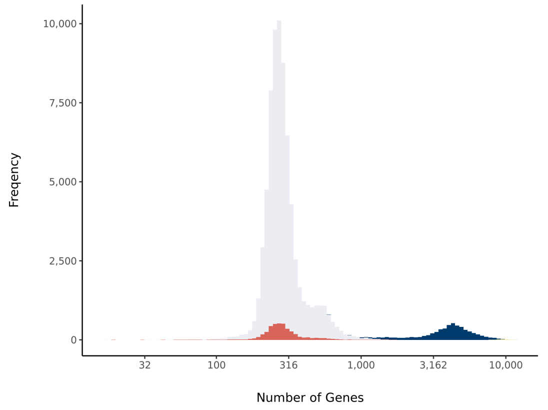

Barcode Rank Plot

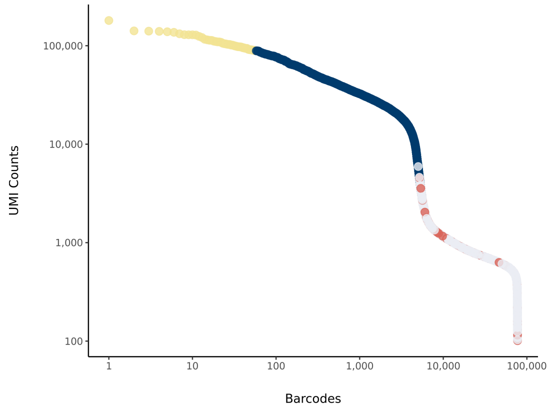

sc_194 Summary

Processed by COPILOT

Summary | Analysis

Parameters

Iteration of Filtering	1
Mitochondrial Expression Threshold	5 %
Top High Quality Cell Filtered	1 %
Doublet Removed	Yes

Cell Stats

Estimated Number of High Quality Cell	4,782
High Quality Cell	5.57 %
Total UMI Counts in High Quality Cell	172,090,018
UMI Counts in High Quality Cell	66.74 %
Median UMI Counts per High Quality Cell	32,039
Median Genes per High Quality Cell	5,049
Total Genes Detected in High Quality Cell	31,783
Cell above Mitochondrial Expression Threshold	3.37 %
Estimated Doublet Rate in High Quality Cell	3.64 %

Sequencing Stats

Number of Reads Processed	548,632,234
Reads Pseudoaligned	87.8 %
Reads on Whitelist	95.95 %
Total UMI Counts	257,856,816
Sequencing Technology	10xv3
Species	Oryza sativa
Transcriptome	MSU7

Sample Stats

Sample	sc_194
Name	X. kitaake box
Source	Benfey Lab
Genotype	X. kitaake box
Transgene	NA
Treatment	NA
Age	4 day
Timepoint	NA
Rep	NA
Target Cells	10,000
Date	2022-06-21
Seq Run	NA

UMI Counts Histogram

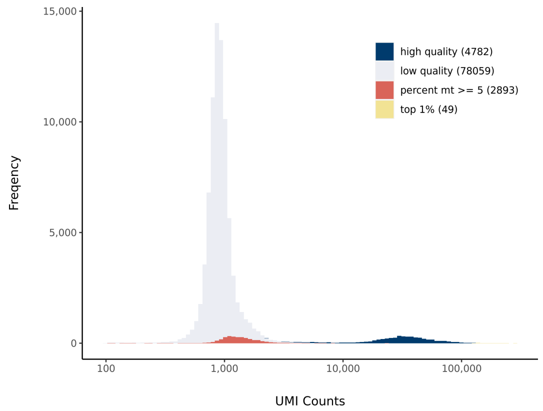

Number of Genes Histogram

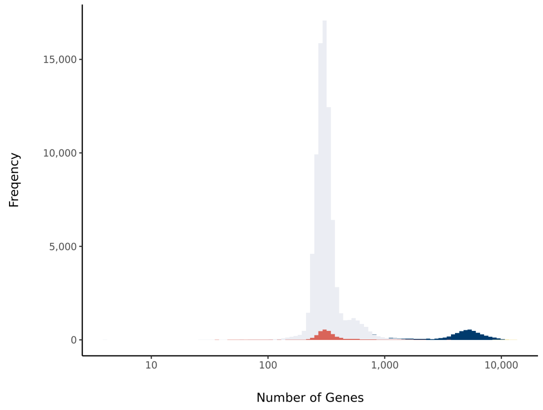

Barcode Rank Plot

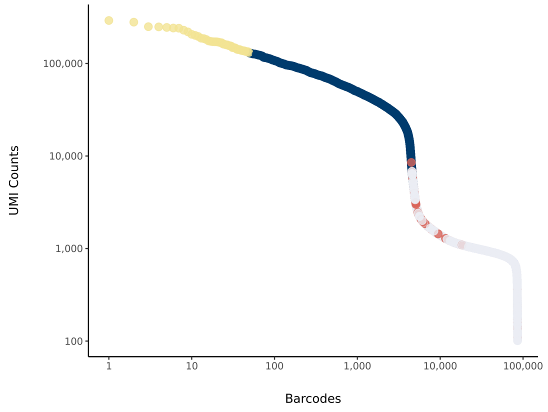

sc_195 Summary

Processed by COPILOT

Summary | Analysis

Parameters

Iteration of Filtering	1
Mitochondrial Expression Threshold	5 %
Top High Quality Cell Filtered	1 %
Doublet Removed	Yes

Cell Stats

Estimated Number of High Quality Cell	4,992
High Quality Cell	5.86 %
Total UMI Counts in High Quality Cell	146,293,247
UMI Counts in High Quality Cell	67.97 %
Median UMI Counts per High Quality Cell	26,990
Median Genes per High Quality Cell	4,666.5
Total Genes Detected in High Quality Cell	31,265
Cell above Mitochondrial Expression Threshold	3.94 %
Estimated Doublet Rate in High Quality Cell	3.8 %

Sequencing Stats

Number of Reads Processed	413,315,420
Reads Pseudoaligned	87.6 %
Reads on Whitelist	95.58 %
Total UMI Counts	215,228,971
Sequencing Technology	10xv3
Species	Oryza sativa
Transcriptome	MSU7

Sample Stats

Sample	sc_195
Name	X. kitaake box
Source	Benfey Lab
Genotype	X. kitaake box
Transgene	NA
Treatment	NA
Age	4 day
Timepoint	NA
Rep	NA
Target Cells	10,000
Date	2022-06-21
Seq Run	NA

UMI Counts Histogram

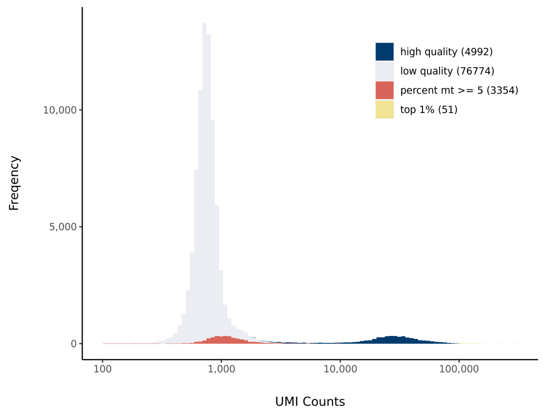

Number of Genes Histogram

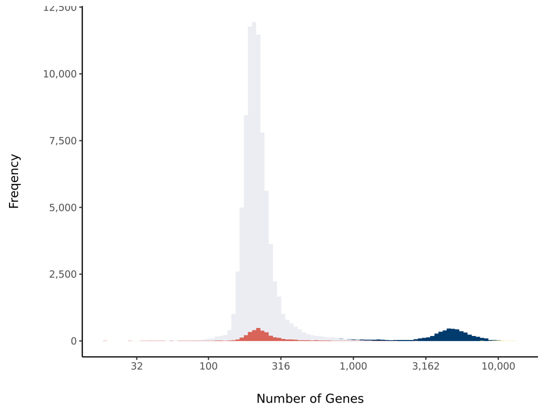

Barcode Rank Plot

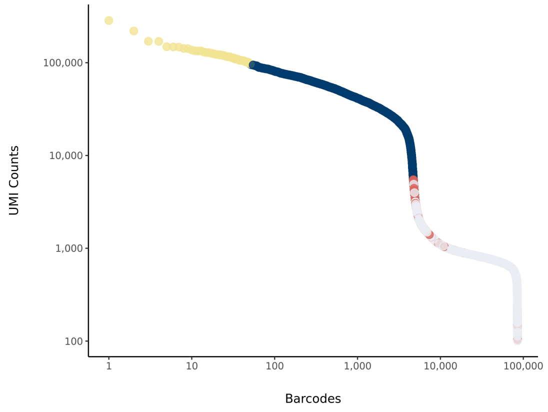

sc_196 Summary

Processed by COPILOT

Summary | Analysis

Parameters

Iteration of Filtering	1
Mitochondrial Expression Threshold	5 %
Top High Quality Cell Filtered	1 %
Doublet Removed	Yes

Cell Stats

Estimated Number of High Quality Cell	4,719
High Quality Cell	5.88 %
Total UMI Counts in High Quality Cell	77,514,592
UMI Counts in High Quality Cell	37.41 %
Median UMI Counts per High Quality Cell	13,970
Median Genes per High Quality Cell	3,284
Total Genes Detected in High Quality Cell	31,700
Cell above Mitochondrial Expression Threshold	19.02 %
Estimated Doublet Rate in High Quality Cell	3.6 %

Sequencing Stats

Number of Reads Processed	654,281,982
Reads Pseudoaligned	85.3 %
Reads on Whitelist	92.99 %
Total UMI Counts	207,177,662
Sequencing Technology	10xv3
Species	Oryza sativa
Transcriptome	MSU7

Sample Stats

Sample	sc_196
Name	X. kitaake box
Source	Benfey Lab
Genotype	X. kitaake box
Transgene	NA
Treatment	NA
Age	4 day
Timepoint	NA
Rep	NA
Target Cells	10,000
Date	2022-07-05
Seq Run	NA

UMI Counts Histogram

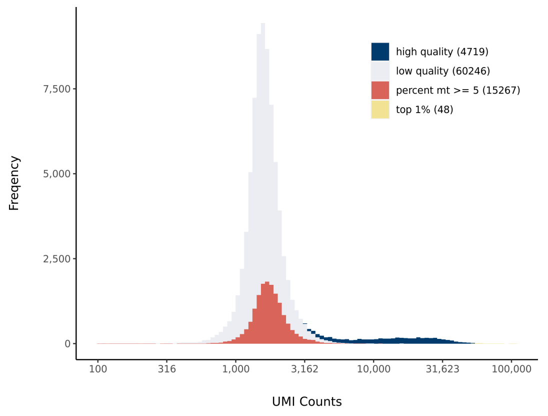

Number of Genes Histogram

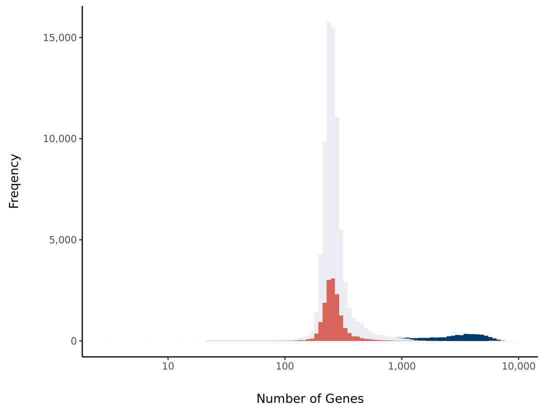

Barcode Rank Plot

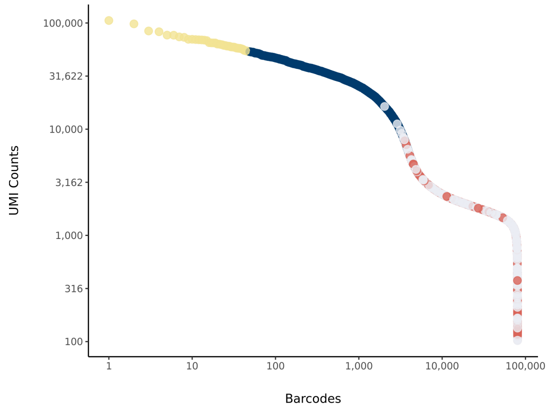

sc_199 Summary

Processed by COPILOT

Summary Analysis

Parameters

Iteration of Filtering	1
Mitochondrial Expression Threshold	5 %
Top High Quality Cell Filtered	1 %
Doublet Removed	Yes

Cell Stats

Estimated Number of High Quality Cell	11,155
High Quality Cell	6.65 %
Total UMI Counts in High Quality Cell	201,215,055
UMI Counts in High Quality Cell	63.73 %
Median UMI Counts per High Quality Cell	16,040
Median Genes per High Quality Cell	3,502
Total Genes Detected in High Quality Cell	33,467
Cell above Mitochondrial Expression Threshold	4.71 %
Estimated Doublet Rate in High Quality Cell	4.46 %

Sequencing Stats

Number of Reads Processed	695,861,968
Reads Pseudoaligned	83.2 %
Reads on Whitelist	94.7 %
Total UMI Counts	315,754,518
Sequencing Technology	10xv3
Species	Oryza sativa
Transcriptome	MSU7

Sample Stats

Sample	sc_199
Name	X. kitaake soil non-compact 1cm tip
Source	Benfey Lab
Genotype	X. kitaake soil
Transgene	NA
Treatment	NA
Age	5 days
Timepoint	NA
Rep	NA
Target Cells	12,000
Date	2022-09-07
Seq Run	NA

UMI Counts Histogram

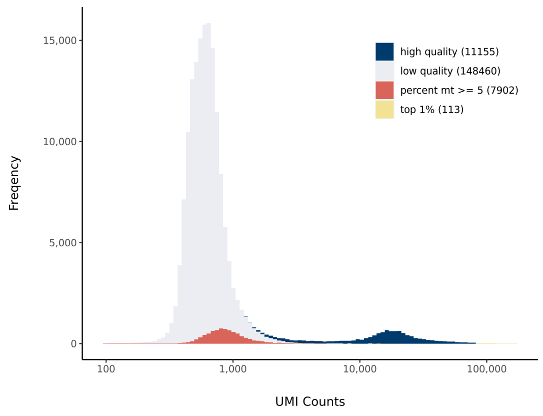

Number of Genes Histogram

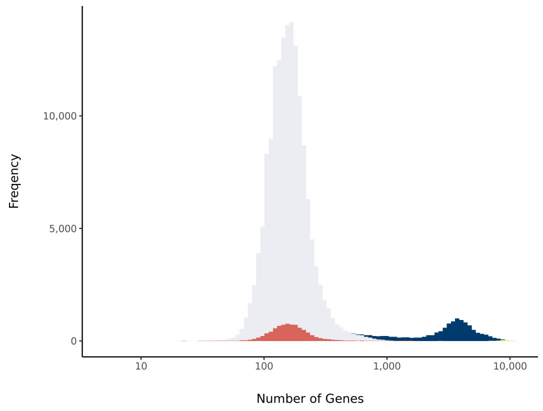

Barcode Rank Plot

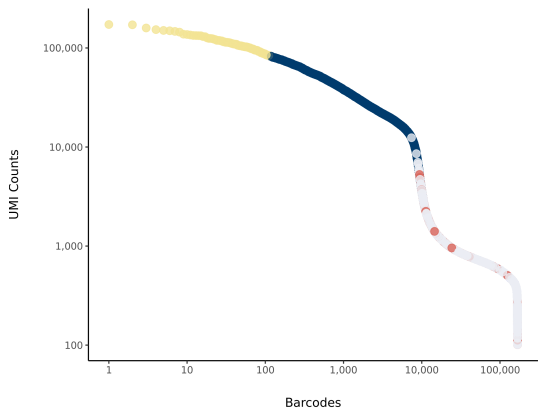

sc_200 Summary

Processed by COPILOT

Summary   Analysis

Parameters

Iteration of Filtering	1
Mitochondrial Expression Threshold	5 %
Top High Quality Cell Filtered	1 %
Doublet Removed	Yes

Cell Stats

Estimated Number of High Quality Cell	18,449
High Quality Cell	11.04 %
Total UMI Counts in High Quality Cell	196,208,424
UMI Counts in High Quality Cell	70.83 %
Median UMI Counts per High Quality Cell	9,383
Median Genes per High Quality Cell	2,797
Total Genes Detected in High Quality Cell	32,153
Cell above Mitochondrial Expression Threshold	1.71 %
Estimated Doublet Rate in High Quality Cell	7.38 %

Sequencing Stats

Number of Reads Processed	455,288,708
Reads Pseudoaligned	84.9 %
Reads on Whitelist	95.73 %
Total UMI Counts	277,021,685
Sequencing Technology	10xv3
Species	Oryza sativa
Transcriptome	MSU7

Sample Stats

Sample	sc_200
Name	X. kitaake soil non-compact 1cm tip
Source	Benfey Lab
Genotype	X. kitaake soil
Transgene	NA
Treatment	NA
Age	5 days
Timepoint	NA
Rep	NA
Target Cells	12,000
Date	2022-09-07
Seq Run	NA

UMI Counts Histogram

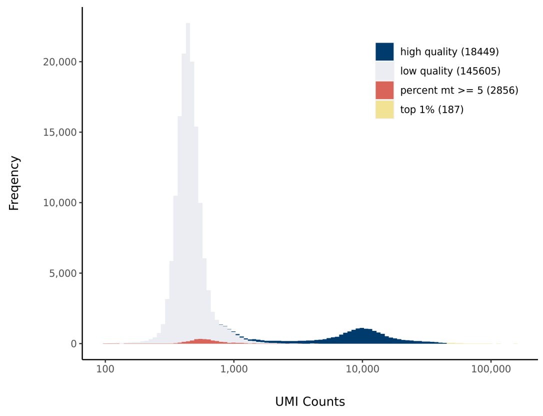

Number of Genes Histogram

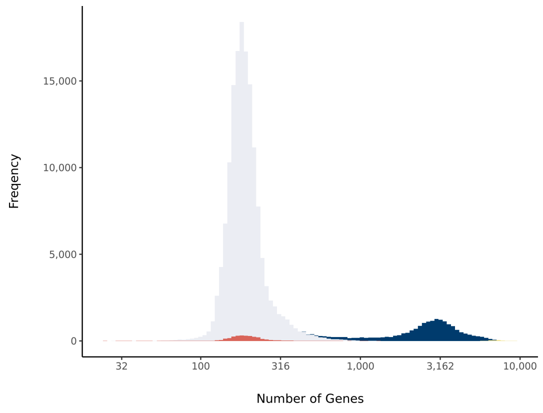

Barcode Rank Plot

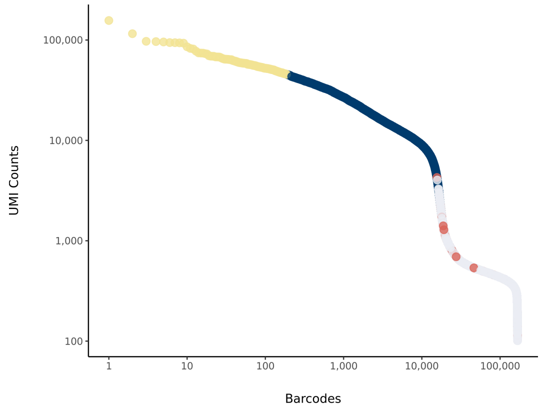

sc_201 Summary

Processed by COPILOT

Parameters

Iteration of Filtering	1
Mitochondrial Expression Threshold	5 %
Top High Quality Cell Filtered	1 %
Doublet Removed	Yes

Cell Stats

Estimated Number of High Quality Cell	14,649
High Quality Cell	8.29 %
Total UMI Counts in High Quality Cell	158,674,815
UMI Counts in High Quality Cell	56.08 %
Median UMI Counts per High Quality Cell	9,227
Median Genes per High Quality Cell	2,819
Total Genes Detected in High Quality Cell	33,754
Cell above Mitochondrial Expression Threshold	8.14 %
Estimated Doublet Rate in High Quality Cell	5.86 %

Sequencing Stats

Number of Reads Processed	659,131,469
Reads Pseudoaligned	80.2 %
Reads on Whitelist	91.18 %
Total UMI Counts	282,927,431
Sequencing Technology	10xv3
Species	Oryza sativa
Transcriptome	MSU7

Sample Stats

Sample	sc_201
Name	X. kitaake soil compact 1cm tip
Source	Benfey Lab
Genotype	X. kitaake soil
Transgene	NA
Treatment	NA
Age	5 days
Timepoint	NA
Rep	NA
Target Cells	20,000
Date	2022-09-07
Seq Run	NA

UMI Counts Histogram

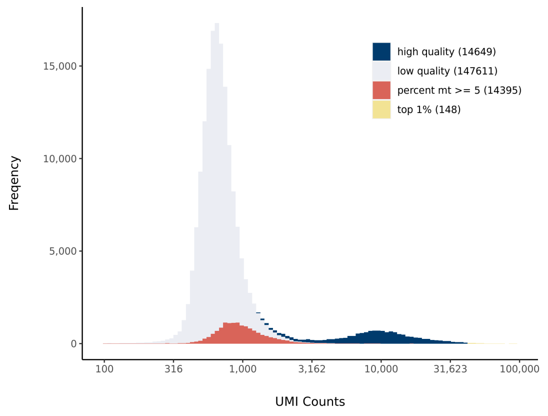

Number of Genes Histogram

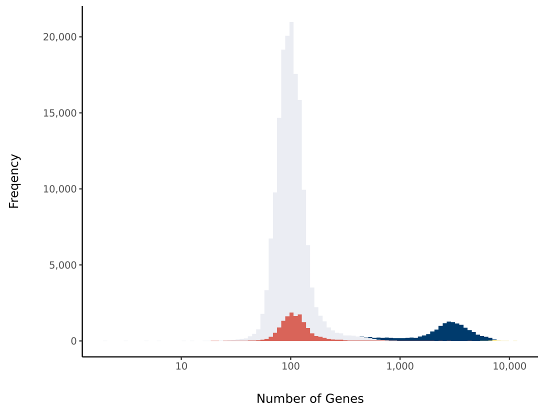

Barcode Rank Plot

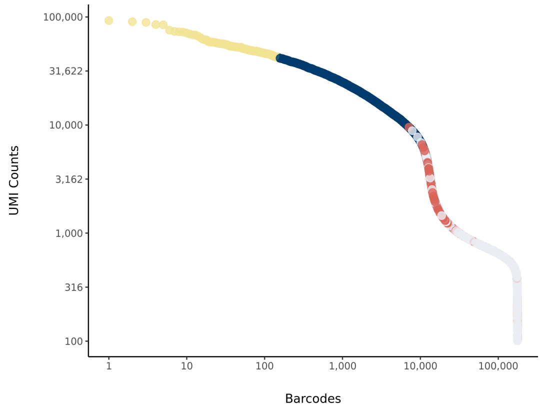

sc_202 Summary

Processed by COPILOT

Summary Analysis

Parameters

Iteration of Filtering	1
Mitochondrial Expression Threshold	5 %
Top High Quality Cell Filtered	1 %
Doublet Removed	Yes

Cell Stats

Estimated Number of High Quality Cell	10,844
High Quality Cell	6.32 %
Total UMI Counts in High Quality Cell	97,171,163
UMI Counts in High Quality Cell	38.39 %
Median UMI Counts per High Quality Cell	7,676
Median Genes per High Quality Cell	2,464
Total Genes Detected in High Quality Cell	32,280
Cell above Mitochondrial Expression Threshold	10.62 %
Estimated Doublet Rate in High Quality Cell	4.34 %

Sequencing Stats

Number of Reads Processed	830,240,949
Reads Pseudoaligned	77.2 %
Reads on Whitelist	87.84 %
Total UMI Counts	253,128,444
Sequencing Technology	10xv3
Species	Oryza sativa
Transcriptome	MSU7

Sample Stats

Sample	sc_202
Name	X. kitaake soil compact 1cm tip
Source	Benfey Lab
Genotype	X. kitaake soil
Transgene	NA
Treatment	NA
Age	5 days
Timepoint	NA
Rep	NA
Target Cells	20,000
Date	2022-09-07
Seq Run	NA

UMI Counts Histogram

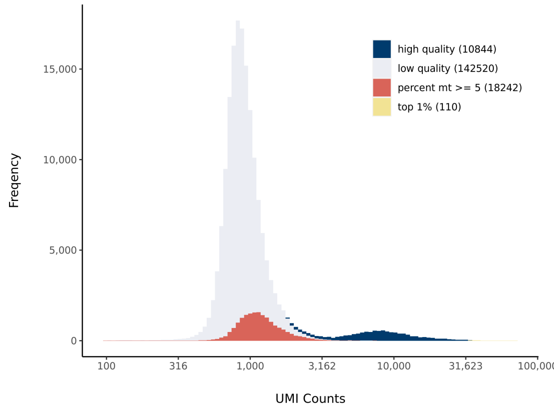

Number of Genes Histogram

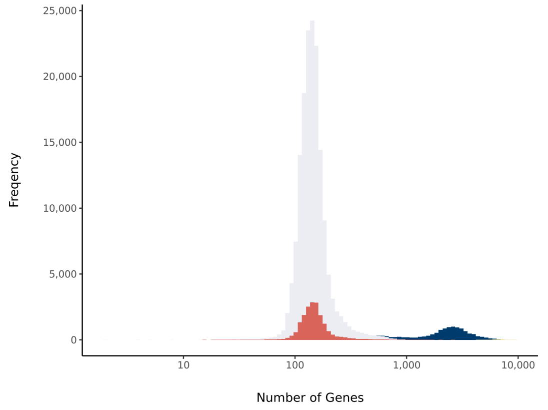

Barcode Rank Plot

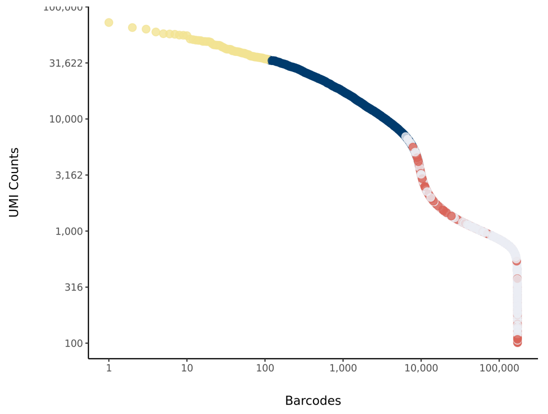

sc_303 Summary

Processed by COPILOT

Summary Analysis

Parameters

Iteration of Filtering	72
Mitochondrial Expression Threshold	5 %
Top High Quality Cell Filtered	1 %
Doublet Removed	Yes

Cell Stats

Estimated Number of High Quality Cell	2,170
High Quality Cell	2.03 %
Total UMI Counts in High Quality Cell	107,382,865
UMI Counts in High Quality Cell	41.81 %
Median UMI Counts per High Quality Cell	39,497.5
Median Genes per High Quality Cell	5,789.5
Total Genes Detected in High Quality Cell	30,808
Cell above Mitochondrial Expression Threshold	3.49 %
Estimated Doublet Rate in High Quality Cell	0.87 %

Sequencing Stats

Number of Reads Processed	1,341,840,619
Reads Pseudoaligned	85.7 %
Reads on Whitelist	95.63 %
Total UMI Counts	256,837,310
Sequencing Technology	10xv3
Species	Oryza sativa
Transcriptome	MSU7

Sample Stats

Sample	sc_303
Name	Kitaake gel condition scRNA-seq, rep#1
Source	Benfey Lab
Genotype	WT
Transgene	NA
Treatment	NA
Age	4 days
Timepoint	NA
Rep	1
Target Cells	NA
Date	2024-06-10
Seq Run	NA

UMI Counts Histogram

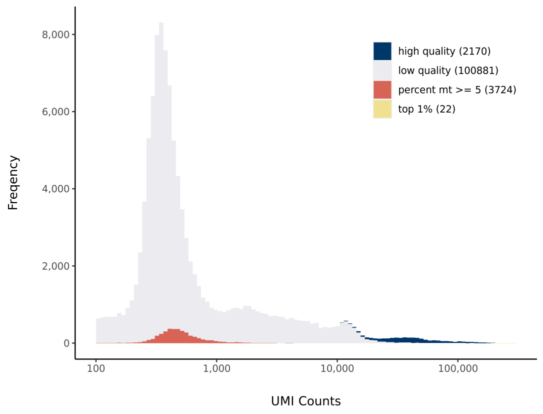

Number of Genes Histogram

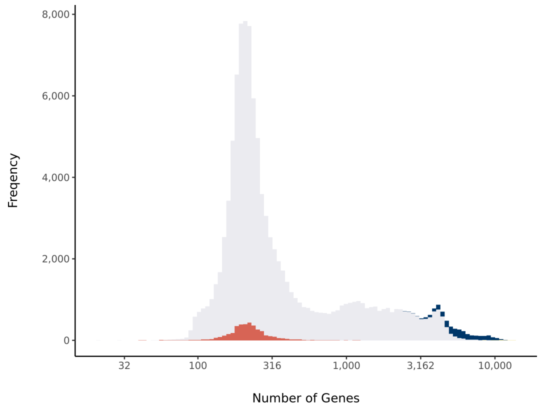

Barcode Rank Plot

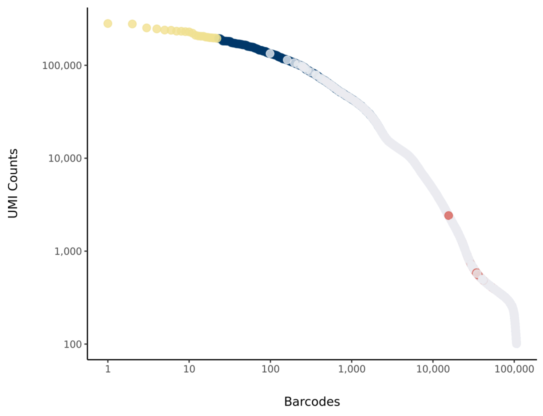

sc_304 Summary

Processed by COPILOT

Summary | Analysis

Parameters

Iteration of Filtering	1
Mitochondrial Expression Threshold	5 %
Top High Quality Cell Filtered	1 %
Doublet Removed	Yes

Cell Stats

Estimated Number of High Quality Cell	2,839
High Quality Cell	3.48 %
Total UMI Counts in High Quality Cell	81,160,087
UMI Counts in High Quality Cell	43.34 %
Median UMI Counts per High Quality Cell	18,772
Median Genes per High Quality Cell	4,313
Total Genes Detected in High Quality Cell	30,558
Cell above Mitochondrial Expression Threshold	5.38 %
Estimated Doublet Rate in High Quality Cell	1.14 %

Sequencing Stats

Number of Reads Processed	1,302,977,145
Reads Pseudoaligned	85.3 %
Reads on Whitelist	94.91 %
Total UMI Counts	187,251,975
Sequencing Technology	10xv3
Species	Oryza sativa
Transcriptome	MSU7

Sample Stats

Sample	sc_304
Name	Kitaake gel condition scRNA-seq, rep#2
Source	Benfey Lab
Genotype	WT
Transgene	NA
Treatment	NA
Age	4 days
Timepoint	NA
Rep	2
Target Cells	NA
Date	2024-06-10
Seq Run	NA

UMI Counts Histogram

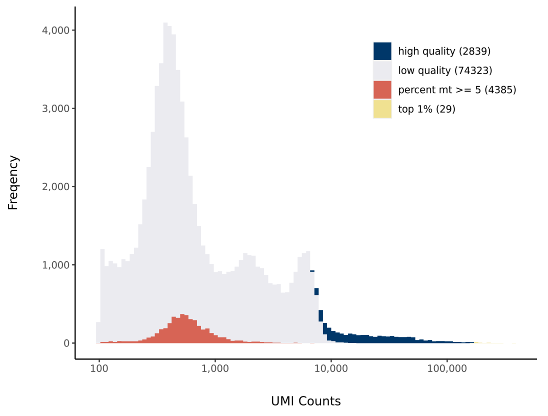

Number of Genes Histogram

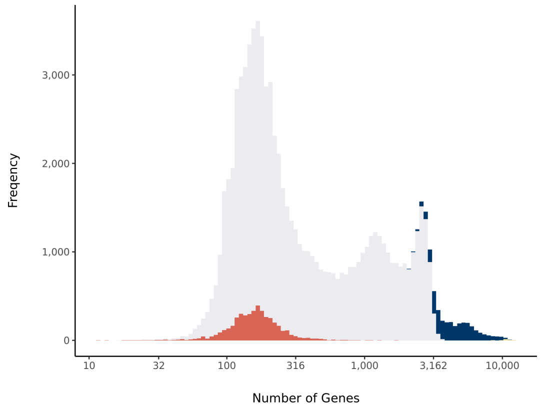

Barcode Rank Plot

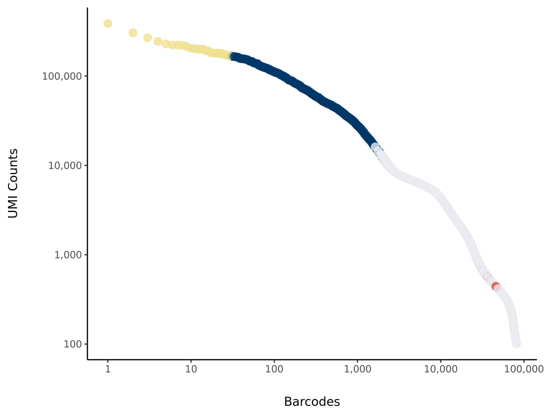

sc_305 Summary

Processed by COPILOT

Summary Analysis

Parameters

Iteration of Filtering	28
Mitochondrial Expression Threshold	5 %
Top High Quality Cell Filtered	1 %
Doublet Removed	Yes

Cell Stats

Estimated Number of High Quality Cell	4,077
High Quality Cell	2.84 %
Total UMI Counts in High Quality Cell	63,699,740
UMI Counts in High Quality Cell	44.67 %
Median UMI Counts per High Quality Cell	12,847
Median Genes per High Quality Cell	3,336
Total Genes Detected in High Quality Cell	30,575
Cell above Mitochondrial Expression Threshold	11.79 %
Estimated Doublet Rate in High Quality Cell	1.63 %

Sequencing Stats

Number of Reads Processed	1,138,984,950
Reads Pseudoaligned	85.3 %
Reads on Whitelist	88.32 %
Total UMI Counts	142,616,072
Sequencing Technology	10xv3
Species	Oryza sativa
Transcriptome	MSU7

Sample Stats

Sample	sc_305
Name	Kitaake non-compacted soil condition scRNA-seq, rep#1
Source	Benfey Lab
Genotype	WT
Transgene	NA
Treatment	NA
Age	4 days
Timepoint	NA
Rep	1
Target Cells	NA
Date	2024-06-10
Seq Run	NA

UMI Counts Histogram

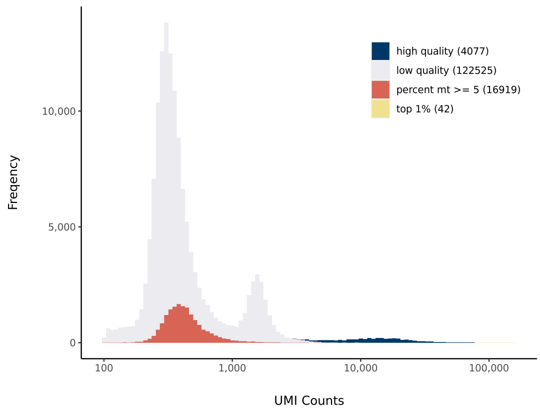

Number of Genes Histogram

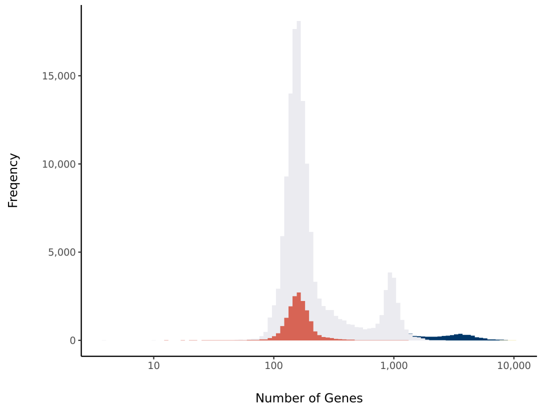

Barcode Rank Plot

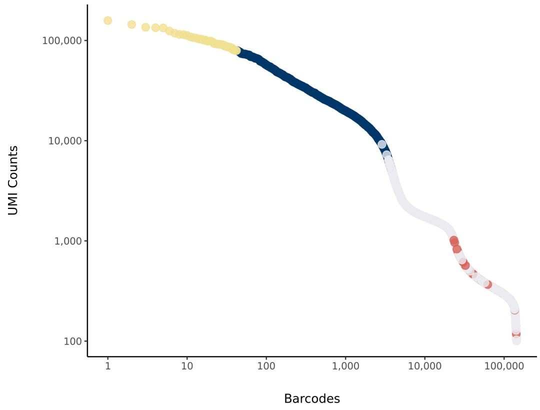

sc_306 Summary

Processed by COPILOT

Summary Analysis

Parameters

Iteration of Filtering	1
Mitochondrial Expression Threshold	5 %
Top High Quality Cell Filtered	1 %
Doublet Removed	Yes

Cell Stats

Estimated Number of High Quality Cell	2,869
High Quality Cell	2.97 %
Total UMI Counts in High Quality Cell	55,480,680
UMI Counts in High Quality Cell	45.09 %
Median UMI Counts per High Quality Cell	16,357
Median Genes per High Quality Cell	3,788
Total Genes Detected in High Quality Cell	29,608
Cell above Mitochondrial Expression Threshold	16.16 %
Estimated Doublet Rate in High Quality Cell	1.15 %

Sequencing Stats

Number of Reads Processed	1,123,165,607
Reads Pseudoaligned	83.6 %
Reads on Whitelist	83.77 %
Total UMI Counts	123,054,985
Sequencing Technology	10xv3
Species	Oryza sativa
Transcriptome	MSU7

Sample Stats

Sample	sc_306
Name	Kitaake non-compacted soil condition scRNA-seq, rep#2
Source	Benfey Lab
Genotype	WT
Transgene	NA
Treatment	NA
Age	4 days
Timepoint	NA
Rep	2
Target Cells	NA
Date	2024-06-10
Seq Run	NA

UMI Counts Histogram

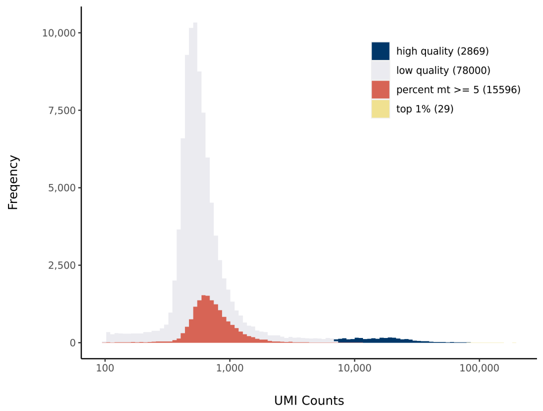

Number of Genes Histogram

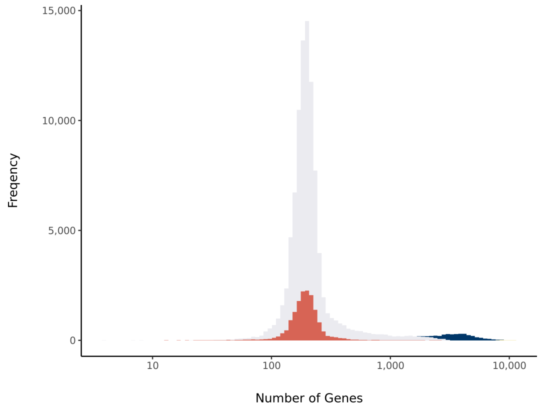

Barcode Rank Plot

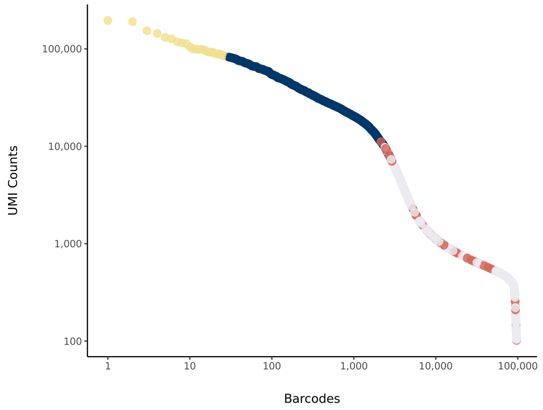

Supplement: Supplementary file 16 — scRNA-seq sample COPILOT summary information and details related to annotation. A combined PDF summary file is also included. [file 41586_2025_8941_MOESM16_ESM.zip › Supplementary Data 1_scRNA_seq data_COPILOT_processing_files/Data-S1_scRNA-seq data summary_Rice.pdf]
