# Supplementary material for: Single-cell transcriptomics reveal how root tissues adapt to soil stress
Source: Nature. 2025 Apr 30;642(8068):721–9. doi: 10.1038/s41586-025-08941-z (PMC12176638; doi:10.1038/s41586-025-08941-z)
Supplement: Supplementary file 16 — scRNA-seq sample COPILOT summary information and details related to annotation. A combined PDF summary file is also included. [file 41586_2025_8941_MOESM16_ESM.zip › Supplementary Data 1_scRNA_seq data_COPILOT_processing_files/sc_304_summary.html]

 R output 

import namespace="mml" implementation="#mathplayer"?


Analysis

Summary

# sc\_304 Summary

### Processed by COPILOT

### Parameters

|  |  |
| --- | --- |
| Iteration of Filtering | 1 |
| Mitochondrial Expression Threshold | 5 % |
| Top High Quality Cell Filtered | 1 % |
| Doublet Removed | Yes |

### Cell Stats

|  |  |
| --- | --- |
| Estimated Number of High Quality Cell | 2,839 |
| High Quality Cell | 3.48 % |
| Total UMI Counts in High Quality Cell | 81,160,087 |
| UMI Counts in High Quality Cell | 43.34 % |
| Median UMI Counts per High Quality Cell | 18,772 |
| Median Genes per High Quality Cell | 4,313 |
| Total Genes Detected in High Quality Cell | 30,558 |
| Cell above Mitochondrial Expression Threshold | 5.38 % |
| Estimated Doublet Rate in High Quality Cell | 1.14 % |

### Sequencing Stats

|  |  |
| --- | --- |
| Number of Reads Processed | 1,302,977,145 |
| Reads Pseudoaligned | 85.3 % |
| Reads on Whitelist | 94.91 % |
| Total UMI Counts | 187,251,975 |
| Sequencing Technology | 10xv3 |
| Species | Oryza sativa |
| Transcriptome | MSU7 |

### Sample Stats

|  |  |
| --- | --- |
| Sample | sc\_304 |
| Name | Kitaake gel condition scRNA-seq, rep#2 |
| Source | Benfey Lab |
| Genotype | WT |
| Transgene | NA |
| Treatment | NA |
| Age | 4 days |
| Timepoint | NA |
| Rep | 2 |
| Target Cells | NA |
| Date | 2024-06-10 |
| Seq Run | NA |

### UMI Counts Histogram

### Number of Genes Histogram

### Barcode Rank Plot

Analysis

Summary

# sc\_304 Analysis

### Processed by COPILOT
