# Supplementary figures and images for: Single-cell transcriptomics reveal how root tissues adapt to soil stress
Source: Nature. 2025 Apr 30;642(8068):721–9. doi: 10.1038/s41586-025-08941-z (PMC12176638; doi:10.1038/s41586-025-08941-z)

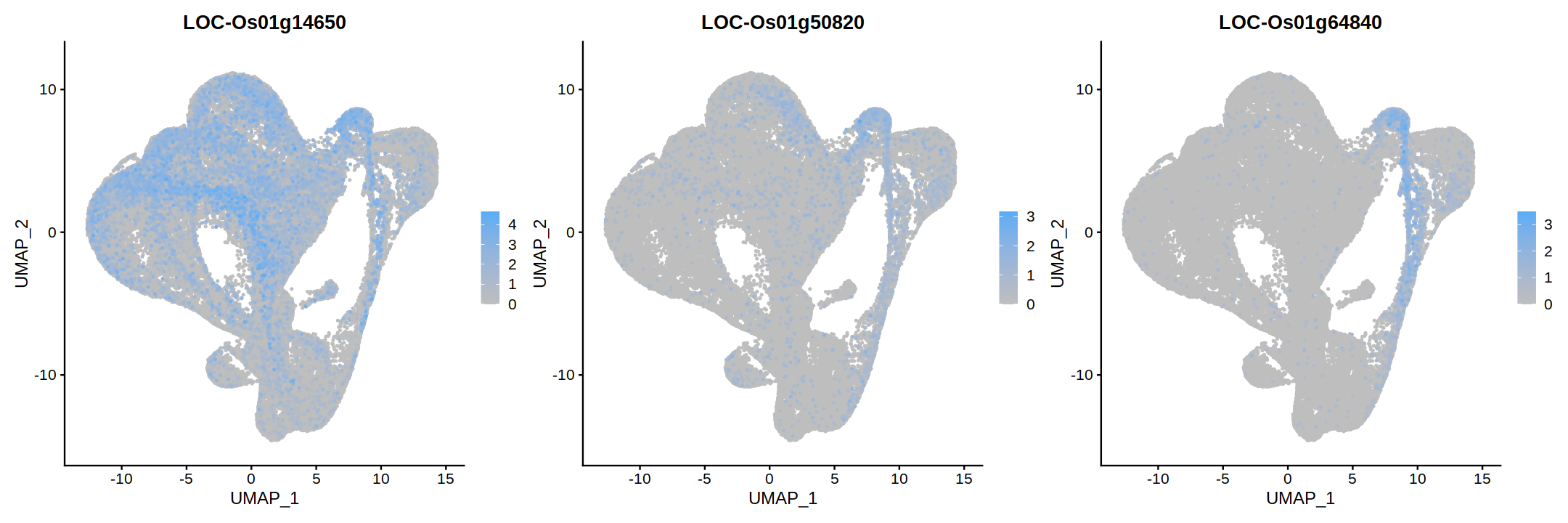

Supplement: Supplementary file 18 — Combined feature plots representing the expression patterns of cell type markers in single-cell RNA-seq data. Each image represents the gene expressions of markers for one certain cell type. [file 41586_2025_8941_MOESM18_ESM.zip › Supplementary Data 3_Marker_expressions_in_gel-based_scRNAseq_Rice/Atrichoblast marker expression.png]

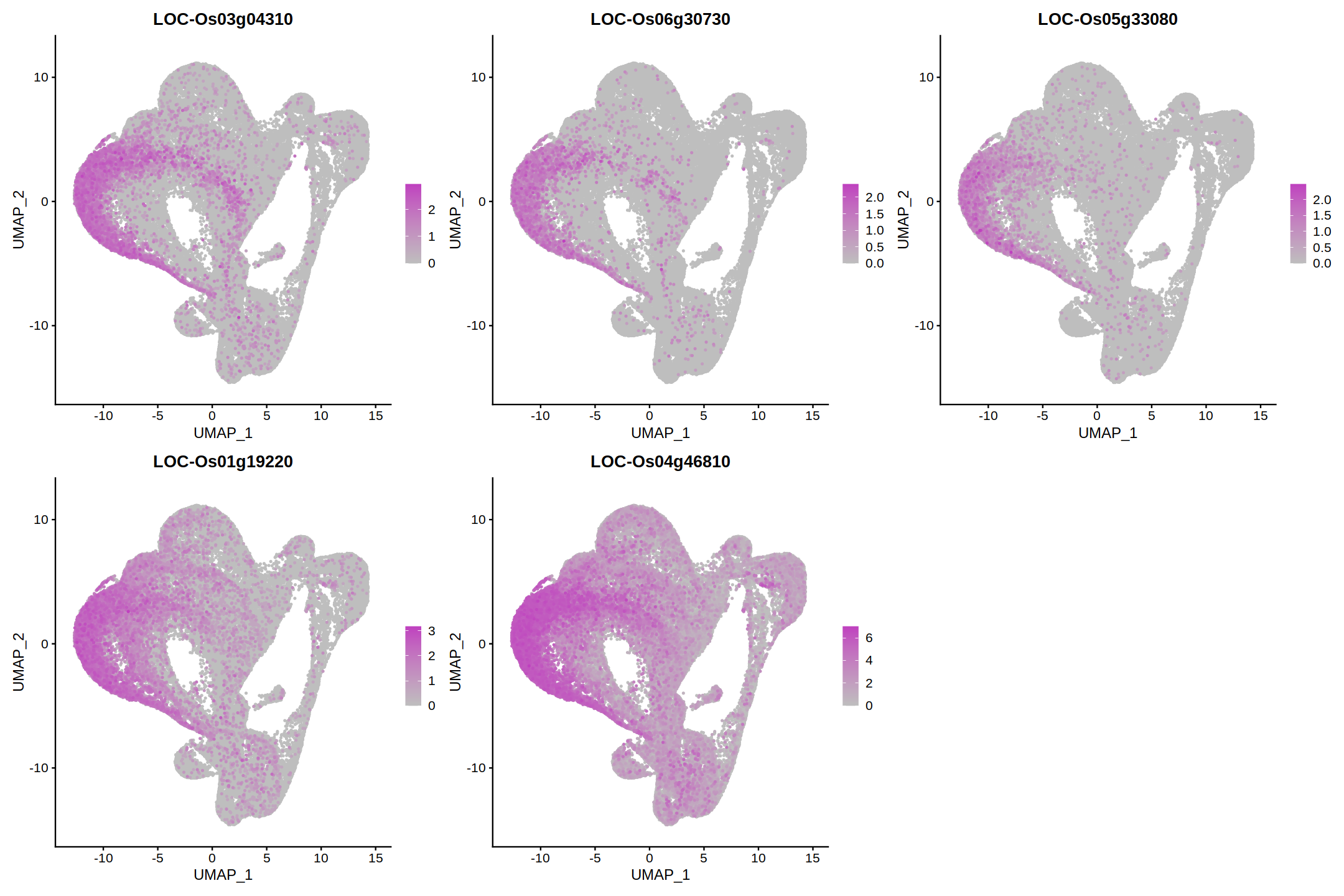

Supplement: Supplementary file 18 — Combined feature plots representing the expression patterns of cell type markers in single-cell RNA-seq data. Each image represents the gene expressions of markers for one certain cell type. [file 41586_2025_8941_MOESM18_ESM.zip › Supplementary Data 3_Marker_expressions_in_gel-based_scRNAseq_Rice/Cortex marker expression.png]

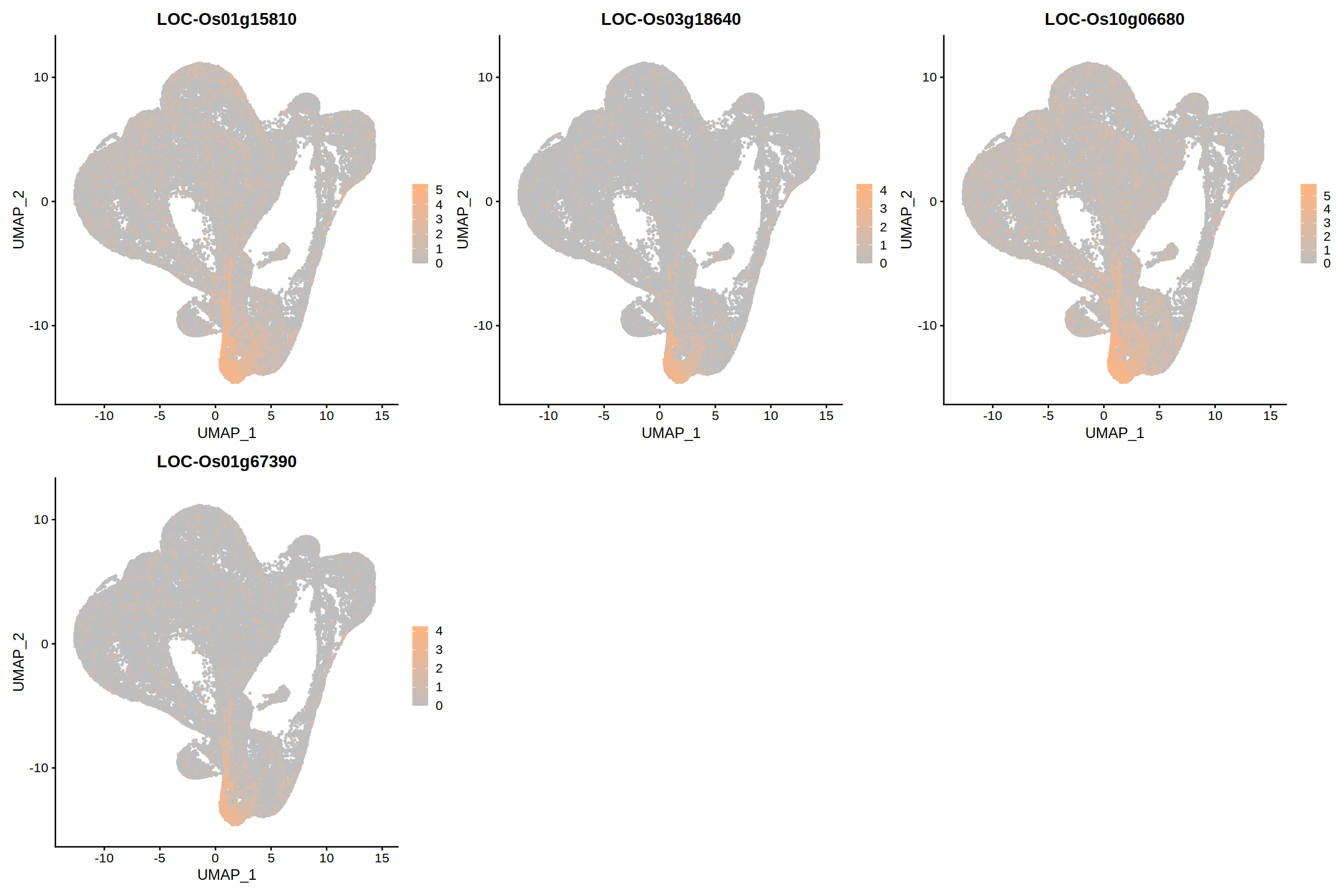

Supplement: Supplementary file 18 — Combined feature plots representing the expression patterns of cell type markers in single-cell RNA-seq data. Each image represents the gene expressions of markers for one certain cell type. [file 41586_2025_8941_MOESM18_ESM.zip › Supplementary Data 3_Marker_expressions_in_gel-based_scRNAseq_Rice/Endodermis marker expression.png]

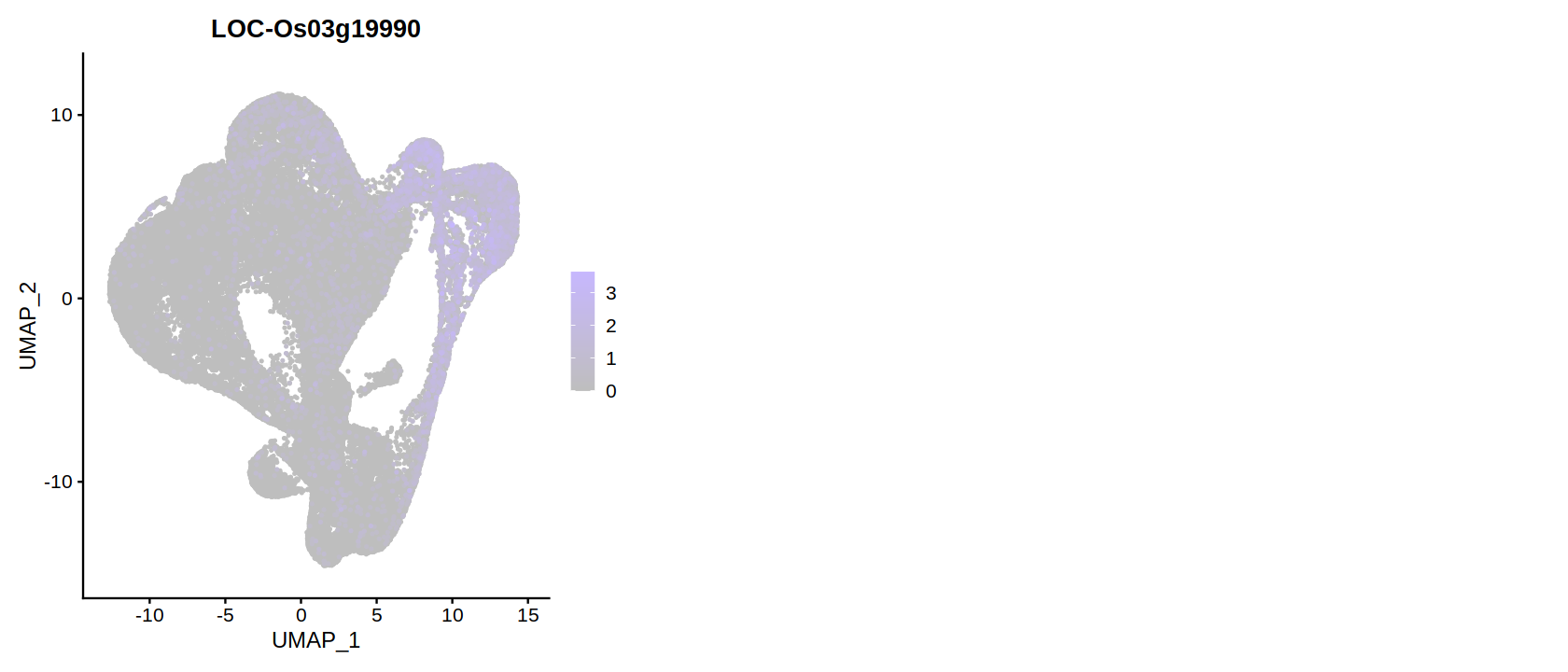

Supplement: Supplementary file 18 — Combined feature plots representing the expression patterns of cell type markers in single-cell RNA-seq data. Each image represents the gene expressions of markers for one certain cell type. [file 41586_2025_8941_MOESM18_ESM.zip › Supplementary Data 3_Marker_expressions_in_gel-based_scRNAseq_Rice/Epidermis marker expression.png]

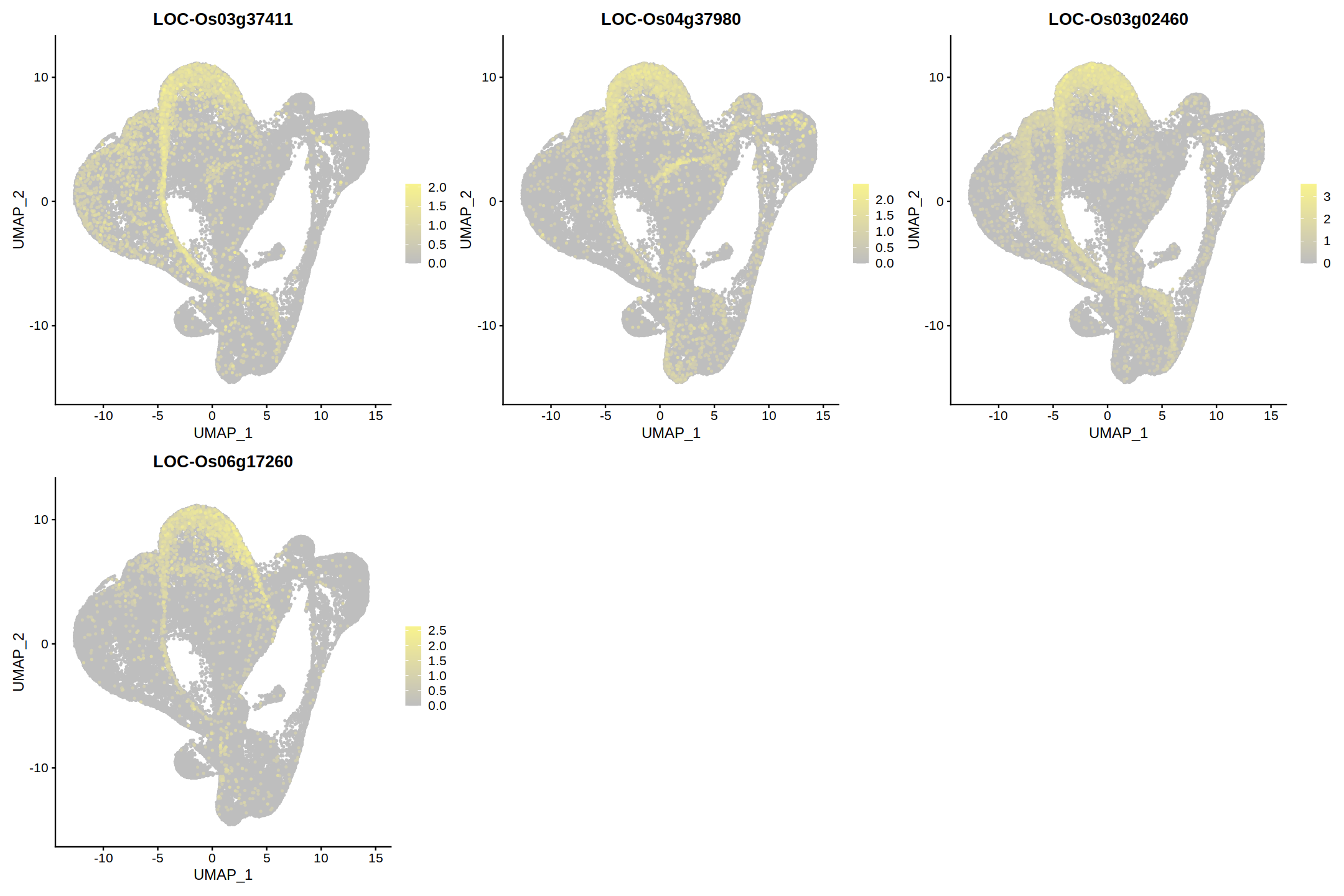

Supplement: Supplementary file 18 — Combined feature plots representing the expression patterns of cell type markers in single-cell RNA-seq data. Each image represents the gene expressions of markers for one certain cell type. [file 41586_2025_8941_MOESM18_ESM.zip › Supplementary Data 3_Marker_expressions_in_gel-based_scRNAseq_Rice/Exodermis marker expression.png]

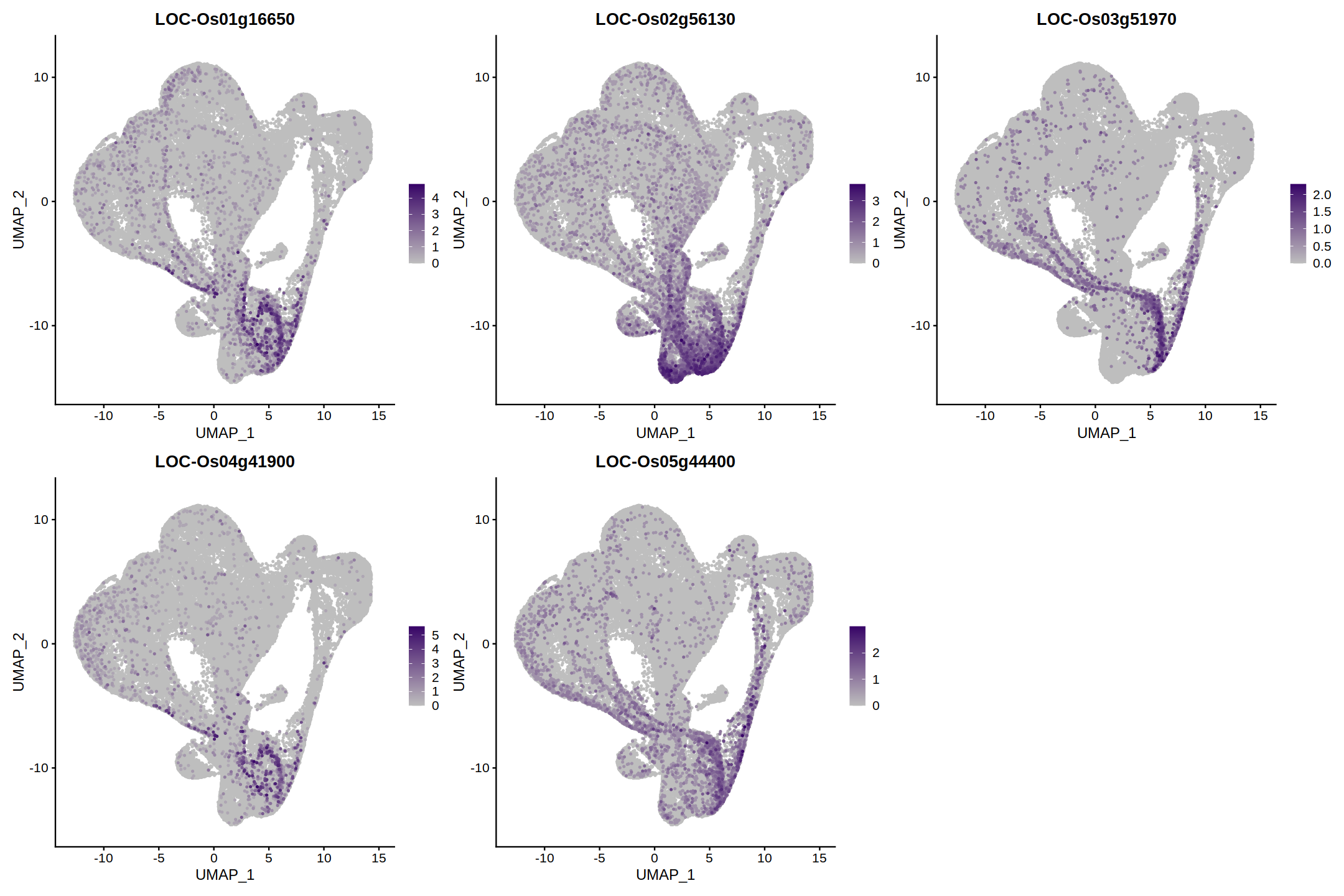

Supplement: Supplementary file 18 — Combined feature plots representing the expression patterns of cell type markers in single-cell RNA-seq data. Each image represents the gene expressions of markers for one certain cell type. [file 41586_2025_8941_MOESM18_ESM.zip › Supplementary Data 3_Marker_expressions_in_gel-based_scRNAseq_Rice/Meristem marker expression.png]

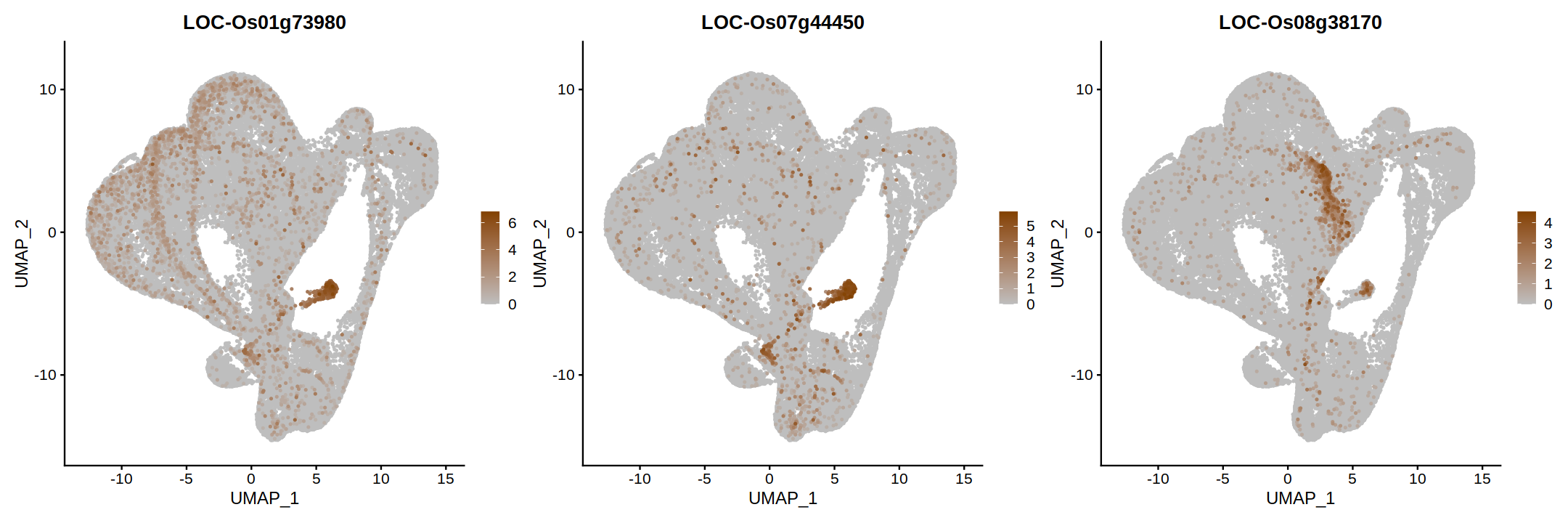

Supplement: Supplementary file 18 — Combined feature plots representing the expression patterns of cell type markers in single-cell RNA-seq data. Each image represents the gene expressions of markers for one certain cell type. [file 41586_2025_8941_MOESM18_ESM.zip › Supplementary Data 3_Marker_expressions_in_gel-based_scRNAseq_Rice/Metaxylem marker expression.png]

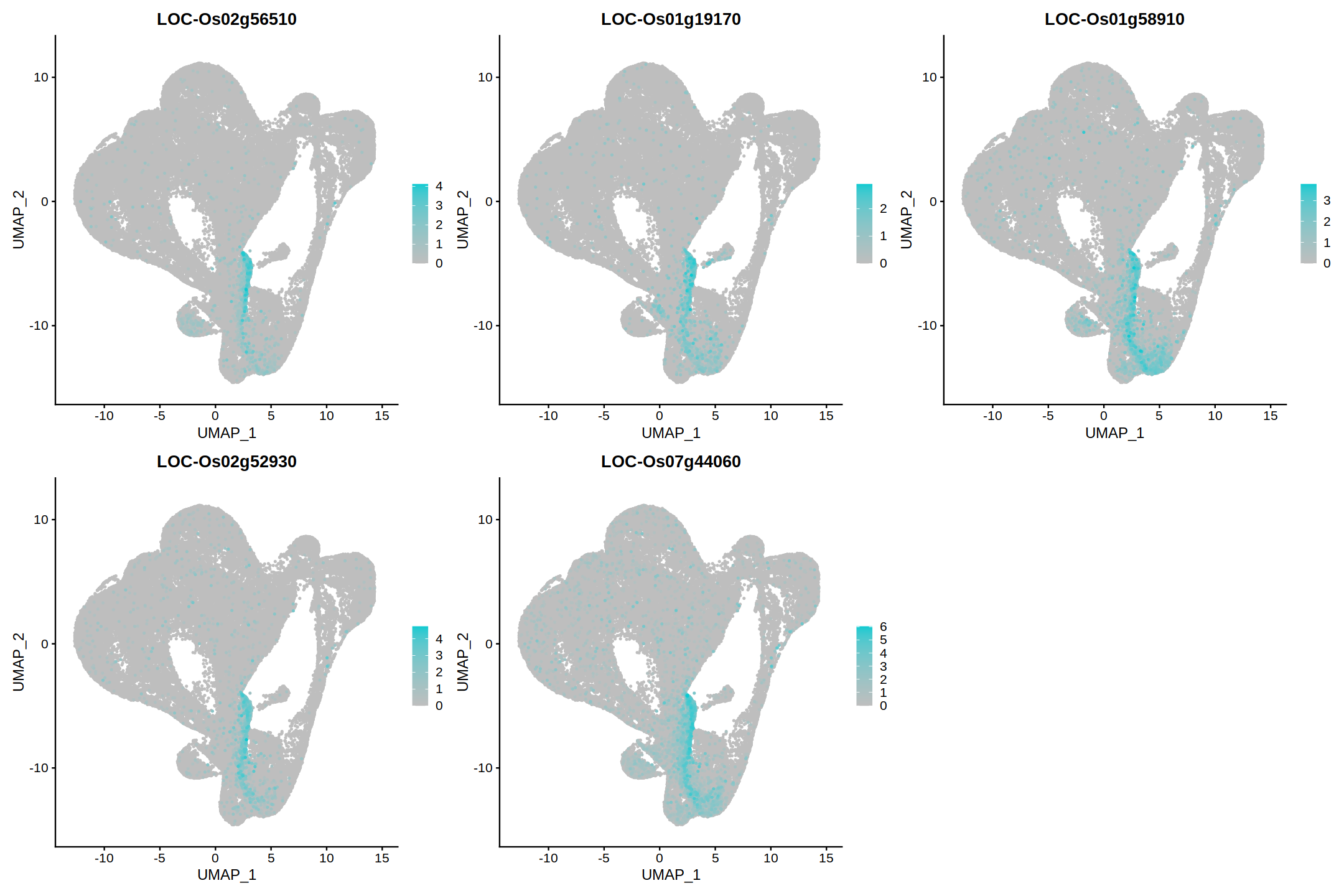

Supplement: Supplementary file 18 — Combined feature plots representing the expression patterns of cell type markers in single-cell RNA-seq data. Each image represents the gene expressions of markers for one certain cell type. [file 41586_2025_8941_MOESM18_ESM.zip › Supplementary Data 3_Marker_expressions_in_gel-based_scRNAseq_Rice/Pericycle marker expression.png]

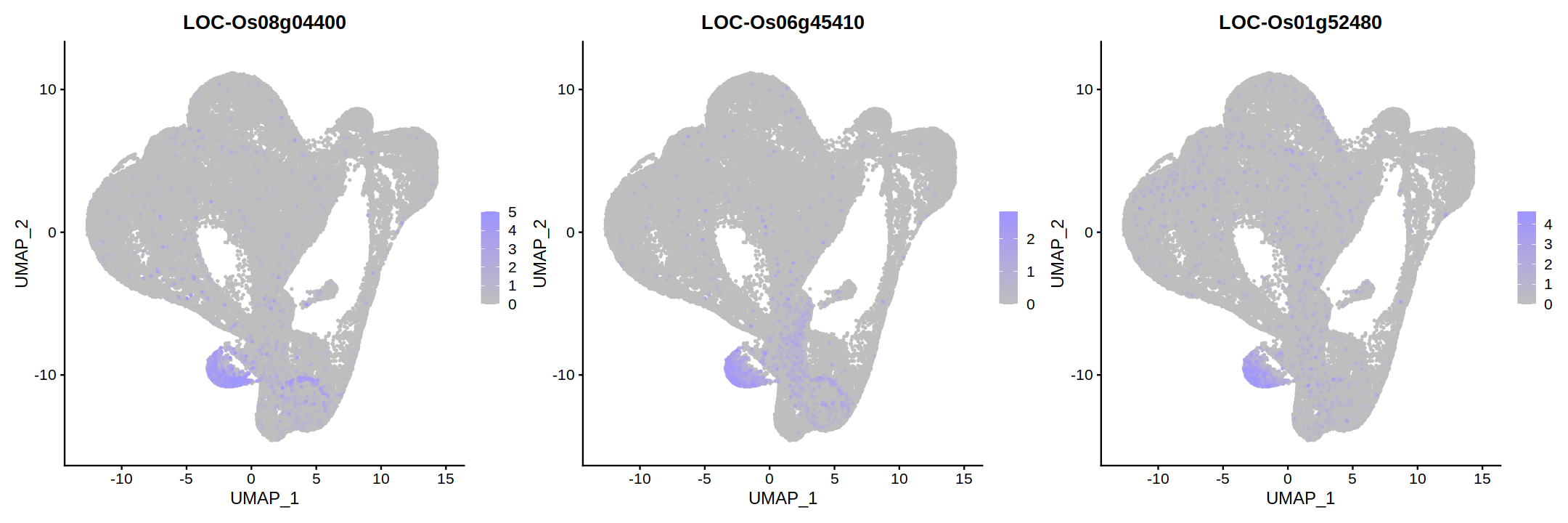

Supplement: Supplementary file 18 — Combined feature plots representing the expression patterns of cell type markers in single-cell RNA-seq data. Each image represents the gene expressions of markers for one certain cell type. [file 41586_2025_8941_MOESM18_ESM.zip › Supplementary Data 3_Marker_expressions_in_gel-based_scRNAseq_Rice/Phloem marker expression.png]

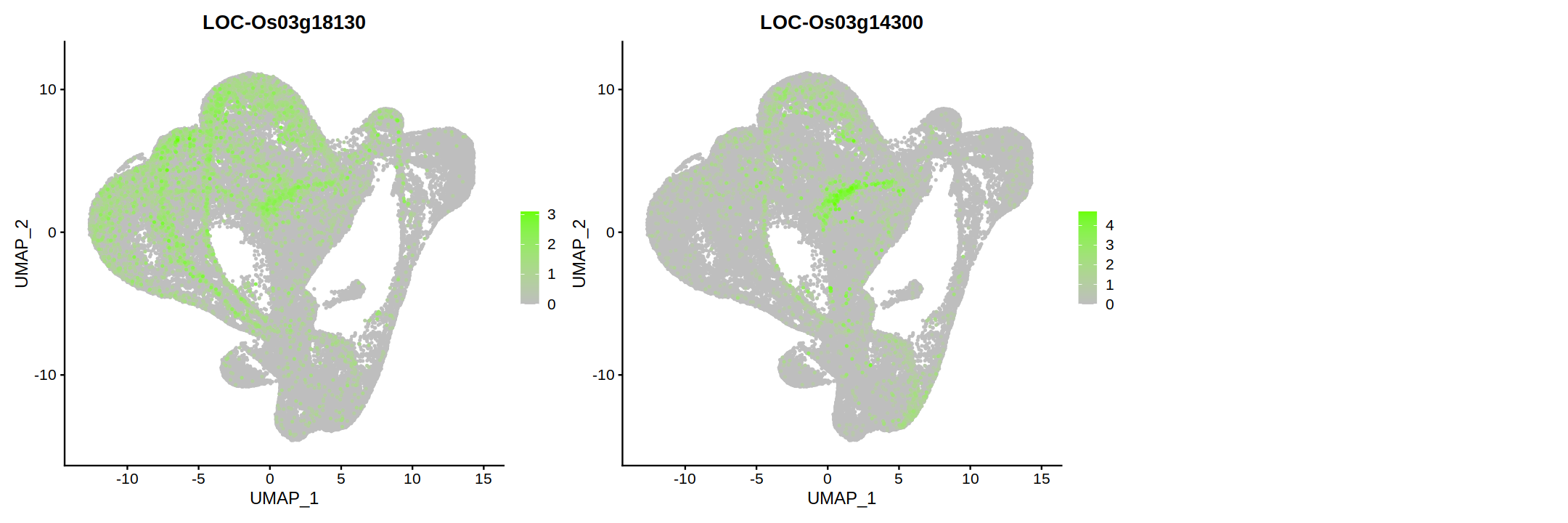

Supplement: Supplementary file 18 — Combined feature plots representing the expression patterns of cell type markers in single-cell RNA-seq data. Each image represents the gene expressions of markers for one certain cell type. [file 41586_2025_8941_MOESM18_ESM.zip › Supplementary Data 3_Marker_expressions_in_gel-based_scRNAseq_Rice/Root cap marker expression.png]

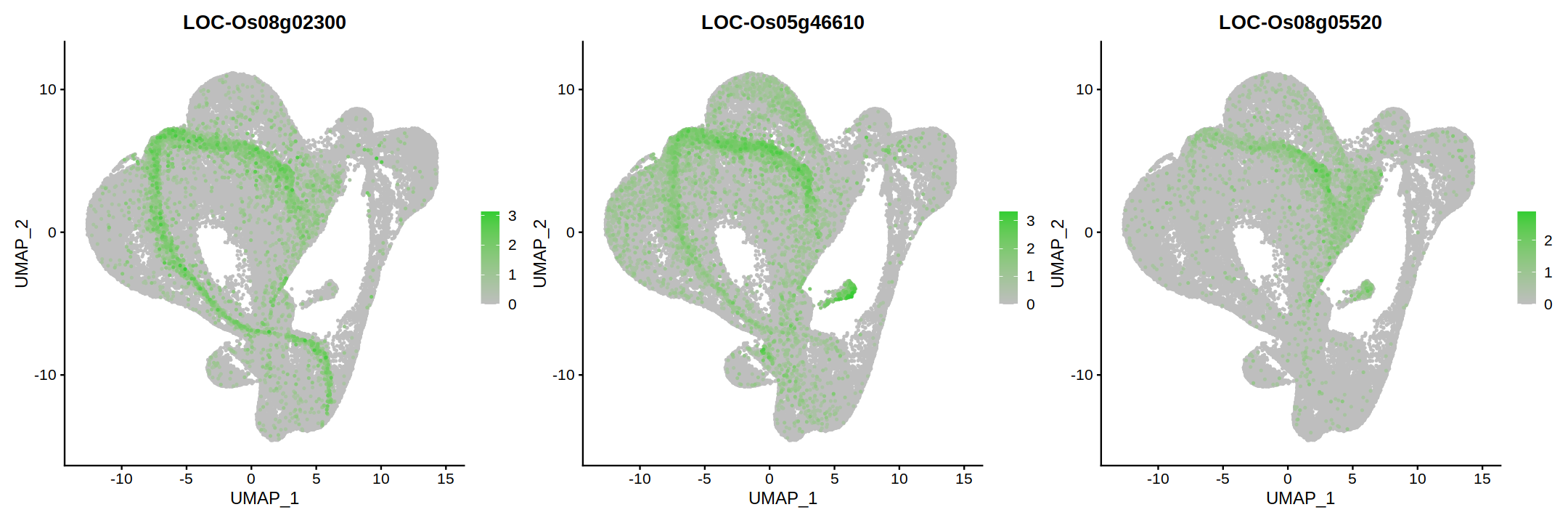

Supplement: Supplementary file 18 — Combined feature plots representing the expression patterns of cell type markers in single-cell RNA-seq data. Each image represents the gene expressions of markers for one certain cell type. [file 41586_2025_8941_MOESM18_ESM.zip › Supplementary Data 3_Marker_expressions_in_gel-based_scRNAseq_Rice/Sclerenchyma marker expression.png]

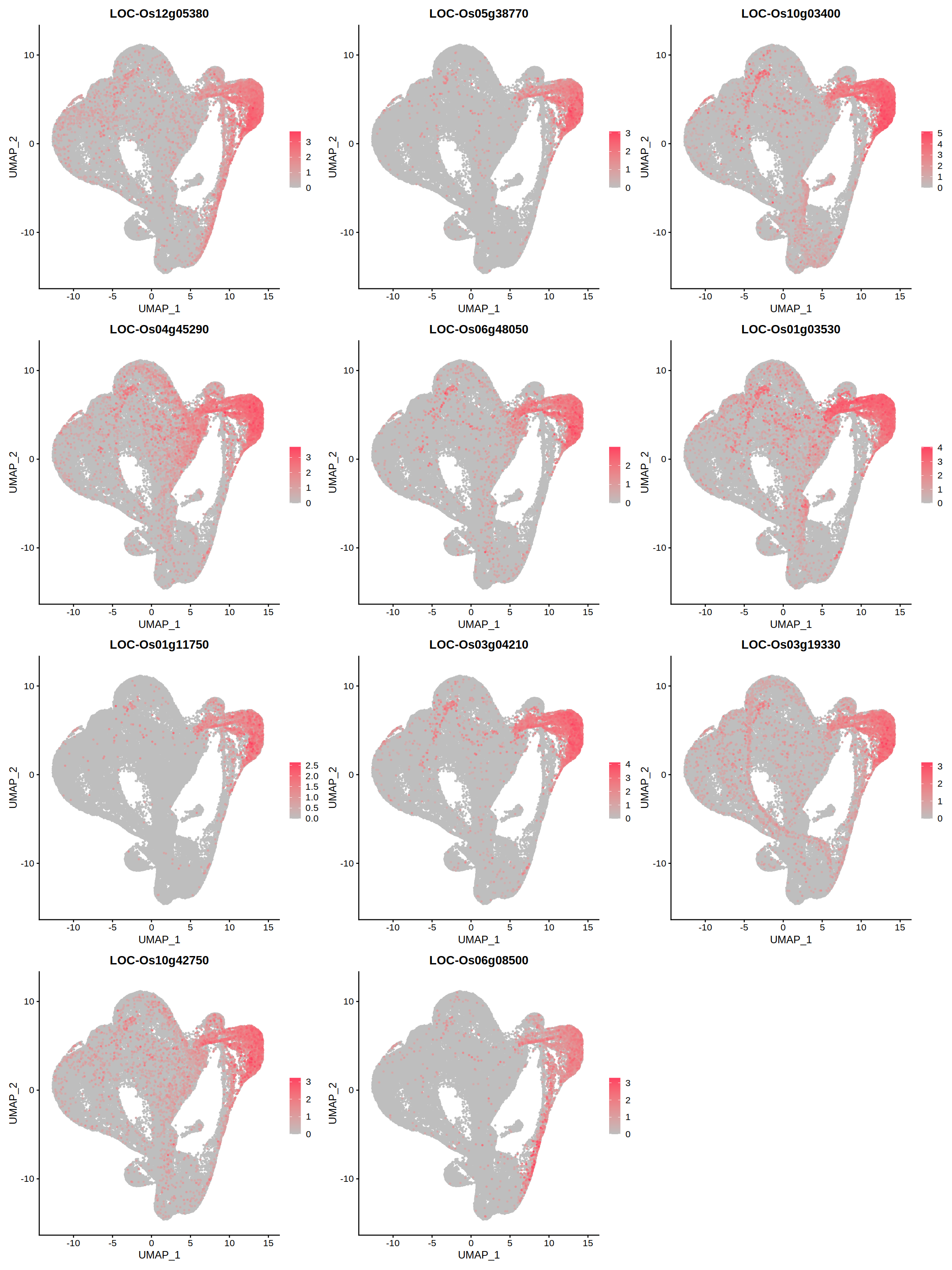

Supplement: Supplementary file 18 — Combined feature plots representing the expression patterns of cell type markers in single-cell RNA-seq data. Each image represents the gene expressions of markers for one certain cell type. [file 41586_2025_8941_MOESM18_ESM.zip › Supplementary Data 3_Marker_expressions_in_gel-based_scRNAseq_Rice/Trichoblast marker expression.png]

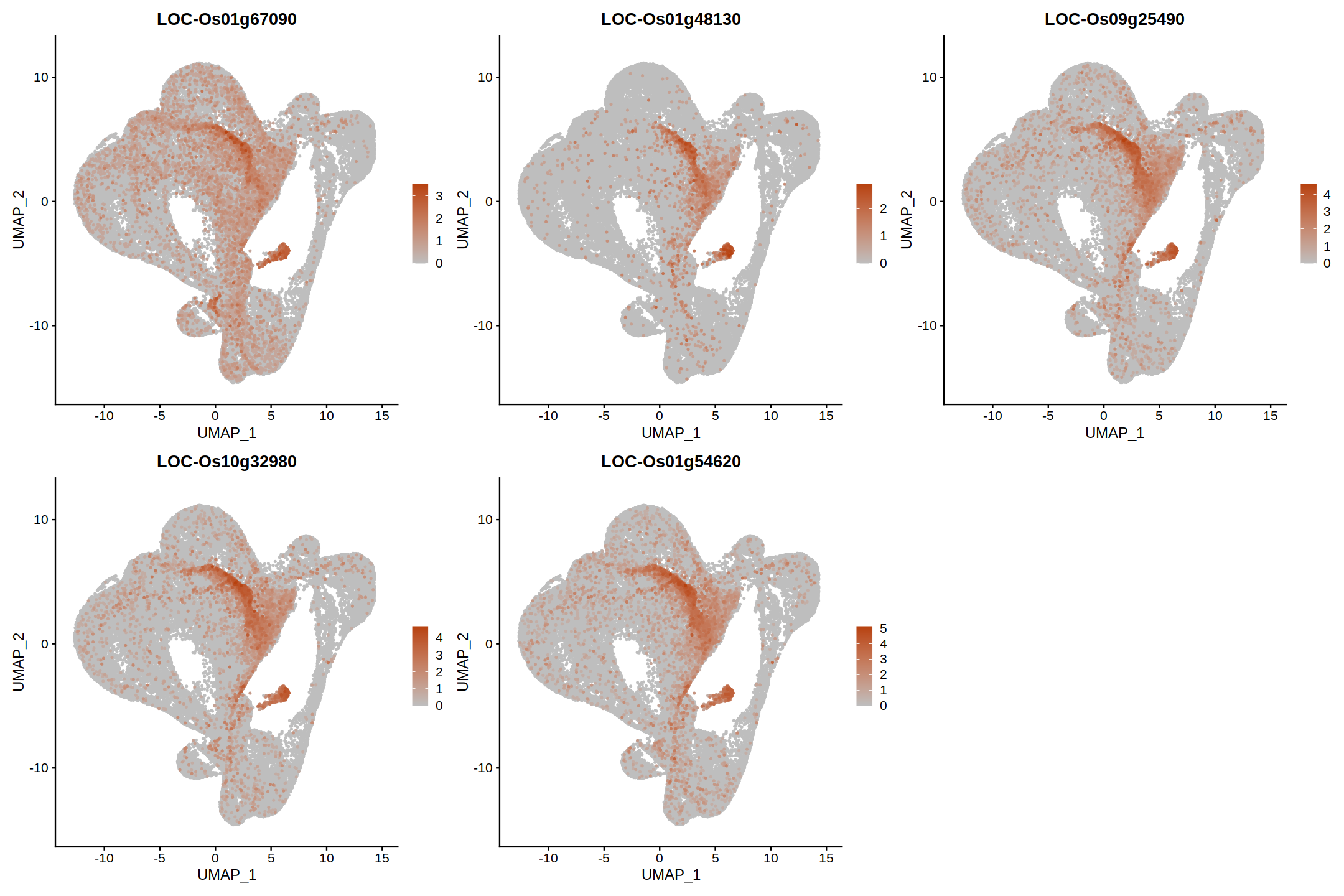

Supplement: Supplementary file 18 — Combined feature plots representing the expression patterns of cell type markers in single-cell RNA-seq data. Each image represents the gene expressions of markers for one certain cell type. [file 41586_2025_8941_MOESM18_ESM.zip › Supplementary Data 3_Marker_expressions_in_gel-based_scRNAseq_Rice/Xylem marker expression.png]

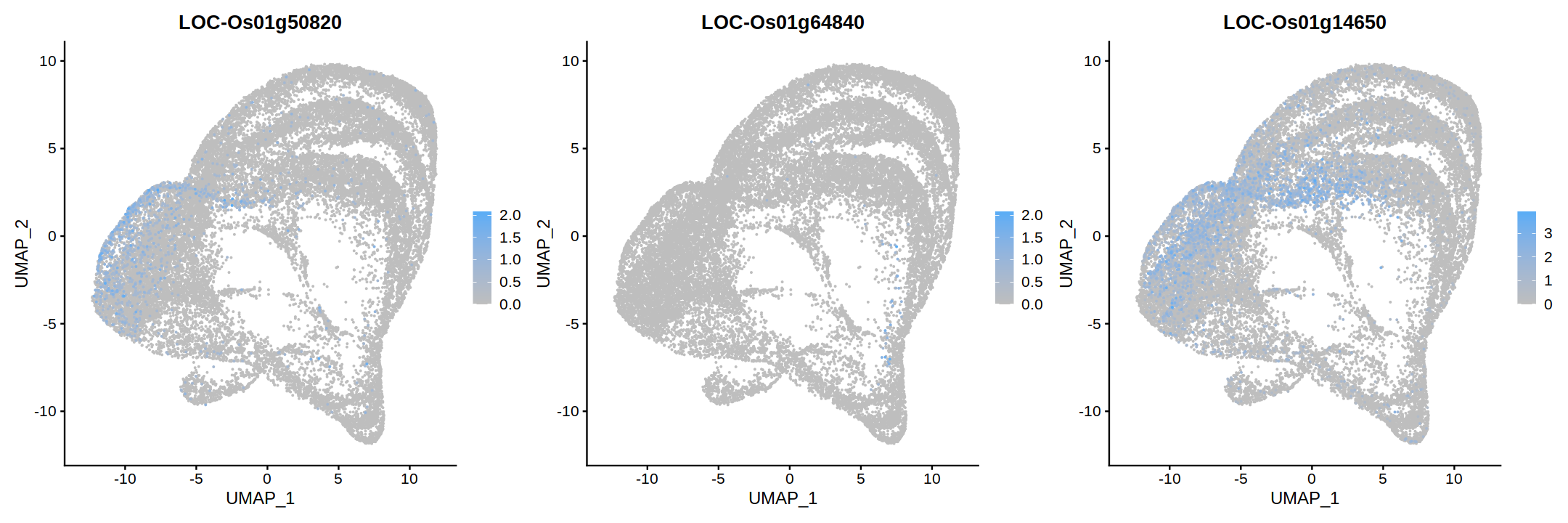

Supplement: Supplementary file 20 — Combined Feature plots representing the expression patterns of cell type markers in single-cell RNA-seq data for non-compacted soil grown roots. Each image represents the gene expressions of markers for one certain cell type. [file 41586_2025_8941_MOESM20_ESM.zip › Supplementary Data 5_Marker_expressions_in_non-compacted-soils-based_scRNAseq_Rice/Atrichoblast-noncompacted.png]

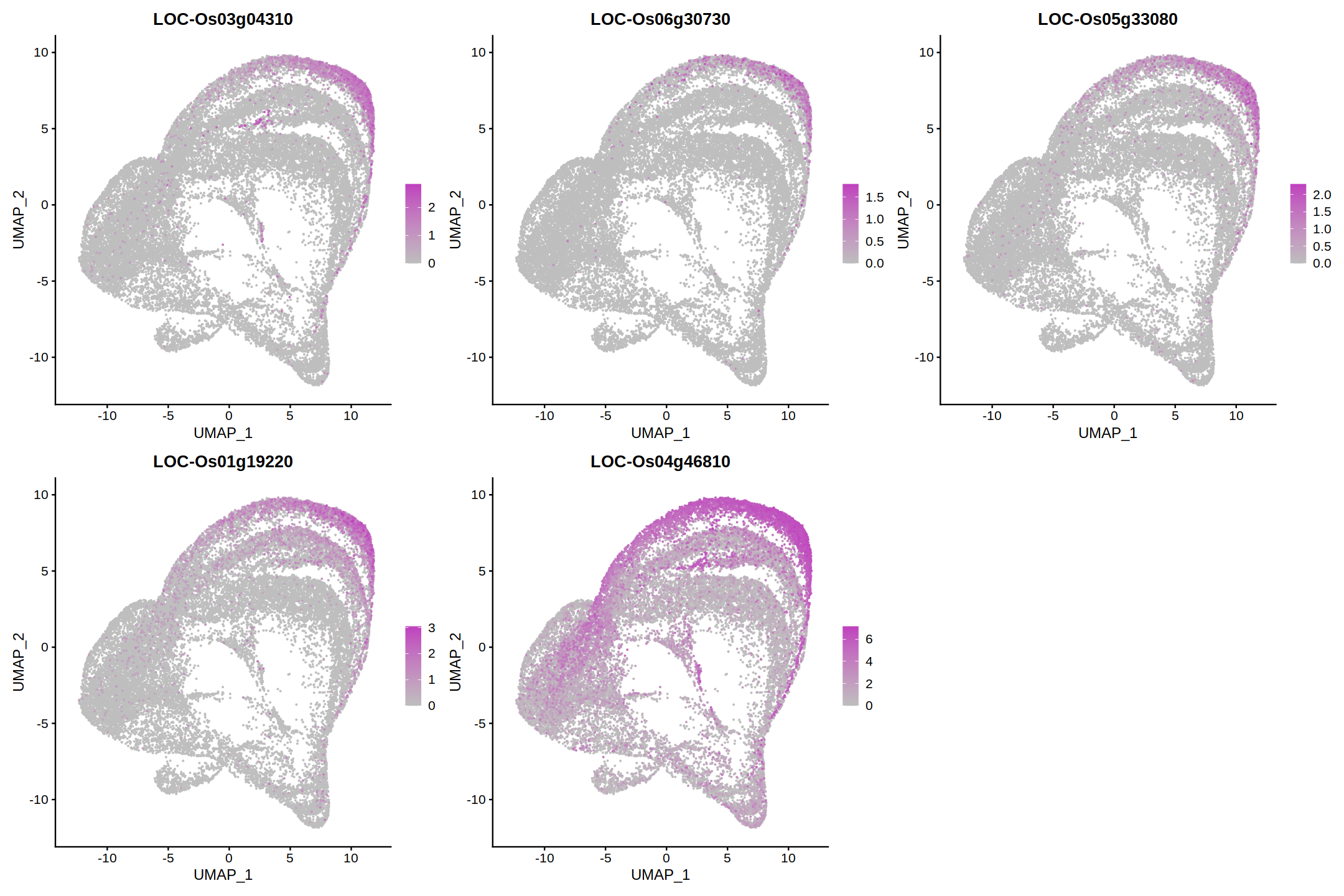

Supplement: Supplementary file 20 — Combined Feature plots representing the expression patterns of cell type markers in single-cell RNA-seq data for non-compacted soil grown roots. Each image represents the gene expressions of markers for one certain cell type. [file 41586_2025_8941_MOESM20_ESM.zip › Supplementary Data 5_Marker_expressions_in_non-compacted-soils-based_scRNAseq_Rice/Cortex-noncompacted.png]

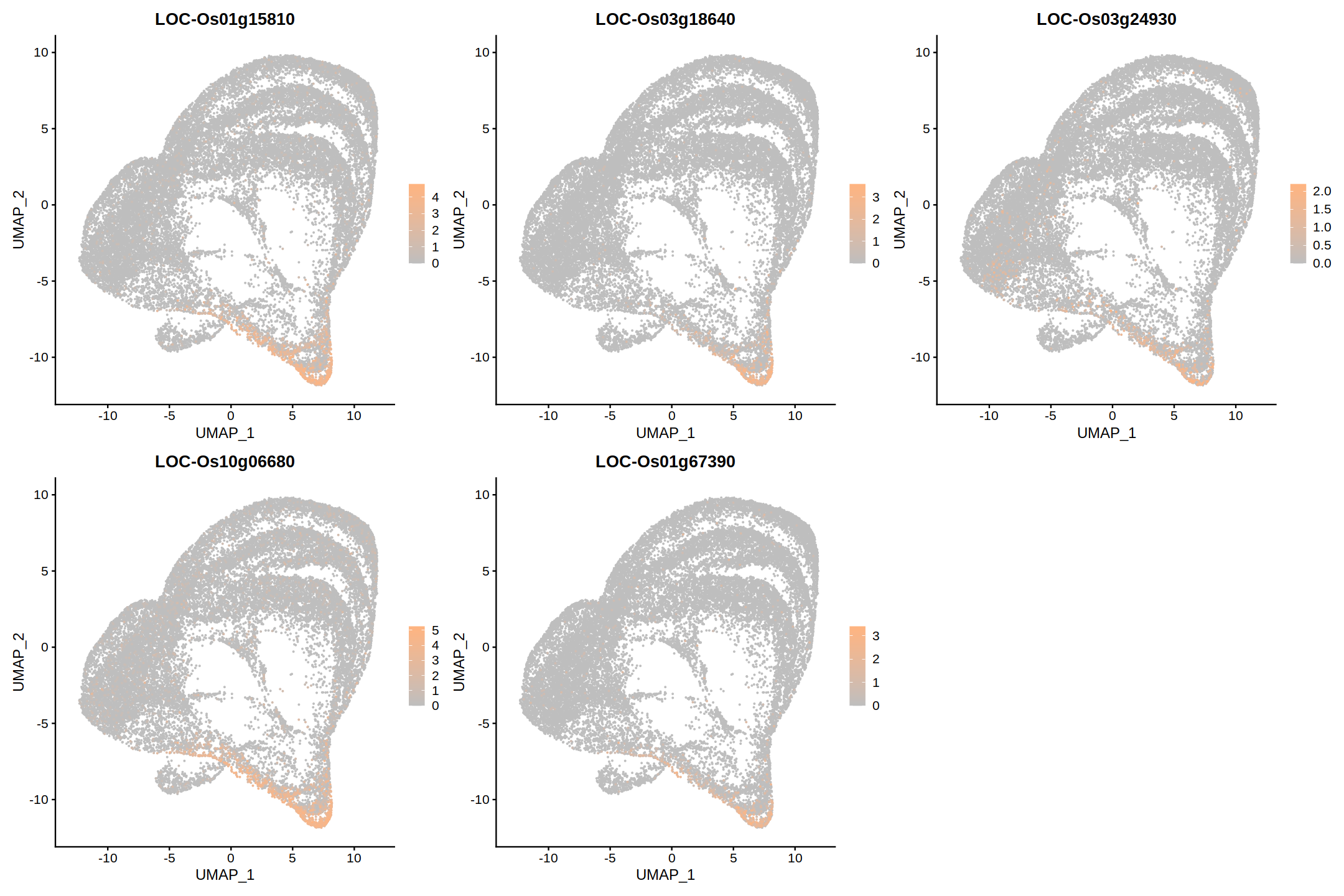

Supplement: Supplementary file 20 — Combined Feature plots representing the expression patterns of cell type markers in single-cell RNA-seq data for non-compacted soil grown roots. Each image represents the gene expressions of markers for one certain cell type. [file 41586_2025_8941_MOESM20_ESM.zip › Supplementary Data 5_Marker_expressions_in_non-compacted-soils-based_scRNAseq_Rice/Endodermis-noncompacted.png]

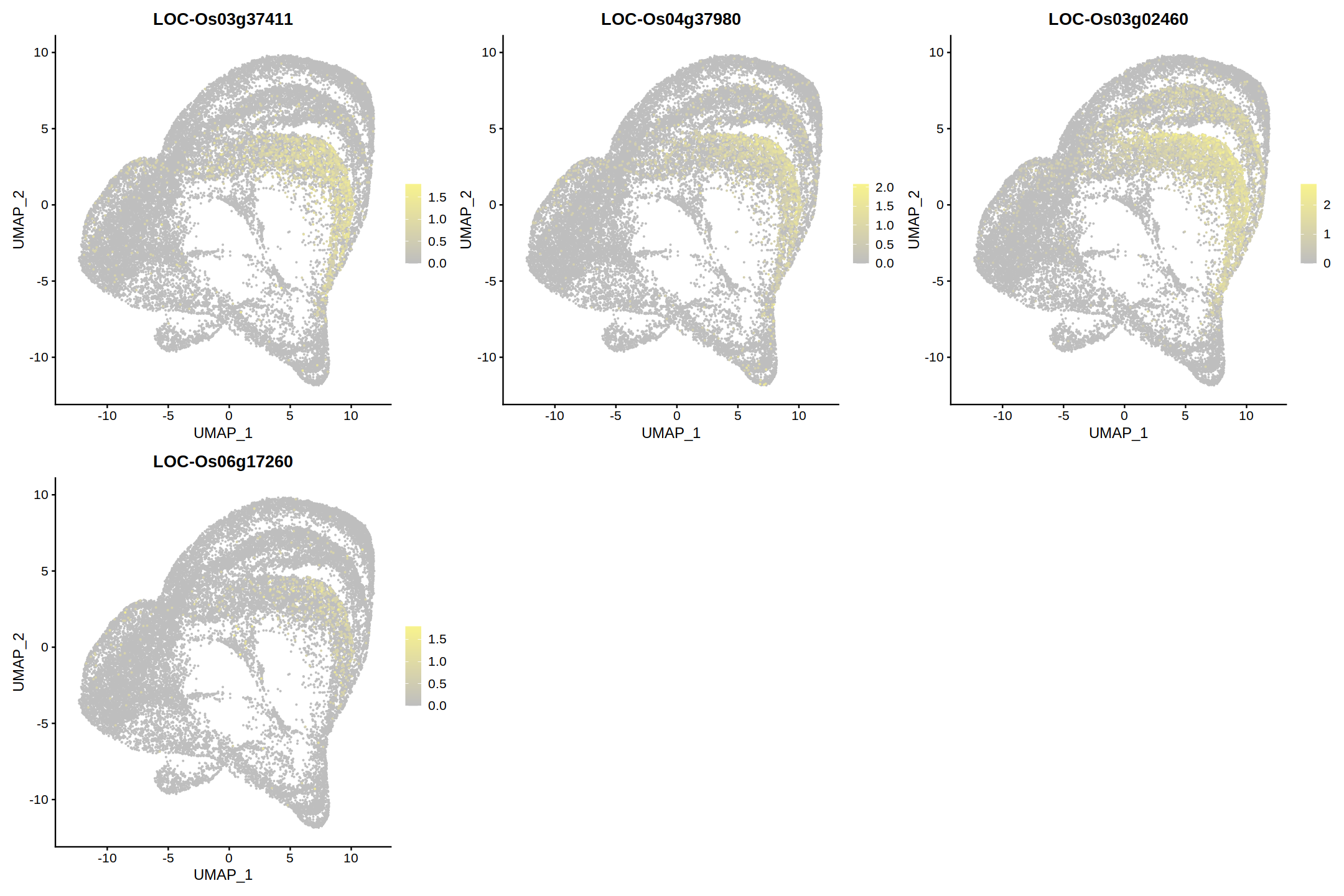

Supplement: Supplementary file 20 — Combined Feature plots representing the expression patterns of cell type markers in single-cell RNA-seq data for non-compacted soil grown roots. Each image represents the gene expressions of markers for one certain cell type. [file 41586_2025_8941_MOESM20_ESM.zip › Supplementary Data 5_Marker_expressions_in_non-compacted-soils-based_scRNAseq_Rice/Exodermis-noncompacted.png]

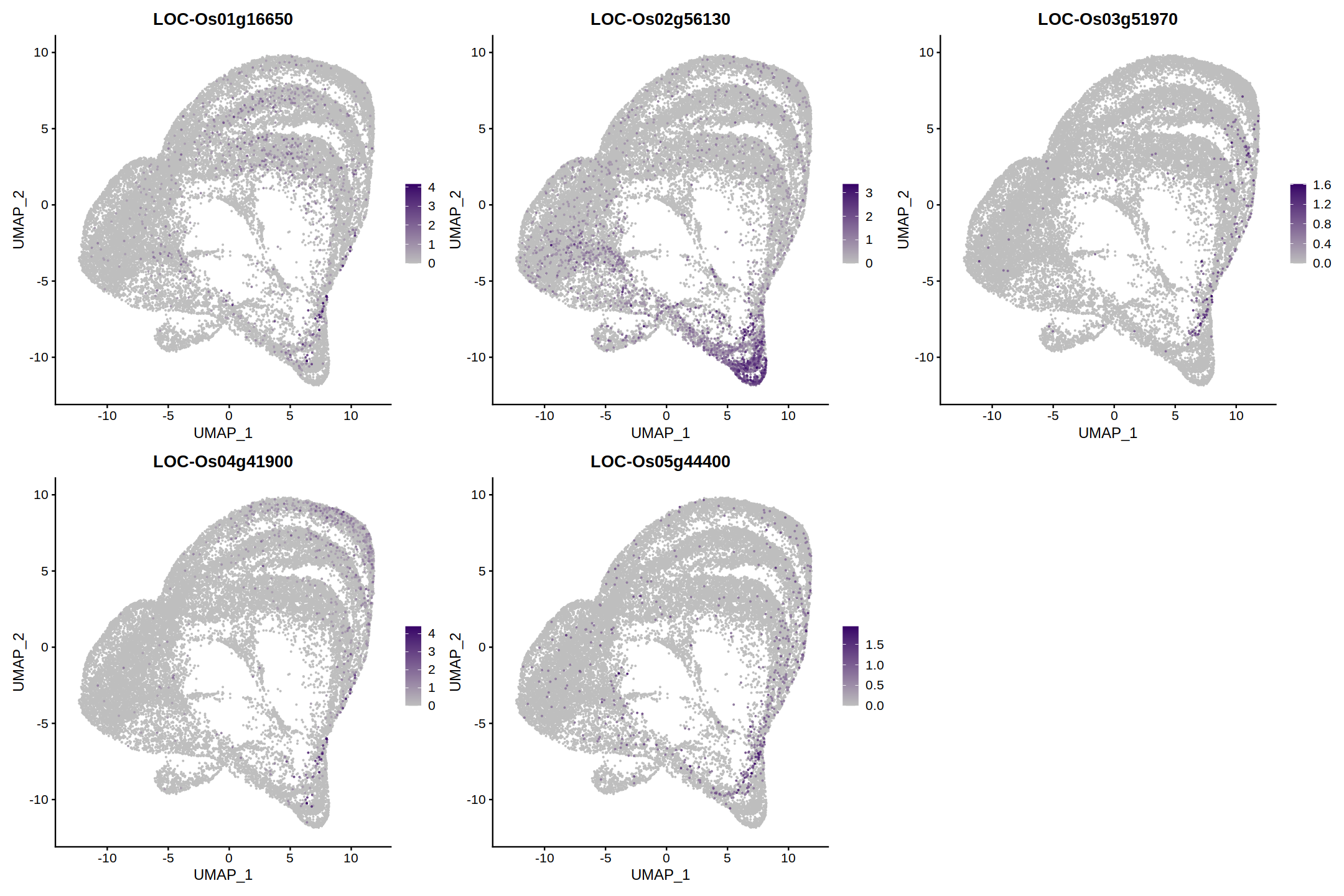

Supplement: Supplementary file 20 — Combined Feature plots representing the expression patterns of cell type markers in single-cell RNA-seq data for non-compacted soil grown roots. Each image represents the gene expressions of markers for one certain cell type. [file 41586_2025_8941_MOESM20_ESM.zip › Supplementary Data 5_Marker_expressions_in_non-compacted-soils-based_scRNAseq_Rice/Meristem marker-noncompacted.png]

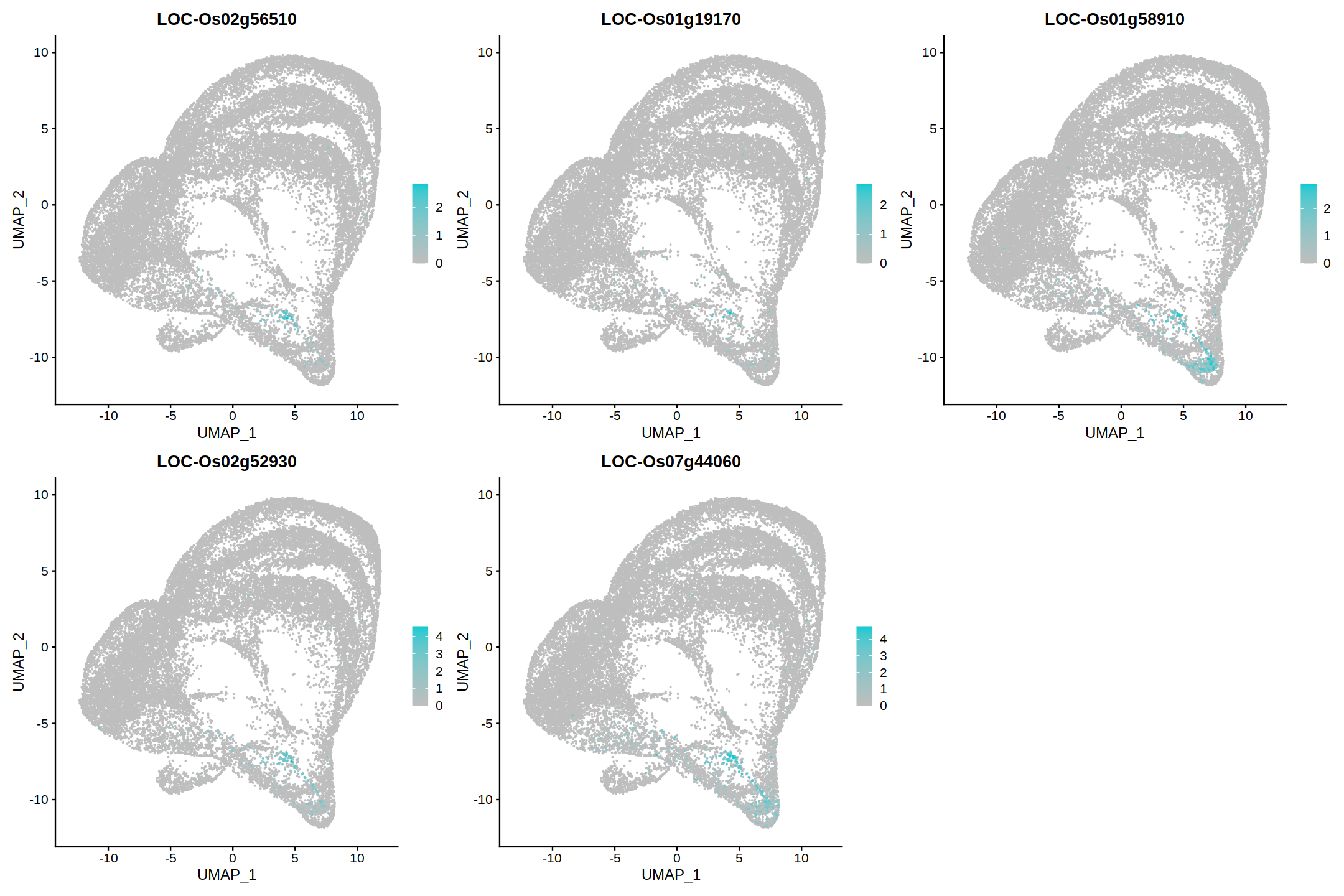

Supplement: Supplementary file 20 — Combined Feature plots representing the expression patterns of cell type markers in single-cell RNA-seq data for non-compacted soil grown roots. Each image represents the gene expressions of markers for one certain cell type. [file 41586_2025_8941_MOESM20_ESM.zip › Supplementary Data 5_Marker_expressions_in_non-compacted-soils-based_scRNAseq_Rice/Pericycle-noncompacted.png]

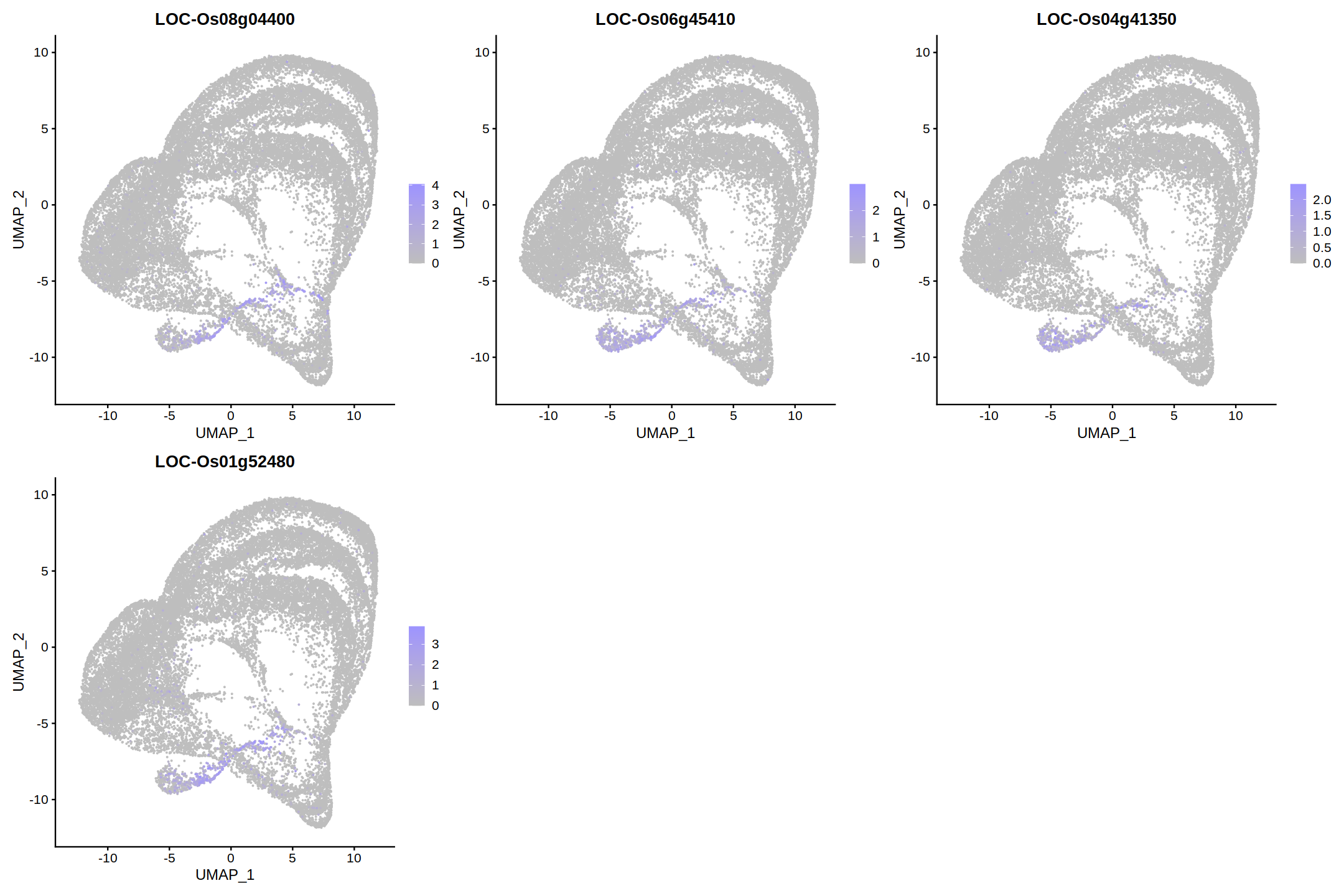

Supplement: Supplementary file 20 — Combined Feature plots representing the expression patterns of cell type markers in single-cell RNA-seq data for non-compacted soil grown roots. Each image represents the gene expressions of markers for one certain cell type. [file 41586_2025_8941_MOESM20_ESM.zip › Supplementary Data 5_Marker_expressions_in_non-compacted-soils-based_scRNAseq_Rice/Phloem-noncompacted.png]

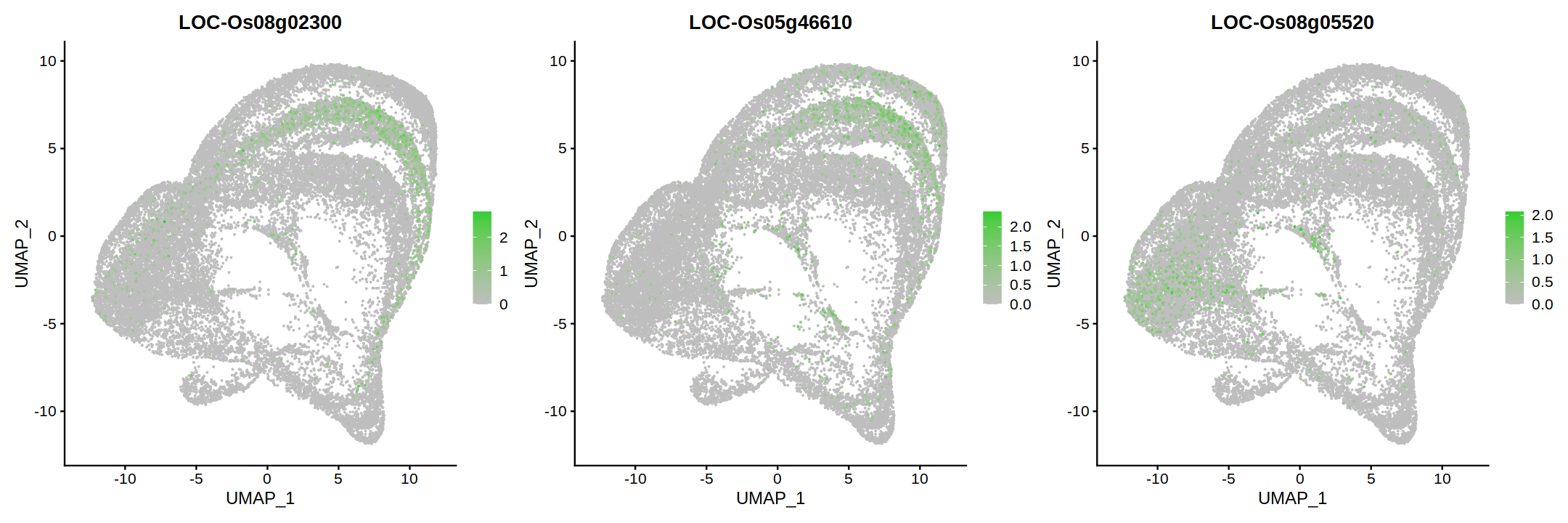

Supplement: Supplementary file 20 — Combined Feature plots representing the expression patterns of cell type markers in single-cell RNA-seq data for non-compacted soil grown roots. Each image represents the gene expressions of markers for one certain cell type. [file 41586_2025_8941_MOESM20_ESM.zip › Supplementary Data 5_Marker_expressions_in_non-compacted-soils-based_scRNAseq_Rice/Sclerenchyma-noncompacted.png]

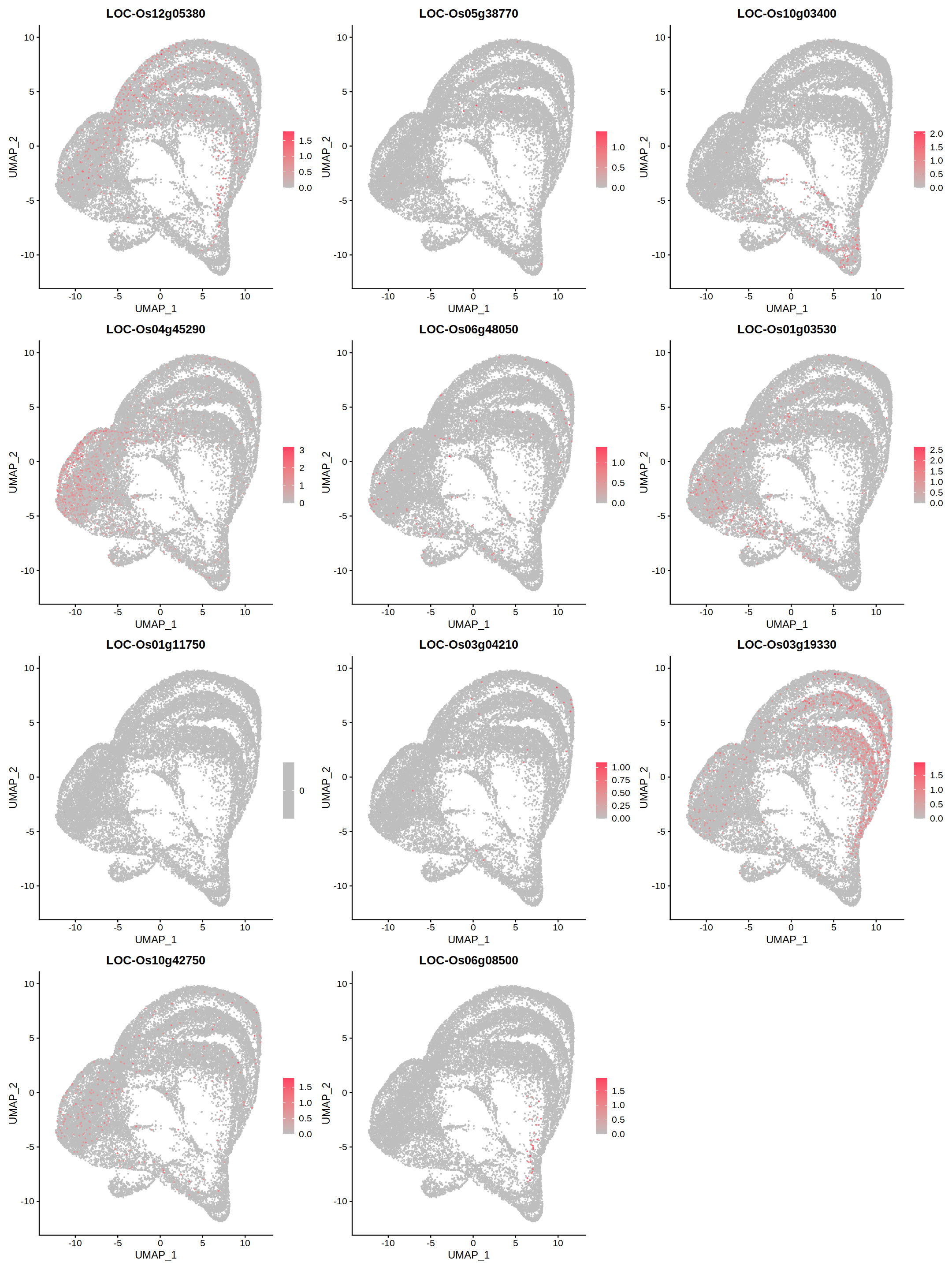

Supplement: Supplementary file 20 — Combined Feature plots representing the expression patterns of cell type markers in single-cell RNA-seq data for non-compacted soil grown roots. Each image represents the gene expressions of markers for one certain cell type. [file 41586_2025_8941_MOESM20_ESM.zip › Supplementary Data 5_Marker_expressions_in_non-compacted-soils-based_scRNAseq_Rice/Trichoblast-noncompacted.png]

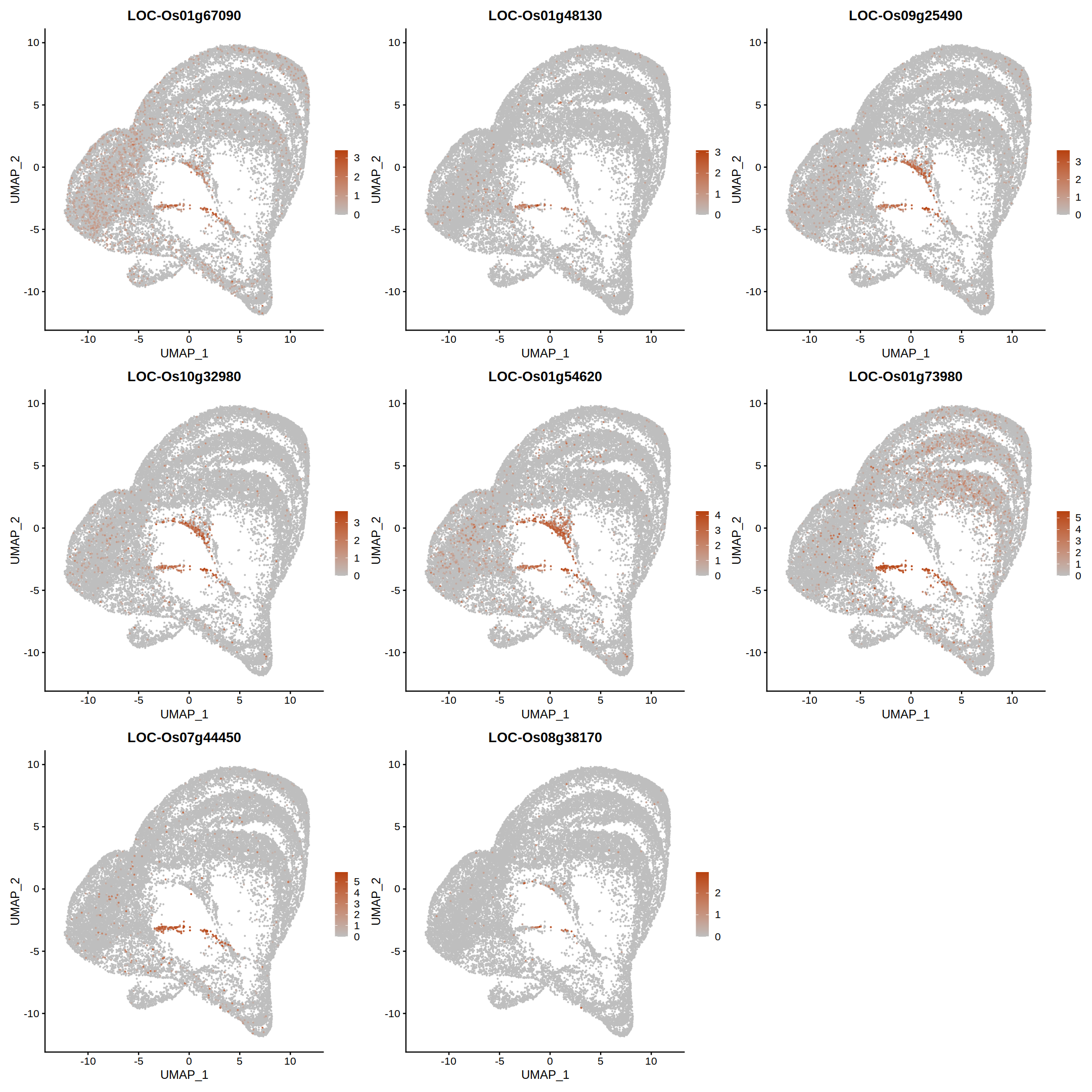

Supplement: Supplementary file 20 — Combined Feature plots representing the expression patterns of cell type markers in single-cell RNA-seq data for non-compacted soil grown roots. Each image represents the gene expressions of markers for one certain cell type. [file 41586_2025_8941_MOESM20_ESM.zip › Supplementary Data 5_Marker_expressions_in_non-compacted-soils-based_scRNAseq_Rice/Xylem-noncompacted.png]

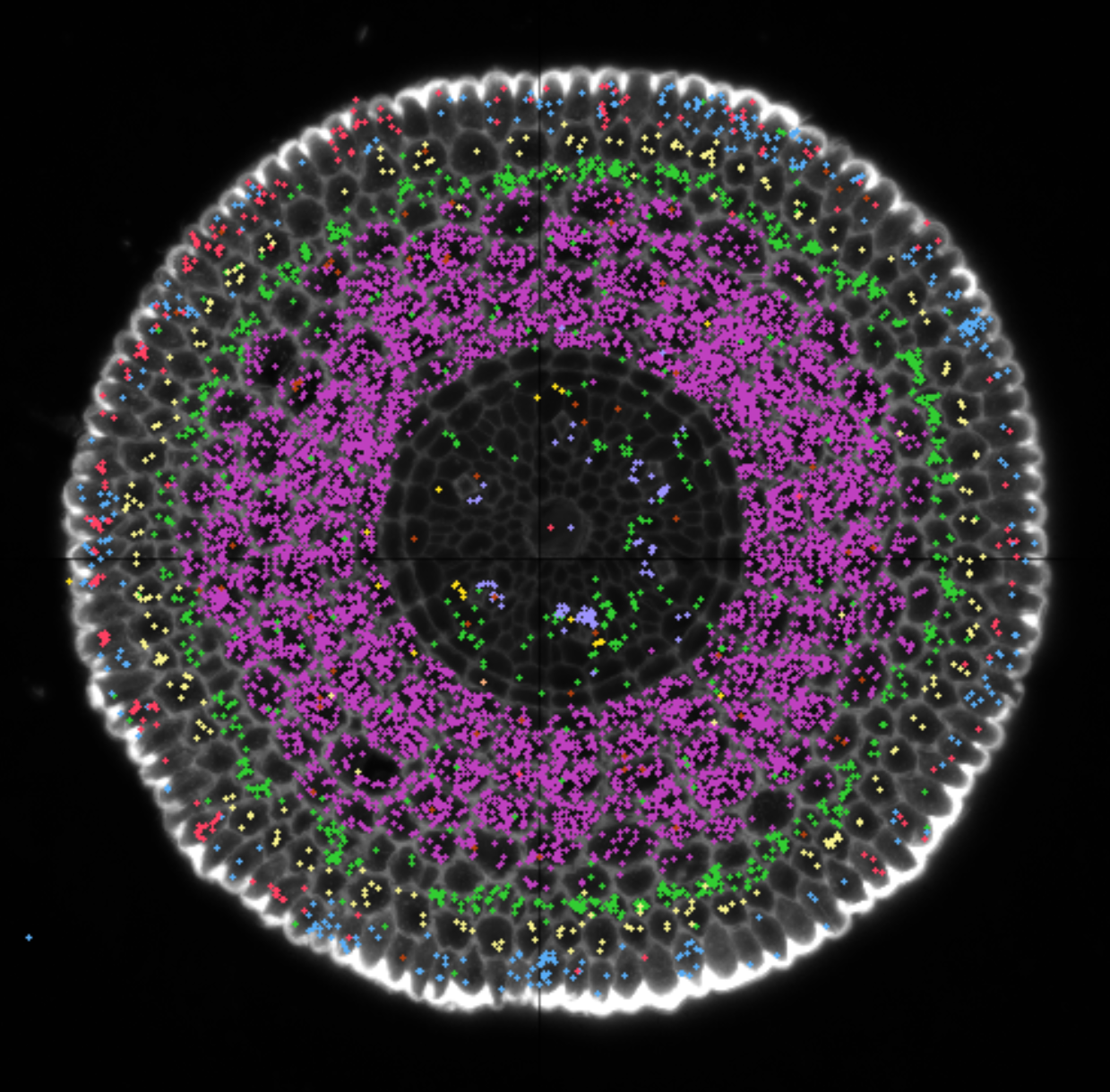

Supplement: Supplementary file 21 — Expression patterns of cell type markers in spatial transcriptomics data for non-compacted soil grown roots. A PDF summary file that includes the sample and gene information for visualization is included. The raw spatial transcriptomics data for non-compacted soil grown roots is also included. [file 41586_2025_8941_MOESM21_ESM.zip › Supplementary Data 6_Marker_expression_in_non-compacted-soils-based_Spatial_transcriptomics_Rice/All selected good markers.png]

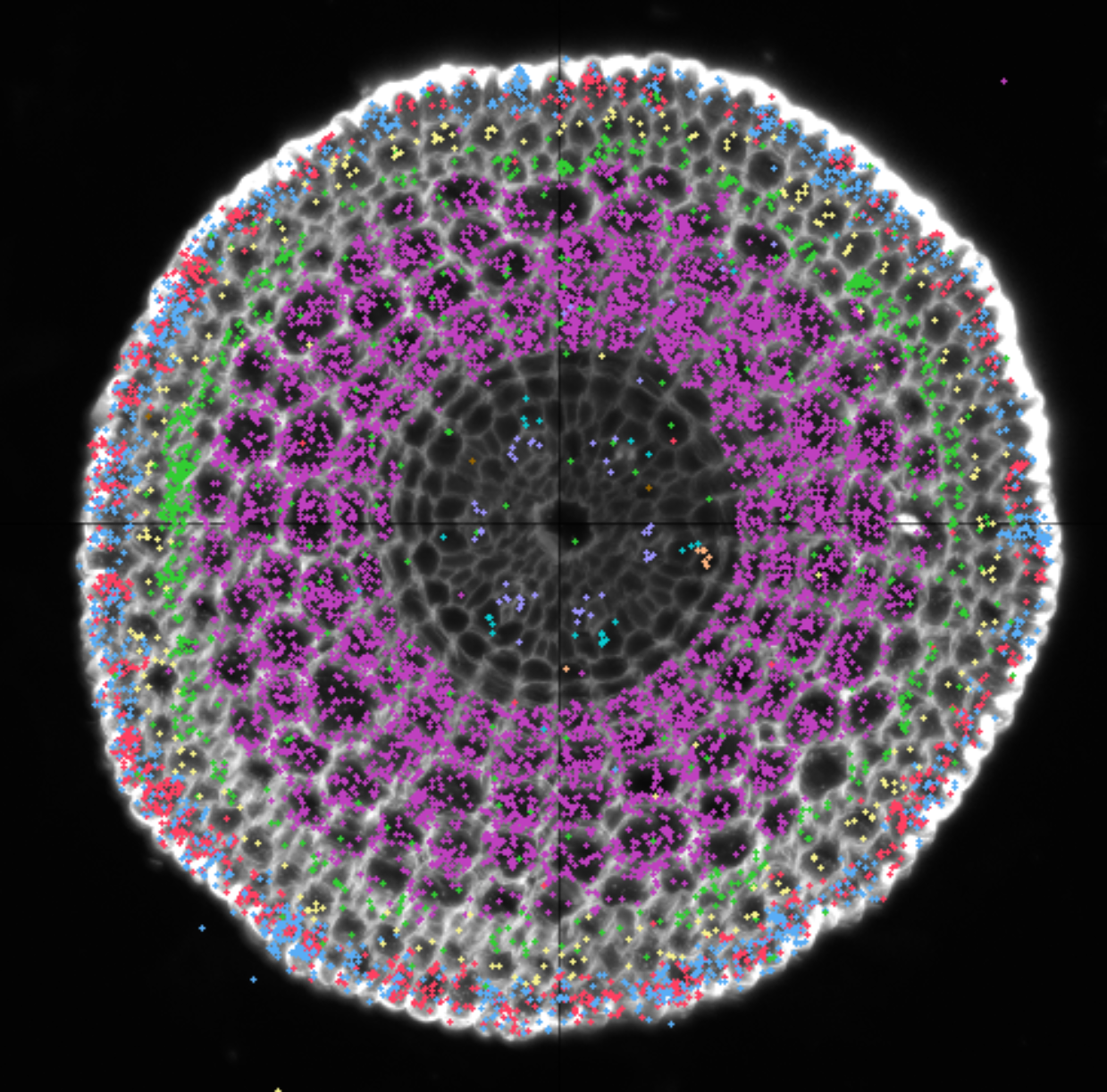

Supplement: Supplementary file 21 — Expression patterns of cell type markers in spatial transcriptomics data for non-compacted soil grown roots. A PDF summary file that includes the sample and gene information for visualization is included. The raw spatial transcriptomics data for non-compacted soil grown roots is also included. [file 41586_2025_8941_MOESM21_ESM.zip › Supplementary Data 6_Marker_expression_in_non-compacted-soils-based_Spatial_transcriptomics_Rice/All selected good markers-2.png]

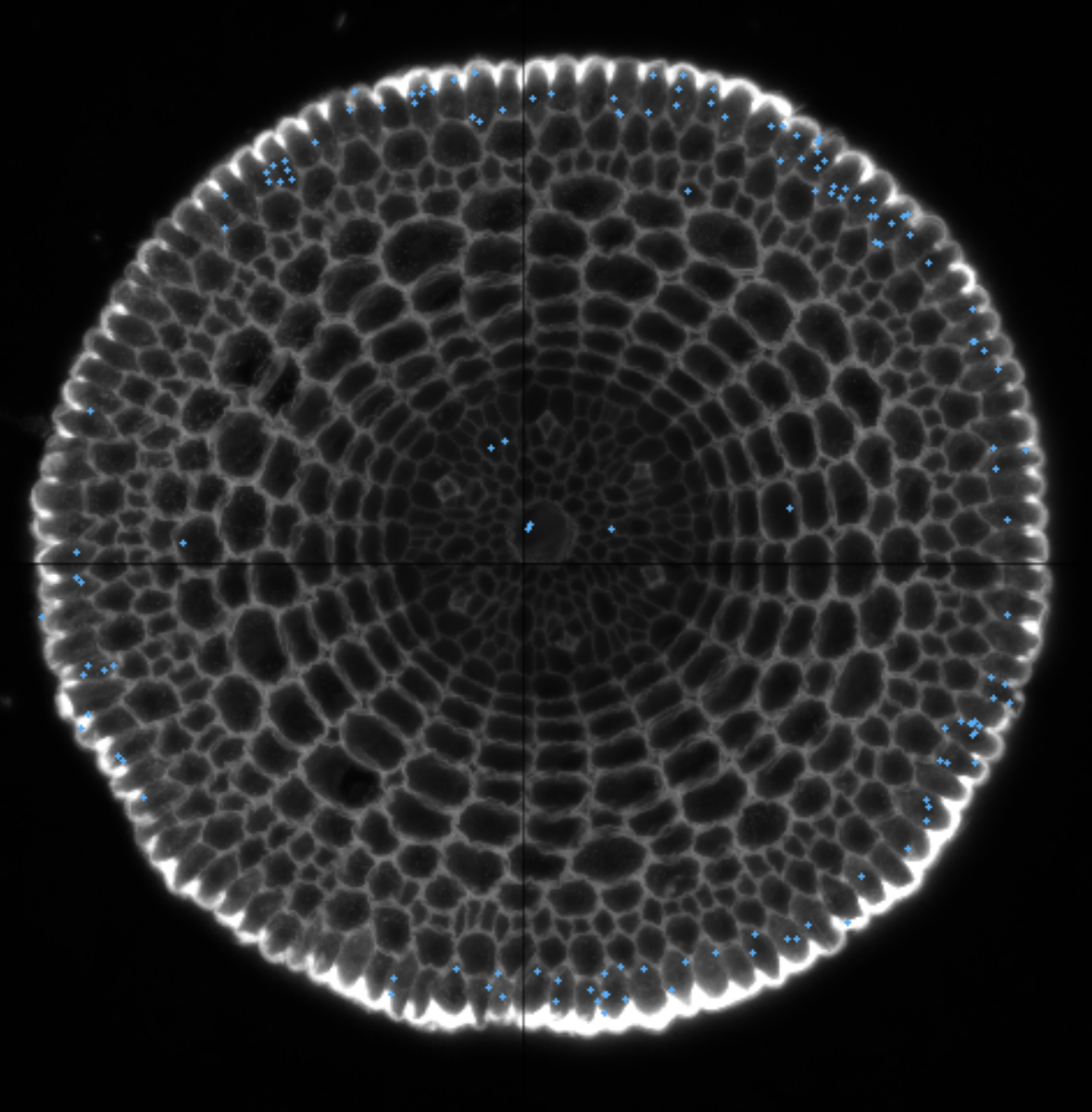

Supplement: Supplementary file 21 — Expression patterns of cell type markers in spatial transcriptomics data for non-compacted soil grown roots. A PDF summary file that includes the sample and gene information for visualization is included. The raw spatial transcriptomics data for non-compacted soil grown roots is also included. [file 41586_2025_8941_MOESM21_ESM.zip › Supplementary Data 6_Marker_expression_in_non-compacted-soils-based_Spatial_transcriptomics_Rice/Atrichoblast-LOC_Os01g50820.png]

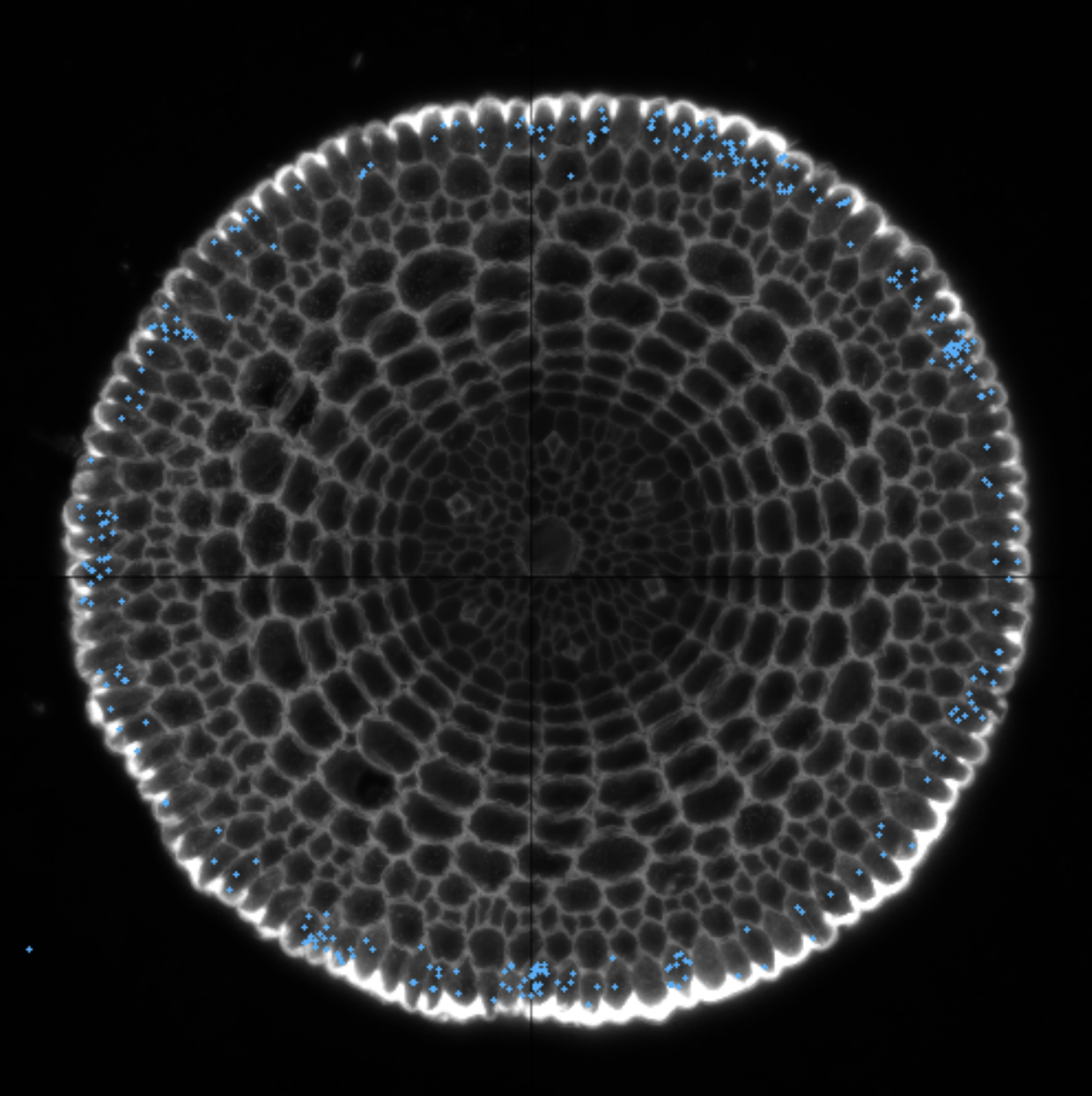

Supplement: Supplementary file 21 — Expression patterns of cell type markers in spatial transcriptomics data for non-compacted soil grown roots. A PDF summary file that includes the sample and gene information for visualization is included. The raw spatial transcriptomics data for non-compacted soil grown roots is also included. [file 41586_2025_8941_MOESM21_ESM.zip › Supplementary Data 6_Marker_expression_in_non-compacted-soils-based_Spatial_transcriptomics_Rice/Atrichoblast-LOC_Os01g64840.png]

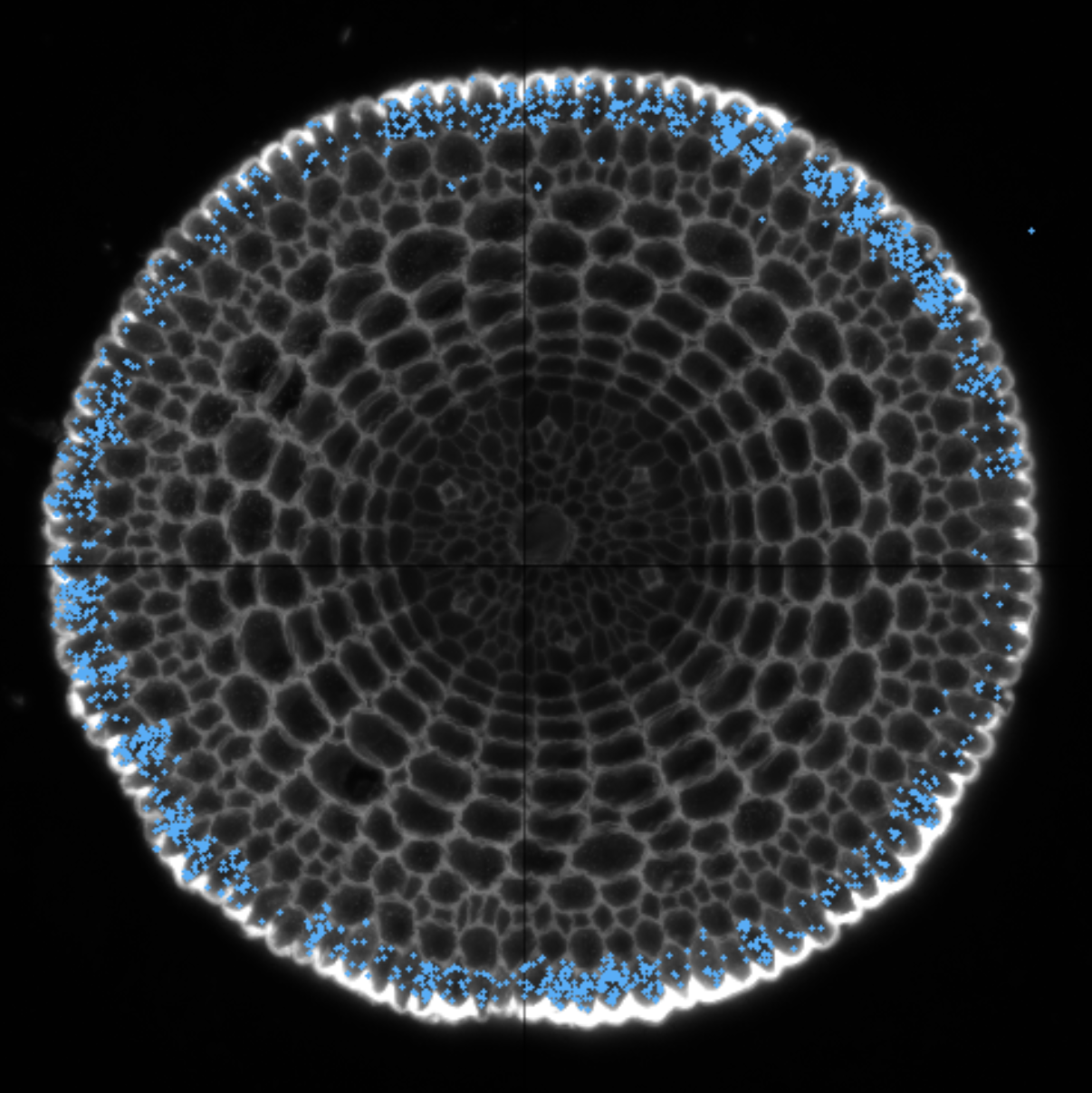

Supplement: Supplementary file 21 — Expression patterns of cell type markers in spatial transcriptomics data for non-compacted soil grown roots. A PDF summary file that includes the sample and gene information for visualization is included. The raw spatial transcriptomics data for non-compacted soil grown roots is also included. [file 41586_2025_8941_MOESM21_ESM.zip › Supplementary Data 6_Marker_expression_in_non-compacted-soils-based_Spatial_transcriptomics_Rice/Atrichoblast-LOC_Os03g09970.png]

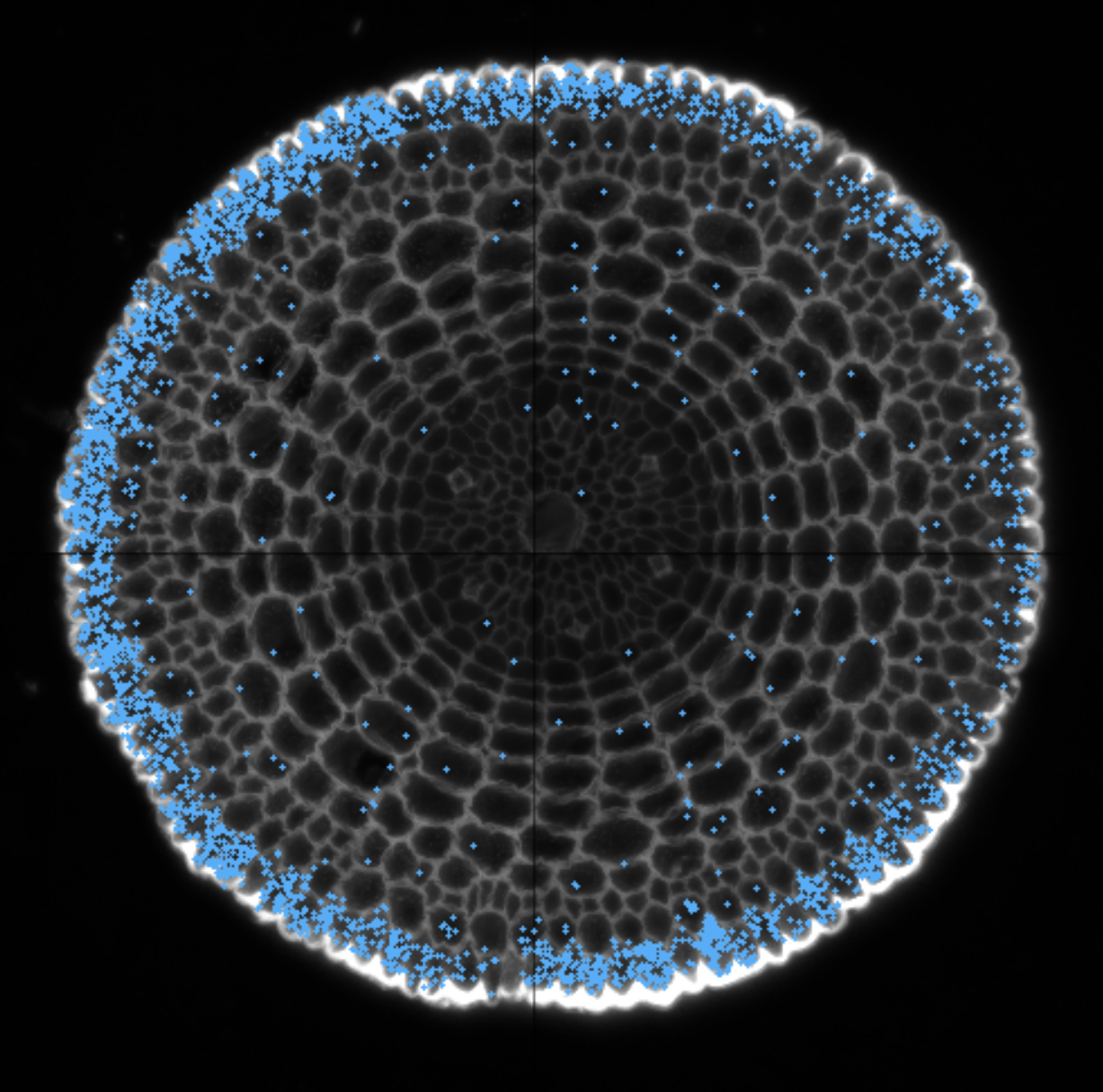

Supplement: Supplementary file 21 — Expression patterns of cell type markers in spatial transcriptomics data for non-compacted soil grown roots. A PDF summary file that includes the sample and gene information for visualization is included. The raw spatial transcriptomics data for non-compacted soil grown roots is also included. [file 41586_2025_8941_MOESM21_ESM.zip › Supplementary Data 6_Marker_expression_in_non-compacted-soils-based_Spatial_transcriptomics_Rice/Atrichoblast-LOC_Os03g19990.png]

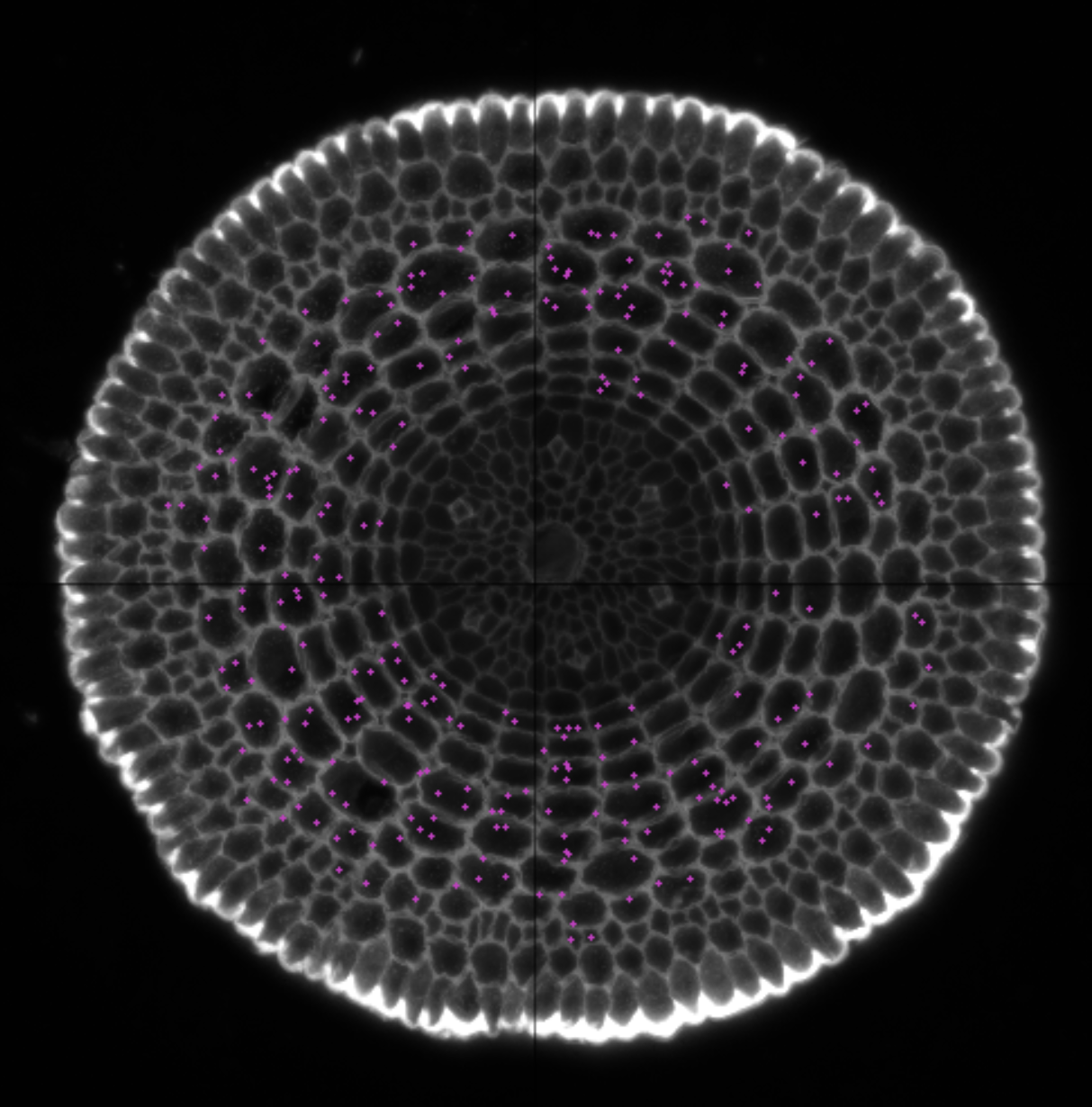

Supplement: Supplementary file 21 — Expression patterns of cell type markers in spatial transcriptomics data for non-compacted soil grown roots. A PDF summary file that includes the sample and gene information for visualization is included. The raw spatial transcriptomics data for non-compacted soil grown roots is also included. [file 41586_2025_8941_MOESM21_ESM.zip › Supplementary Data 6_Marker_expression_in_non-compacted-soils-based_Spatial_transcriptomics_Rice/Cortex-LOC_Os01g19220.png]

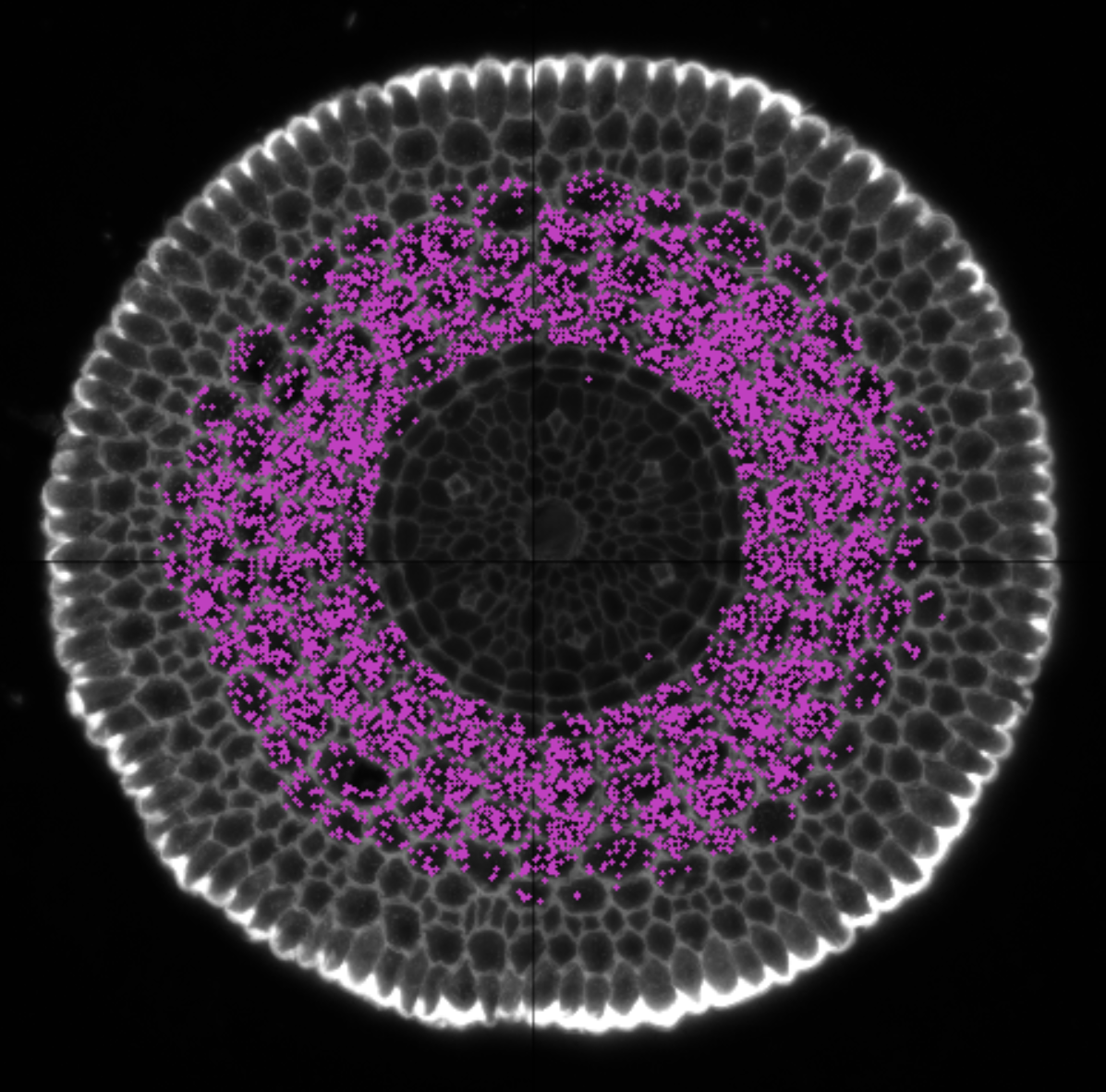

Supplement: Supplementary file 21 — Expression patterns of cell type markers in spatial transcriptomics data for non-compacted soil grown roots. A PDF summary file that includes the sample and gene information for visualization is included. The raw spatial transcriptomics data for non-compacted soil grown roots is also included. [file 41586_2025_8941_MOESM21_ESM.zip › Supplementary Data 6_Marker_expression_in_non-compacted-soils-based_Spatial_transcriptomics_Rice/Cortex-LOC_Os03g04310.png]

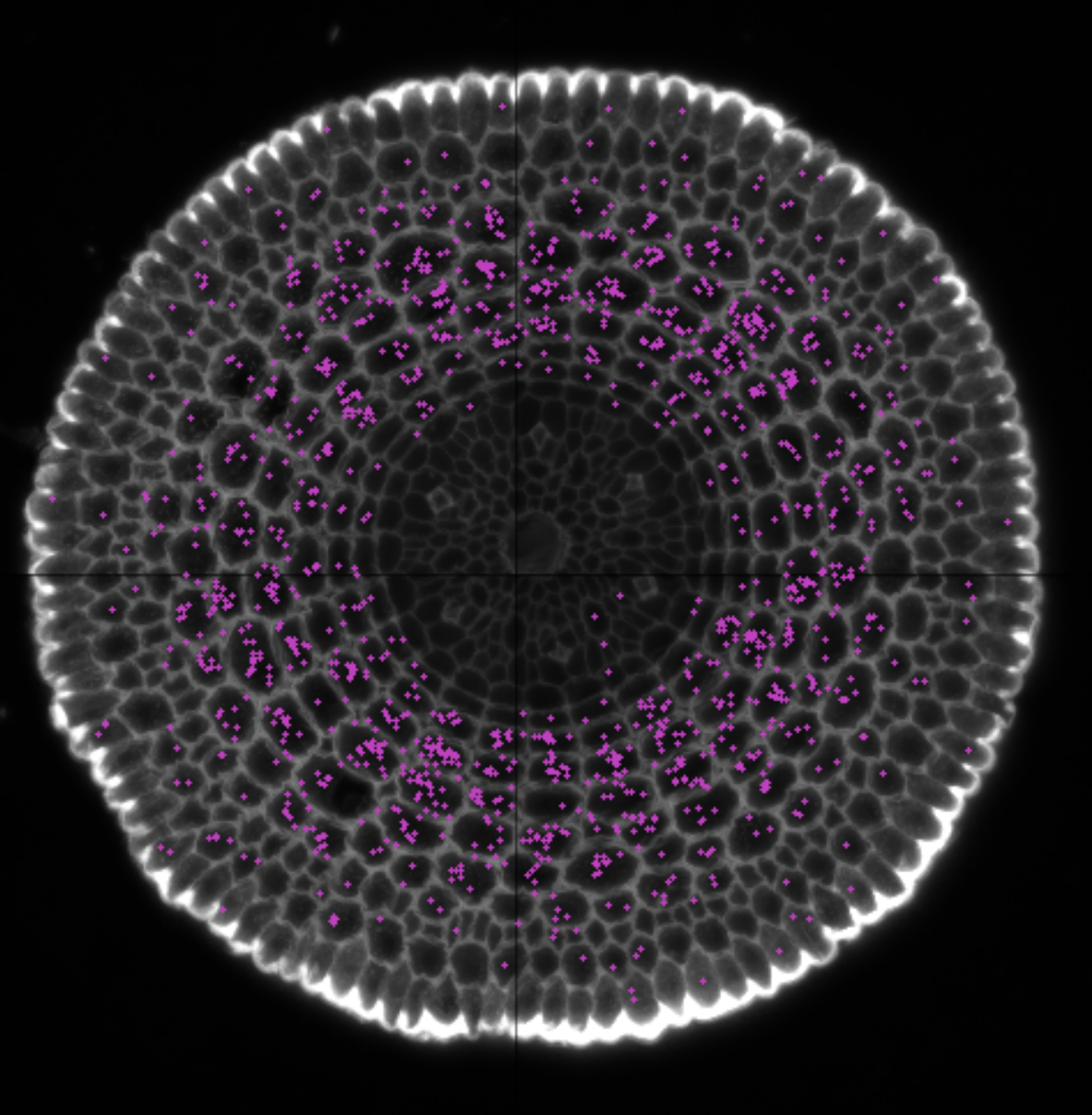

Supplement: Supplementary file 21 — Expression patterns of cell type markers in spatial transcriptomics data for non-compacted soil grown roots. A PDF summary file that includes the sample and gene information for visualization is included. The raw spatial transcriptomics data for non-compacted soil grown roots is also included. [file 41586_2025_8941_MOESM21_ESM.zip › Supplementary Data 6_Marker_expression_in_non-compacted-soils-based_Spatial_transcriptomics_Rice/Cortex-LOC_Os04g49560.png]

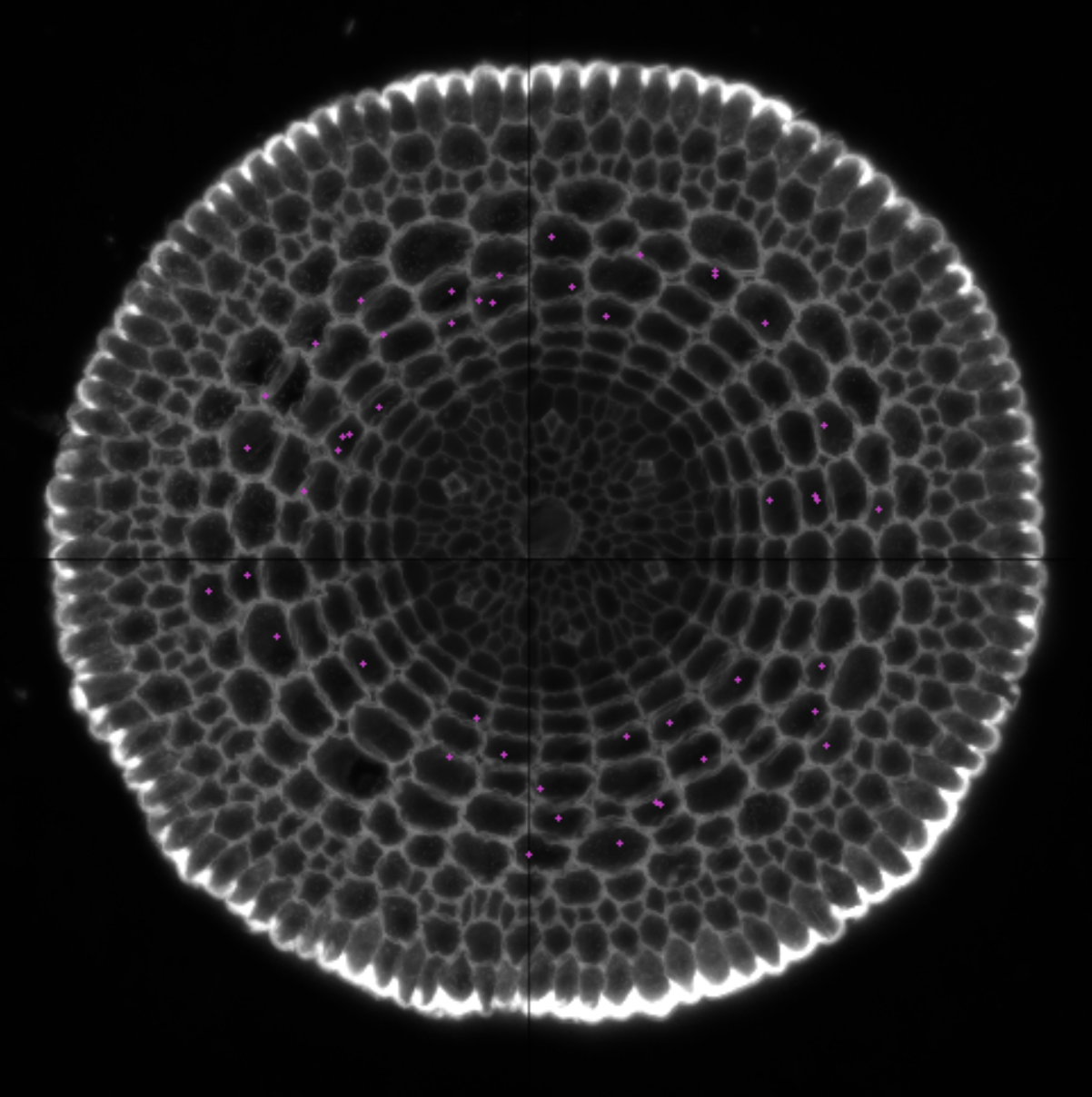

Supplement: Supplementary file 21 — Expression patterns of cell type markers in spatial transcriptomics data for non-compacted soil grown roots. A PDF summary file that includes the sample and gene information for visualization is included. The raw spatial transcriptomics data for non-compacted soil grown roots is also included. [file 41586_2025_8941_MOESM21_ESM.zip › Supplementary Data 6_Marker_expression_in_non-compacted-soils-based_Spatial_transcriptomics_Rice/Cortex-LOC_Os05g33080.png]

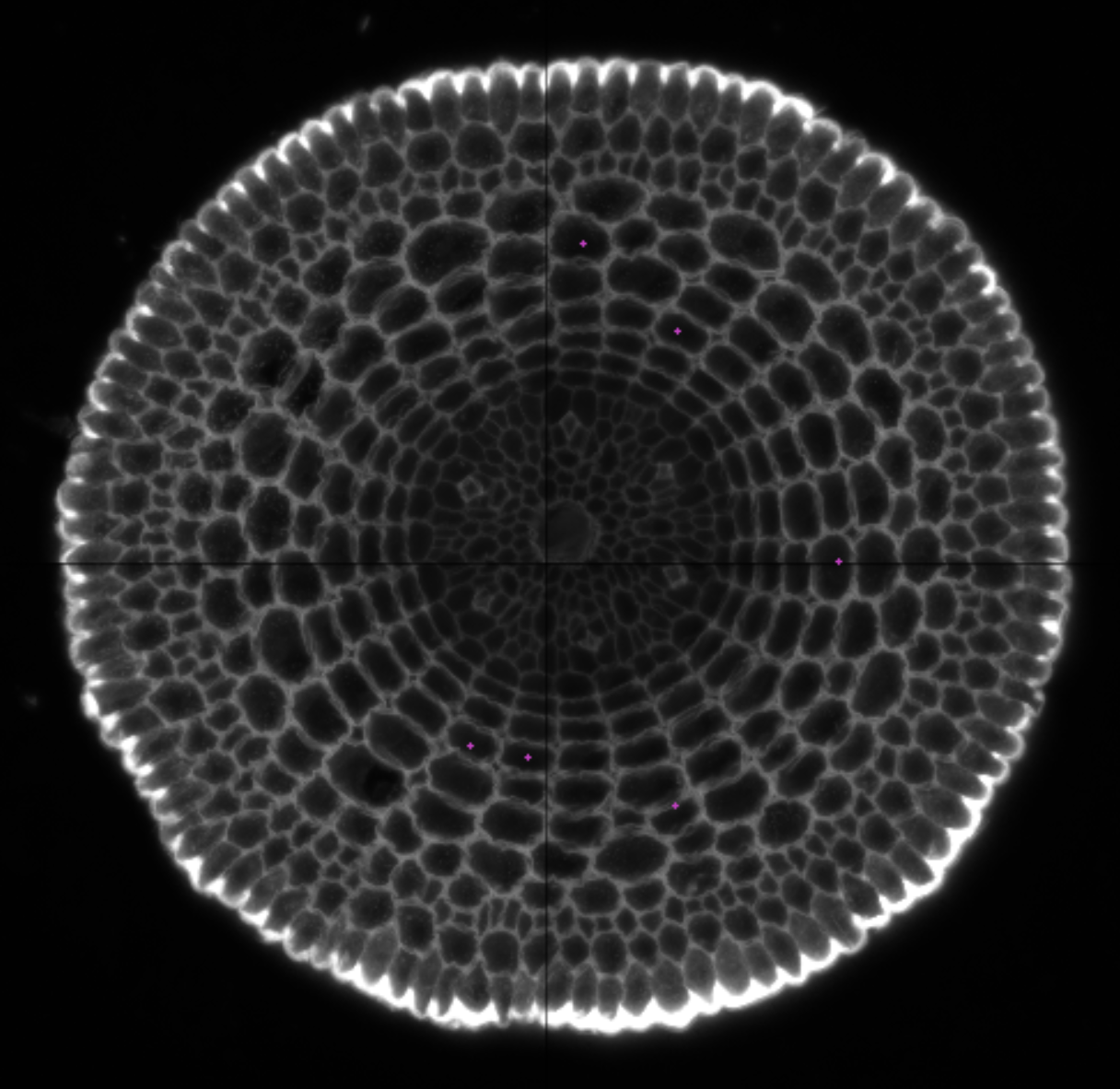

Supplement: Supplementary file 21 — Expression patterns of cell type markers in spatial transcriptomics data for non-compacted soil grown roots. A PDF summary file that includes the sample and gene information for visualization is included. The raw spatial transcriptomics data for non-compacted soil grown roots is also included. [file 41586_2025_8941_MOESM21_ESM.zip › Supplementary Data 6_Marker_expression_in_non-compacted-soils-based_Spatial_transcriptomics_Rice/Cortex-LOC_Os06g30730.png]

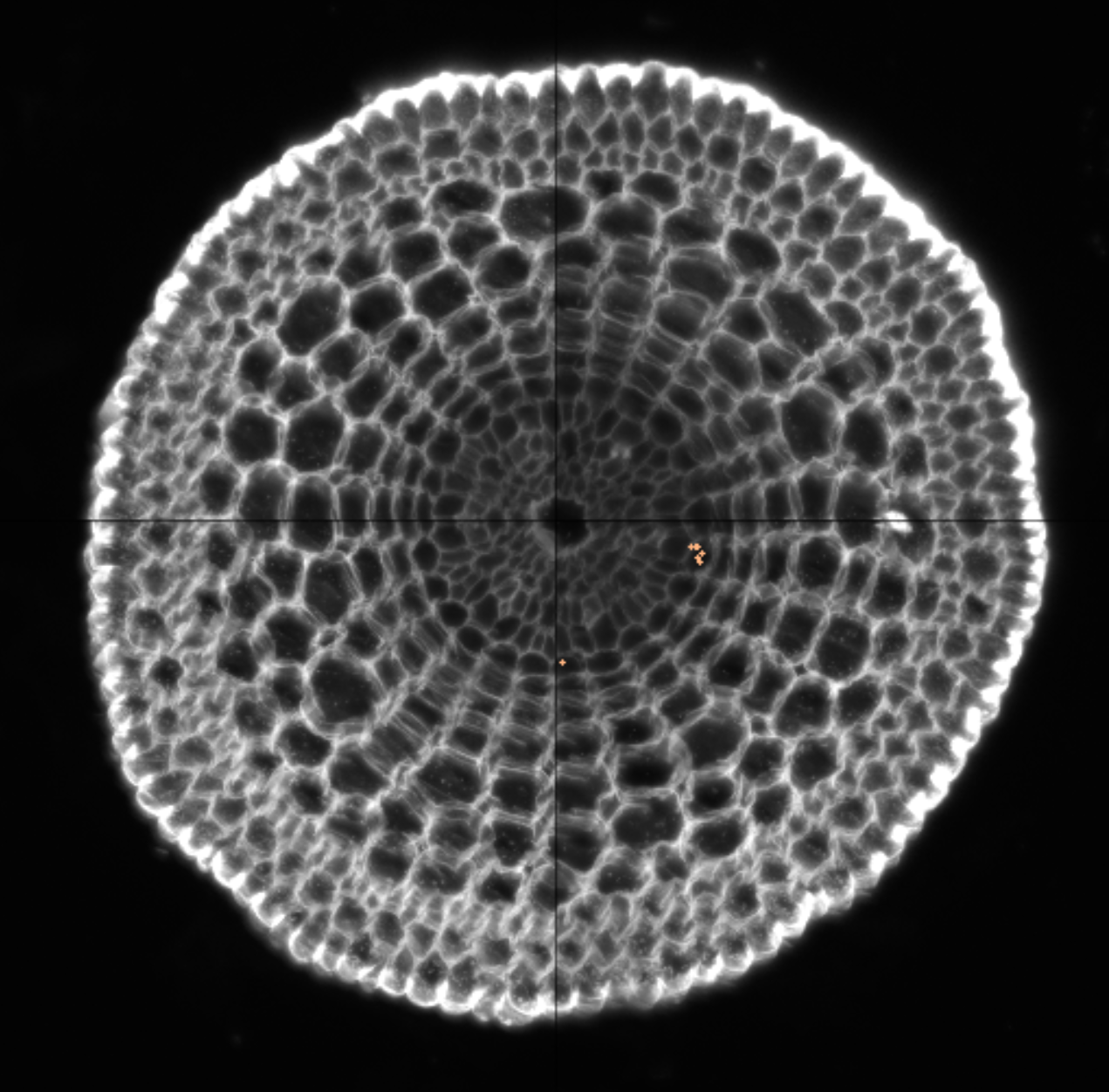

Supplement: Supplementary file 21 — Expression patterns of cell type markers in spatial transcriptomics data for non-compacted soil grown roots. A PDF summary file that includes the sample and gene information for visualization is included. The raw spatial transcriptomics data for non-compacted soil grown roots is also included. [file 41586_2025_8941_MOESM21_ESM.zip › Supplementary Data 6_Marker_expression_in_non-compacted-soils-based_Spatial_transcriptomics_Rice/Endodermis-LOC_Os01g15810.png]

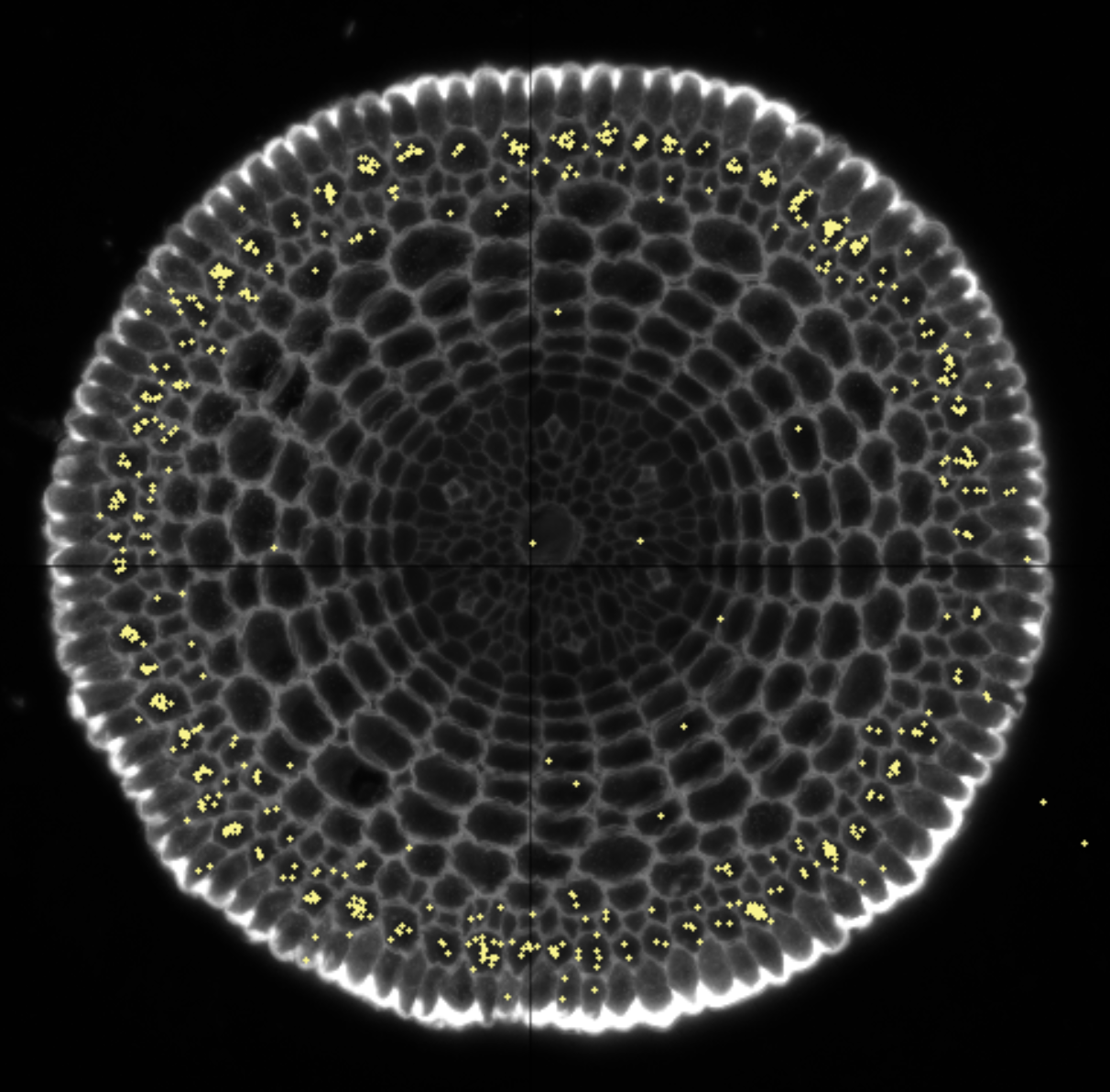

Supplement: Supplementary file 21 — Expression patterns of cell type markers in spatial transcriptomics data for non-compacted soil grown roots. A PDF summary file that includes the sample and gene information for visualization is included. The raw spatial transcriptomics data for non-compacted soil grown roots is also included. [file 41586_2025_8941_MOESM21_ESM.zip › Supplementary Data 6_Marker_expression_in_non-compacted-soils-based_Spatial_transcriptomics_Rice/Exodermis-LOC_Os03g02460.png]

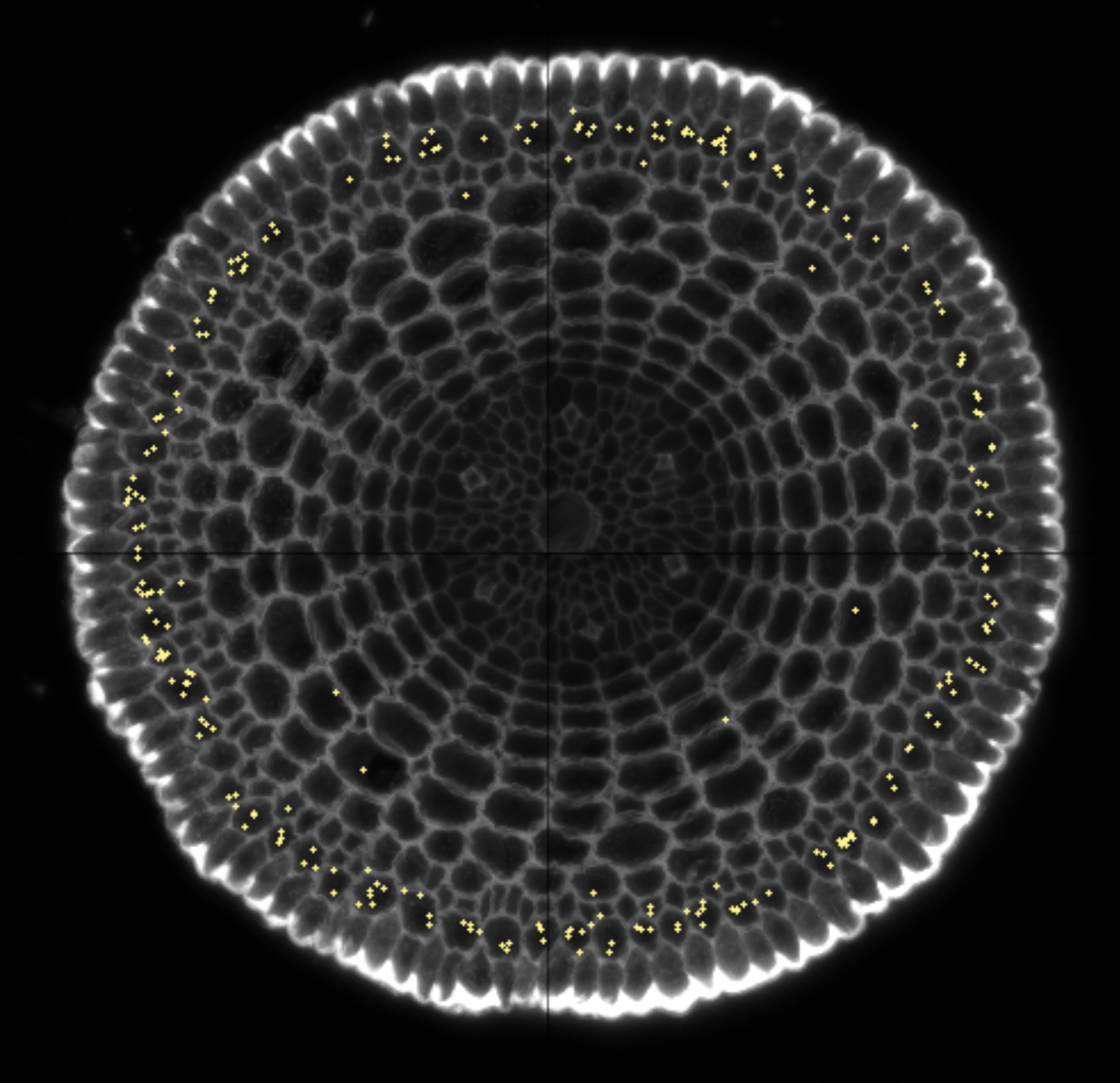

Supplement: Supplementary file 21 — Expression patterns of cell type markers in spatial transcriptomics data for non-compacted soil grown roots. A PDF summary file that includes the sample and gene information for visualization is included. The raw spatial transcriptomics data for non-compacted soil grown roots is also included. [file 41586_2025_8941_MOESM21_ESM.zip › Supplementary Data 6_Marker_expression_in_non-compacted-soils-based_Spatial_transcriptomics_Rice/Exodermis-LOC_Os03g37411.png]

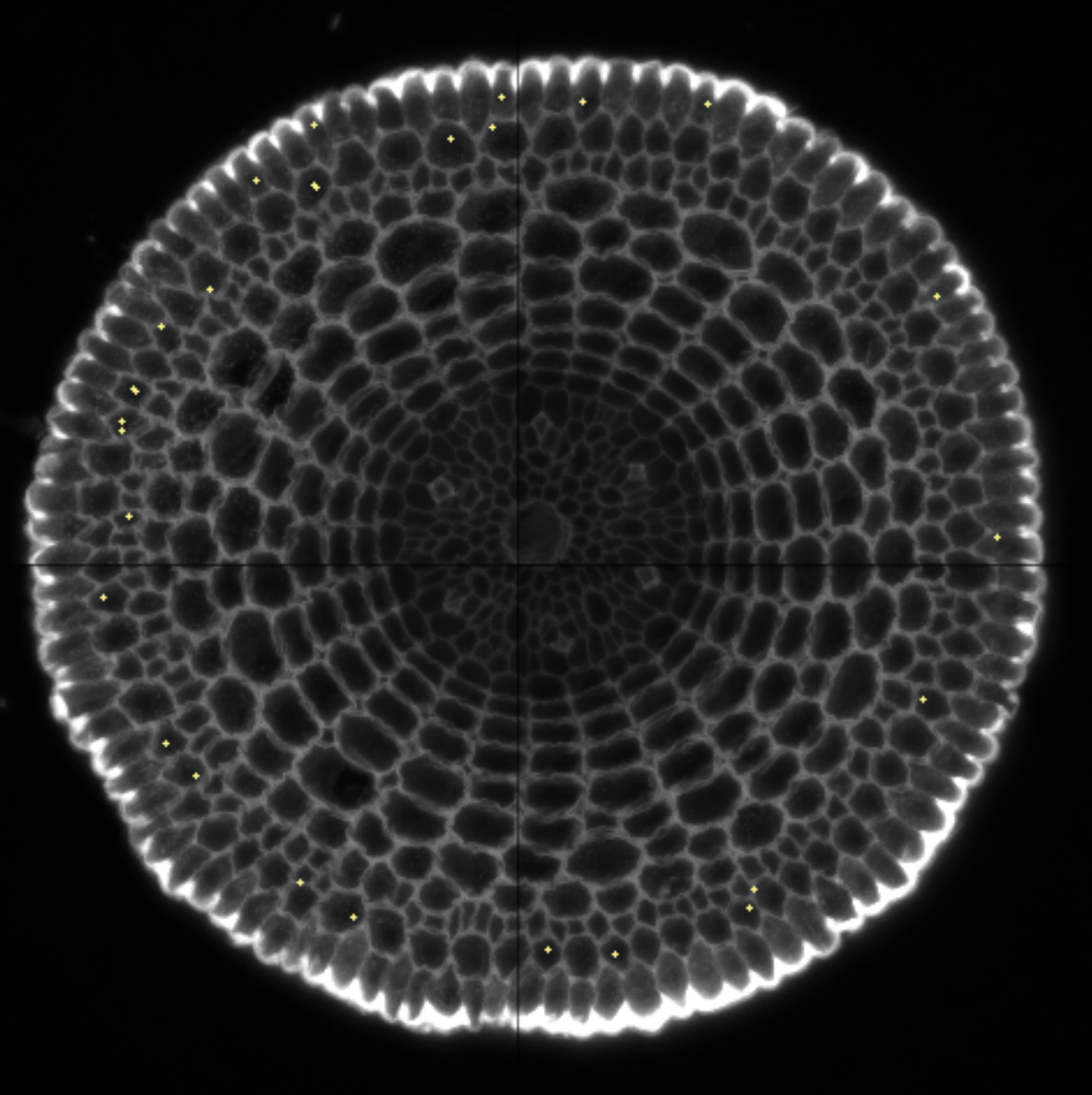

Supplement: Supplementary file 21 — Expression patterns of cell type markers in spatial transcriptomics data for non-compacted soil grown roots. A PDF summary file that includes the sample and gene information for visualization is included. The raw spatial transcriptomics data for non-compacted soil grown roots is also included. [file 41586_2025_8941_MOESM21_ESM.zip › Supplementary Data 6_Marker_expression_in_non-compacted-soils-based_Spatial_transcriptomics_Rice/Exodermis-LOC_Os04g37980.png]

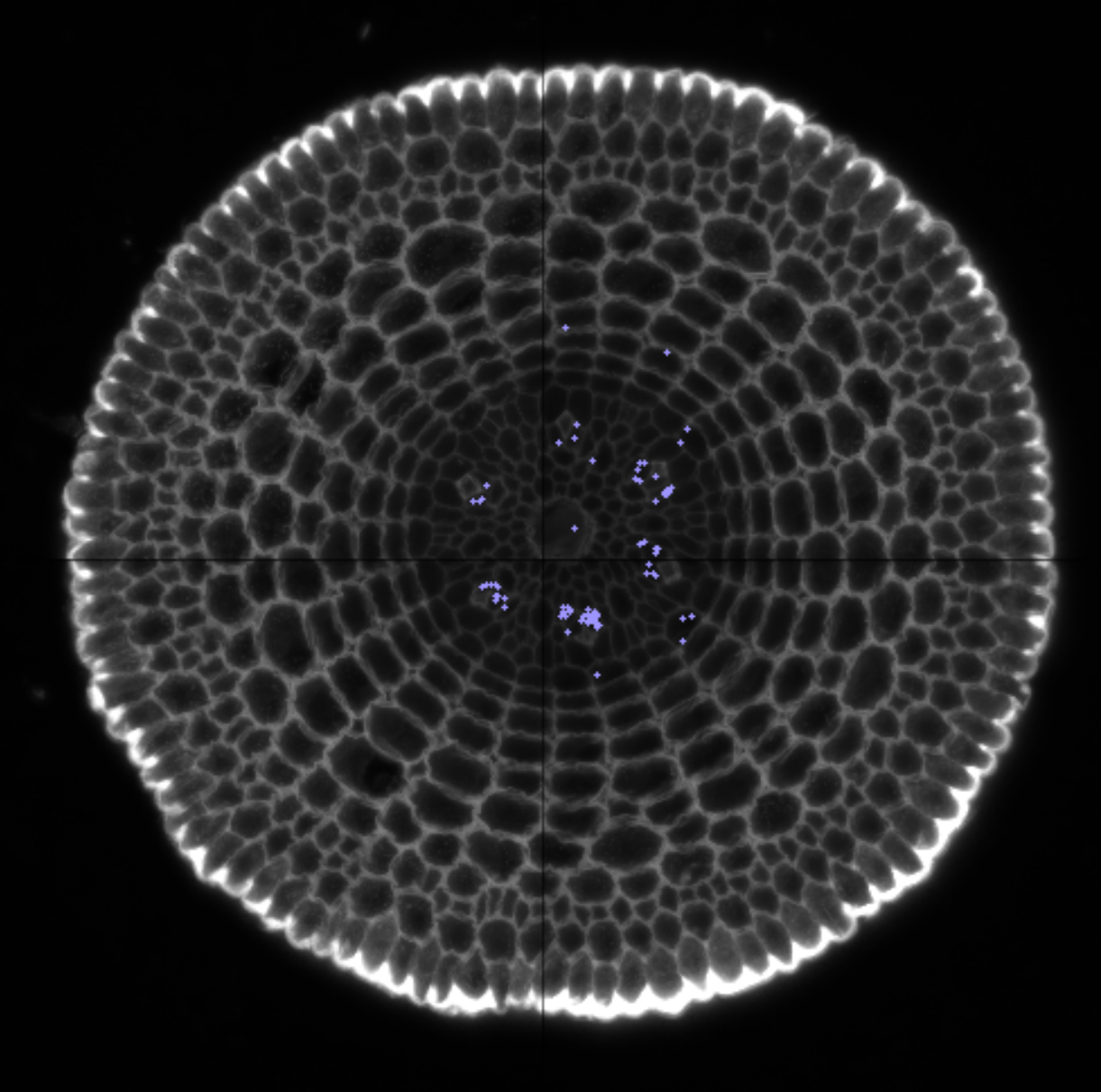

Supplement: Supplementary file 21 — Expression patterns of cell type markers in spatial transcriptomics data for non-compacted soil grown roots. A PDF summary file that includes the sample and gene information for visualization is included. The raw spatial transcriptomics data for non-compacted soil grown roots is also included. [file 41586_2025_8941_MOESM21_ESM.zip › Supplementary Data 6_Marker_expression_in_non-compacted-soils-based_Spatial_transcriptomics_Rice/Phloem-LOC_Os06g45410.png]

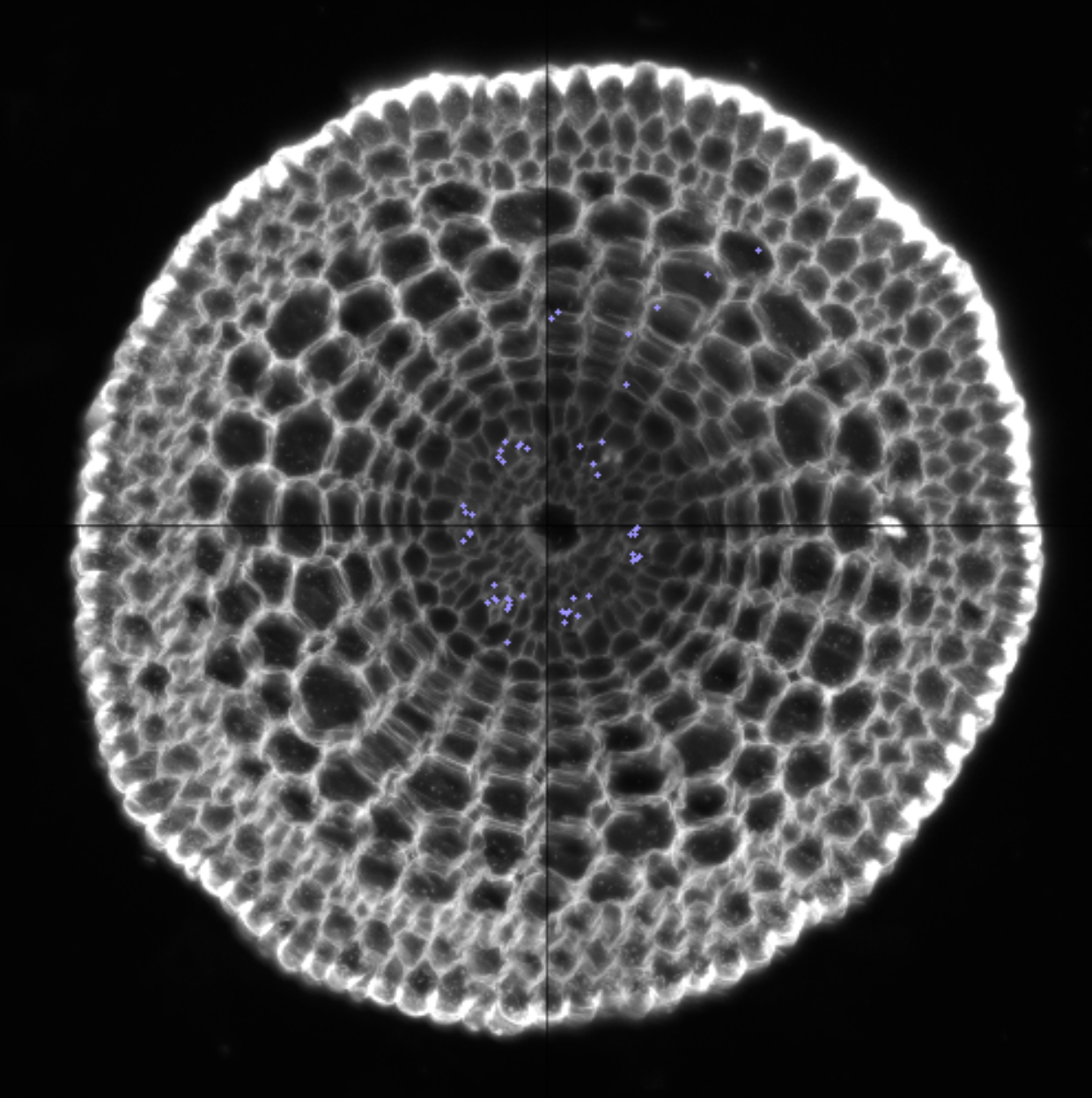

Supplement: Supplementary file 21 — Expression patterns of cell type markers in spatial transcriptomics data for non-compacted soil grown roots. A PDF summary file that includes the sample and gene information for visualization is included. The raw spatial transcriptomics data for non-compacted soil grown roots is also included. [file 41586_2025_8941_MOESM21_ESM.zip › Supplementary Data 6_Marker_expression_in_non-compacted-soils-based_Spatial_transcriptomics_Rice/Phloem-LOC_Os06g45410-2.png]

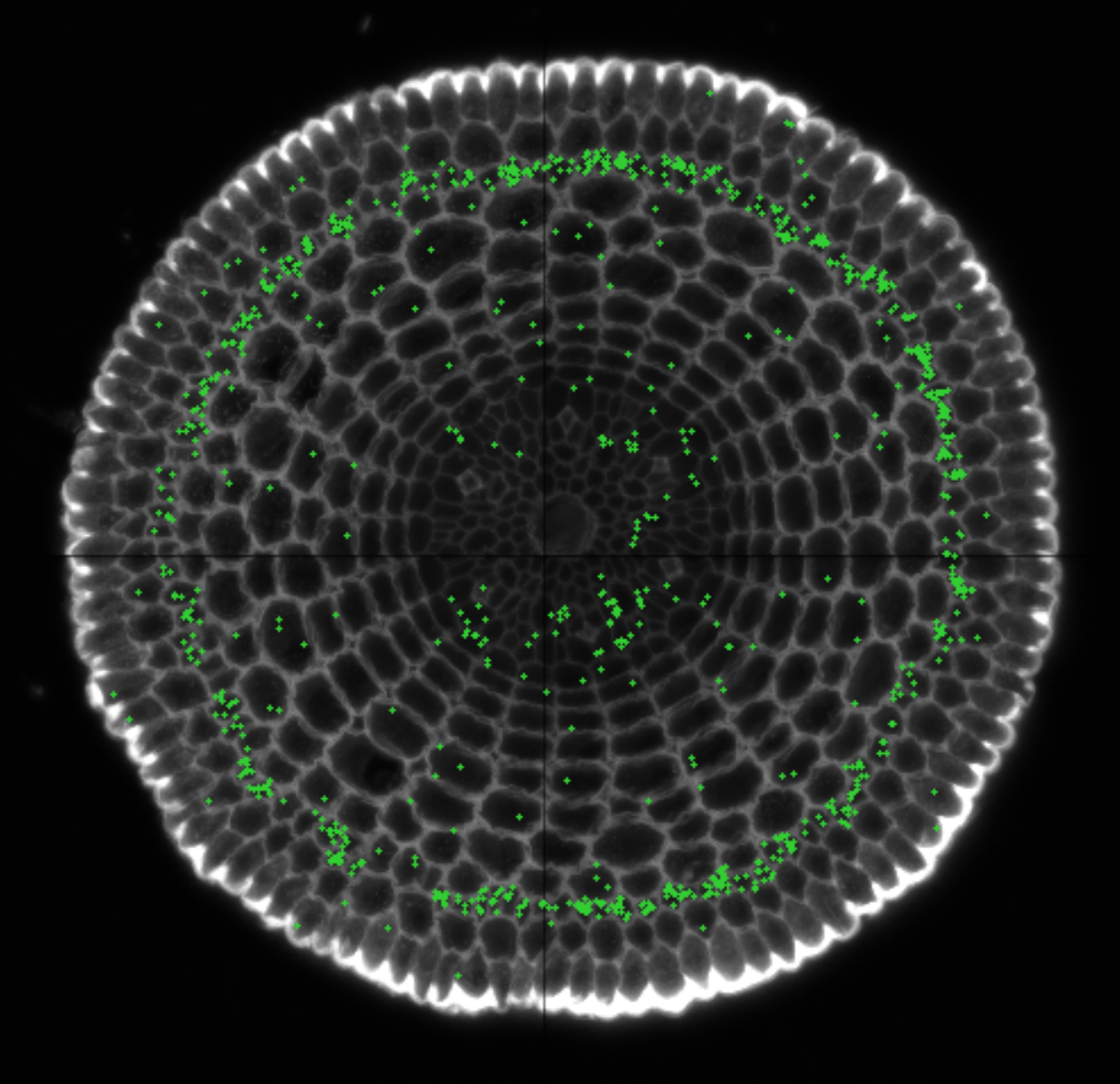

Supplement: Supplementary file 21 — Expression patterns of cell type markers in spatial transcriptomics data for non-compacted soil grown roots. A PDF summary file that includes the sample and gene information for visualization is included. The raw spatial transcriptomics data for non-compacted soil grown roots is also included. [file 41586_2025_8941_MOESM21_ESM.zip › Supplementary Data 6_Marker_expression_in_non-compacted-soils-based_Spatial_transcriptomics_Rice/Sclerenchyma-LOC_Os08g02300.png]

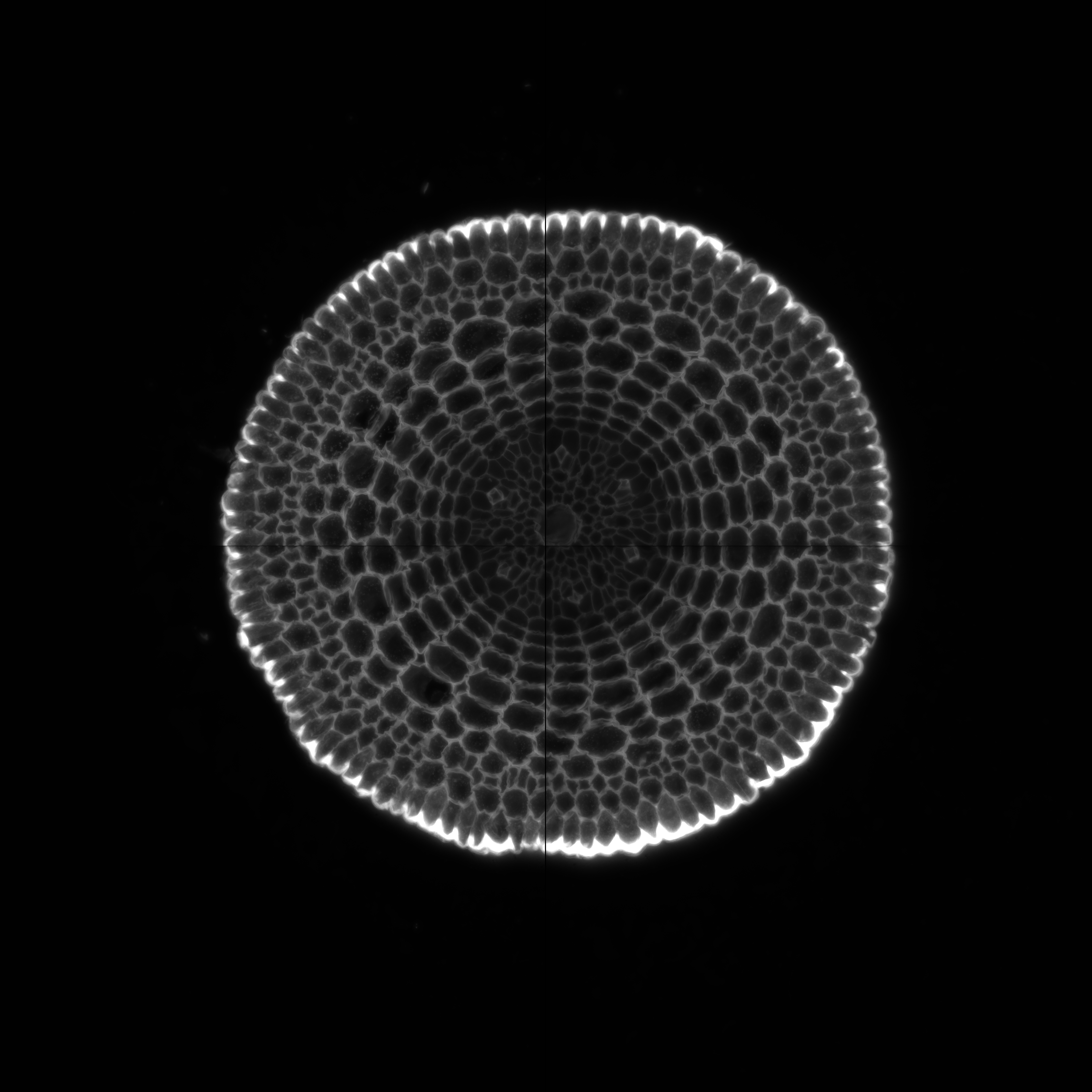

Supplement: Supplementary file 21 — Expression patterns of cell type markers in spatial transcriptomics data for non-compacted soil grown roots. A PDF summary file that includes the sample and gene information for visualization is included. The raw spatial transcriptomics data for non-compacted soil grown roots is also included. [file 41586_2025_8941_MOESM21_ESM.zip › Supplementary Data 6_Marker_expression_in_non-compacted-soils-based_Spatial_transcriptomics_Rice/Soil-NC-spatial-raw data/33474-Benfey-S2_B1-2_Calco.tiff]

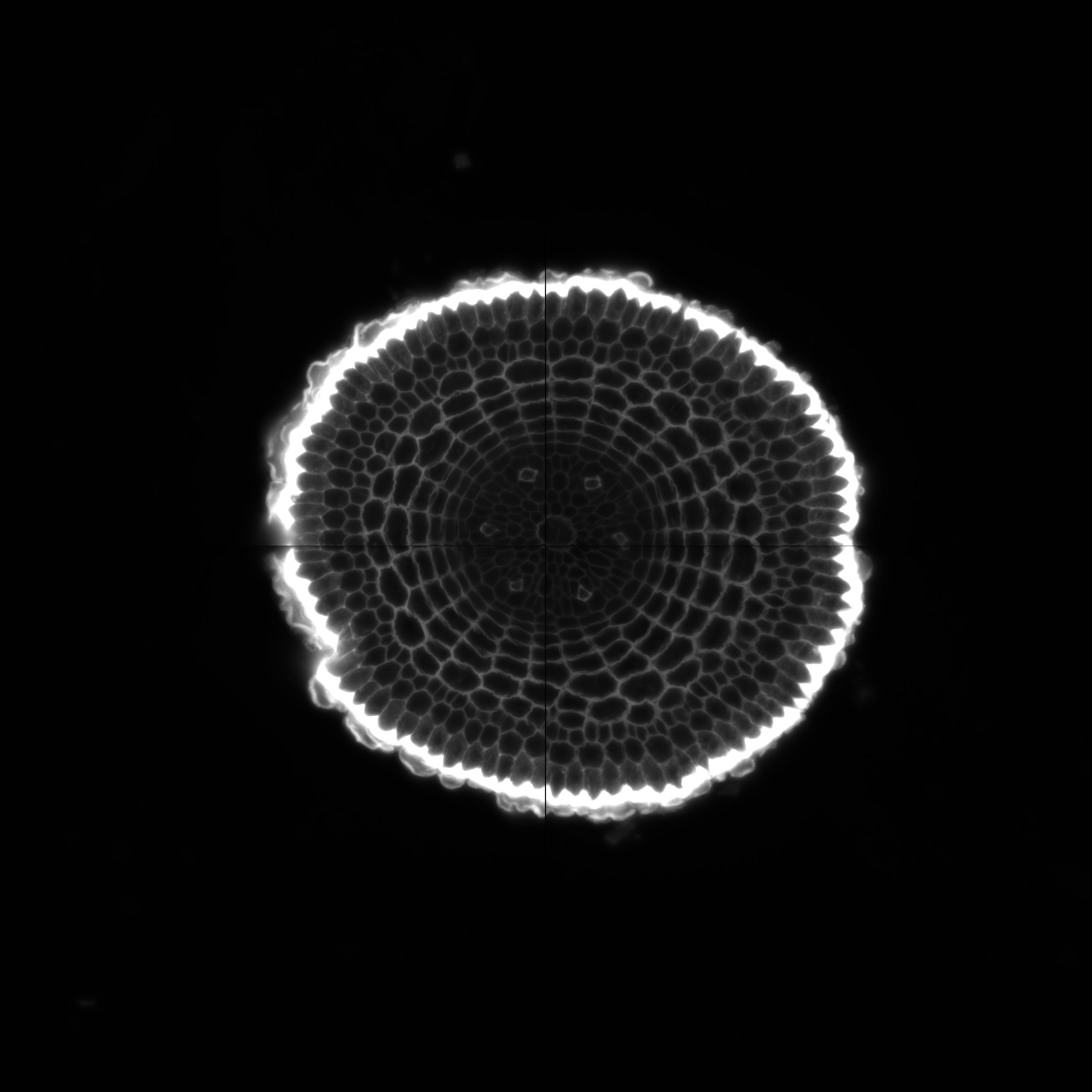

Supplement: Supplementary file 21 — Expression patterns of cell type markers in spatial transcriptomics data for non-compacted soil grown roots. A PDF summary file that includes the sample and gene information for visualization is included. The raw spatial transcriptomics data for non-compacted soil grown roots is also included. [file 41586_2025_8941_MOESM21_ESM.zip › Supplementary Data 6_Marker_expression_in_non-compacted-soils-based_Spatial_transcriptomics_Rice/Soil-NC-spatial-raw data/33474-Benfey-S2_B2-1_Calco.tiff]

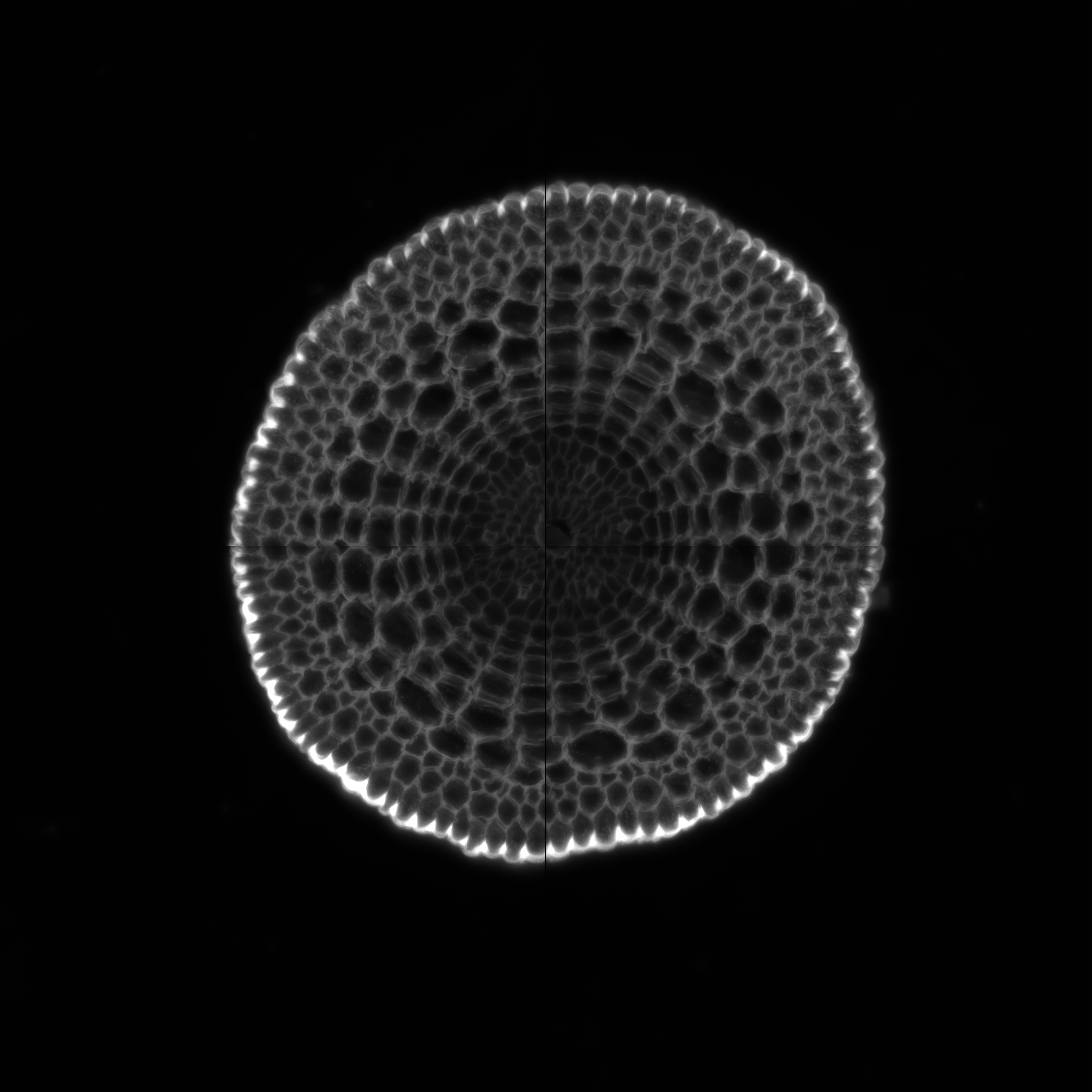

Supplement: Supplementary file 21 — Expression patterns of cell type markers in spatial transcriptomics data for non-compacted soil grown roots. A PDF summary file that includes the sample and gene information for visualization is included. The raw spatial transcriptomics data for non-compacted soil grown roots is also included. [file 41586_2025_8941_MOESM21_ESM.zip › Supplementary Data 6_Marker_expression_in_non-compacted-soils-based_Spatial_transcriptomics_Rice/Soil-NC-spatial-raw data/33474-Benfey-S2_D2-1_Calco.tiff]

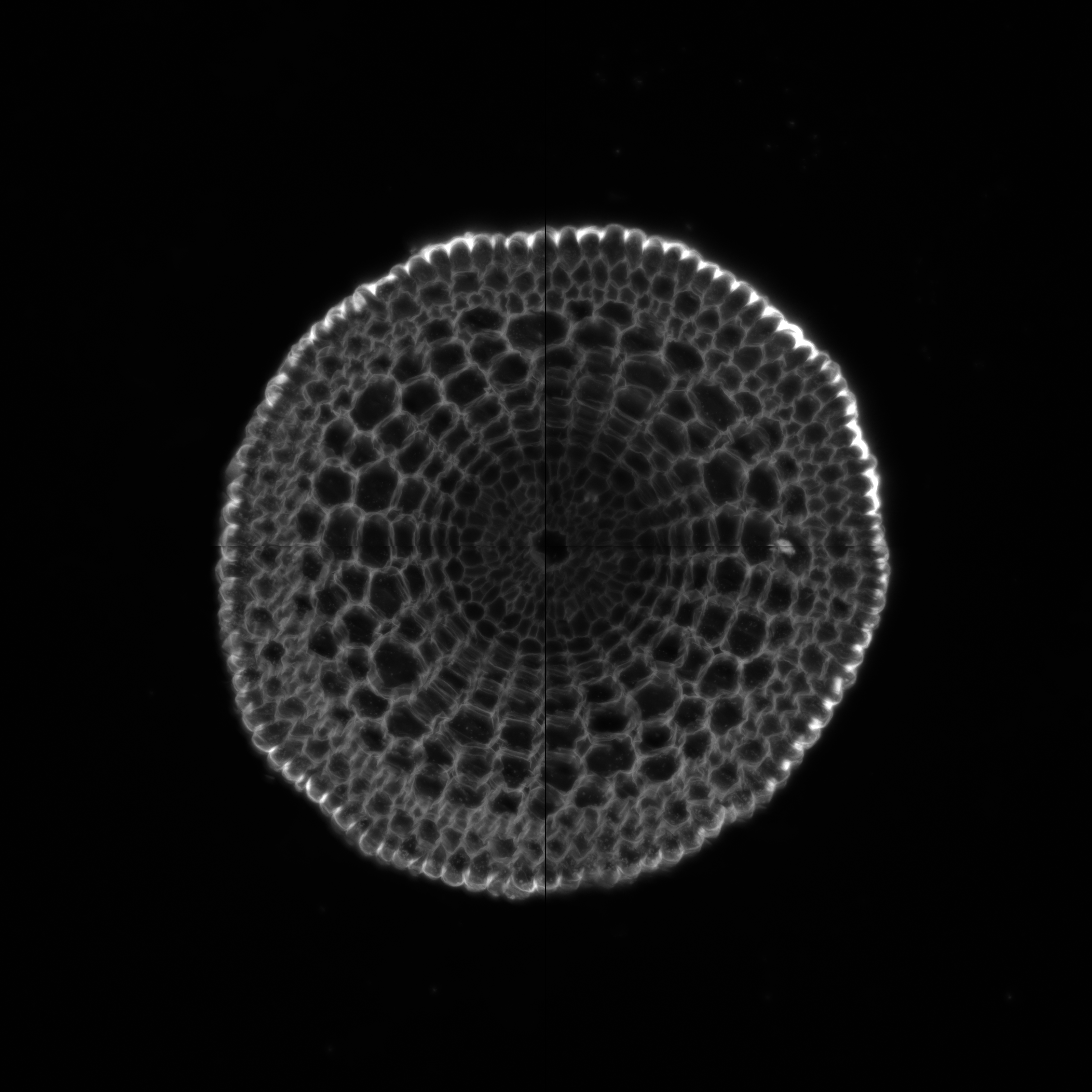

Supplement: Supplementary file 21 — Expression patterns of cell type markers in spatial transcriptomics data for non-compacted soil grown roots. A PDF summary file that includes the sample and gene information for visualization is included. The raw spatial transcriptomics data for non-compacted soil grown roots is also included. [file 41586_2025_8941_MOESM21_ESM.zip › Supplementary Data 6_Marker_expression_in_non-compacted-soils-based_Spatial_transcriptomics_Rice/Soil-NC-spatial-raw data/33474-Benfey-S2_D2-2_Calco.tiff]

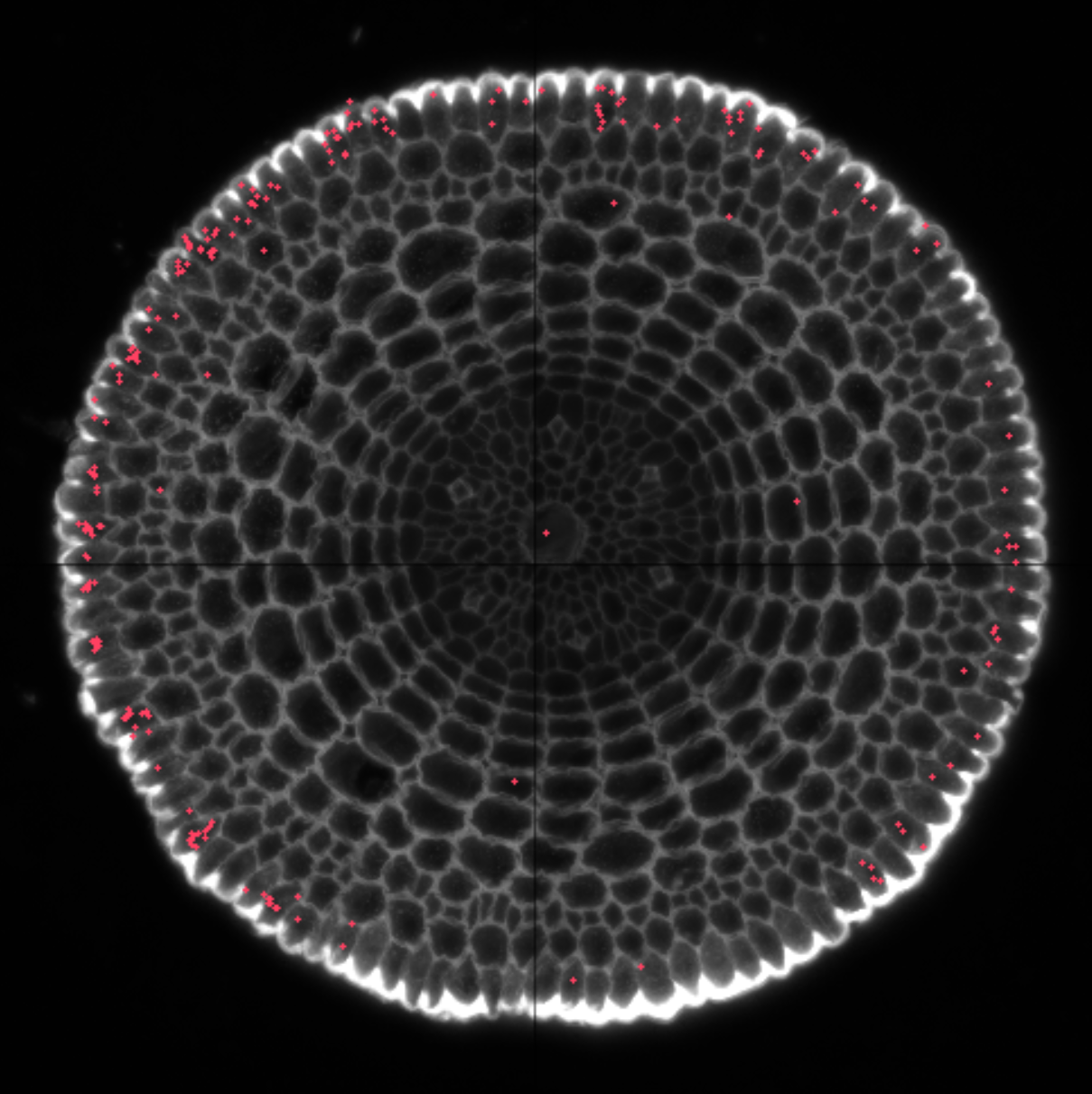

Supplement: Supplementary file 21 — Expression patterns of cell type markers in spatial transcriptomics data for non-compacted soil grown roots. A PDF summary file that includes the sample and gene information for visualization is included. The raw spatial transcriptomics data for non-compacted soil grown roots is also included. [file 41586_2025_8941_MOESM21_ESM.zip › Supplementary Data 6_Marker_expression_in_non-compacted-soils-based_Spatial_transcriptomics_Rice/Trichoblast-LOC_Os10g42750.png]

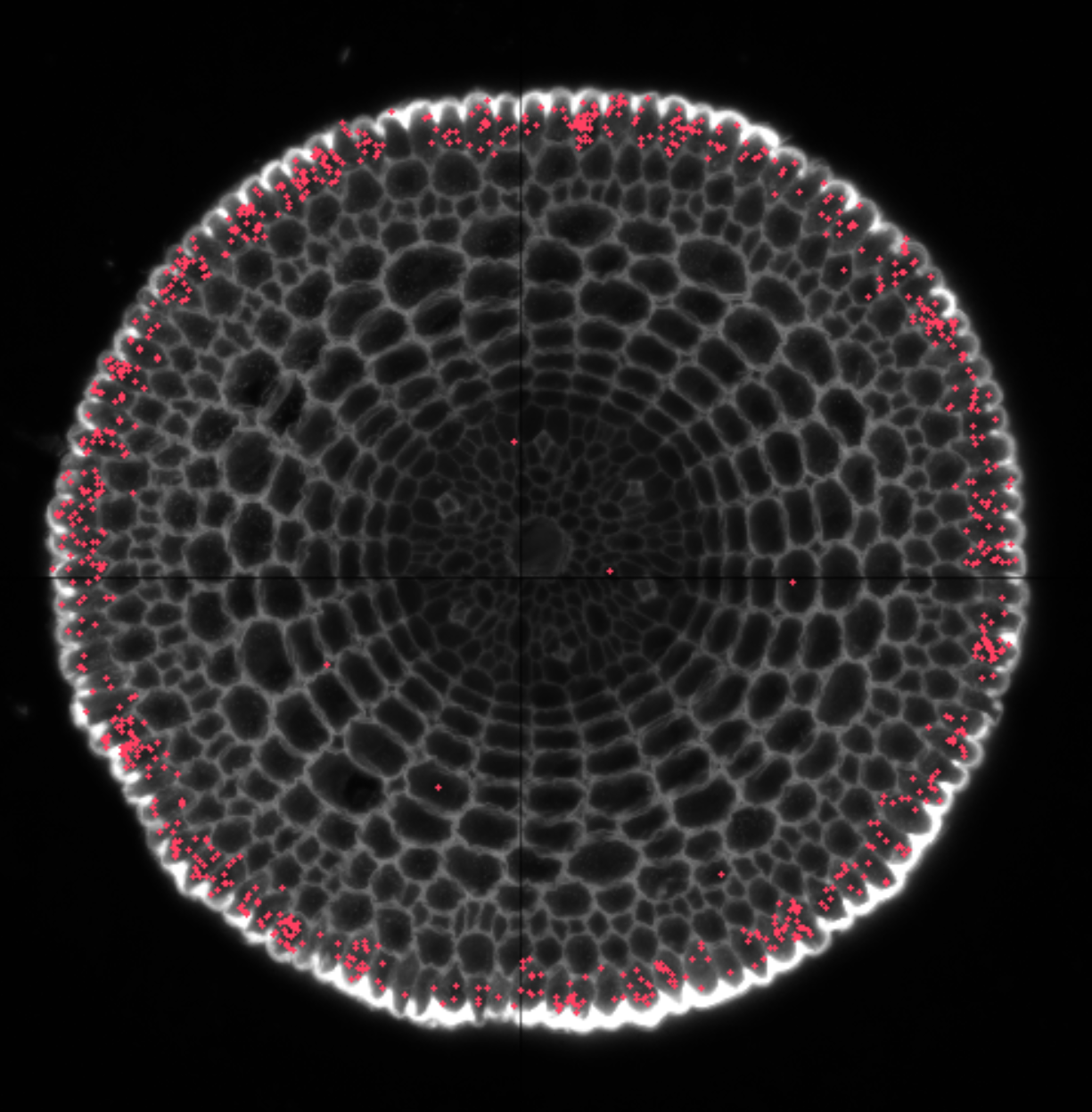

Supplement: Supplementary file 21 — Expression patterns of cell type markers in spatial transcriptomics data for non-compacted soil grown roots. A PDF summary file that includes the sample and gene information for visualization is included. The raw spatial transcriptomics data for non-compacted soil grown roots is also included. [file 41586_2025_8941_MOESM21_ESM.zip › Supplementary Data 6_Marker_expression_in_non-compacted-soils-based_Spatial_transcriptomics_Rice/Trichoblast-LOC_Os12g05380.png]

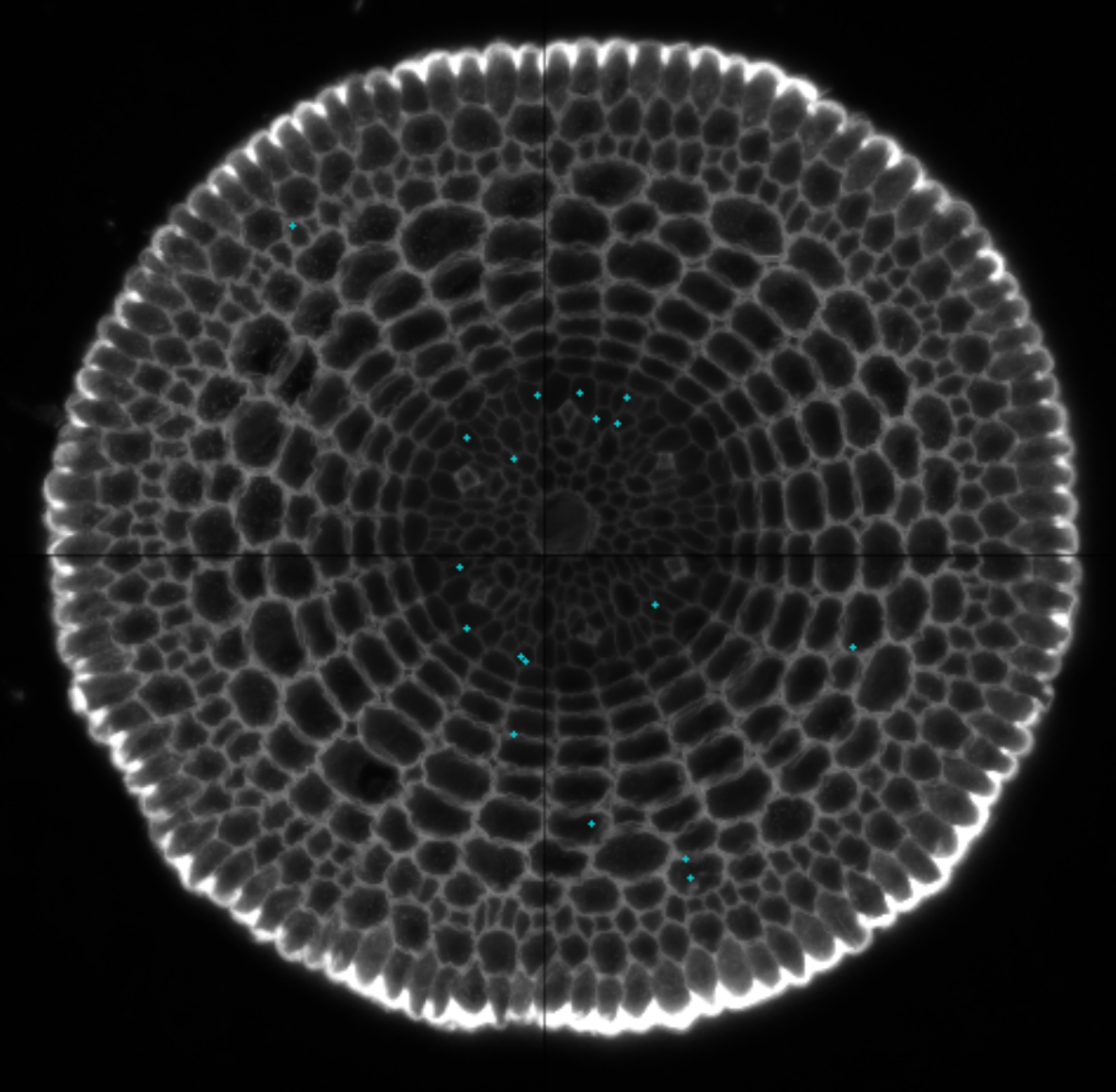

Supplement: Supplementary file 21 — Expression patterns of cell type markers in spatial transcriptomics data for non-compacted soil grown roots. A PDF summary file that includes the sample and gene information for visualization is included. The raw spatial transcriptomics data for non-compacted soil grown roots is also included. [file 41586_2025_8941_MOESM21_ESM.zip › Supplementary Data 6_Marker_expression_in_non-compacted-soils-based_Spatial_transcriptomics_Rice/Vascular tissue-LOC_Os01g19170.png]

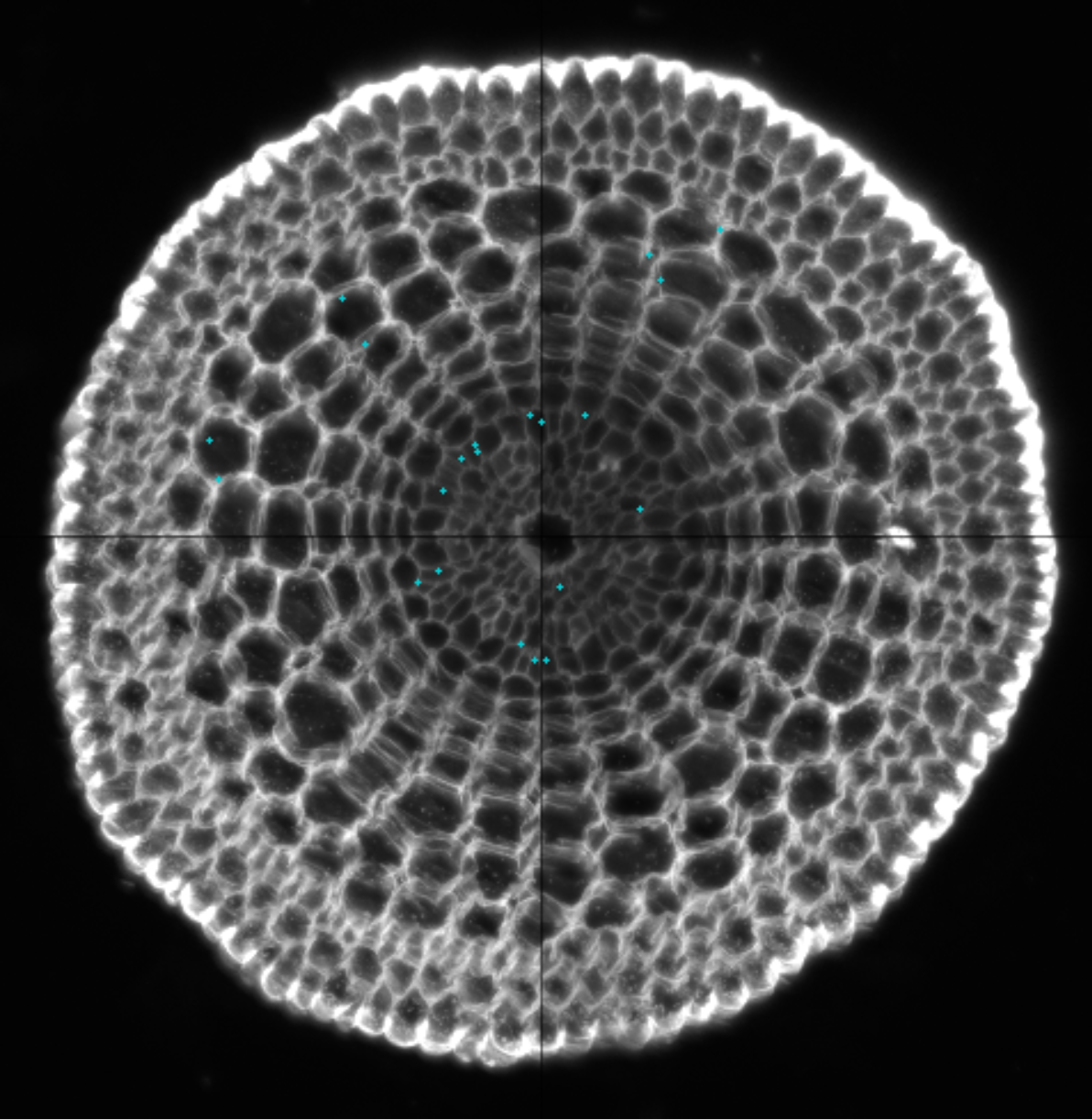

Supplement: Supplementary file 21 — Expression patterns of cell type markers in spatial transcriptomics data for non-compacted soil grown roots. A PDF summary file that includes the sample and gene information for visualization is included. The raw spatial transcriptomics data for non-compacted soil grown roots is also included. [file 41586_2025_8941_MOESM21_ESM.zip › Supplementary Data 6_Marker_expression_in_non-compacted-soils-based_Spatial_transcriptomics_Rice/Vascular tissue-LOC_Os01g19170-2.png]

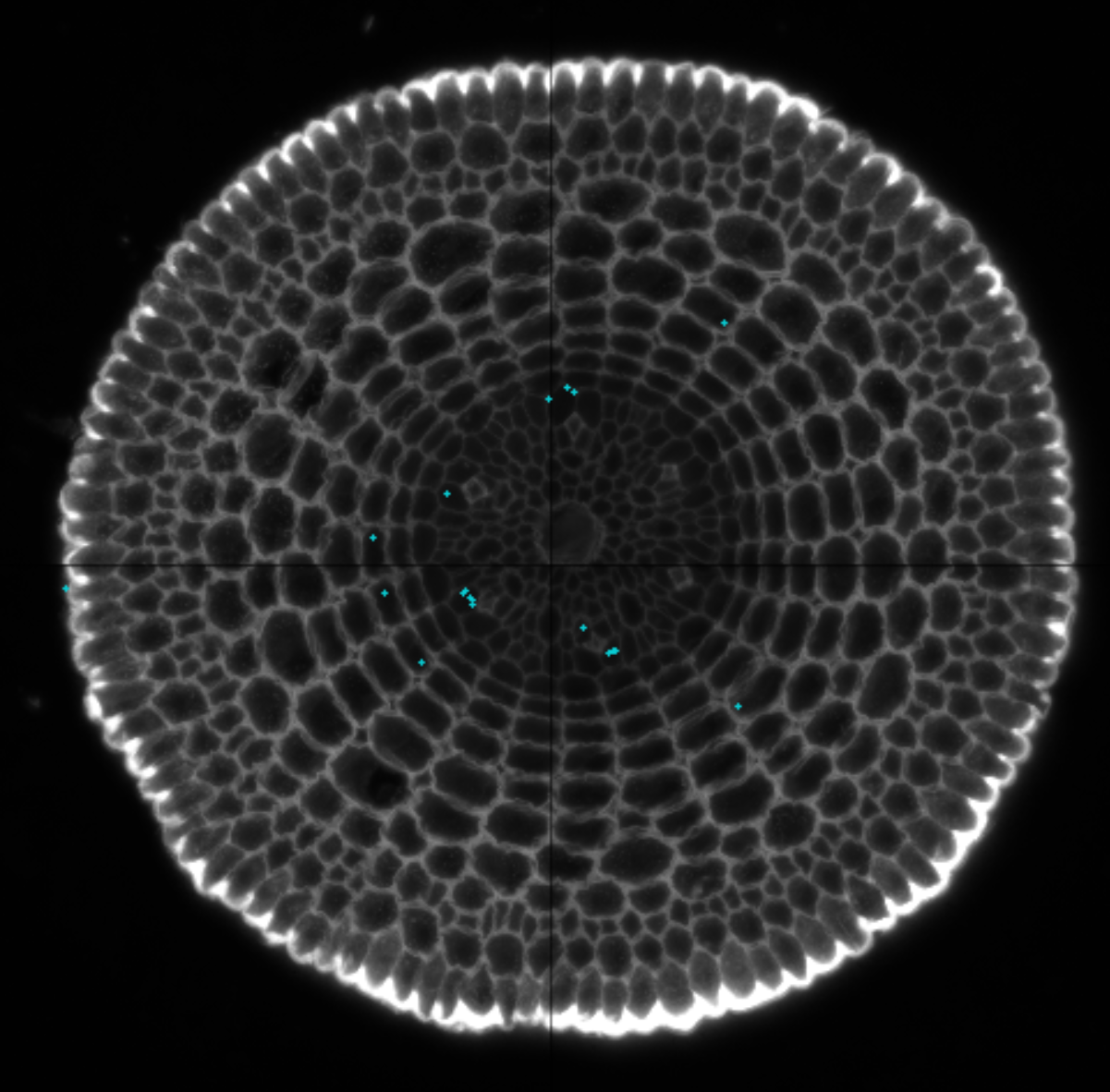

Supplement: Supplementary file 21 — Expression patterns of cell type markers in spatial transcriptomics data for non-compacted soil grown roots. A PDF summary file that includes the sample and gene information for visualization is included. The raw spatial transcriptomics data for non-compacted soil grown roots is also included. [file 41586_2025_8941_MOESM21_ESM.zip › Supplementary Data 6_Marker_expression_in_non-compacted-soils-based_Spatial_transcriptomics_Rice/Vascular tissue-LOC_Os02g56510.png]

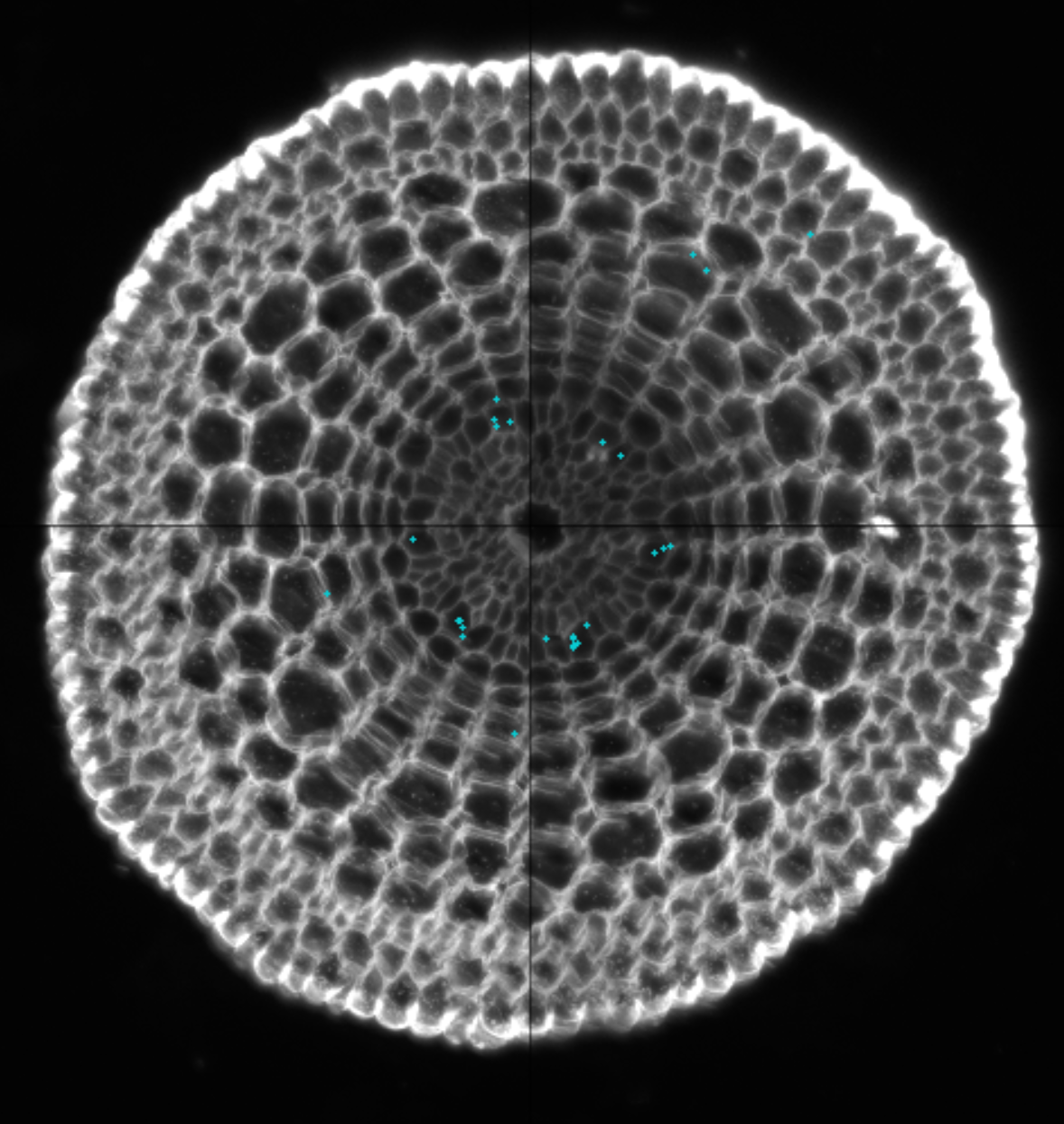

Supplement: Supplementary file 21 — Expression patterns of cell type markers in spatial transcriptomics data for non-compacted soil grown roots. A PDF summary file that includes the sample and gene information for visualization is included. The raw spatial transcriptomics data for non-compacted soil grown roots is also included. [file 41586_2025_8941_MOESM21_ESM.zip › Supplementary Data 6_Marker_expression_in_non-compacted-soils-based_Spatial_transcriptomics_Rice/Vascular tissue-LOC_Os02g56510-2.png]

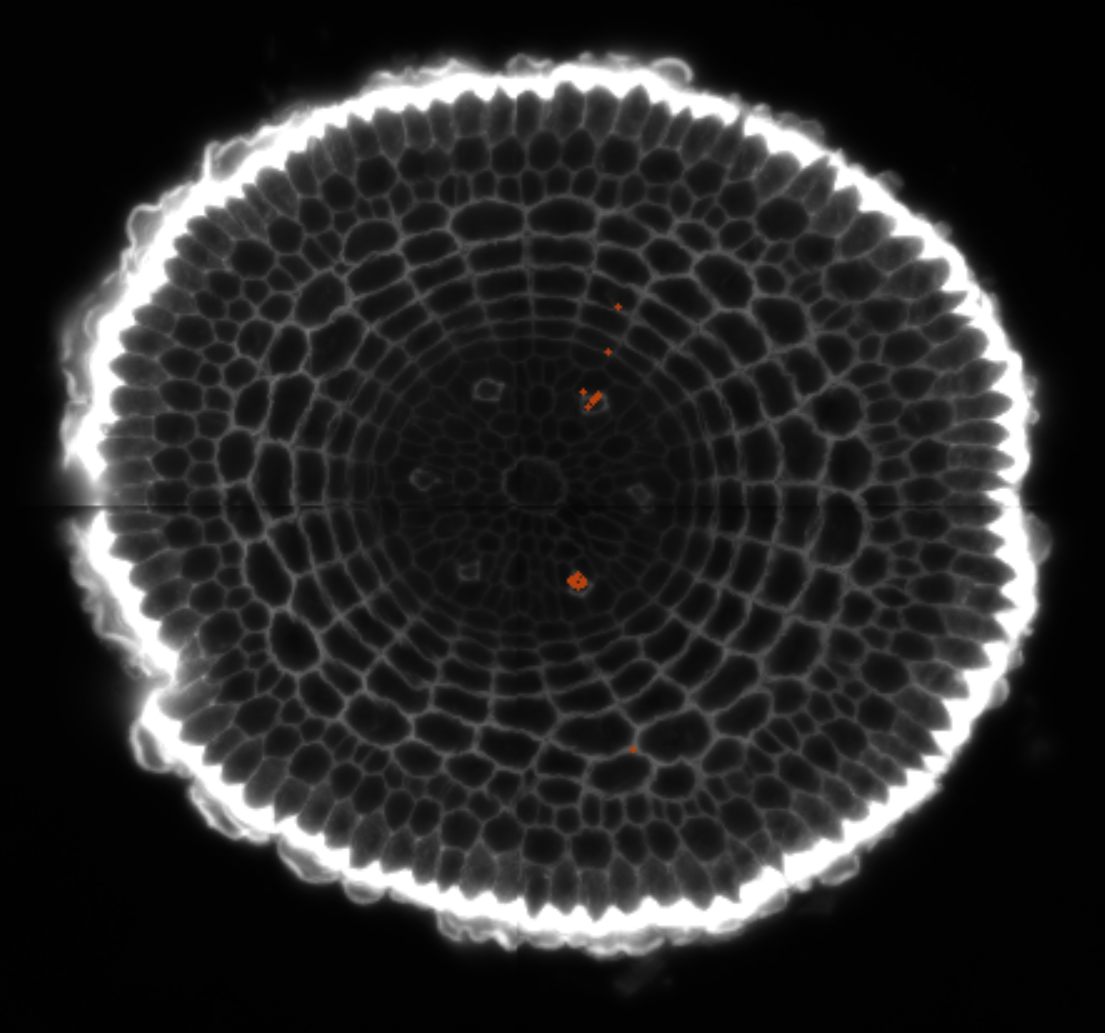

Supplement: Supplementary file 21 — Expression patterns of cell type markers in spatial transcriptomics data for non-compacted soil grown roots. A PDF summary file that includes the sample and gene information for visualization is included. The raw spatial transcriptomics data for non-compacted soil grown roots is also included. [file 41586_2025_8941_MOESM21_ESM.zip › Supplementary Data 6_Marker_expression_in_non-compacted-soils-based_Spatial_transcriptomics_Rice/Xylem-LOC_Os01g48130.png]

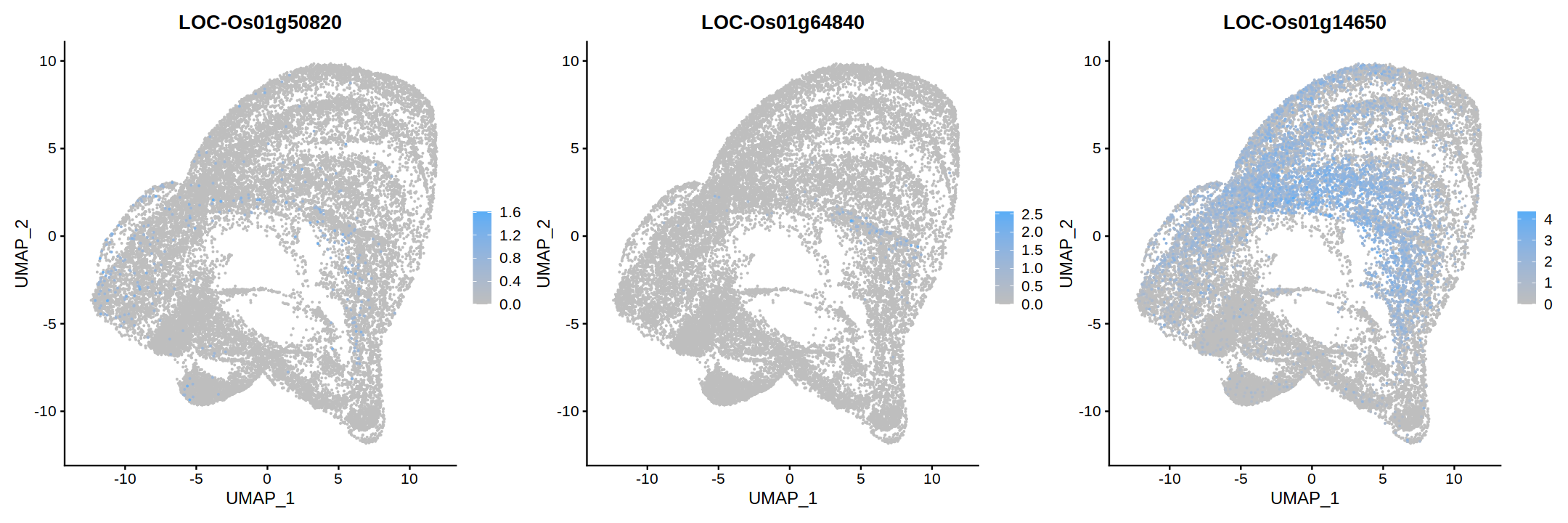

Supplement: Supplementary file 22 — Combined feature plots representing the expression patterns of cell type markers in scRNA-seq data for compacted soil grown roots. Each image represents the gene expressions of markers for one certain cell type. [file 41586_2025_8941_MOESM22_ESM.zip › Supplementary Data 7_Marker_expressions_in_compacted-soils-based_scRNAseq_Rice/Atrichoblast-compacted.png]

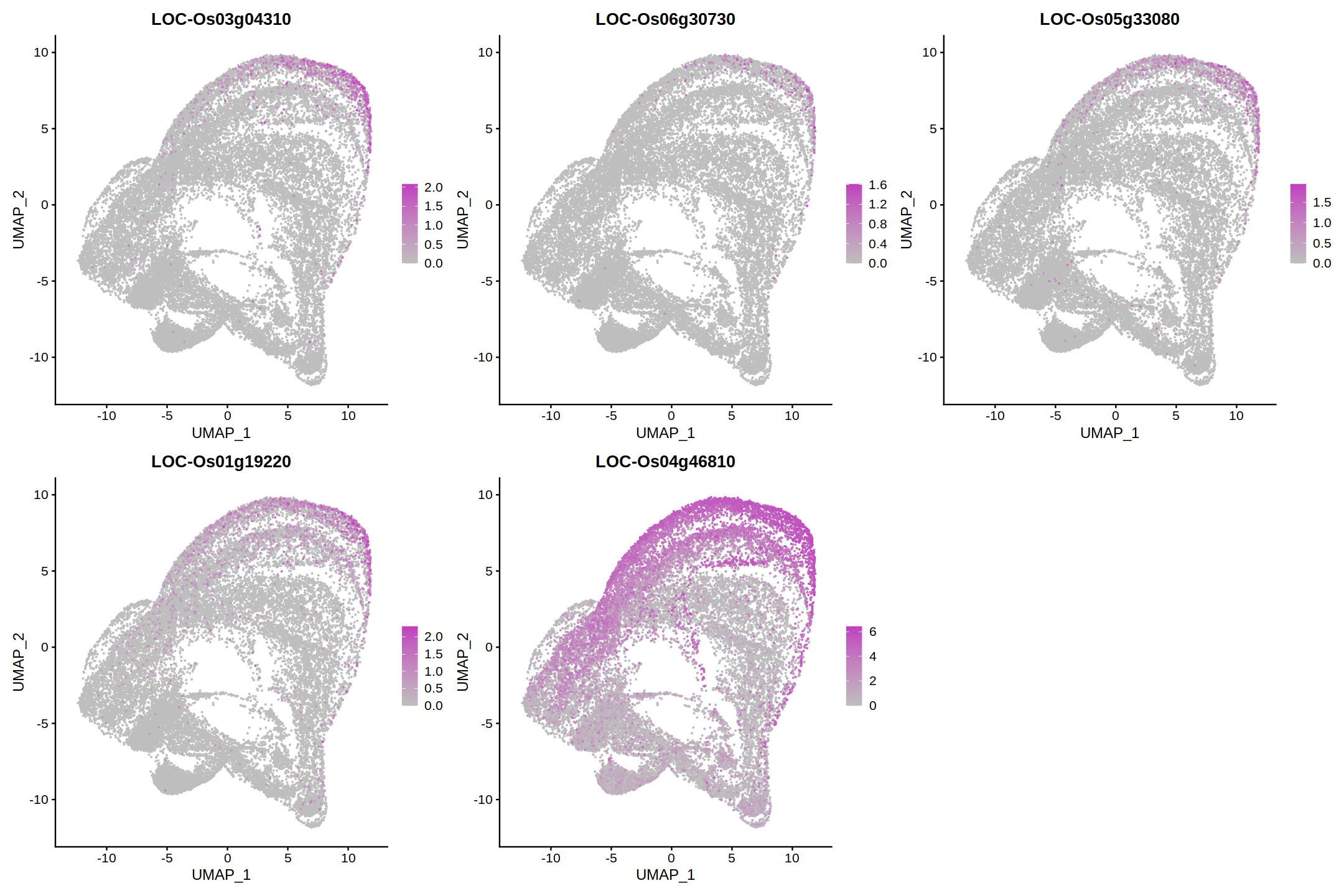

Supplement: Supplementary file 22 — Combined feature plots representing the expression patterns of cell type markers in scRNA-seq data for compacted soil grown roots. Each image represents the gene expressions of markers for one certain cell type. [file 41586_2025_8941_MOESM22_ESM.zip › Supplementary Data 7_Marker_expressions_in_compacted-soils-based_scRNAseq_Rice/Cortex-compacted.png]

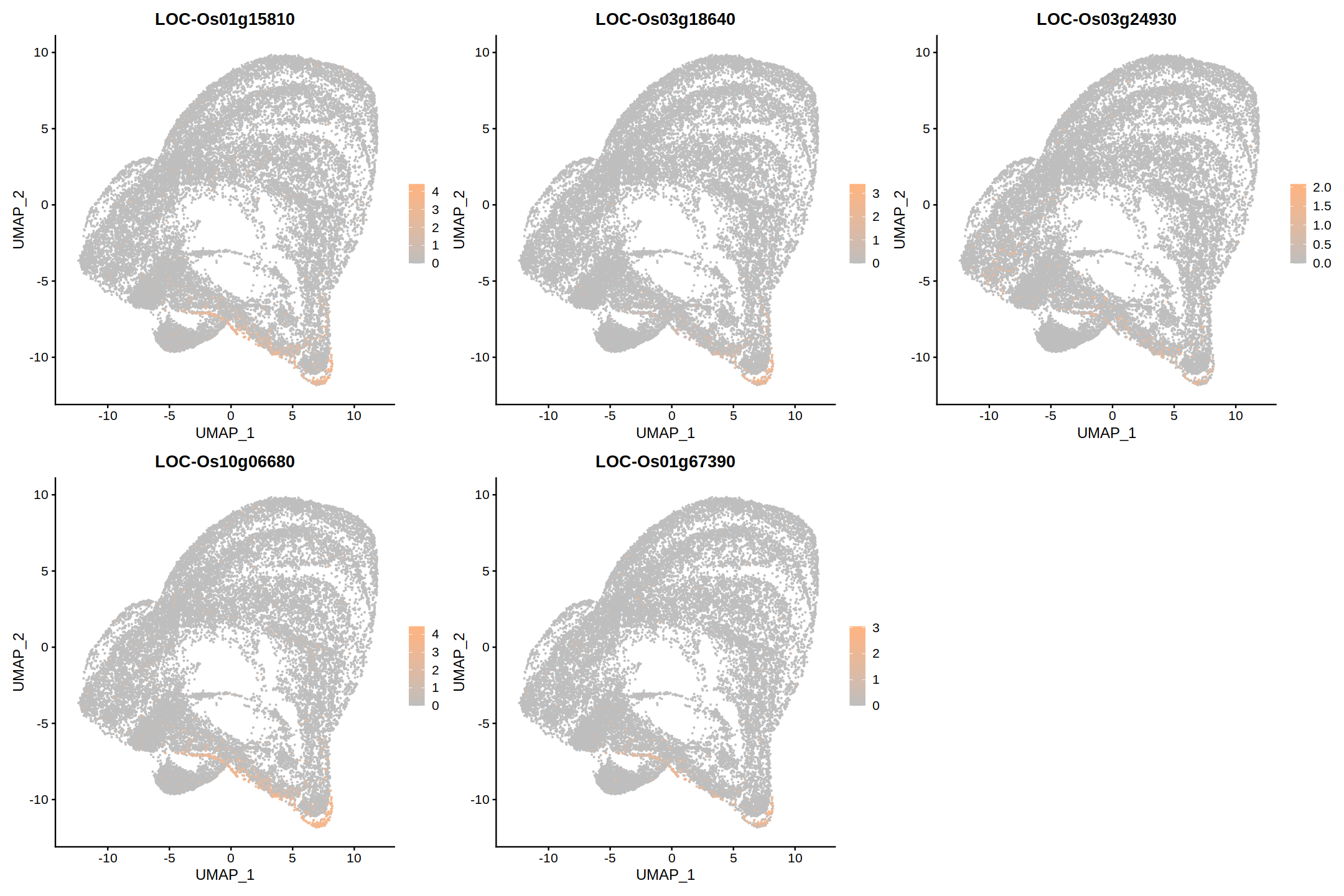

Supplement: Supplementary file 22 — Combined feature plots representing the expression patterns of cell type markers in scRNA-seq data for compacted soil grown roots. Each image represents the gene expressions of markers for one certain cell type. [file 41586_2025_8941_MOESM22_ESM.zip › Supplementary Data 7_Marker_expressions_in_compacted-soils-based_scRNAseq_Rice/Endodermis-compacted.png]

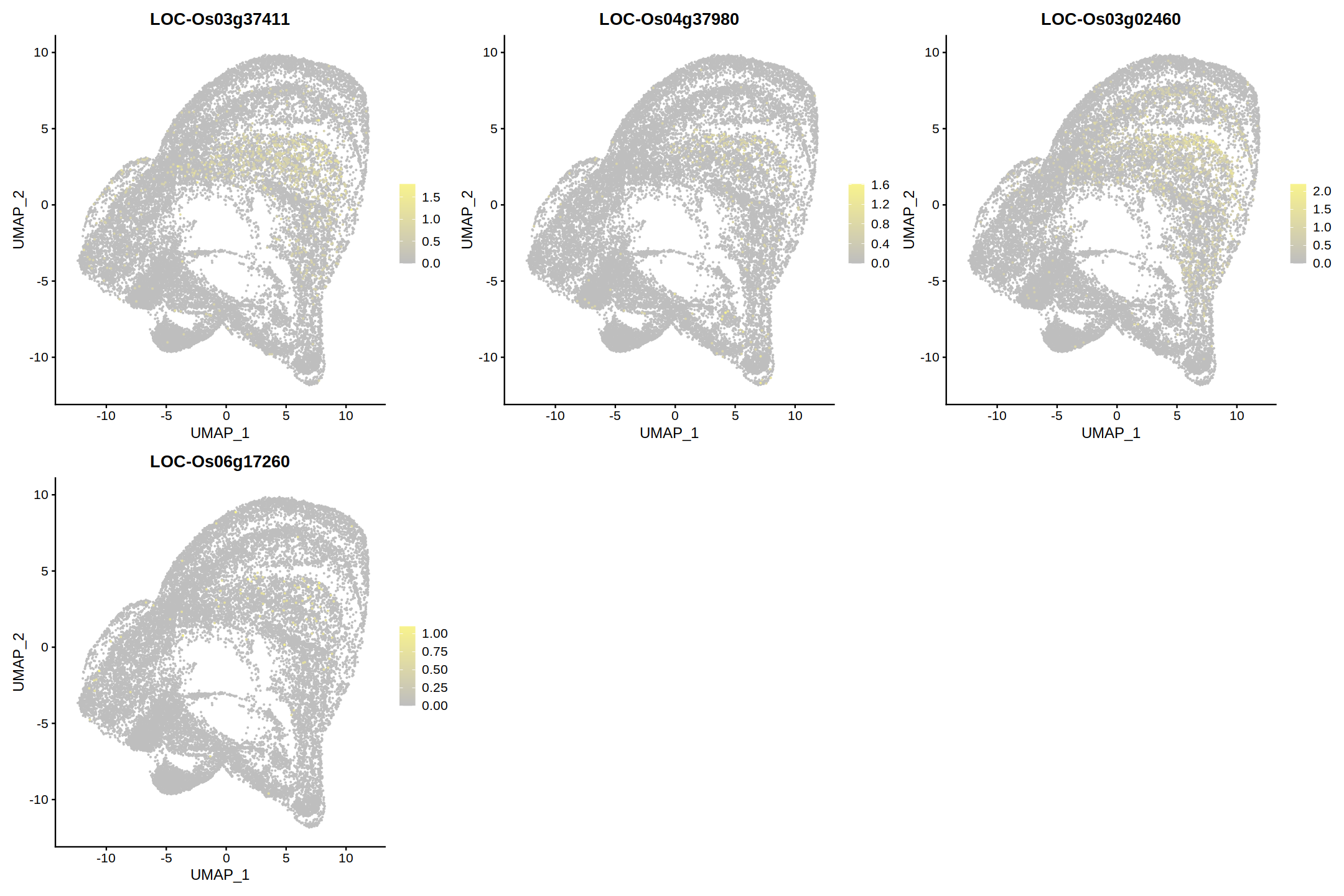

Supplement: Supplementary file 22 — Combined feature plots representing the expression patterns of cell type markers in scRNA-seq data for compacted soil grown roots. Each image represents the gene expressions of markers for one certain cell type. [file 41586_2025_8941_MOESM22_ESM.zip › Supplementary Data 7_Marker_expressions_in_compacted-soils-based_scRNAseq_Rice/Exodermis-Compacted.png]

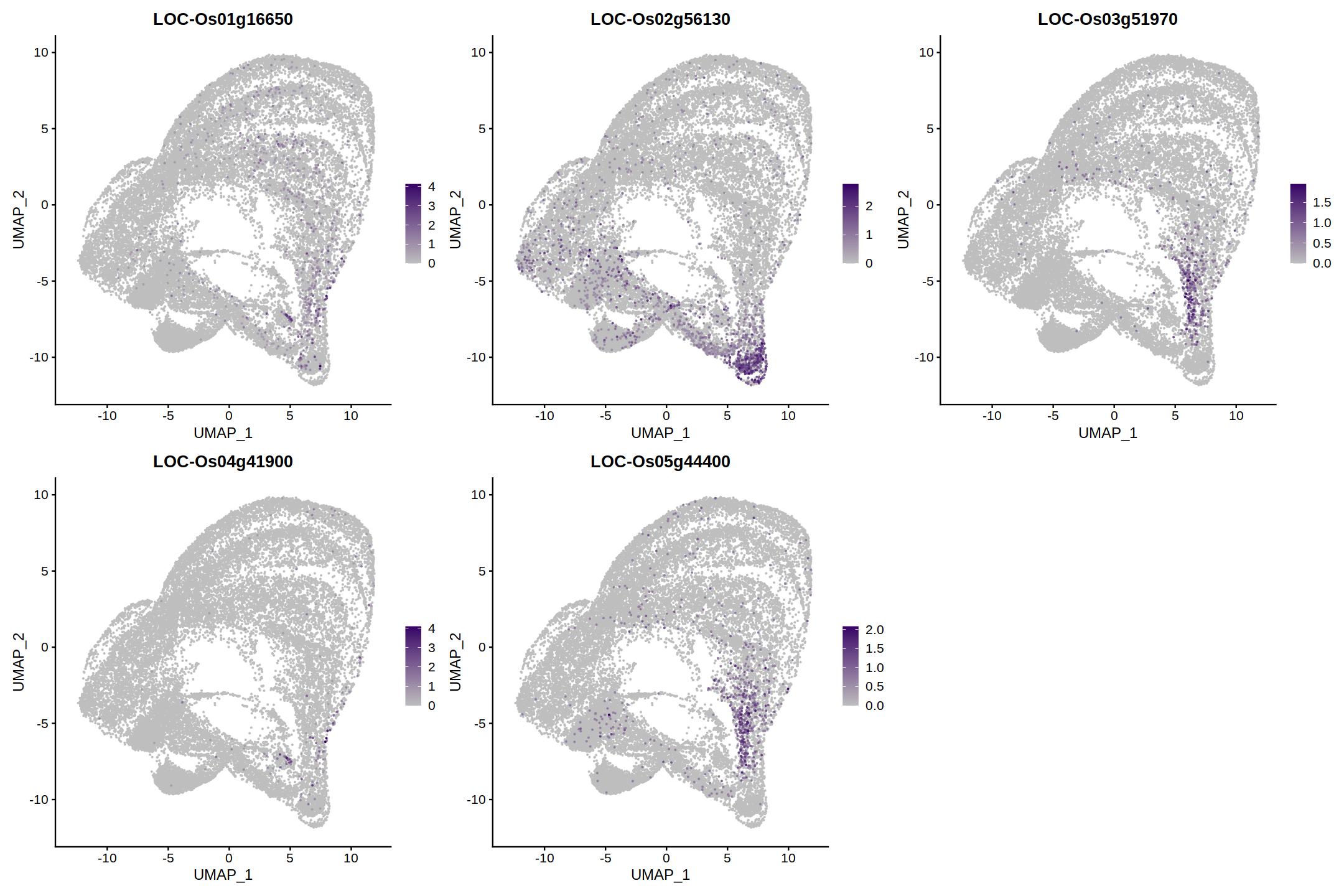

Supplement: Supplementary file 22 — Combined feature plots representing the expression patterns of cell type markers in scRNA-seq data for compacted soil grown roots. Each image represents the gene expressions of markers for one certain cell type. [file 41586_2025_8941_MOESM22_ESM.zip › Supplementary Data 7_Marker_expressions_in_compacted-soils-based_scRNAseq_Rice/Meristem marker-compacted.png]

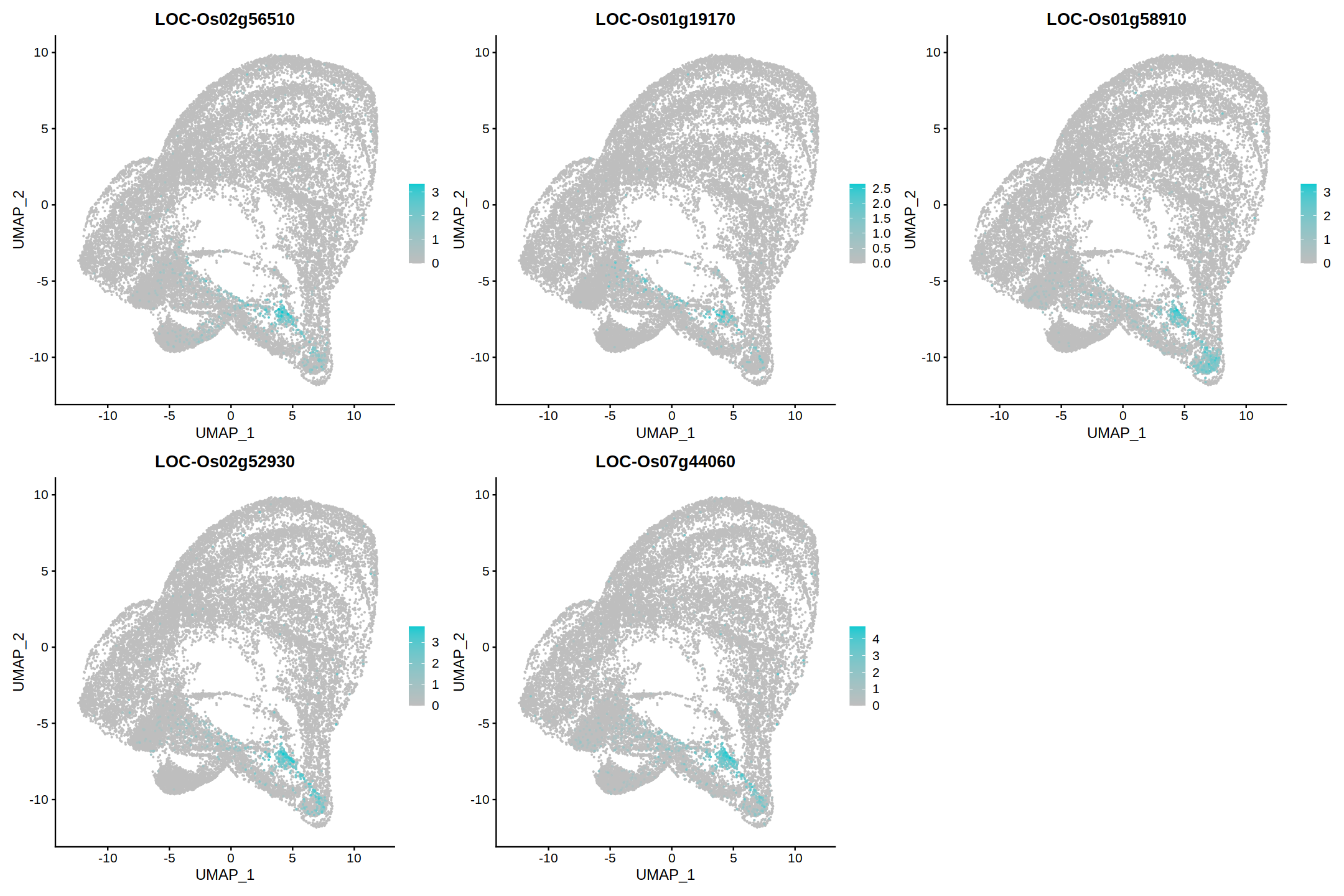

Supplement: Supplementary file 22 — Combined feature plots representing the expression patterns of cell type markers in scRNA-seq data for compacted soil grown roots. Each image represents the gene expressions of markers for one certain cell type. [file 41586_2025_8941_MOESM22_ESM.zip › Supplementary Data 7_Marker_expressions_in_compacted-soils-based_scRNAseq_Rice/Pericycle-compacted.png]

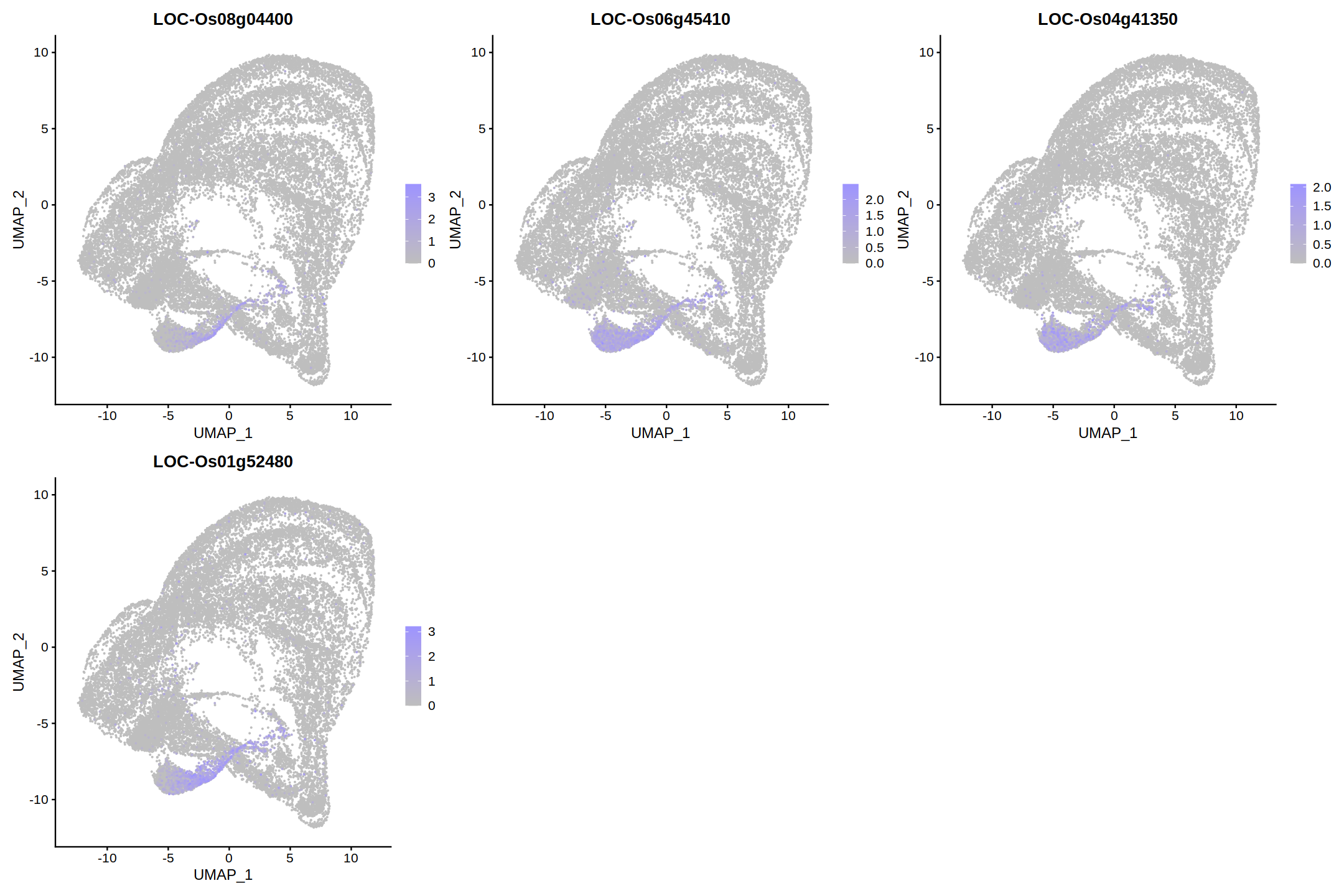

Supplement: Supplementary file 22 — Combined feature plots representing the expression patterns of cell type markers in scRNA-seq data for compacted soil grown roots. Each image represents the gene expressions of markers for one certain cell type. [file 41586_2025_8941_MOESM22_ESM.zip › Supplementary Data 7_Marker_expressions_in_compacted-soils-based_scRNAseq_Rice/Phloem-compacted.png]

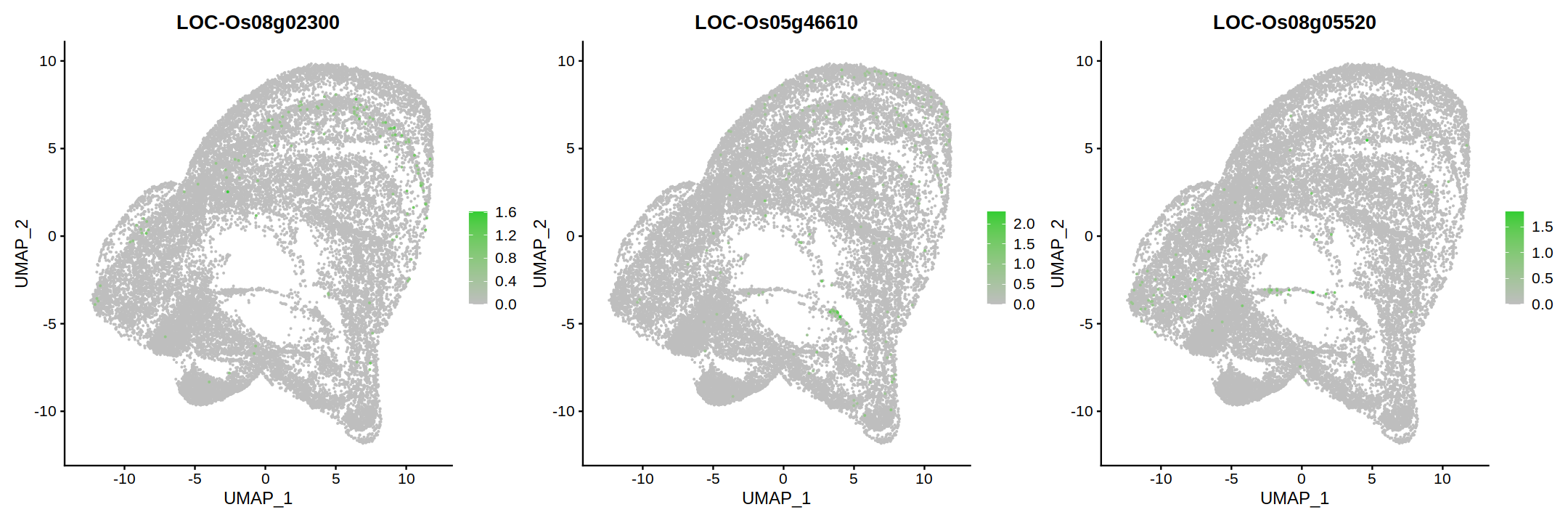

Supplement: Supplementary file 22 — Combined feature plots representing the expression patterns of cell type markers in scRNA-seq data for compacted soil grown roots. Each image represents the gene expressions of markers for one certain cell type. [file 41586_2025_8941_MOESM22_ESM.zip › Supplementary Data 7_Marker_expressions_in_compacted-soils-based_scRNAseq_Rice/Sclerenchyma-compacted.png]

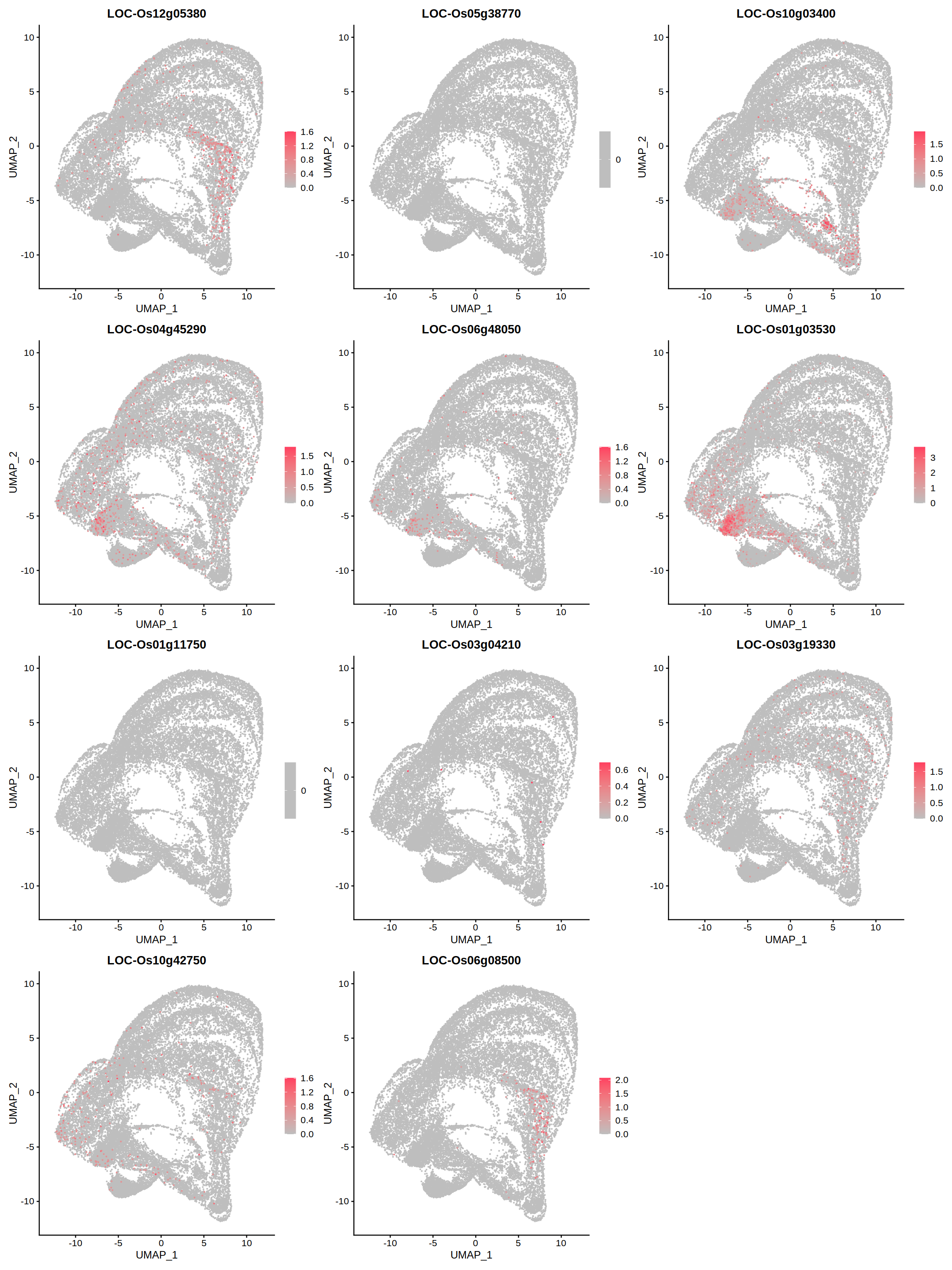

Supplement: Supplementary file 22 — Combined feature plots representing the expression patterns of cell type markers in scRNA-seq data for compacted soil grown roots. Each image represents the gene expressions of markers for one certain cell type. [file 41586_2025_8941_MOESM22_ESM.zip › Supplementary Data 7_Marker_expressions_in_compacted-soils-based_scRNAseq_Rice/Trichoblast-compacted.png]

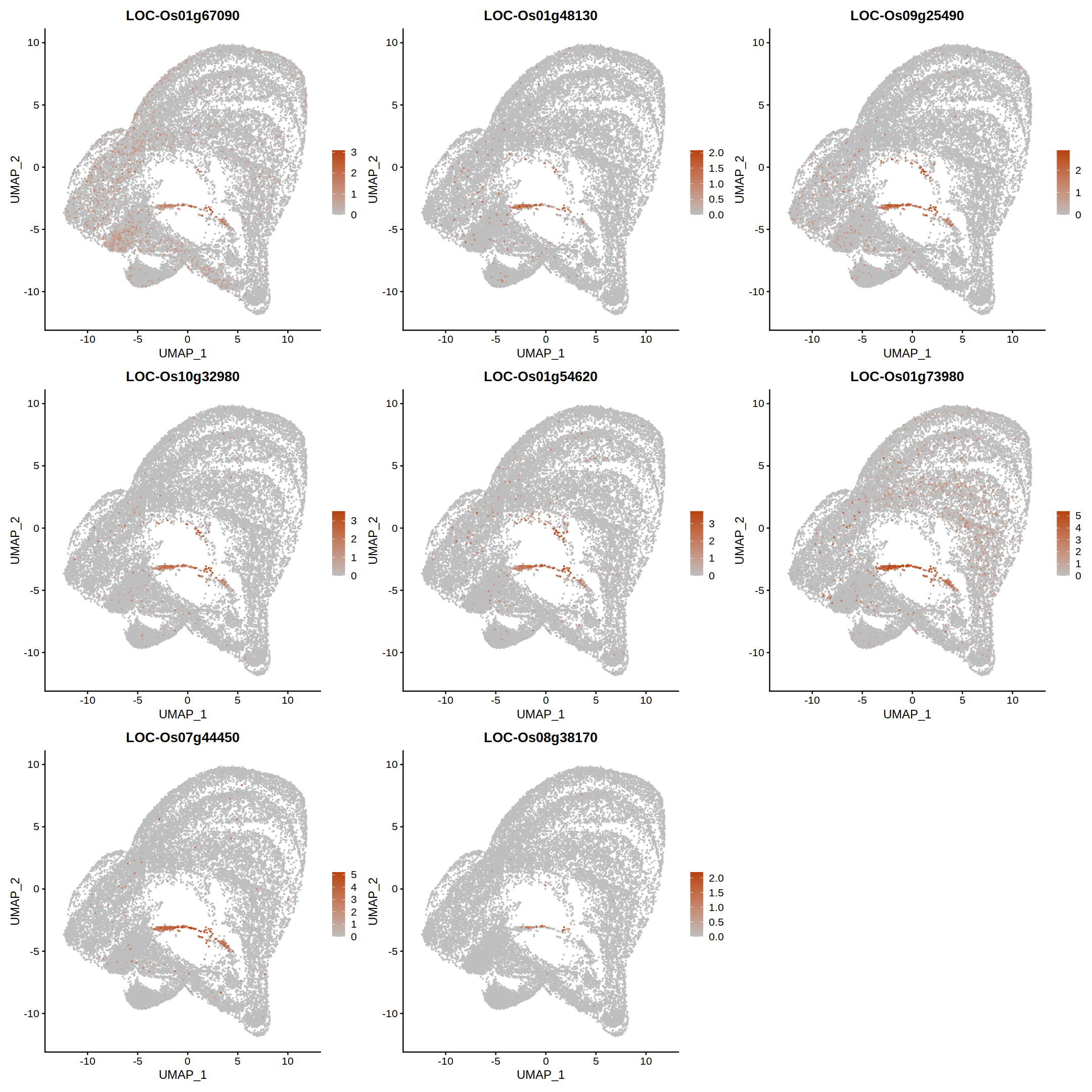

Supplement: Supplementary file 22 — Combined feature plots representing the expression patterns of cell type markers in scRNA-seq data for compacted soil grown roots. Each image represents the gene expressions of markers for one certain cell type. [file 41586_2025_8941_MOESM22_ESM.zip › Supplementary Data 7_Marker_expressions_in_compacted-soils-based_scRNAseq_Rice/Xylem-compacted.png]

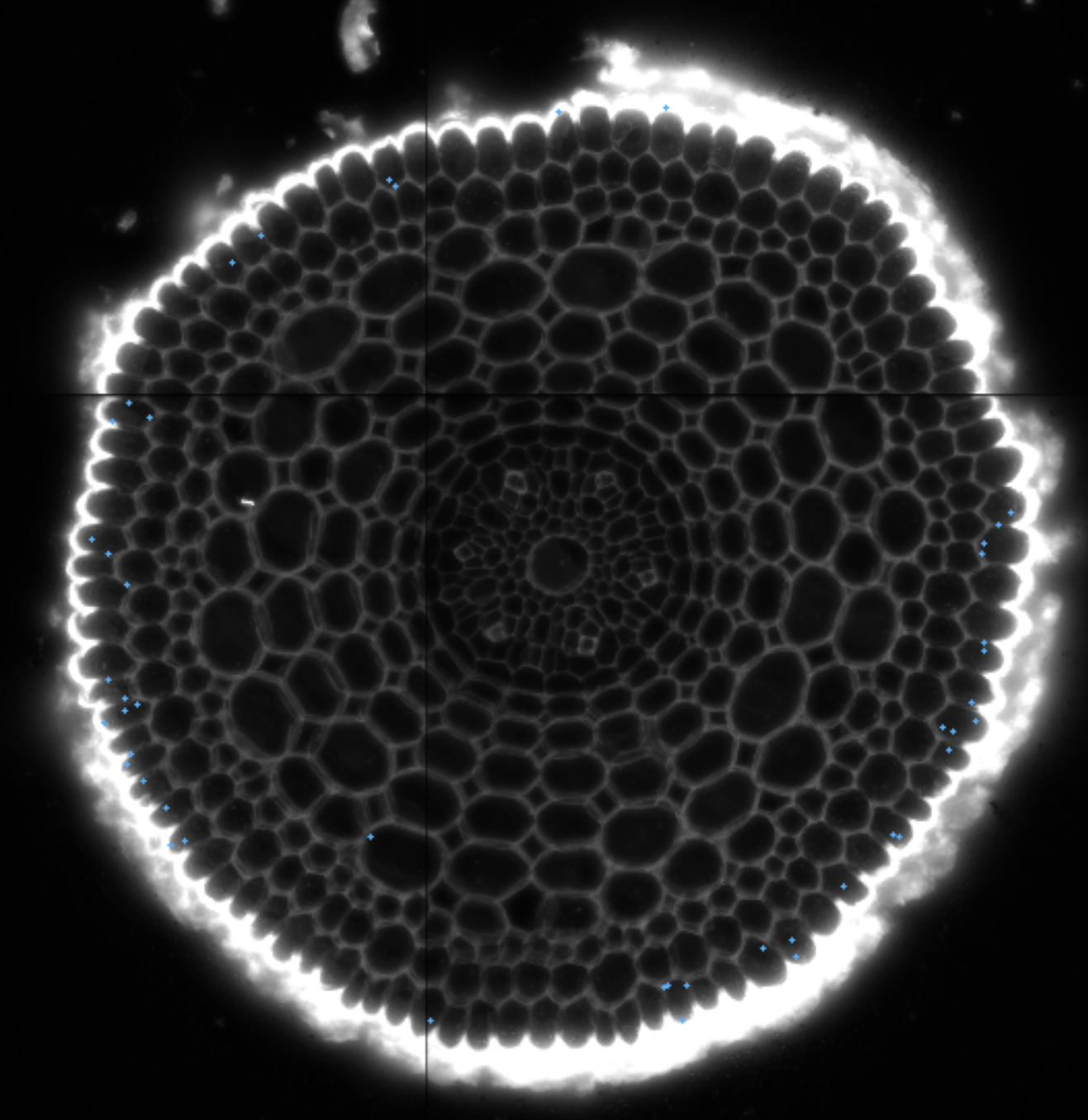

Supplement: Supplementary file 23 — Expression patterns of cell type markers in spatial transcriptomics data for compacted soil grown roots. A PDF summary file that includes the sample and gene information for visualization is included. The raw spatial transcriptomics data for compacted soil grown roots is also included. [file 41586_2025_8941_MOESM23_ESM.zip › Supplementary Data 8_Marker_expression_in_compacted-soils-based_Spatial_transcriptomics_Rice/Atrichoblast-LOC_Os01g50820.png]

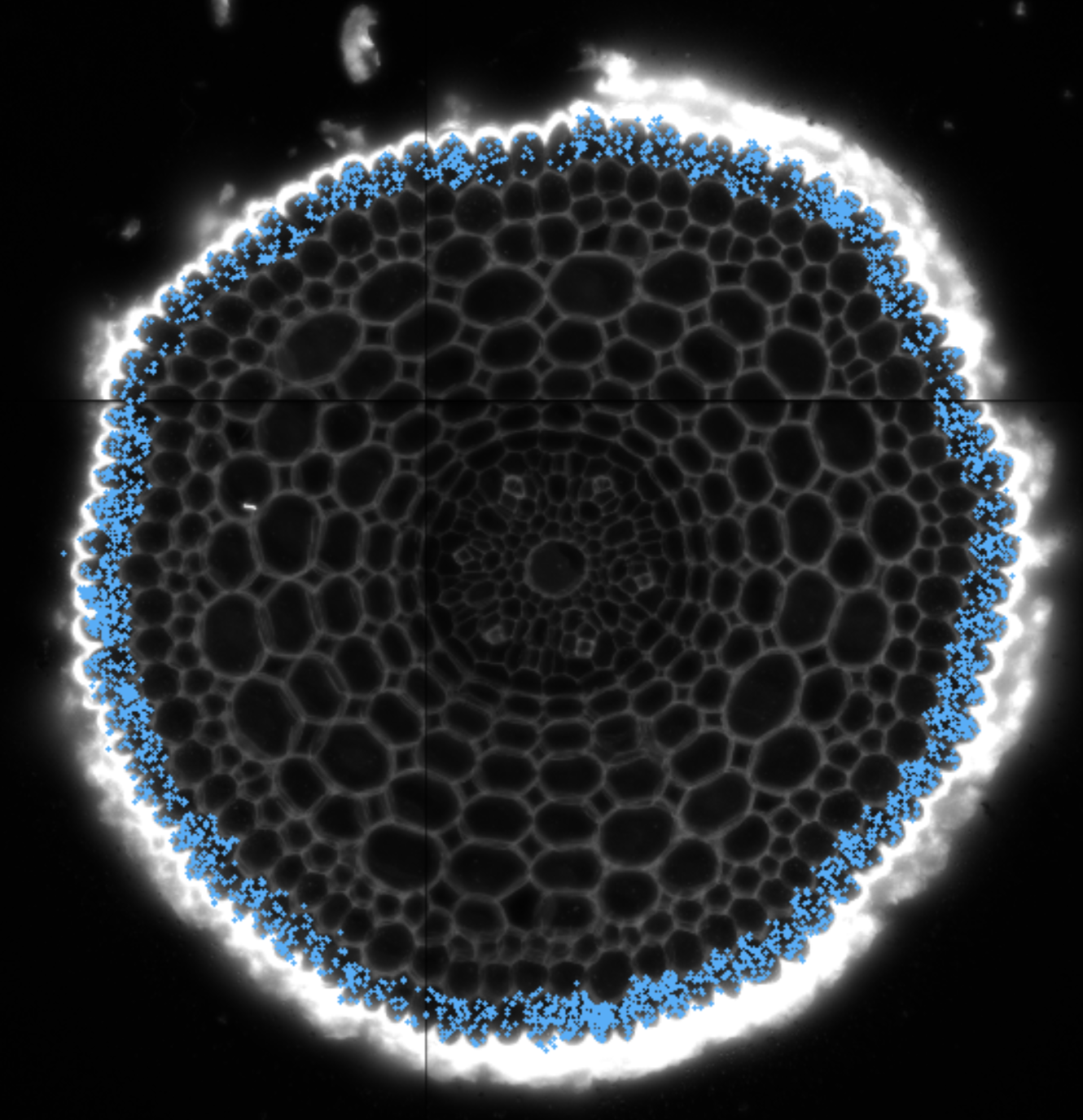

Supplement: Supplementary file 23 — Expression patterns of cell type markers in spatial transcriptomics data for compacted soil grown roots. A PDF summary file that includes the sample and gene information for visualization is included. The raw spatial transcriptomics data for compacted soil grown roots is also included. [file 41586_2025_8941_MOESM23_ESM.zip › Supplementary Data 8_Marker_expression_in_compacted-soils-based_Spatial_transcriptomics_Rice/Atrichoblast-LOC_Os01g64840.png]

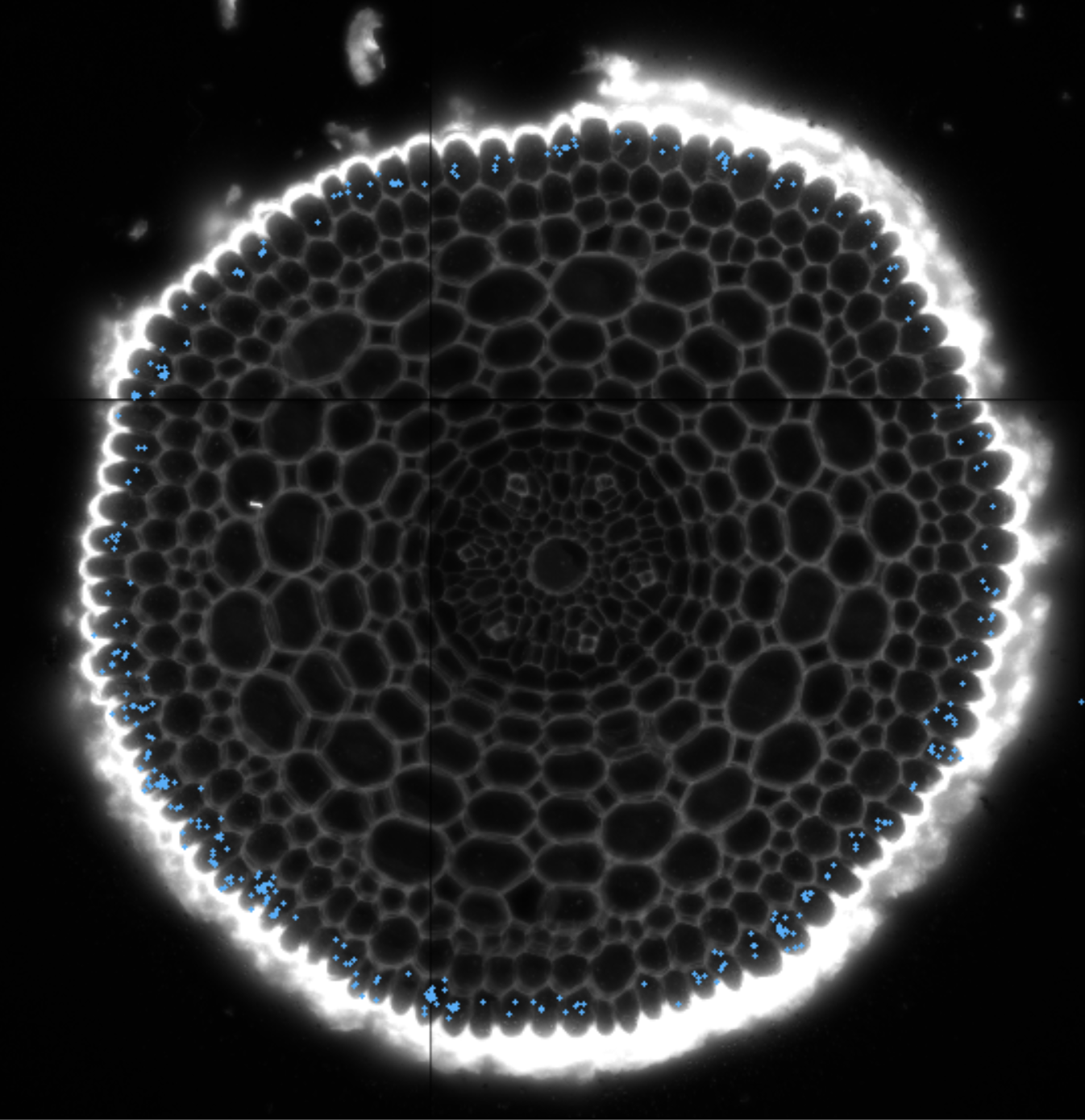

Supplement: Supplementary file 23 — Expression patterns of cell type markers in spatial transcriptomics data for compacted soil grown roots. A PDF summary file that includes the sample and gene information for visualization is included. The raw spatial transcriptomics data for compacted soil grown roots is also included. [file 41586_2025_8941_MOESM23_ESM.zip › Supplementary Data 8_Marker_expression_in_compacted-soils-based_Spatial_transcriptomics_Rice/Atrichoblast-LOC_Os03g09970.png]

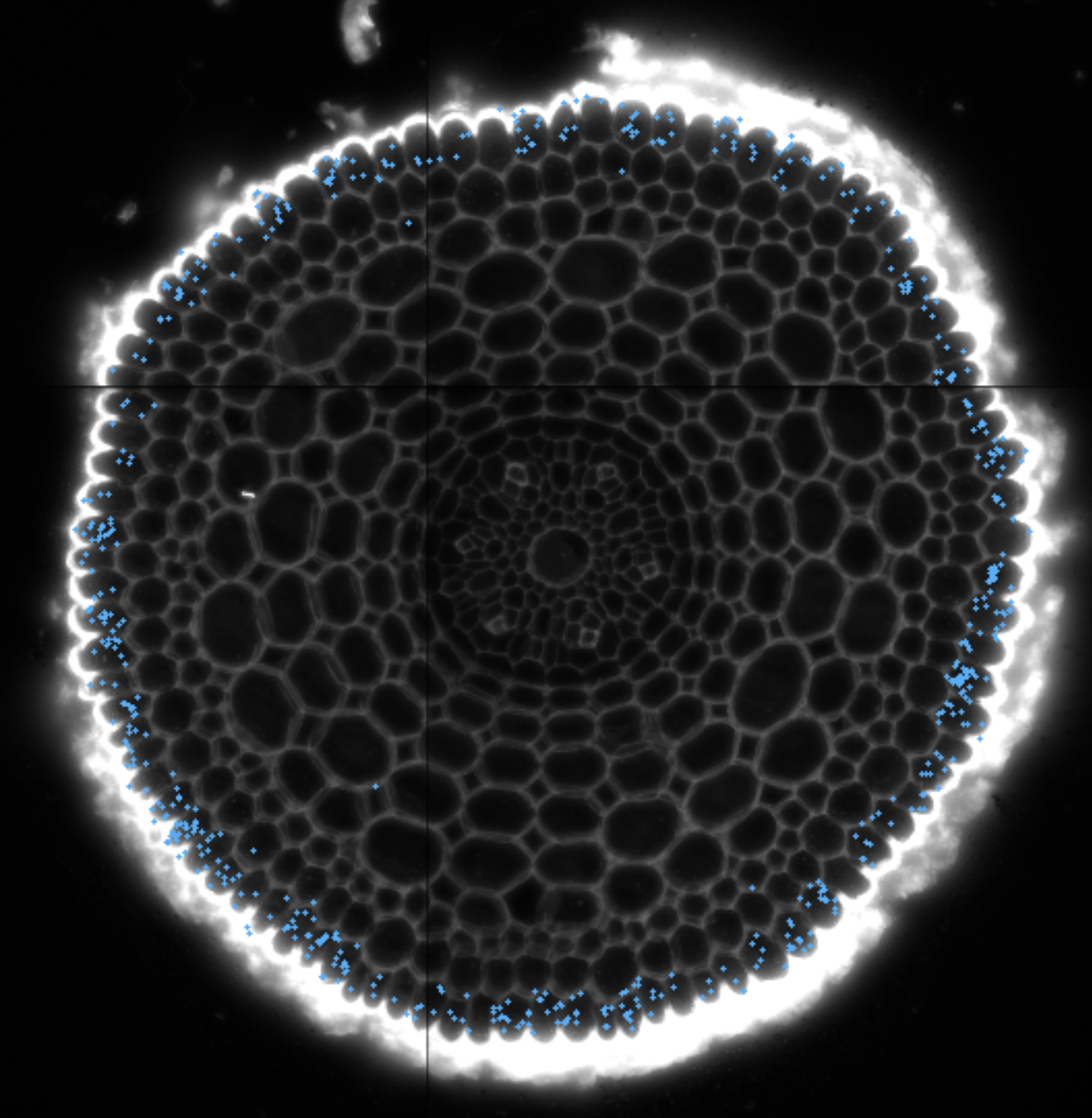

Supplement: Supplementary file 23 — Expression patterns of cell type markers in spatial transcriptomics data for compacted soil grown roots. A PDF summary file that includes the sample and gene information for visualization is included. The raw spatial transcriptomics data for compacted soil grown roots is also included. [file 41586_2025_8941_MOESM23_ESM.zip › Supplementary Data 8_Marker_expression_in_compacted-soils-based_Spatial_transcriptomics_Rice/Atrichoblast-LOC_Os03g19990.png]

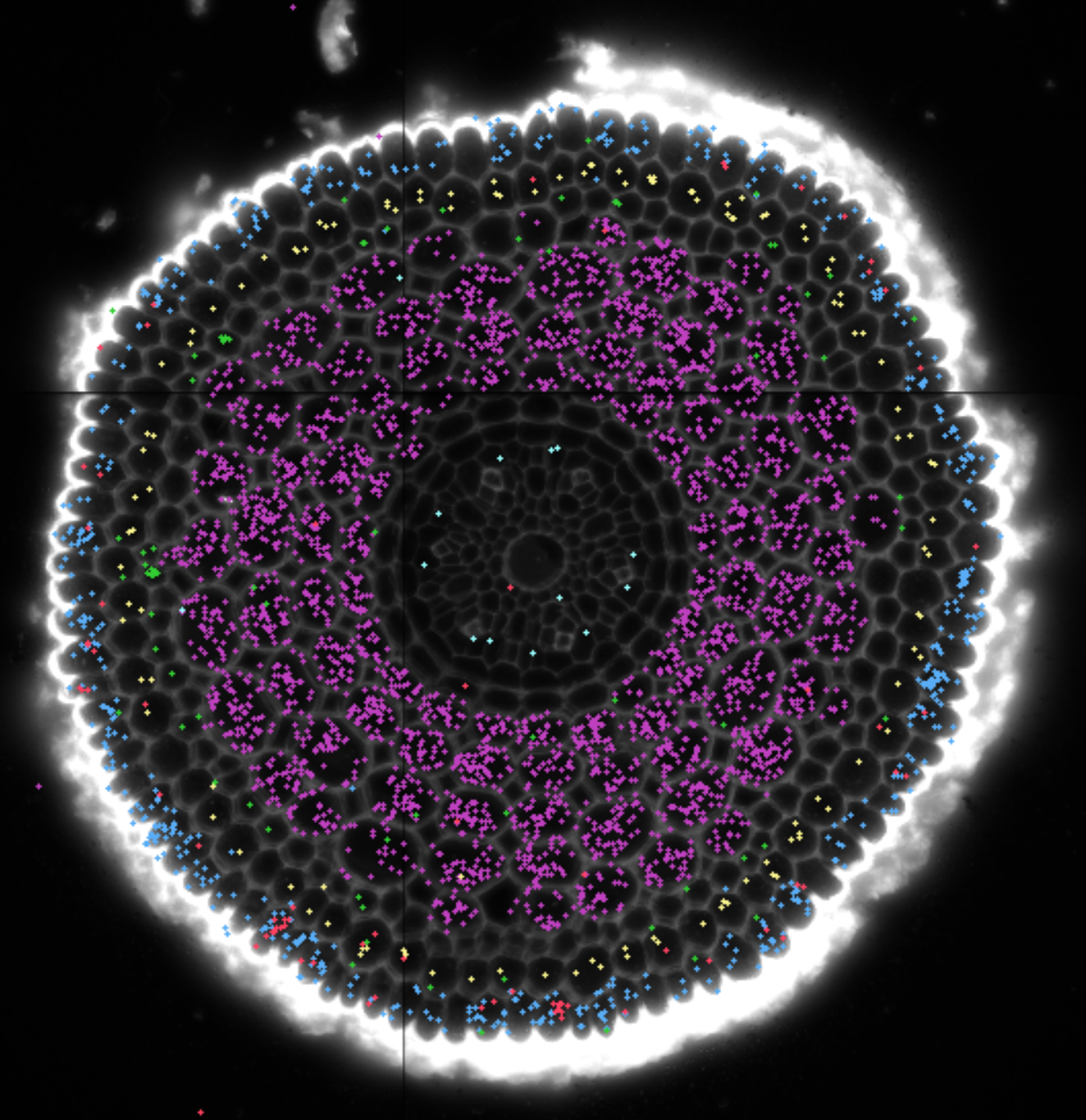

Supplement: Supplementary file 23 — Expression patterns of cell type markers in spatial transcriptomics data for compacted soil grown roots. A PDF summary file that includes the sample and gene information for visualization is included. The raw spatial transcriptomics data for compacted soil grown roots is also included. [file 41586_2025_8941_MOESM23_ESM.zip › Supplementary Data 8_Marker_expression_in_compacted-soils-based_Spatial_transcriptomics_Rice/Compacted-selected good markers.png]

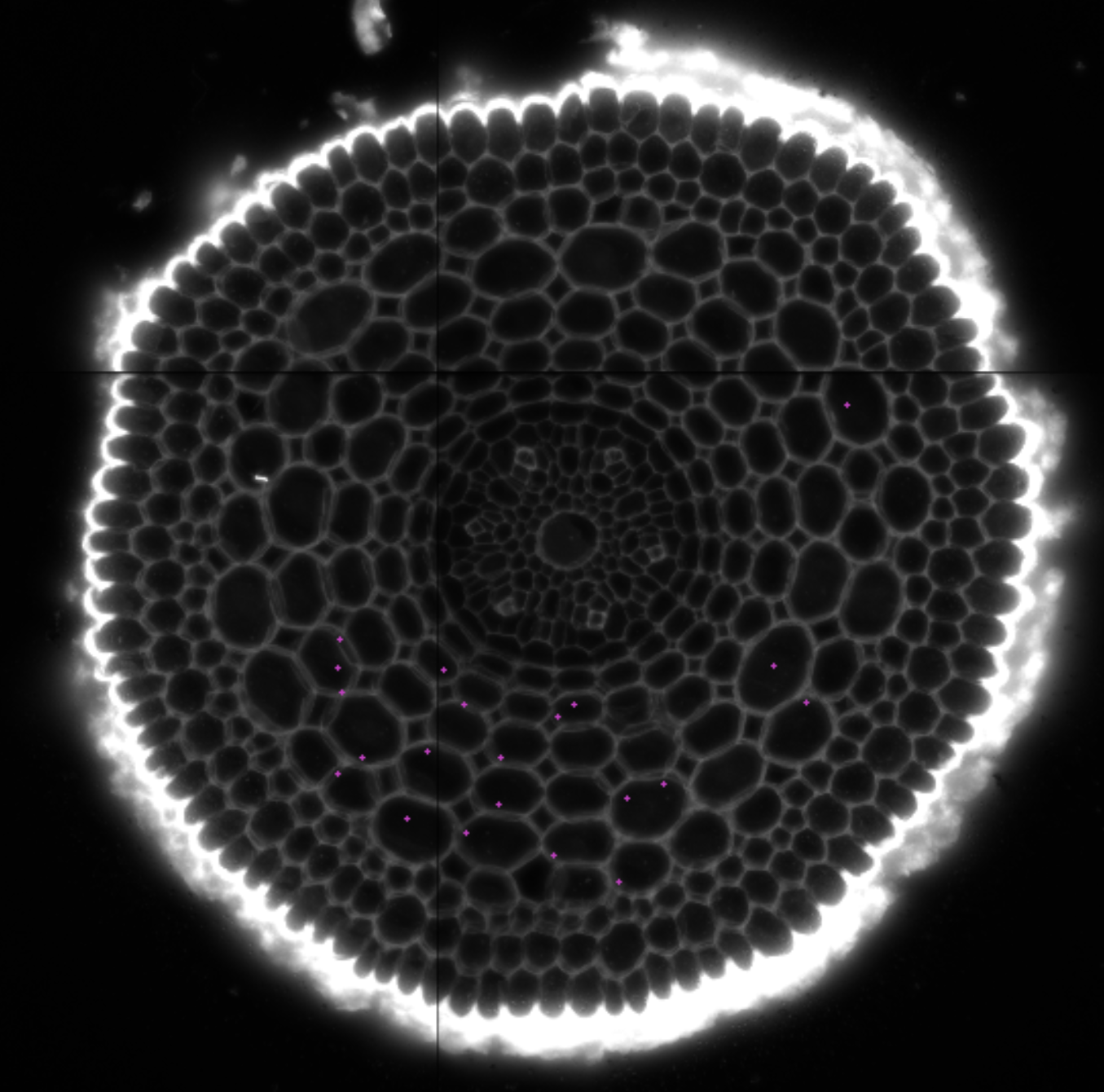

Supplement: Supplementary file 23 — Expression patterns of cell type markers in spatial transcriptomics data for compacted soil grown roots. A PDF summary file that includes the sample and gene information for visualization is included. The raw spatial transcriptomics data for compacted soil grown roots is also included. [file 41586_2025_8941_MOESM23_ESM.zip › Supplementary Data 8_Marker_expression_in_compacted-soils-based_Spatial_transcriptomics_Rice/Cortex-LOC_Os01g19220.png]

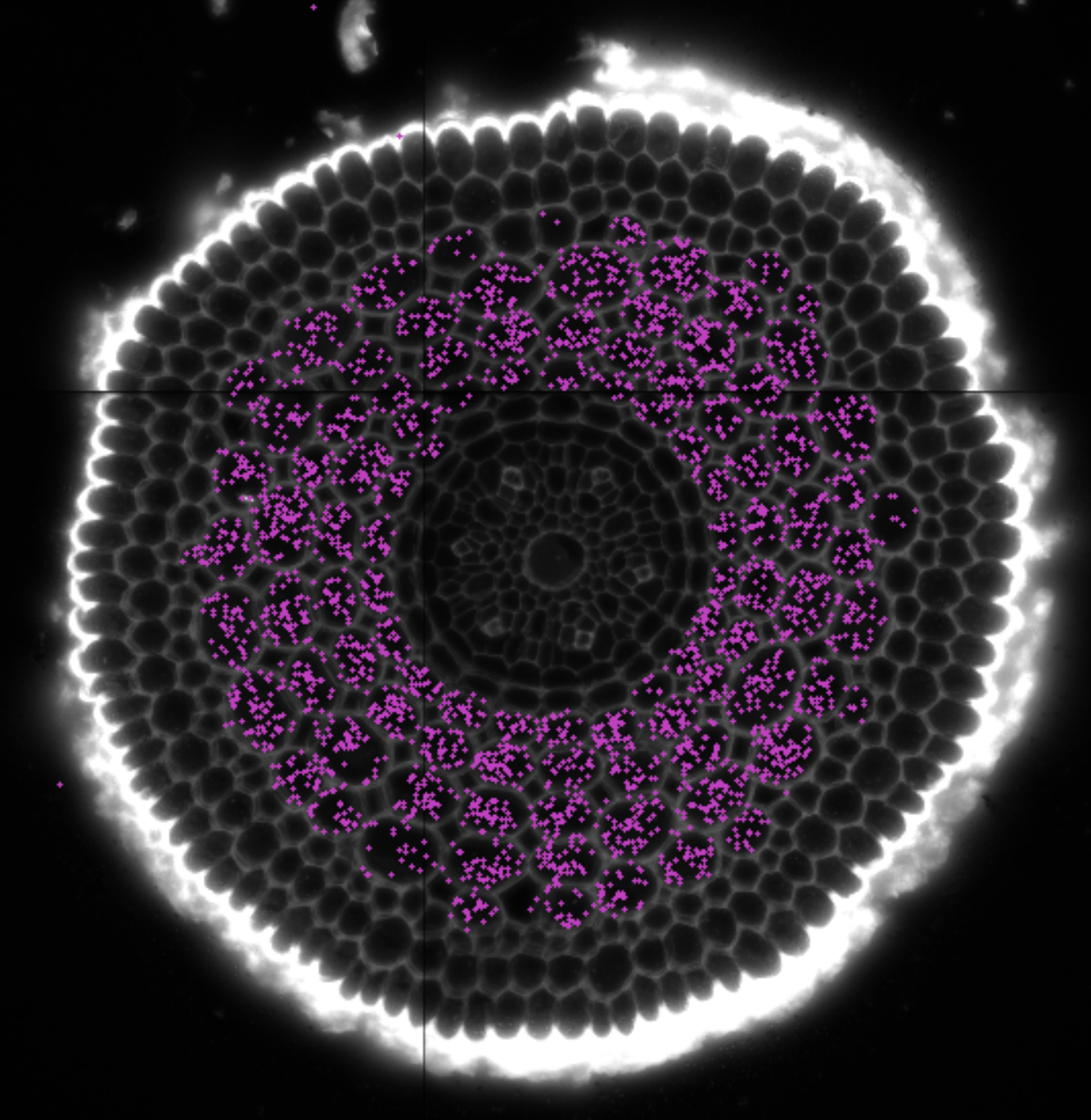

Supplement: Supplementary file 23 — Expression patterns of cell type markers in spatial transcriptomics data for compacted soil grown roots. A PDF summary file that includes the sample and gene information for visualization is included. The raw spatial transcriptomics data for compacted soil grown roots is also included. [file 41586_2025_8941_MOESM23_ESM.zip › Supplementary Data 8_Marker_expression_in_compacted-soils-based_Spatial_transcriptomics_Rice/Cortex-LOC_Os03g04310.png]

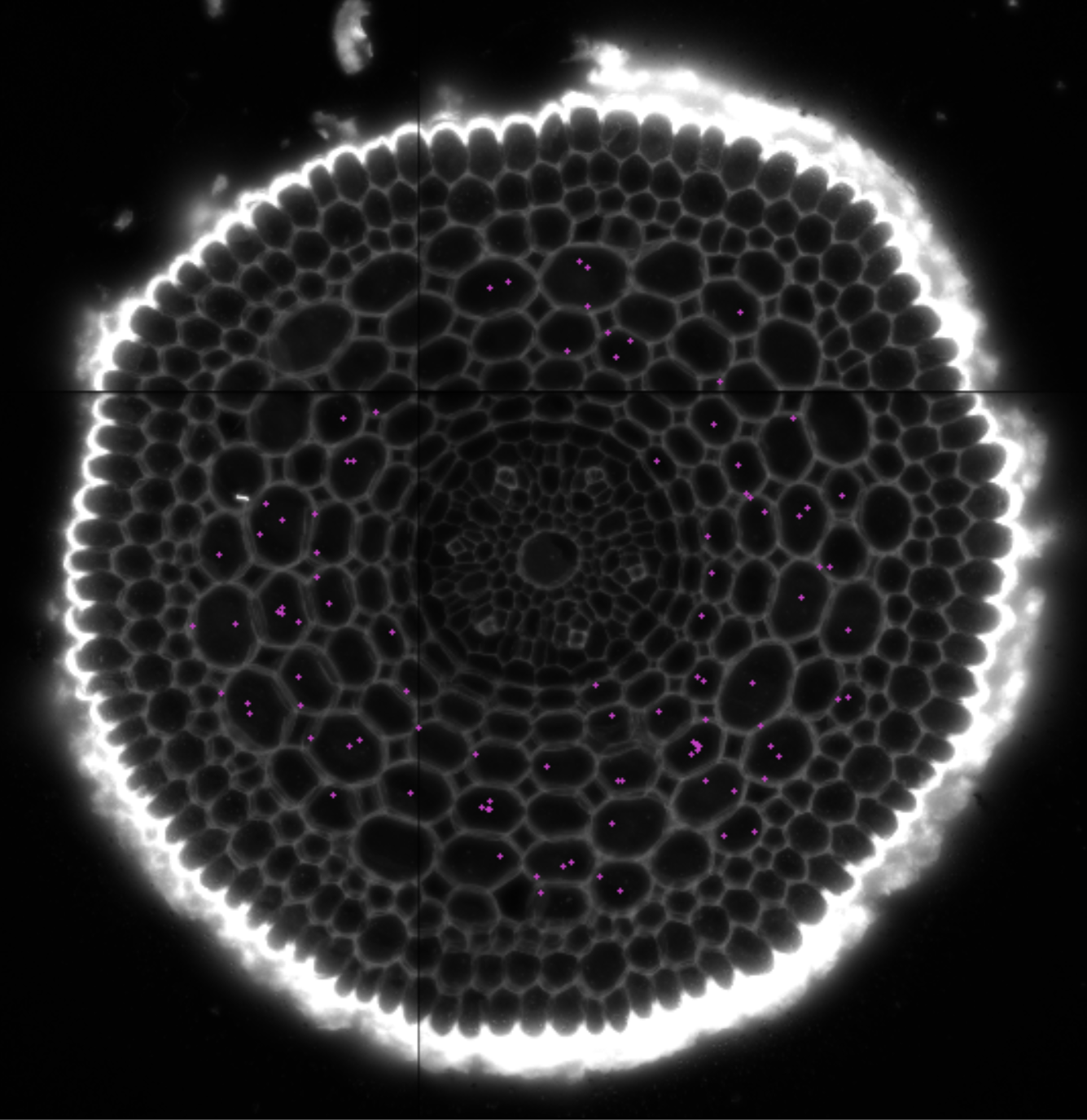

Supplement: Supplementary file 23 — Expression patterns of cell type markers in spatial transcriptomics data for compacted soil grown roots. A PDF summary file that includes the sample and gene information for visualization is included. The raw spatial transcriptomics data for compacted soil grown roots is also included. [file 41586_2025_8941_MOESM23_ESM.zip › Supplementary Data 8_Marker_expression_in_compacted-soils-based_Spatial_transcriptomics_Rice/Cortex-LOC_Os05g33080.png]

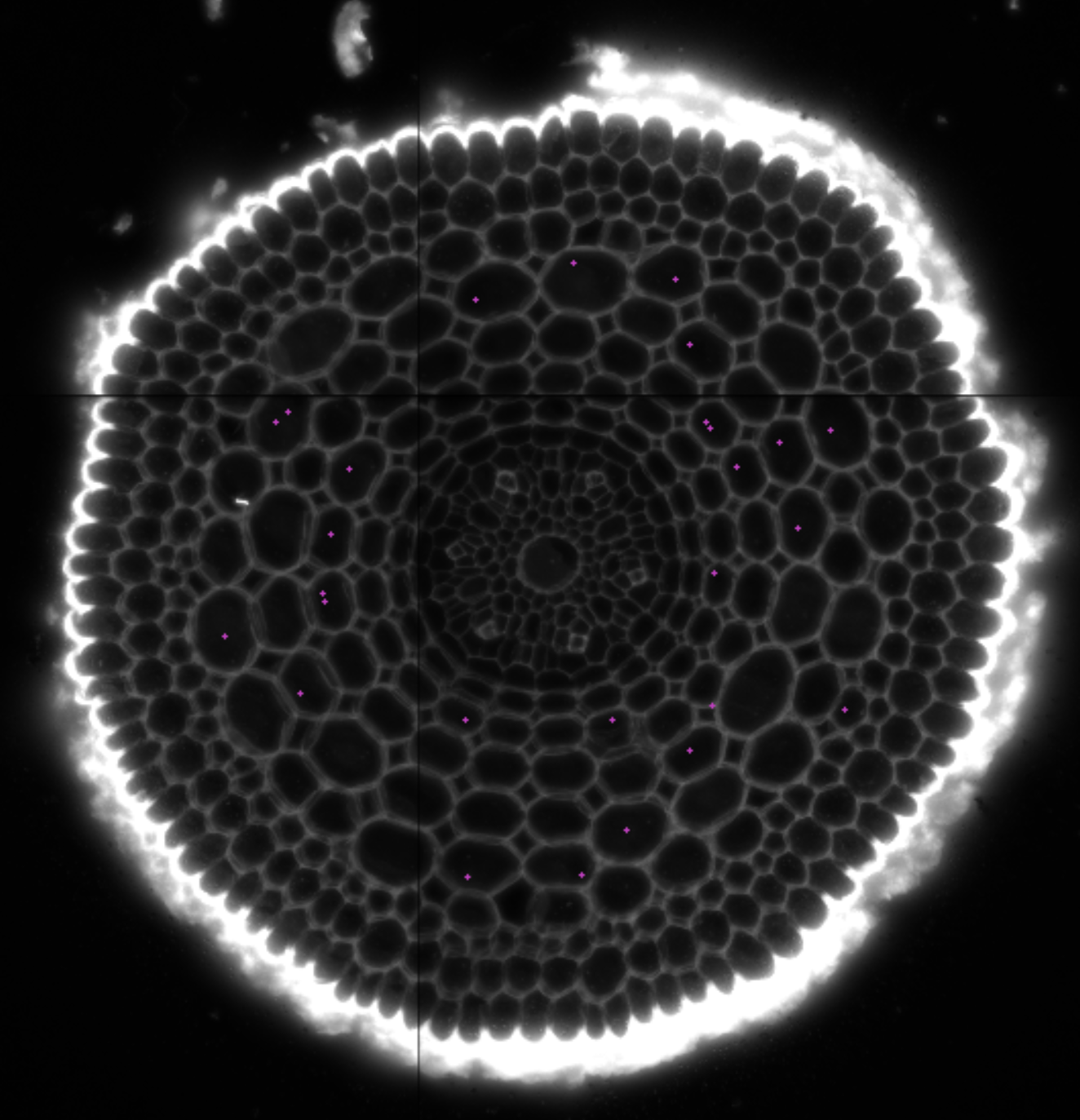

Supplement: Supplementary file 23 — Expression patterns of cell type markers in spatial transcriptomics data for compacted soil grown roots. A PDF summary file that includes the sample and gene information for visualization is included. The raw spatial transcriptomics data for compacted soil grown roots is also included. [file 41586_2025_8941_MOESM23_ESM.zip › Supplementary Data 8_Marker_expression_in_compacted-soils-based_Spatial_transcriptomics_Rice/Cortex-LOC_Os06g30730.png]

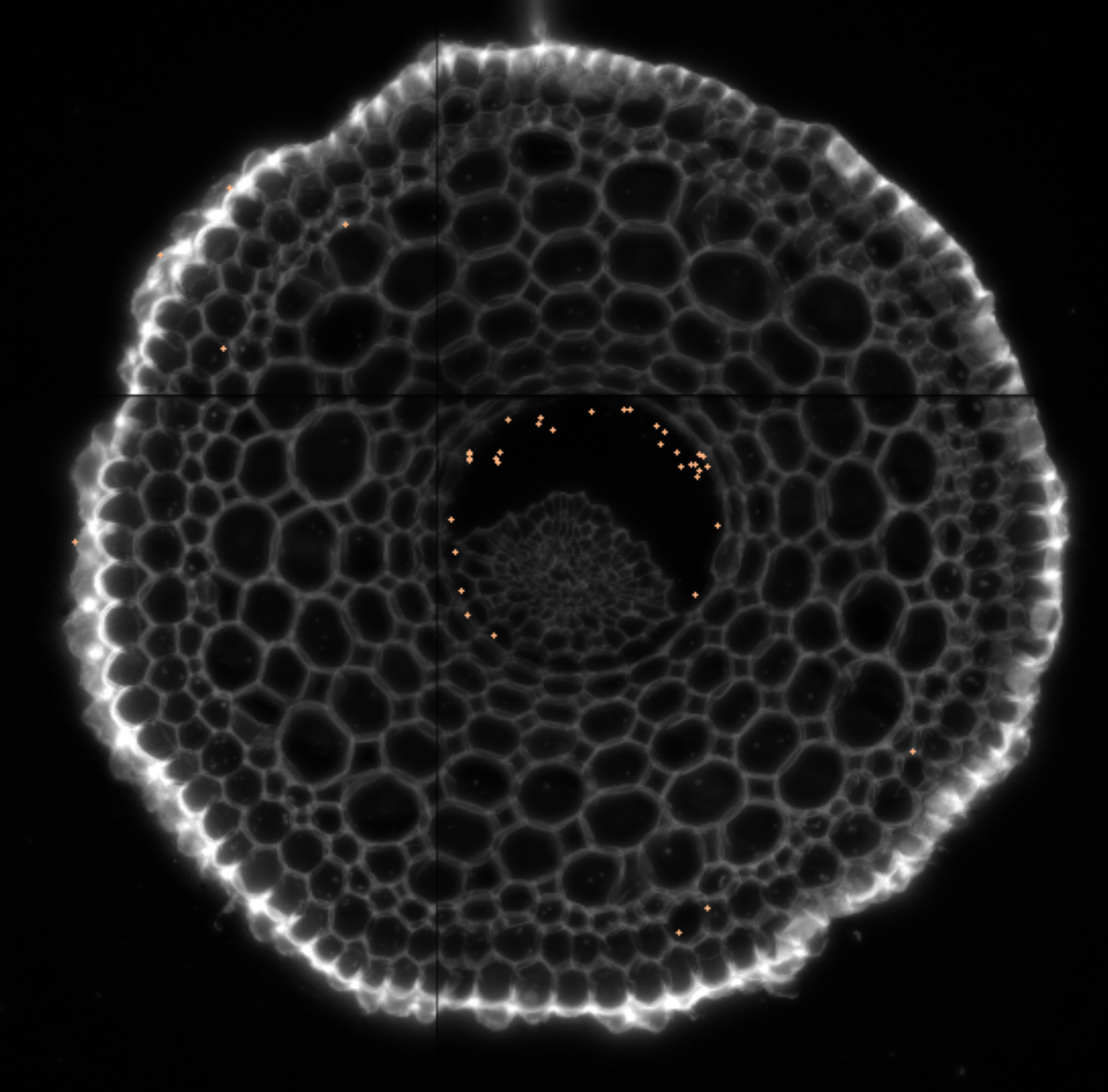

Supplement: Supplementary file 23 — Expression patterns of cell type markers in spatial transcriptomics data for compacted soil grown roots. A PDF summary file that includes the sample and gene information for visualization is included. The raw spatial transcriptomics data for compacted soil grown roots is also included. [file 41586_2025_8941_MOESM23_ESM.zip › Supplementary Data 8_Marker_expression_in_compacted-soils-based_Spatial_transcriptomics_Rice/Endodermis-LOC_Os01g15810.png]

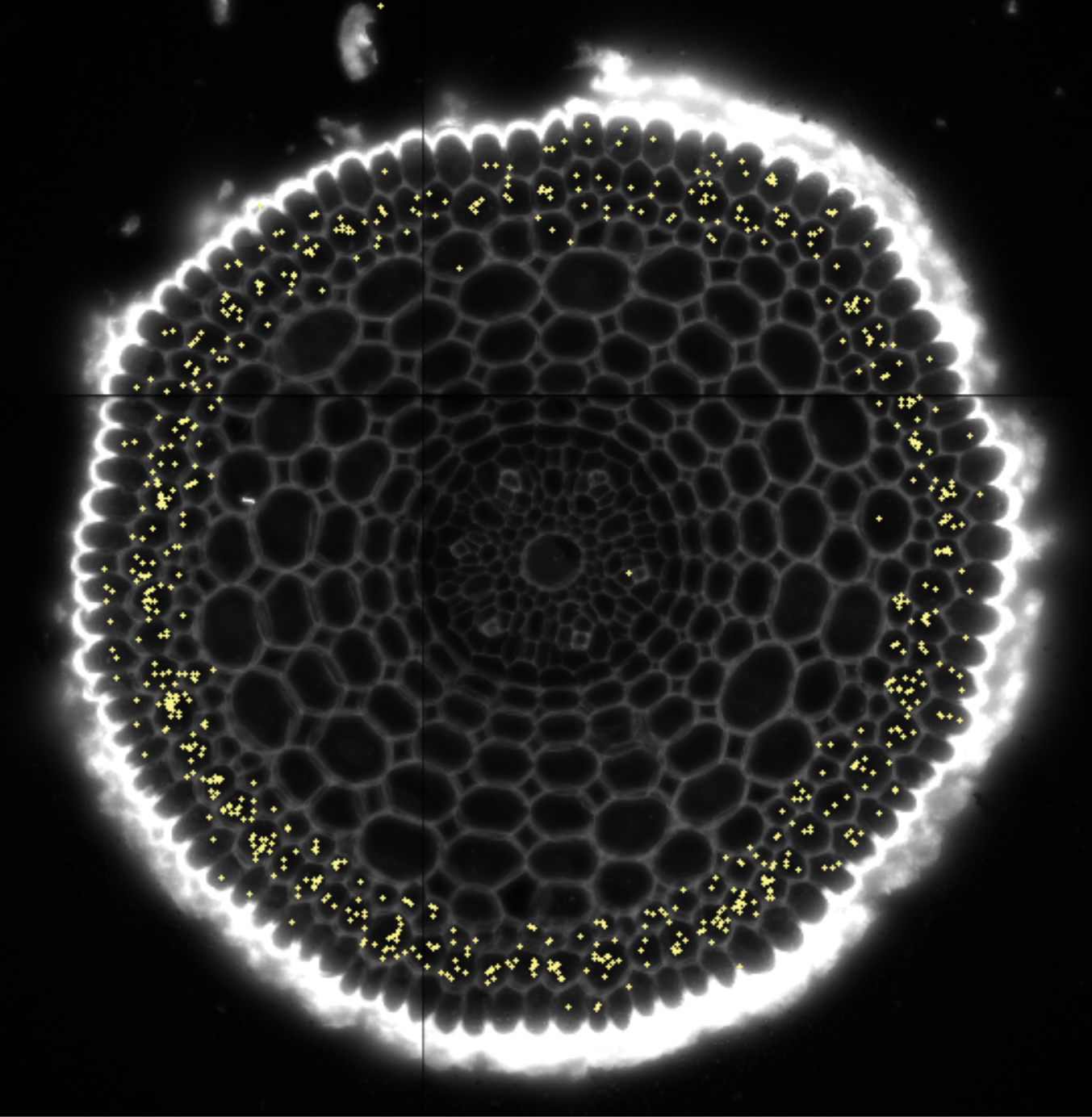

Supplement: Supplementary file 23 — Expression patterns of cell type markers in spatial transcriptomics data for compacted soil grown roots. A PDF summary file that includes the sample and gene information for visualization is included. The raw spatial transcriptomics data for compacted soil grown roots is also included. [file 41586_2025_8941_MOESM23_ESM.zip › Supplementary Data 8_Marker_expression_in_compacted-soils-based_Spatial_transcriptomics_Rice/Exodermis-LOC_Os03g02460.png]

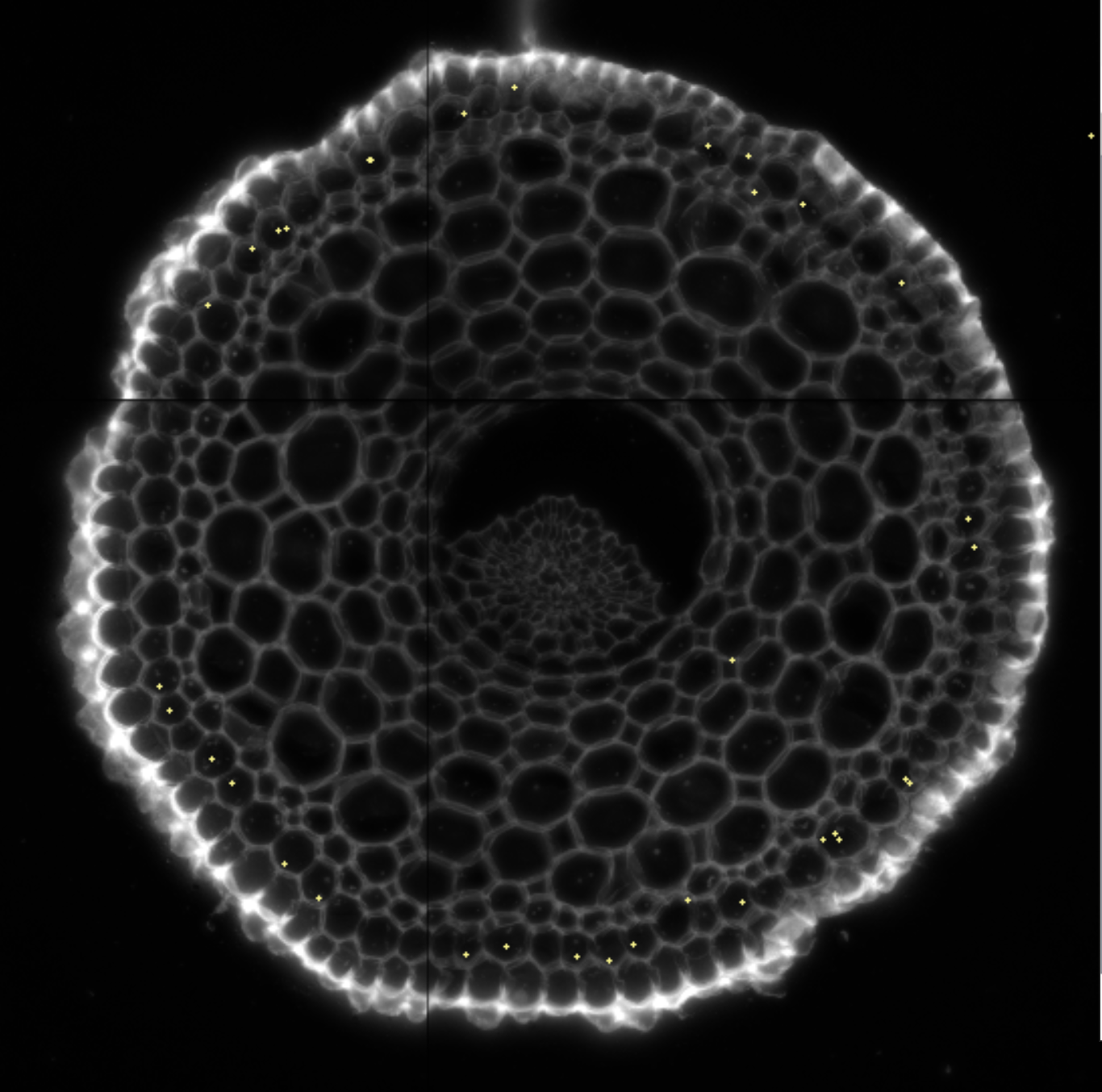

Supplement: Supplementary file 23 — Expression patterns of cell type markers in spatial transcriptomics data for compacted soil grown roots. A PDF summary file that includes the sample and gene information for visualization is included. The raw spatial transcriptomics data for compacted soil grown roots is also included. [file 41586_2025_8941_MOESM23_ESM.zip › Supplementary Data 8_Marker_expression_in_compacted-soils-based_Spatial_transcriptomics_Rice/Exodermis-LOC_Os03g37411.png]

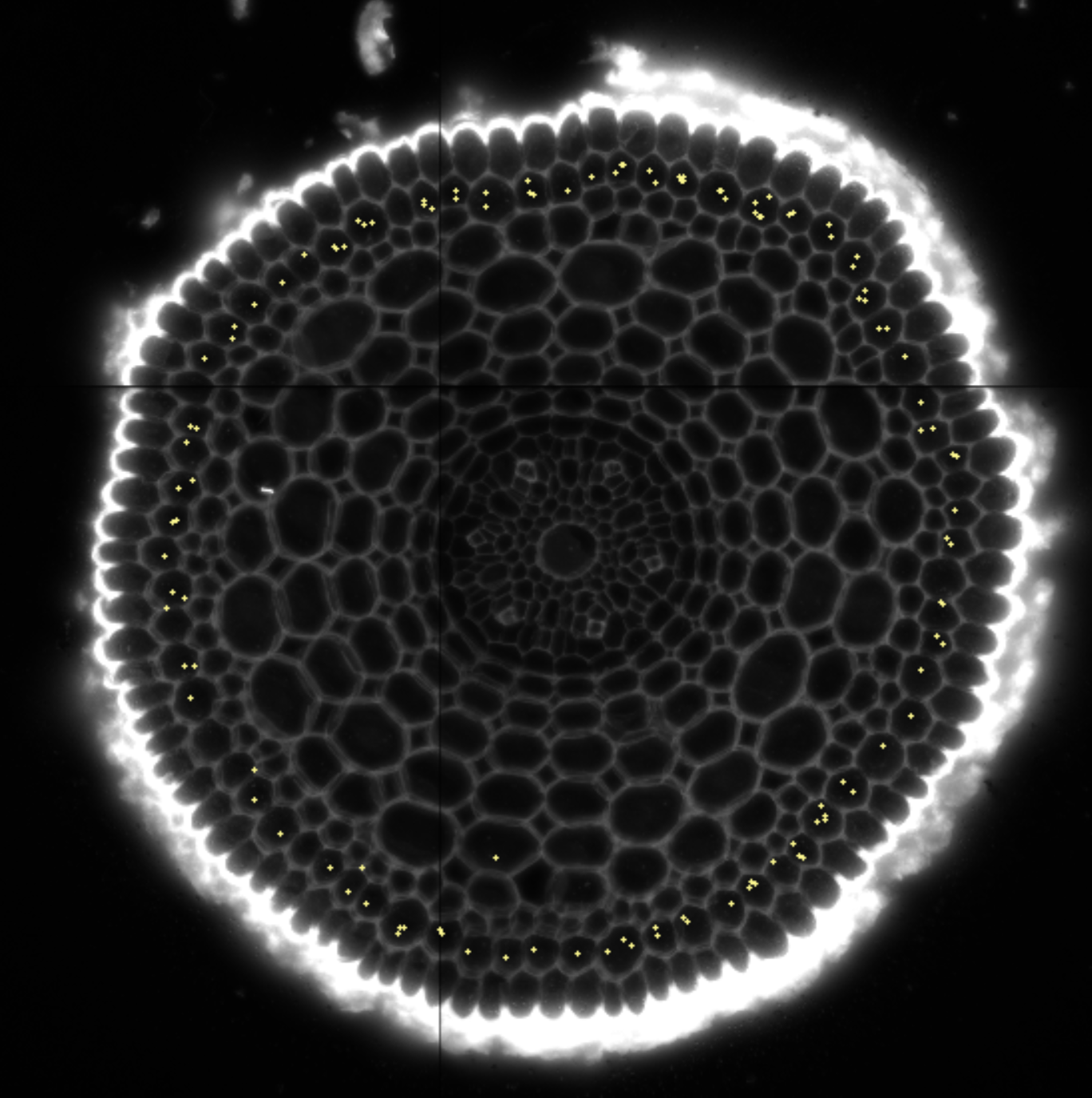

Supplement: Supplementary file 23 — Expression patterns of cell type markers in spatial transcriptomics data for compacted soil grown roots. A PDF summary file that includes the sample and gene information for visualization is included. The raw spatial transcriptomics data for compacted soil grown roots is also included. [file 41586_2025_8941_MOESM23_ESM.zip › Supplementary Data 8_Marker_expression_in_compacted-soils-based_Spatial_transcriptomics_Rice/Exodermis-LOC_Os03g37411-2.png]

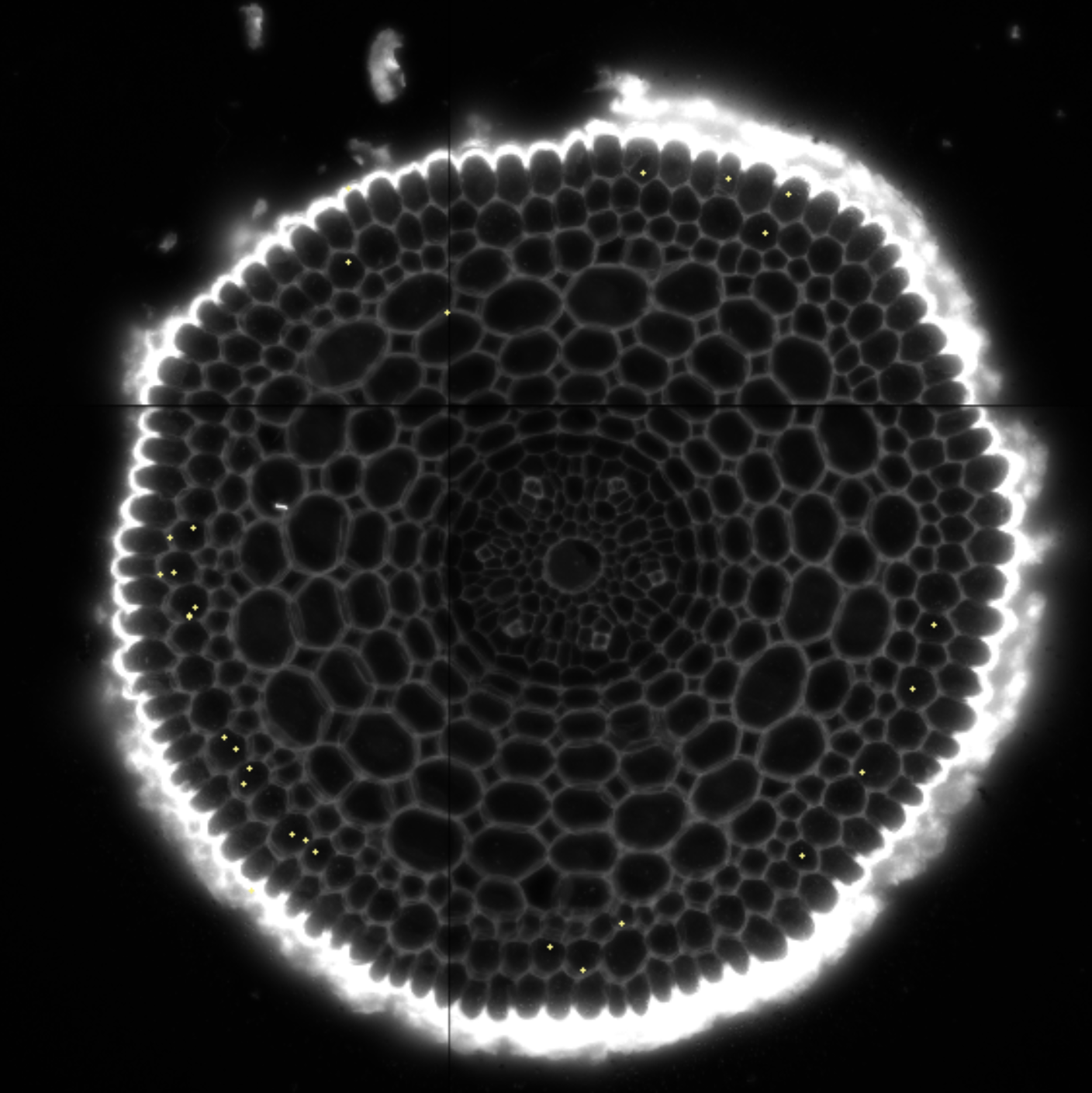

Supplement: Supplementary file 23 — Expression patterns of cell type markers in spatial transcriptomics data for compacted soil grown roots. A PDF summary file that includes the sample and gene information for visualization is included. The raw spatial transcriptomics data for compacted soil grown roots is also included. [file 41586_2025_8941_MOESM23_ESM.zip › Supplementary Data 8_Marker_expression_in_compacted-soils-based_Spatial_transcriptomics_Rice/Exodermis-LOC_Os04g37980.png]

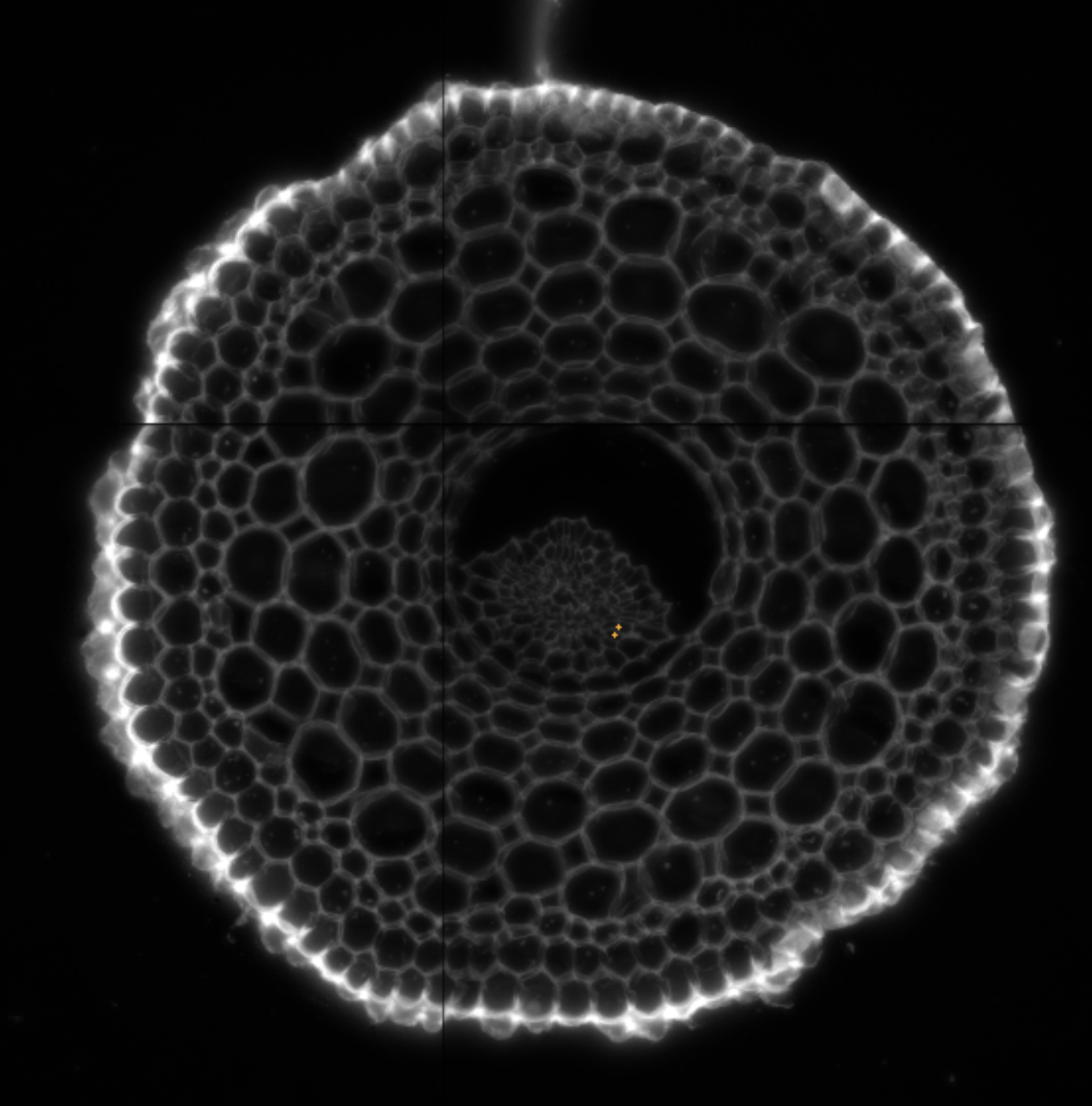

Supplement: Supplementary file 23 — Expression patterns of cell type markers in spatial transcriptomics data for compacted soil grown roots. A PDF summary file that includes the sample and gene information for visualization is included. The raw spatial transcriptomics data for compacted soil grown roots is also included. [file 41586_2025_8941_MOESM23_ESM.zip › Supplementary Data 8_Marker_expression_in_compacted-soils-based_Spatial_transcriptomics_Rice/Phloem-LOC_Os06g45410.png]

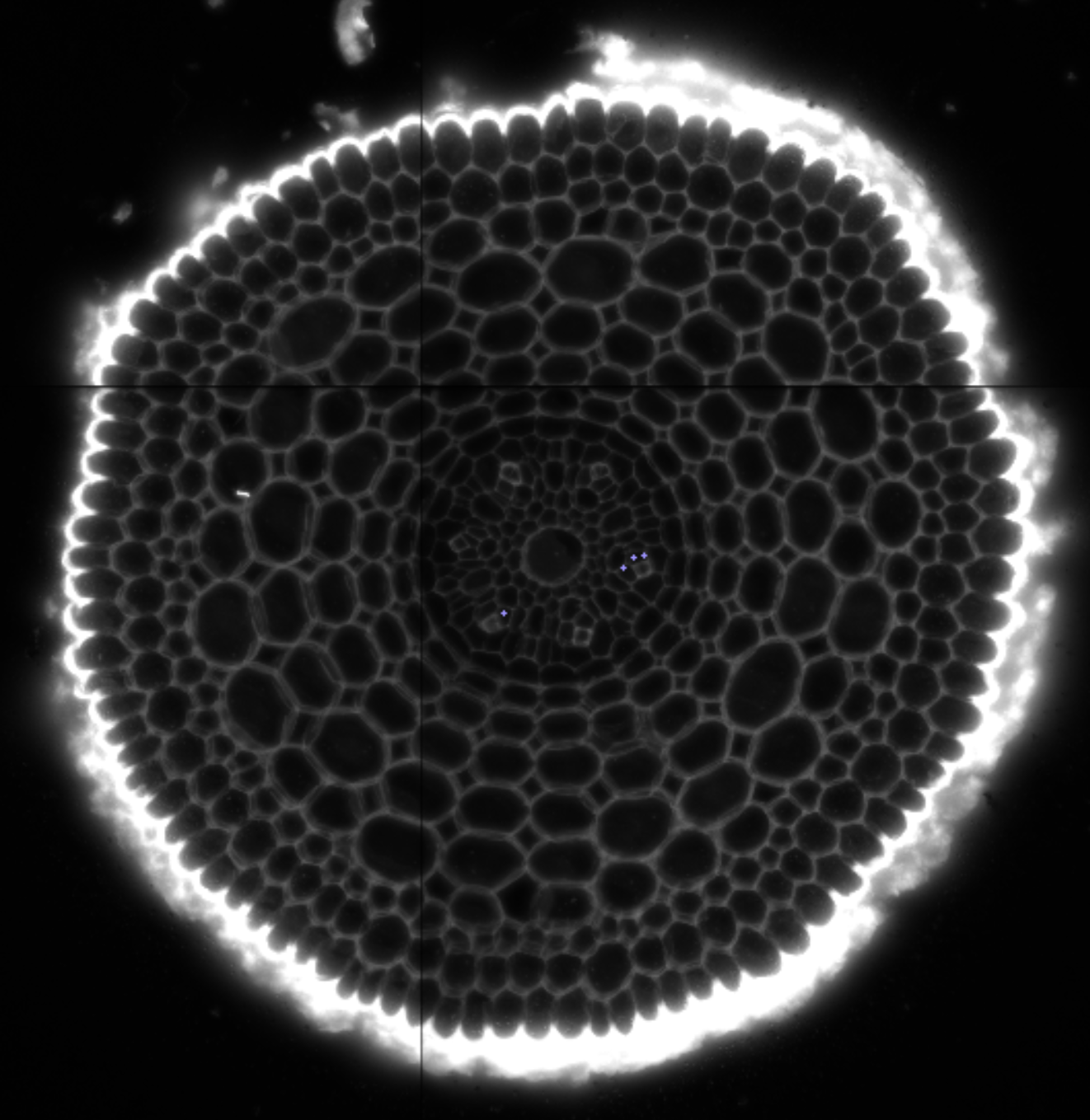

Supplement: Supplementary file 23 — Expression patterns of cell type markers in spatial transcriptomics data for compacted soil grown roots. A PDF summary file that includes the sample and gene information for visualization is included. The raw spatial transcriptomics data for compacted soil grown roots is also included. [file 41586_2025_8941_MOESM23_ESM.zip › Supplementary Data 8_Marker_expression_in_compacted-soils-based_Spatial_transcriptomics_Rice/Phloem-LOC_Os06g45410-2.png]

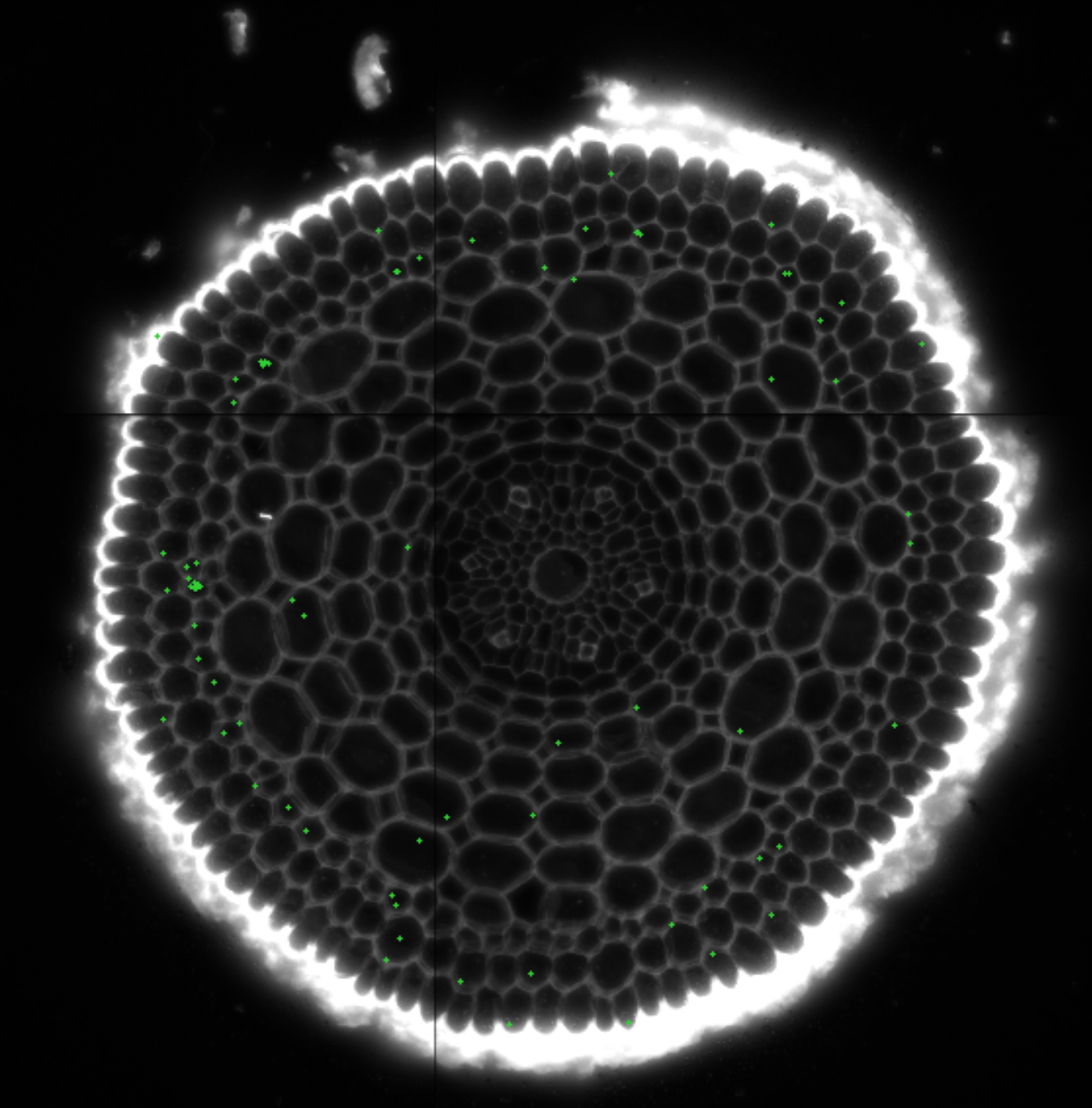

Supplement: Supplementary file 23 — Expression patterns of cell type markers in spatial transcriptomics data for compacted soil grown roots. A PDF summary file that includes the sample and gene information for visualization is included. The raw spatial transcriptomics data for compacted soil grown roots is also included. [file 41586_2025_8941_MOESM23_ESM.zip › Supplementary Data 8_Marker_expression_in_compacted-soils-based_Spatial_transcriptomics_Rice/Sclerenchyma-LOC_Os08g02300.png]

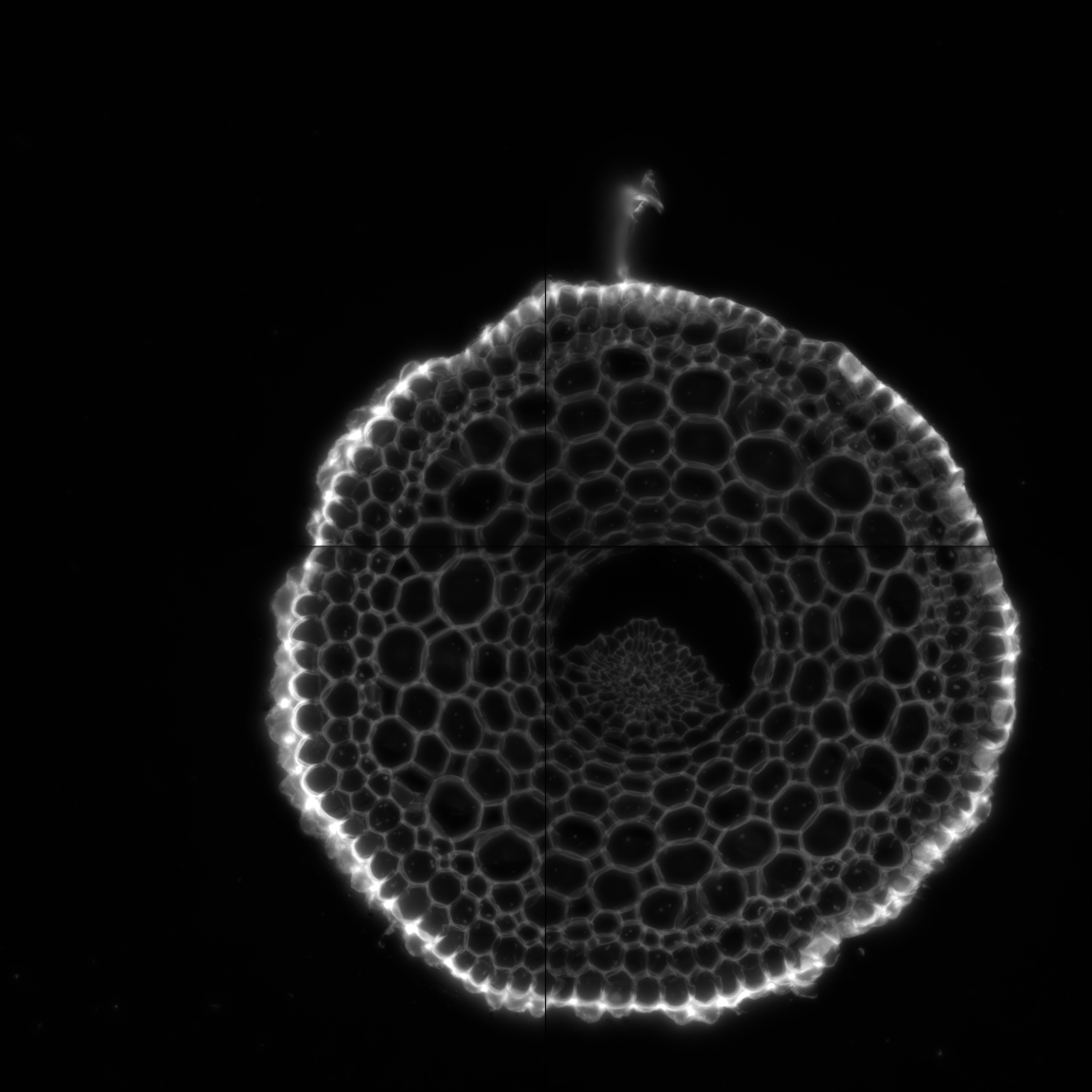

Supplement: Supplementary file 23 — Expression patterns of cell type markers in spatial transcriptomics data for compacted soil grown roots. A PDF summary file that includes the sample and gene information for visualization is included. The raw spatial transcriptomics data for compacted soil grown roots is also included. [file 41586_2025_8941_MOESM23_ESM.zip › Supplementary Data 8_Marker_expression_in_compacted-soils-based_Spatial_transcriptomics_Rice/Soil-CMP-spatial-raw data/33282-Benfey2_C1-1_Calco.tiff]

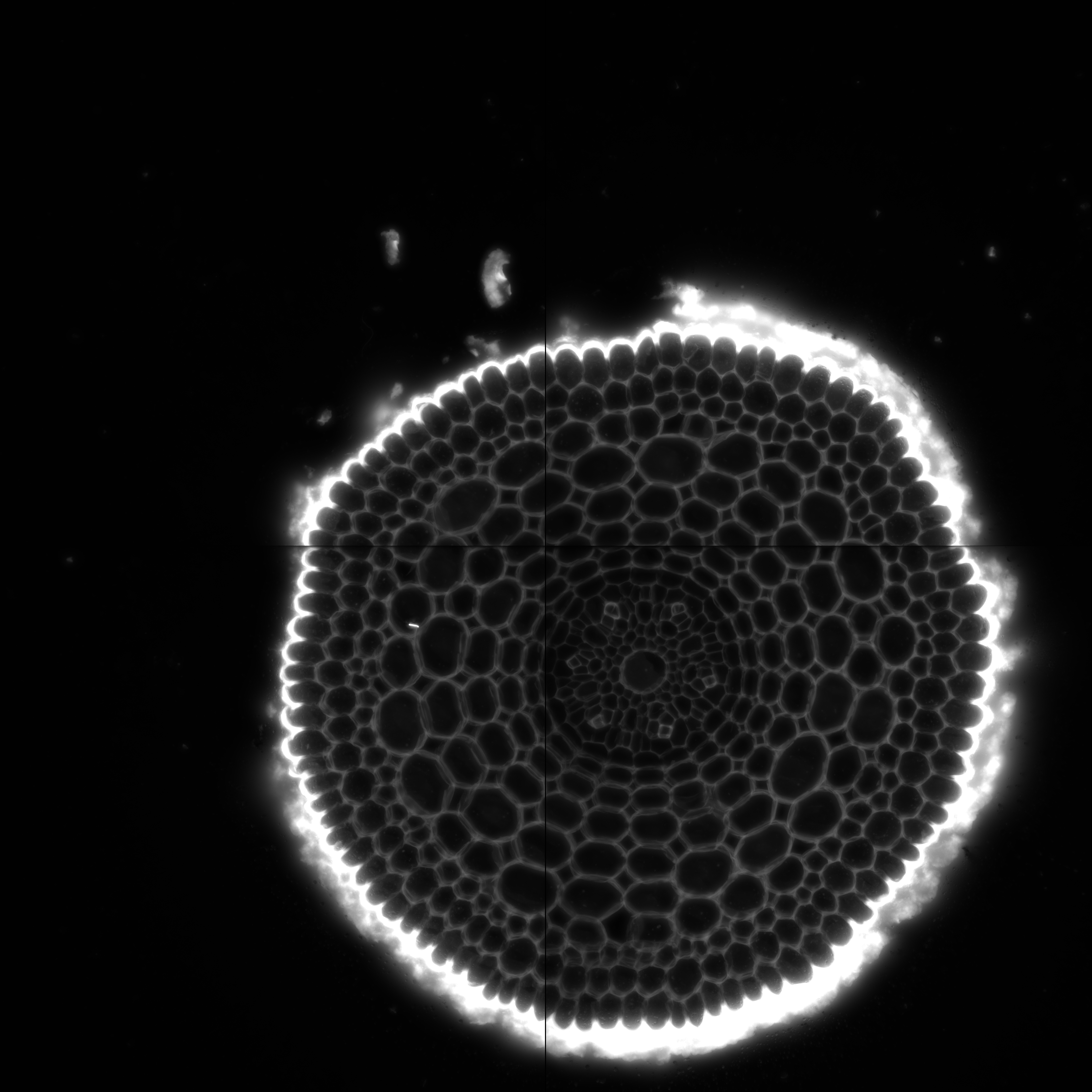

Supplement: Supplementary file 23 — Expression patterns of cell type markers in spatial transcriptomics data for compacted soil grown roots. A PDF summary file that includes the sample and gene information for visualization is included. The raw spatial transcriptomics data for compacted soil grown roots is also included. [file 41586_2025_8941_MOESM23_ESM.zip › Supplementary Data 8_Marker_expression_in_compacted-soils-based_Spatial_transcriptomics_Rice/Soil-CMP-spatial-raw data/33282-Benfey2_C1-2_Calco.tiff]

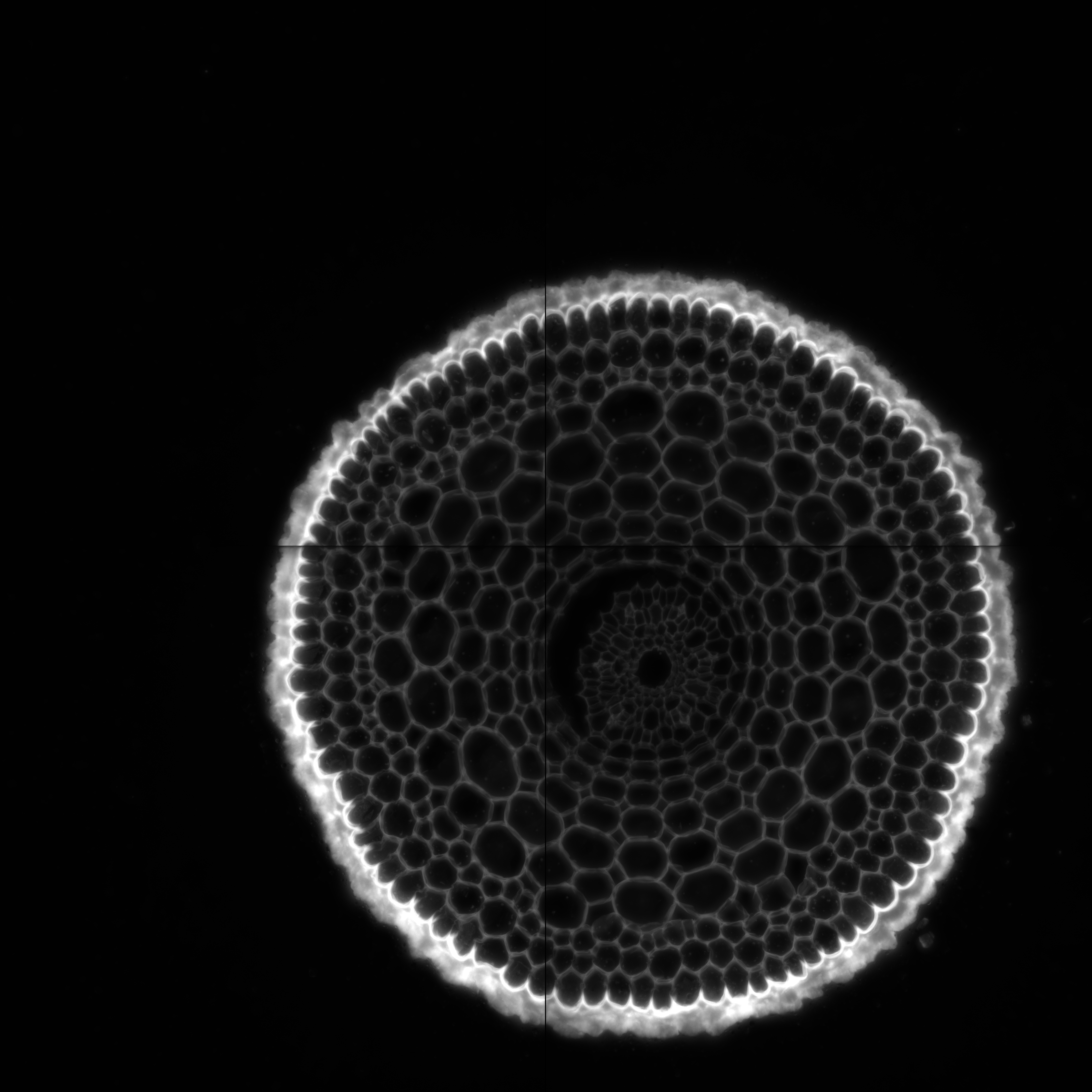

Supplement: Supplementary file 23 — Expression patterns of cell type markers in spatial transcriptomics data for compacted soil grown roots. A PDF summary file that includes the sample and gene information for visualization is included. The raw spatial transcriptomics data for compacted soil grown roots is also included. [file 41586_2025_8941_MOESM23_ESM.zip › Supplementary Data 8_Marker_expression_in_compacted-soils-based_Spatial_transcriptomics_Rice/Soil-CMP-spatial-raw data/33282-Benfey2_D1-1_Calco.tiff]

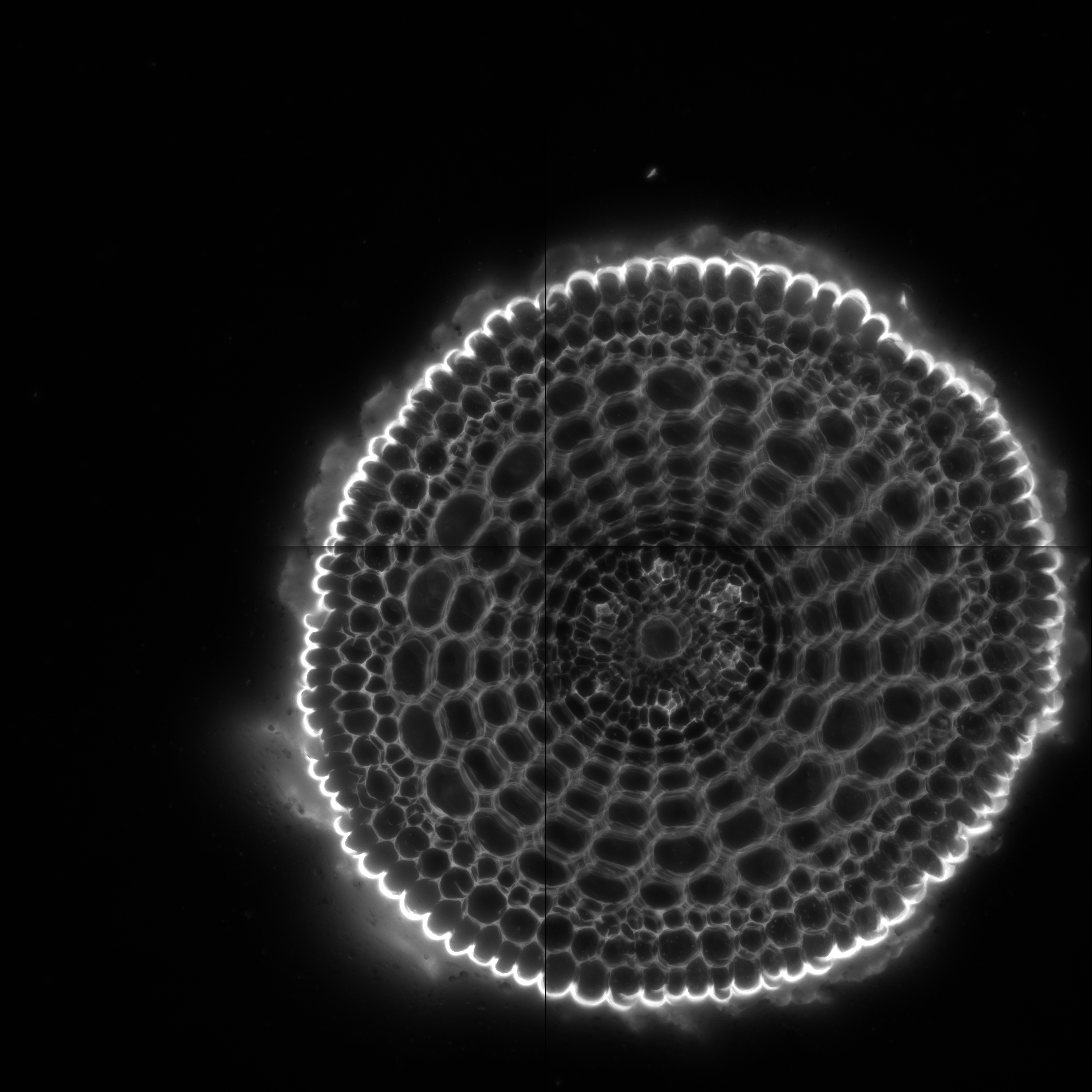

Supplement: Supplementary file 23 — Expression patterns of cell type markers in spatial transcriptomics data for compacted soil grown roots. A PDF summary file that includes the sample and gene information for visualization is included. The raw spatial transcriptomics data for compacted soil grown roots is also included. [file 41586_2025_8941_MOESM23_ESM.zip › Supplementary Data 8_Marker_expression_in_compacted-soils-based_Spatial_transcriptomics_Rice/Soil-CMP-spatial-raw data/33282-Benfey2_D1-2_Calco.tiff]

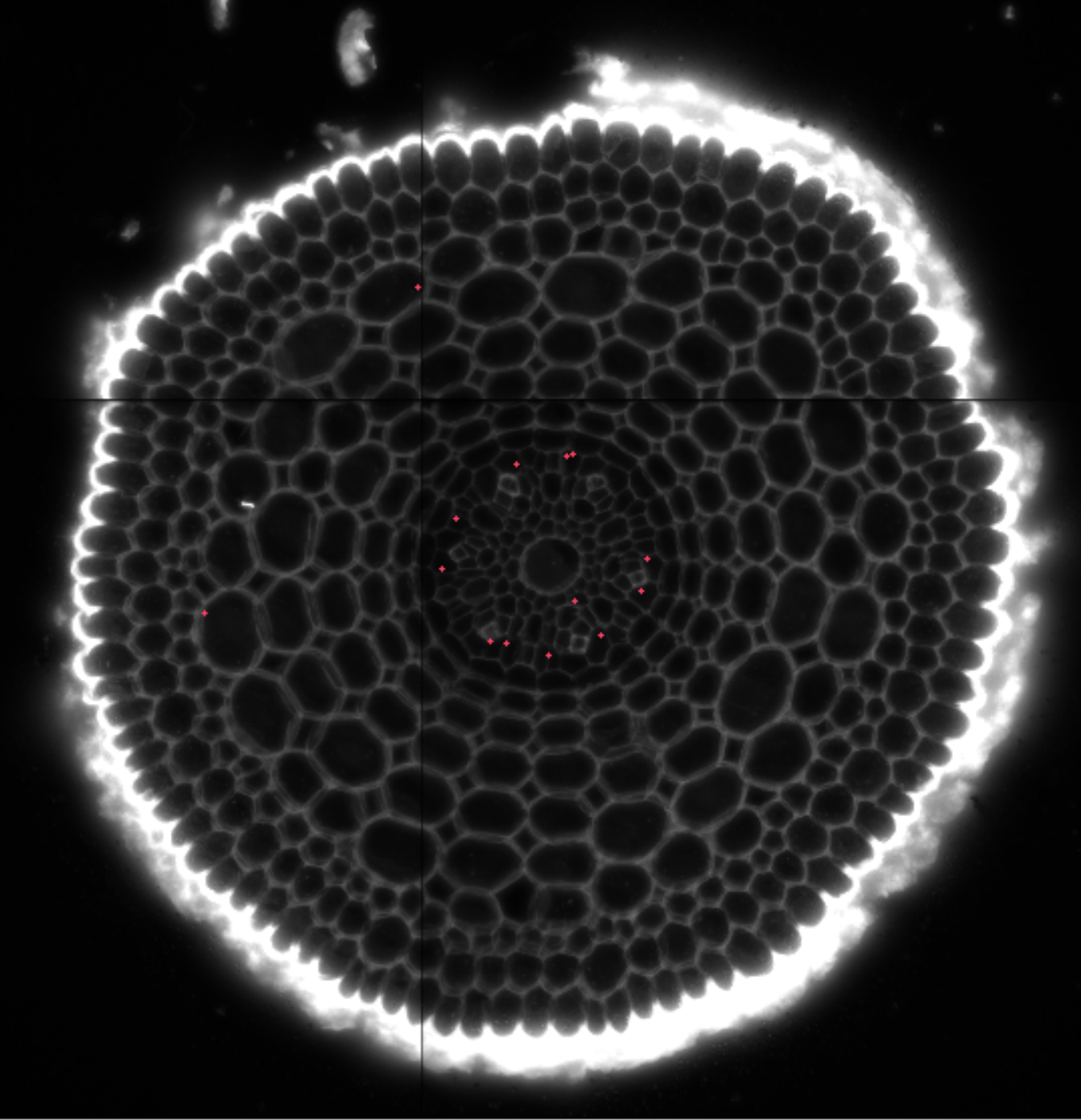

Supplement: Supplementary file 23 — Expression patterns of cell type markers in spatial transcriptomics data for compacted soil grown roots. A PDF summary file that includes the sample and gene information for visualization is included. The raw spatial transcriptomics data for compacted soil grown roots is also included. [file 41586_2025_8941_MOESM23_ESM.zip › Supplementary Data 8_Marker_expression_in_compacted-soils-based_Spatial_transcriptomics_Rice/Stele-LOC_Os10g03400.png]

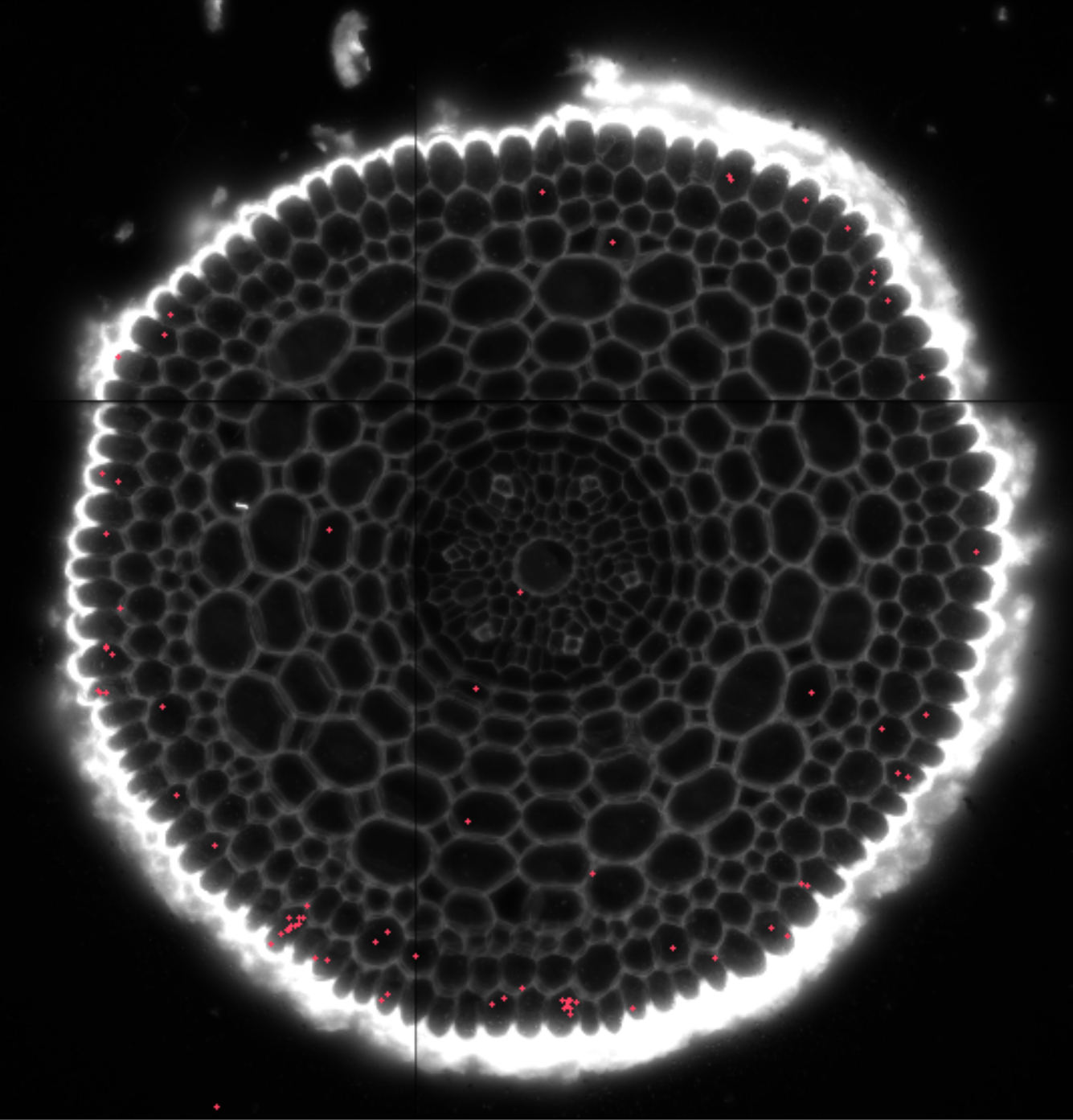

Supplement: Supplementary file 23 — Expression patterns of cell type markers in spatial transcriptomics data for compacted soil grown roots. A PDF summary file that includes the sample and gene information for visualization is included. The raw spatial transcriptomics data for compacted soil grown roots is also included. [file 41586_2025_8941_MOESM23_ESM.zip › Supplementary Data 8_Marker_expression_in_compacted-soils-based_Spatial_transcriptomics_Rice/Trichoblast-LOC_Os10g42750.png]

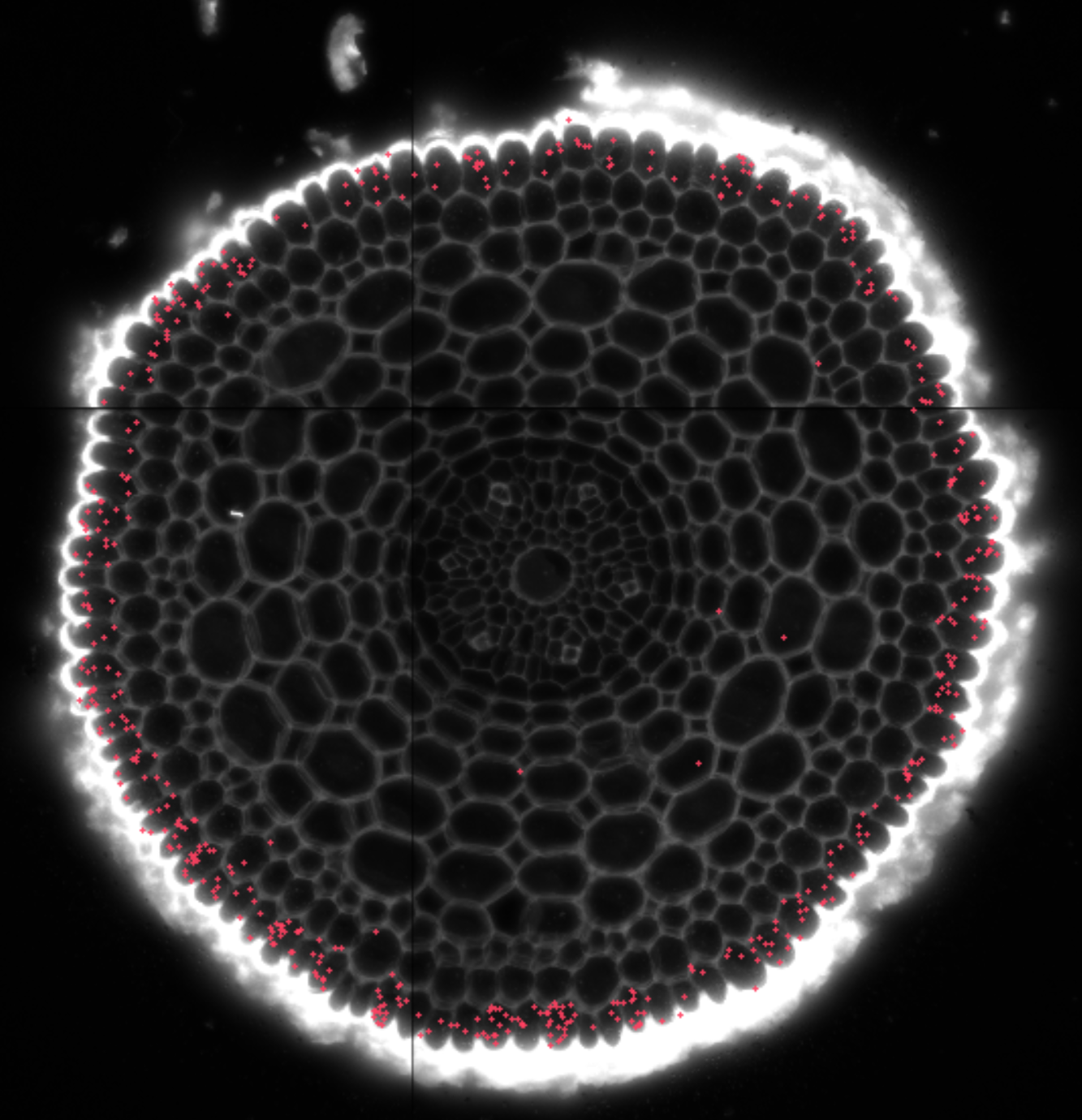

Supplement: Supplementary file 23 — Expression patterns of cell type markers in spatial transcriptomics data for compacted soil grown roots. A PDF summary file that includes the sample and gene information for visualization is included. The raw spatial transcriptomics data for compacted soil grown roots is also included. [file 41586_2025_8941_MOESM23_ESM.zip › Supplementary Data 8_Marker_expression_in_compacted-soils-based_Spatial_transcriptomics_Rice/Trichoblast-LOC_Os12g05380.png]

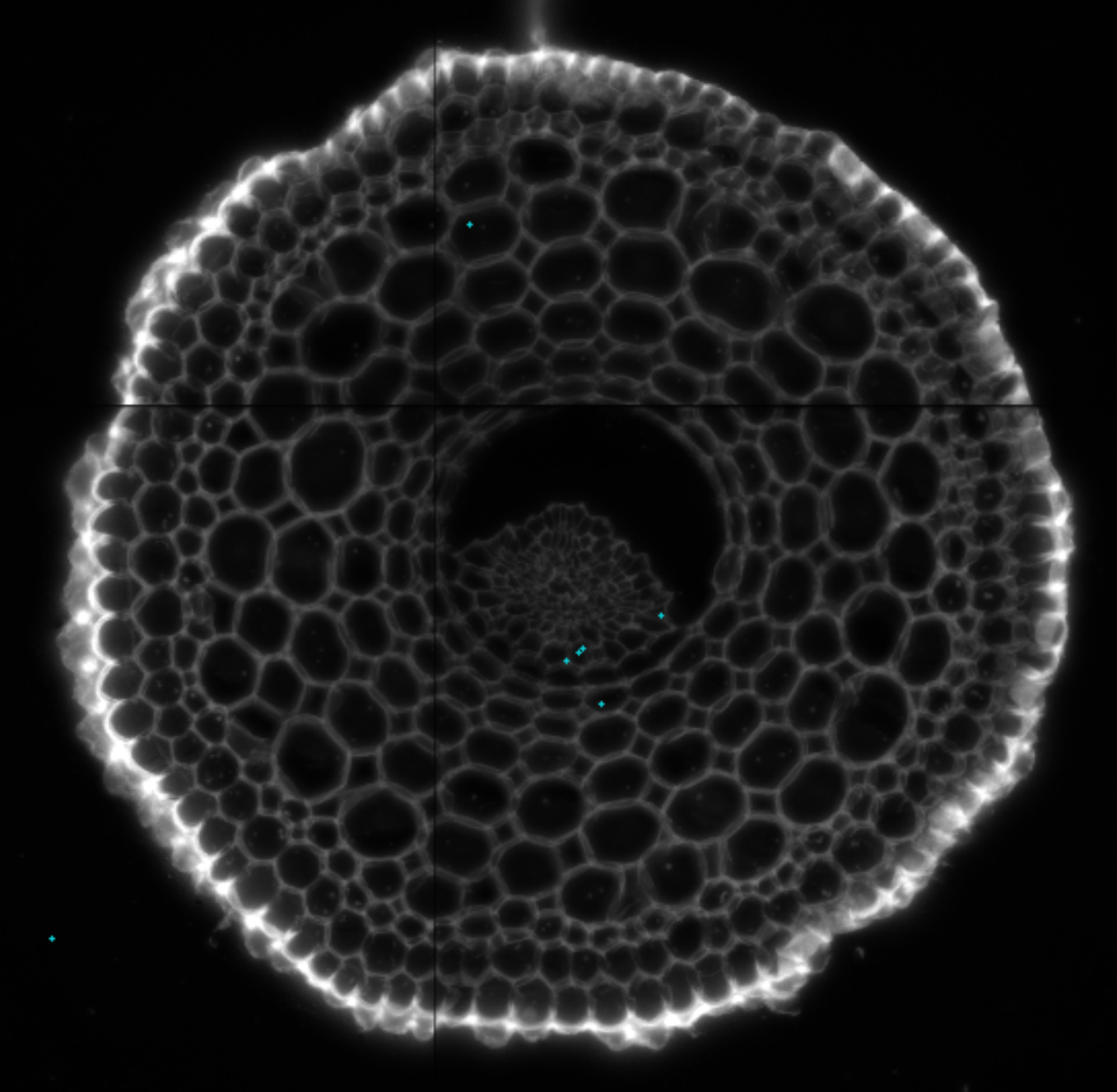

Supplement: Supplementary file 23 — Expression patterns of cell type markers in spatial transcriptomics data for compacted soil grown roots. A PDF summary file that includes the sample and gene information for visualization is included. The raw spatial transcriptomics data for compacted soil grown roots is also included. [file 41586_2025_8941_MOESM23_ESM.zip › Supplementary Data 8_Marker_expression_in_compacted-soils-based_Spatial_transcriptomics_Rice/Vascular tissue-LOC_Os01g19170.png]

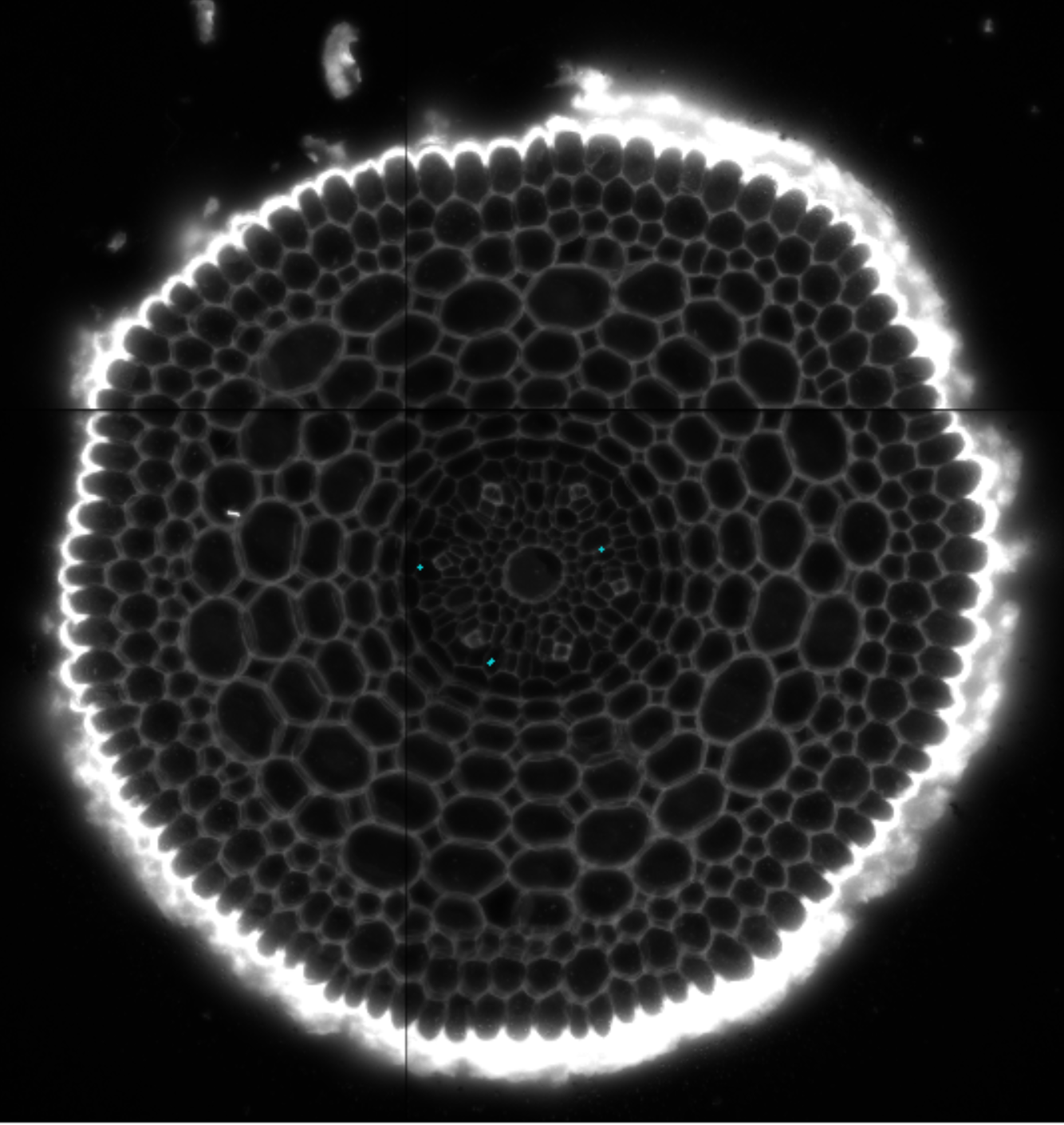

Supplement: Supplementary file 23 — Expression patterns of cell type markers in spatial transcriptomics data for compacted soil grown roots. A PDF summary file that includes the sample and gene information for visualization is included. The raw spatial transcriptomics data for compacted soil grown roots is also included. [file 41586_2025_8941_MOESM23_ESM.zip › Supplementary Data 8_Marker_expression_in_compacted-soils-based_Spatial_transcriptomics_Rice/Vascular tissue-LOC_Os01g19170-2.png]
