# Supplementary material for: Single-cell transcriptomics reveal how root tissues adapt to soil stress
Source: Nature. 2025 Apr 30;642(8068):721–9. doi: 10.1038/s41586-025-08941-z (PMC12176638; doi:10.1038/s41586-025-08941-z)
Supplement: Supplementary file 21 — Expression patterns of cell type markers in spatial transcriptomics data for non-compacted soil grown roots. A PDF summary file that includes the sample and gene information for visualization is included. The raw spatial transcriptomics data for non-compacted soil grown roots is also included. [file 41586_2025_8941_MOESM21_ESM.zip › Supplementary Data 6_Marker_expression_in_non-compacted-soils-based_Spatial_transcriptomics_Rice/Summary of non-compacted soil spatial.pdf]

Combined

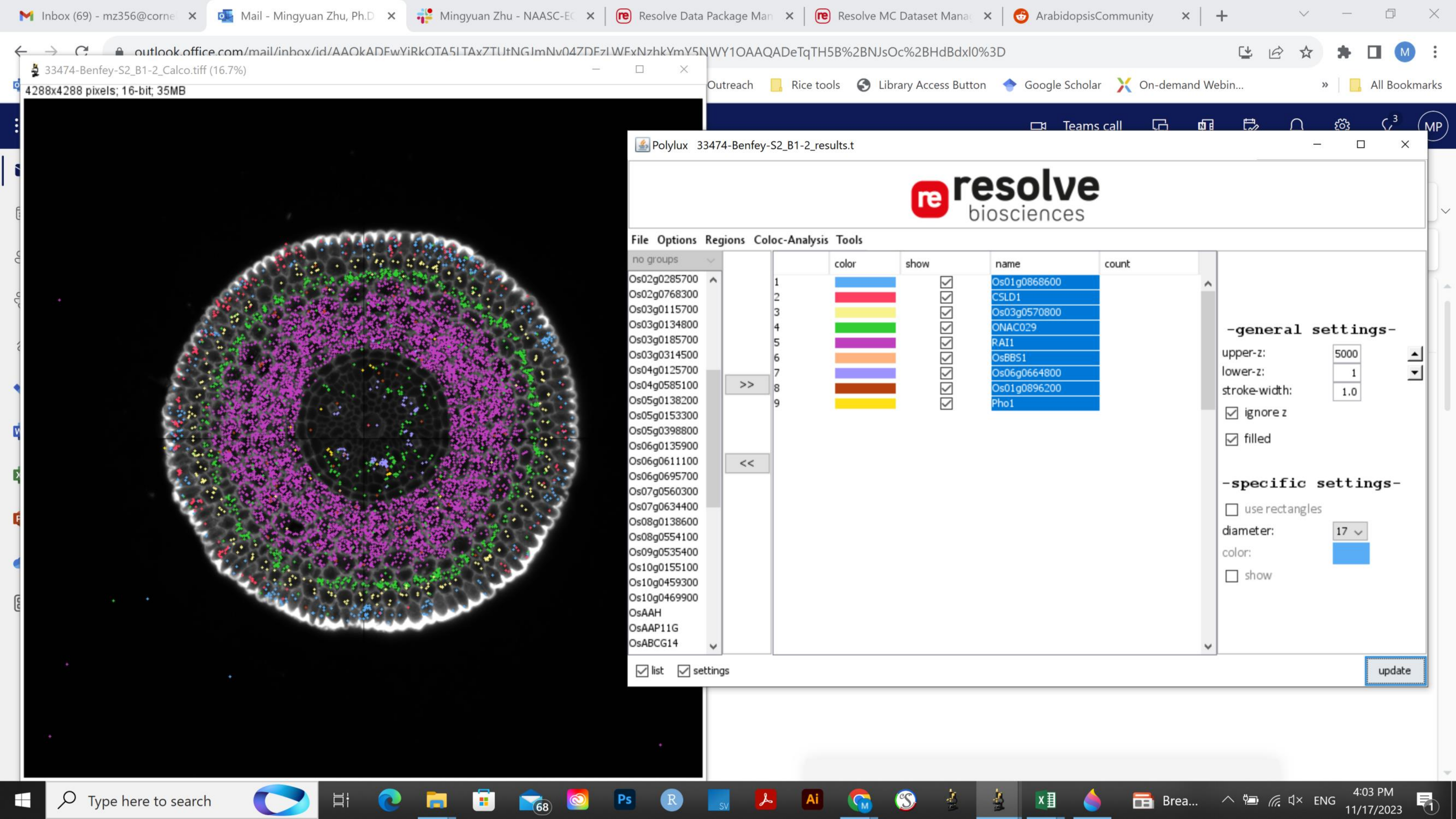

Inbox (69) - mz356@corne
Mail - Mingyuan Zhu, Ph.D
Mingyuan Zhu - NAASC-EC
Resolve Data Package Man
Resolve MC Dataset Mana
ArabidopsisCommunity
outlook.office.com/mail/inbox/id/AAQkADEwYjRkOTA5LTAxZTUtNGJmNy04ZDEzLWExNzhkYmY5NWY1OAAQADeTqTH5B%2BNJsOc%2BHdBdxl0%3D
Outreach
Rice tools
Library Access Button
Google Scholar
On-demand Webin...
All Bookmarks
Teams call
33474-Benfey-S2_B1-2_Calco.tiff (16.7%)
4288x4288 pixels; 16-bit; 35MB
Polylux 33474-Benfey-S2_B1-2_results.t
resolve
biosciences
File
Options
Regions
Coloc-Analysis
Tools
no groups
Os02g0285700
Os02g0768300
Os03g0115700
Os03g0134800
Os03g0185700
Os03g0314500
Os04g0125700
Os04g0585100
Os05g0138200
Os05g0153300
Os05g0398800
Os06g0135900
Os06g0611100
Os06g0695700
Os07g0560300
Os07g0634400
Os08g0138600
Os08g0554100
Os09g0535400
Os10g0155100
Os10g0459300
Os10g0469900
OsAAH
OsAAP11G
OsABCG14
>>
<<
color
show
name
count
1 Os01g0868600
2 CSLD1
3 Os03g0570800
4 ONAC029
5 RAI1
6 OsBBS1
7 Os06g0664800
8 Os01g0896200
9 Pho1
-general settings-
upper-z: 5000
lower-z: 1
stroke-width: 1.0
ignore z
filled
-specific settings-
use rectangles
diameter: 17
color:
show
list
settings
update
Type here to search
Brea...
ENG
4:03 PM
11/17/2023

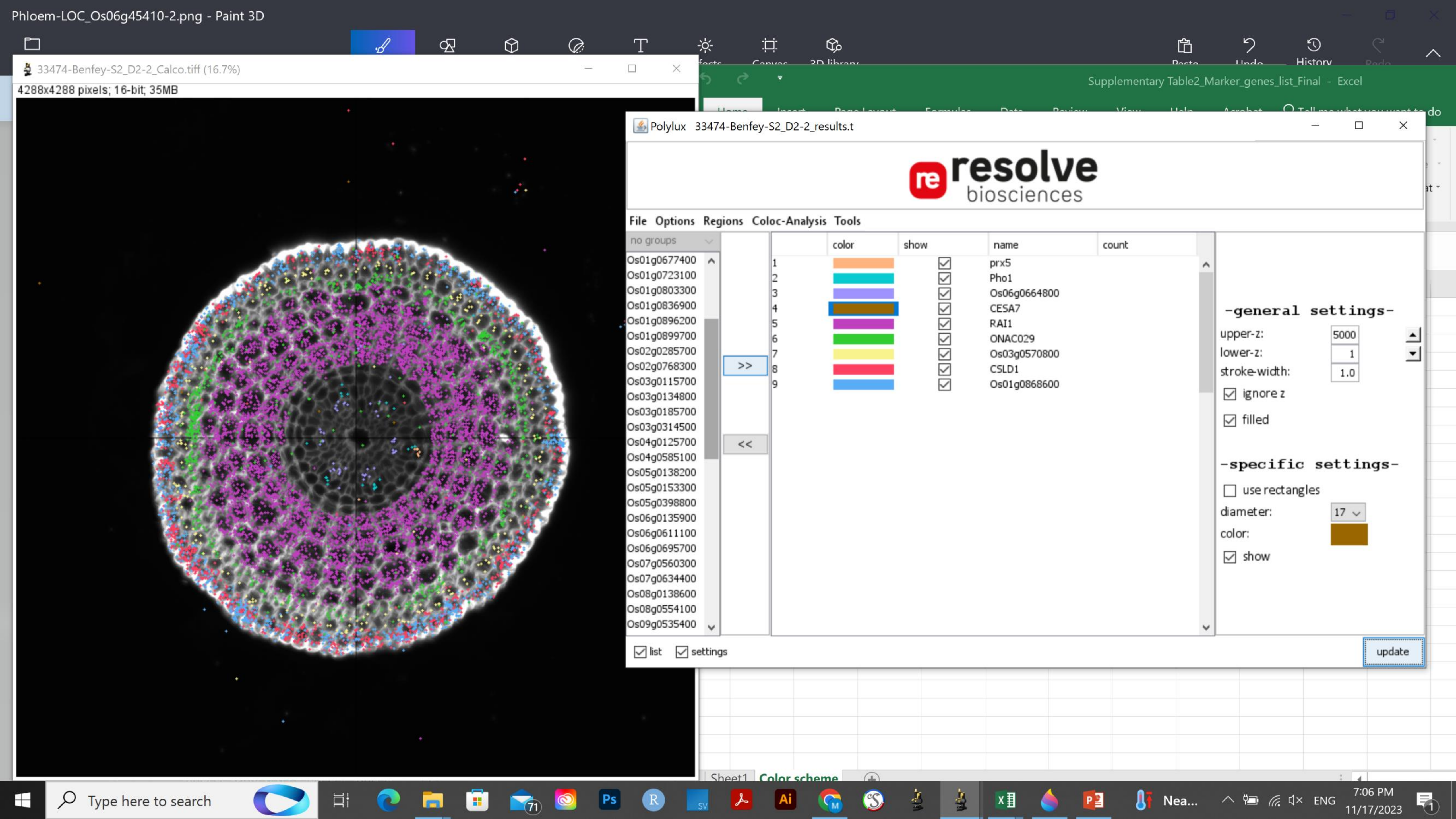

Phloem-LOC_Os06g45410-2.png - Paint 3D
33474-Benfey-S2_D2-2_Calco.tiff (16.7%)
4288x4288 pixels; 16-bit; 35MB
Supplementary Table2_Marker_genes_list_Final - Excel
Polylux 33474-Benfey-S2_D2-2_results.t
resolve biosciences
File Options Regions Coloc-Analysis Tools
no groups
Os01g0677400
Os01g0723100
Os01g0803300
Os01g0836900
Os01g0896200
Os01g0899700
Os02g0285700
Os02g0768300
Os03g0115700
Os03g0134800
Os03g0185700
Os03g0314500
Os04g0125700
Os04g0585100
Os05g0138200
Os05g0153300
Os05g0398800
Os06g0135900
Os06g0611100
Os06g0695700
Os07g0560300
Os07g0634400
Os08g0138600
Os08g0554100
Os09g0535400
>>
<<
color show name count
1 prx5
2 Pho1
3 Os06g0664800
4 CESA7
5 RAI1
6 ONAC029
7 Os03g0570800
8 CSLD1
9 Os01g0868600
-general settings-
upper-z: 5000
lower-z: 1
stroke-width: 1.0
ignore z
filled
-specific settings-
use rectangles
diameter: 17
color:
show
list
settings
update
Sheet1 Color scheme
Type here to search
ENG
7:06 PM
11/17/2023

Atrichoblast

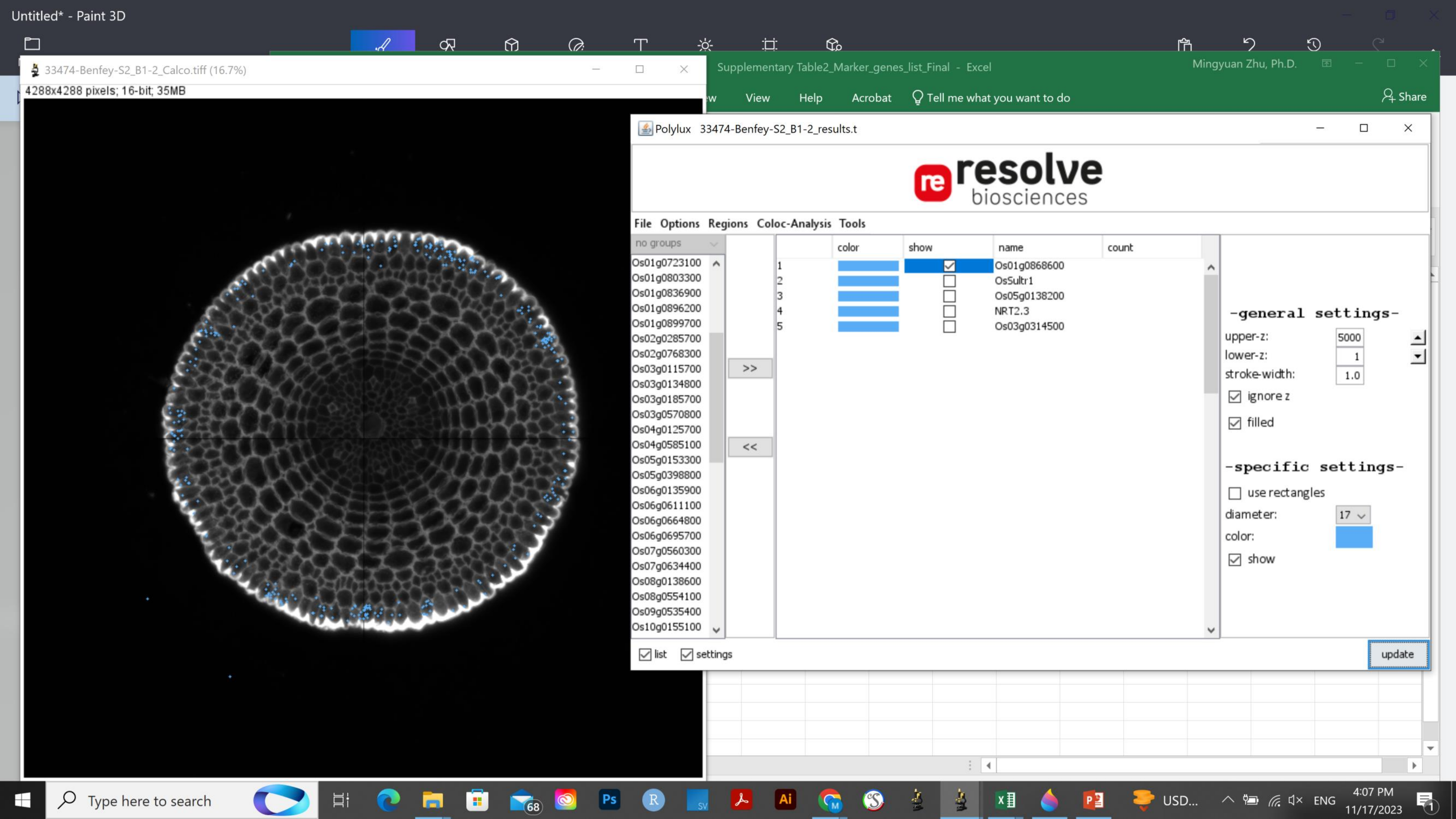

Untitled* - Paint 3D
33474-Benfey-S2_B1-2_Calco.tiff (16.7%)
4288x4288 pixels; 16-bit; 35MB
Supplementary Table2_Marker_genes_list_Final - Excel
Mingyuan Zhu, Ph.D.
View
Help
Acrobat
Tell me what you want to do
Share
Polylux 33474-Benfey-S2_B1-2_results.t
resolve biosciences
File Options Regions Coloc-Analysis Tools
no groups
Os01g0723100
Os01g0803300
Os01g0836900
Os01g0896200
Os01g0899700
Os02g0285700
Os02g0768300
Os03g0115700
Os03g0134800
Os03g0185700
Os03g0570800
Os04g0125700
Os04g0585100
Os05g0153300
Os05g0398800
Os06g0135900
Os06g0611100
Os06g0664800
Os06g0695700
Os07g0560300
Os07g0634400
Os08g0138600
Os08g0554100
Os09g0535400
Os10g0155100
>>
<<
color
show
name
count
1 Os01g0868600
2 OsSultr1
3 Os05g0138200
4 NRT2.3
5 Os03g0314500
-general settings-
upper-z: 5000
lower-z: 1
stroke-width: 1.0
ignore z
filled
-specific settings-
use rectangles
diameter: 17
color:
show
list
settings
update
Type here to search
68
USD...
ENG
4:07 PM
11/17/2023

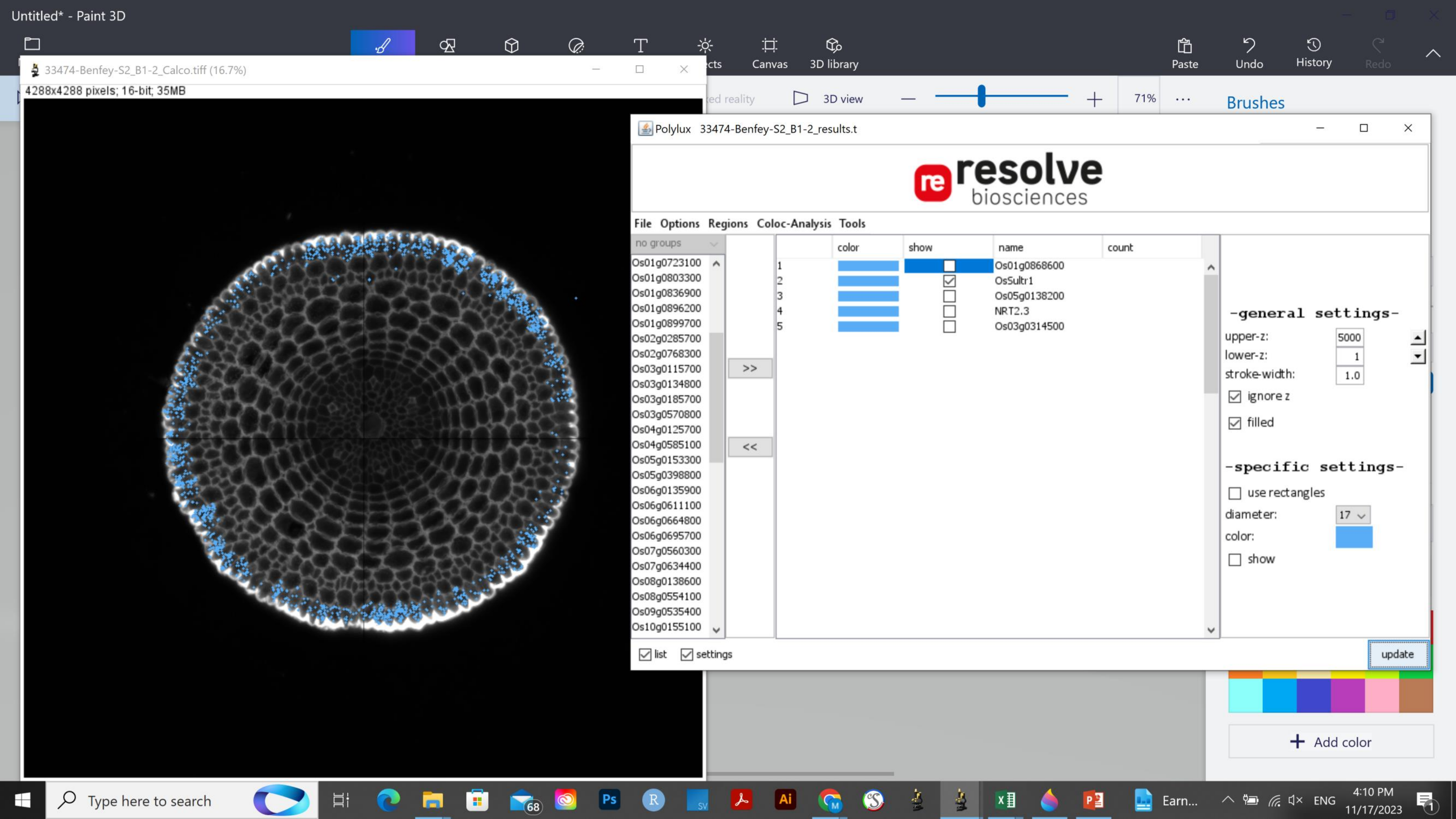
Untitled* - Paint 3D
Canvas
3D library
Paste
Undo
History
Redo
3D view
71%
Brushes
33474-Benfey-S2_B1-2_Calco.tiff (16.7%)
4288x4288 pixels; 16-bit; 35MB
Polylux 33474-Benfey-S2_B1-2_results.t
resolve biosciences
File Options Regions Coloc-Analysis Tools
no groups
Os01g0723100
Os01g0803300
Os01g0836900
Os01g0896200
Os01g0899700
Os02g0285700
Os02g0768300
Os03g0115700
Os03g0134800
Os03g0185700
Os03g0570800
Os04g0125700
Os04g0585100
Os05g0153300
Os05g0398800
Os06g0135900
Os06g0611100
Os06g0664800
Os06g0695700
Os07g0560300
Os07g0634400
Os08g0138600
Os08g0554100
Os09g0535400
Os10g0155100
>>
<<
color show name count
1 Os01g0868600
2 OsSultr1
3 Os05g0138200
4 NRT2.3
5 Os03g0314500
-general settings-
upper-z: 5000
lower-z: 1
stroke-width: 1.0
ignore z
filled
-specific settings-
use rectangles
diameter: 17
color:
show
list
settings
update
Add color
Type here to search
ENG
4:10 PM
11/17/2023

Untitled* - Paint 3D

Canvas | 3D library | Paste | Undo | History | Redo

3D view | 71% | Brushes

33474-Benfey-S2_B1-2_Calco.tiff (16.7%)

4288x4288 pixels; 16-bit; 35MB

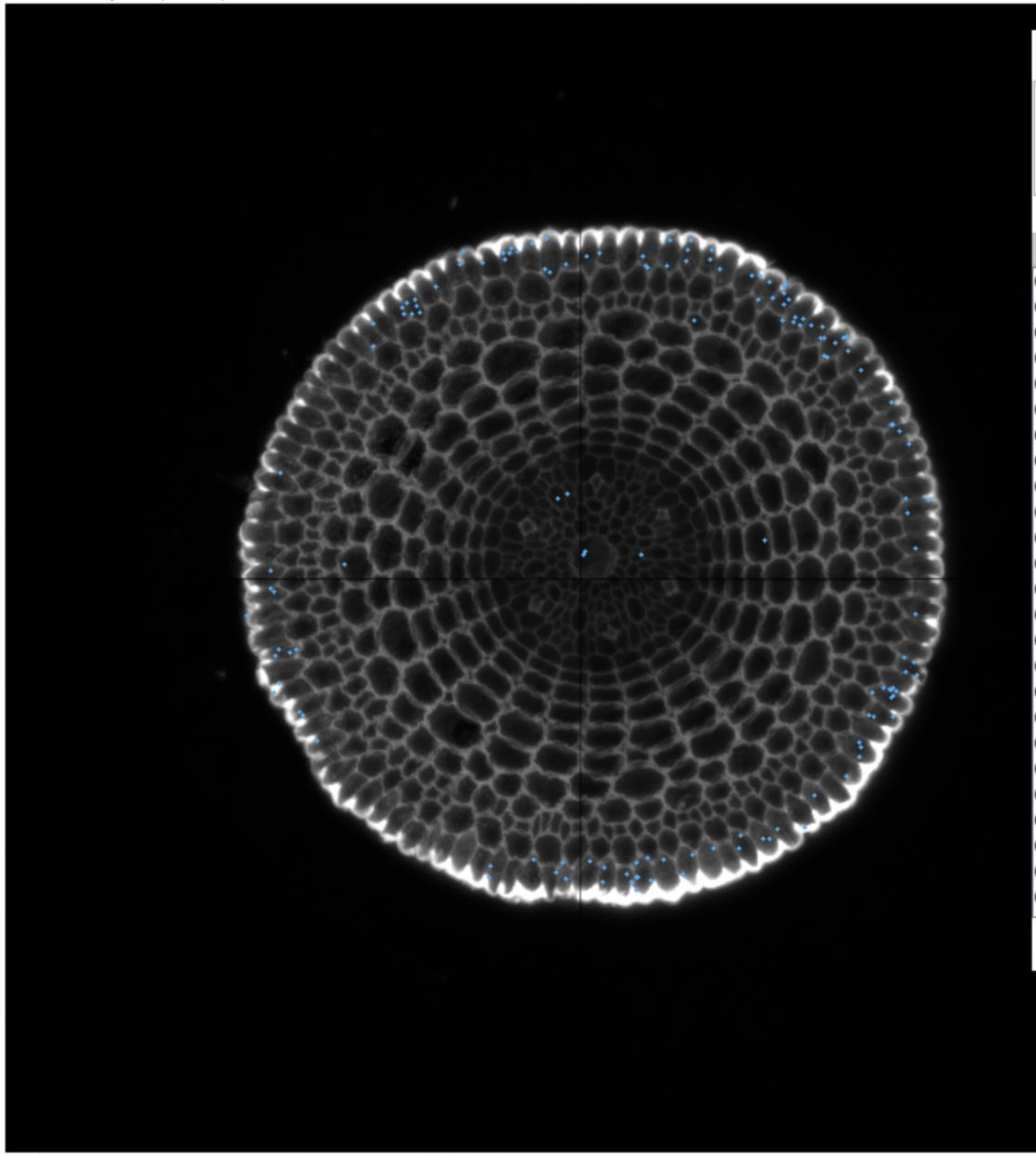

Polylux 33474-Benfey-S2_B1-2_results.t

resolve biosciences

File Options Regions Coloc-Analysis Tools

no groups

Os01g0723100
Os01g0803300
Os01g0836900
Os01g0896200
Os01g0899700
Os02g0285700
Os02g0768300
Os03g0115700
Os03g0134800
Os03g0185700
Os03g0570800
Os04g0125700
Os04g0585100
Os05g0153300
Os05g0398800
Os06g0135900
Os06g0611100
Os06g0664800
Os06g0695700
Os07g0560300
Os07g0634400
Os08g0138600
Os08g0554100
Os09g0535400
Os10g0155100

>>

<<

	color	show	name	count
1		☐	Os01g0868600	
2		☐	OsSultr1	
3		☐	Os05g0138200	
4		☑	NRT2.3	
5		☐	Os03g0314500	

-general settings-

upper-z: 5000

lower-z: 1

stroke-width: 1.0

☑ ignore z

☑ filled

-specific settings-

☐ use rectangles

diameter: 17

color:

☑ show

☑ list ☑ settings

update

Add color

Type here to search

70°F ENG 4:11 PM 11/17/2023

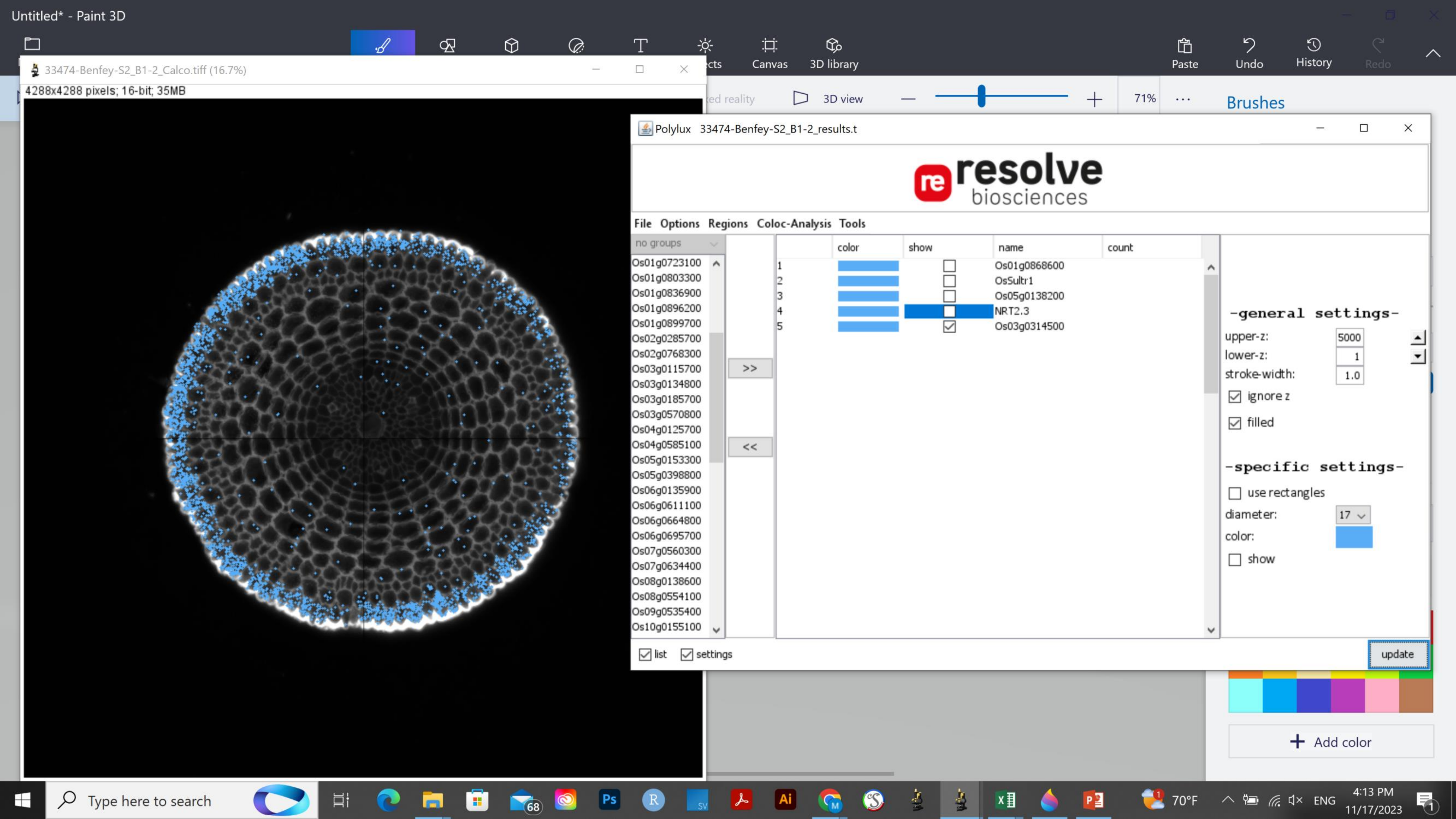

Untitled* - Paint 3D
Canvas
3D library
Paste
Undo
History
Redo
3D view
71%
Brushes
33474-Benfey-S2_B1-2_Calco.tiff (16.7%)
4288x4288 pixels; 16-bit; 35MB
Polylux 33474-Benfey-S2_B1-2_results.t
resolve biosciences
File Options Regions Coloc-Analysis Tools
no groups
Os01g0723100
Os01g0803300
Os01g0836900
Os01g0896200
Os01g0899700
Os02g0285700
Os02g0768300
Os03g0115700
Os03g0134800
Os03g0185700
Os03g0570800
Os04g0125700
Os04g0585100
Os05g0153300
Os05g0398800
Os06g0135900
Os06g0611100
Os06g0664800
Os06g0695700
Os07g0560300
Os07g0634400
Os08g0138600
Os08g0554100
Os09g0535400
Os10g0155100
>>
<<
color show name count
1 Os01g0868600
2 OsSultr1
3 Os05g0138200
4 NRT2.3
5 Os03g0314500
-general settings-
upper-z: 5000
lower-z: 1
stroke-width: 1.0
ignore z
filled
-specific settings-
use rectangles
diameter: 17
color:
show
list
settings
update
Add color
Type here to search
70°F
ENG
4:13 PM
11/17/2023

Trichoblast

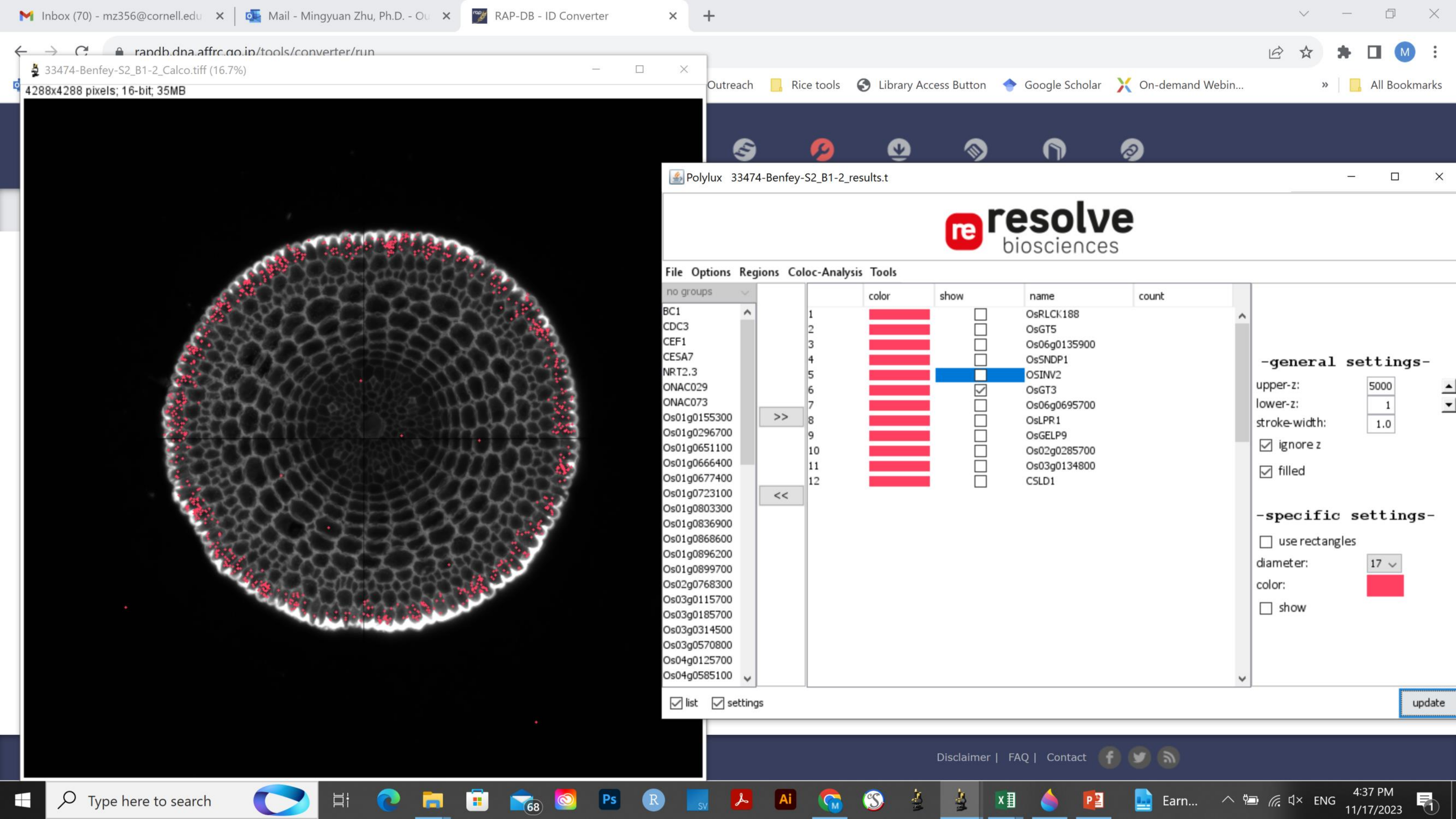

Inbox (70) - mz356@cornell.edu
Mail - Mingyuan Zhu, Ph.D. - Ou
RAP-DB - ID Converter
rapdb.dna.affrc.go.jp/tools/converter/run
Outreach
Rice tools
Library Access Button
Google Scholar
On-demand Webin...
All Bookmarks
33474-Benfey-S2_B1-2_Calco.tiff (16.7%)
4288x4288 pixels; 16-bit; 35MB
Polylux 33474-Benfey-S2_B1-2_results.t
resolve biosciences
File Options Regions Coloc-Analysis Tools
no groups
BC1
CDC3
CEF1
CESA7
NRT2.3
ONAC029
ONAC073
Os01g0155300
Os01g0296700
Os01g0651100
Os01g0666400
Os01g0677400
Os01g0723100
Os01g0803300
Os01g0836900
Os01g0868600
Os01g0896200
Os01g0899700
Os02g0768300
Os03g0115700
Os03g0185700
Os03g0314500
Os03g0570800
Os04g0125700
Os04g0585100
>>
<<
color show name count
1 OsRLCK188
2 OsGT5
3 Os06g0135900
4 OsSNDP1
5 OSINV2
6 OsGT3
7 Os06g0695700
8 OsLPR1
9 OsGELP9
10 Os02g0285700
11 Os03g0134800
12 CSLD1
-general settings-
upper-z: 5000
lower-z: 1
stroke-width: 1.0
ignore z
filled
-specific settings-
use rectangles
diameter: 17
color:
show
list
settings
update
Disclaimer | FAQ | Contact
Type here to search
Earn...
ENG
4:37 PM
11/17/2023

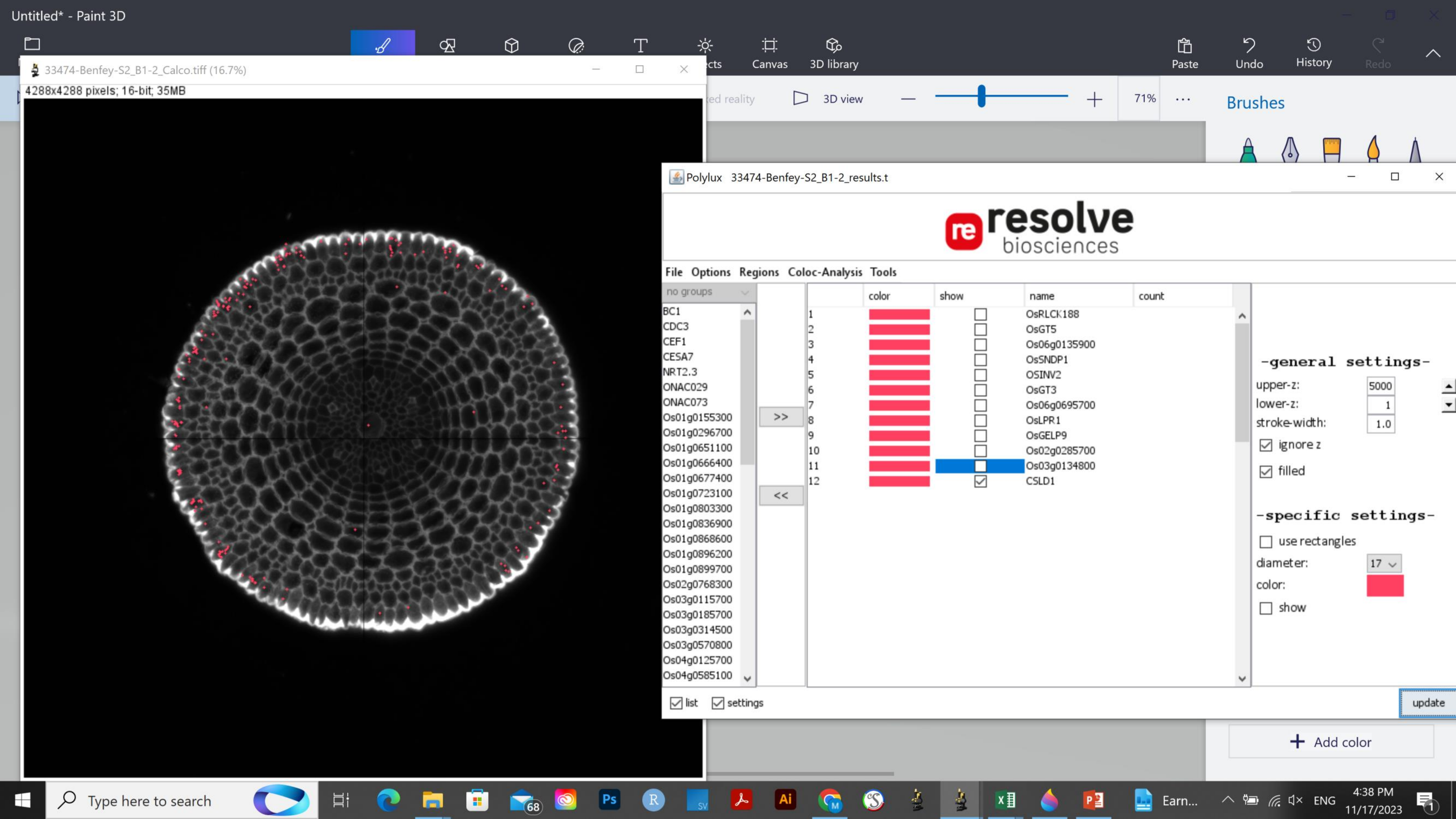
Untitled* - Paint 3D
Canvas
3D library
Paste
Undo
History
Redo
3D view
71%
Brushes
33474-Benfey-S2_B1-2_Calco.tiff (16.7%)
4288x4288 pixels; 16-bit; 35MB
Polylux 33474-Benfey-S2_B1-2_results.t
resolve biosciences
File Options Regions Coloc-Analysis Tools
no groups
BC1
CDC3
CEF1
CESA7
NRT2.3
ONAC029
ONAC073
Os01g0155300
Os01g0296700
Os01g0651100
Os01g0666400
Os01g0677400
Os01g0723100
Os01g0803300
Os01g0836900
Os01g0868600
Os01g0896200
Os01g0899700
Os02g0768300
Os03g0115700
Os03g0185700
Os03g0314500
Os03g0570800
Os04g0125700
Os04g0585100
>>
<<
color
show
name
count
1 OsRLCK188
2 OsGT5
3 Os06g0135900
4 OsSNDP1
5 OSINV2
6 OsGT3
7 Os06g0695700
8 OsLPR1
9 OsGELP9
10 Os02g0285700
11 Os03g0134800
12 CSLD1
-general settings-
upper-z: 5000
lower-z: 1
stroke-width: 1.0
ignore z
filled
-specific settings-
use rectangles
diameter: 17
color:
show
list
settings
update
Add color
Type here to search
68
ENG
4:38 PM
11/17/2023

Exodermis

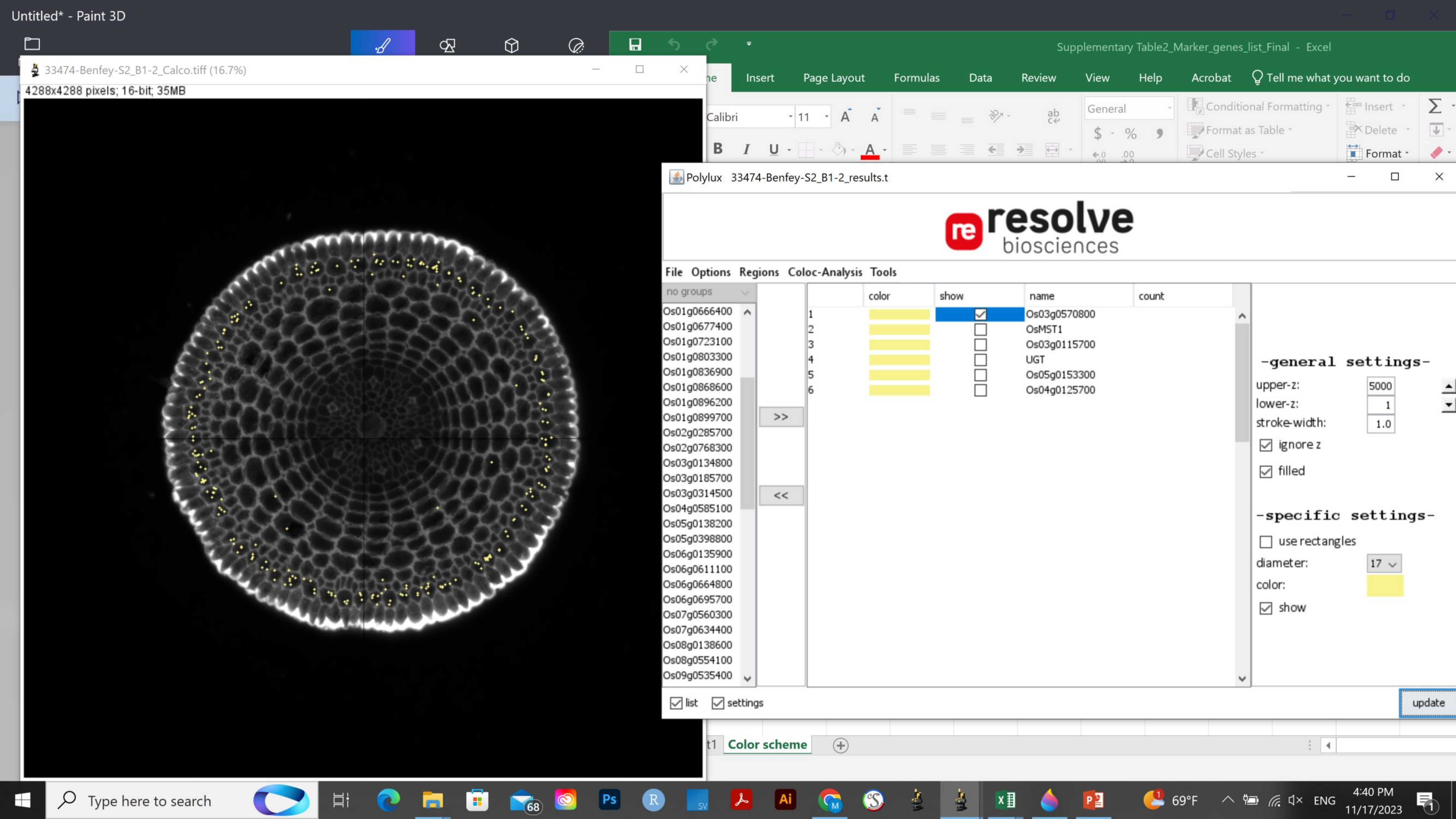
Untitled* - Paint 3D
33474-Benfey-S2_B1-2_Calco.tiff (16.7%)
4288x4288 pixels; 16-bit; 35MB
Supplementary Table2_Marker_genes_list_Final - Excel
Insert
Page Layout
Formulas
Data
Review
View
Help
Acrobat
Tell me what you want to do
Calibri
11
General
Conditional Formatting
Format as Table
Cell Styles
Insert
Delete
Format
Polylux 33474-Benfey-S2_B1-2_results.t
resolve biosciences
File
Options
Regions
Coloc-Analysis
Tools
no groups
Os01g0666400
Os01g0677400
Os01g0723100
Os01g0803300
Os01g0836900
Os01g0868600
Os01g0896200
Os01g0899700
Os02g0285700
Os02g0768300
Os03g0134800
Os03g0185700
Os03g0314500
Os04g0585100
Os05g0138200
Os05g0398800
Os06g0135900
Os06g0611100
Os06g0664800
Os06g0695700
Os07g0560300
Os07g0634400
Os08g0138600
Os08g0554100
Os09g0535400
>>
<<
color
show
name
count
1 Os03g0570800
2 OsMST1
3 Os03g0115700
4 UGT
5 Os05g0153300
6 Os04g0125700
-general settings-
upper-z: 5000
lower-z: 1
stroke-width: 1.0
ignore z
filled
-specific settings-
use rectangles
diameter: 17
color:
show
list
settings
update
Color scheme
Type here to search
69°F
ENG
4:40 PM
11/17/2023

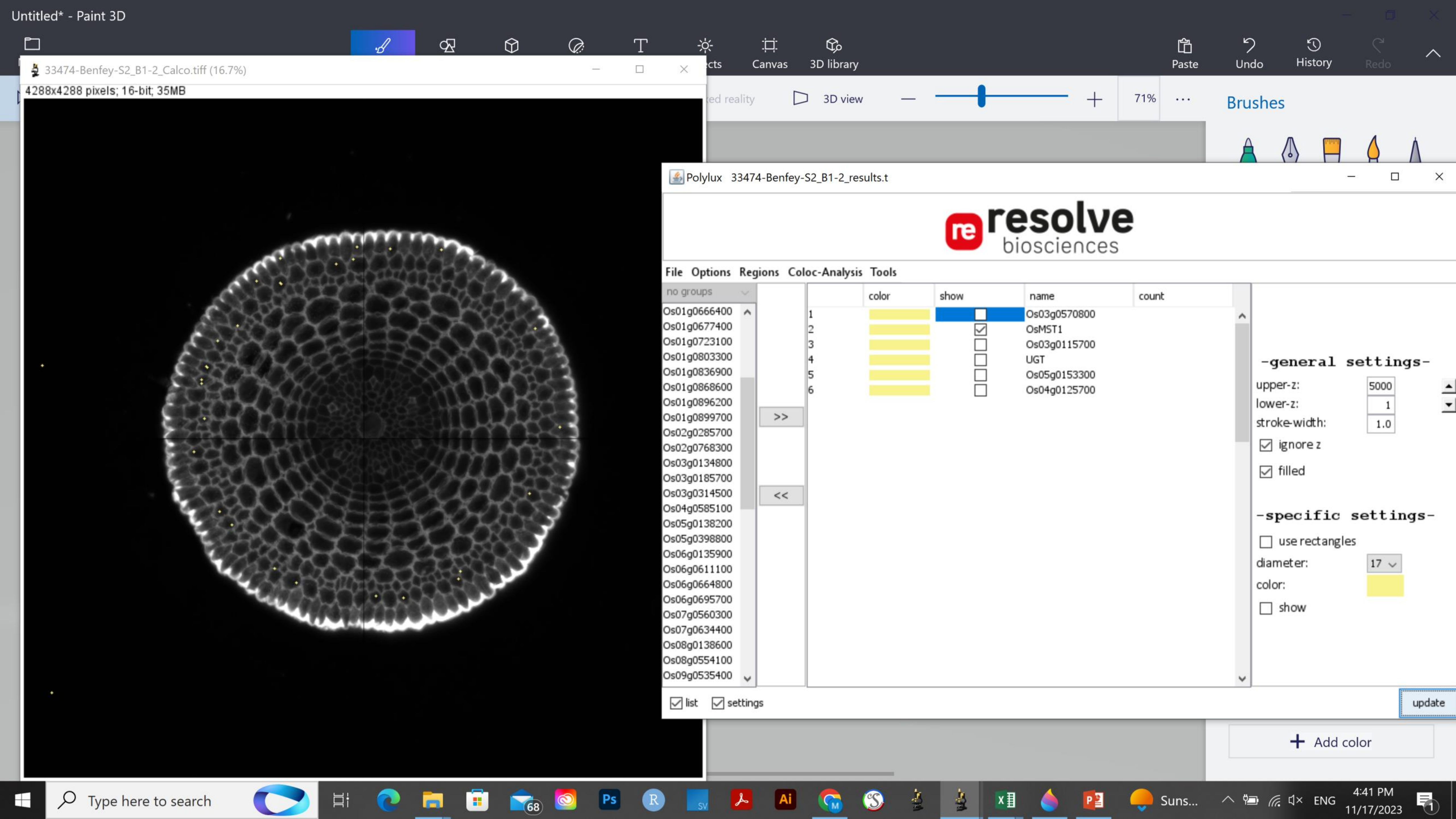
Untitled* - Paint 3D
Canvas
3D library
Paste
Undo
History
Redo
3D view
71%
Brushes
33474-Benfey-S2_B1-2_Calco.tiff (16.7%)
4288x4288 pixels; 16-bit; 35MB
Polylux 33474-Benfey-S2_B1-2_results.t
resolve biosciences
File Options Regions Coloc-Analysis Tools
no groups
Os01g0666400
Os01g0677400
Os01g0723100
Os01g0803300
Os01g0836900
Os01g0868600
Os01g0896200
Os01g0899700
Os02g0285700
Os02g0768300
Os03g0134800
Os03g0185700
Os03g0314500
Os04g0585100
Os05g0138200
Os05g0398800
Os06g0135900
Os06g0611100
Os06g0664800
Os06g0695700
Os07g0560300
Os07g0634400
Os08g0138600
Os08g0554100
Os09g0535400
color
show
name
count
1 Os03g0570800
2 OsMST1
3 Os03g0115700
4 UGT
5 Os05g0153300
6 Os04g0125700
-general settings-
upper-z: 5000
lower-z: 1
stroke-width: 1.0
ignore z
filled
-specific settings-
use rectangles
diameter: 17
color:
show
list
settings
update
Add color
Type here to search
Suns...
ENG
4:41 PM
11/17/2023

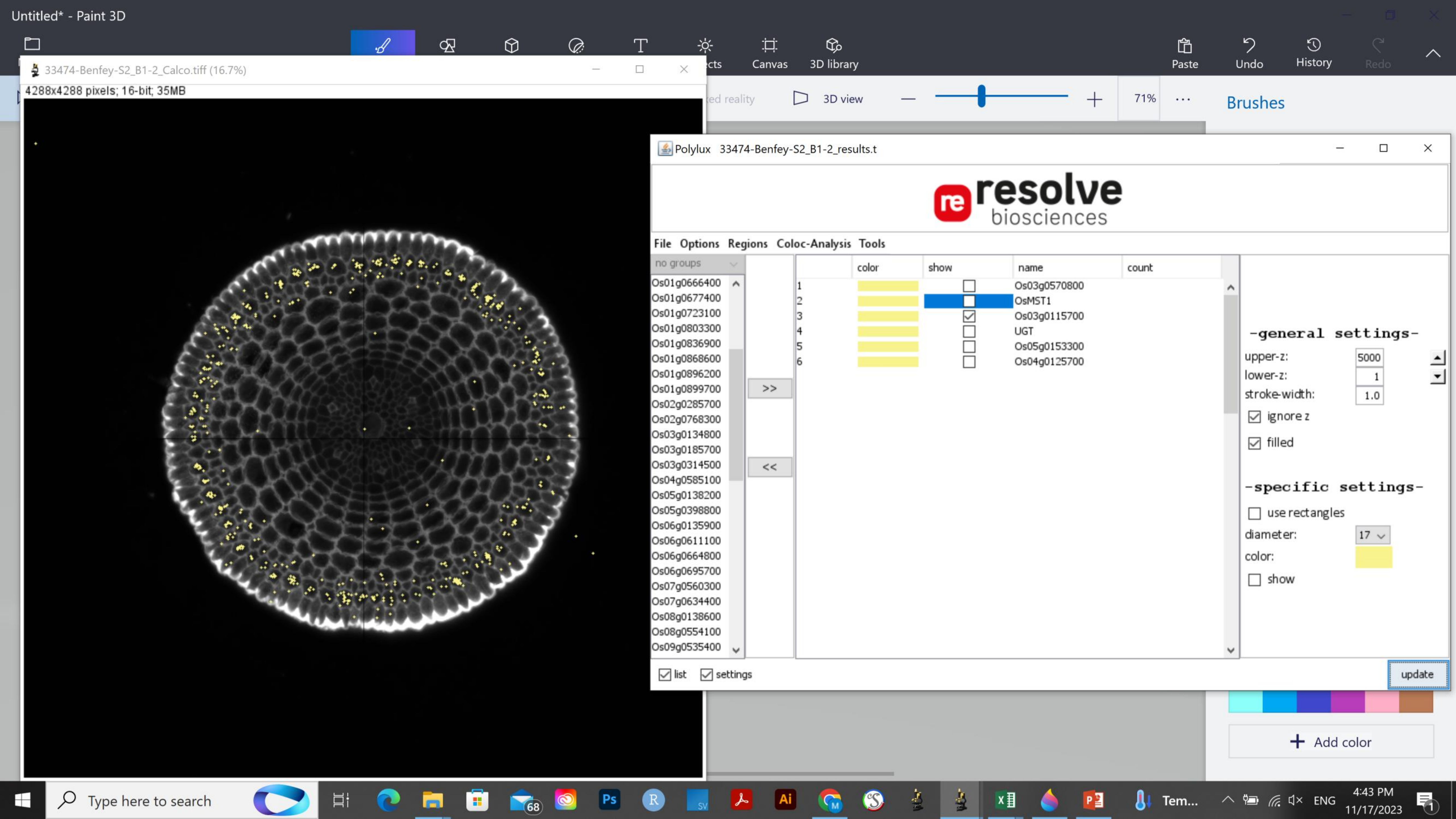
Untitled* - Paint 3D
Canvas
3D library
Paste
Undo
History
Redo
3D view
71%
Brushes
33474-Benfey-S2_B1-2_Calco.tiff (16.7%)
4288x4288 pixels; 16-bit; 35MB
Polylux 33474-Benfey-S2_B1-2_results.t
resolve biosciences
File Options Regions Coloc-Analysis Tools
no groups
Os01g0666400
Os01g0677400
Os01g0723100
Os01g0803300
Os01g0836900
Os01g0868600
Os01g0896200
Os01g0899700
Os02g0285700
Os02g0768300
Os03g0134800
Os03g0185700
Os03g0314500
Os04g0585100
Os05g0138200
Os05g0398800
Os06g0135900
Os06g0611100
Os06g0664800
Os06g0695700
Os07g0560300
Os07g0634400
Os08g0138600
Os08g0554100
Os09g0535400
>>
<<
color show name count
1 Os03g0570800
2 OsMST1
3 Os03g0115700
4 UGT
5 Os05g0153300
6 Os04g0125700
-general settings-
upper-z: 5000
lower-z: 1
stroke-width: 1.0
ignore z
filled
-specific settings-
use rectangles
diameter: 17
color:
show
list
settings
update
Add color
Type here to search
Tem...
ENG
4:43 PM
11/17/2023

Sclerenchyma

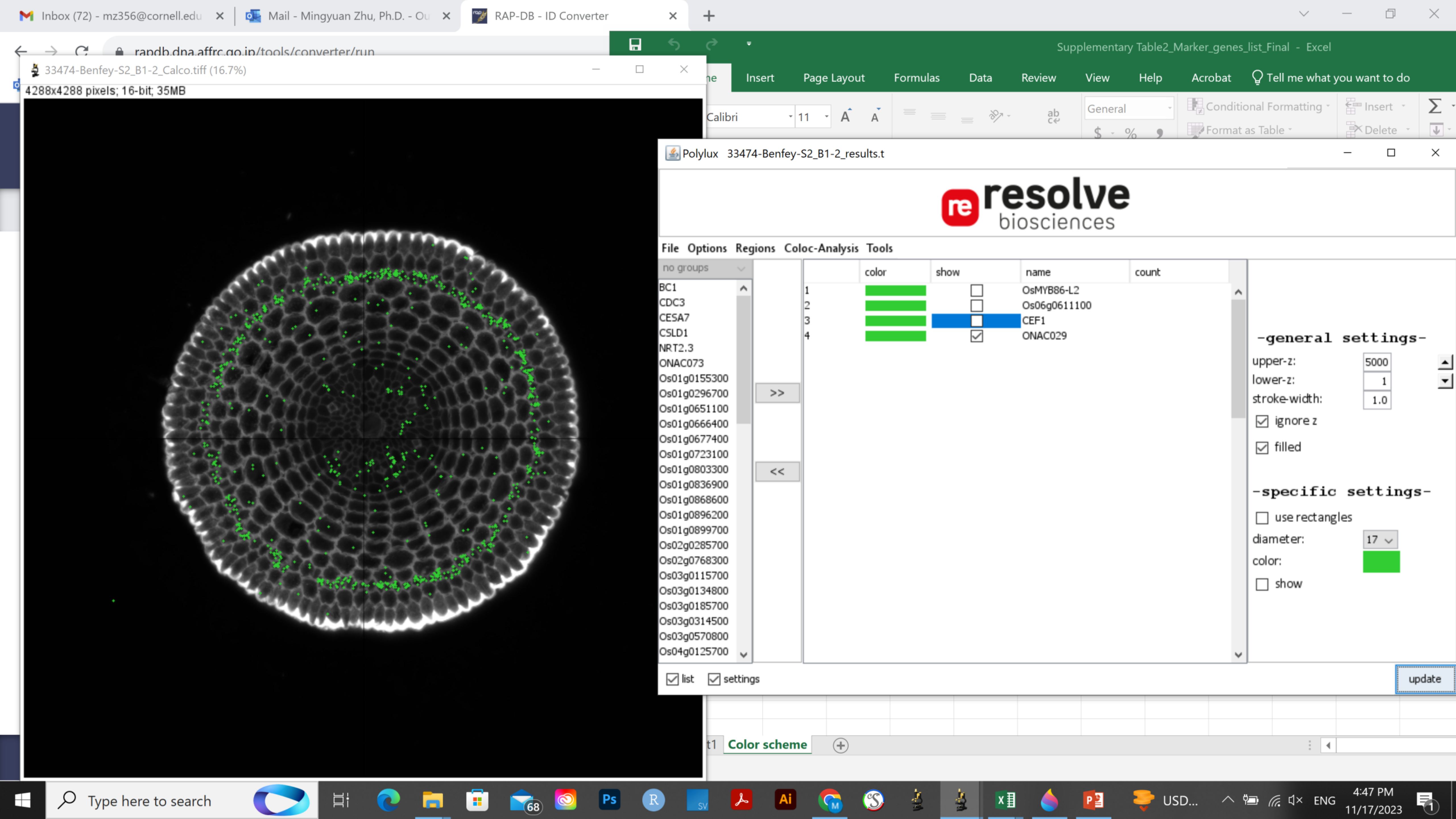

Inbox (72) - mz356@cornell.edu
Mail - Mingyuan Zhu, Ph.D. - Ou
RAP-DB - ID Converter
rapdb.dna.affrc.go.jp/tools/converter/run
Supplementary Table2_Marker_genes_list_Final - Excel
Insert
Page Layout
Formulas
Data
Review
View
Help
Acrobat
Tell me what you want to do
Calibri
11
General
Conditional Formatting
Format as Table
Insert
Delete
33474-Benfey-S2_B1-2_Calco.tiff (16.7%)
4288x4288 pixels; 16-bit; 35MB
Polylux 33474-Benfey-S2_B1-2_results.t
resolve
biosciences
File Options Regions Coloc-Analysis Tools
no groups
BC1
CDC3
CESA7
CSLD1
NRT2.3
ONAC073
Os01g0155300
Os01g0296700
Os01g0651100
Os01g0666400
Os01g0677400
Os01g0723100
Os01g0803300
Os01g0836900
Os01g0868600
Os01g0896200
Os01g0899700
Os02g0285700
Os02g0768300
Os03g0115700
Os03g0134800
Os03g0185700
Os03g0314500
Os03g0570800
Os04g0125700
>>
<<
color
show
name
count
1
OsMYB86-L2
2
Os06g0611100
3
CEF1
4
ONAC029
-general settings-
upper-z:
5000
lower-z:
1
stroke-width:
1.0
ignore z
filled
-specific settings-
use rectangles
diameter:
17
color:
show
list
settings
update
Color scheme
Type here to search
USD...
ENG
4:47 PM
11/17/2023

Cortex

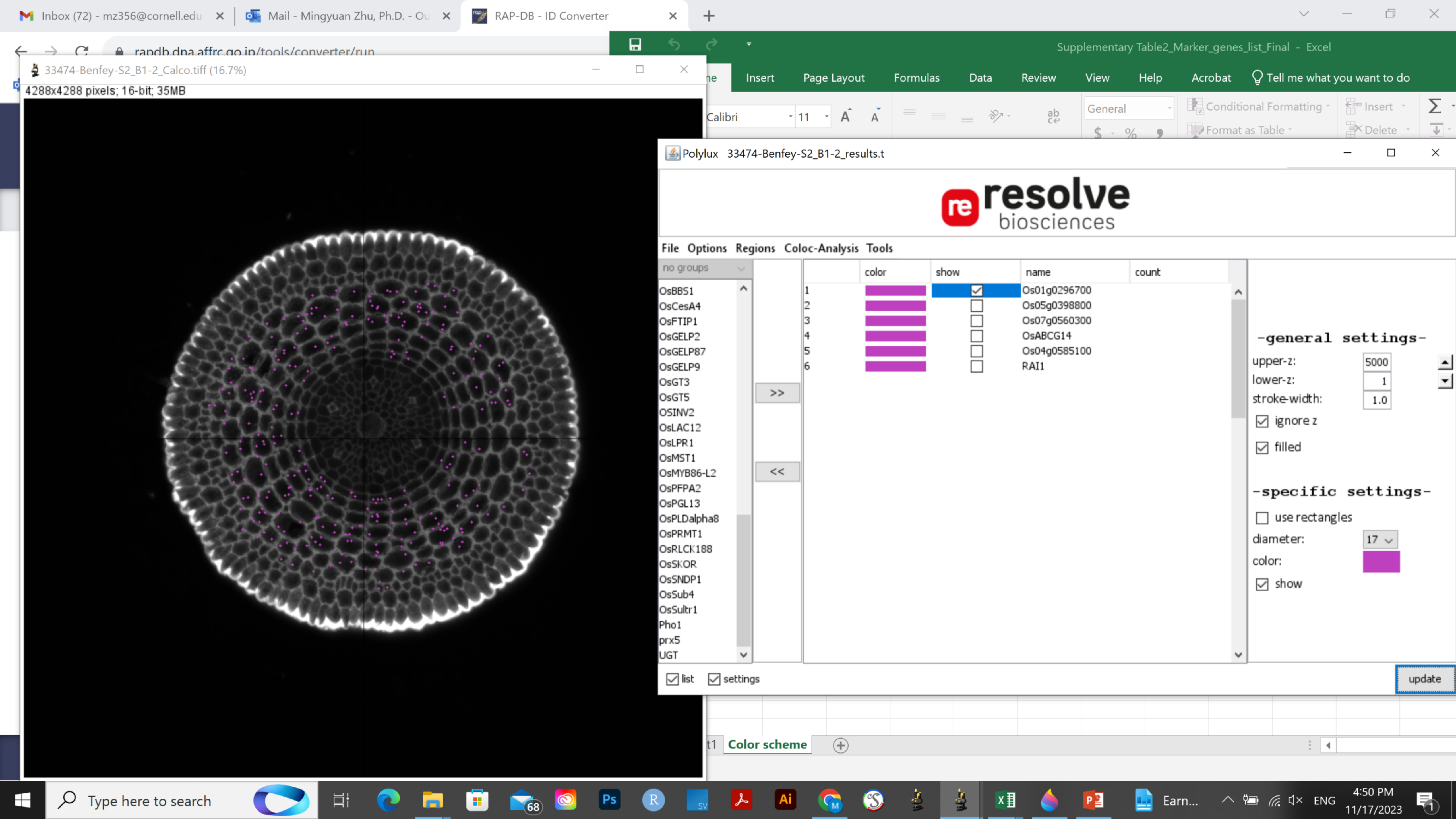

Inbox (72) - mz356@cornell.edu
Mail - Mingyuan Zhu, Ph.D. - Ou
RAP-DB - ID Converter
rapdb.dna.affrc.go.jp/tools/converter/run
33474-Benfey-S2_B1-2_Calco.tiff (16.7%)
4288x4288 pixels; 16-bit; 35MB
Supplementary Table2_Marker_genes_list_Final - Excel
Insert
Page Layout
Formulas
Data
Review
View
Help
Acrobat
Tell me what you want to do
Calibri
11
General
Conditional Formatting
Format as Table
Insert
Delete
Polylux 33474-Benfey-S2_B1-2_results.t
resolve biosciences
File Options Regions Coloc-Analysis Tools
no groups
OsBBS1
OsCesA4
OsFTIP1
OsGELP2
OsGELP87
OsGELP9
OsGT3
OsGT5
OSINV2
OsLAC12
OsLPR1
OsMST1
OsMYB86-L2
OsPFPA2
OsPGL13
OsPLDalpha8
OsPRMT1
OsRLCK188
OsSKOR
OsSNDP1
OsSub4
OsSultr1
Pho1
prx5
UGT
>>
<<
color
show
name
count
1 Os01g0296700
2 Os05g0398800
3 Os07g0560300
4 OsABCG14
5 Os04g0585100
6 RAI1
-general settings-
upper-z: 5000
lower-z: 1
stroke-width: 1.0
ignore z
filled
-specific settings-
use rectangles
diameter: 17
color:
show
list
settings
update
Color scheme
Type here to search
ENG
4:50 PM
11/17/2023

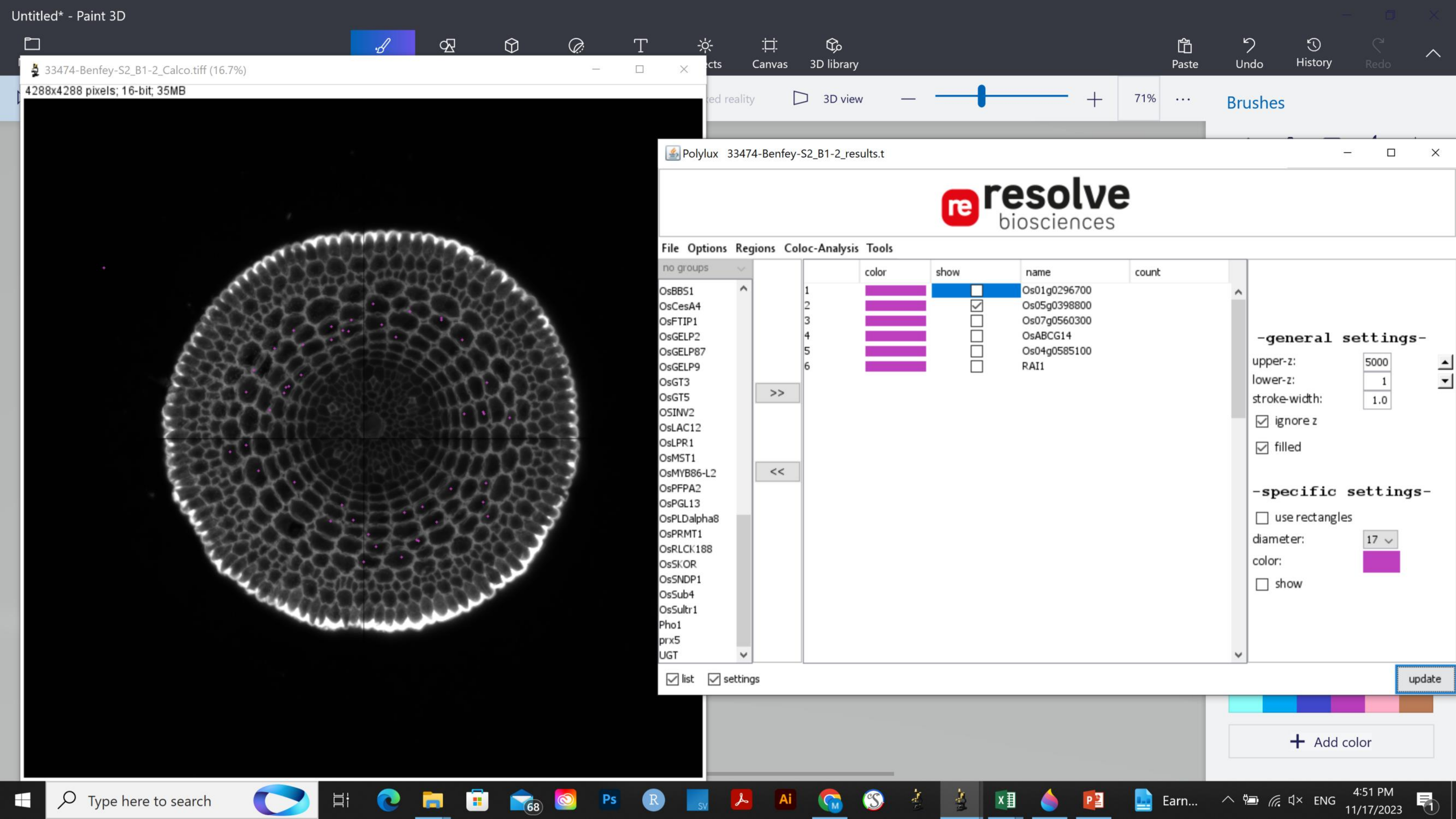
Untitled* - Paint 3D
33474-Benfey-S2_B1-2_Calco.tiff (16.7%)
4288x4288 pixels; 16-bit; 35MB
Canvas
3D library
Paste
Undo
History
Redo
3D view
71%
Brushes
Polylux 33474-Benfey-S2_B1-2_results.t
resolve biosciences
File Options Regions Coloc-Analysis Tools
no groups
OsBBS1
OsCesA4
OsFTIP1
OsGELP2
OsGELP87
OsGELP9
OsGT3
OsGT5
OSINV2
OsLAC12
OsLPR1
OsMST1
OsMYB86-L2
OsPFPA2
OsPGL13
OsPLDalpha8
OsPRMT1
OsRLCK188
OsSKOR
OsSNDP1
OsSub4
OsSultr1
Pho1
prx5
UGT
>>
<<
color
show
name
count
1 Os01g0296700
2 Os05g0398800
3 Os07g0560300
4 OsABCG14
5 Os04g0585100
6 RAI1
-general settings-
upper-z: 5000
lower-z: 1
stroke-width: 1.0
ignore z
filled
-specific settings-
use rectangles
diameter: 17
color:
show
list
settings
update
Add color
Type here to search
ENG
4:51 PM
11/17/2023

Untitled* - Paint 3D

33474-Benfey-S2_B1-2_Calco.tiff (16.7%)

4288x4288 pixels; 16-bit; 35MB

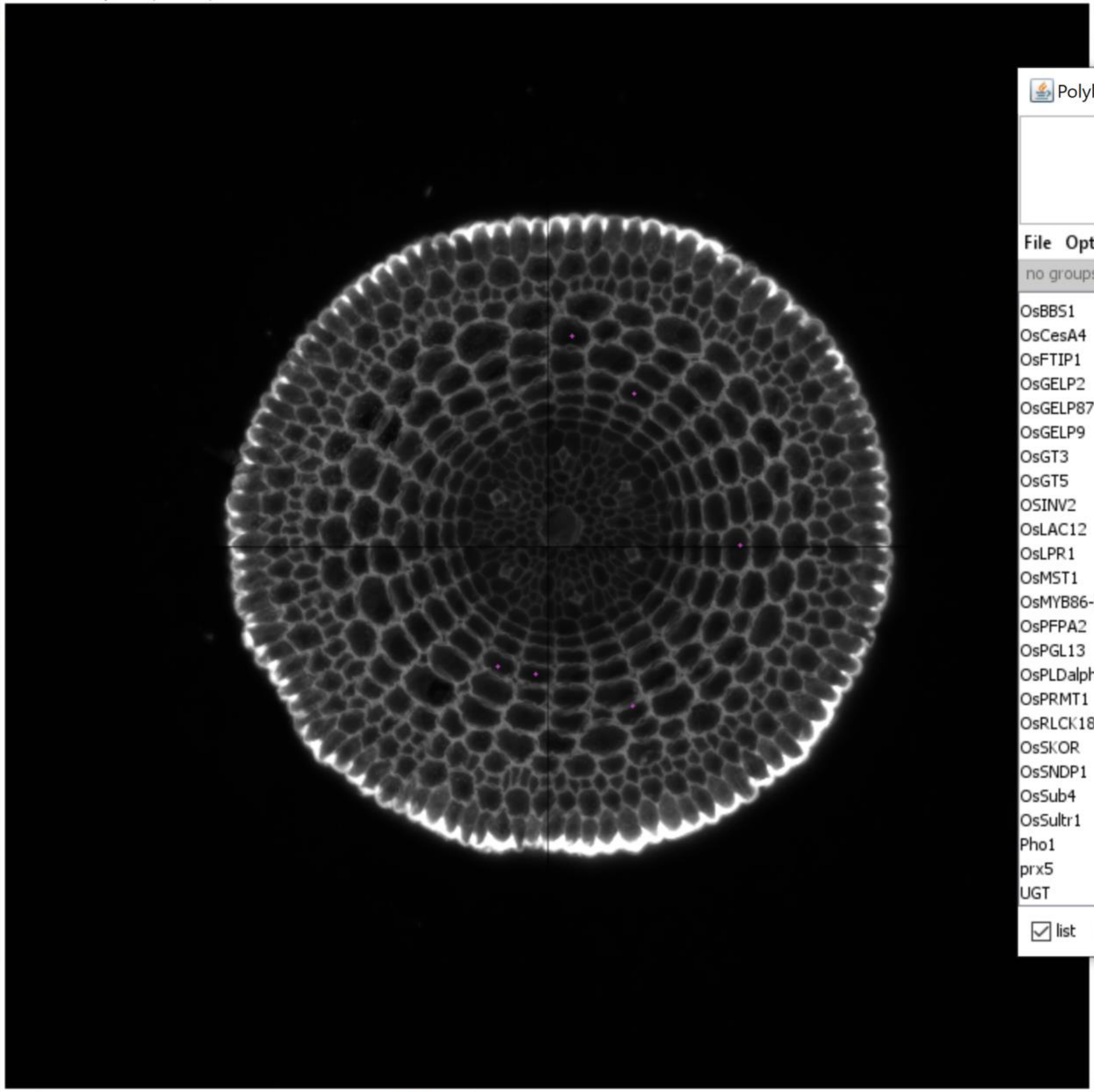

Canvas | 3D library | Paste | Undo | History | Redo

3D view | 71% | Brushes

Polylux 33474-Benfey-S2_B1-2_results.t

resolve biosciences

File Options Regions Coloc-Analysis Tools

no groups

OsBBS1
OsCesA4
OsFTIP1
OsGELP2
OsGELP87
OsGELP9
OsGT3
OsGT5
OSINV2
OsLAC12
OsLPR1
OsMST1
OsMYB86-L2
OsPFPA2
OsPGL13
OsPLDalpha8
OsPRMT1
OsRLCK188
OsSKOR
OsSNDP1
OsSub4
OsSultr1
Pho1
prx5
UGT

>>

<<

	color	show	name	count
1		☐	Os01g0296700	
2		☐	Os05g0398800	
3		☐	Os07g0560300	
4		☑	OsABCG14	
5		☐	Os04g0585100	
6		☐	RAI1	

-general settings-

upper-z: 5000

lower-z: 1

stroke-width: 1.0

☑ ignore z

☑ filled

-specific settings-

☐ use rectangles

diameter: 17

color:

☐ show

☑ list ☑ settings

update

Add color

Type here to search

68°F ENG 4:52 PM 11/17/2023

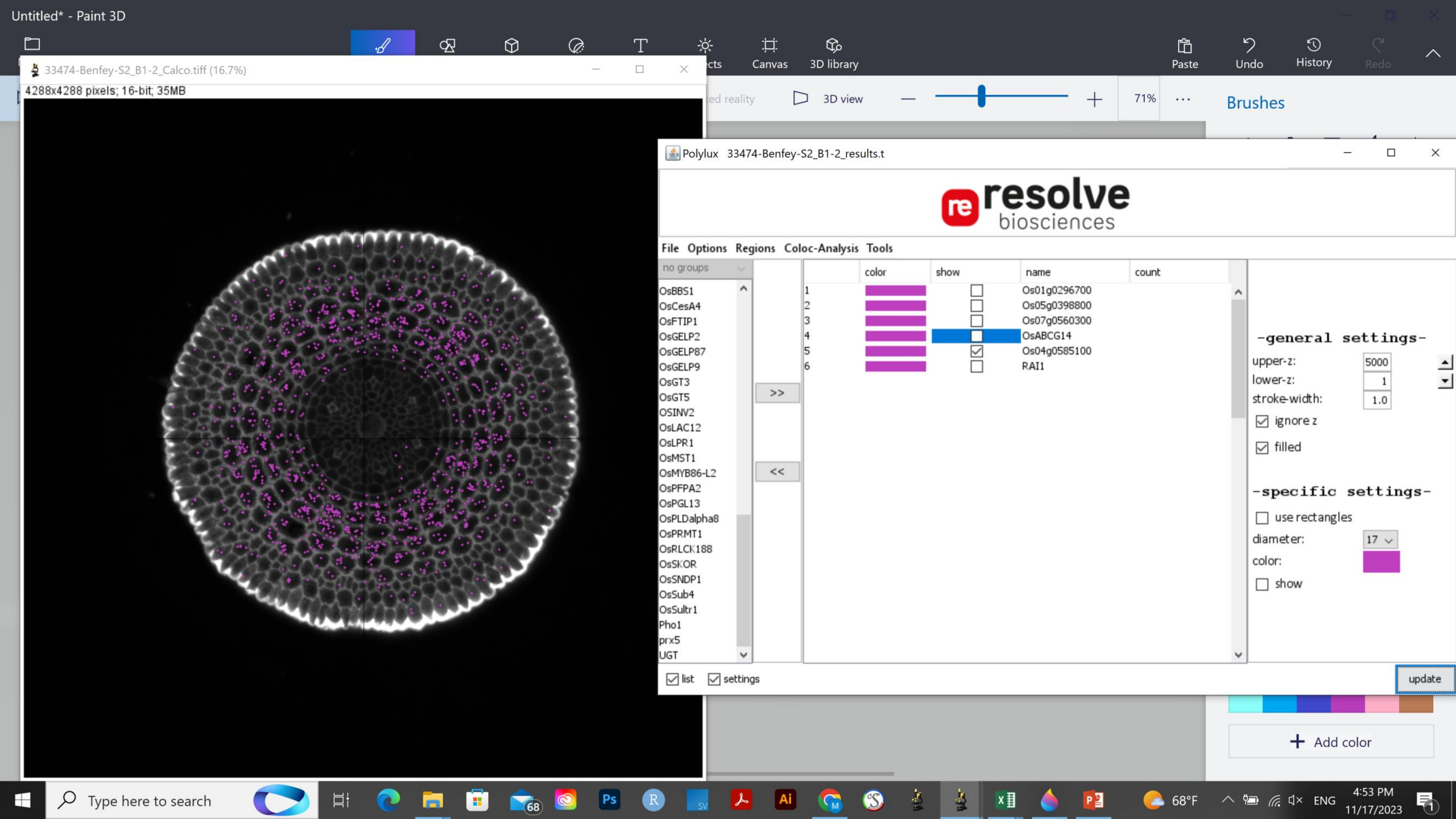

Untitled* - Paint 3D
33474-Benfey-S2_B1-2_Calco.tiff (16.7%)
4288x4288 pixels; 16-bit; 35MB
Canvas
3D library
Paste
Undo
History
Redo
3D view
71%
Brushes
Polylux 33474-Benfey-S2_B1-2_results.t
resolve biosciences
File Options Regions Coloc-Analysis Tools
no groups
OsBBS1
OsCesA4
OsFTIP1
OsGELP2
OsGELP87
OsGELP9
OsGT3
OsGT5
OSINV2
OsLAC12
OsLPR1
OsMST1
OsMYB86-L2
OsPFPA2
OsPGL13
OsPLDalpha8
OsPRMT1
OsRLCK188
OsSKOR
OsSNDP1
OsSub4
OsSultr1
Pho1
prx5
UGT
>>
<<
color
show
name
count
1 Os01g0296700
2 Os05g0398800
3 Os07g0560300
4 OsABCG14
5 Os04g0585100
6 RAI1
-general settings-
upper-z: 5000
lower-z: 1
stroke-width: 1.0
ignore z
filled
-specific settings-
use rectangles
diameter: 17
color:
show
list
settings
update
Add color
Type here to search
68
68°F
ENG
4:53 PM
11/17/2023

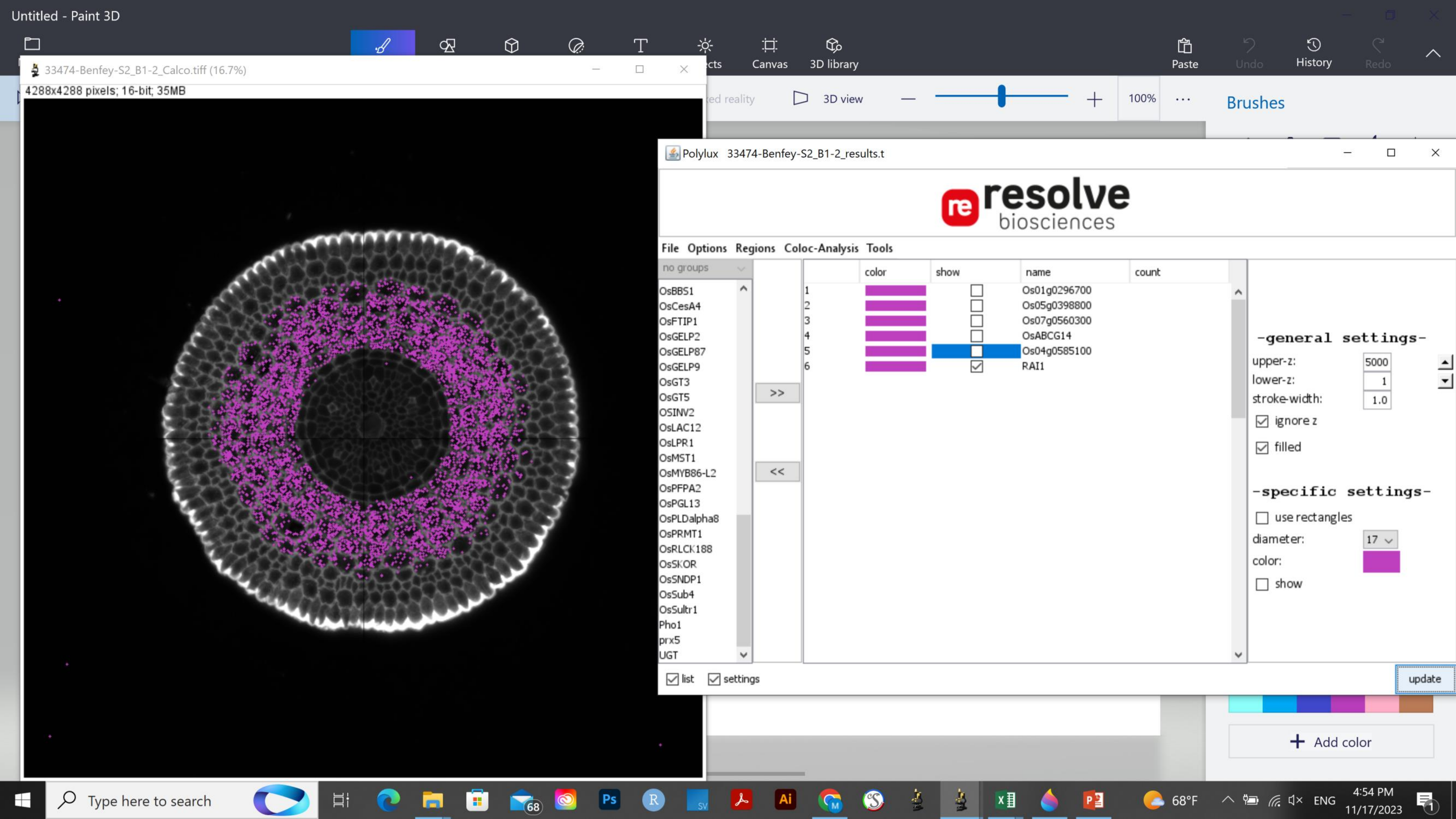
Untitled - Paint 3D
33474-Benfey-S2_B1-2_Calco.tiff (16.7%)
4288x4288 pixels; 16-bit; 35MB
Canvas
3D library
Paste
Undo
History
Redo
3D view
100%
Brushes
Polylux 33474-Benfey-S2_B1-2_results.t
resolve biosciences
File Options Regions Coloc-Analysis Tools
no groups
OsBBS1
OsCesA4
OsFTIP1
OsGELP2
OsGELP87
OsGELP9
OsGT3
OsGT5
OSINV2
OsLAC12
OsLPR1
OsMST1
OsMYB86-L2
OsPFPA2
OsPGL13
OsPLDalpha8
OsPRMT1
OsRLCK188
OsSKOR
OsSNDP1
OsSub4
OsSultr1
Pho1
prx5
UGT
>>
<<
color show name count
1 Os01g0296700
2 Os05g0398800
3 Os07g0560300
4 OsABCG14
5 Os04g0585100
6 RAI1
-general settings-
upper-z: 5000
lower-z: 1
stroke-width: 1.0
ignore z
filled
-specific settings-
use rectangles
diameter: 17
color:
show
list
settings
update
Add color
Type here to search
68°F
ENG
4:54 PM
11/17/2023

Endodermis

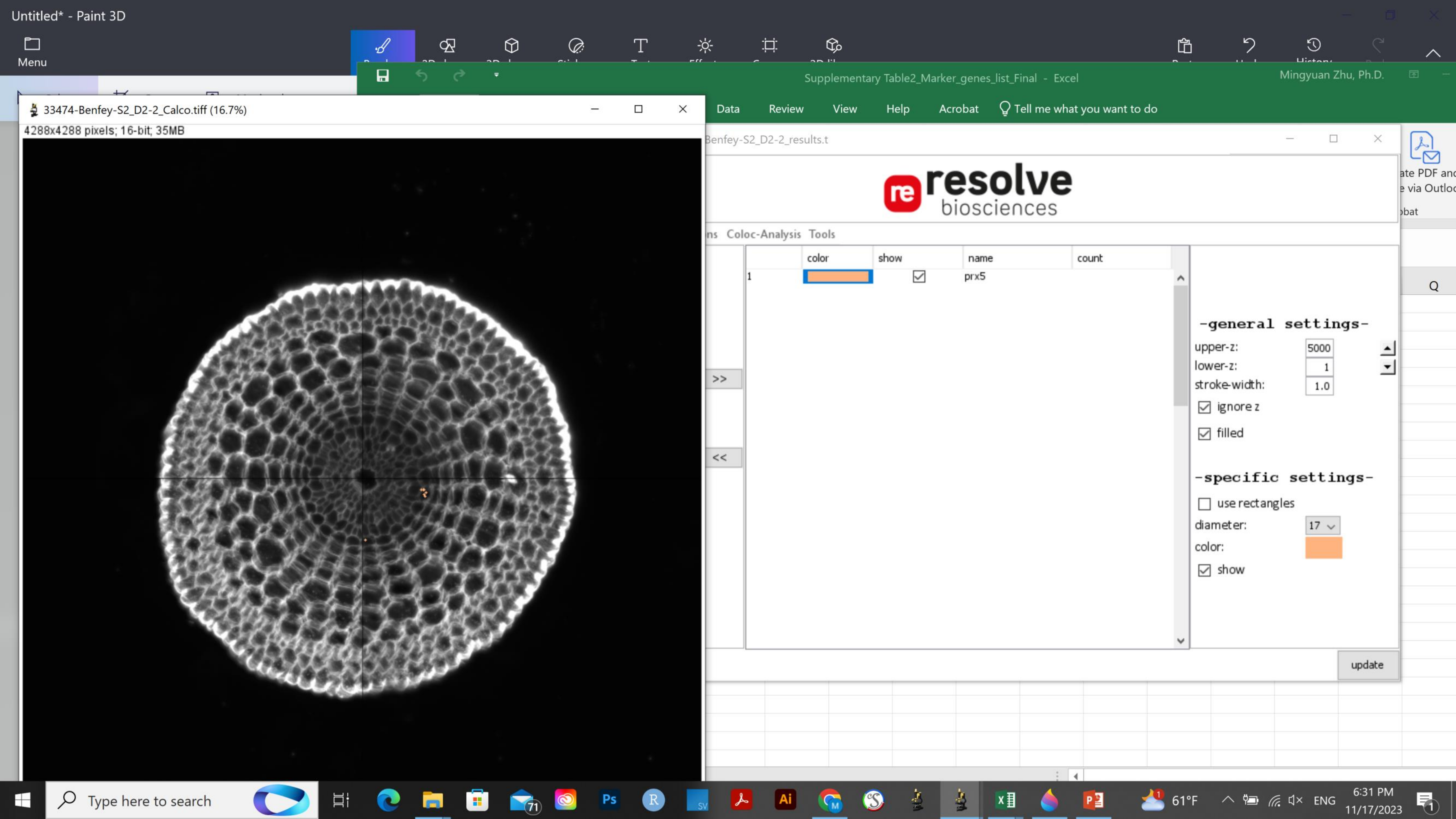
Untitled* - Paint 3D
Menu
Supplementary Table2_Marker_genes_list_Final - Excel
Mingyuan Zhu, Ph.D.
33474-Benfey-S2_D2-2_Calco.tiff (16.7%)
4288x4288 pixels; 16-bit; 35MB
Data
Review
View
Help
Acrobat
Tell me what you want to do
Benfey-S2_D2-2_results.t
resolve
biosciences
Coloc-Analysis
Tools
color
show
name
count
1
prx5
-general settings-
upper-z:
5000
lower-z:
1
stroke-width:
1.0
ignore z
filled
-specific settings-
use rectangles
diameter:
17
color:
show
update
Type here to search
61°F
ENG
6:31 PM
11/17/2023

Pericycle/Procambium

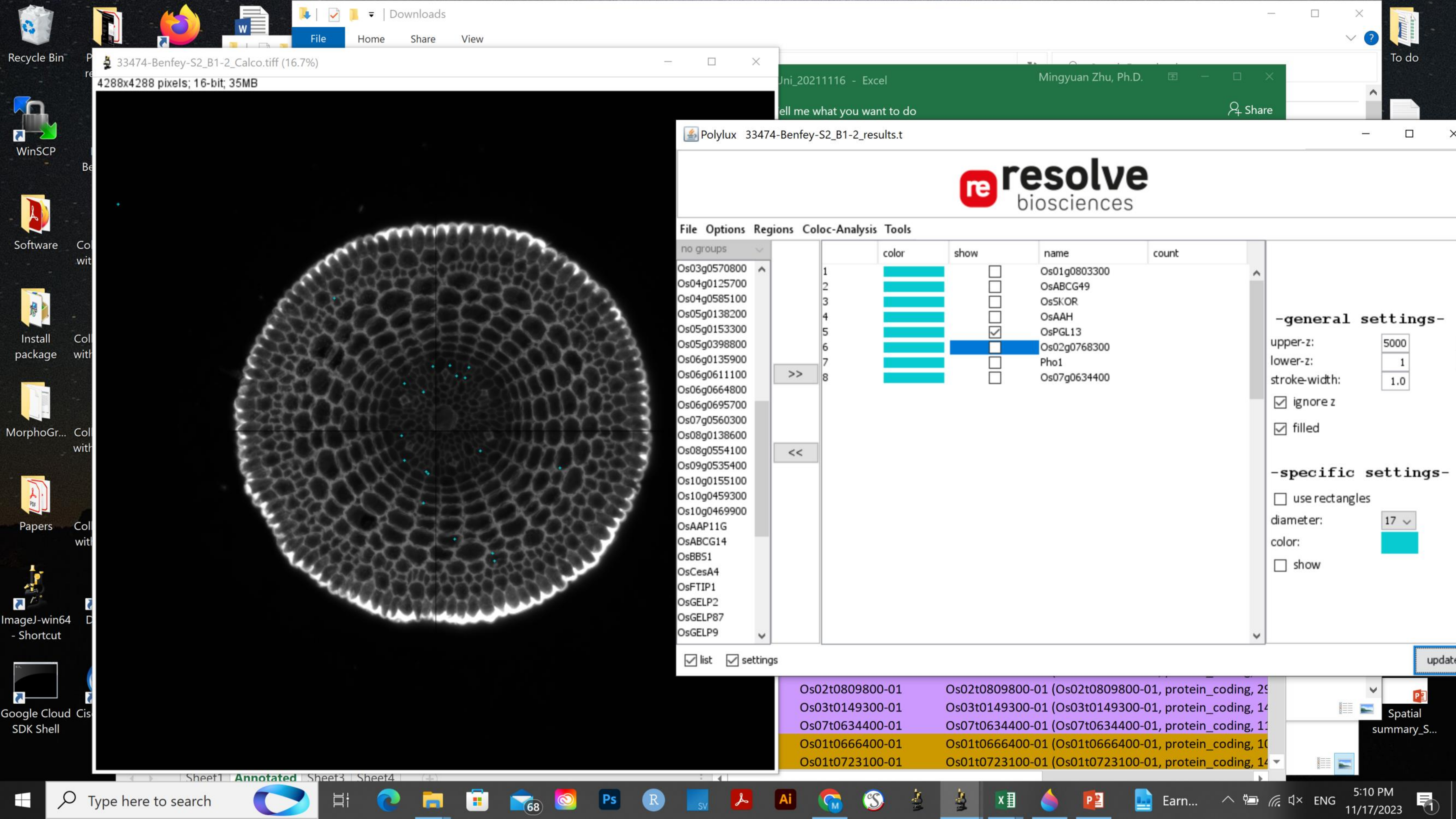

33474-Benfey-S2_B1-2_Calco.tiff (16.7%)
4288x4288 pixels; 16-bit; 35MB
Polylux 33474-Benfey-S2_B1-2_results.t
resolve biosciences
File Options Regions Coloc-Analysis Tools
no groups
Os03g0570800
Os04g0125700
Os04g0585100
Os05g0138200
Os05g0153300
Os05g0398800
Os06g0135900
Os06g0611100
Os06g0664800
Os06g0695700
Os07g0560300
Os08g0138600
Os08g0554100
Os09g0535400
Os10g0155100
Os10g0459300
Os10g0469900
OsAAP11G
OsABCG14
OsBBS1
OsCesA4
OsFTIP1
OsGELP2
OsGELP87
OsGELP9
>>
<<
color
show
name
count
1 Os01g0803300
2 OsABCG49
3 OsSKOR
4 OsAAH
5 OsPGL13
6 Os02g0768300
7 Pho1
8 Os07g0634400
-general settings-
upper-z: 5000
lower-z: 1
stroke-width: 1.0
ignore z
filled
-specific settings-
use rectangles
diameter: 17
color:
show
list
settings
update
Downloads
File Home Share View
Uni_20211116 - Excel
Mingyuan Zhu, Ph.D.
ell me what you want to do
Share
Os02t0809800-01 Os02t0809800-01 (Os02t0809800-01, protein_coding, 2
Os03t0149300-01 Os03t0149300-01 (Os03t0149300-01, protein_coding, 14
Os07t0634400-01 Os07t0634400-01 (Os07t0634400-01, protein_coding, 11
Os01t0666400-01 Os01t0666400-01 (Os01t0666400-01, protein_coding, 10
Os01t0723100-01 Os01t0723100-01 (Os01t0723100-01, protein_coding, 14
Sheet1 Annotated Sheet3 Sheet4
Recycle Bin
WinSCP
Software
Install package
MorphoGr...
Papers
ImageJ-win64 - Shortcut
Google Cloud SDK Shell
To do
Spatial summary_S...
Type here to search
Earn...
ENG
5:10 PM
11/17/2023

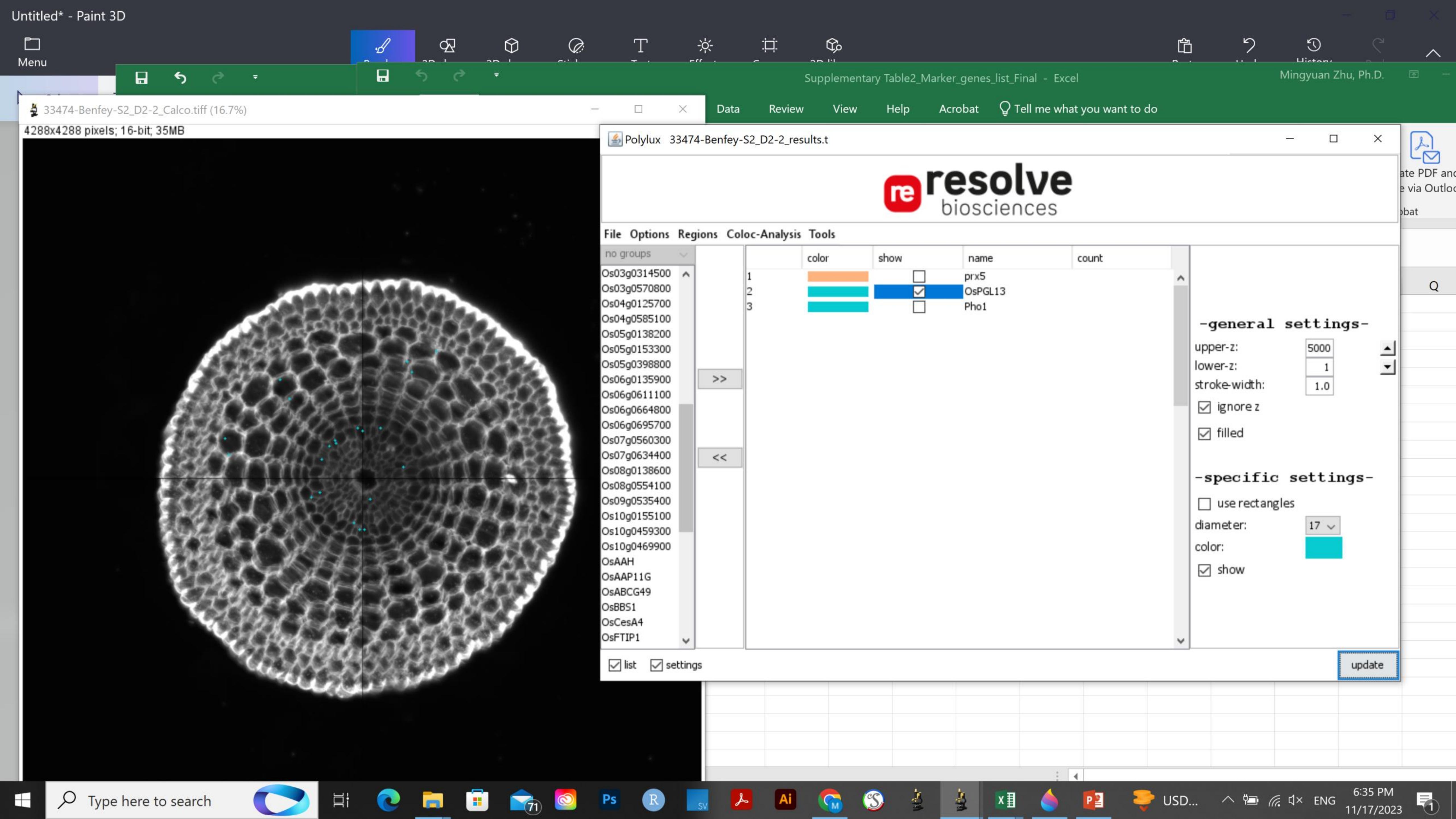
Untitled* - Paint 3D
Menu
Supplementary Table2_Marker_genes_list_Final - Excel
Mingyuan Zhu, Ph.D.
Data
Review
View
Help
Acrobat
Tell me what you want to do
33474-Benfey-S2_D2-2_Calco.tiff (16.7%)
4288x4288 pixels; 16-bit; 35MB
Polylux 33474-Benfey-S2_D2-2_results.t
resolve
biosciences
File
Options
Regions
Coloc-Analysis
Tools
no groups
Os03g0314500
Os03g0570800
Os04g0125700
Os04g0585100
Os05g0138200
Os05g0153300
Os05g0398800
Os06g0135900
Os06g0611100
Os06g0664800
Os06g0695700
Os07g0560300
Os07g0634400
Os08g0138600
Os08g0554100
Os09g0535400
Os10g0155100
Os10g0459300
Os10g0469900
OsAAH
OsAAP11G
OsABCG49
OsBBS1
OsCesA4
OsFTIP1
>>
<<
color
show
name
count
1
prx5
2
OsPGL13
3
Pho1
-general settings-
upper-z:
5000
lower-z:
1
stroke-width:
1.0
ignore z
filled
-specific settings-
use rectangles
diameter:
17
color:
show
list
settings
update
Type here to search
6:35 PM
11/17/2023
ENG

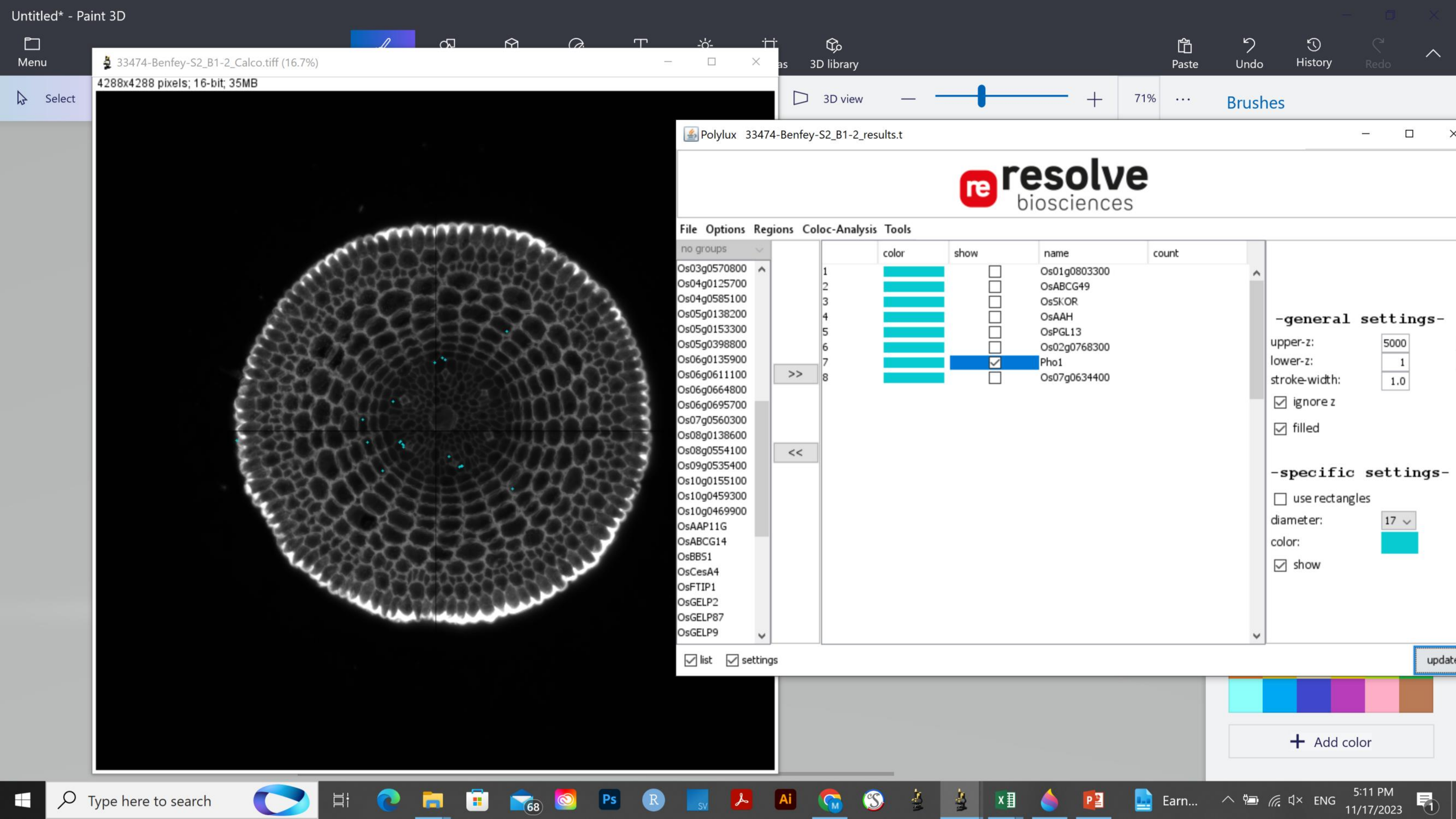
Untitled* - Paint 3D
Menu
3D library
Paste
Undo
History
Redo
Select
3D view
71%
Brushes
33474-Benfey-S2_B1-2_Calco.tiff (16.7%)
4288x4288 pixels; 16-bit; 35MB
Polylux 33474-Benfey-S2_B1-2_results.t
resolve biosciences
File Options Regions Coloc-Analysis Tools
no groups
Os03g0570800
Os04g0125700
Os04g0585100
Os05g0138200
Os05g0153300
Os05g0398800
Os06g0135900
Os06g0611100
Os06g0664800
Os06g0695700
Os07g0560300
Os08g0138600
Os08g0554100
Os09g0535400
Os10g0155100
Os10g0459300
Os10g0469900
OsAAP11G
OsABCG14
OsBBS1
OsCesA4
OsFTIP1
OsGELP2
OsGELP87
OsGELP9
>>
<<
color show name count
1 Os01g0803300
2 OsABCG49
3 OsSKOR
4 OsAAH
5 OsPGL13
6 Os02g0768300
7 Pho1
8 Os07g0634400
-general settings-
upper-z: 5000
lower-z: 1
stroke-width: 1.0
ignore z
filled
-specific settings-
use rectangles
diameter: 17
color:
show
list
settings
update
Add color
Type here to search
68
ENG
5:11 PM
11/17/2023

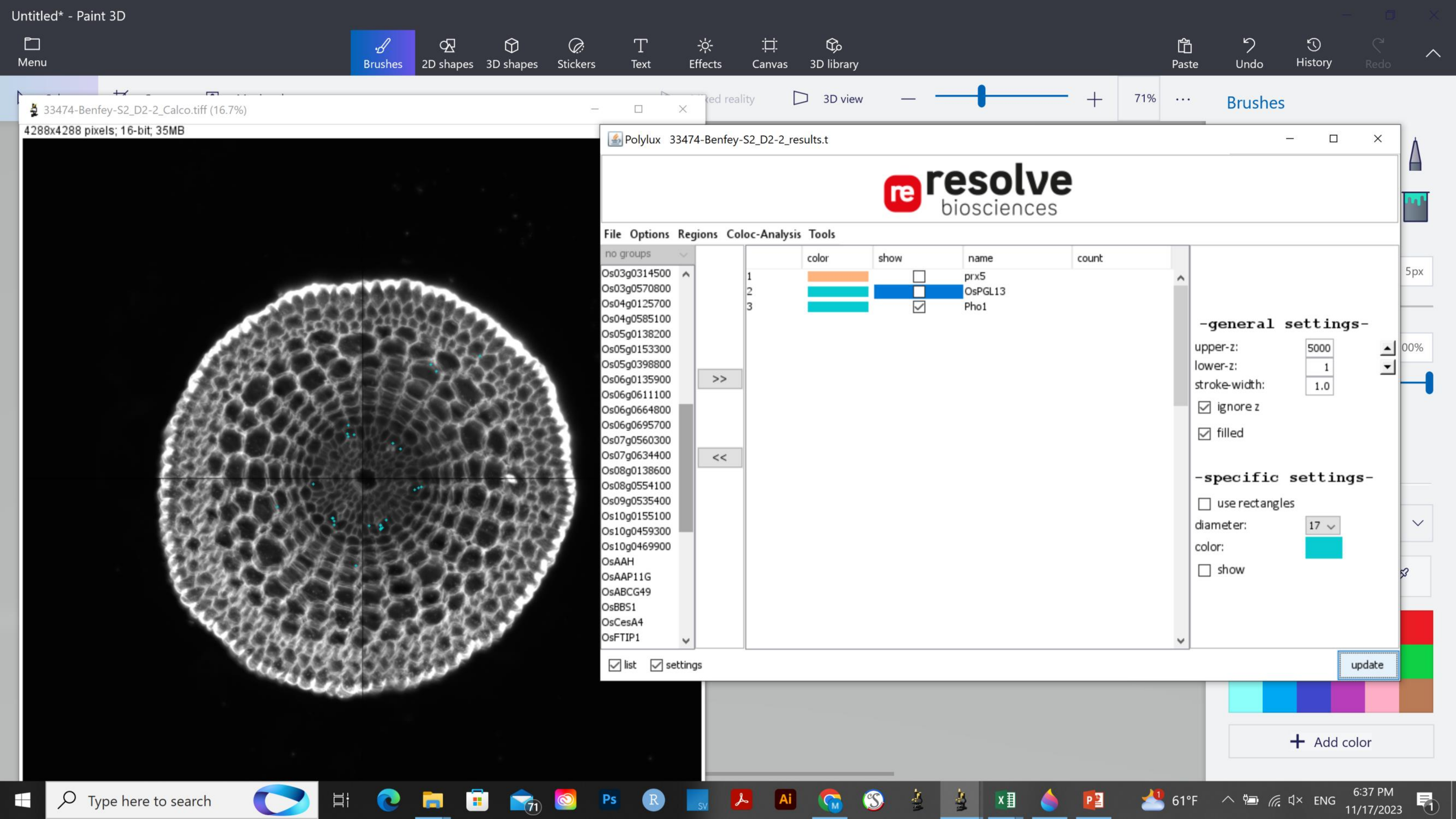
Untitled* - Paint 3D
Menu
Brushes
2D shapes
3D shapes
Stickers
Text
Effects
Canvas
3D library
Paste
Undo
History
Redo
3D view
71%
Brushes
33474-Benfey-S2_D2-2_Calco.tiff (16.7%)
4288x4288 pixels; 16-bit; 35MB
Polylux 33474-Benfey-S2_D2-2_results.t
resolve biosciences
File Options Regions Coloc-Analysis Tools
no groups
Os03g0314500
Os03g0570800
Os04g0125700
Os04g0585100
Os05g0138200
Os05g0153300
Os05g0398800
Os06g0135900
Os06g0611100
Os06g0664800
Os06g0695700
Os07g0560300
Os07g0634400
Os08g0138600
Os08g0554100
Os09g0535400
Os10g0155100
Os10g0459300
Os10g0469900
OsAAH
OsAAP11G
OsABCG49
OsBBS1
OsCesA4
OsFTIP1
color show name count
1 prx5
2 OsPGL13
3 Pho1
-general settings-
upper-z: 5000
lower-z: 1
stroke-width: 1.0
ignore z
filled
-specific settings-
use rectangles
diameter: 17
color:
show
list
settings
update
5px
Add color
Type here to search
61°F
ENG
6:37 PM
11/17/2023

Phloem

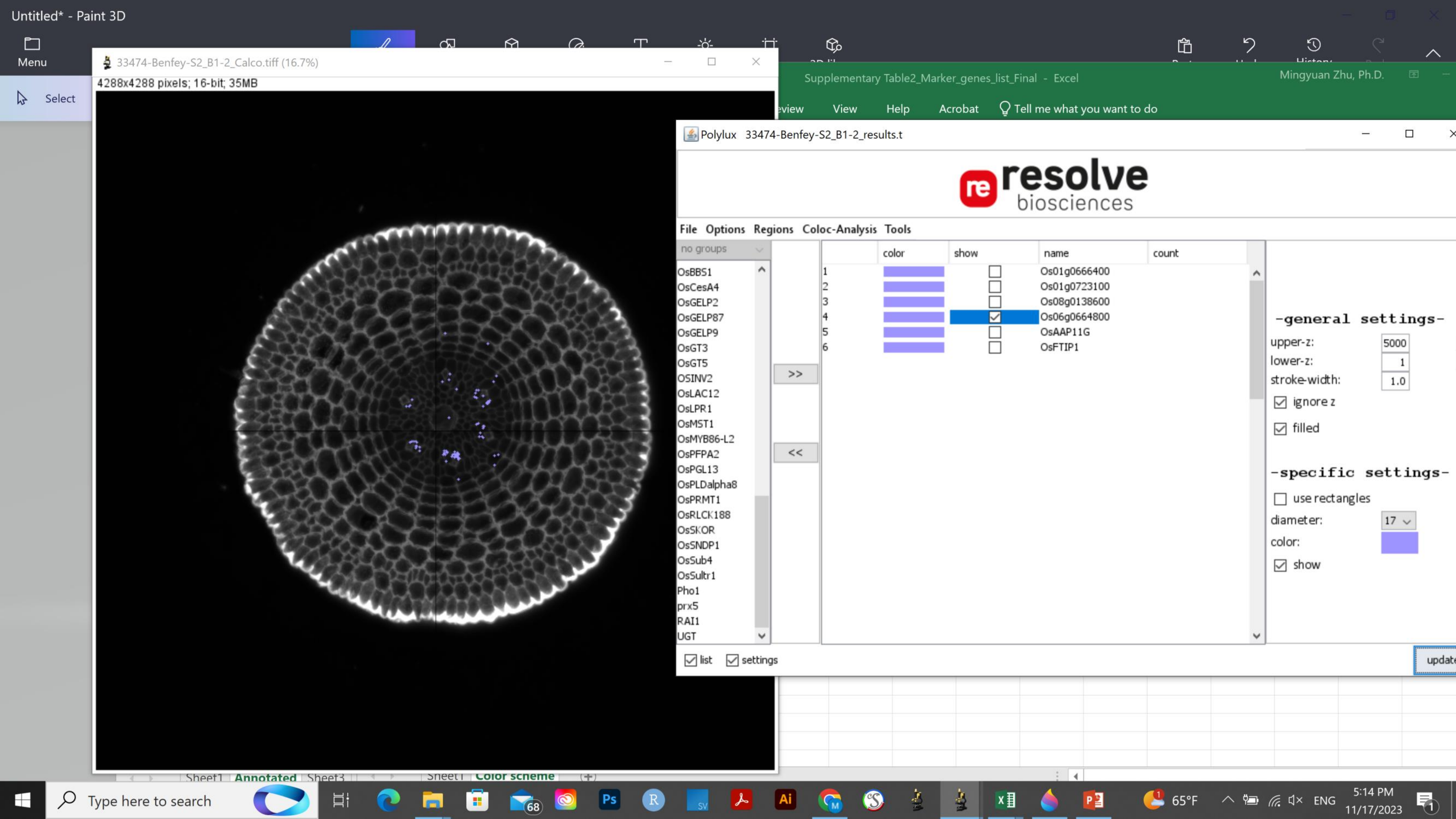
Untitled* - Paint 3D
Menu
Select
33474-Benfey-S2_B1-2_Calco.tiff (16.7%)
4288x4288 pixels; 16-bit; 35MB
Supplementary Table2_Marker_genes_list_Final - Excel
Mingyuan Zhu, Ph.D.
View
Help
Acrobat
Tell me what you want to do
Polylux 33474-Benfey-S2_B1-2_results.t
resolve biosciences
File Options Regions Coloc-Analysis Tools
no groups
OsBBS1
OsCesA4
OsGELP2
OsGELP87
OsGELP9
OsGT3
OsGT5
OSINV2
OsLAC12
OsLPR1
OsMST1
OsMYB86-L2
OsPFPA2
OsPGL13
OsPLDalpha8
OsPRMT1
OsRLCK188
OsSKOR
OsSNDP1
OsSub4
OsSultr1
Pho1
prx5
RAI1
UGT
>>
<<
color
show
name
count
1 Os01g0666400
2 Os01g0723100
3 Os08g0138600
4 Os06g0664800
5 OsAAP11G
6 OsFTIP1
-general settings-
upper-z: 5000
lower-z: 1
stroke-width: 1.0
ignore z
filled
-specific settings-
use rectangles
diameter: 17
color:
show
list
settings
Sheet1
Annotated
Sheet3
Color scheme
Type here to search
65°F
ENG
5:14 PM
11/17/2023

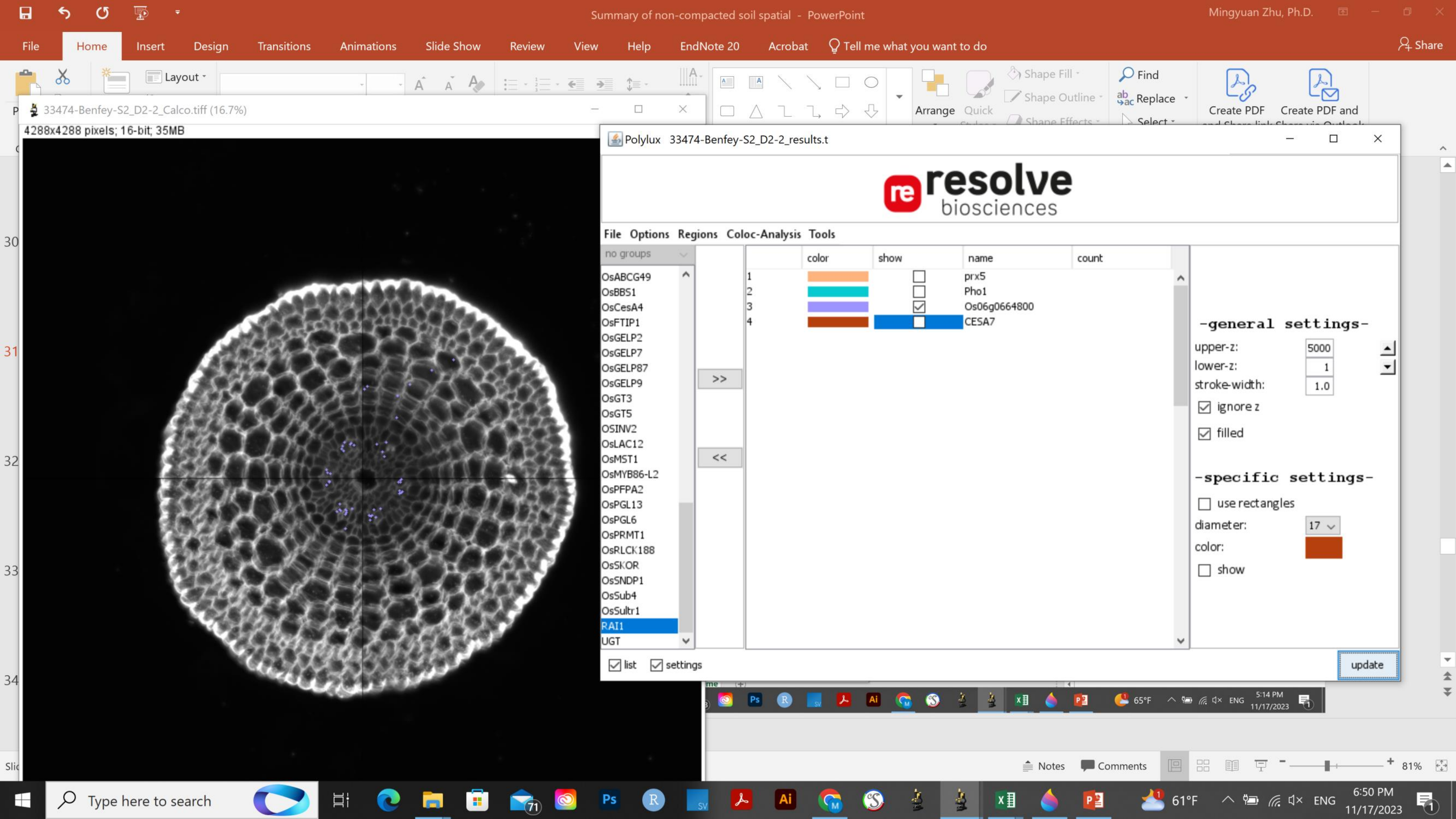

Summary of non-compacted soil spatial - PowerPoint
Mingyuan Zhu, Ph.D.
File
Home
Insert
Design
Transitions
Animations
Slide Show
Review
View
Help
EndNote 20
Acrobat
Tell me what you want to do
Share
Layout
Shape Fill
Shape Outline
Shape Effects
Arrange
Quick Styles
Find
Replace
Select
Create PDF
Create PDF and
33474-Benfey-S2_D2-2_Calco.tiff (16.7%)
4288x4288 pixels; 16-bit; 35MB
Polylux 33474-Benfey-S2_D2-2_results.t
resolve
biosciences
File Options Regions Coloc-Analysis Tools
no groups
OsABCG49
OsBBS1
OsCesA4
OsFTIP1
OsGELP2
OsGELP7
OsGELP87
OsGELP9
OsGT3
OsGT5
OSINV2
OsLAC12
OsMST1
OsMYB86-L2
OsPFPA2
OsPGL13
OsPGL6
OsPRMT1
OsRLCK188
OsSKOR
OsSNDP1
OsSub4
OsSultr1
RAI1
UGT
>>
<<
color
show
name
count
1 prx5
2 Pho1
3 Os06g0664800
4 CESA7
-general settings-
upper-z: 5000
lower-z: 1
stroke-width: 1.0
ignore z
filled
-specific settings-
use rectangles
diameter: 17
color:
show
list
settings
update
30
31
32
33
34
Notes
Comments
81%
Type here to search
65°F
ENG
5:14 PM
11/17/2023
61°F
6:50 PM
11/17/2023

Xylem

Untitled* - Paint 3D

Menu | Brushes | 2D shapes | 3D shapes | Stickers | Text | Effects | Canvas | 3D library | Paste | Undo | History | Redo

reality | 3D view | 71% | Brushes

33474-Benfey-S2_B2-1_Calco.tiff (16.7%)

4288x4288 pixels; 16-bit; 35MB

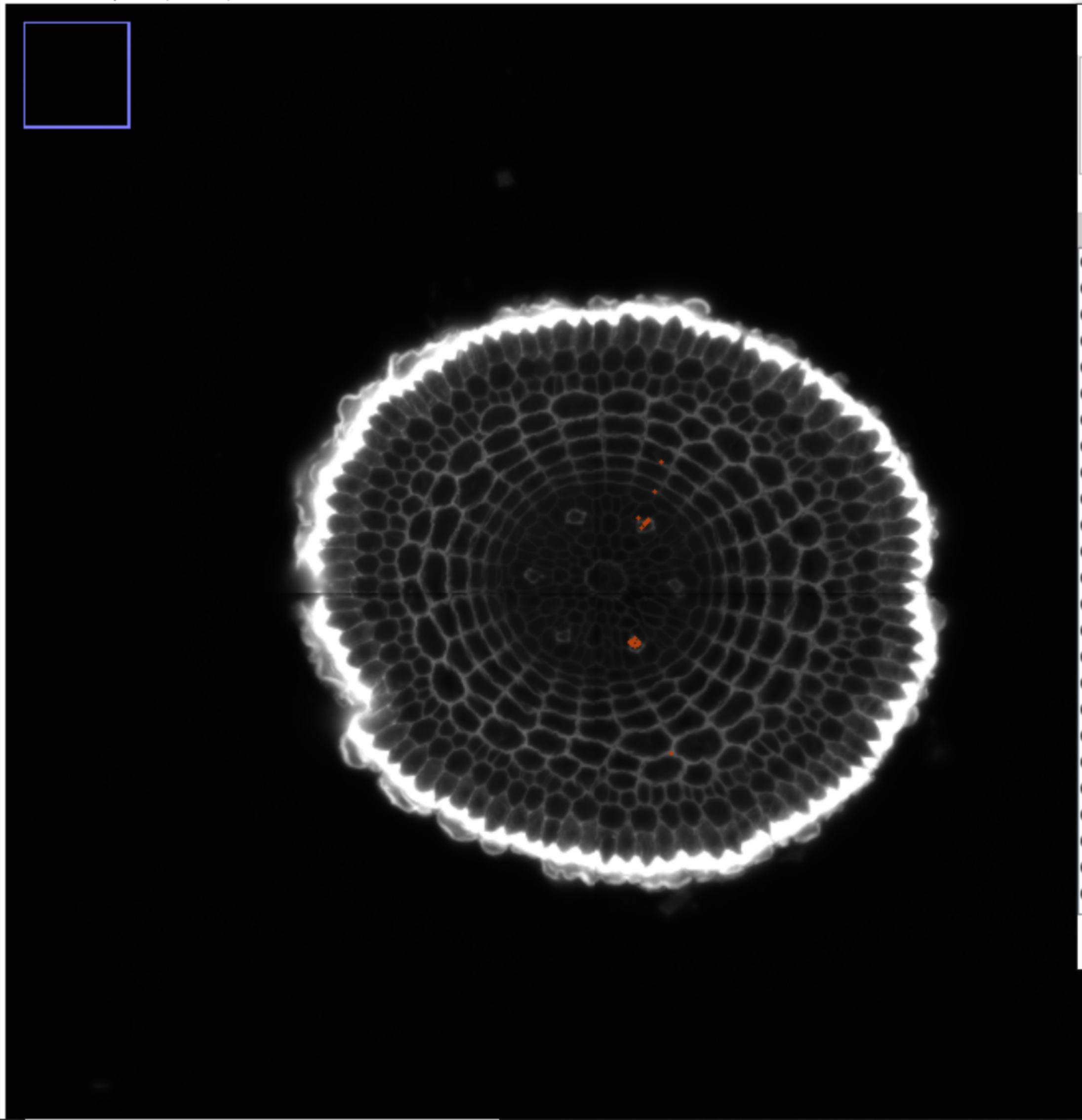

Polylux 33474-Benfey-S2_B2-1_results.t

resolve biosciences

File Options Regions Coloc-Analysis Tools

no groups

Os03g0115700
Os03g0134800
Os03g0185700
Os03g0314500
Os03g0570800
Os04g0125700
Os04g0585100
Os05g0153300
Os05g0398800
Os06g0135900
Os06g0611100
Os06g0695700
Os07g0560300
Os07g0634400
Os08g0138600
Os08g0554100
Os09g0535400
Os10g0155100
Os10g0459300
Os10g0469900
OsAAH
OsAAP11G
OsABCG49
OsBBS1
OsCesA4

>>

<<

	color	show	name	count
1		☑	ONAC073	
2		☐	Os06g0664800	

-general settings-

upper-z: 5000

lower-z: 1

stroke-width: 1.0

☑ ignore z

☑ filled

-specific settings-

☐ use rectangles

diameter: 17

color:

☐ show

☑ list ☑ settings

update

+ Add color

Type here to search

68

62°F ENG 6:09 PM 11/17/2023
