# Supplementary material for: Single-cell transcriptomics reveal how root tissues adapt to soil stress
Source: Nature. 2025 Apr 30;642(8068):721–9. doi: 10.1038/s41586-025-08941-z (PMC12176638; doi:10.1038/s41586-025-08941-z)
Supplement: Supplementary file 23 — Expression patterns of cell type markers in spatial transcriptomics data for compacted soil grown roots. A PDF summary file that includes the sample and gene information for visualization is included. The raw spatial transcriptomics data for compacted soil grown roots is also included. [file 41586_2025_8941_MOESM23_ESM.zip › Supplementary Data 8_Marker_expression_in_compacted-soils-based_Spatial_transcriptomics_Rice/Summary of spatial-compacted soils.pdf]

Combined

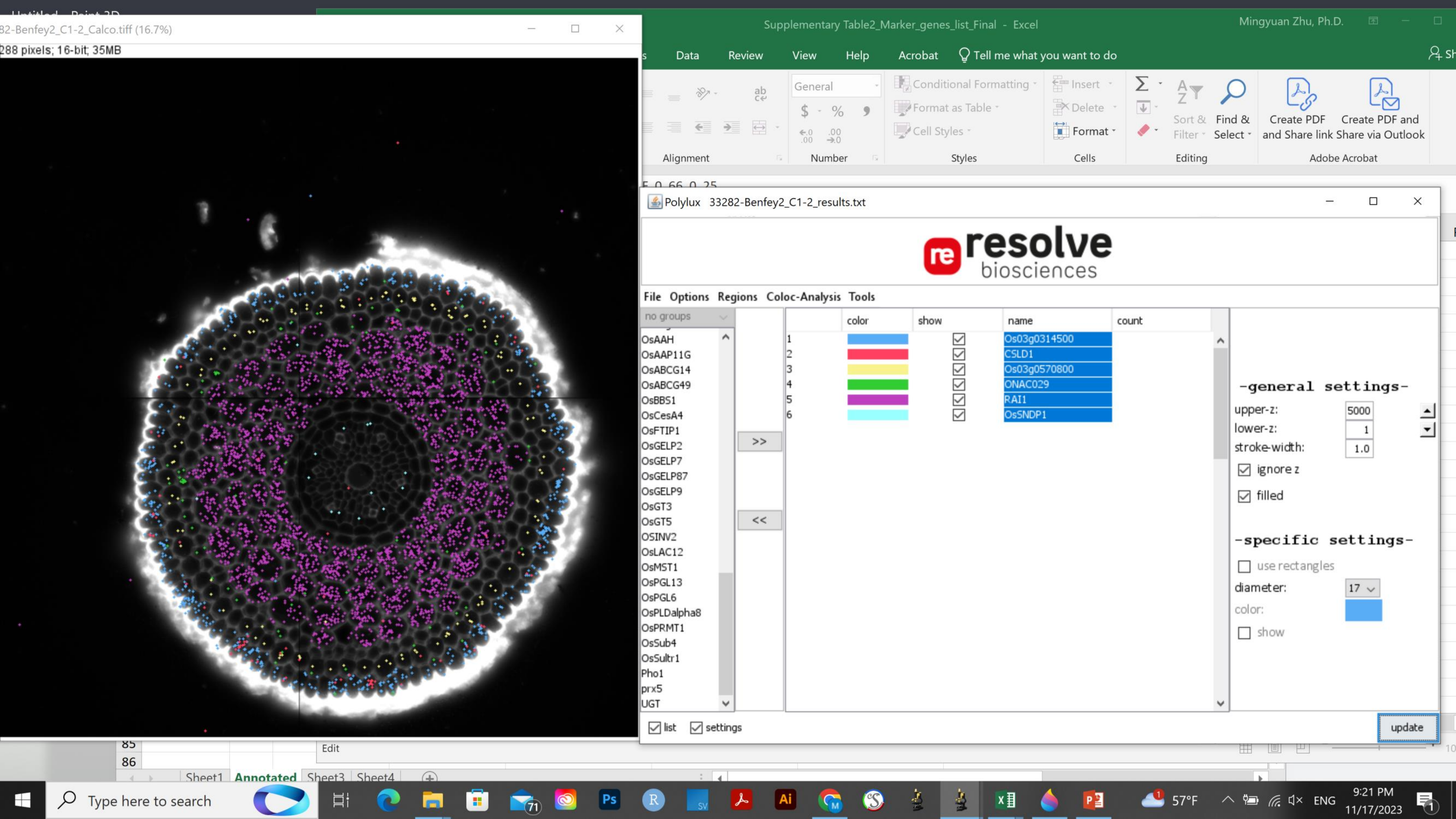
82-Benfey2_C1-2_Calco.tiff (16.7%)
288 pixels; 16-bit; 35MB
Supplementary Table2_Marker_genes_list_Final - Excel
Mingyuan Zhu, Ph.D.
Data
Review
View
Help
Acrobat
Tell me what you want to do
General
Conditional Formatting
Format as Table
Cell Styles
Insert
Delete
Format
Sort & Filter
Find & Select
Create PDF and Share link
Create PDF and Share via Outlook
Alignment
Number
Styles
Cells
Editing
Adobe Acrobat
Polylux 33282-Benfey2_C1-2_results.txt
resolve biosciences
File
Options
Regions
Coloc-Analysis
Tools
no groups
OsAAH
OsAAP11G
OsABCG14
OsABCG49
OsBBS1
OsCesA4
OsFTIP1
OsGELP2
OsGELP7
OsGELP87
OsGELP9
OsGT3
OsGT5
OSINV2
OsLAC12
OsMST1
OsPGL13
OsPGL6
OsPLDalpha8
OsPRMT1
OsSub4
OsSultr1
Pho1
prx5
UGT
color
show
name
count
1 Os03g0314500
2 CSLD1
3 Os03g0570800
4 ONAC029
5 RAI1
6 OsSNDP1
-general settings-
upper-z: 5000
lower-z: 1
stroke-width: 1.0
ignore z
filled
-specific settings-
use rectangles
diameter: 17
color:
show
list
settings
update
Sheet1
Annotated
Sheet3
Sheet4
Type here to search
57°F
ENG
9:21 PM
11/17/2023

Atrichoblast

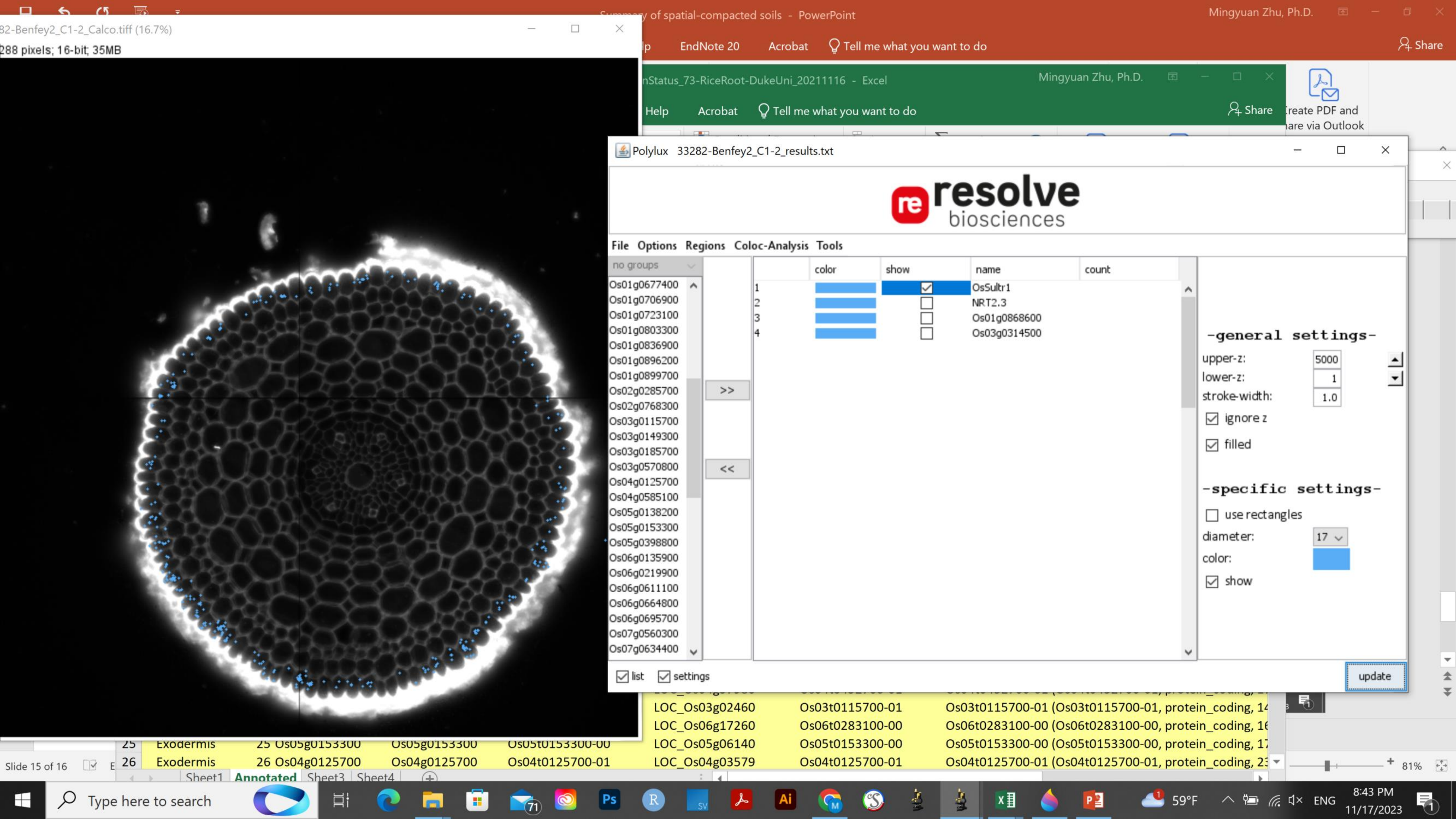
Summary of spatial-compacted soils - PowerPoint
Mingyuan Zhu, Ph.D.
EndNote 20
Acrobat
Tell me what you want to do
Share
32-Benfey2_C1-2_Calco.tiff (16.7%)
288 pixels; 16-bit; 35MB
nStatus_73-RiceRoot-DukeUni_20211116 - Excel
Help
Create PDF and Share via Outlook
Polylux 33282-Benfey2_C1-2_results.txt
resolve biosciences
File Options Regions Coloc-Analysis Tools
no groups
Os01g0677400
Os01g0706900
Os01g0723100
Os01g0803300
Os01g0836900
Os01g0896200
Os01g0899700
Os02g0285700
Os02g0768300
Os03g0115700
Os03g0149300
Os03g0185700
Os03g0570800
Os04g0125700
Os04g0585100
Os05g0138200
Os05g0153300
Os05g0398800
Os06g0135900
Os06g0219900
Os06g0611100
Os06g0664800
Os06g0695700
Os07g0560300
Os07g0634400
>>
<<
color show name count
1 OsSultr1
2 NRT2.3
3 Os01g0868600
4 Os03g0314500
-general settings-
upper-z: 5000
lower-z: 1
stroke-width: 1.0
ignore z
filled
-specific settings-
use rectangles
diameter: 17
color:
show
list
settings
update
LOC_Os03g02460 Os03t0115700-01 Os03t0115700-01 (Os03t0115700-01, protein_coding, 14
LOC_Os06g17260 Os06t0283100-00 Os06t0283100-00 (Os06t0283100-00, protein_coding, 16
25 Exodermis 25 Os05g0153300 Os05g0153300 Os05t0153300-00 LOC_Os05g06140 Os05t0153300-00 Os05t0153300-00 (Os05t0153300-00, protein_coding, 17
26 Exodermis 26 Os04g0125700 Os04g0125700 Os04t0125700-01 LOC_Os04g03579 Os04t0125700-01 Os04t0125700-01 (Os04t0125700-01, protein_coding, 23
Sheet1 Annotated Sheet3 Sheet4
Slide 15 of 16
81%
Type here to search
59°F
ENG
8:43 PM
11/17/2023

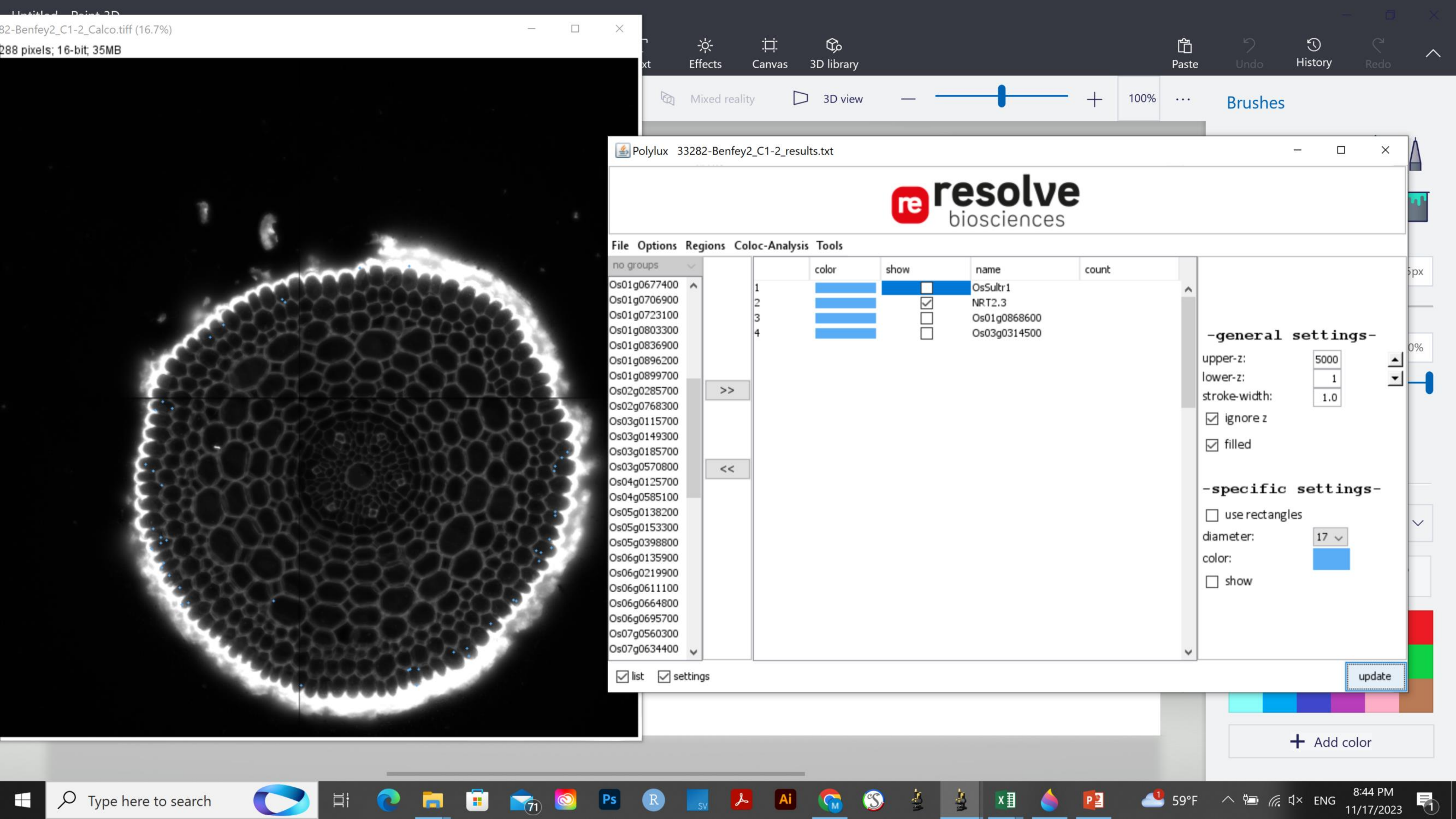

82-Benfey2_C1-2_Calco.tiff (16.7%)
288 pixels; 16-bit; 35MB
Effects
Canvas
3D library
Paste
Undo
History
Redo
Mixed reality
3D view
100%
Brushes
Polylux 33282-Benfey2_C1-2_results.txt
re resolve biosciences
File Options Regions Coloc-Analysis Tools
no groups
Os01g0677400
Os01g0706900
Os01g0723100
Os01g0803300
Os01g0836900
Os01g0896200
Os01g0899700
Os02g0285700
Os02g0768300
Os03g0115700
Os03g0149300
Os03g0185700
Os03g0570800
Os04g0125700
Os04g0585100
Os05g0138200
Os05g0153300
Os05g0398800
Os06g0135900
Os06g0219900
Os06g0611100
Os06g0664800
Os06g0695700
Os07g0560300
Os07g0634400
>>
<<
color
show
name
count
1 OsSultr1
2 NRT2.3
3 Os01g0868600
4 Os03g0314500
-general settings-
upper-z: 5000
lower-z: 1
stroke-width: 1.0
ignore z
filled
-specific settings-
use rectangles
diameter: 17
color:
show
list
settings
update
Add color
Type here to search
59°F
ENG
8:44 PM
11/17/2023

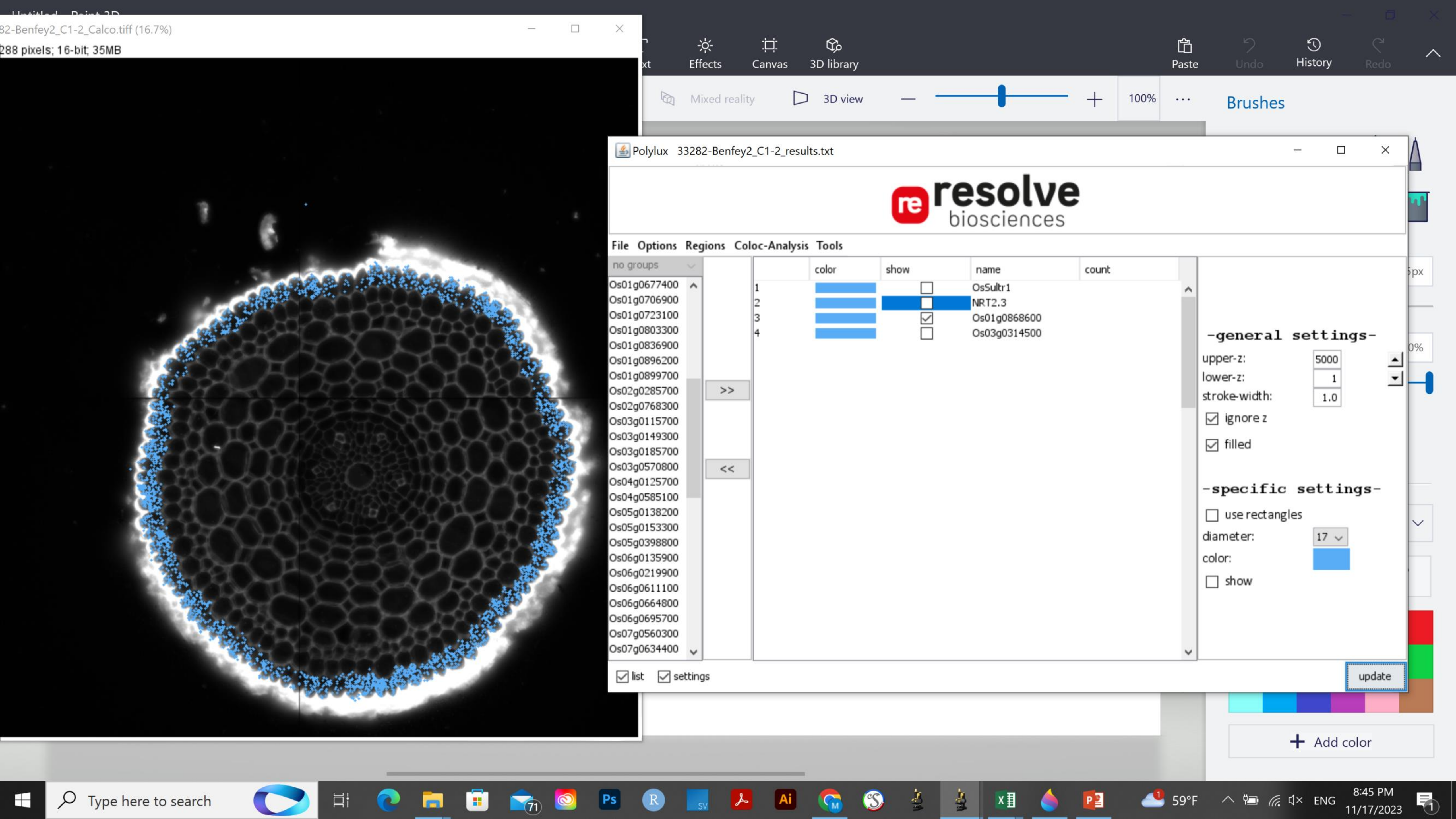
82-Benfey2_C1-2_Calco.tiff (16.7%)
288 pixels; 16-bit; 35MB
Effects
Canvas
3D library
Paste
Undo
History
Redo
Mixed reality
3D view
100%
Brushes
Polylux 33282-Benfey2_C1-2_results.txt
resolve biosciences
File Options Regions Coloc-Analysis Tools
no groups
Os01g0677400
Os01g0706900
Os01g0723100
Os01g0803300
Os01g0836900
Os01g0896200
Os01g0899700
Os02g0285700
Os02g0768300
Os03g0115700
Os03g0149300
Os03g0185700
Os03g0570800
Os04g0125700
Os04g0585100
Os05g0138200
Os05g0153300
Os05g0398800
Os06g0135900
Os06g0219900
Os06g0611100
Os06g0664800
Os06g0695700
Os07g0560300
Os07g0634400
>>
<<
color show name count
1 OsSultr1
2 NRT2.3
3 Os01g0868600
4 Os03g0314500
-general settings-
upper-z: 5000
lower-z: 1
stroke-width: 1.0
ignore z
filled
-specific settings-
use rectangles
diameter: 17
color:
show
list
settings
update
Add color
Type here to search
59°F
ENG
8:45 PM
11/17/2023

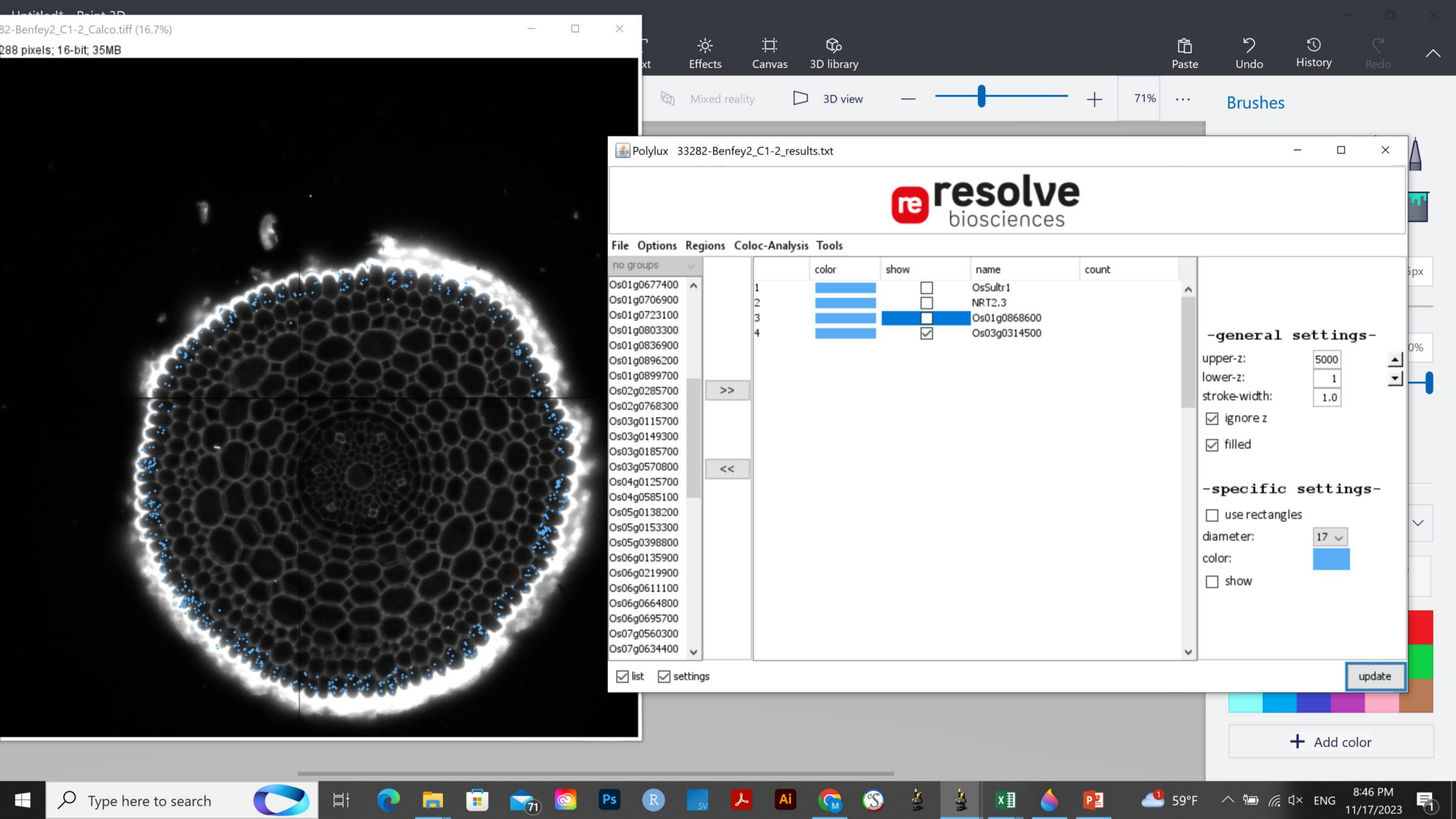

82-Benfey2_C1-2_Calco.tiff (16.7%)
288 pixels; 16-bit; 35MB
Effects
Canvas
3D library
Paste
Undo
History
Redo
Mixed reality
3D view
71%
Brushes
Polylux 33282-Benfey2_C1-2_results.txt
resolve biosciences
File Options Regions Coloc-Analysis Tools
no groups
Os01g0677400
Os01g0706900
Os01g0723100
Os01g0803300
Os01g0836900
Os01g0896200
Os01g0899700
Os02g0285700
Os02g0768300
Os03g0115700
Os03g0149300
Os03g0185700
Os03g0570800
Os04g0125700
Os04g0585100
Os05g0138200
Os05g0153300
Os05g0398800
Os06g0135900
Os06g0219900
Os06g0611100
Os06g0664800
Os06g0695700
Os07g0560300
Os07g0634400
>>
<<
color
show
name
count
1 OsSultr1
2 NRT2.3
3 Os01g0868600
4 Os03g0314500
-general settings-
upper-z: 5000
lower-z: 1
stroke-width: 1.0
ignore z
filled
-specific settings-
use rectangles
diameter: 17
color:
show
list
settings
update
Add color
Type here to search
59°F
ENG
8:46 PM
11/17/2023

Trichoblast

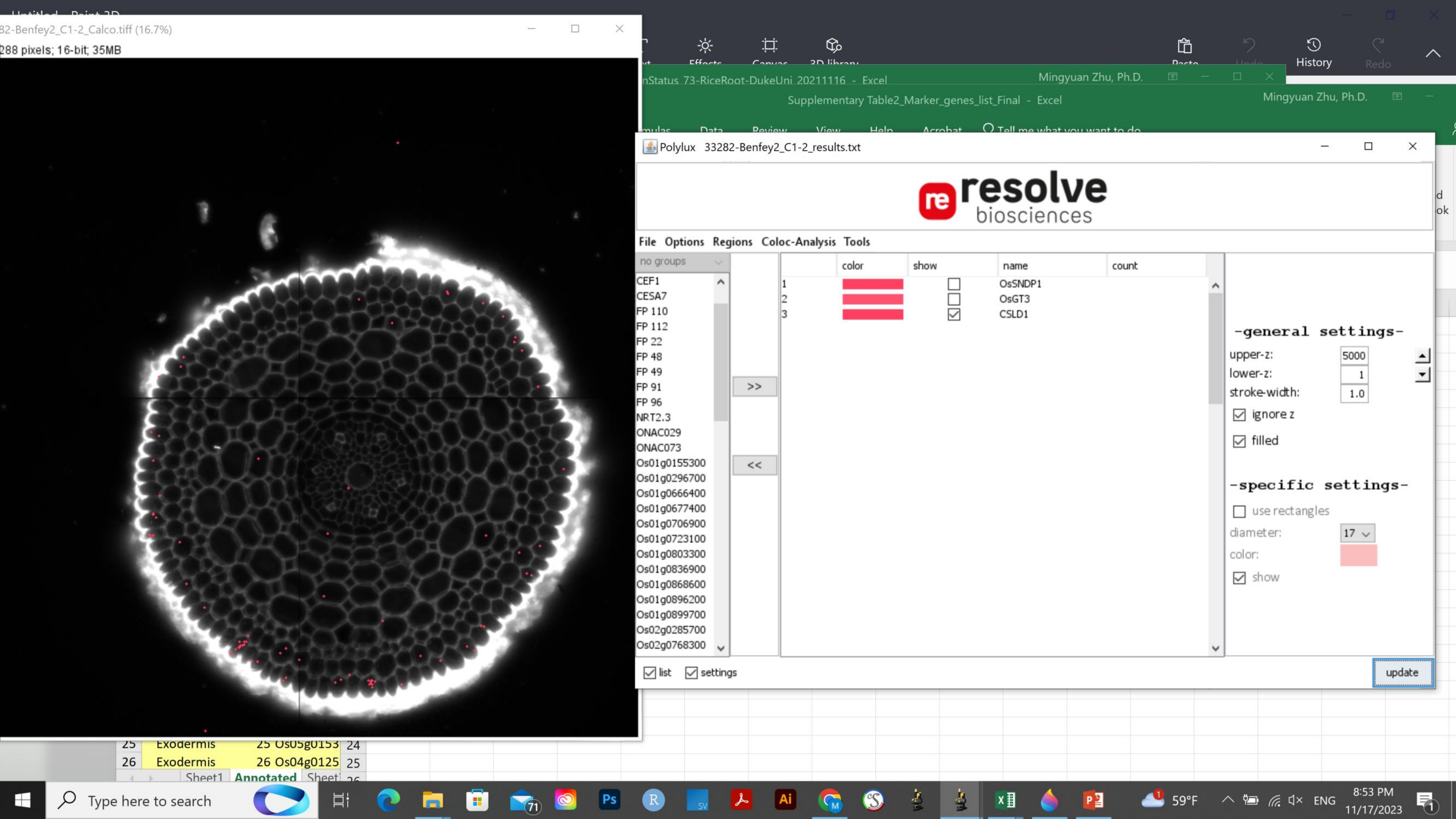
82-Benfey2_C1-2_Calco.tiff (16.7%)
288 pixels; 16-bit; 35MB
nStatus 73-RiceRoot-DukeUni 20211116 - Excel
Mingyuan Zhu, Ph.D.
Supplementary Table2_Marker_genes_list_Final - Excel
Mingyuan Zhu, Ph.D.
History
Polylux 33282-Benfey2_C1-2_results.txt
resolve biosciences
File Options Regions Coloc-Analysis Tools
no groups
CEF1
CESA7
FP 110
FP 112
FP 22
FP 48
FP 49
FP 91
FP 96
NRT2.3
ONAC029
ONAC073
Os01g0155300
Os01g0296700
Os01g0666400
Os01g0677400
Os01g0706900
Os01g0723100
Os01g0803300
Os01g0836900
Os01g0868600
Os01g0896200
Os01g0899700
Os02g0285700
Os02g0768300
>>
<<
color
show
name
count
1 OsSNDP1
2 OsGT3
3 CSLD1
-general settings-
upper-z: 5000
lower-z: 1
stroke-width: 1.0
ignore z
filled
-specific settings-
use rectangles
diameter: 17
color:
show
list
settings
update
25 Exodermis 25 Os05g0153 24
26 Exodermis 26 Os04g0125 25
Sheet1 Annotated
Type here to search
59°F
ENG
8:53 PM
11/17/2023

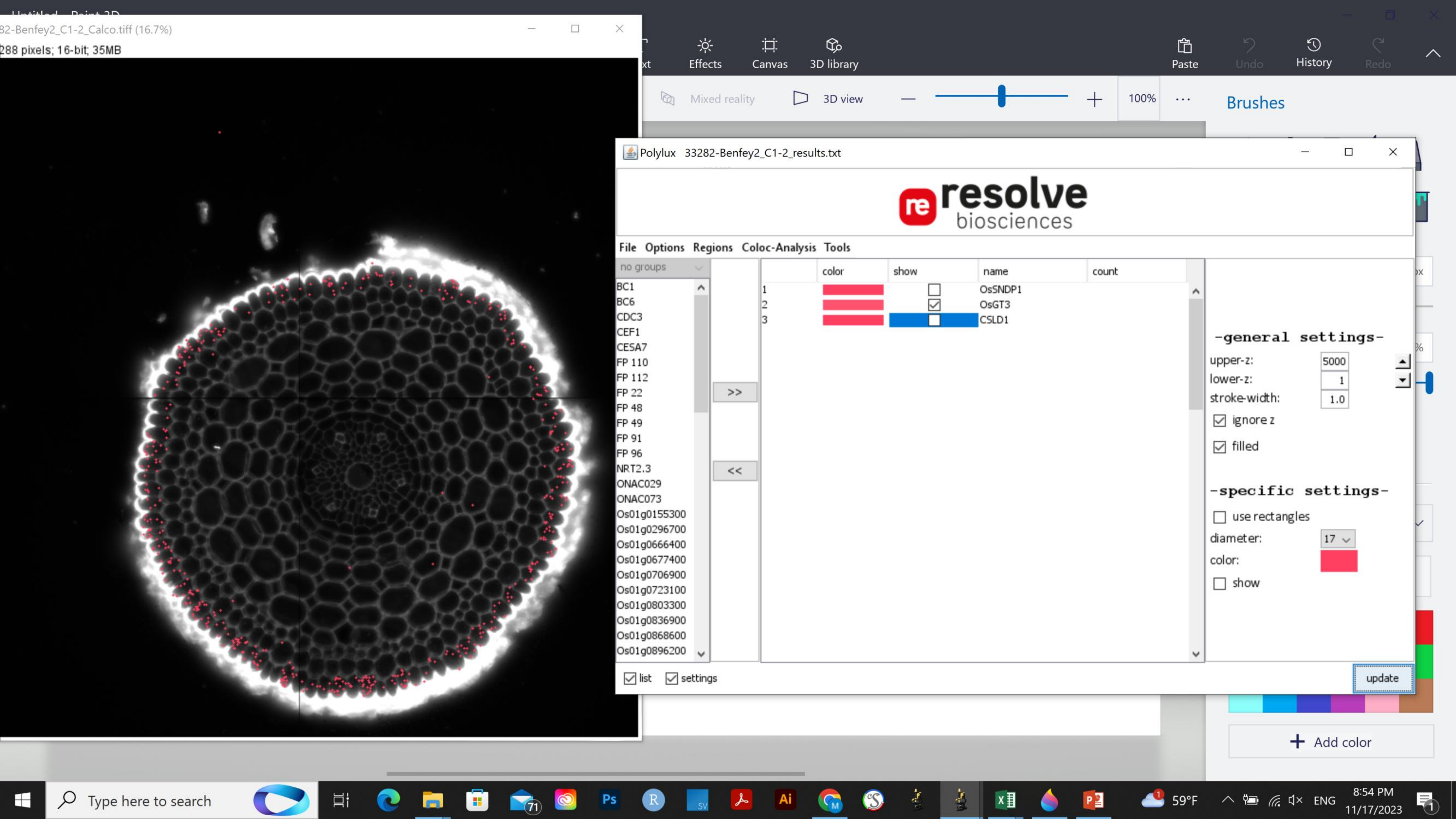
82-Benfey2_C1-2_Calco.tiff (16.7%)
288 pixels; 16-bit; 35MB
Effects
Canvas
3D library
Paste
Undo
History
Redo
Mixed reality
3D view
100%
Brushes
Polylux 33282-Benfey2_C1-2_results.txt
resolve
biosciences
File Options Regions Coloc-Analysis Tools
no groups
BC1
BC6
CDC3
CEF1
CESA7
FP 110
FP 112
FP 22
FP 48
FP 49
FP 91
FP 96
NRT2.3
ONAC029
ONAC073
Os01g0155300
Os01g0296700
Os01g0666400
Os01g0677400
Os01g0706900
Os01g0723100
Os01g0803300
Os01g0836900
Os01g0868600
Os01g0896200
color
show
name
count
1 OsSNDP1
2 OsGT3
3 CSLD1
-general settings-
upper-z: 5000
lower-z: 1
stroke-width: 1.0
ignore z
filled
-specific settings-
use rectangles
diameter: 17
color:
show
list
settings
update
Add color
Type here to search
59°F
ENG
8:54 PM
11/17/2023

Exodermis

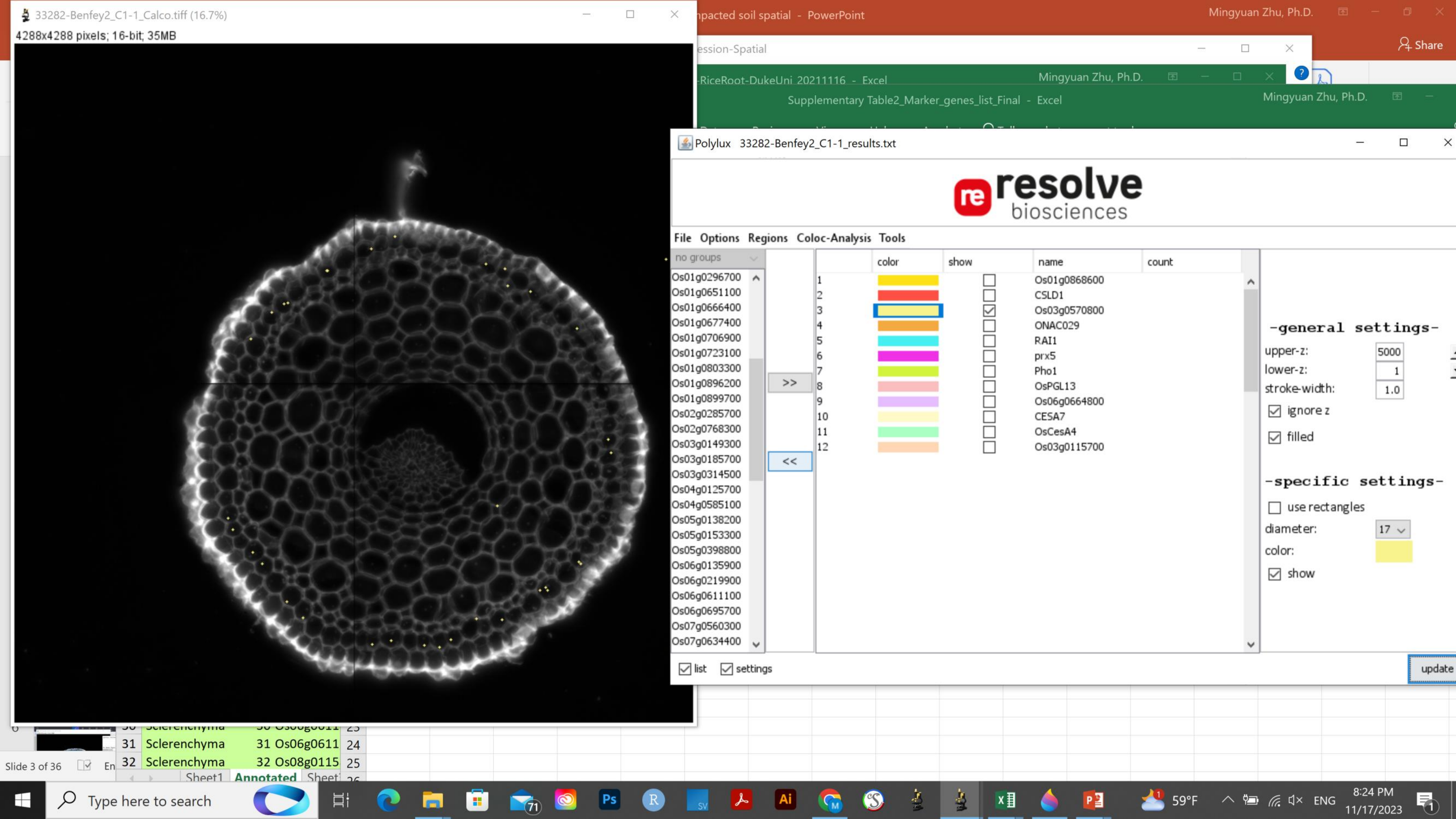
33282-Benfey2_C1-1_Calco.tiff (16.7%)
4288x4288 pixels; 16-bit; 35MB
Polylux 33282-Benfey2_C1-1_results.txt
resolve biosciences
File Options Regions Coloc-Analysis Tools
no groups
Os01g0296700
Os01g0651100
Os01g0666400
Os01g0677400
Os01g0706900
Os01g0723100
Os01g0803300
Os01g0896200
Os01g0899700
Os02g0285700
Os02g0768300
Os03g0149300
Os03g0185700
Os03g0314500
Os04g0125700
Os04g0585100
Os05g0138200
Os05g0153300
Os05g0398800
Os06g0135900
Os06g0219900
Os06g0611100
Os06g0695700
Os07g0560300
Os07g0634400
>>
<<
color show name count
1 Os01g0868600
2 CSLD1
3 Os03g0570800
4 ONAC029
5 RAI1
6 prx5
7 Pho1
8 OsPGL13
9 Os06g0664800
10 CESA7
11 OsCesA4
12 Os03g0115700
-general settings-
upper-z: 5000
lower-z: 1
stroke-width: 1.0
ignore z
filled
-specific settings-
use rectangles
diameter: 17
color:
show
list
settings
update
npacted soil spatial - PowerPoint
Mingyuan Zhu, Ph.D.
Share
ession-Spatial
-RiceRoot-DukeUni_20211116 - Excel
Supplementary Table2_Marker_genes_list_Final - Excel
31 Sclerenchyma 31 Os06g0611 24
32 Sclerenchyma 32 Os08g0115 25
Sheet1 Annotated
Slide 3 of 36
Type here to search
59°F
ENG
8:24 PM
11/17/2023

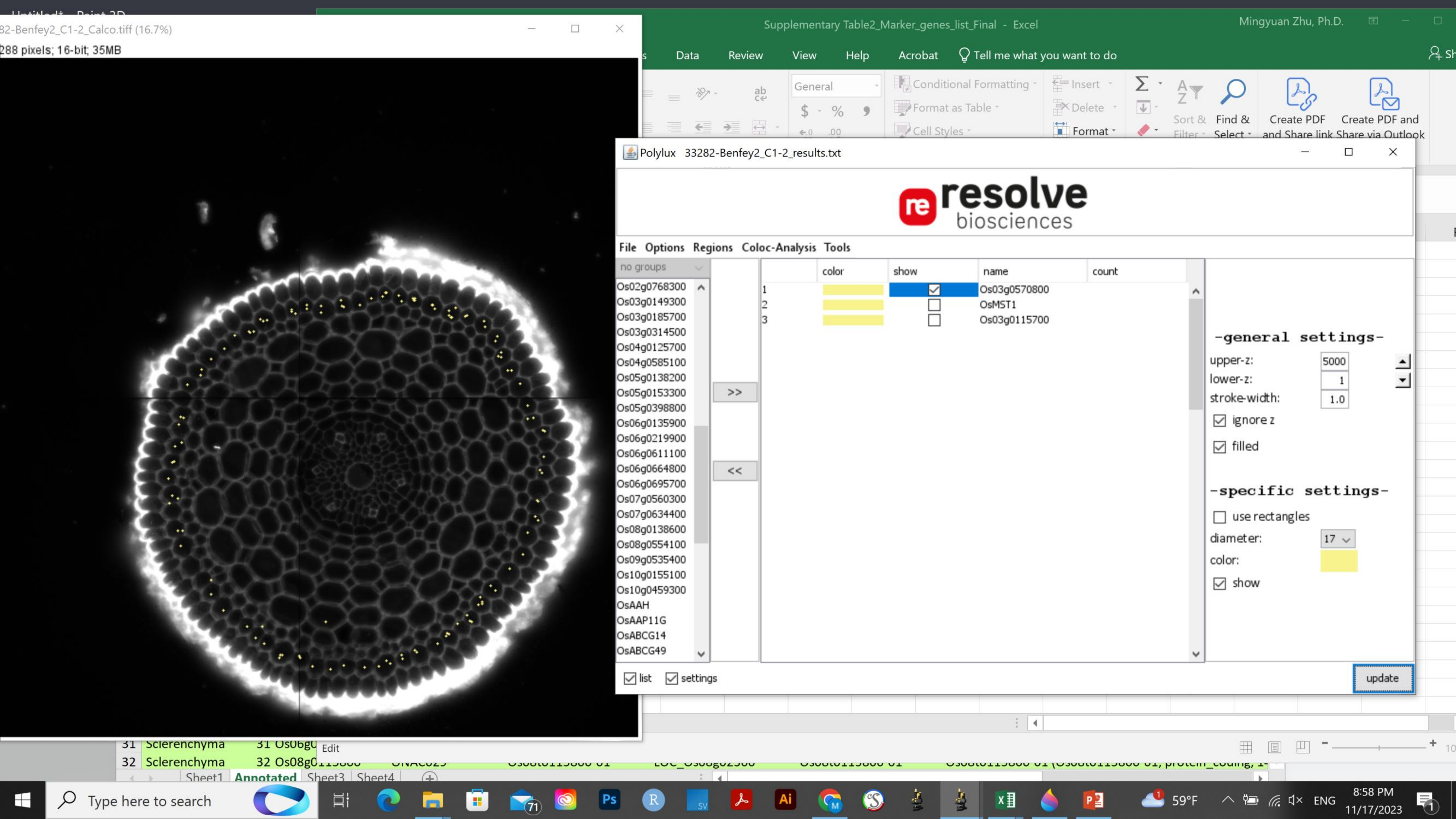
32-Benfey2_C1-2_Calco.tiff (16.7%)
288 pixels; 16-bit; 35MB
Supplementary Table2_Marker_genes_list_Final - Excel
Mingyuan Zhu, Ph.D.
Data
Review
View
Help
Acrobat
Tell me what you want to do
General
Conditional Formatting
Format as Table
Cell Styles
Insert
Delete
Format
Sort & Filter
Find & Select
Create PDF and Share link
Create PDF and Share via Outlook
Polylux 33282-Benfey2_C1-2_results.txt
resolve biosciences
File
Options
Regions
Coloc-Analysis
Tools
no groups
Os02g0768300
Os03g0149300
Os03g0185700
Os03g0314500
Os04g0125700
Os04g0585100
Os05g0138200
Os05g0153300
Os05g0398800
Os06g0135900
Os06g0219900
Os06g0611100
Os06g0664800
Os06g0695700
Os07g0560300
Os07g0634400
Os08g0138600
Os08g0554100
Os09g0535400
Os10g0155100
Os10g0459300
OsAAH
OsAAP11G
OsABCG14
OsABCG49
>>
<<
color
show
name
count
1 Os03g0570800
2 OsMST1
3 Os03g0115700
-general settings-
upper-z: 5000
lower-z: 1
stroke-width: 1.0
ignore z
filled
-specific settings-
use rectangles
diameter: 17
color:
show
list
settings
update
31 Sclerenchyma 31 Os06g0
32 Sclerenchyma 32 Os08g0
Edit
Sheet1
Annotated
Sheet3
Sheet4
Type here to search
59°F
ENG
8:58 PM
11/17/2023

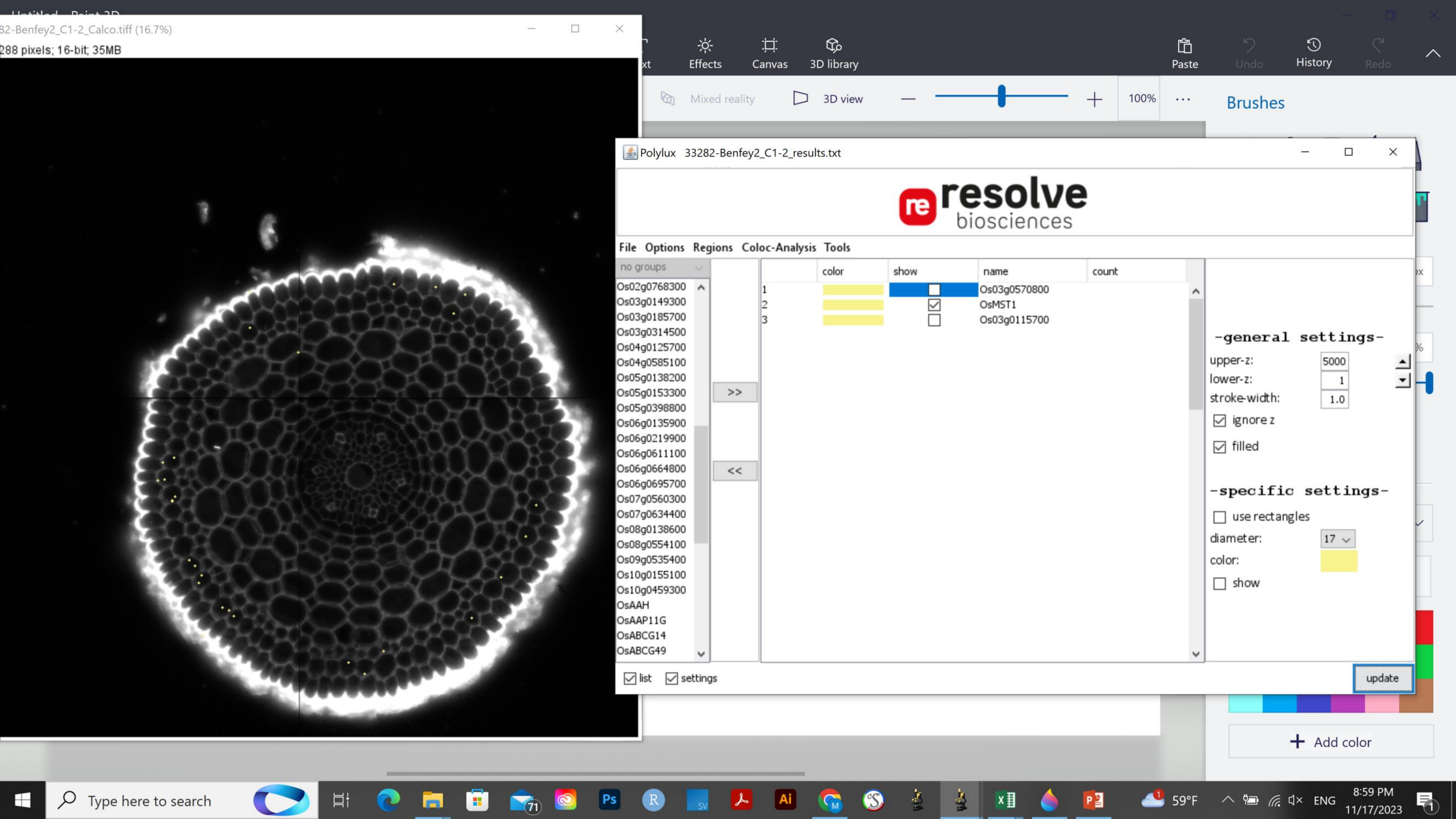
82-Benfey2_C1-2_Calco.tiff (16.7%)
288 pixels; 16-bit; 35MB
Effects
Canvas
3D library
Paste
Undo
History
Redo
Mixed reality
3D view
100%
Brushes
Polylux 33282-Benfey2_C1-2_results.txt
resolve biosciences
File Options Regions Coloc-Analysis Tools
no groups
Os02g0768300
Os03g0149300
Os03g0185700
Os03g0314500
Os04g0125700
Os04g0585100
Os05g0138200
Os05g0153300
Os05g0398800
Os06g0135900
Os06g0219900
Os06g0611100
Os06g0664800
Os06g0695700
Os07g0560300
Os07g0634400
Os08g0138600
Os08g0554100
Os09g0535400
Os10g0155100
Os10g0459300
OsAAH
OsAAP11G
OsABCG14
OsABCG49
>>
<<
color
show
name
count
1 Os03g0570800
2 OsMST1
3 Os03g0115700
-general settings-
upper-z: 5000
lower-z: 1
stroke-width: 1.0
ignore z
filled
-specific settings-
use rectangles
diameter: 17
color:
show
list
settings
update
Add color
Type here to search
59°F
ENG
8:59 PM
11/17/2023

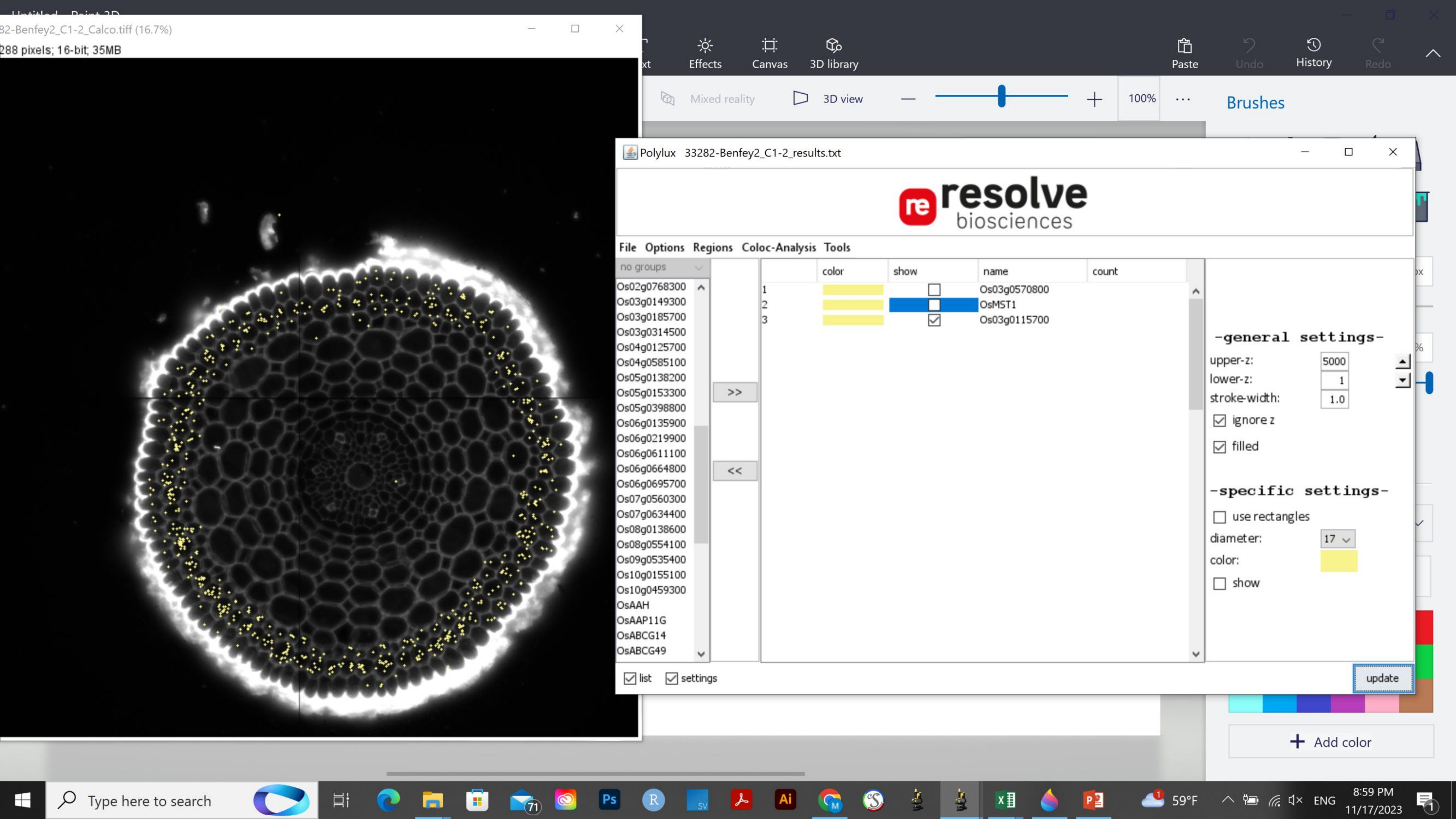
82-Benfey2_C1-2_Calco.tiff (16.7%)
288 pixels; 16-bit; 35MB
Effects
Canvas
3D library
Paste
Undo
History
Redo
Mixed reality
3D view
100%
Brushes
Polylux 33282-Benfey2_C1-2_results.txt
resolve biosciences
File Options Regions Coloc-Analysis Tools
no groups
Os02g0768300
Os03g0149300
Os03g0185700
Os03g0314500
Os04g0125700
Os04g0585100
Os05g0138200
Os05g0153300
Os05g0398800
Os06g0135900
Os06g0219900
Os06g0611100
Os06g0664800
Os06g0695700
Os07g0560300
Os07g0634400
Os08g0138600
Os08g0554100
Os09g0535400
Os10g0155100
Os10g0459300
OsAAH
OsAAP11G
OsABCG14
OsABCG49
>>
<<
color
show
name
count
1 Os03g0570800
2 OsMST1
3 Os03g0115700
-general settings-
upper-z: 5000
lower-z: 1
stroke-width: 1.0
ignore z
filled
-specific settings-
use rectangles
diameter: 17
color:
show
list
settings
update
Add color
Type here to search
59°F
ENG
8:59 PM
11/17/2023

Sclerenchyma

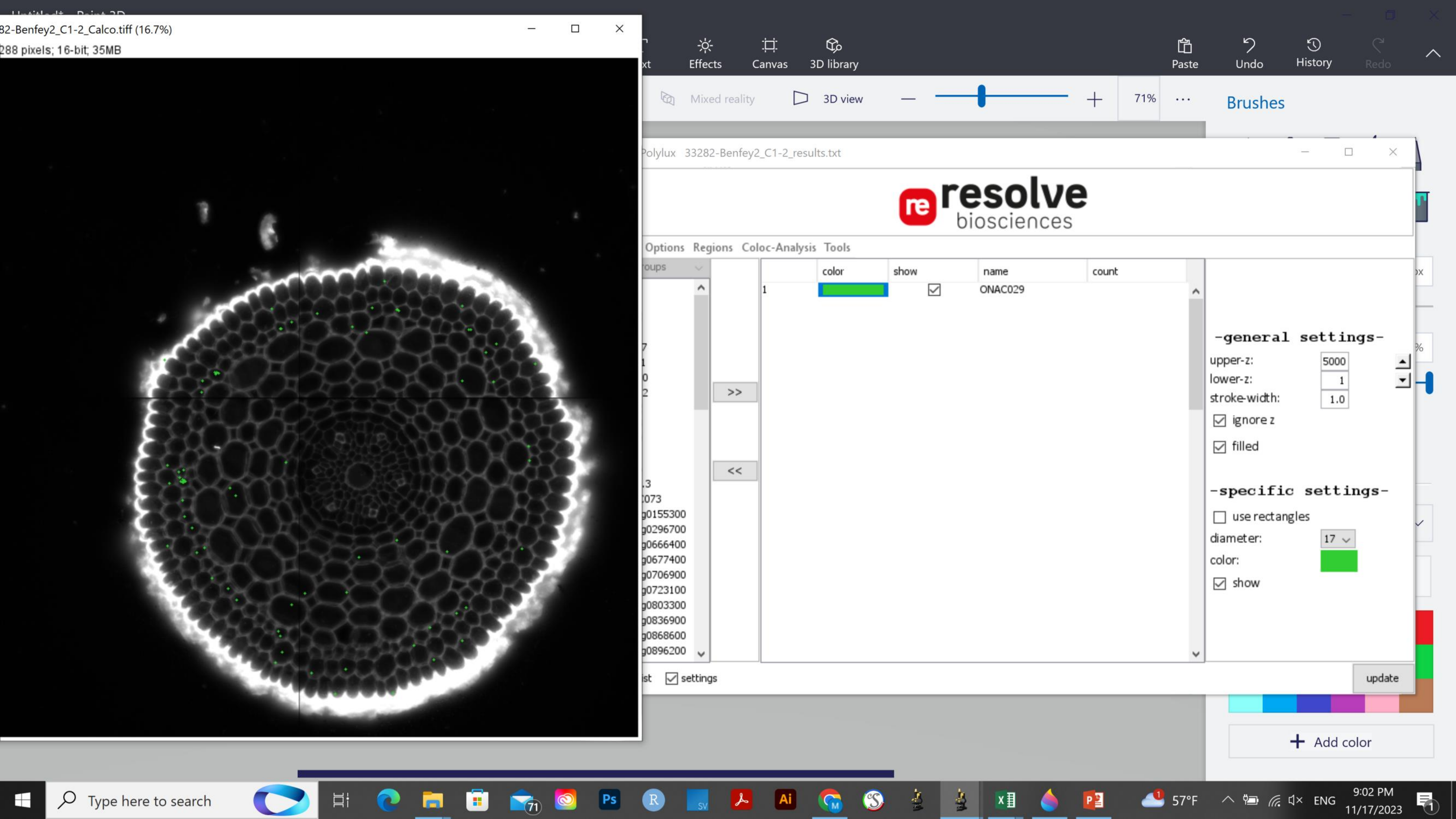

32-Benfey2_C1-2_Calco.tiff (16.7%)
288 pixels; 16-bit; 35MB
Effects
Canvas
3D library
Paste
Undo
History
Redo
Mixed reality
3D view
71%
Brushes
Polylux 33282-Benfey2_C1-2_results.txt
resolve
biosciences
Options
Regions
Coloc-Analysis
Tools
color
show
name
count
1
ONAC029
-general settings-
upper-z:
5000
lower-z:
1
stroke-width:
1.0
ignore z
filled
-specific settings-
use rectangles
diameter:
17
color:
show
settings
update
Add color
Type here to search
57°F
ENG
9:02 PM
11/17/2023

Cortex

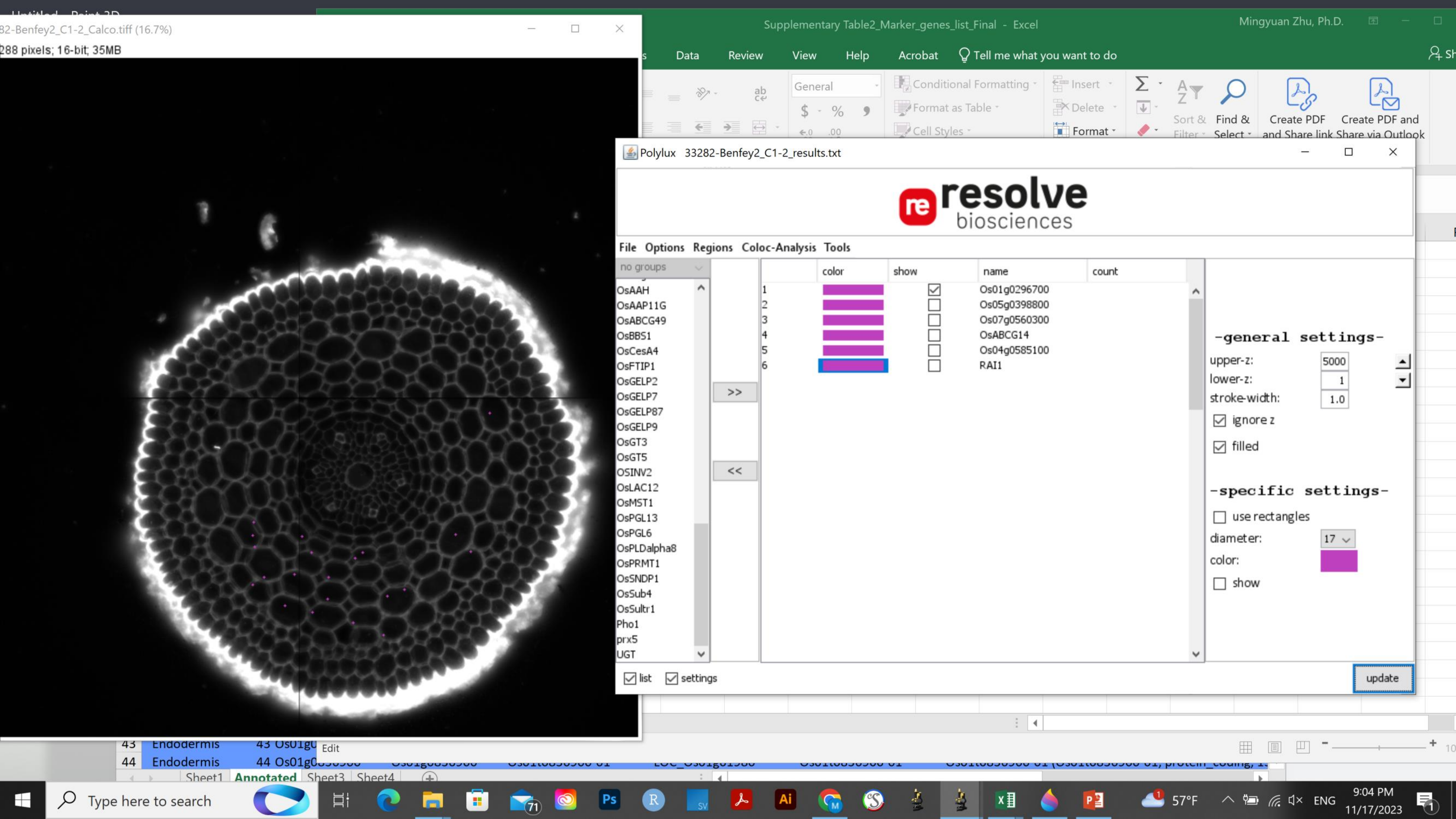
82-Benfey2_C1-2_Calco.tiff (16.7%)
288 pixels; 16-bit; 35MB
Supplementary Table2_Marker_genes_list_Final - Excel
Mingyuan Zhu, Ph.D.
Data
Review
View
Help
Acrobat
Tell me what you want to do
General
Conditional Formatting
Format as Table
Cell Styles
Insert
Delete
Format
Sort & Filter
Find & Select
Create PDF and Share link
Create PDF and Share via Outlook
Polylux 33282-Benfey2_C1-2_results.txt
resolve
biosciences
File
Options
Regions
Coloc-Analysis
Tools
no groups
OsAAH
OsAAP11G
OsABCG49
OsBBS1
OsCesA4
OsFTIP1
OsGELP2
OsGELP7
OsGELP87
OsGELP9
OsGT3
OsGT5
OSINV2
OsLAC12
OsMST1
OsPGL13
OsPGL6
OsPLDalpha8
OsPRMT1
OsSNDP1
OsSub4
OsSultr1
Pho1
prx5
UGT
>>
<<
color
show
name
count
1 Os01g0296700
2 Os05g0398800
3 Os07g0560300
4 OsABCG14
5 Os04g0585100
6 RAI1
-general settings-
upper-z: 5000
lower-z: 1
stroke-width: 1.0
ignore z
filled
-specific settings-
use rectangles
diameter: 17
color:
show
list
settings
update
43 Endodermis 43 Os01g0
44 Endodermis 44 Os01g0
Edit
Sheet1
Annotated
Sheet3
Sheet4
Type here to search
57°F
ENG
9:04 PM
11/17/2023

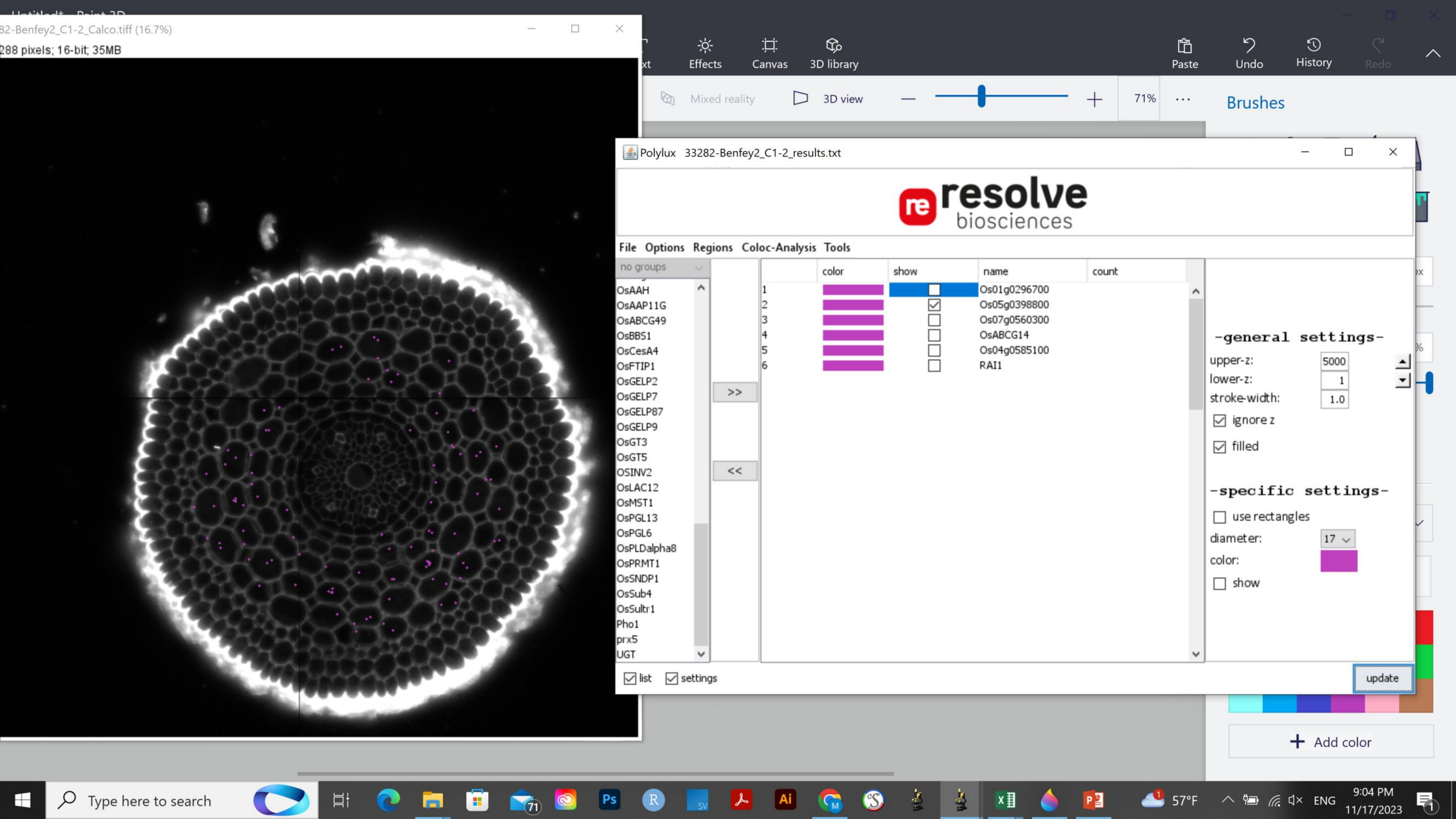
82-Benfey2_C1-2_Calco.tiff (16.7%)
288 pixels; 16-bit; 35MB
Effects
Canvas
3D library
Paste
Undo
History
Redo
Mixed reality
3D view
71%
Brushes
Polylux 33282-Benfey2_C1-2_results.txt
resolve biosciences
File Options Regions Coloc-Analysis Tools
no groups
OsAAH
OsAAP11G
OsABCG49
OsBBS1
OsCesA4
OsFTIP1
OsGELP2
OsGELP7
OsGELP87
OsGELP9
OsGT3
OsGT5
OSINV2
OsLAC12
OsMST1
OsPGL13
OsPGL6
OsPLDalpha8
OsPRMT1
OsSNDP1
OsSub4
OsSultr1
Pho1
prx5
UGT
>>
<<
color
show
name
count
1 Os01g0296700
2 Os05g0398800
3 Os07g0560300
4 OsABCG14
5 Os04g0585100
6 RAI1
-general settings-
upper-z: 5000
lower-z: 1
stroke-width: 1.0
ignore z
filled
-specific settings-
use rectangles
diameter: 17
color:
show
list
settings
update
Add color
Type here to search
57°F
ENG
9:04 PM
11/17/2023

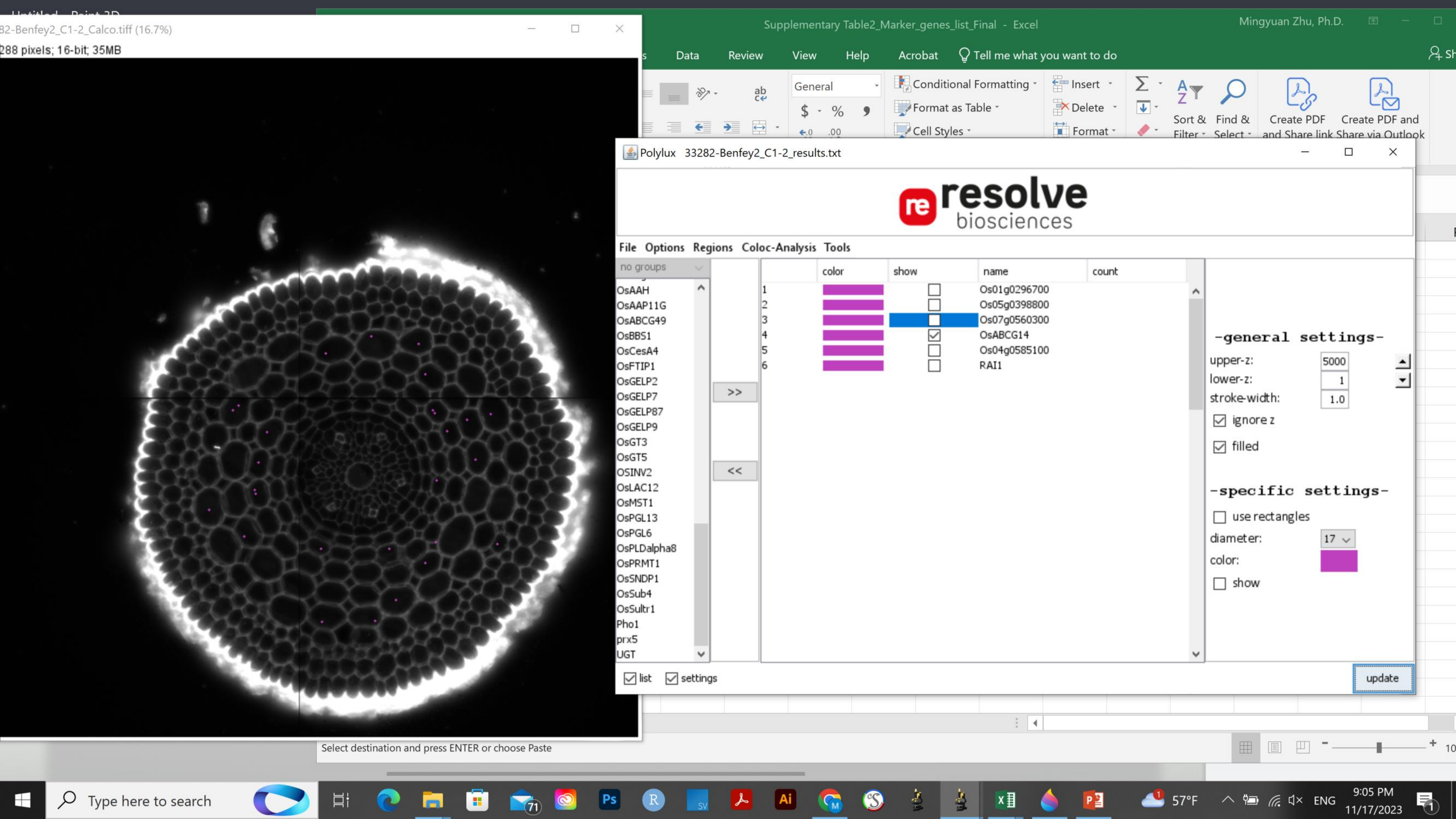
82-Benfey2_C1-2_Calco.tiff (16.7%)
288 pixels; 16-bit; 35MB
Supplementary Table2_Marker_genes_list_Final - Excel
Mingyuan Zhu, Ph.D.
Data
Review
View
Help
Acrobat
Tell me what you want to do
General
Conditional Formatting
Format as Table
Cell Styles
Insert
Delete
Format
Sort & Filter
Find & Select
Create PDF and Share link
Create PDF and Share via Outlook
Polylux 33282-Benfey2_C1-2_results.txt
resolve biosciences
File Options Regions Coloc-Analysis Tools
no groups
OsAAH
OsAAP11G
OsABCG49
OsBBS1
OsCesA4
OsFTIP1
OsGELP2
OsGELP7
OsGELP87
OsGELP9
OsGT3
OsGT5
OSINV2
OsLAC12
OsMST1
OsPGL13
OsPGL6
OsPLDalpha8
OsPRMT1
OsSNDP1
OsSub4
OsSultr1
Pho1
prx5
UGT
>>
<<
color
show
name
count
1 Os01g0296700
2 Os05g0398800
3 Os07g0560300
4 OsABCG14
5 Os04g0585100
6 RAI1
-general settings-
upper-z: 5000
lower-z: 1
stroke-width: 1.0
ignore z
filled
-specific settings-
use rectangles
diameter: 17
color:
show
list
settings
update
Select destination and press ENTER or choose Paste
Type here to search
57°F
ENG
9:05 PM
11/17/2023

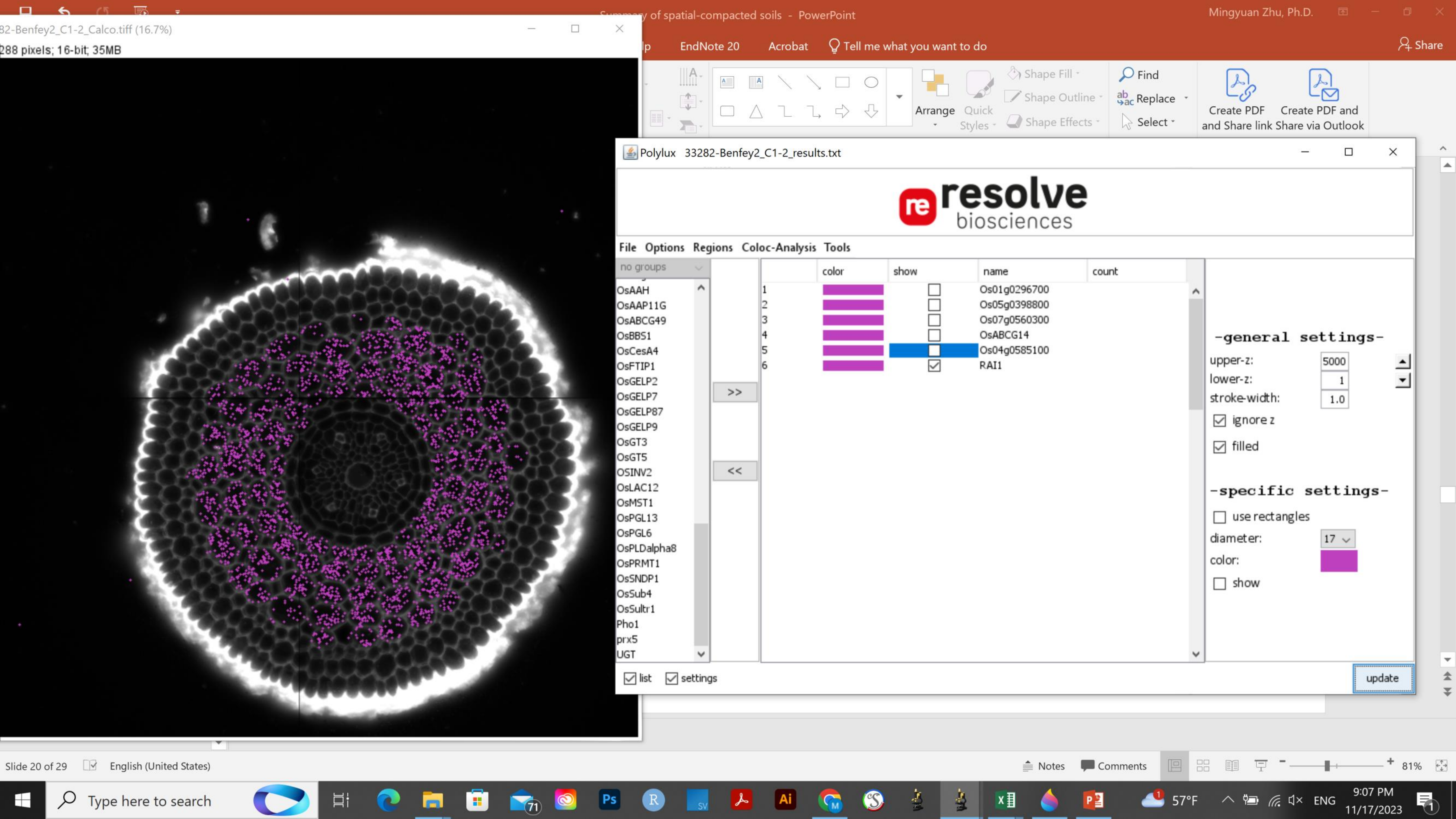
Summary of spatial-compacted soils - PowerPoint
Mingyuan Zhu, Ph.D.
82-Benfey2_C1-2_Calco.tiff (16.7%)
288 pixels; 16-bit; 35MB
EndNote 20
Acrobat
Tell me what you want to do
Share
Shape Fill
Shape Outline
Shape Effects
Arrange
Quick Styles
Find
Replace
Select
Create PDF and Share link
Create PDF and Share via Outlook
Polylux 33282-Benfey2_C1-2_results.txt
resolve biosciences
File Options Regions Coloc-Analysis Tools
no groups
OsAAH
OsAAP11G
OsABCG49
OsBBS1
OsCesA4
OsFTIP1
OsGELP2
OsGELP7
OsGELP87
OsGELP9
OsGT3
OsGT5
OSINV2
OsLAC12
OsMST1
OsPGL13
OsPGL6
OsPLDalpha8
OsPRMT1
OsSNDP1
OsSub4
OsSultr1
Pho1
prx5
UGT
>>
<<
color
show
name
count
1 Os01g0296700
2 Os05g0398800
3 Os07g0560300
4 OsABCG14
5 Os04g0585100
6 RAI1
-general settings-
upper-z: 5000
lower-z: 1
stroke-width: 1.0
ignore z
filled
-specific settings-
use rectangles
diameter: 17
color:
show
list
settings
update
Slide 20 of 29
English (United States)
Notes
Comments
81%
Type here to search
57°F
ENG
9:07 PM
11/17/2023

Endodermis

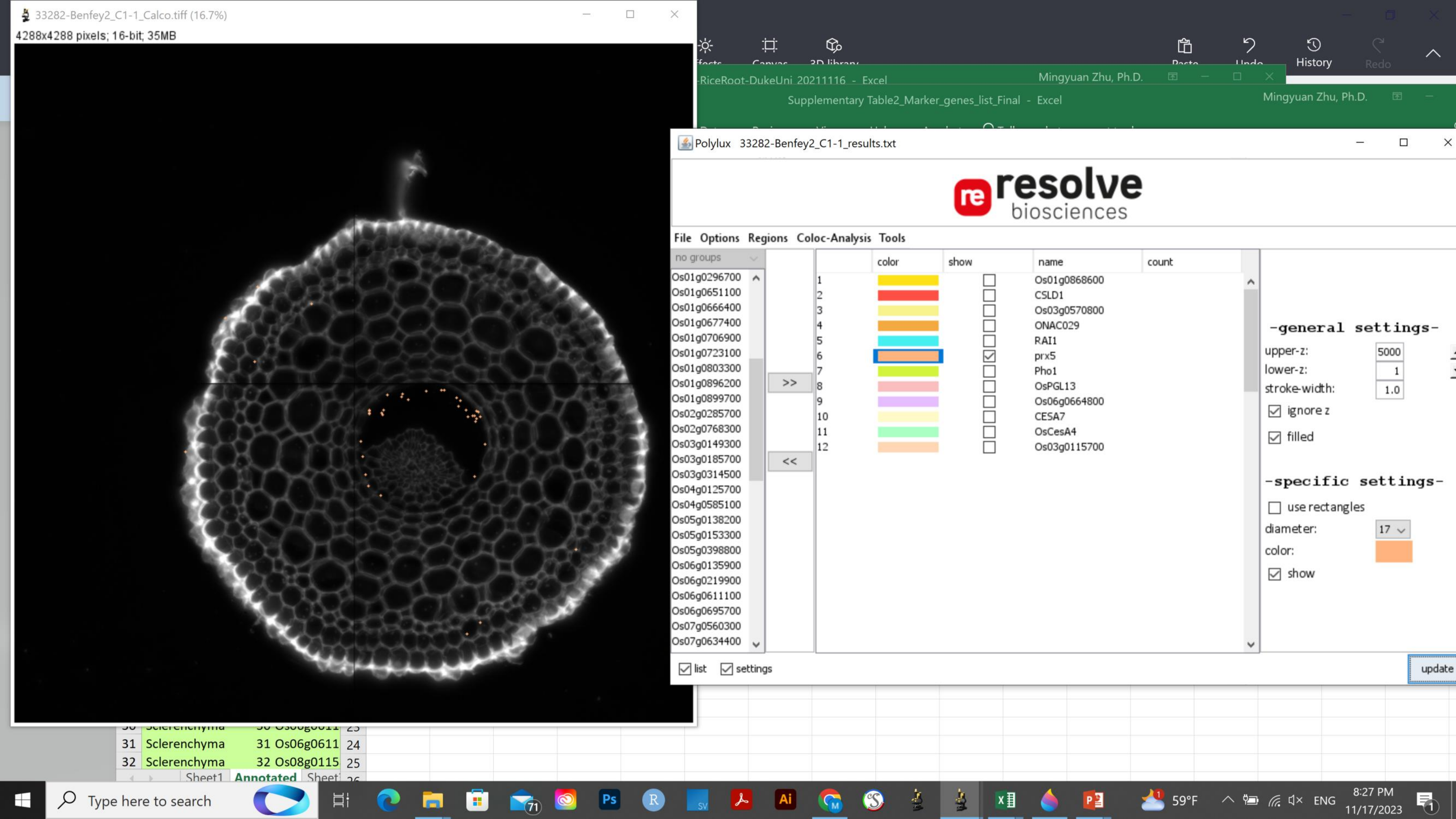
33282-Benfey2_C1-1_Calco.tiff (16.7%)
4288x4288 pixels; 16-bit; 35MB
History
Redo
RiceRoot-DukeUni_20211116 - Excel
Mingyuan Zhu, Ph.D.
Supplementary Table2_Marker_genes_list_Final - Excel
Mingyuan Zhu, Ph.D.
Polylux 33282-Benfey2_C1-1_results.txt
resolve
biosciences
File Options Regions Coloc-Analysis Tools
no groups
Os01g0296700
Os01g0651100
Os01g0666400
Os01g0677400
Os01g0706900
Os01g0723100
Os01g0803300
Os01g0896200
Os01g0899700
Os02g0285700
Os02g0768300
Os03g0149300
Os03g0185700
Os03g0314500
Os04g0125700
Os04g0585100
Os05g0138200
Os05g0153300
Os05g0398800
Os06g0135900
Os06g0219900
Os06g0611100
Os06g0695700
Os07g0560300
Os07g0634400
>>
<<
color show name count
1 Os01g0868600
2 CSLD1
3 Os03g0570800
4 ONAC029
5 RAI1
6 prx5
7 Pho1
8 OsPGL13
9 Os06g0664800
10 CESA7
11 OsCesA4
12 Os03g0115700
-general settings-
upper-z: 5000
lower-z: 1
stroke-width: 1.0
ignore z
filled
-specific settings-
use rectangles
diameter: 17
color:
show
list
settings
update
31 Sclerenchyma 31 Os06g0611 24
32 Sclerenchyma 32 Os08g0115 25
Sheet1 Annotated
Type here to search
59°F
ENG
8:27 PM
11/17/2023

Pericycle/Procambium

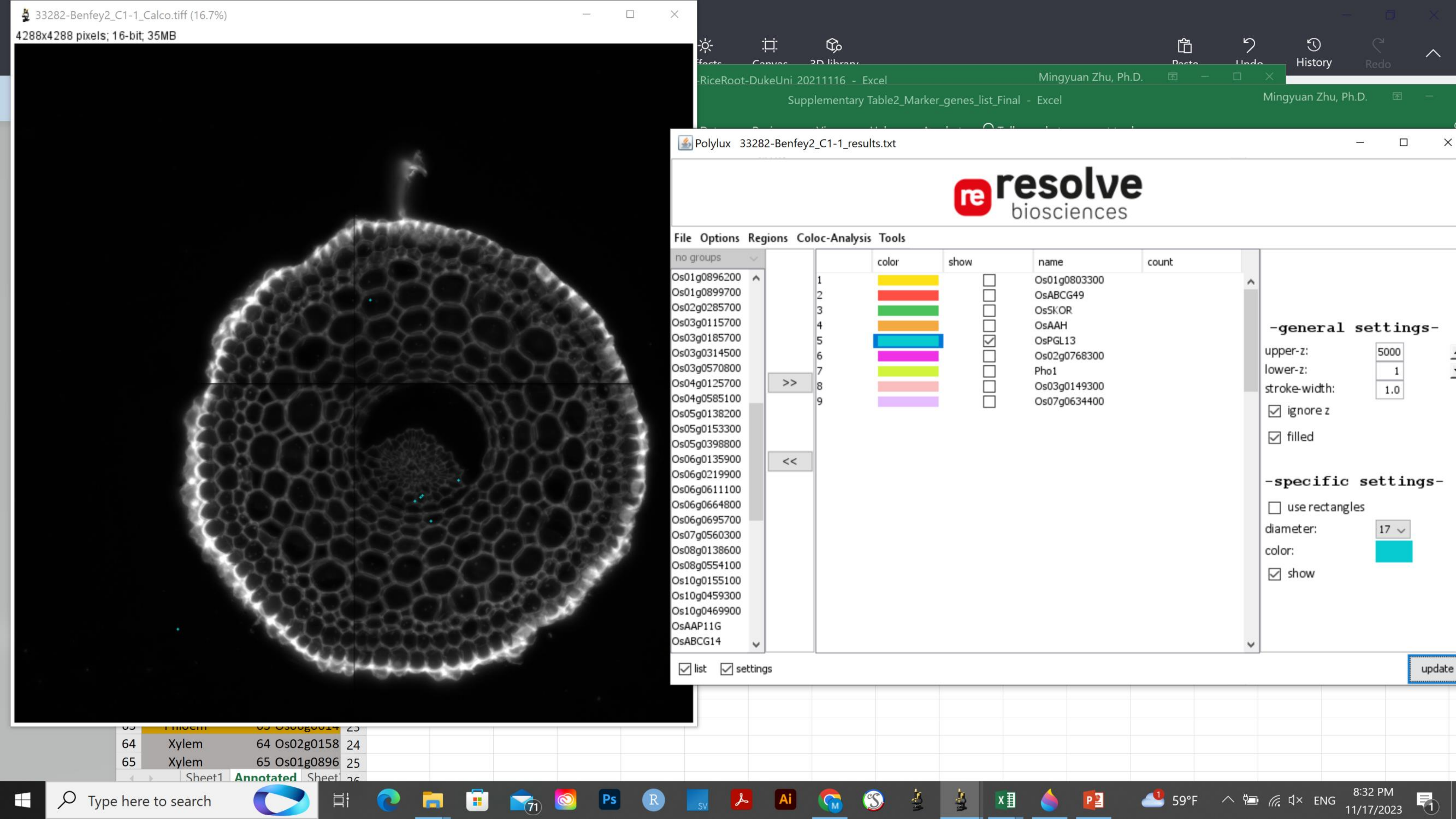
33282-Benfey2_C1-1_Calco.tiff (16.7%)
4288x4288 pixels; 16-bit; 35MB
RiceRoot-DukeUni 20211116 - Excel
Mingyuan Zhu, Ph.D.
Supplementary Table2_Marker_genes_list_Final - Excel
Mingyuan Zhu, Ph.D.
History
Redo
Polylux 33282-Benfey2_C1-1_results.txt
resolve
biosciences
File Options Regions Coloc-Analysis Tools
no groups
Os01g0896200
Os01g0899700
Os02g0285700
Os03g0115700
Os03g0185700
Os03g0314500
Os03g0570800
Os04g0125700
Os04g0585100
Os05g0138200
Os05g0153300
Os05g0398800
Os06g0135900
Os06g0219900
Os06g0611100
Os06g0664800
Os06g0695700
Os07g0560300
Os08g0138600
Os08g0554100
Os10g0155100
Os10g0459300
Os10g0469900
OsAAP11G
OsABCG14
>>
<<
color
show
name
count
1 Os01g0803300
2 OsABCG49
3 OsSKOR
4 OsAAH
5 OsPGL13
6 Os02g0768300
7 Pho1
8 Os03g0149300
9 Os07g0634400
-general settings-
upper-z: 5000
lower-z: 1
stroke-width: 1.0
ignore z
filled
-specific settings-
use rectangles
diameter: 17
color:
show
list
settings
update
64 Xylem 64 Os02g0158 24
65 Xylem 65 Os01g0896 25
Sheet1 Annotated
Type here to search
59°F
ENG
8:32 PM
11/17/2023

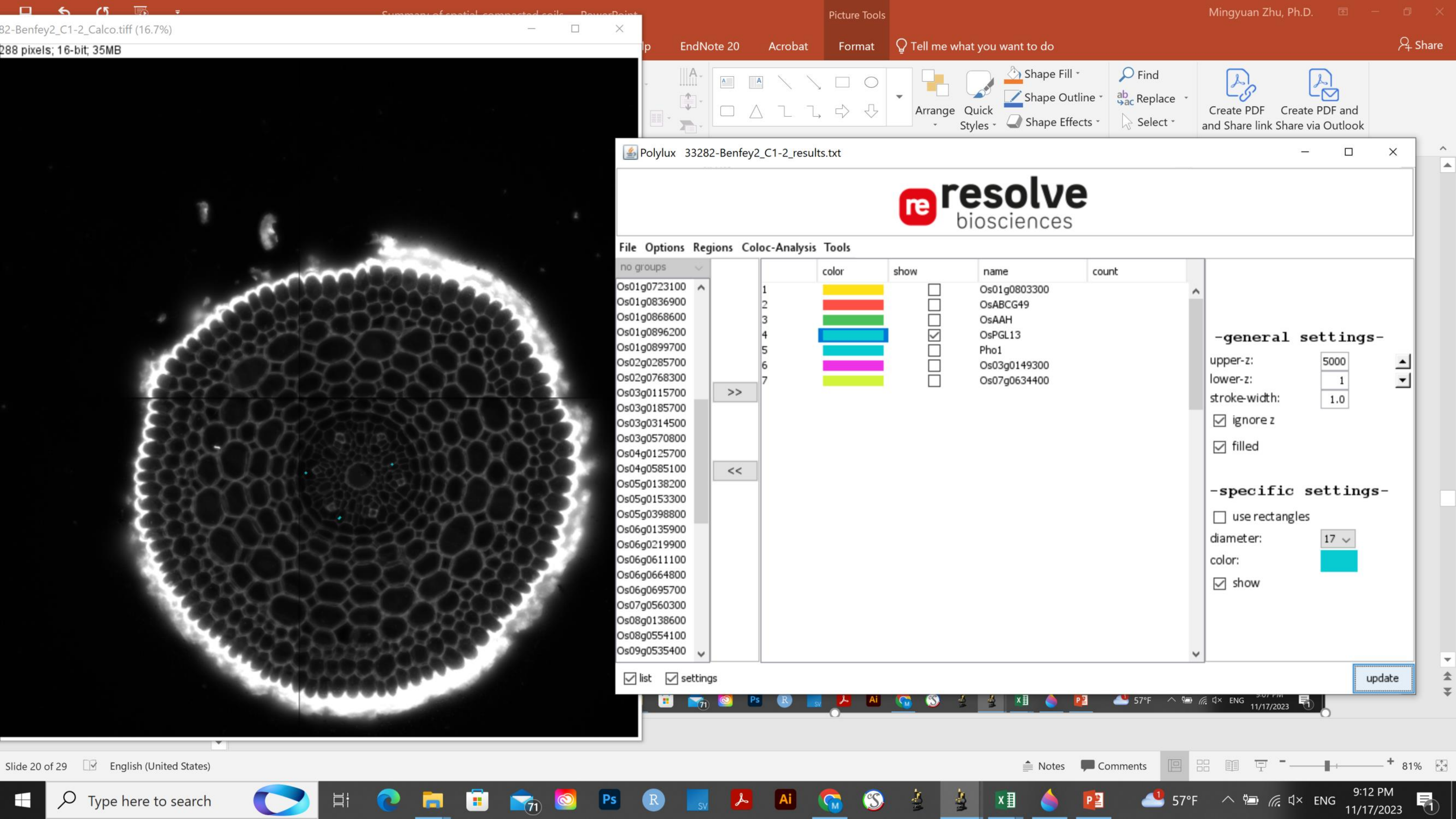

82-Benfey2_C1-2_Calco.tiff (16.7%)
288 pixels; 16-bit; 35MB
Polylux 33282-Benfey2_C1-2_results.txt
resolve biosciences
File Options Regions Coloc-Analysis Tools
no groups
Os01g0723100
Os01g0836900
Os01g0868600
Os01g0896200
Os01g0899700
Os02g0285700
Os02g0768300
Os03g0115700
Os03g0185700
Os03g0314500
Os03g0570800
Os04g0125700
Os04g0585100
Os05g0138200
Os05g0153300
Os05g0398800
Os06g0135900
Os06g0219900
Os06g0611100
Os06g0664800
Os06g0695700
Os07g0560300
Os08g0138600
Os08g0554100
Os09g0535400
>>
<<
color show name count
1 Os01g0803300
2 OsABCG49
3 OsAAH
4 OsPGL13
5 Pho1
6 Os03g0149300
7 Os07g0634400
-general settings-
upper-z: 5000
lower-z: 1
stroke-width: 1.0
ignore z
filled
-specific settings-
use rectangles
diameter: 17
color:
show
list settings
update
EndNote 20 Acrobat Format Tell me what you want to do
Picture Tools
Mingyuan Zhu, Ph.D.
Share
Shape Fill
Shape Outline
Shape Effects
Arrange
Quick Styles
Find
Replace
Select
Create PDF and Share link
Create PDF and Share via Outlook
Slide 20 of 29
English (United States)
Notes Comments 81%
Type here to search
57°F ENG
9:12 PM
11/17/2023

Phloem

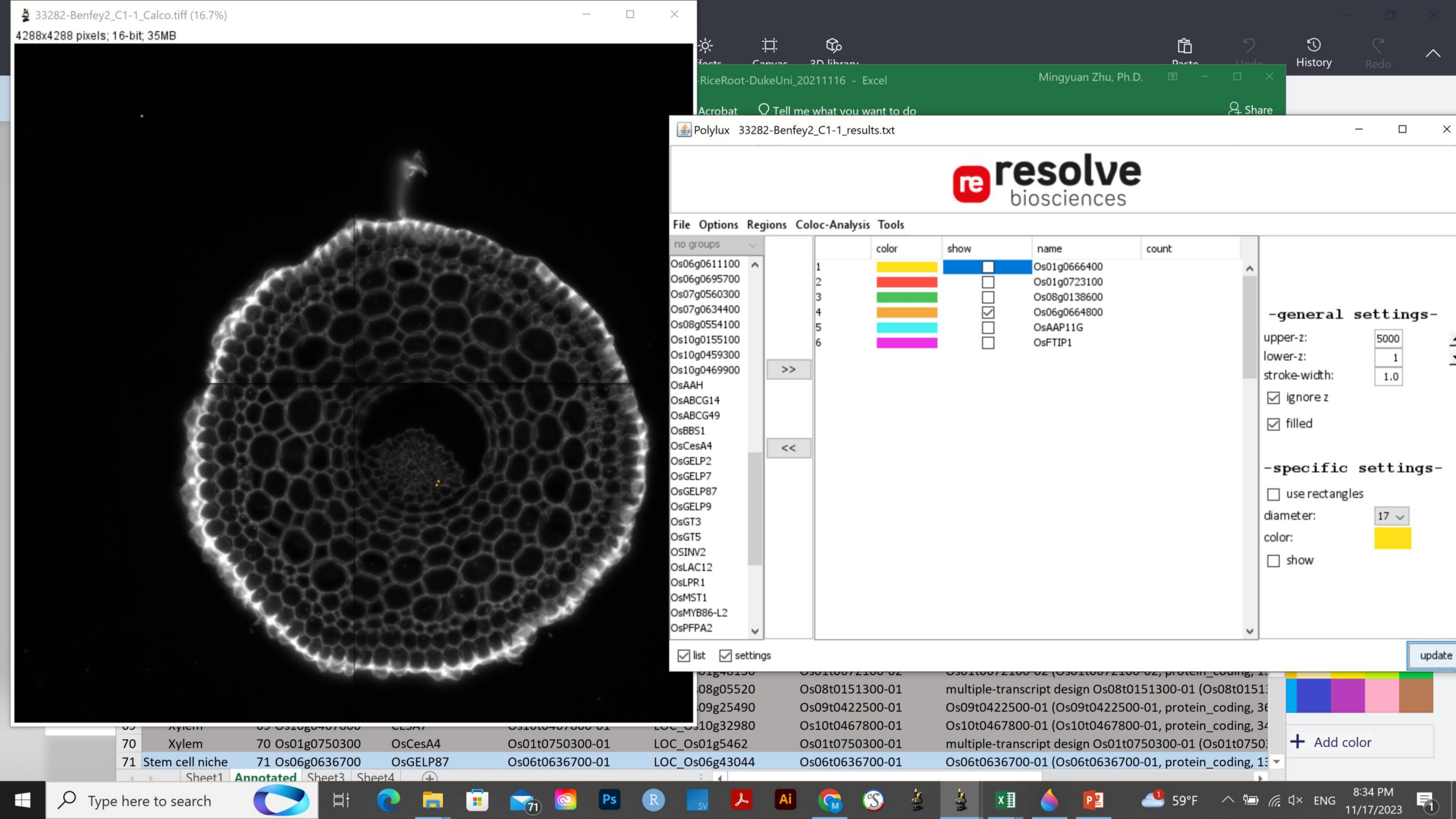
33282-Benfey2_C1-1_Calco.tiff (16.7%)
4288x4288 pixels; 16-bit; 35MB
History
Redo
-RiceRoot-DukeUni_20211116 - Excel
Mingyuan Zhu, Ph.D.
Acrobat
Tell me what you want to do
Share
Polylux 33282-Benfey2_C1-1_results.txt
resolve
biosciences
File
Options
Regions
Coloc-Analysis
Tools
no groups
Os06g0611100
Os06g0695700
Os07g0560300
Os07g0634400
Os08g0554100
Os10g0155100
Os10g0459300
Os10g0469900
OsAAH
OsABCG14
OsABCG49
OsBBS1
OsCesA4
OsGELP2
OsGELP7
OsGELP87
OsGELP9
OsGT3
OsGT5
OSINV2
OsLAC12
OsLPR1
OsMST1
OsMYB86-L2
OsPFPA2
>>
<<
color
show
name
count
1 Os01g0666400
2 Os01g0723100
3 Os08g0138600
4 Os06g0664800
5 OsAAP11G
6 OsFTIP1
-general settings-
upper-z: 5000
lower-z: 1
stroke-width: 1.0
ignore z
filled
-specific settings-
use rectangles
diameter: 17
color:
show
list
settings
update
Os08t0151300-01
multiple-transcript design Os08t0151300-01 (Os08t0151
Os09t0422500-01
Os09t0422500-01 (Os09t0422500-01, protein_coding, 3
LOC_Os10g32980
Os10t0467800-01
Os10t0467800-01 (Os10t0467800-01, protein_coding, 3
70 Xylem 70 Os01g0750300 OsCesA4 Os01t0750300-01 LOC_Os01g5462 Os01t0750300-01 multiple-transcript design Os01t0750300-01 (Os01t0750
71 Stem cell niche 71 Os06g0636700 OsGELP87 Os06t0636700-01 LOC_Os06g43044 Os06t0636700-01 Os06t0636700-01 (Os06t0636700-01, protein_coding, 1
Sheet1
Annotated
Sheet3
Sheet4
Add color
Type here to search
59°F
ENG
8:34 PM
11/17/2023

82-Benfey2_C1-2_Calco.tiff (16.7%)

288 pixels; 16-bit; 35MB

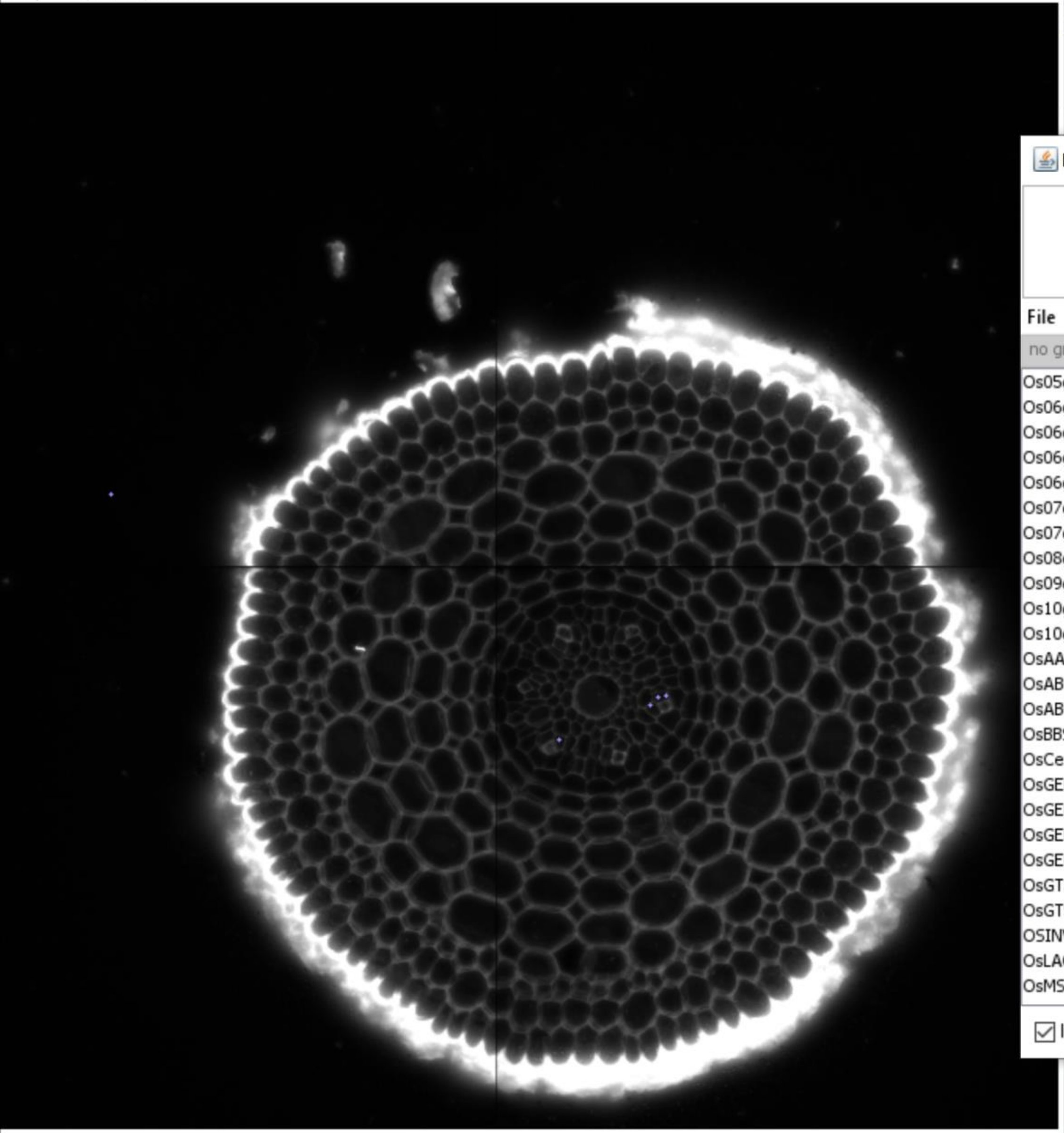

Effects Canvas 3D library Paste Undo History Redo

Mixed reality 3D view 71% Brushes

Polylux 33282-Benfey2_C1-2_results.txt

resolve biosciences

File Options Regions Coloc-Analysis Tools

no groups

Os05g0398800
Os06g0135900
Os06g0219900
Os06g0611100
Os06g0695700
Os07g0560300
Os07g0634400
Os08g0554100
Os09g0535400
Os10g0155100
Os10g0459300
OsAAH
OsABCG14
OsABCG49
OsBBS1
OsCesA4
OsGELP2
OsGELP7
OsGELP87
OsGELP9
OsGT3
OsGT5
OSINV2
OsLAC12
OsMST1

>>

<<

	color	show	name	count
1		☐	Os01g0666400	
2		☐	Os01g0723100	
3		☐	Os08g0138600	
4		☑	Os06g0664800	
5		☐	OsAAP11G	
6		☐	OsFTIP1	

-general settings-

upper-z: 5000

lower-z: 1

stroke-width: 1.0

☑ ignore z

☑ filled

-specific settings-

☐ use rectangles

diameter: 17

color:

☑ show

☑ list ☑ settings

update

+ Add color

Type here to search

57°F ENG 9:14 PM 11/17/2023

Xylem

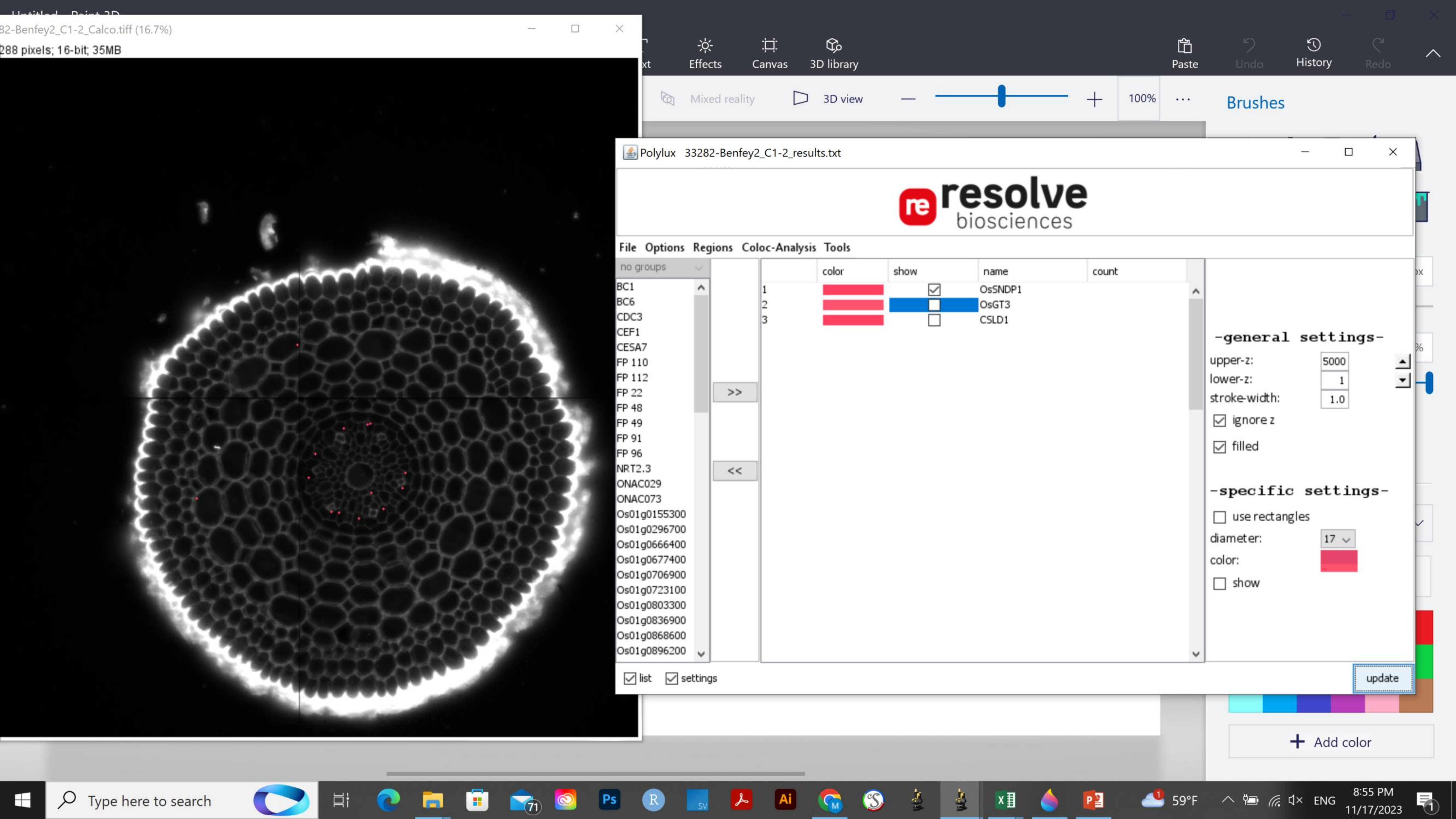
82-Benfey2_C1-2_Calco.tiff (16.7%)
288 pixels; 16-bit; 35MB
Effects
Canvas
3D library
Paste
Undo
History
Redo
Mixed reality
3D view
100%
Brushes
Polylux 33282-Benfey2_C1-2_results.txt
resolve
biosciences
File Options Regions Coloc-Analysis Tools
no groups
BC1
BC6
CDC3
CEF1
CESA7
FP 110
FP 112
FP 22
FP 48
FP 49
FP 91
FP 96
NRT2.3
ONAC029
ONAC073
Os01g0155300
Os01g0296700
Os01g0666400
Os01g0677400
Os01g0706900
Os01g0723100
Os01g0803300
Os01g0836900
Os01g0868600
Os01g0896200
>>
<<
color show name count
1 OsSNDP1
2 OsGT3
3 CSLD1
-general settings-
upper-z: 5000
lower-z: 1
stroke-width: 1.0
ignore z
filled
-specific settings-
use rectangles
diameter: 17
color:
show
list
settings
update
Add color
Type here to search
59°F
ENG
8:55 PM
11/17/2023
