# Supplementary material for: Single-cell transcriptomics reveal how root tissues adapt to soil stress
Source: Nature. 2025 Apr 30;642(8068):721–9. doi: 10.1038/s41586-025-08941-z (PMC12176638; doi:10.1038/s41586-025-08941-z)
Supplement: Supplementary file 24 — Jupyter notebook for checking the cell type identity for low-quality cells after the COPILOT processing of soil-based scRNA-seq data is included. [file 41586_2025_8941_MOESM24_ESM.zip › Data-S9_Cell_type_identity_checking_for_low-quality_cells_in_soil-based_scRNA-seq_data_Rice/Data-S9_Cell_type_identity_checking_for_low-quality_cells_in_soil-based_scRNA-seq_data_Rice.html]

Annotation\_on\_low\_quality\_cells


In [1]:

```
rm(list=ls())
setwd("/hpc/group/pbenfeylab/CheWei/")
```

In [2]:

```
suppressMessages(library(Matrix))
suppressMessages(library(DropletUtils))
suppressMessages(library(ggplot2))
suppressMessages(library(scales))
suppressMessages(library(rjson))
suppressMessages(library(R2HTML))
suppressMessages(library(zeallot))
suppressMessages(library(Seurat))
suppressMessages(library(tidyverse))
suppressMessages(library(DoubletFinder))
suppressMessages(library(rhdf5))
suppressMessages(library(grid))
suppressMessages(library(patchwork))
```

```
Warning message:
“package ‘Matrix’ was built under R version 4.2.3”
Warning message:
“package ‘ggplot2’ was built under R version 4.2.3”
Warning message:
“package ‘dplyr’ was built under R version 4.2.3”
```

In [3]:

```
sessionInfo()
```

```
R version 4.2.2 (2022-10-31)
Platform: x86_64-conda-linux-gnu (64-bit)
Running under: CentOS Stream 8

Matrix products: default
BLAS/LAPACK: /hpc/group/pbenfeylab/ch416/miniconda3/envs/seu4/lib/libopenblasp-r0.3.21.so

locale:
 [1] LC_CTYPE=C.UTF-8           LC_NUMERIC=C              
 [3] LC_TIME=en_US.UTF-8        LC_COLLATE=en_US.UTF-8    
 [5] LC_MONETARY=en_US.UTF-8    LC_MESSAGES=en_US.UTF-8   
 [7] LC_PAPER=en_US.UTF-8       LC_NAME=C                 
 [9] LC_ADDRESS=C               LC_TELEPHONE=C            
[11] LC_MEASUREMENT=en_US.UTF-8 LC_IDENTIFICATION=C       

attached base packages:
[1] grid      stats4    stats     graphics  grDevices utils     datasets 
[8] methods   base     

other attached packages:
 [1] patchwork_1.1.2             rhdf5_2.42.1               
 [3] DoubletFinder_2.0.3         forcats_0.5.2              
 [5] stringr_1.5.0               dplyr_1.1.1                
 [7] purrr_1.0.1                 readr_2.1.3                
 [9] tidyr_1.3.0                 tibble_3.2.1               
[11] tidyverse_1.3.2             SeuratObject_4.1.3         
[13] Seurat_4.1.1.9001           zeallot_0.1.0              
[15] R2HTML_2.3.3                rjson_0.2.21               
[17] scales_1.2.1                ggplot2_3.4.2              
[19] DropletUtils_1.18.1         SingleCellExperiment_1.20.0
[21] SummarizedExperiment_1.28.0 Biobase_2.58.0             
[23] GenomicRanges_1.50.0        GenomeInfoDb_1.34.8        
[25] IRanges_2.32.0              S4Vectors_0.36.0           
[27] BiocGenerics_0.44.0         MatrixGenerics_1.10.0      
[29] matrixStats_0.63.0          Matrix_1.5-4               

loaded via a namespace (and not attached):
  [1] utf8_1.2.3                spatstat.explore_3.1-0   
  [3] reticulate_1.28           R.utils_2.12.2           
  [5] tidyselect_1.2.0          htmlwidgets_1.6.2        
  [7] BiocParallel_1.32.5       Rtsne_0.16               
  [9] munsell_0.5.0             codetools_0.2-19         
 [11] ica_1.0-3                 pbdZMQ_0.3-8             
 [13] future_1.32.0             miniUI_0.1.1.1           
 [15] withr_2.5.0               spatstat.random_3.1-4    
 [17] colorspace_2.1-0          progressr_0.13.0         
 [19] uuid_1.1-0                ROCR_1.0-11              
 [21] tensor_1.5                listenv_0.9.0            
 [23] repr_1.1.4                GenomeInfoDbData_1.2.9   
 [25] polyclip_1.10-4           parallelly_1.35.0        
 [27] vctrs_0.6.2               generics_0.1.3           
 [29] timechange_0.1.1          R6_2.5.1                 
 [31] locfit_1.5-9.6            bitops_1.0-7             
 [33] rhdf5filters_1.10.1       spatstat.utils_3.0-2     
 [35] DelayedArray_0.24.0       assertthat_0.2.1         
 [37] promises_1.2.0.1          googlesheets4_1.0.1      
 [39] gtable_0.3.3              beachmat_2.14.0          
 [41] globals_0.16.2            goftest_1.2-3            
 [43] rlang_1.1.0               splines_4.2.2            
 [45] lazyeval_0.2.2            gargle_1.2.1             
 [47] spatstat.geom_3.1-0       broom_1.0.2              
 [49] reshape2_1.4.4            abind_1.4-5              
 [51] modelr_0.1.10             backports_1.4.1          
 [53] httpuv_1.6.9              tools_4.2.2              
 [55] ellipsis_0.3.2            RColorBrewer_1.1-3       
 [57] ggridges_0.5.4            Rcpp_1.0.10              
 [59] plyr_1.8.8                base64enc_0.1-3          
 [61] sparseMatrixStats_1.10.0  zlibbioc_1.44.0          
 [63] RCurl_1.98-1.6            deldir_1.0-6             
 [65] pbapply_1.7-0             cowplot_1.1.1            
 [67] zoo_1.8-12                haven_2.5.1              
 [69] ggrepel_0.9.3             cluster_2.1.4            
 [71] fs_1.6.1                  magrittr_2.0.3           
 [73] data.table_1.14.8         RSpectra_0.16-1          
 [75] scattermore_0.8           reprex_2.0.2             
 [77] lmtest_0.9-40             RANN_2.6.1               
 [79] googledrive_2.0.0         fitdistrplus_1.1-8       
 [81] hms_1.1.2                 mime_0.12                
 [83] evaluate_0.20             xtable_1.8-4             
 [85] readxl_1.4.1              fastDummies_1.6.3        
 [87] gridExtra_2.3             compiler_4.2.2           
 [89] KernSmooth_2.23-20        crayon_1.5.2             
 [91] R.oo_1.25.0               htmltools_0.5.5          
 [93] later_1.3.0               tzdb_0.3.0               
 [95] lubridate_1.9.0           DBI_1.1.3                
 [97] dbplyr_2.2.1              MASS_7.3-58.3            
 [99] cli_3.6.1                 R.methodsS3_1.8.2        
[101] parallel_4.2.2            igraph_1.4.2             
[103] pkgconfig_2.0.3           sp_1.6-0                 
[105] IRdisplay_1.1             plotly_4.10.1            
[107] scuttle_1.8.0             spatstat.sparse_3.0-1    
[109] xml2_1.3.3                dqrng_0.3.0              
[111] XVector_0.38.0            rvest_1.0.3              
[113] digest_0.6.31             sctransform_0.3.5        
[115] RcppAnnoy_0.0.20          spatstat.data_3.0-1      
[117] cellranger_1.1.0          leiden_0.4.3             
[119] uwot_0.1.14               edgeR_3.40.1             
[121] DelayedMatrixStats_1.20.0 shiny_1.7.4              
[123] lifecycle_1.0.3           nlme_3.1-162             
[125] jsonlite_1.8.4            Rhdf5lib_1.20.0          
[127] viridisLite_0.4.1         limma_3.54.0             
[129] fansi_1.0.4               pillar_1.9.0             
[131] lattice_0.21-8            fastmap_1.1.1            
[133] httr_1.4.5                survival_3.4-0           
[135] glue_1.6.2                png_0.1-8                
[137] stringi_1.7.12            HDF5Array_1.26.0         
[139] RcppHNSW_0.4.1            IRkernel_1.3.1.9000      
[141] irlba_2.3.5.1             future.apply_1.10.0
```

In [4]:

```
setwd("/hpc/group/pbenfeylab/CheWei/COPILOT_other_projects/MSU7_v3_1/")
```

In [5]:

```
source("./COPILOT_functions_HT.R")
```

In [6]:

```
pp.os.genes <- as.character(read.table("/hpc/group/pbenfeylab/CheWei/COPILOT_other_projects/Rice_Protoplasting_DEgene_FC2_list.txt", header=F)$V1)
pp.os.genes <- gsub(pattern = "_", replacement = "-", x = pp.os.genes)

mt.os.genes <- as.character(read.table("/hpc/group/pbenfeylab/CheWei/COPILOT_other_projects/MSU7_mt_name.txt", header=F)$V1)
cp.os.genes <- as.character(read.table("/hpc/group/pbenfeylab/CheWei/COPILOT_other_projects/MSU7_cp_name.txt", header=F)$V1)

sample.name <- "sc_192"
species.name = "Oryza sativa" 
transcriptome.name = "MSU7"
mt.pattern = mt.os.genes 
mt.threshold = 5
cp.pattern = cp.os.genes
top.percent = 1
filtering.ratio = 1
doublet.rate = 5
do.seurat = TRUE
do.annotation = FALSE
unwanted.genes = pp.os.genes
HVG = FALSE
HVGN = 2000
dir_to_bulk = "/hpc/group/pbenfeylab/CheWei/COPILOT_other_projects/Root_bulk_rice_curated.RD"
res = 0.5
res.pseudo.bulk = 50
min.UMI.low.quality = 100
min.UMI.high.quality = 300
legend.position = c(0.8,0.8)
dir_to_h5 = NULL
```

In [7]:

```
# load raw mtx
spliced <- readMM(paste0("./",sample.name,"/spliced.mtx"))
rownames(spliced) <- as.character(read.table(paste0("./",sample.name,"/spliced.barcodes.txt"), header=F)$V1)
colnames(spliced) <- as.character(read.table(paste0("./",sample.name,"/spliced.genes.txt"), header=F)$V1)
spliced <- t(spliced)
unspliced <- readMM(paste0("./",sample.name,"/unspliced.mtx"))
rownames(unspliced) <- as.character(read.table(paste0("./",sample.name,"/unspliced.barcodes.txt"), header=F)$V1)
colnames(unspliced) <- as.character(read.table(paste0("./",sample.name,"/unspliced.genes.txt"), header=F)$V1)
unspliced <- t(unspliced)
# Create matrix spliced, unspliced and combined 
bcs_use <- intersect(colnames(spliced),colnames(unspliced))
tot_genes <- Matrix::rowSums(spliced)
genes_use <- rownames(spliced)[tot_genes > 0]
sf <- spliced[genes_use, bcs_use]
uf <- unspliced[genes_use, bcs_use]
afr <- sf+uf
tot_gene <- Matrix::colSums(afr)
bcs_use <- colnames(afr)[tot_gene > min.UMI.low.quality]
sf <- spliced[genes_use, bcs_use]
uf <- unspliced[genes_use, bcs_use]
afr <- sf+uf
```

In [8]:

```
nc <- colSums(afr)
ng <- colSums(afr>0)
lnc <- log10(nc)
pmt <- (colSums(afr[grep(paste(mt.pattern, collapse = "|"),rownames(afr)),])/nc)*100 # percent mt
lnc.mt <- lnc[pmt >= mt.threshold]
if (length(lnc.mt)==0){
  mmt <- log10(min.UMI.high.quality)# If no high mt cell, then set the first filtering threshold at min UMI counts threshold         
} else if (length(lnc.mt)==1) {
  mmt <- lnc.mt # log10 mean UMI counts for cells above threshold
} else {
  dd <- density(lnc.mt)
  mmt <- max(dd$x[find_modes(dd$y)][dd$x[find_modes(dd$y)] < max(boxplot.stats(lnc.mt)$stats)]) # find the maximum mode
}
```

In [9]:

```
lcu <- as.numeric(log10(cumsum(rev(sort(nc))))) # log culmulative UMI counts
snc <- as.numeric(rev(sort(nc))) # sorted UMI counts
lin <- log10(seq(1,ncol(afr),1))
dYu <- diff(lcu)/diff(lin)  # the derivative  
dX <- rowMeans(embed(lin,2)) # centers the X values for plotting
local_min <- min(dYu[1:(length(which(lnc>mmt))-1)]) #local min derivative
cnu <- which(dYu<=local_min)[1]
if (snc[cnu] < min.UMI.high.quality){
  cnu <- length(which(nc > min.UMI.high.quality))# If no local minimum, then set the first filtering threshold at min UMI counts threshold  
}

ct <- sort(nc,decreasing = TRUE)[cnu] # UMI threshold at elbow point 

# Filtering on UMI counts (first round)
filter.UMI.thres.n=c(min.UMI.low.quality,ct)
filter.UMI.thres=c(ct,1000000)
nf <- afr[,(colSums(afr) > filter.UMI.thres.n[1])&(colSums(afr) <= filter.UMI.thres.n[2])] # lower quality cells
af <- afr[,(colSums(afr) > filter.UMI.thres[1])&(colSums(afr) < filter.UMI.thres[2])] # higher quality cells
nnf <- log1p((nf/colSums(nf))*10000) #log normalization
naf <- log1p((af/colSums(af))*10000) #log normalization
np <- rowSums(nnf)/ncol(nnf) # low quality cell profile
sp <- rowSums(naf)/ncol(naf) # high quality cell profile

cornp <- apply(naf,2,cor,y=np)
corsp <- apply(naf,2,cor,y=sp)

sidx <- c()
nidx <- c()
for (i in 1:ncol(af)){
  if (corsp[i] > cornp[i]){
    sidx <- c(sidx, i)
  } else {
    nidx <- c(nidx, i)
  }
}

#Decide filtering threshold: lenient: 1, strict:0, moderate: 0 < filtering.ratio < 1
nidx_thres <- length(nidx)*filtering.ratio #moderate
print(paste0("threshold cell number: ",nidx_thres))
n_iteration <- 0
print(paste0("removed cells: ",length(nidx)))
while(length(nidx)!=0 & length(nidx) >= nidx_thres){
  n_iteration <- n_iteration+1 
  #nf <- cbind(nf,afr[,colnames(af)[nidx]])
  nf <- cbind(nf,afr[,which(colnames(afr) %in% colnames(af)[nidx])])
  #af <- afr[,colnames(af)[sidx]]
  af <- afr[,which(colnames(afr) %in% colnames(af)[sidx])]
  nnf <- log1p((nf/colSums(nf))*10000) #log normalization
  naf <- log1p((af/colSums(af))*10000) #log normalization
  np <- rowSums(nnf)/ncol(nnf) # low quality cell profile
  sp <- rowSums(naf)/ncol(naf) # high quality cell profile
  cornp <- apply(naf,2,cor,y=np)
  corsp <- apply(naf,2,cor,y=sp)   
  sidx <- c()
  nidx <- c()
  for (i in 1:ncol(af)){            
    if (corsp[i] > cornp[i]){                
      sidx <- c(sidx, i)
    } else {                
      nidx <- c(nidx, i)
    }
  }
  print(paste0("iteration: ",n_iteration))
  print(paste0("removed cells: ",length(nidx)))
}
if (is.null(dir_to_h5)){
  sf <- sf[,colnames(af)]
  uf <- uf[,colnames(af)] 
}

message("Iteration finished")
```

```
Warning message in asMethod(object):
“sparse->dense coercion: allocating vector of size 2.6 GiB”
Warning message in asMethod(object):
“sparse->dense coercion: allocating vector of size 2.6 GiB”
```

```
[1] "threshold cell number: 3232"
[1] "removed cells: 3232"
```

```
Warning message in asMethod(object):
“sparse->dense coercion: allocating vector of size 1.8 GiB”
Warning message in asMethod(object):
“sparse->dense coercion: allocating vector of size 1.8 GiB”
```

```
[1] "iteration: 1"
[1] "removed cells: 331"
```

```
Iteration finished
```

In [10]:

```
#prepare data.frame for ggplot2
nc <- colSums(af)
ng <- colSums(af>0)
lnc <- log10(nc)
lng <-log10(ng)
pmts <- (colSums(af[grep(paste(mt.pattern, collapse = "|"),rownames(af)),])/nc)*100 # percent mt for high quality cell
ncn <- colSums(nf)
ngn <- colSums(nf>0)
lncn <- log10(ncn)
lngn <- log10(ngn)
pmtn <- (colSums(nf[grep(paste(mt.pattern, collapse = "|"),rownames(nf)),])/ncn)*100 # percent mt for low quality cell
```

In [11]:

```
select <- rep(paste("high quality"),length(lnc))
label_mt <- paste0("percent mt >= ",mt.threshold)
select[which(pmts >= mt.threshold)] <- label_mt # index of percent mt > mt.threshold
quantile_idx <- which(lnc < quantile(lnc[which(select=="high quality")],probs = (1-top.percent/100))) # quantile top 1 % tail to determine outliers
ssidx <- intersect(quantile_idx, which(select=="high quality")) # index of final high quality cell
select[ssidx] <- paste("high quality (",length(ssidx),")",sep="")
select[which(select=="high quality")] <- paste("top ",top.percent,"% (",length(which(select=="high quality")),")",sep="")
nonselect <- rep("low quality",length(lncn))
nonselect[which(pmtn >= mt.threshold)] <- label_mt # index of percent mt > mt.threshold
select <- c(select,nonselect)
select[which(select==label_mt)] <- paste("percent mt >= ",mt.threshold," (",length(which(select==label_mt)),")",sep="")
select[which(select=="low quality")] <- paste0("low quality (",length(which(select=="low quality")),")")
lncc <- c(lnc,lncn)
lngc <- c(lng,lngn)
lnc_gg <- data.frame(select=select,lnc=lncc)
lnc_gg$rank <- match(lnc_gg$lnc,sort(lnc_gg$lnc,decreasing = TRUE))
lnc_gg$lnc <- 10^lnc_gg$lnc
lnc_gg$select <- factor(lnc_gg$select, levels = c(paste0("high quality (",length(grep("high",select)),")"), 
                                                  paste0("low quality (",length(grep("low",select)),")"),
                                                  paste0("percent mt >= ",mt.threshold," (",length(grep("percent",select)),")"),
                                                  paste0("top ",top.percent,"% (",length(grep("top",select)),")")))
lng_gg <- data.frame(select=select,lng=lngc)
lng_gg$rank <- match(lng_gg$lng,sort(lng_gg$lng,decreasing = TRUE))
lng_gg$lng <- 10^lng_gg$lng
lng_gg$select <- factor(lng_gg$select, levels = c(paste0("high quality (",length(grep("high",select)),")"), 
                                                  paste0("low quality (",length(grep("low",select)),")"),
                                                  paste0("percent mt >= ",mt.threshold," (",length(grep("percent",select)),")"),
                                                  paste0("top ",top.percent,"% (",length(grep("top",select)),")")))

af <- af[,ssidx]
if (is.null(dir_to_h5)){
  sf <- sf[,ssidx]
  uf <- uf[,ssidx]
}
```

## Plotting¶

In [12]:

```
bc_rank_plot <- function(lnc_gg){
    print({
     ggplot(lnc_gg, aes(x=rank, y=lnc, col = select, alpha=select)) +   
     geom_point(size=3) + 
     scale_x_log10(labels = plain, breaks = trans_breaks("log10", function(x) round(10^x, 0))) + 
     scale_y_log10(labels = plain, breaks = trans_breaks('log10', function(x) floor(10^x)))+
     scale_color_manual(values = c("#003B6D","#EBEDF3","#d96459","#f2e394"), name = NULL, guide = guide_legend(reverse = TRUE)) +
     scale_alpha_manual(values = c(0.8,0.8,0.8,0.8)) +
     labs(x = 'Barcodes', y = 'UMI Counts') +
     guides(alpha = FALSE, colour = guide_legend(reverse = TRUE, override.aes=list(size = 5))) +
     theme_bw() + 
     theme(panel.border = element_blank(), panel.grid.major = element_blank(),
            panel.grid.minor = element_blank(), axis.line = element_line(colour = "black"),
            axis.title.x = element_text(margin = margin(t = 20, r = 0, b = 0, l = 0)),
            axis.title.y = element_text(margin = margin(t = 0, r = 20, b = 0, l = 0)),
            legend.background = element_rect(fill = 'transparent'),
            legend.position = "none",legend.key=element_blank(),
            plot.margin=unit(c(0,1,0,0),"cm"))
    })
}

UMI_hist_plot <- function(lnc_gg,legend.position){
    print({
    ggplot(lnc_gg, aes(lnc, fill = select)) +
    geom_histogram(bins = 100)+
    scale_fill_manual(values = c("#003B6D","#EBEDF3","#d96459","#f2e394"))+
    xlab("UMI Counts")+
    ylab("Freqency")+
    scale_y_continuous(labels = plain)+
    scale_x_log10(labels = plain, breaks = trans_breaks("log10", function(x) round(10^x, 0))) + 
    theme(panel.background=element_blank(),plot.background=element_blank(),legend.title = element_blank(), axis.line = element_line(colour = "black"),
            axis.title.x = element_text(margin = margin(t = 20, r = 0, b = 0, l = 0)),
            axis.title.y = element_text(margin = margin(t = 0, r = 20, b = 0, l = 0)),legend.position=legend.position,
            plot.margin=unit(c(0,1,0,0),"cm"))
    })
}

Gene_hist_plot <- function(lng_gg){
    print({
    ggplot(lng_gg, aes(lng, fill = select)) +
    geom_histogram(bins = 100)+
    scale_fill_manual(values = c("#003B6D","#EBEDF3","#d96459","#f2e394"))+
    xlab("Number of Genes")+
    ylab("Freqency")+
    scale_y_continuous(labels = plain)+
    scale_x_log10(labels = plain, breaks = trans_breaks("log10", function(x) round(10^x, 0))) + 
    theme(panel.background=element_blank(),plot.background=element_blank(),legend.title = element_blank(), axis.line = element_line(colour = "black"),
            axis.title.x = element_text(margin = margin(t = 20, r = 0, b = 0, l = 0)),
            axis.title.y = element_text(margin = margin(t = 0, r = 20, b = 0, l = 0)),legend.position="none",
            plot.margin=unit(c(0,1,0,0),"cm"))
    })
}
```

In [13]:

```
UMI_hist_plot(lnc_gg=lnc_gg, legend.position=legend.position)
Gene_hist_plot(lng_gg=lng_gg)
bc_rank_plot(lnc_gg=lnc_gg)
```

```
Warning message:
“The `<scale>` argument of `guides()` cannot be `FALSE`. Use "none" instead as of ggplot2 3.3.4.”
```

In [14]:

```
lnc_gg
```

A data.frame: 76801 x 3

|  | select | lnc | rank |
| --- | --- | --- | --- |
|  | <fct> | <dbl> | <int> |
| AAACCCAAGAGTCAAT | high quality (7021) | 40535 | 613 |
| AAACCCAAGCTGAGCA | high quality (7021) | 44733 | 475 |
| AAACCCAAGTTGGCGA | high quality (7021) | 16794 | 4074 |
| AAACCCAGTATACCTG | high quality (7021) | 10044 | 5502 |
| AAACCCATCCGTACGG | high quality (7021) | 37617 | 761 |
| AAACCCATCGAACTCA | high quality (7021) | 18862 | 3560 |
| AAACGAAAGCGCCTTG | high quality (7021) | 9684 | 5556 |
| AAACGAAAGCTCTATG | high quality (7021) | 13499 | 4861 |
| AAACGAACAATGGCCC | high quality (7021) | 1782 | 7616 |
| AAACGAAGTATTGAGA | top 1% (71) | 118962 | 17 |
| AAACGAAGTCGCAGTC | high quality (7021) | 34822 | 934 |
| AAACGAAGTGATGTAA | high quality (7021) | 38587 | 703 |
| AAACGAAGTGCATGTT | high quality (7021) | 39865 | 644 |
| AAACGAAGTGTCCTAA | high quality (7021) | 23205 | 2530 |
| AAACGAAGTTTACCAG | high quality (7021) | 39685 | 652 |
| AAACGAATCACTACGA | high quality (7021) | 23639 | 2446 |
| AAACGAATCAGGACGA | high quality (7021) | 15833 | 4322 |
| AAACGAATCTTGATTC | high quality (7021) | 12368 | 5071 |
| AAACGCTAGCACCTGC | high quality (7021) | 23708 | 2436 |
| AAACGCTCACAGCGCT | high quality (7021) | 12193 | 5104 |
| AAACGCTCAGTCTTCC | high quality (7021) | 13086 | 4939 |
| AAACGCTGTAGTCTTG | high quality (7021) | 8580 | 5742 |
| AAACGCTGTATGCAAA | high quality (7021) | 40148 | 630 |
| AAACGCTTCAAACGAA | high quality (7021) | 29625 | 1431 |
| AAACGCTTCGCAGAGA | high quality (7021) | 26420 | 1905 |
| AAAGAACAGGGCCTCT | high quality (7021) | 40665 | 604 |
| AAAGAACAGTCGGCAA | high quality (7021) | 19798 | 3307 |
| AAAGAACCAGCTGTTA | high quality (7021) | 37900 | 745 |
| AAAGAACCAGGCGATA | high quality (7021) | 8625 | 5733 |
| AAAGAACCATACCACA | high quality (7021) | 32748 | 1116 |
| ... | ... | ... | ... |
| TTTATGCAGCTACTAC | low quality (65686) | 1179 | 9926 |
| TTTATGCTCAATCTTC | low quality (65686) | 1251 | 9389 |
| TTTCACATCTTTCCGG | low quality (65686) | 1471 | 8290 |
| TTTCAGTCAGCAGATG | low quality (65686) | 1324 | 8916 |
| TTTCAGTCATGAATCC | low quality (65686) | 1598 | 7937 |
| TTTCATGGTTCGGCTG | percent mt >= 5 (4023) | 1305 | 9035 |
| TTTCCTCGTCGCACAC | percent mt >= 5 (4023) | 1209 | 9668 |
| TTTCCTCTCCGTGTCT | low quality (65686) | 1327 | 8900 |
| TTTCCTCTCCTTGACC | low quality (65686) | 7031 | 5967 |
| TTTCCTCTCGTAGCTA | low quality (65686) | 2919 | 6855 |
| TTTCGATGTACGACTT | low quality (65686) | 1220 | 9584 |
| TTTCGATGTGGCACTC | low quality (65686) | 1320 | 8945 |
| TTTCGATTCCATTGCC | percent mt >= 5 (4023) | 1523 | 8128 |
| TTTGACTAGATGCTTC | low quality (65686) | 1319 | 8949 |
| TTTGACTCAGAATCGG | low quality (65686) | 1252 | 9381 |
| TTTGACTCAGGATTCT | low quality (65686) | 1163 | 10060 |
| TTTGACTGTATACGGG | percent mt >= 5 (4023) | 1325 | 8910 |
| TTTGACTGTTAGGACG | low quality (65686) | 1334 | 8867 |
| TTTGACTTCGCGCCAA | percent mt >= 5 (4023) | 1735 | 7677 |
| TTTGATCAGCAGCAGT | low quality (65686) | 1306 | 9026 |
| TTTGATCCAGCACACC | low quality (65686) | 1447 | 8362 |
| TTTGATCCAGCTGAAG | low quality (65686) | 1282 | 9177 |
| TTTGATCTCCGCTGTT | low quality (65686) | 1725 | 7687 |
| TTTGATCTCCGTGTCT | percent mt >= 5 (4023) | 1168 | 10014 |
| TTTGATCTCTCGCAGG | low quality (65686) | 1364 | 8724 |
| TTTGGAGCAATCGAAA | low quality (65686) | 1146 | 10217 |
| TTTGGTTAGAAGGCTC | low quality (65686) | 1740 | 7671 |
| TTTGGTTCACAGCCTG | low quality (65686) | 1583 | 7977 |
| TTTGGTTGTTCCGCTT | low quality (65686) | 1244 | 9437 |
| TTTGTTGTCGTAGAGG | low quality (65686) | 1287 | 9144 |

## Annotate all cells¶

In [15]:

```
## All cells
seu <- suppressWarnings(CreateSeuratObject(counts = afr, assay = "RNA"))
```

In [16]:

```
lnc_gg$barcodes <- rownames(lnc_gg)
seu$barcodes <- colnames(seu)
join <- left_join(seu@meta.data, lnc_gg, by="barcodes")
seu$quality_anno <- join$select
```

In [51]:

```
seu$quality_anno <- as.character(seu$quality_anno)
seu$quality_anno[grep('low quality',seu$quality_anno)]='low quality'
seu$quality_anno[grep('high quality',seu$quality_anno)]='high quality'
seu$quality_anno[grep('percent mt',seu$quality_anno)]='percent mt > 5%'
seu$quality_anno[grep('top',seu$quality_anno)]='top 1%'
seu$quality_anno <- factor(seu$quality_anno, levels = c("high quality", "low quality", "percent mt > 5%", "top 1%"))
```

In [52]:

```
## Subset 10k cells
seu_sub <- subset(seu, cells=names(rev(sort(seu$nCount_RNA))[1:10000]))
```

In [53]:

```
head(seu_sub@meta.data)
```

A data.frame: 6 x 5

|  | orig.ident | nCount\_RNA | nFeature\_RNA | barcodes | quality\_anno |
| --- | --- | --- | --- | --- | --- |
|  | <fct> | <dbl> | <int> | <chr> | <fct> |
| CTCCACAAGGATGGCT | SeuratProject | 189644 | 10711 | CTCCACAAGGATGGCT | top 1% |
| CATTCATTCAAGTGGG | SeuratProject | 186682 | 11697 | CATTCATTCAAGTGGG | top 1% |
| CTATCCGAGGCGACAT | SeuratProject | 166833 | 10250 | CTATCCGAGGCGACAT | top 1% |
| TGATTTCTCAAATGCC | SeuratProject | 155552 | 10744 | TGATTTCTCAAATGCC | top 1% |
| AGACAGGAGGCGTTAG | SeuratProject | 150390 | 10044 | AGACAGGAGGCGTTAG | top 1% |
| CAATCGAAGTGAGGCT | SeuratProject | 147870 | 10385 | CAATCGAAGTGAGGCT | top 1% |

In [54]:

```
table(seu_sub$quality_anno)
```

```
   high quality     low quality percent mt > 5%          top 1% 
           7021            2411             497              71
```

In [55]:

```
## SCTransform
suppressWarnings(
seu_sub <- SCTransform(seu_sub, variable.features.n = nrow(seu), assay = "RNA", new.assay.name = "SCT", verbose = TRUE)
)
```

```
Calculating cell attributes from input UMI matrix: log_umi

Variance stabilizing transformation of count matrix of size 28133 by 10000

Model formula is y ~ log_umi

Get Negative Binomial regression parameters per gene

Using 2000 genes, 5000 cells
```

```
  |======================================================================| 100%
```

```
Found 64 outliers - those will be ignored in fitting/regularization step


Second step: Get residuals using fitted parameters for 28133 genes
```

```
  |======================================================================| 100%
```

```
Computing corrected count matrix for 28133 genes
```

```
  |======================================================================| 100%
```

```
Calculating gene attributes

Wall clock passed: Time difference of 2.330525 mins

Determine variable features

Place corrected count matrix in counts slot

Centering data matrix

Set default assay to SCT
```

## Load markers¶

In [111]:

```
zscore <- function(x){(x-mean(x))/sd(x)}
known.good.markers.gel <- read.csv("../../Meta_data/Markers_for_annotation_Final_Gel.csv")
known.good.markers.gel$Gene.ID <- gsub("^LOC_","LOC-",known.good.markers.gel$Gene.ID)
known.good.markers.soil <- read.csv("../../Meta_data/Markers_for_annotation_Final_Soil.csv")
known.good.markers.soil$Gene.ID <- gsub("^LOC_","LOC-",known.good.markers.soil$Gene.ID)
```

In [112]:

```
#known.good.markers.soil$Cell.type <- gsub("Late stage Endodermis", "Endodermis", known.good.markers.soil$Cell.type, ignore.case = FALSE, perl = FALSE,fixed = T, useBytes = FALSE);
known.good.markers.gel$Cell.type <- gsub("Early Metaxylem", "Metaxylem", known.good.markers.gel$Cell.type, ignore.case = FALSE, perl = FALSE,fixed = T, useBytes = FALSE);
known.good.markers.gel$Cell.type <- gsub("Meristem", "Stem cell niche", known.good.markers.gel$Cell.type, ignore.case = FALSE, perl = FALSE,fixed = T, useBytes = FALSE);
known.good.markers.gel$Cell.type <- gsub("Root Cap junction", "Root Cap", known.good.markers.gel$Cell.type, ignore.case = FALSE, perl = FALSE,fixed = T, useBytes = FALSE);
known.good.markers.gel$Cell.type <- gsub("Late stage Endodermis", "Endodermis", known.good.markers.gel$Cell.type, ignore.case = FALSE, perl = FALSE,fixed = T, useBytes = FALSE);
known.good.markers.gel$Cell.type <- gsub("Metaxylem", "Xylem", known.good.markers.gel$Cell.type, ignore.case = FALSE, perl = FALSE,fixed = T, useBytes = FALSE);
```

In [113]:

```
known.good.markers.gel <- known.good.markers.gel[-which(known.good.markers.gel$Source != "Benfey Lab"),]
known.good.markers.gel <- known.good.markers.gel[-which(known.good.markers.gel$Gene.ID == "LOC-Os03g11734"),]
```

In [114]:

```
known.good.markers.gel
```

A data.frame: 33 x 5

|  | Gene.ID | Cell.type | Source | Atlas.Expression | X |
| --- | --- | --- | --- | --- | --- |
|  | <chr> | <chr> | <chr> | <chr> | <chr> |
| 2 | LOC-Os01g50820 | Atrichoblast | Benfey Lab | Confirmed |  |
| 3 | LOC-Os01g64840 | Atrichoblast | Benfey Lab | Confirmed |  |
| 4 | LOC-Os01g19220 | Cortex | Benfey Lab | Confirmed |  |
| 5 | LOC-Os03g04310 | Cortex | Benfey Lab | Confirmed |  |
| 6 | LOC-Os05g33080 | Cortex | Benfey Lab | Confirmed |  |
| 7 | LOC-Os06g30730 | Cortex | Benfey Lab | Confirmed |  |
| 10 | LOC-Os01g15810 | Endodermis | Benfey Lab | Confirmed |  |
| 11 | LOC-Os01g67390 | Endodermis | Benfey Lab | Confirmed |  |
| 12 | LOC-Os03g18640 | Endodermis | Benfey Lab | Confirmed |  |
| 13 | LOC-Os03g24930 | Endodermis | Benfey Lab | Confirmed |  |
| 14 | LOC-Os10g06680 | Endodermis | Benfey Lab | Confirmed |  |
| 15 | LOC-Os03g02460 | Exodermis | Benfey Lab | Confirmed |  |
| 16 | LOC-Os03g37411 | Exodermis | Benfey Lab | Confirmed |  |
| 17 | LOC-Os04g37980 | Exodermis | Benfey Lab | Confirmed |  |
| 25 | LOC-Os01g19170 | Pericycle | Benfey Lab | Confirmed |  |
| 26 | LOC-Os01g58910 | Pericycle | Benfey Lab | Confirmed |  |
| 27 | LOC-Os07g44060 | Pericycle | Benfey Lab | Confirmed |  |
| 29 | LOC-Os04g41350 | Phloem | Benfey Lab | Confirmed |  |
| 30 | LOC-Os06g45410 | Phloem | Benfey Lab | Confirmed |  |
| 31 | LOC-Os08g04400 | Phloem | Benfey Lab | Confirmed |  |
| 34 | LOC-Os08g02300 | Sclerenchyma | Benfey Lab | Confirmed |  |
| 35 | LOC-Os01g03530 | Trichoblast | Benfey Lab | Confirmed | LPR1 |
| 36 | LOC-Os01g11750 | Trichoblast | Benfey Lab | Confirmed | GELP9 |
| 37 | LOC-Os03g04210 | Trichoblast | Benfey Lab | Confirmed |  |
| 38 | LOC-Os04g45290 | Trichoblast | Benfey Lab | Confirmed | VIN1 |
| 39 | LOC-Os05g38770 | Trichoblast | Benfey Lab | Confirmed | RCLK188 |
| 41 | LOC-Os06g48050 | Trichoblast | Benfey Lab | Confirmed |  |
| 42 | LOC-Os10g03400 | Trichoblast | Benfey Lab | Confirmed | SNDP1 |
| 44 | LOC-Os01g48130 | Xylem | Benfey Lab | Confirmed |  |
| 45 | LOC-Os01g54620 | Xylem | Benfey Lab | Confirmed |  |
| 46 | LOC-Os01g67090 | Xylem | Benfey Lab | Confirmed |  |
| 47 | LOC-Os09g25490 | Xylem | Benfey Lab | Confirmed |  |
| 48 | LOC-Os10g32980 | Xylem | Benfey Lab | Confirmed |  |

In [115]:

```
known.good.markers.soil
```

A data.frame: 9 x 4

| Gene.ID | Cell.type | Source | X |
| --- | --- | --- | --- |
| <chr> | <chr> | <chr> | <chr> |
| LOC-Os01g64840 | Atrichoblast | Spatial, Benfey lab | Os01g0868600 |
| LOC-Os03g04310 | Cortex | Spatial, Benfey lab | Os03g0135700 |
| LOC-Os01g15810 | Late stage Endodermis | Spatial, Benfey lab | Os01g0263000 |
| LOC-Os03g37411 | Exodermis | Spatial, Benfey lab | Os03g0570800 |
| LOC-Os01g19170 | Pericycle | Spatial, Benfey lab | Os01g0296200 |
| LOC-Os06g45410 | Phloem | Spatial, Benfey lab | Os06g0664800 |
| LOC-Os08g02300 | Sclerenchyma | Spatial, Benfey lab | Os08g0115800 |
| LOC-Os10g42750 | Trichoblast | Spatial, Benfey lab | Os10g0578200 |
| LOC-Os01g48130 | Xylem | Spatial, Benfey lab | Os01g0672100 |

In [60]:

```
## Decide marker list to use
known.good.markers <- known.good.markers.gel
```

In [61]:

```
seu_sub <- RunPCA(seu_sub, verbose = FALSE, approx = TRUE)
seu_sub <- RunUMAP(seu_sub, reduction = "pca", dims = 1:50)
seu_sub <- FindNeighbors(seu_sub, reduction = "pca")
```

```
17:00:03 UMAP embedding parameters a = 0.9922 b = 1.112

17:00:03 Read 10000 rows and found 50 numeric columns

17:00:03 Using Annoy for neighbor search, n_neighbors = 30

17:00:03 Building Annoy index with metric = cosine, n_trees = 50

0%   10   20   30   40   50   60   70   80   90   100%

[----|----|----|----|----|----|----|----|----|----|

*
*
*
*
*
*
*
*
*
*
*
*
*
*
*
*
*
*
*
*
*
*
*
*
*
*
*
*
*
*
*
*
*
*
*
*
*
*
*
*
*
*
*
*
*
*
*
*
*
*
|

17:00:05 Writing NN index file to temp file /tmp/RtmpS82Et5/file13be59125c6714

17:00:05 Searching Annoy index using 1 thread, search_k = 3000

17:00:08 Annoy recall = 100%

17:00:11 Commencing smooth kNN distance calibration using 1 thread
 with target n_neighbors = 30

17:00:16 Initializing from normalized Laplacian + noise (using RSpectra)

17:00:16 Commencing optimization for 500 epochs, with 423752 positive edges

17:00:31 Optimization finished

Computing nearest neighbor graph

Computing SNN
```

In [62]:

```
suppressMessages(suppressWarnings(
  seu_sub <- FindClusters(seu_sub, resolution = 0.1, algorithm = 3)
))
```

```
Modularity Optimizer version 1.3.0 by Ludo Waltman and Nees Jan van Eck

Number of nodes: 10000
Number of edges: 308221

Running smart local moving algorithm...
Maximum modularity in 10 random starts: 0.9529
Number of communities: 6
Elapsed time: 5 seconds
```

In [67]:

```
options(repr.plot.width=8, repr.plot.height=8)
p2 <- DimPlot(seu_sub, reduction = "umap", label=TRUE)+NoLegend()+ggtitle("Clusters")
```

In [64]:

```
msc <- c()
suppressWarnings(
for (i in as.character(unique(known.good.markers$Cell.type))){
    if (length(known.good.markers[which(known.good.markers$Cell.type== i),]$Gene.ID)>1){
    msc <- cbind(msc, as.numeric(apply(apply(as.matrix(seu_sub@assays$SCT@data)[known.good.markers[which(known.good.markers$Cell.type== i),]$Gene.ID,], 1, zscore), 1, mean)))       
    } else {
    msc <- cbind(msc, as.numeric(zscore(as.matrix(seu_sub@assays$SCT@data)[known.good.markers[which(known.good.markers$Cell.type== i),]$Gene.ID,])))      
    }

}
)
colnames(msc) <- as.character(unique(known.good.markers$Cell.type))
rownames(msc) <- colnames(seu_sub)
```

In [33]:

```
#anno <- seu_sub$seurat_clusters
#for (i in unique(seu_sub$seurat_clusters)){
#    if (max(apply(msc[which(seu_sub$seurat_clusters==i),],2,mean))>0){
#        ct <- names(which.max(apply(msc[which(seu_sub$seurat_clusters==i),],2,mean)))
#    } else {
#        ct <- "NA"
#    }
#        anno <- gsub(paste0("^",i,"$"), ct, anno, ignore.case = FALSE, perl = FALSE,fixed = FALSE, useBytes = FALSE)
#}
```

In [34]:

```
#seu_sub$score.crude.anno <- anno
```

In [86]:

```
# Plot marker annotation
#order <- c("Stem cell niche", "Sclerenchyma", "Columella", "Root Cap", "Atrichoblast", "Trichoblast", "Cortex", "Endodermis", "Phloem", "Xylem", "Vascular Tissue","Pericycle","Exodermis","Epidermis", "Protoxylem", "Metaxylem", "NA")
#palette <- c("#9400d3", "#DCD0FF","#5ab953", "#bfef45", "#008080", "#21B6A8", "#82b6ff", "#0000FF","#e6194b", "#9a6324", "#ffe119", "#ff9900", "#ffd4e3", "#dd77ec", "#9a6324", "#ddaa6f", "#EEEEEE")
#seu_sub$score.crude.anno <- factor(seu_sub$score.crude.anno , levels = order[sort(match(unique(seu_sub$score.crude.anno),order))]) 
#color <- palette[sort(match(unique(seu_sub$score.crude.anno),order))]
#options(repr.plot.width=8, repr.plot.height=8)
#p1 <- DimPlot(seu_sub, pt.size=2,reduction = "umap", group.by = "score.crude.anno", cols = color)+ggtitle("Z-Score Annotation")
```

In [98]:

```
options(repr.plot.width=8, repr.plot.height=8)
p1 <- DimPlot(seu_sub, reduction = "umap", group.by="quality_anno", cols=c("#003B6D","#EBEDF3","#d96459","#f2e394"),pt.size=1)
```

In [76]:

```
options(repr.plot.width=8, repr.plot.height=8)
(p3 <- ggplot(seu_sub@meta.data) +
  aes(x = seurat_clusters, fill = factor(quality_anno)) +
  geom_bar(position = "fill")+
  scale_fill_manual("Quality", values = c("#003B6D","#EBEDF3","#d96459","#f2e394"))
 + theme_classic()+ theme(axis.text.x = element_text(size=18,,angle = 0, vjust = 0.5, hjust=0.5))+xlab("Clusters")+ylab("Proportion")
)
```

In [89]:

```
options(repr.plot.width=12, repr.plot.height=8)
p1 + p2 + p3 + plot_annotation(
  title = 'sc_192',
  theme = theme(plot.title = element_text(size = 24))
)
```

In [78]:

```
seu_sub$Atrichoblast <- msc[,1]
seu_sub$Cortex <- msc[,2]
seu_sub$Endodermis <- msc[,3]
seu_sub$Exodermis <- msc[,4]
seu_sub$Pericycle <- msc[,5]
seu_sub$Phloem <- msc[,6]
seu_sub$Sclerenchyma <- msc[,7]
seu_sub$Trichoblast <- msc[,8]
seu_sub$Xylem <- msc[,9]
```

In [79]:

```
head(seu_sub@meta.data)
```

A data.frame: 6 x 18

|  | orig.ident | nCount\_RNA | nFeature\_RNA | barcodes | quality\_anno | nCount\_SCT | nFeature\_SCT | SCT\_snn\_res.0.1 | seurat\_clusters | Atrichoblast | Cortex | Endodermis | Exodermis | Pericycle | Phloem | Sclerenchyma | Trichoblast | Xylem |
| --- | --- | --- | --- | --- | --- | --- | --- | --- | --- | --- | --- | --- | --- | --- | --- | --- | --- | --- |
|  | <fct> | <dbl> | <int> | <chr> | <fct> | <dbl> | <int> | <fct> | <fct> | <dbl> | <dbl> | <dbl> | <dbl> | <dbl> | <dbl> | <dbl> | <dbl> | <dbl> |
| CTCCACAAGGATGGCT | SeuratProject | 189644 | 10711 | CTCCACAAGGATGGCT | top 1% | 11153 | 2709 | 3 | 3 | -0.08720169 | -0.1987674 | 2.40164264 | -0.1687598 | -0.1074084 | -0.1009859 | -0.1807478 | -0.1397487 | -0.2763372 |
| CATTCATTCAAGTGGG | SeuratProject | 186682 | 11697 | CATTCATTCAAGTGGG | top 1% | 11833 | 3378 | 3 | 3 | -0.08720169 | -0.1987674 | -0.07135777 | -0.1687598 | 1.3392096 | -0.1009859 | -0.1807478 | -0.1397487 | -0.2763372 |
| CTATCCGAGGCGACAT | SeuratProject | 166833 | 10250 | CTATCCGAGGCGACAT | top 1% | 11375 | 2789 | 3 | 3 | -0.08720169 | -0.1987674 | -0.24707896 | -0.1687598 | -0.1074084 | -0.1009859 | -0.1807478 | -0.1397487 | -0.2763372 |
| TGATTTCTCAAATGCC | SeuratProject | 155552 | 10744 | TGATTTCTCAAATGCC | top 1% | 11533 | 3445 | 3 | 3 | -0.08720169 | -0.1987674 | 0.76866794 | -0.1687598 | -0.1074084 | -0.1009859 | -0.1807478 | -0.1397487 | -0.2763372 |
| AGACAGGAGGCGTTAG | SeuratProject | 150390 | 10044 | AGACAGGAGGCGTTAG | top 1% | 11294 | 2986 | 3 | 3 | -0.08720169 | -0.1987674 | 0.81202292 | -0.1687598 | -0.1074084 | -0.1009859 | -0.1807478 | -0.1397487 | -0.2763372 |
| CAATCGAAGTGAGGCT | SeuratProject | 147870 | 10385 | CAATCGAAGTGAGGCT | top 1% | 11684 | 3356 | 3 | 3 | -0.08720169 | -0.1987674 | -0.24707896 | -0.1687598 | -0.1074084 | -0.1009859 | -0.1807478 | -0.1397487 | -0.2763372 |

In [90]:

```
(p4 <- VlnPlot(seu_sub, features = c("Atrichoblast", "Trichoblast", "Exodermis", "Sclerenchyma", "Cortex","Endodermis", "Pericycle","Phloem","Xylem")))
```

In [102]:

```
Idents(seu_sub) <- "quality_anno"
(p5 <- VlnPlot(seu_sub, features = c("Atrichoblast", "Trichoblast", "Exodermis", "Sclerenchyma", "Cortex","Endodermis", "Pericycle","Phloem","Xylem"))+xlab(""))
```

In [91]:

```
f1 <- FeaturePlot(seu_sub, reduction = "umap", feature="Atrichoblast", order=TRUE ,pt.size=1)
f2 <- FeaturePlot(seu_sub, reduction = "umap", feature="Trichoblast", order=TRUE ,pt.size=1)
f3 <- FeaturePlot(seu_sub, reduction = "umap", feature="Exodermis", order=TRUE,pt.size=1)
f4 <- FeaturePlot(seu_sub, reduction = "umap", feature="Sclerenchyma", order=TRUE,pt.size=1)
f5 <- FeaturePlot(seu_sub, reduction = "umap", feature="Cortex", order=TRUE,pt.size=1)
f6 <- FeaturePlot(seu_sub, reduction = "umap", feature="Endodermis", order=TRUE,pt.size=1)
f7 <- FeaturePlot(seu_sub, reduction = "umap", feature="Pericycle", order=TRUE,pt.size=1)
f8 <- FeaturePlot(seu_sub, reduction = "umap", feature="Phloem", order=TRUE,pt.size=1)
f9 <- FeaturePlot(seu_sub, reduction = "umap", feature="Xylem", order=TRUE,pt.size=1)
```

In [99]:

```
options(repr.plot.width=12, repr.plot.height=16)
(p1 | p2 |p3) / (f1 | f2 | f3) / (f4 | f5 | f6) / (f7 | f8 | f9)+ plot_annotation(
  title = 'sc_192',
  subtitle = 'The feature plots shows averaged z-score of cell type markers',
  theme = theme(plot.title = element_text(size = 18),
               plot.subtitle = element_text(size = 18))
)
```

In [103]:

```
options(repr.plot.width=10, repr.plot.height=12)
(p1) / (p5) + plot_layout(heights = c(1, 2)) + plot_annotation(
  title = 'sc_192',
  subtitle = 'The feature plots shows averaged z-score of cell type markers',
  theme = theme(plot.title = element_text(size = 18),
               plot.subtitle = element_text(size = 18))
)
```

## Write the function to automitize the process¶

In [71]:

```
pp.os.genes <- as.character(read.table("/hpc/group/pbenfeylab/CheWei/COPILOT_other_projects/Rice_Protoplasting_DEgene_FC2_list.txt", header=F)$V1)
pp.os.genes <- gsub(pattern = "_", replacement = "-", x = pp.os.genes)

mt.os.genes <- as.character(read.table("/hpc/group/pbenfeylab/CheWei/COPILOT_other_projects/MSU7_mt_name.txt", header=F)$V1)
cp.os.genes <- as.character(read.table("/hpc/group/pbenfeylab/CheWei/COPILOT_other_projects/MSU7_cp_name.txt", header=F)$V1)

sample.name <- "sc_192"
species.name = "Oryza sativa" 
transcriptome.name = "MSU7"
mt.pattern = mt.os.genes 
mt.threshold = 5
cp.pattern = cp.os.genes
top.percent = 1
filtering.ratio = 1
doublet.rate = 5
do.seurat = TRUE
do.annotation = FALSE
unwanted.genes = pp.os.genes
HVG = FALSE
HVGN = 2000
dir_to_bulk = "/hpc/group/pbenfeylab/CheWei/COPILOT_other_projects/Root_bulk_rice_curated.RD"
res = 0.5
res.pseudo.bulk = 50
min.UMI.low.quality = 100
min.UMI.high.quality = 300
legend.position = c(0.8,0.8)
dir_to_h5 = NULL

zscore <- function(x){(x-mean(x))/sd(x)}
known.good.markers.gel <- read.csv("../../Meta_data/Markers_for_annotation_Final_Gel.csv")
known.good.markers.gel$Gene.ID <- gsub("^LOC_","LOC-",known.good.markers.gel$Gene.ID)
known.good.markers.soil <- read.csv("../../Meta_data/Markers_for_annotation_Final_Soil.csv")
known.good.markers.soil$Gene.ID <- gsub("^LOC_","LOC-",known.good.markers.soil$Gene.ID)

known.good.markers.soil$Cell.type <- gsub("Late stage Endodermis", "Endodermis", known.good.markers.soil$Cell.type, ignore.case = FALSE, perl = FALSE,fixed = T, useBytes = FALSE);
known.good.markers.gel$Cell.type <- gsub("Early Metaxylem", "Metaxylem", known.good.markers.gel$Cell.type, ignore.case = FALSE, perl = FALSE,fixed = T, useBytes = FALSE);
known.good.markers.gel$Cell.type <- gsub("Meristem", "Stem cell niche", known.good.markers.gel$Cell.type, ignore.case = FALSE, perl = FALSE,fixed = T, useBytes = FALSE);
known.good.markers.gel$Cell.type <- gsub("Root Cap junction", "Root Cap", known.good.markers.gel$Cell.type, ignore.case = FALSE, perl = FALSE,fixed = T, useBytes = FALSE);
known.good.markers.gel$Cell.type <- gsub("Late stage Endodermis", "Endodermis", known.good.markers.gel$Cell.type, ignore.case = FALSE, perl = FALSE,fixed = T, useBytes = FALSE);
known.good.markers.gel$Cell.type <- gsub("Metaxylem", "Xylem", known.good.markers.gel$Cell.type, ignore.case = FALSE, perl = FALSE,fixed = T, useBytes = FALSE);

known.good.markers.gel <- known.good.markers.gel[-which(known.good.markers.gel$Source != "Benfey Lab"),]
known.good.markers.gel <- known.good.markers.gel[-which(known.good.markers.gel$Gene.ID == "LOC-Os03g11734"),]
```

In [104]:

```
check_low_quality <- function(sample.name, target.cells, marker.list){
# load raw mtx
spliced <- readMM(paste0("./",sample.name,"/spliced.mtx"))
rownames(spliced) <- as.character(read.table(paste0("./",sample.name,"/spliced.barcodes.txt"), header=F)$V1)
colnames(spliced) <- as.character(read.table(paste0("./",sample.name,"/spliced.genes.txt"), header=F)$V1)
spliced <- t(spliced)
unspliced <- readMM(paste0("./",sample.name,"/unspliced.mtx"))
rownames(unspliced) <- as.character(read.table(paste0("./",sample.name,"/unspliced.barcodes.txt"), header=F)$V1)
colnames(unspliced) <- as.character(read.table(paste0("./",sample.name,"/unspliced.genes.txt"), header=F)$V1)
unspliced <- t(unspliced)
# Create matrix spliced, unspliced and combined 
bcs_use <- intersect(colnames(spliced),colnames(unspliced))
tot_genes <- Matrix::rowSums(spliced)
genes_use <- rownames(spliced)[tot_genes > 0]
sf <- spliced[genes_use, bcs_use]
uf <- unspliced[genes_use, bcs_use]
afr <- sf+uf
tot_gene <- Matrix::colSums(afr)
bcs_use <- colnames(afr)[tot_gene > min.UMI.low.quality]
sf <- spliced[genes_use, bcs_use]
uf <- unspliced[genes_use, bcs_use]
afr <- sf+uf
nc <- colSums(afr)
ng <- colSums(afr>0)
lnc <- log10(nc)
pmt <- (colSums(afr[grep(paste(mt.pattern, collapse = "|"),rownames(afr)),])/nc)*100 # percent mt
lnc.mt <- lnc[pmt >= mt.threshold]
if (length(lnc.mt)==0){
  mmt <- log10(min.UMI.high.quality)# If no high mt cell, then set the first filtering threshold at min UMI counts threshold         
} else if (length(lnc.mt)==1) {
  mmt <- lnc.mt # log10 mean UMI counts for cells above threshold
} else {
  dd <- density(lnc.mt)
  mmt <- max(dd$x[find_modes(dd$y)][dd$x[find_modes(dd$y)] < max(boxplot.stats(lnc.mt)$stats)]) # find the maximum mode
}
lcu <- as.numeric(log10(cumsum(rev(sort(nc))))) # log culmulative UMI counts
snc <- as.numeric(rev(sort(nc))) # sorted UMI counts
lin <- log10(seq(1,ncol(afr),1))
dYu <- diff(lcu)/diff(lin)  # the derivative  
dX <- rowMeans(embed(lin,2)) # centers the X values for plotting
local_min <- min(dYu[1:(length(which(lnc>mmt))-1)]) #local min derivative
cnu <- which(dYu<=local_min)[1]
if (snc[cnu] < min.UMI.high.quality){
  cnu <- length(which(nc > min.UMI.high.quality))# If no local minimum, then set the first filtering threshold at min UMI counts threshold  
}

ct <- sort(nc,decreasing = TRUE)[cnu] # UMI threshold at elbow point 

# Filtering on UMI counts (first round)
filter.UMI.thres.n=c(min.UMI.low.quality,ct)
filter.UMI.thres=c(ct,1000000)
nf <- afr[,(colSums(afr) > filter.UMI.thres.n[1])&(colSums(afr) <= filter.UMI.thres.n[2])] # lower quality cells
af <- afr[,(colSums(afr) > filter.UMI.thres[1])&(colSums(afr) < filter.UMI.thres[2])] # higher quality cells
nnf <- log1p((nf/colSums(nf))*10000) #log normalization
naf <- log1p((af/colSums(af))*10000) #log normalization
np <- rowSums(nnf)/ncol(nnf) # low quality cell profile
sp <- rowSums(naf)/ncol(naf) # high quality cell profile

cornp <- apply(naf,2,cor,y=np)
corsp <- apply(naf,2,cor,y=sp)

sidx <- c()
nidx <- c()
for (i in 1:ncol(af)){
  if (corsp[i] > cornp[i]){
    sidx <- c(sidx, i)
  } else {
    nidx <- c(nidx, i)
  }
}

#Decide filtering threshold: lenient: 1, strict:0, moderate: 0 < filtering.ratio < 1
nidx_thres <- length(nidx)*filtering.ratio #moderate
print(paste0("threshold cell number: ",nidx_thres))
n_iteration <- 0
print(paste0("removed cells: ",length(nidx)))
while(length(nidx)!=0 & length(nidx) >= nidx_thres){
  n_iteration <- n_iteration+1 
  #nf <- cbind(nf,afr[,colnames(af)[nidx]])
  nf <- cbind(nf,afr[,which(colnames(afr) %in% colnames(af)[nidx])])
  #af <- afr[,colnames(af)[sidx]]
  af <- afr[,which(colnames(afr) %in% colnames(af)[sidx])]
  nnf <- log1p((nf/colSums(nf))*10000) #log normalization
  naf <- log1p((af/colSums(af))*10000) #log normalization
  np <- rowSums(nnf)/ncol(nnf) # low quality cell profile
  sp <- rowSums(naf)/ncol(naf) # high quality cell profile
  cornp <- apply(naf,2,cor,y=np)
  corsp <- apply(naf,2,cor,y=sp)   
  sidx <- c()
  nidx <- c()
  for (i in 1:ncol(af)){            
    if (corsp[i] > cornp[i]){                
      sidx <- c(sidx, i)
    } else {                
      nidx <- c(nidx, i)
    }
  }
  print(paste0("iteration: ",n_iteration))
  print(paste0("removed cells: ",length(nidx)))
}
if (is.null(dir_to_h5)){
  sf <- sf[,colnames(af)]
  uf <- uf[,colnames(af)] 
}

message("Iteration finished")
    
#prepare data.frame for ggplot2
nc <- colSums(af)
ng <- colSums(af>0)
lnc <- log10(nc)
lng <-log10(ng)
pmts <- (colSums(af[grep(paste(mt.pattern, collapse = "|"),rownames(af)),])/nc)*100 # percent mt for high quality cell
ncn <- colSums(nf)
ngn <- colSums(nf>0)
lncn <- log10(ncn)
lngn <- log10(ngn)
pmtn <- (colSums(nf[grep(paste(mt.pattern, collapse = "|"),rownames(nf)),])/ncn)*100 # percent mt for low quality cell
    
select <- rep(paste("high quality"),length(lnc))
label_mt <- paste0("percent mt >= ",mt.threshold)
select[which(pmts >= mt.threshold)] <- label_mt # index of percent mt > mt.threshold
quantile_idx <- which(lnc < quantile(lnc[which(select=="high quality")],probs = (1-top.percent/100))) # quantile top 1 % tail to determine outliers
ssidx <- intersect(quantile_idx, which(select=="high quality")) # index of final high quality cell
select[ssidx] <- paste("high quality (",length(ssidx),")",sep="")
select[which(select=="high quality")] <- paste("top ",top.percent,"% (",length(which(select=="high quality")),")",sep="")
nonselect <- rep("low quality",length(lncn))
nonselect[which(pmtn >= mt.threshold)] <- label_mt # index of percent mt > mt.threshold
select <- c(select,nonselect)
select[which(select==label_mt)] <- paste("percent mt >= ",mt.threshold," (",length(which(select==label_mt)),")",sep="")
select[which(select=="low quality")] <- paste0("low quality (",length(which(select=="low quality")),")")
lncc <- c(lnc,lncn)
lngc <- c(lng,lngn)
lnc_gg <- data.frame(select=select,lnc=lncc)
lnc_gg$rank <- match(lnc_gg$lnc,sort(lnc_gg$lnc,decreasing = TRUE))
lnc_gg$lnc <- 10^lnc_gg$lnc
lnc_gg$select <- factor(lnc_gg$select, levels = c(paste0("high quality (",length(grep("high",select)),")"), 
                                                  paste0("low quality (",length(grep("low",select)),")"),
                                                  paste0("percent mt >= ",mt.threshold," (",length(grep("percent",select)),")"),
                                                  paste0("top ",top.percent,"% (",length(grep("top",select)),")")))
lng_gg <- data.frame(select=select,lng=lngc)
lng_gg$rank <- match(lng_gg$lng,sort(lng_gg$lng,decreasing = TRUE))
lng_gg$lng <- 10^lng_gg$lng
lng_gg$select <- factor(lng_gg$select, levels = c(paste0("high quality (",length(grep("high",select)),")"), 
                                                  paste0("low quality (",length(grep("low",select)),")"),
                                                  paste0("percent mt >= ",mt.threshold," (",length(grep("percent",select)),")"),
                                                  paste0("top ",top.percent,"% (",length(grep("top",select)),")")))

af <- af[,ssidx]
if (is.null(dir_to_h5)){
  sf <- sf[,ssidx]
  uf <- uf[,ssidx]
}

## All cells
seu <- suppressWarnings(CreateSeuratObject(counts = afr, assay = "RNA"))

lnc_gg$barcodes <- rownames(lnc_gg)
seu$barcodes <- colnames(seu)
join <- left_join(seu@meta.data, lnc_gg, by="barcodes")
seu$quality_anno <- join$select
seu$quality_anno <- as.character(seu$quality_anno)
seu$quality_anno[grep('low quality',seu$quality_anno)]='low quality'
seu$quality_anno[grep('high quality',seu$quality_anno)]='high quality'
seu$quality_anno[grep('percent mt',seu$quality_anno)]='percent mt > 5%'
seu$quality_anno[grep('top',seu$quality_anno)]='top 1%'
seu$quality_anno <- factor(seu$quality_anno, levels = c("high quality", "low quality", "percent mt > 5%", "top 1%"))
    
## Subset 
seu_sub <- subset(seu, cells=names(rev(sort(seu$nCount_RNA))[1:target.cells]))
## SCTransform
suppressWarnings(
seu_sub <- SCTransform(seu_sub, variable.features.n = nrow(seu), assay = "RNA", new.assay.name = "SCT", verbose = FALSE)
)

seu_sub <- RunPCA(seu_sub, verbose = FALSE, approx = TRUE)
seu_sub <- RunUMAP(seu_sub, reduction = "pca", dims = 1:50, verbose = FALSE)
seu_sub <- FindNeighbors(seu_sub, reduction = "pca")
suppressMessages(suppressWarnings(
  seu_sub <- FindClusters(seu_sub, resolution = 10, algorithm = 3)
))
    
#options(repr.plot.width=8, repr.plot.height=8)
#print(DimPlot(seu_sub, reduction = "umap", label=TRUE)+NoLegend())

## Decide marker list to use
known.good.markers <- marker.list
    
msc <- c()
suppressWarnings(
for (i in as.character(unique(known.good.markers$Cell.type))){
    if (length(known.good.markers[which(known.good.markers$Cell.type== i),]$Gene.ID)>1){
    msc <- cbind(msc, as.numeric(apply(apply(as.matrix(seu_sub@assays$SCT@data)[known.good.markers[which(known.good.markers$Cell.type== i),]$Gene.ID,], 1, zscore), 1, mean)))       
    } else {
    msc <- cbind(msc, as.numeric(zscore(as.matrix(seu_sub@assays$SCT@data)[known.good.markers[which(known.good.markers$Cell.type== i),]$Gene.ID,])))      
    }

}
)
colnames(msc) <- as.character(unique(known.good.markers$Cell.type))
rownames(msc) <- colnames(seu_sub)

#anno <- seu_sub$seurat_clusters
#for (i in unique(seu_sub$seurat_clusters)){
#    if (max(apply(msc[which(seu_sub$seurat_clusters==i),],2,mean))>0){
#        ct <- names(which.max(apply(msc[which(seu_sub$seurat_clusters==i),],2,mean)))
#    } else {
#        ct <- "NA"
#    }
#        anno <- gsub(paste0("^",i,"$"), ct, anno, ignore.case = FALSE, perl = FALSE,fixed = FALSE, useBytes = FALSE)
#}

#seu_sub$score.crude.anno <- anno

# Plot marker annotation
#order <- c("Stem cell niche", "Sclerenchyma", "Columella", "Root Cap", "Atrichoblast", "Trichoblast", "Cortex", "Endodermis", "Phloem", "Xylem", "Vascular Tissue","Pericycle","Exodermis","Epidermis", "Protoxylem", "Metaxylem", "NA")
#palette <- c("#9400d3", "#DCD0FF","#5ab953", "#bfef45", "#008080", "#21B6A8", "#82b6ff", "#0000FF","#e6194b", "#9a6324", "#ffe119", "#ff9900", "#ffd4e3", "#dd77ec", "#9a6324", "#ddaa6f", "#EEEEEE")
#seu_sub$score.crude.anno <- factor(seu_sub$score.crude.anno , levels = order[sort(match(unique(seu_sub$score.crude.anno),order))]) 
#color <- palette[sort(match(unique(seu_sub$score.crude.anno),order))]
#options(repr.plot.width=8, repr.plot.height=8)
#p1 <- DimPlot(seu_sub, pt.size=2,reduction = "umap", group.by = "score.crude.anno", cols = color)+ggtitle("Z-Score Annotation")

options(repr.plot.width=8, repr.plot.height=8)
p1 <- DimPlot(seu_sub, reduction = "umap", group.by="quality_anno", cols=c("#003B6D","#EBEDF3","#d96459","#f2e394"),pt.size=1)
    
#options(repr.plot.width=16, repr.plot.height=8)
#print(p1 + p2 + plot_annotation(
#  title = sample.name,
#  theme = theme(plot.title = element_text(size = 24))
#-))

seu_sub$Atrichoblast <- msc[,1]
seu_sub$Cortex <- msc[,2]
seu_sub$Endodermis <- msc[,3]
seu_sub$Exodermis <- msc[,4]
seu_sub$Pericycle <- msc[,5]
seu_sub$Phloem <- msc[,6]
seu_sub$Sclerenchyma <- msc[,7]
seu_sub$Trichoblast <- msc[,8]
seu_sub$Xylem <- msc[,9]

#f1 <- FeaturePlot(seu_sub, reduction = "umap", feature="Atrichoblast")
#f2 <- FeaturePlot(seu_sub, reduction = "umap", feature="Trichoblast")
#f3 <- FeaturePlot(seu_sub, reduction = "umap", feature="Exodermis")
#f4 <- FeaturePlot(seu_sub, reduction = "umap", feature="Sclerenchyma")
#f5 <- FeaturePlot(seu_sub, reduction = "umap", feature="Cortex")
#f6 <- FeaturePlot(seu_sub, reduction = "umap", feature="Endodermis")
#f7 <- FeaturePlot(seu_sub, reduction = "umap", feature="Pericycle")
#f8 <- FeaturePlot(seu_sub, reduction = "umap", feature="Phloem")
#f9 <- FeaturePlot(seu_sub, reduction = "umap", feature="Xylem")

Idents(seu_sub) <- "quality_anno"
(p2 <- VlnPlot(seu_sub, features = c("Atrichoblast", "Trichoblast", "Exodermis", "Sclerenchyma", "Cortex","Endodermis", "Pericycle","Phloem","Xylem"))+xlab(""))
    
options(repr.plot.width=10, repr.plot.height=12)
print(
(p1) / (p2)+ plot_layout(heights = c(1, 2))+ plot_annotation(
  title = sample.name,
  subtitle = 'The feature plots shows averaged z-score of cell type markers',
  theme = theme(plot.title = element_text(size = 18),
               plot.subtitle = element_text(size = 18))
))

}
```

In [105]:

```
check_low_quality("sc_192", 10000, known.good.markers.gel)
```

```
Warning message in asMethod(object):
“sparse->dense coercion: allocating vector of size 2.6 GiB”
Warning message in asMethod(object):
“sparse->dense coercion: allocating vector of size 2.6 GiB”
```

```
[1] "threshold cell number: 3232"
[1] "removed cells: 3232"
```

```
Warning message in asMethod(object):
“sparse->dense coercion: allocating vector of size 1.8 GiB”
Warning message in asMethod(object):
“sparse->dense coercion: allocating vector of size 1.8 GiB”
```

```
[1] "iteration: 1"
[1] "removed cells: 331"
```

```
Iteration finished

Computing nearest neighbor graph

Computing SNN
```

```
Modularity Optimizer version 1.3.0 by Ludo Waltman and Nees Jan van Eck

Number of nodes: 10000
Number of edges: 308221

Running smart local moving algorithm...
Maximum modularity in 10 random starts: 0.6753
Number of communities: 97
Elapsed time: 4 seconds
```

In [106]:

```
check_low_quality("sc_193", 10000, known.good.markers.gel)
```

```
Warning message in asMethod(object):
“sparse->dense coercion: allocating vector of size 1.9 GiB”
Warning message in asMethod(object):
“sparse->dense coercion: allocating vector of size 1.9 GiB”
```

```
[1] "threshold cell number: 1896"
[1] "removed cells: 1896"
```

```
Warning message in asMethod(object):
“sparse->dense coercion: allocating vector of size 1.4 GiB”
Warning message in asMethod(object):
“sparse->dense coercion: allocating vector of size 1.4 GiB”
```

```
[1] "iteration: 1"
[1] "removed cells: 255"
```

```
Iteration finished

Computing nearest neighbor graph

Computing SNN
```

```
Modularity Optimizer version 1.3.0 by Ludo Waltman and Nees Jan van Eck

Number of nodes: 10000
Number of edges: 303876

Running smart local moving algorithm...
Maximum modularity in 10 random starts: 0.6673
Number of communities: 94
Elapsed time: 4 seconds
```

In [107]:

```
check_low_quality("sc_194", 10000, known.good.markers.gel)
```

```
Warning message in asMethod(object):
“sparse->dense coercion: allocating vector of size 1.5 GiB”
Warning message in asMethod(object):
“sparse->dense coercion: allocating vector of size 1.5 GiB”
```

```
[1] "threshold cell number: 1481"
[1] "removed cells: 1481"
```

```
Warning message in asMethod(object):
“sparse->dense coercion: allocating vector of size 1.2 GiB”
Warning message in asMethod(object):
“sparse->dense coercion: allocating vector of size 1.2 GiB”
```

```
[1] "iteration: 1"
[1] "removed cells: 177"
```

```
Iteration finished

Computing nearest neighbor graph

Computing SNN
```

```
Modularity Optimizer version 1.3.0 by Ludo Waltman and Nees Jan van Eck

Number of nodes: 10000
Number of edges: 309121

Running smart local moving algorithm...
Maximum modularity in 10 random starts: 0.6539
Number of communities: 88
Elapsed time: 4 seconds
```

In [108]:

```
check_low_quality("sc_195", 10000, known.good.markers.gel)
```

```
Warning message in asMethod(object):
“sparse->dense coercion: allocating vector of size 1.9 GiB”
Warning message in asMethod(object):
“sparse->dense coercion: allocating vector of size 1.9 GiB”
```

```
[1] "threshold cell number: 2665"
[1] "removed cells: 2665"
```

```
Warning message in asMethod(object):
“sparse->dense coercion: allocating vector of size 1.2 GiB”
Warning message in asMethod(object):
“sparse->dense coercion: allocating vector of size 1.2 GiB”
```

```
[1] "iteration: 1"
[1] "removed cells: 252"
```

```
Iteration finished

Computing nearest neighbor graph

Computing SNN
```

```
Modularity Optimizer version 1.3.0 by Ludo Waltman and Nees Jan van Eck

Number of nodes: 10000
Number of edges: 312806

Running smart local moving algorithm...
Maximum modularity in 10 random starts: 0.6702
Number of communities: 94
Elapsed time: 4 seconds
```

In [109]:

```
check_low_quality("sc_196", 10000, known.good.markers.gel)
```

```
Warning message in asMethod(object):
“sparse->dense coercion: allocating vector of size 1.6 GiB”
Warning message in asMethod(object):
“sparse->dense coercion: allocating vector of size 1.6 GiB”
```

```
[1] "threshold cell number: 1365"
[1] "removed cells: 1365"
```

```
Warning message in asMethod(object):
“sparse->dense coercion: allocating vector of size 1.2 GiB”
Warning message in asMethod(object):
“sparse->dense coercion: allocating vector of size 1.2 GiB”
```

```
[1] "iteration: 1"
[1] "removed cells: 120"
```

```
Iteration finished

Computing nearest neighbor graph

Computing SNN
```

```
Modularity Optimizer version 1.3.0 by Ludo Waltman and Nees Jan van Eck

Number of nodes: 10000
Number of edges: 310697

Running smart local moving algorithm...
Maximum modularity in 10 random starts: 0.6751
Number of communities: 96
Elapsed time: 4 seconds
```

In [116]:

```
check_low_quality("sc_199", 20000, known.good.markers.soil)
```

```
Warning message in asMethod(object):
“sparse->dense coercion: allocating vector of size 4.3 GiB”
Warning message in asMethod(object):
“sparse->dense coercion: allocating vector of size 4.3 GiB”
```

```
[1] "threshold cell number: 5024"
[1] "removed cells: 5024"
```

```
Warning message in asMethod(object):
“sparse->dense coercion: allocating vector of size 3.0 GiB”
Warning message in asMethod(object):
“sparse->dense coercion: allocating vector of size 3.0 GiB”
```

```
[1] "iteration: 1"
[1] "removed cells: 651"
```

```
Iteration finished

Computing nearest neighbor graph

Computing SNN
```

```
Modularity Optimizer version 1.3.0 by Ludo Waltman and Nees Jan van Eck

Number of nodes: 20000
Number of edges: 623162

Running smart local moving algorithm...
Maximum modularity in 10 random starts: 0.7205
Number of communities: 113
Elapsed time: 11 seconds
```

In [117]:

```
check_low_quality("sc_200", 20000, known.good.markers.soil)
```

```
Warning message in asMethod(object):
“sparse->dense coercion: allocating vector of size 6.9 GiB”
Warning message in asMethod(object):
“sparse->dense coercion: allocating vector of size 6.9 GiB”
```

```
[1] "threshold cell number: 8840"
[1] "removed cells: 8840"
```

```
Warning message in asMethod(object):
“sparse->dense coercion: allocating vector of size 4.7 GiB”
Warning message in asMethod(object):
“sparse->dense coercion: allocating vector of size 4.7 GiB”
```

```
[1] "iteration: 1"
[1] "removed cells: 770"
```

```
Iteration finished

Computing nearest neighbor graph

Computing SNN
```

```
Modularity Optimizer version 1.3.0 by Ludo Waltman and Nees Jan van Eck

Number of nodes: 20000
Number of edges: 623603

Running smart local moving algorithm...
Maximum modularity in 10 random starts: 0.7291
Number of communities: 114
Elapsed time: 10 seconds
```

In [118]:

```
check_low_quality("sc_201", 20000, known.good.markers.soil)
```

```
Warning message in asMethod(object):
“sparse->dense coercion: allocating vector of size 5.7 GiB”
Warning message in asMethod(object):
“sparse->dense coercion: allocating vector of size 5.7 GiB”
```

```
[1] "threshold cell number: 6581"
[1] "removed cells: 6581"
```

```
Warning message in asMethod(object):
“sparse->dense coercion: allocating vector of size 4.0 GiB”
Warning message in asMethod(object):
“sparse->dense coercion: allocating vector of size 4.0 GiB”
```

```
[1] "iteration: 1"
[1] "removed cells: 416"
```

```
Iteration finished

Computing nearest neighbor graph

Computing SNN
```

```
Modularity Optimizer version 1.3.0 by Ludo Waltman and Nees Jan van Eck

Number of nodes: 20000
Number of edges: 634467

Running smart local moving algorithm...
Maximum modularity in 10 random starts: 0.7239
Number of communities: 110
Elapsed time: 10 seconds
```

In [119]:

```
check_low_quality("sc_202", 20000, known.good.markers.soil)
```

```
Warning message in asMethod(object):
“sparse->dense coercion: allocating vector of size 3.9 GiB”
Warning message in asMethod(object):
“sparse->dense coercion: allocating vector of size 3.9 GiB”
```

```
[1] "threshold cell number: 3634"
[1] "removed cells: 3634"
```

```
Warning message in asMethod(object):
“sparse->dense coercion: allocating vector of size 2.9 GiB”
Warning message in asMethod(object):
“sparse->dense coercion: allocating vector of size 2.9 GiB”
```

```
[1] "iteration: 1"
[1] "removed cells: 174"
```

```
Iteration finished

Computing nearest neighbor graph

Computing SNN
```

```
Modularity Optimizer version 1.3.0 by Ludo Waltman and Nees Jan van Eck

Number of nodes: 20000
Number of edges: 628558

Running smart local moving algorithm...
Maximum modularity in 10 random starts: 0.6947
Number of communities: 112
Elapsed time: 10 seconds
```
