# Supplementary material for: Single-cell transcriptomics reveal how root tissues adapt to soil stress
Source: Nature. 2025 Apr 30;642(8068):721–9. doi: 10.1038/s41586-025-08941-z (PMC12176638; doi:10.1038/s41586-025-08941-z)
Supplement: Supplementary file 19 — Expression patterns of cell type markers in spatial transcriptomics data for gel-grown roots. A PDF summary file that includes all the sample and gene information for visualization is included. The raw spatial transcriptomics data for gel-grown roots, as well as the list of candidate genes for probe designing are also included. [file 41586_2025_8941_MOESM19_ESM.zip › 41586_2025_8941_MOESM19_ESM/Supplementary Data 4_Marker_expression_in_gel-based_Spatial_transcriptomics_Rice/Marker expression-spatial summary.pdf]

Longitudinal sections

Spatial-radial

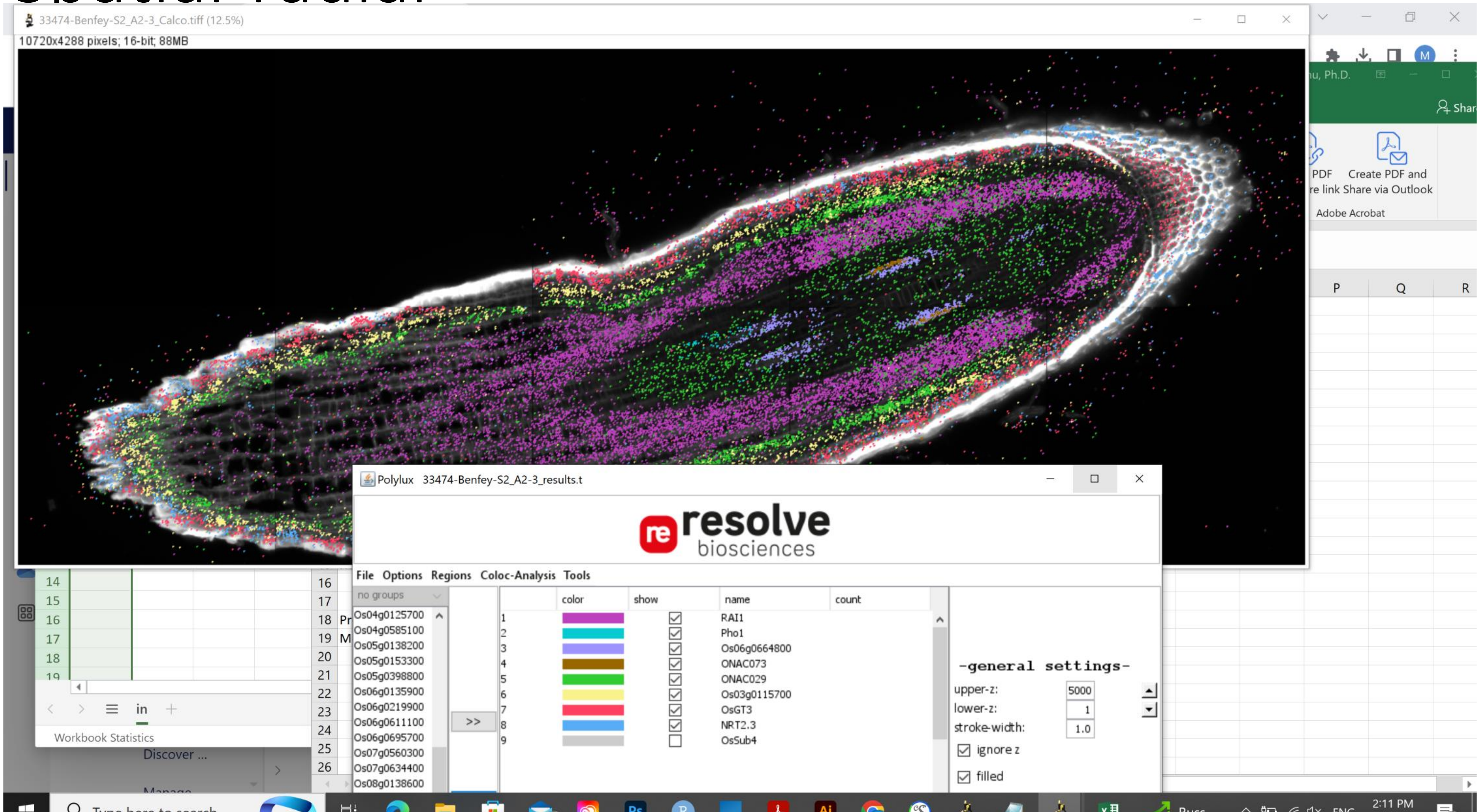

Trichoblast markers with different expression induction timing

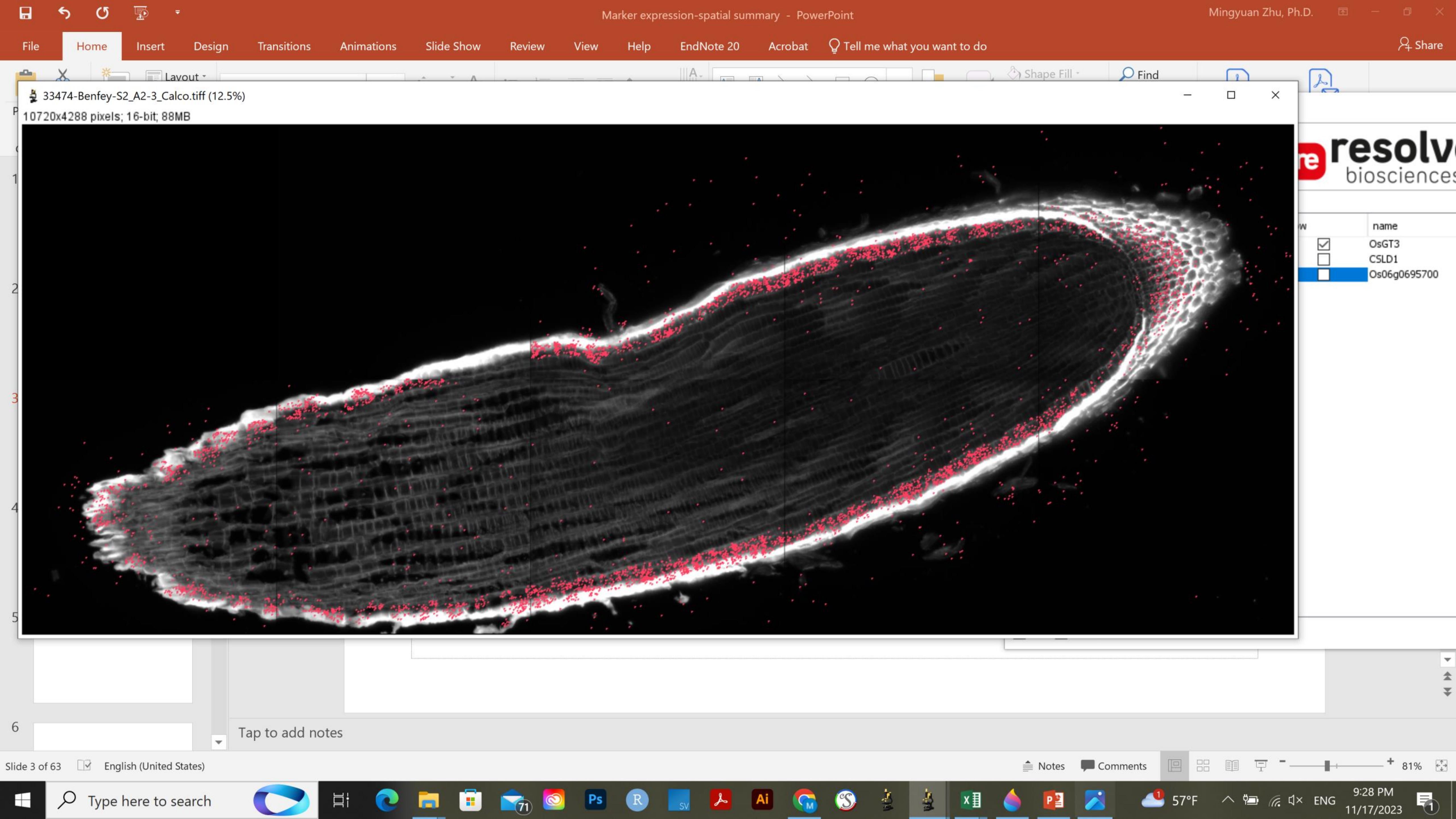
Marker expression-spatial summary - PowerPoint
Mingyuan Zhu, Ph.D.
File
Home
Insert
Design
Transitions
Animations
Slide Show
Review
View
Help
EndNote 20
Acrobat
Tell me what you want to do
Share
Layout
Shape Fill
Find
33474-Benfey-S2_A2-3_Calco.tiff (12.5%)
10720x4288 pixels; 16-bit; 88MB
resolve biosciences
name
OsGT3
CSLD1
Os06g0695700
Tap to add notes
Slide 3 of 63
English (United States)
Notes
Comments
81%
Type here to search
57°F
ENG
9:28 PM
11/17/2023

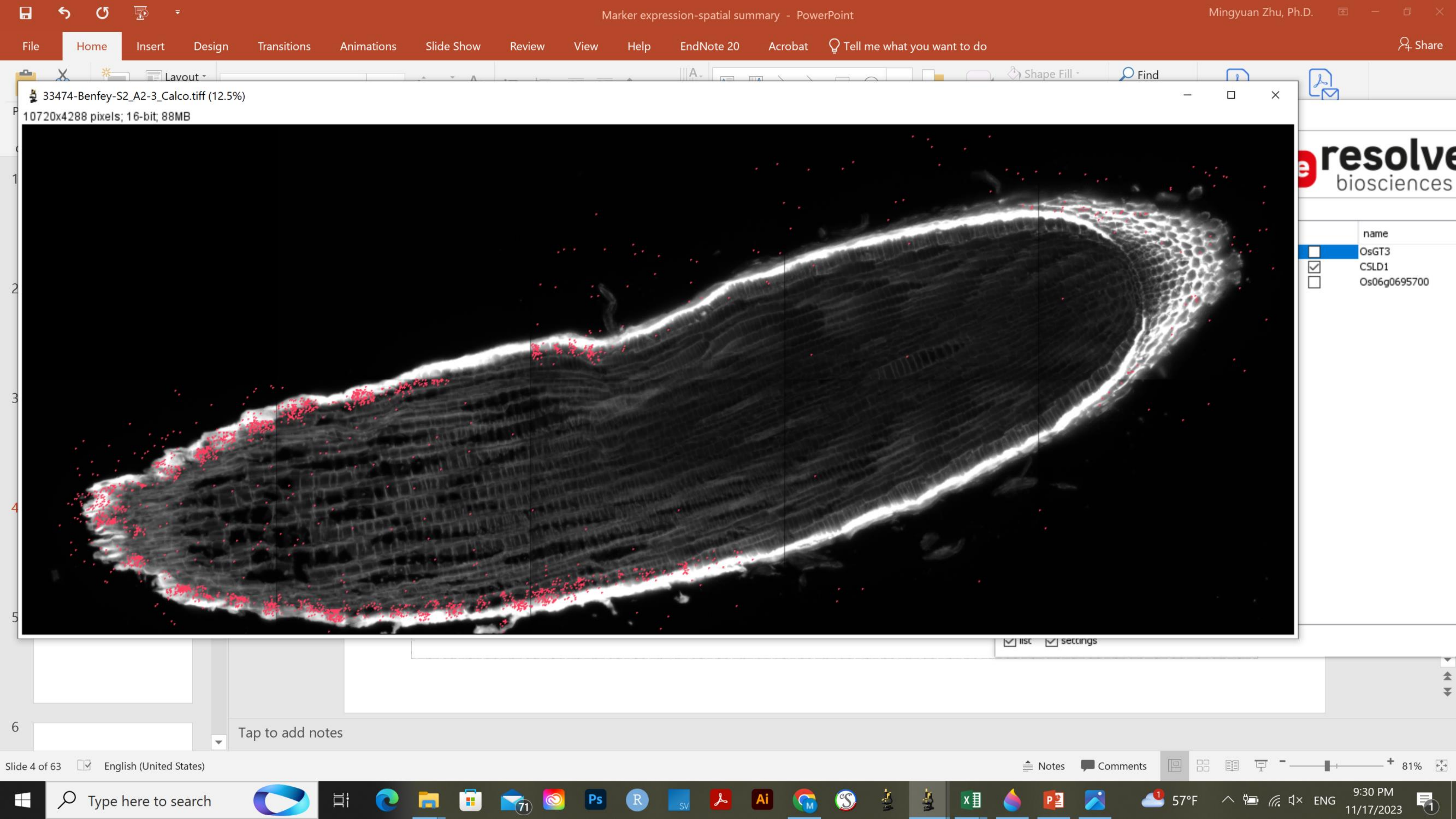

Marker expression-spatial summary - PowerPoint
Mingyuan Zhu, Ph.D.
File
Home
Insert
Design
Transitions
Animations
Slide Show
Review
View
Help
EndNote 20
Acrobat
Tell me what you want to do
Share
Layout
Shape Fill
Find
33474-Benfey-S2_A2-3_Calco.tiff (12.5%)
10720x4288 pixels; 16-bit; 88MB
resolve biosciences
name
OsGT3
CSLD1
Os06g0695700
list
settings
Tap to add notes
Slide 4 of 63
English (United States)
Notes
Comments
81%
Type here to search
57°F
ENG
9:30 PM
11/17/2023

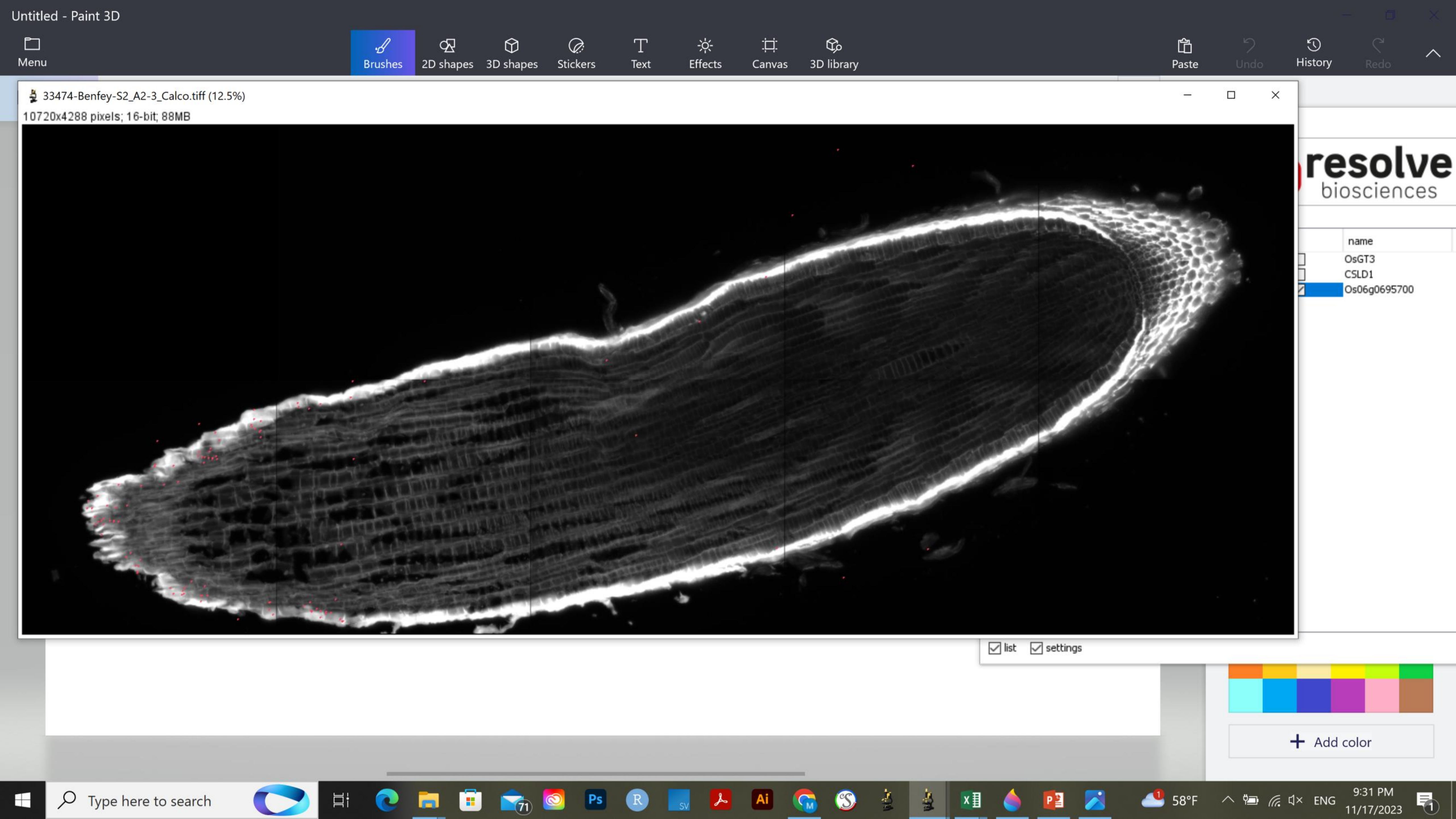
Untitled - Paint 3D
Menu
Brushes
2D shapes
3D shapes
Stickers
Text
Effects
Canvas
3D library
Paste
Undo
History
Redo
33474-Benfey-S2_A2-3_Calco.tiff (12.5%)
10720x4288 pixels; 16-bit; 88MB
resolve
biosciences
name
OsGT3
CSLD1
Os06g0695700
list
settings
Add color
Type here to search
58°F
ENG
9:31 PM
11/17/2023

Combined cell type marker expressions

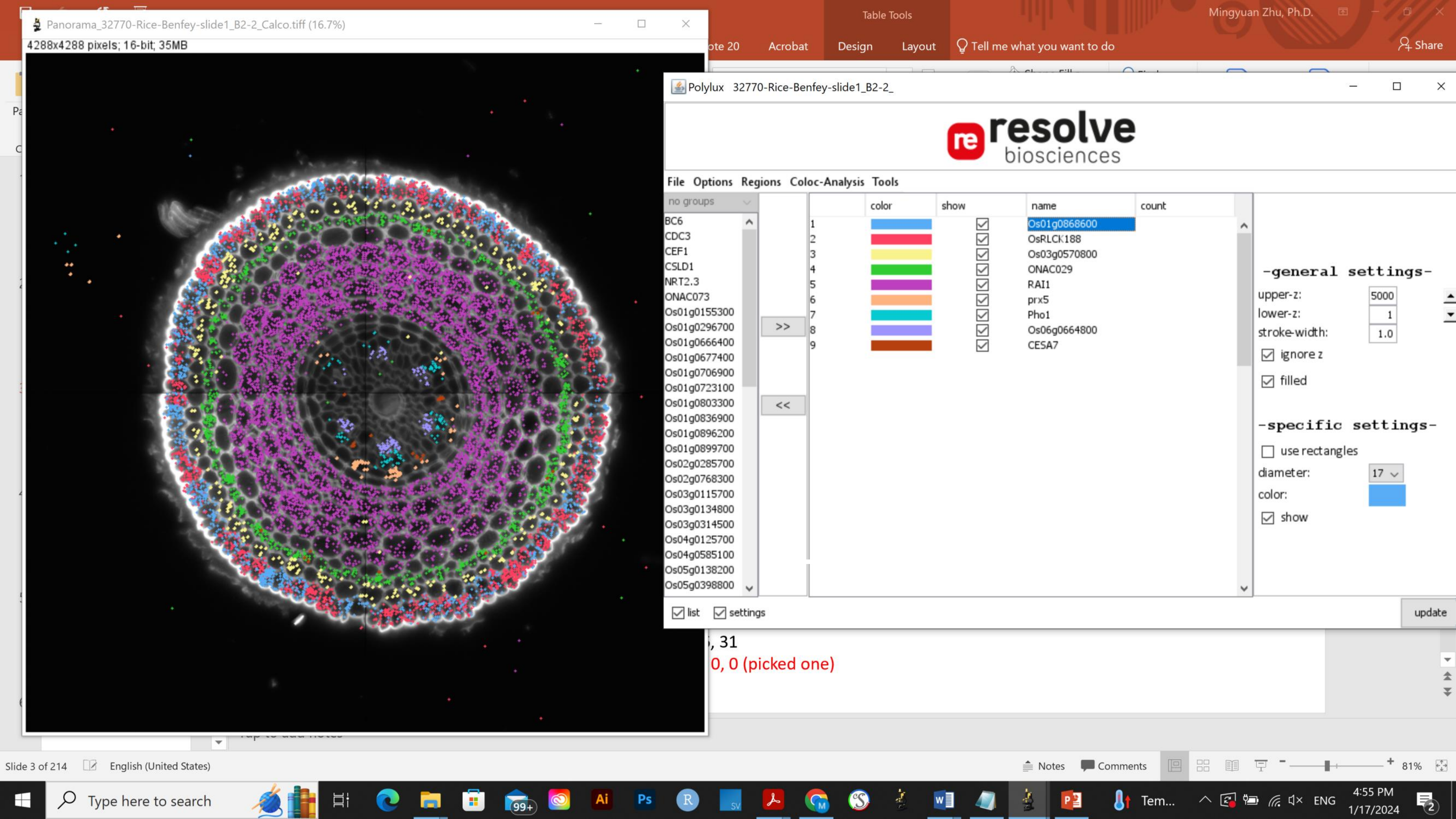

Panorama_32770-Rice-Benfey-slide1_B2-2_Calco.tiff (16.7%)
4288x4288 pixels; 16-bit; 35MB
Table Tools
Mingyuan Zhu, Ph.D.
Acrobat
Design
Layout
Tell me what you want to do
Share
Polylux 32770-Rice-Benfey-slide1_B2-2_
resolve biosciences
File Options Regions Coloc-Analysis Tools
no groups
BC6
CDC3
CEF1
CSLD1
NRT2.3
ONAC073
Os01g0155300
Os01g0296700
Os01g0666400
Os01g0677400
Os01g0706900
Os01g0723100
Os01g0803300
Os01g0836900
Os01g0896200
Os01g0899700
Os02g0285700
Os02g0768300
Os03g0115700
Os03g0134800
Os03g0314500
Os04g0125700
Os04g0585100
Os05g0138200
Os05g0398800
>>
<<
color
show
name
count
1 Os01g0868600
2 OsRLCK188
3 Os03g0570800
4 ONAC029
5 RAI1
6 prx5
7 Pho1
8 Os06g0664800
9 CESA7
-general settings-
upper-z: 5000
lower-z: 1
stroke-width: 1.0
ignore z
filled
-specific settings-
use rectangles
diameter: 17
color:
show
list
settings
update
, 31
0, 0 (picked one)
Slide 3 of 214
English (United States)
Notes
Comments
81%
Type here to search
4:55 PM
1/17/2024

Epidermis

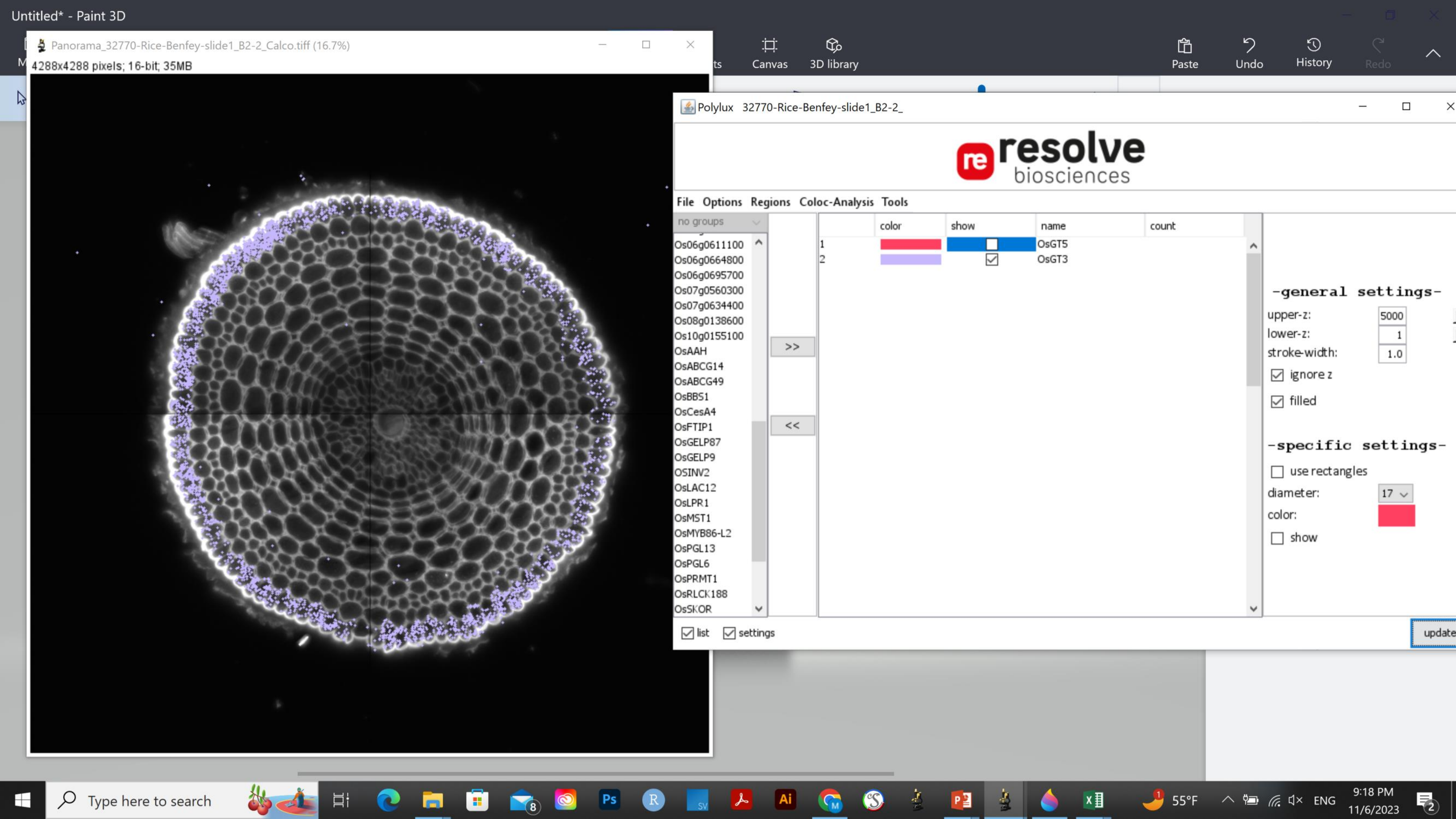
Untitled* - Paint 3D
Panorama_32770-Rice-Benfey-slide1_B2-2_Calco.tiff (16.7%)
4288x4288 pixels; 16-bit; 35MB
Canvas
3D library
Paste
Undo
History
Redo
Polylux 32770-Rice-Benfey-slide1_B2-2_
resolve biosciences
File Options Regions Coloc-Analysis Tools
no groups
Os06g0611100
Os06g0664800
Os06g0695700
Os07g0560300
Os07g0634400
Os08g0138600
Os10g0155100
OsAAH
OsABCG14
OsABCG49
OsBBS1
OsCesA4
OsFTIP1
OsGELP87
OsGELP9
OSINV2
OsLAC12
OsLPR1
OsMST1
OsMYB86-L2
OsPGL13
OsPGL6
OsPRMT1
OsRLCK188
OsSKOR
>>
<<
color
show
name
count
1 OsGT5
2 OsGT3
-general settings-
upper-z: 5000
lower-z: 1
stroke-width: 1.0
ignore z
filled
-specific settings-
use rectangles
diameter: 17
color:
show
list
settings
update
Type here to search
55°F
ENG
9:18 PM
11/6/2023

Atrichoblast

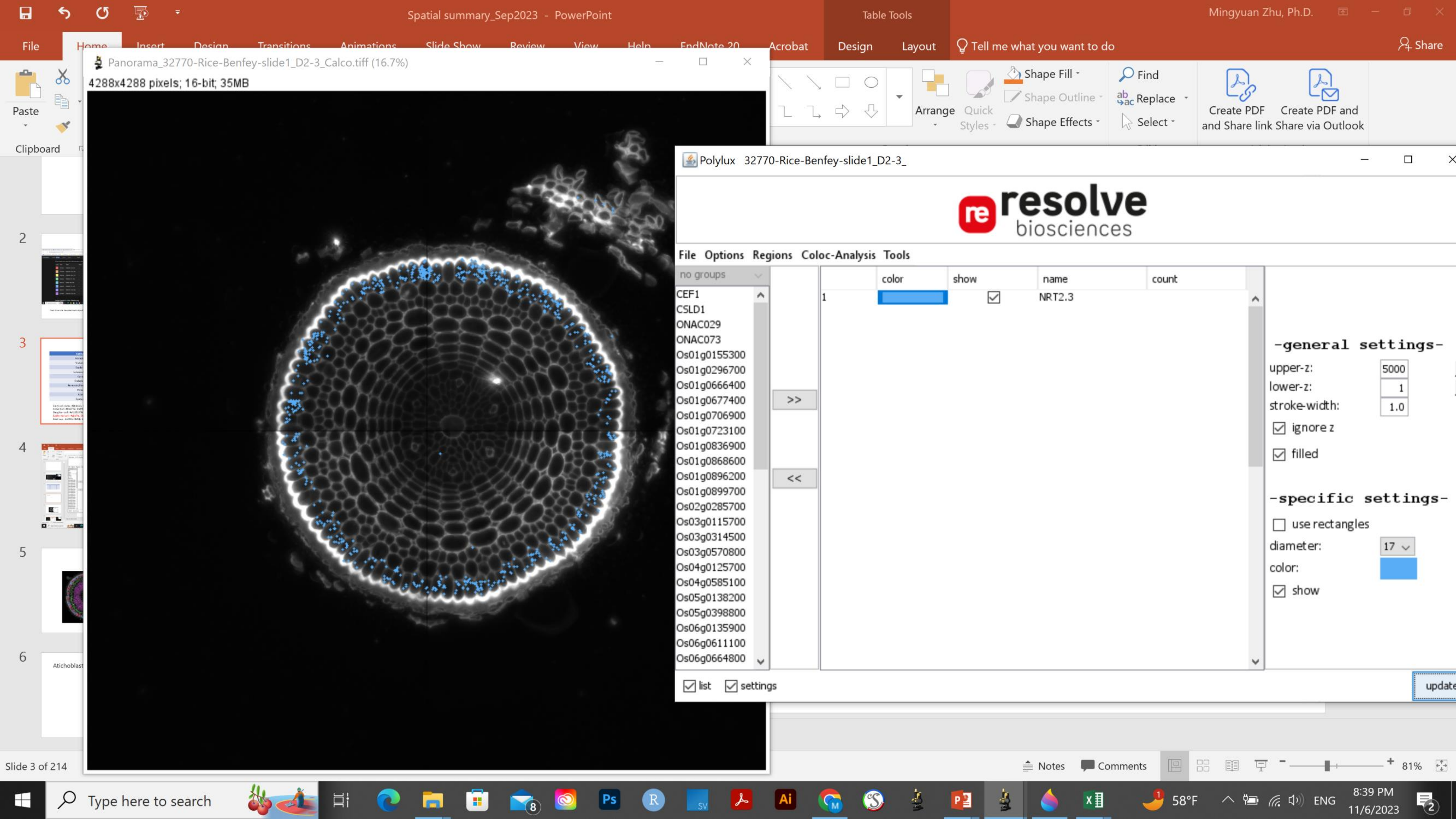
Spatial summary_Sep2023 - PowerPoint
Table Tools
Mingyuan Zhu, Ph.D.
File
Home
Insert
Design
Transitions
Animations
Slide Show
Review
View
Help
EndNote 20
Acrobat
Design
Layout
Tell me what you want to do
Share
Paste
Clipboard
Arrange
Quick Styles
Shape Fill
Shape Outline
Shape Effects
Find
Replace
Select
Create PDF and Share link
Create PDF and Share via Outlook
Panorama_32770-Rice-Benfey-slide1_D2-3_Calco.tiff (16.7%)
4288x4288 pixels; 16-bit; 35MB
Polylux 32770-Rice-Benfey-slide1_D2-3_
resolve biosciences
File
Options
Regions
Coloc-Analysis
Tools
no groups
CEF1
CSLD1
ONAC029
ONAC073
Os01g0155300
Os01g0296700
Os01g0666400
Os01g0677400
Os01g0706900
Os01g0723100
Os01g0836900
Os01g0868600
Os01g0896200
Os01g0899700
Os02g0285700
Os03g0115700
Os03g0314500
Os03g0570800
Os04g0125700
Os04g0585100
Os05g0138200
Os05g0398800
Os06g0135900
Os06g0611100
Os06g0664800
>>
<<
color
show
name
count
1
NRT2.3
-general settings-
upper-z:
5000
lower-z:
1
stroke-width:
1.0
ignore z
filled
-specific settings-
use rectangles
diameter:
17
color:
show
list
settings
update
Slide 3 of 214
Notes
Comments
81%
Type here to search
58°F
ENG
8:39 PM
11/6/2023

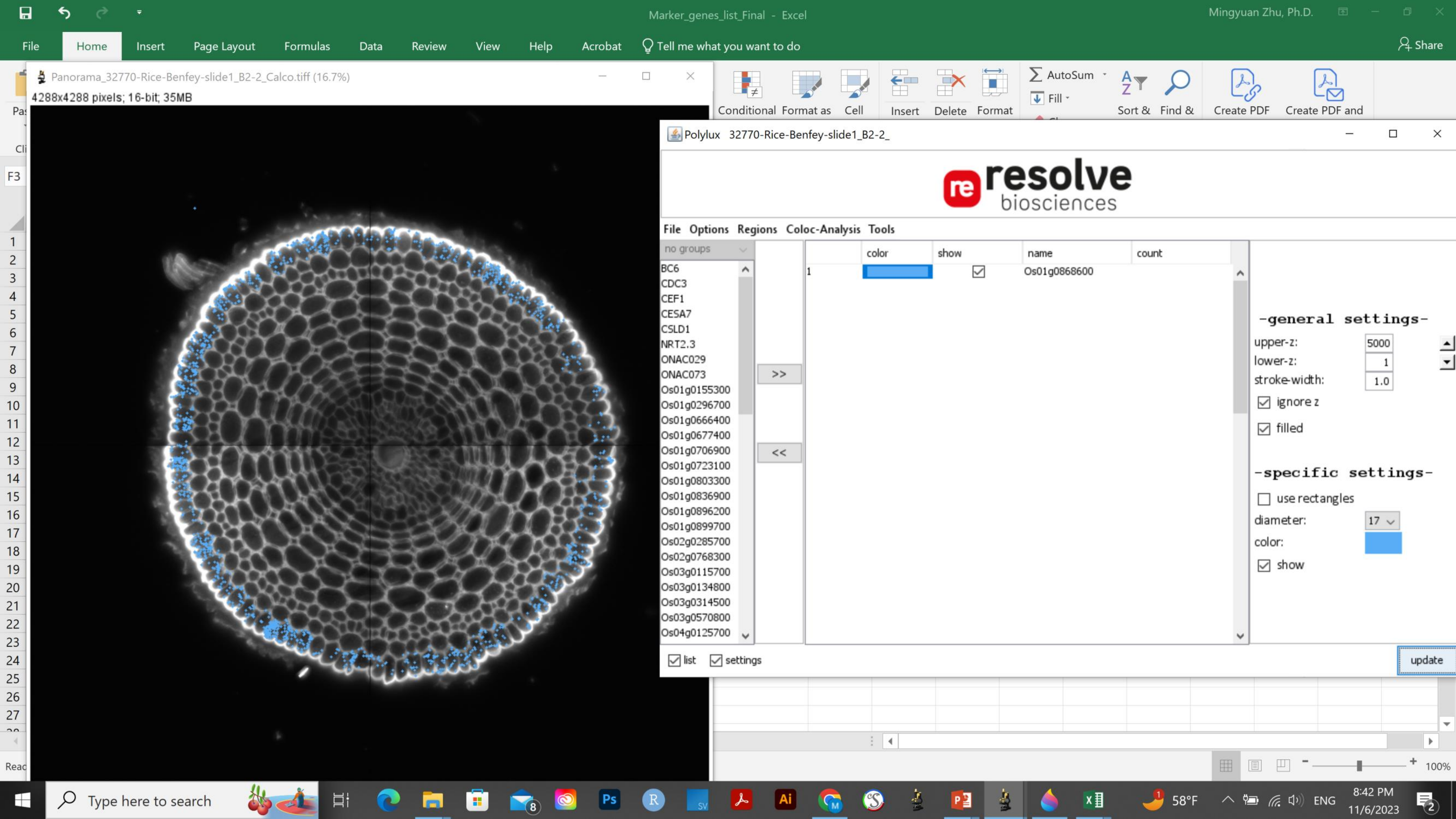
Marker_genes_list_Final - Excel
Mingyuan Zhu, Ph.D.
File
Home
Insert
Page Layout
Formulas
Data
Review
View
Help
Acrobat
Tell me what you want to do
Share
Panorama_32770-Rice-Benfey-slide1_B2-2_Calco.tiff (16.7%)
4288x4288 pixels; 16-bit; 35MB
Conditional
Format as
Cell
Insert
Delete
Format
AutoSum
Fill
Sort &
Find &
Create PDF
Create PDF and
Polylux 32770-Rice-Benfey-slide1_B2-2_
resolve
biosciences
File Options Regions Coloc-Analysis Tools
no groups
BC6
CDC3
CEF1
CESA7
CSLD1
NRT2.3
ONAC029
ONAC073
Os01g0155300
Os01g0296700
Os01g0666400
Os01g0677400
Os01g0706900
Os01g0723100
Os01g0803300
Os01g0836900
Os01g0896200
Os01g0899700
Os02g0285700
Os02g0768300
Os03g0115700
Os03g0134800
Os03g0314500
Os03g0570800
Os04g0125700
>>
<<
color
show
name
count
1
Os01g0868600
-general settings-
upper-z: 5000
lower-z: 1
stroke-width: 1.0
ignore z
filled
-specific settings-
use rectangles
diameter: 17
color:
show
list
settings
update
Type here to search
58°F
ENG
8:42 PM
11/6/2023
100%

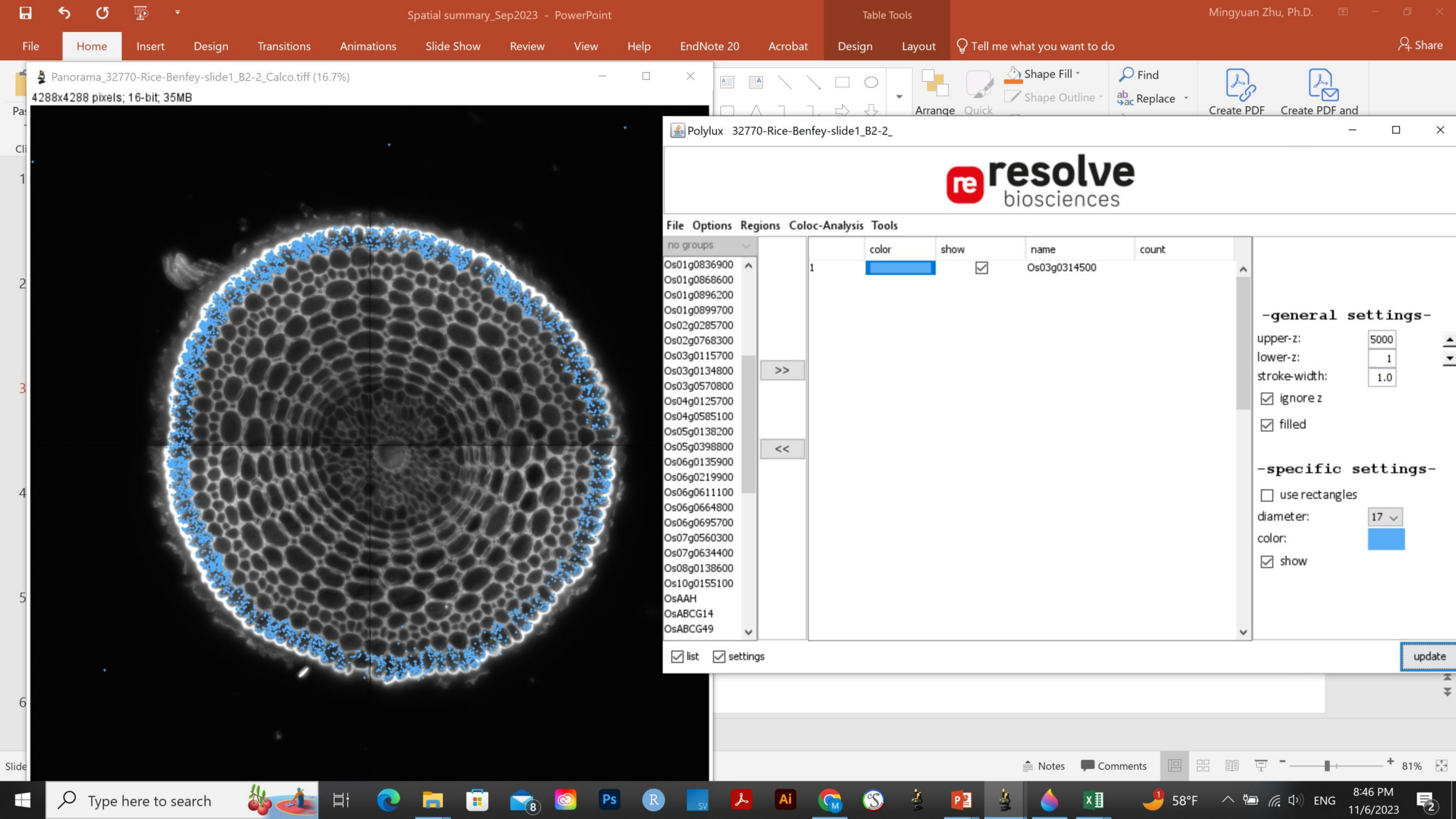
Spatial summary_Sep2023 - PowerPoint
Table Tools
Mingyuan Zhu, Ph.D.
File Home Insert Design Transitions Animations Slide Show Review View Help EndNote 20 Acrobat Design Layout
Tell me what you want to do
Share
Panorama_32770-Rice-Benfey-slide1_B2-2_Calco.tiff (16.7%)
4288x4288 pixels; 16-bit; 35MB
Shape Fill
Shape Outline
Arrange
Find
Replace
Create PDF
Create PDF and
Polylux 32770-Rice-Benfey-slide1_B2-2_
resolve biosciences
File Options Regions Coloc-Analysis Tools
no groups
Os01g0836900
Os01g0868600
Os01g0896200
Os01g0899700
Os02g0285700
Os02g0768300
Os03g0115700
Os03g0134800
Os03g0570800
Os04g0125700
Os04g0585100
Os05g0138200
Os05g0398800
Os06g0135900
Os06g0219900
Os06g0611100
Os06g0664800
Os06g0695700
Os07g0560300
Os07g0634400
Os08g0138600
Os10g0155100
OsAAH
OsABCG14
OsABCG49
>>
<<
color show name count
1 Os03g0314500
-general settings-
upper-z: 5000
lower-z: 1
stroke-width: 1.0
ignore z
filled
-specific settings-
use rectangles
diameter: 17
color:
show
list
settings
update
Notes
Comments
81%
Type here to search
58°F
ENG
8:46 PM
11/6/2023

Trichoblast

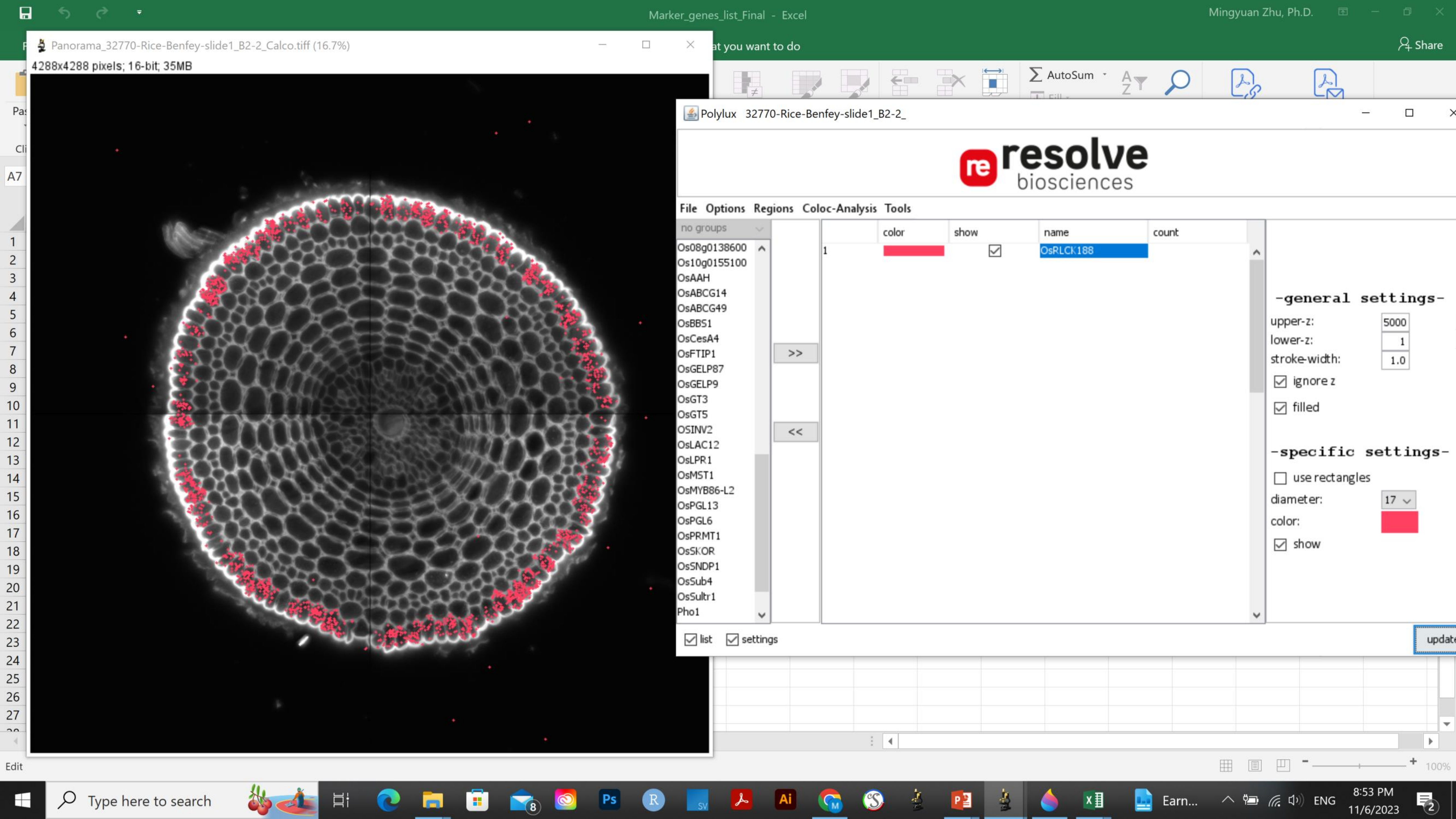
Marker_genes_list_Final - Excel
Mingyuan Zhu, Ph.D.
Share
Panorama_32770-Rice-Benfey-slide1_B2-2_Calco.tiff (16.7%)
4288x4288 pixels; 16-bit; 35MB
Polylux 32770-Rice-Benfey-slide1_B2-2_
resolve biosciences
File Options Regions Coloc-Analysis Tools
no groups
Os08g0138600
Os10g0155100
OsAAH
OsABCG14
OsABCG49
OsBBS1
OsCesA4
OsFTIP1
OsGELP87
OsGELP9
OsGT3
OsGT5
OSINV2
OsLAC12
OsLPR1
OsMST1
OsMYB86-L2
OsPGL13
OsPGL6
OsPRMT1
OsSKOR
OsSNDP1
OsSub4
OsSultr1
Pho1
>>
<<
color
show
name
count
1
OsRLCK188
-general settings-
upper-z: 5000
lower-z: 1
stroke-width: 1.0
ignore z
filled
-specific settings-
use rectangles
diameter: 17
color:
show
list
settings
update
AutoSum
Type here to search
8:53 PM
11/6/2023
ENG

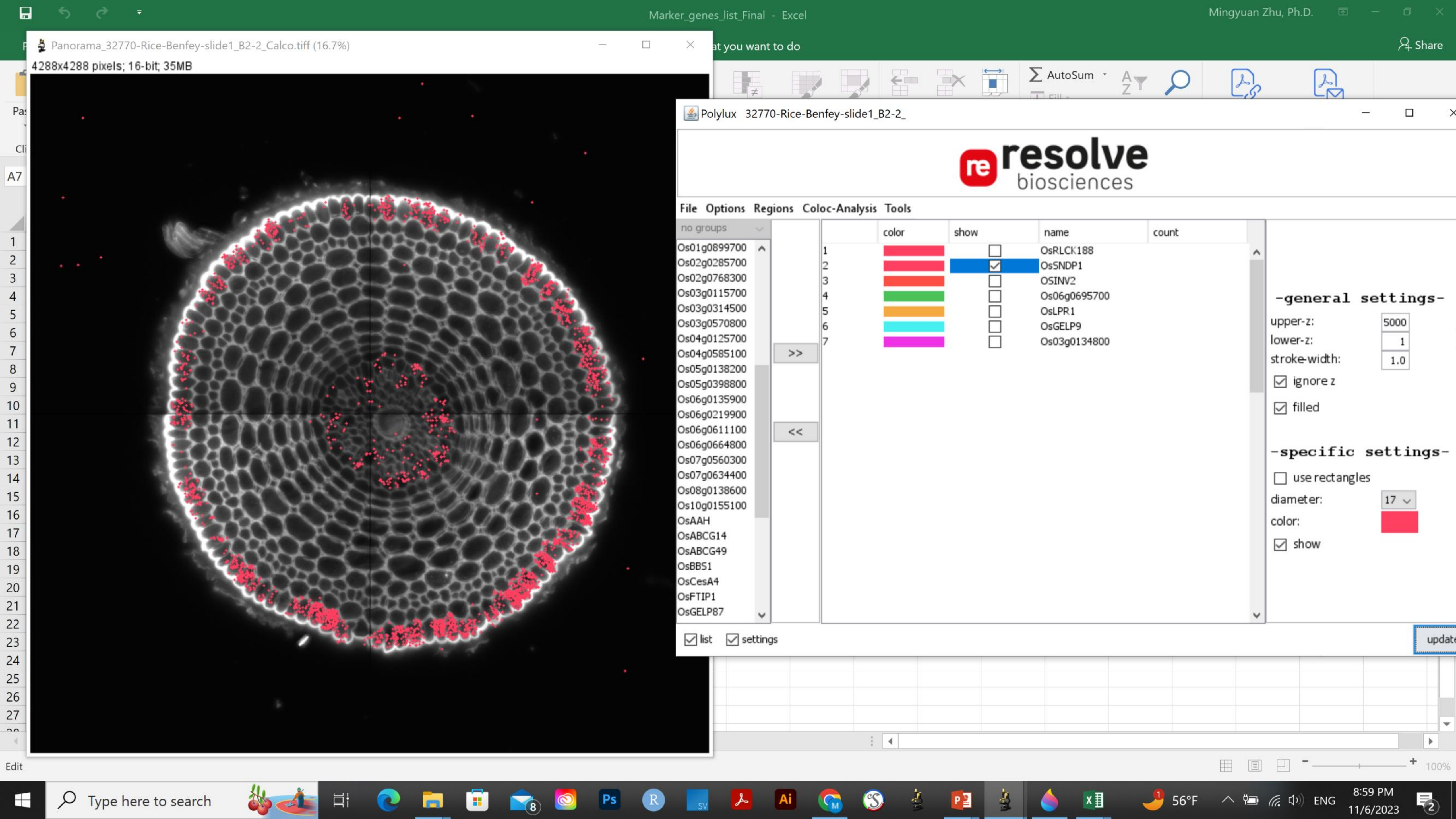
Marker_genes_list_Final - Excel
Mingyuan Zhu, Ph.D.
Share
Panorama_32770-Rice-Benfey-slide1_B2-2_Calco.tiff (16.7%)
4288x4288 pixels; 16-bit; 35MB
Polylux 32770-Rice-Benfey-slide1_B2-2_
resolve biosciences
File Options Regions Coloc-Analysis Tools
no groups
Os01g0899700
Os02g0285700
Os02g0768300
Os03g0115700
Os03g0314500
Os03g0570800
Os04g0125700
Os04g0585100
Os05g0138200
Os05g0398800
Os06g0135900
Os06g0219900
Os06g0611100
Os06g0664800
Os07g0560300
Os07g0634400
Os08g0138600
Os10g0155100
OsAAH
OsABCG14
OsABCG49
OsBBS1
OsCesA4
OsFTIP1
OsGELP87
color show name count
1 OsRLCK188
2 OsSNDP1
3 OSINV2
4 Os06g0695700
5 OsLPR1
6 OsGELP9
7 Os03g0134800
-general settings-
upper-z: 5000
lower-z: 1
stroke-width: 1.0
ignore z
filled
-specific settings-
use rectangles
diameter: 17
color:
show
list
settings
update
AutoSum
Type here to search
56°F
ENG
8:59 PM
11/6/2023

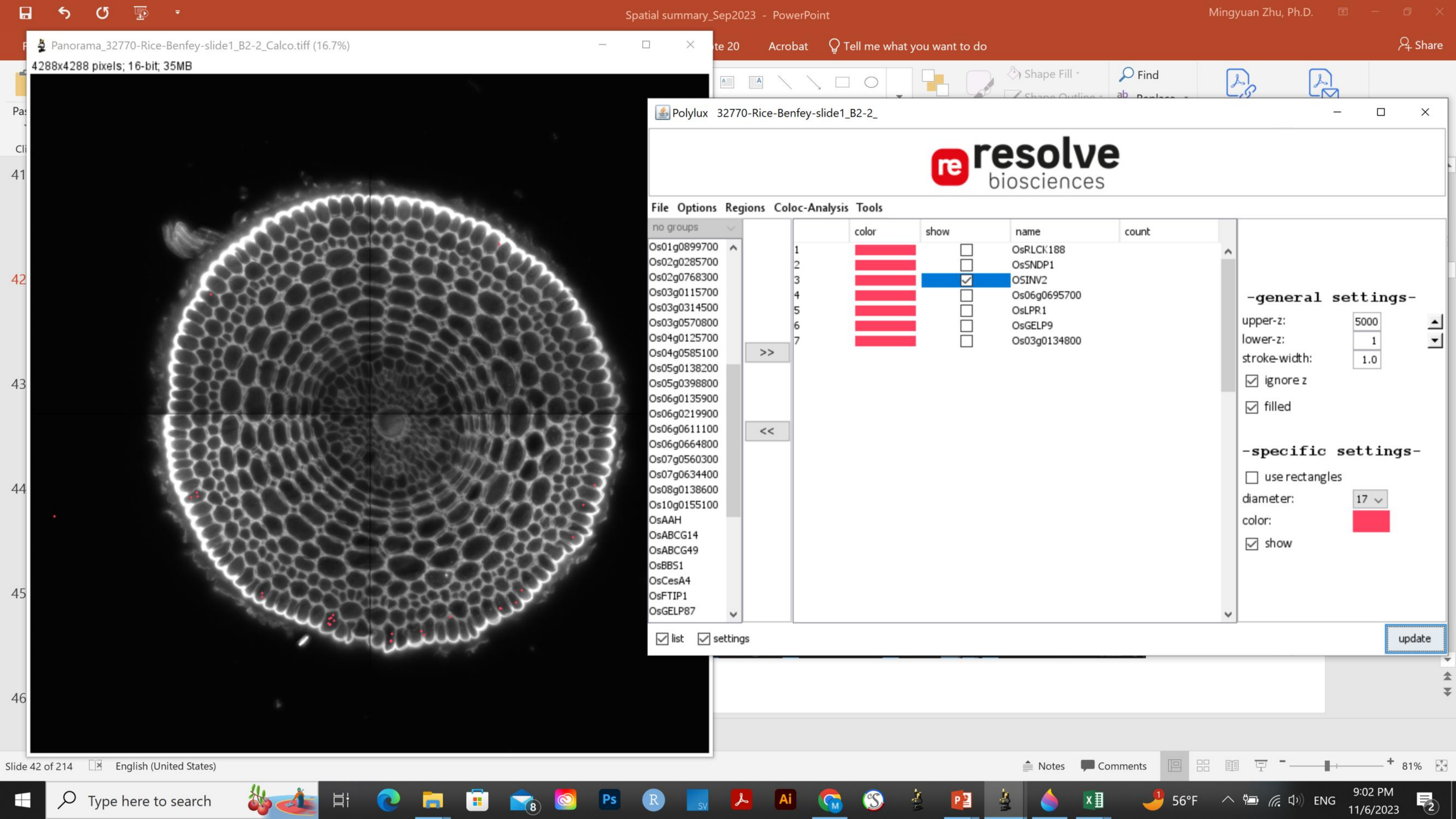

Spatial summary_Sep2023 - PowerPoint
Mingyuan Zhu, Ph.D.
Panorama_32770-Rice-Benfey-slide1_B2-2_Calco.tiff (16.7%)
4288x4288 pixels; 16-bit; 35MB
Polylux 32770-Rice-Benfey-slide1_B2-2_
resolve biosciences
File Options Regions Coloc-Analysis Tools
no groups
Os01g0899700
Os02g0285700
Os02g0768300
Os03g0115700
Os03g0314500
Os03g0570800
Os04g0125700
Os04g0585100
Os05g0138200
Os05g0398800
Os06g0135900
Os06g0219900
Os06g0611100
Os06g0664800
Os07g0560300
Os07g0634400
Os08g0138600
Os10g0155100
OsAAH
OsABCG14
OsABCG49
OsBBS1
OsCesA4
OsFTIP1
OsGELP87
>>
<<
color show name count
1 OsRLCK188
2 OsSNDP1
3 OSINV2
4 Os06g0695700
5 OsLPR1
6 OsGELP9
7 Os03g0134800
-general settings-
upper-z: 5000
lower-z: 1
stroke-width: 1.0
ignore z
filled
-specific settings-
use rectangles
diameter: 17
color:
show
list settings
update
Slide 42 of 214
English (United States)
Notes Comments 81%
Type here to search
56°F
ENG
9:02 PM
11/6/2023

Marker_genes_list_Final - Excel

Mingyuan Zhu, Ph.D.

Panorama_32770-Rice-Benfey-slide1_B2-2_Calco.tiff (16.7%)

4288x4288 pixels; 16-bit; 35MB

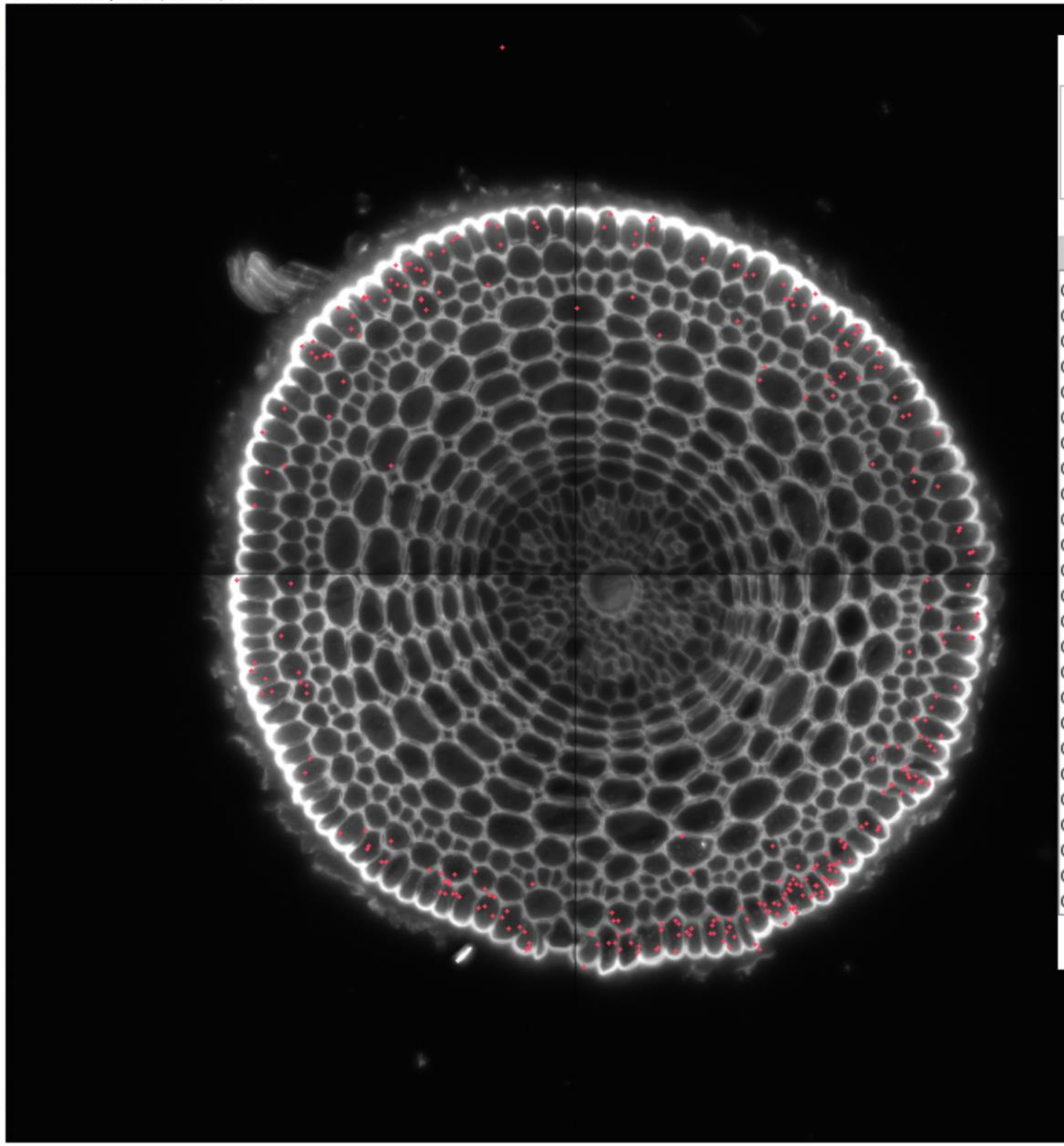

Polylux 32770-Rice-Benfey-slide1_B2-2_

resolve biosciences

File Options Regions Coloc-Analysis Tools

no groups

Os06g0611100
Os06g0664800
Os06g0695700
Os07g0560300
Os07g0634400
Os08g0138600
Os10g0155100
OsAAH
OsABCG14
OsABCG49
OsBBS1
OsCesA4
OsFTIP1
OsGELP87
OsGELP9
OSINV2
OsLAC12
OsLPR1
OsMST1
OsMYB86-L2
OsPGL13
OsPGL6
OsPRMT1
OsRLCK188
OsSKOR

>>

<<

	color	show	name	count
1		☑	OsGT5	
2		☐	OsGT3	

-general settings-

upper-z: 5000

lower-z: 1

stroke-width: 1.0

☑ ignore z

☑ filled

-specific settings-

☐ use rectangles

diameter: 17

color:

☑ show

☑ list ☑ settings

update

Edit

Type here to search

56°F ENG 9:16 PM 11/6/2023

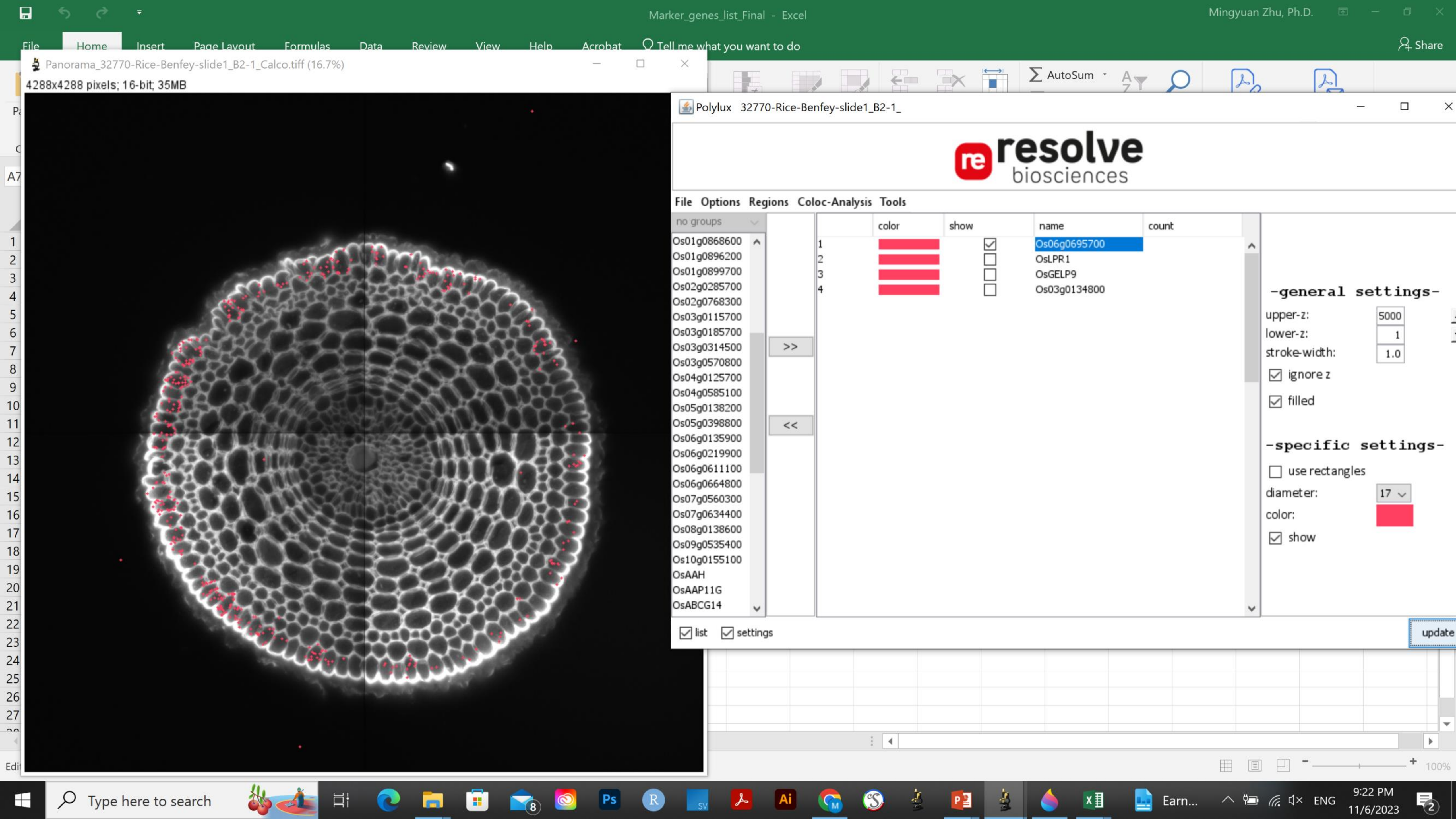
Marker_genes_list_Final - Excel
Mingyuan Zhu, Ph.D.
File
Home
Insert
Page Layout
Formulas
Data
Review
View
Help
Acrobat
Tell me what you want to do
Share
AutoSum
Panorama_32770-Rice-Benfey-slide1_B2-1_Calco.tiff (16.7%)
4288x4288 pixels; 16-bit; 35MB
Polylux 32770-Rice-Benfey-slide1_B2-1_
resolve biosciences
File
Options
Regions
Coloc-Analysis
Tools
no groups
Os01g0868600
Os01g0896200
Os01g0899700
Os02g0285700
Os02g0768300
Os03g0115700
Os03g0185700
Os03g0314500
Os03g0570800
Os04g0125700
Os04g0585100
Os05g0138200
Os05g0398800
Os06g0135900
Os06g0219900
Os06g0611100
Os06g0664800
Os07g0560300
Os07g0634400
Os08g0138600
Os09g0535400
Os10g0155100
OsAAH
OsAAP11G
OsABCG14
>>
<<
color
show
name
count
1 Os06g0695700
2 OsLPR1
3 OsGELP9
4 Os03g0134800
-general settings-
upper-z: 5000
lower-z: 1
stroke-width: 1.0
ignore z
filled
-specific settings-
use rectangles
diameter: 17
color:
show
list
settings
update
Type here to search
9:22 PM
11/6/2023
ENG
Earn...

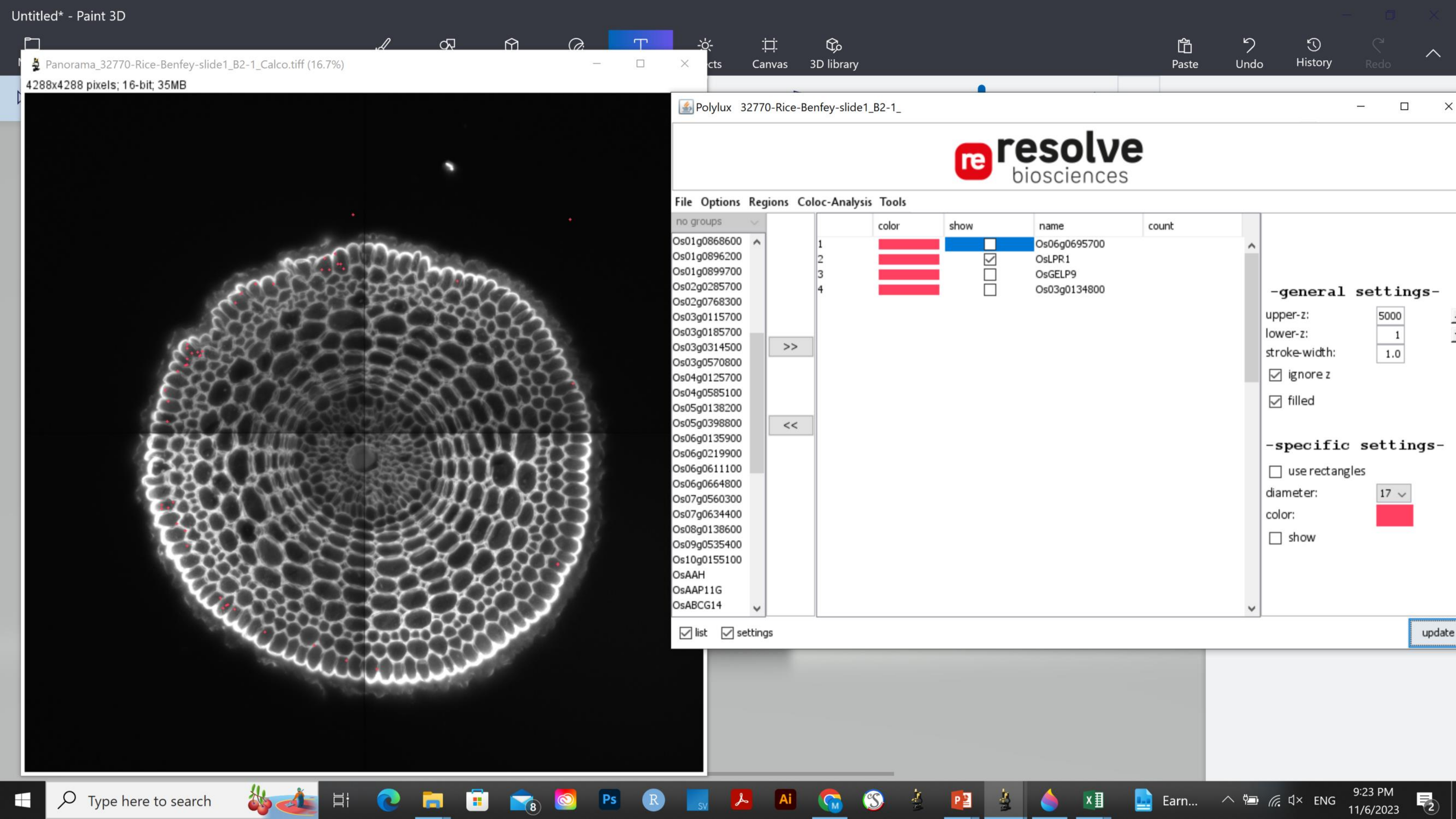
Untitled* - Paint 3D
Canvas
3D library
Paste
Undo
History
Redo
Panorama_32770-Rice-Benfey-slide1_B2-1_Calco.tiff (16.7%)
4288x4288 pixels; 16-bit; 35MB
Polylux 32770-Rice-Benfey-slide1_B2-1_
re resolve biosciences
File Options Regions Coloc-Analysis Tools
no groups
Os01g0868600
Os01g0896200
Os01g0899700
Os02g0285700
Os02g0768300
Os03g0115700
Os03g0185700
Os03g0314500
Os03g0570800
Os04g0125700
Os04g0585100
Os05g0138200
Os05g0398800
Os06g0135900
Os06g0219900
Os06g0611100
Os06g0664800
Os07g0560300
Os07g0634400
Os08g0138600
Os09g0535400
Os10g0155100
OsAAH
OsAAP11G
OsABCG14
>>
<<
color
show
name
count
1 Os06g0695700
2 OsLPR1
3 OsGELP9
4 Os03g0134800
-general settings-
upper-z: 5000
lower-z: 1
stroke-width: 1.0
ignore z
filled
-specific settings-
use rectangles
diameter: 17
color:
show
list
settings
update
Type here to search
ENG
9:23 PM
11/6/2023

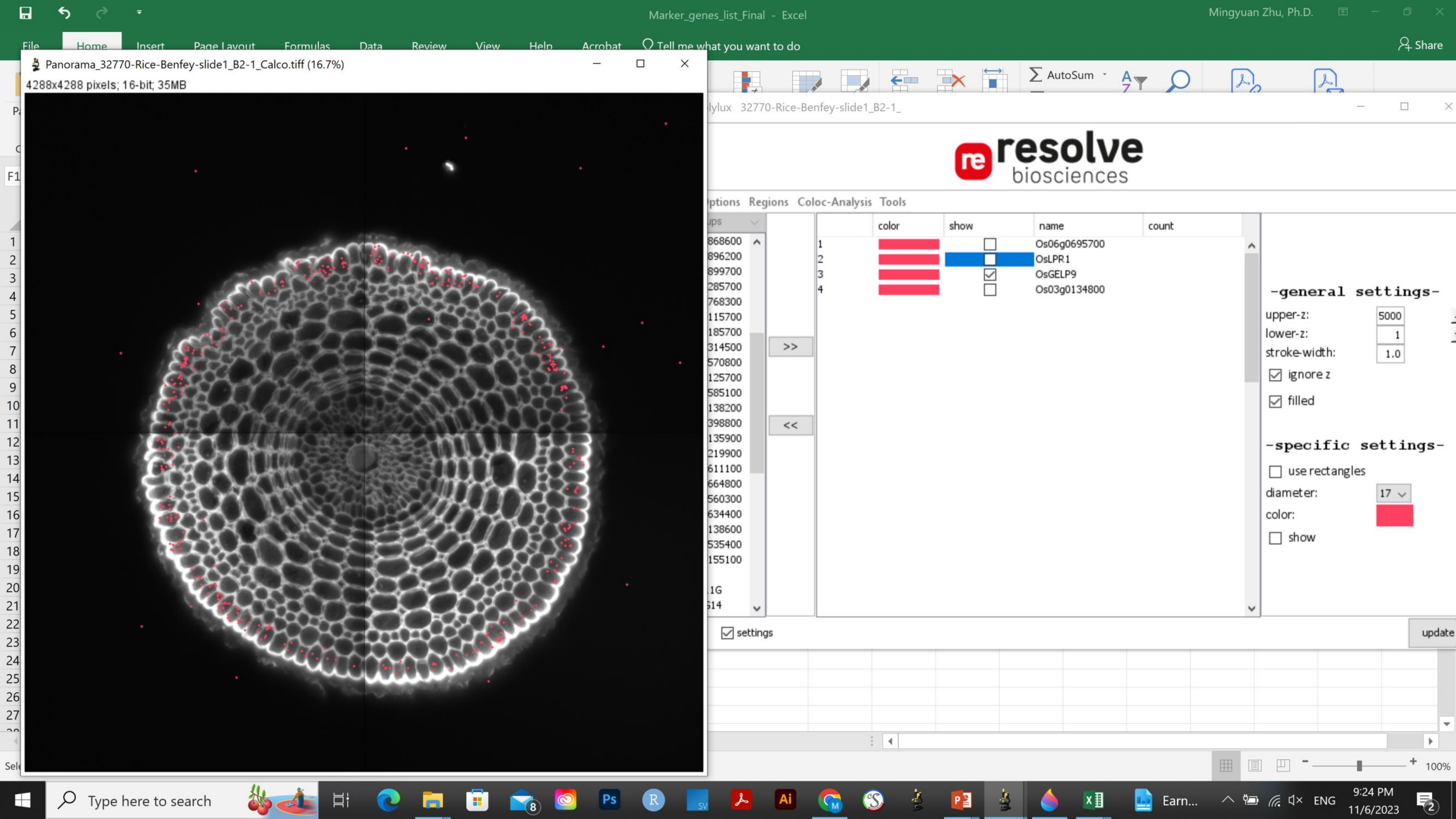
Marker_genes_list_Final - Excel
Mingyuan Zhu, Ph.D.
File
Home
Insert
Page Layout
Formulas
Data
Review
View
Help
Acrobat
Tell me what you want to do
Share
Panorama_32770-Rice-Benfey-slide1_B2-1_Calco.tiff (16.7%)
4288x4288 pixels; 16-bit; 35MB
AutoSum
lylux 32770-Rice-Benfey-slide1_B2-1_
resolve biosciences
ptions
Regions
Coloc-Analysis
Tools
color
show
name
count
1 Os06g0695700
2 OsLPR1
3 OsGELP9
4 Os03g0134800
868600
896200
899700
285700
768300
115700
185700
314500
570800
125700
585100
138200
398800
135900
219900
611100
664800
560300
634400
138600
535400
155100
1G
514
>>
<<
-general settings-
upper-z: 5000
lower-z: 1
stroke-width: 1.0
ignore z
filled
-specific settings-
use rectangles
diameter: 17
color:
show
settings
update
100%
Type here to search
Earn...
ENG
9:24 PM
11/6/2023

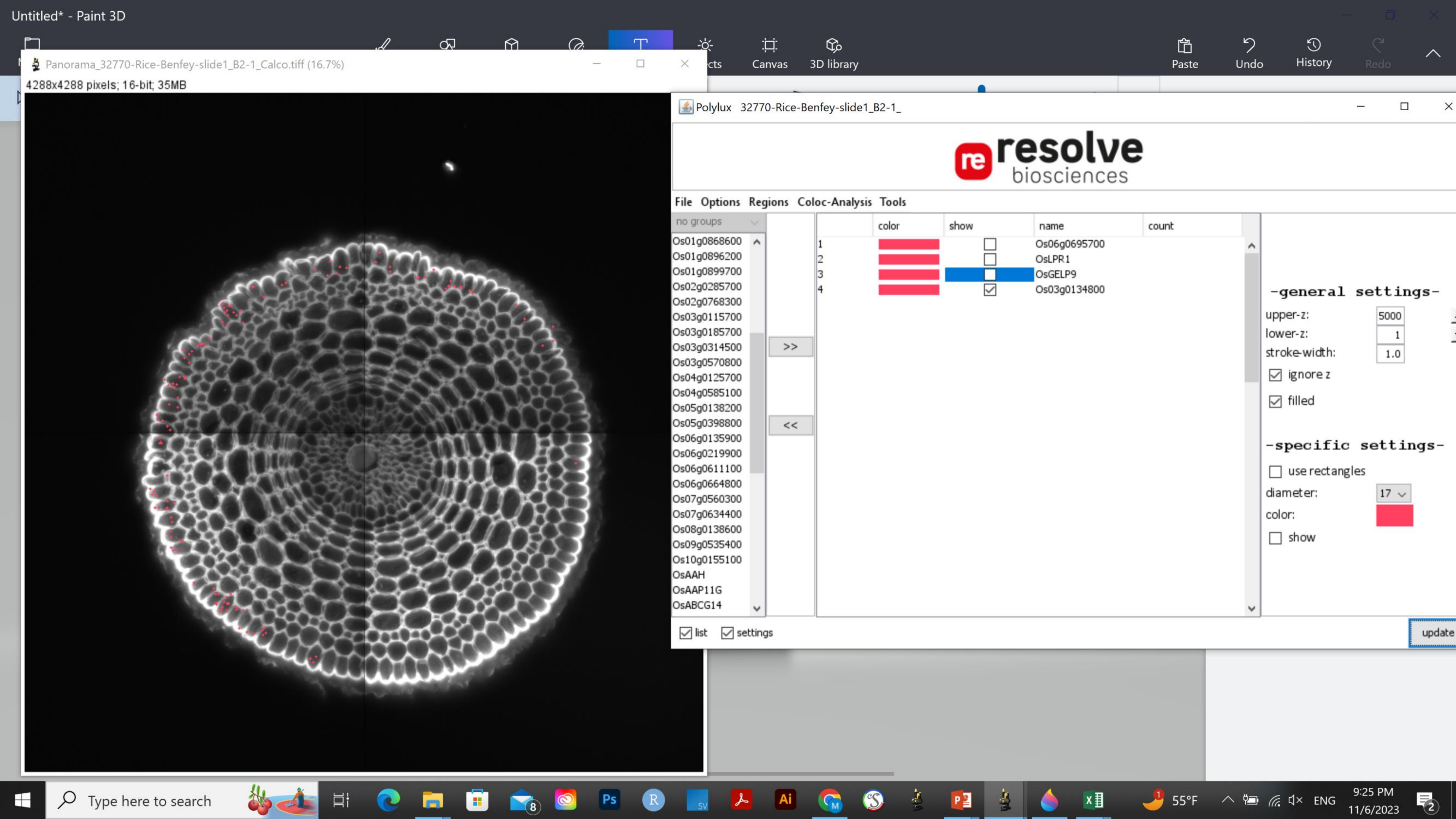
Untitled* - Paint 3D
Canvas
3D library
Paste
Undo
History
Redo
Panorama_32770-Rice-Benfey-slide1_B2-1_Calco.tiff (16.7%)
4288x4288 pixels; 16-bit; 35MB
Polylux 32770-Rice-Benfey-slide1_B2-1_
resolve biosciences
File Options Regions Coloc-Analysis Tools
no groups
Os01g0868600
Os01g0896200
Os01g0899700
Os02g0285700
Os02g0768300
Os03g0115700
Os03g0185700
Os03g0314500
Os03g0570800
Os04g0125700
Os04g0585100
Os05g0138200
Os05g0398800
Os06g0135900
Os06g0219900
Os06g0611100
Os06g0664800
Os07g0560300
Os07g0634400
Os08g0138600
Os09g0535400
Os10g0155100
OsAAH
OsAAP11G
OsABCG14
>>
<<
color show name count
1 Os06g0695700
2 OsLPR1
3 OsGELP9
4 Os03g0134800
-general settings-
upper-z: 5000
lower-z: 1
stroke-width: 1.0
ignore z
filled
-specific settings-
use rectangles
diameter: 17
color:
show
list
settings
update
Type here to search
55°F
ENG
9:25 PM
11/6/2023

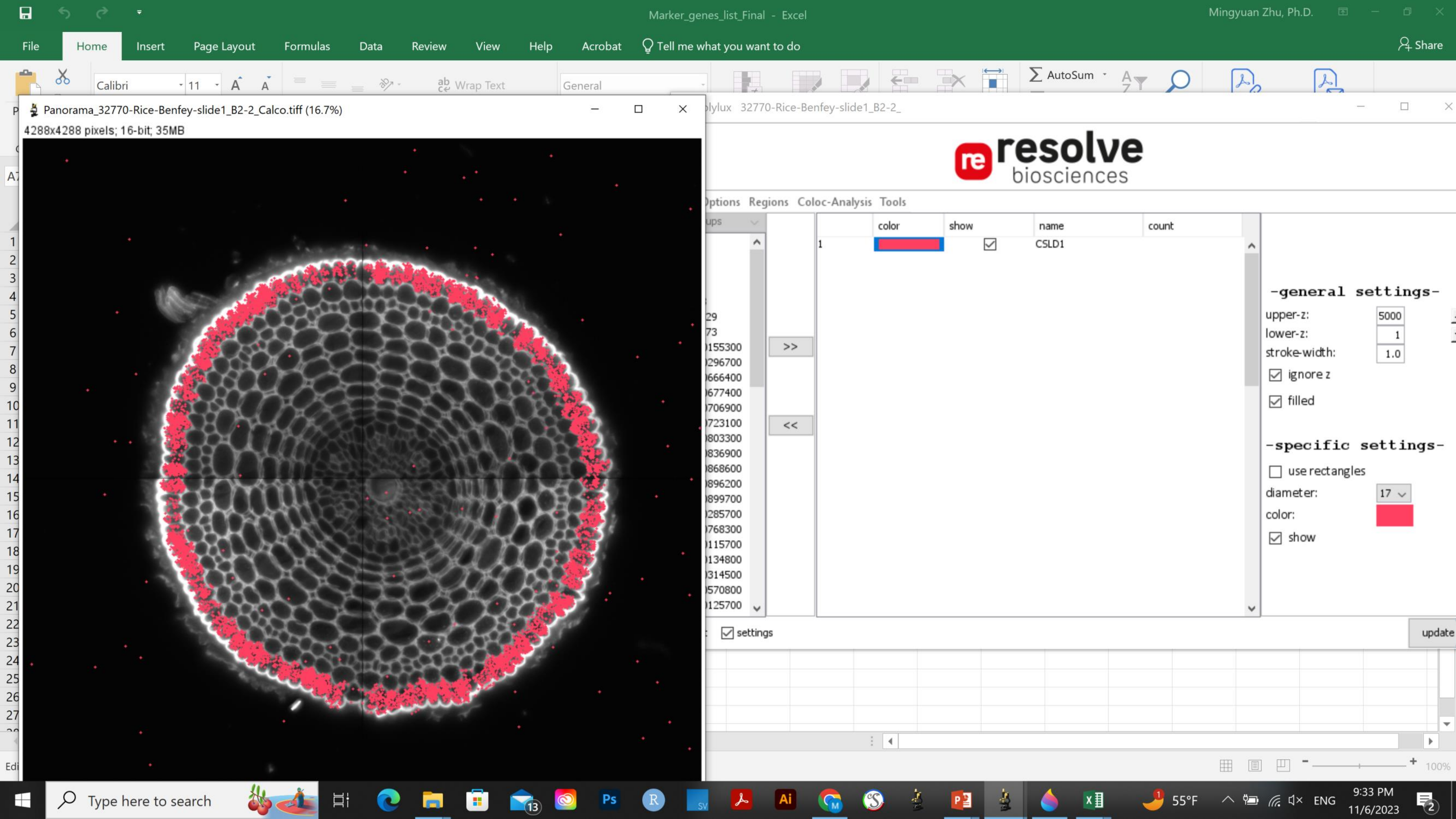

Marker_genes_list_Final - Excel
Mingyuan Zhu, Ph.D.
File
Home
Insert
Page Layout
Formulas
Data
Review
View
Help
Acrobat
Tell me what you want to do
Share
Calibri
11
Wrap Text
General
AutoSum
Panorama_32770-Rice-Benfey-slide1_B2-2_Calco.tiff (16.7%)
4288x4288 pixels; 16-bit; 35MB
olylux 32770-Rice-Benfey-slide1_B2-2_
resolve
biosciences
Options
Regions
Coloc-Analysis
Tools
color
show
name
count
1
CSLD1
-general settings-
upper-z:
5000
lower-z:
1
stroke-width:
1.0
ignore z
filled
-specific settings-
use rectangles
diameter:
17
color:
show
settings
update
Type here to search
55°F
ENG
9:33 PM
11/6/2023

Exodermis

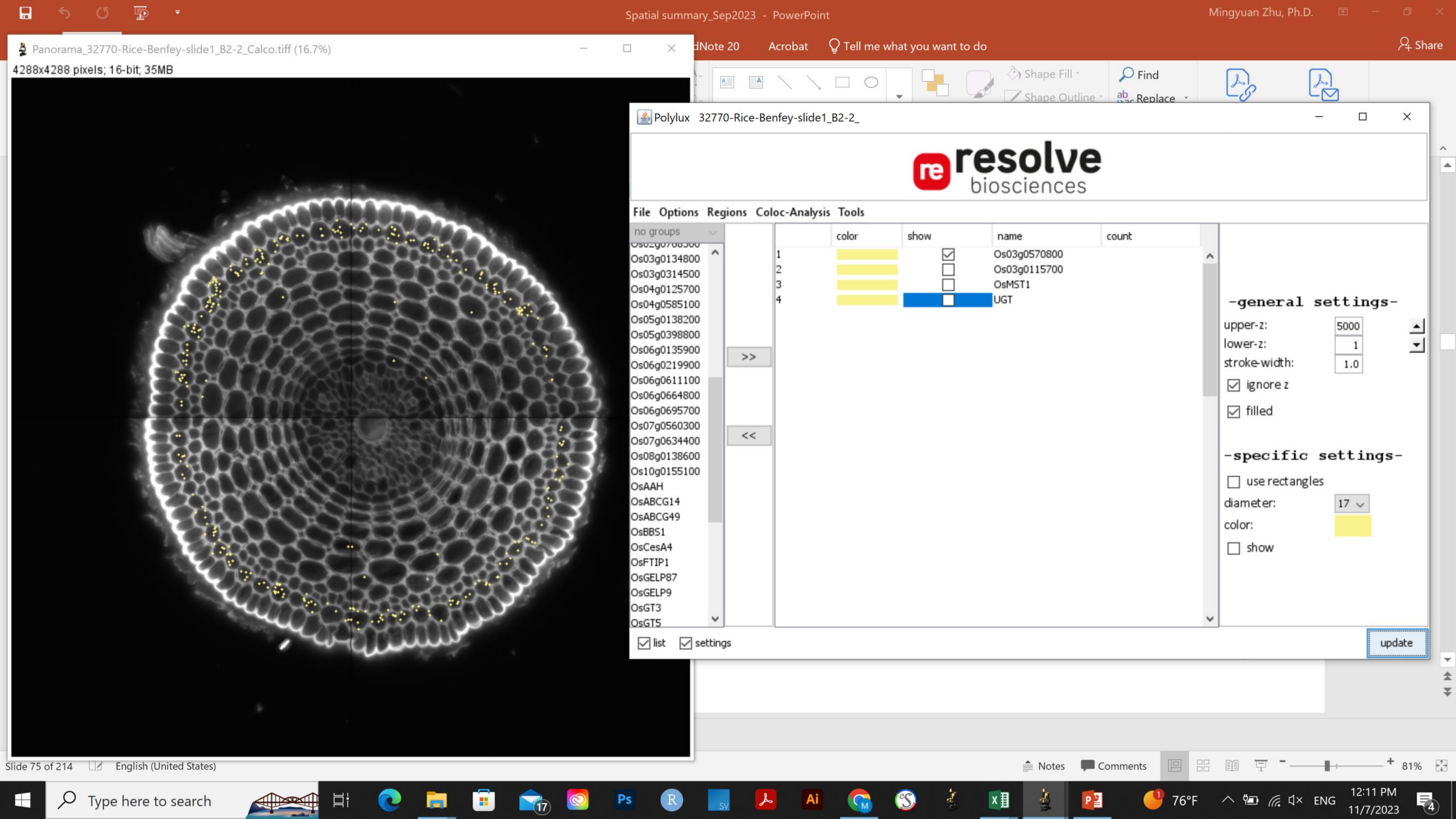
Spatial summary_Sep2023 - PowerPoint
Mingyuan Zhu, Ph.D.
Panorama_32770-Rice-Benfey-slide1_B2-2_Calco.tiff (16.7%)
4288x4288 pixels; 16-bit; 35MB
dNote 20
Acrobat
Tell me what you want to do
Share
Shape Fill
Shape Outline
Find
Replace
Polylux 32770-Rice-Benfey-slide1_B2-2_
resolve
biosciences
File
Options
Regions
Coloc-Analysis
Tools
no groups
Os03g0134800
Os03g0314500
Os04g0125700
Os04g0585100
Os05g0138200
Os05g0398800
Os06g0135900
Os06g0219900
Os06g0611100
Os06g0664800
Os06g0695700
Os07g0560300
Os07g0634400
Os08g0138600
Os10g0155100
OsAAH
OsABCG14
OsABCG49
OsBBS1
OsCesA4
OsFTIP1
OsGELP87
OsGELP9
OsGT3
OsGT5
>>
<<
color
show
name
count
1
Os03g0570800
2
Os03g0115700
3
OsMST1
4
UGT
-general settings-
upper-z:
5000
lower-z:
1
stroke-width:
1.0
ignore z
filled
-specific settings-
use rectangles
diameter:
17
color:
show
list
settings
update
Slide 75 of 214
English (United States)
Notes
Comments
81%
Type here to search
76°F
ENG
12:11 PM
11/7/2023

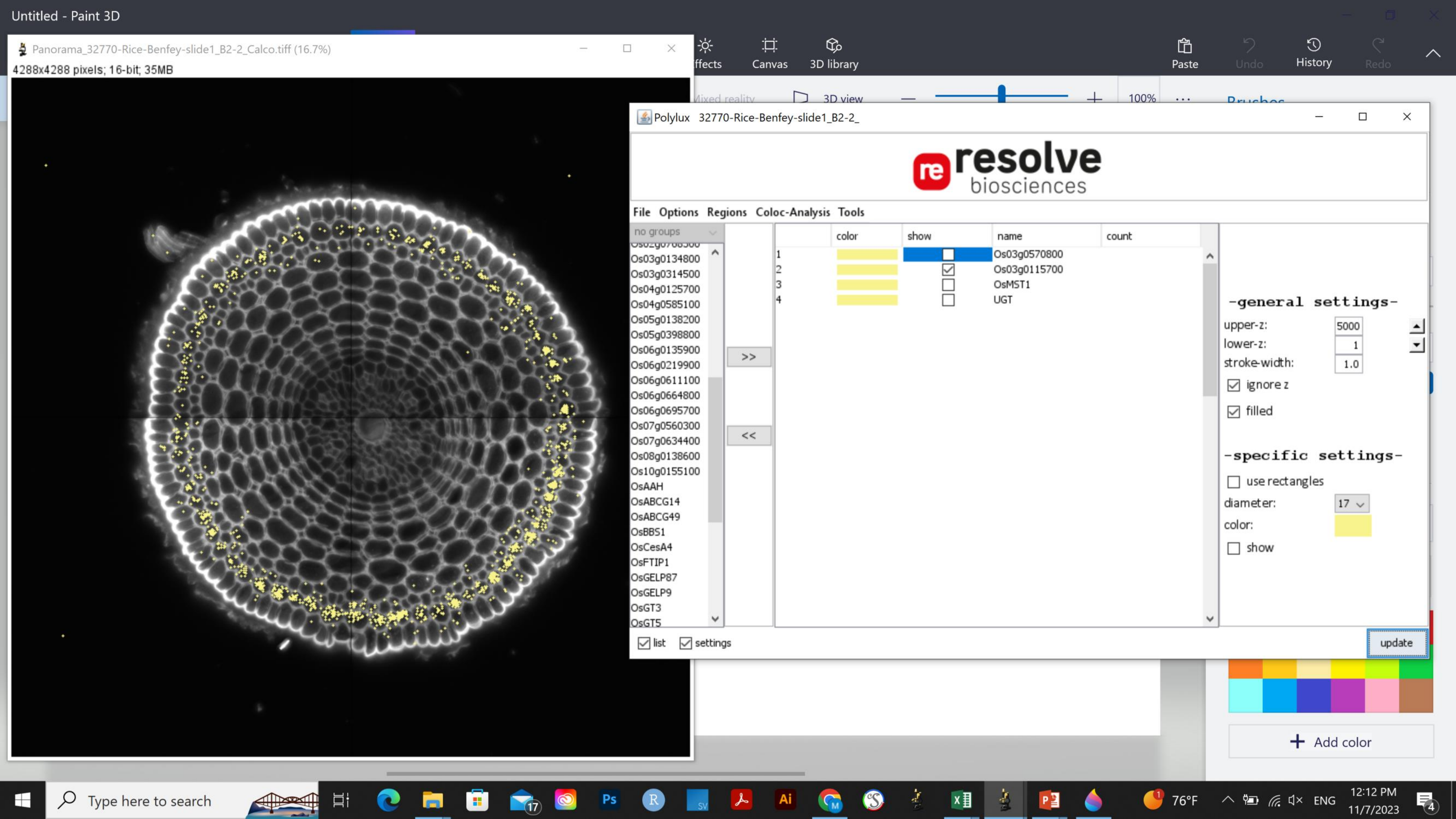

Untitled - Paint 3D
Panorama_32770-Rice-Benfey-slide1_B2-2_Calco.tiff (16.7%)
4288x4288 pixels; 16-bit; 35MB
Canvas
3D library
Paste
Undo
History
Redo
Polylux 32770-Rice-Benfey-slide1_B2-2_
resolve
biosciences
File Options Regions Coloc-Analysis Tools
no groups
Os03g0134800
Os03g0314500
Os04g0125700
Os04g0585100
Os05g0138200
Os05g0398800
Os06g0135900
Os06g0219900
Os06g0611100
Os06g0664800
Os06g0695700
Os07g0560300
Os07g0634400
Os08g0138600
Os10g0155100
OsAAH
OsABCG14
OsABCG49
OsBBS1
OsCesA4
OsFTIP1
OsGELP87
OsGELP9
OsGT3
OsGT5
>>
<<
color
show
name
count
1 Os03g0570800
2 Os03g0115700
3 OsMST1
4 UGT
-general settings-
upper-z: 5000
lower-z: 1
stroke-width: 1.0
ignore z
filled
-specific settings-
use rectangles
diameter: 17
color:
show
list
settings
update
Add color
Type here to search
76°F
ENG
12:12 PM
11/7/2023

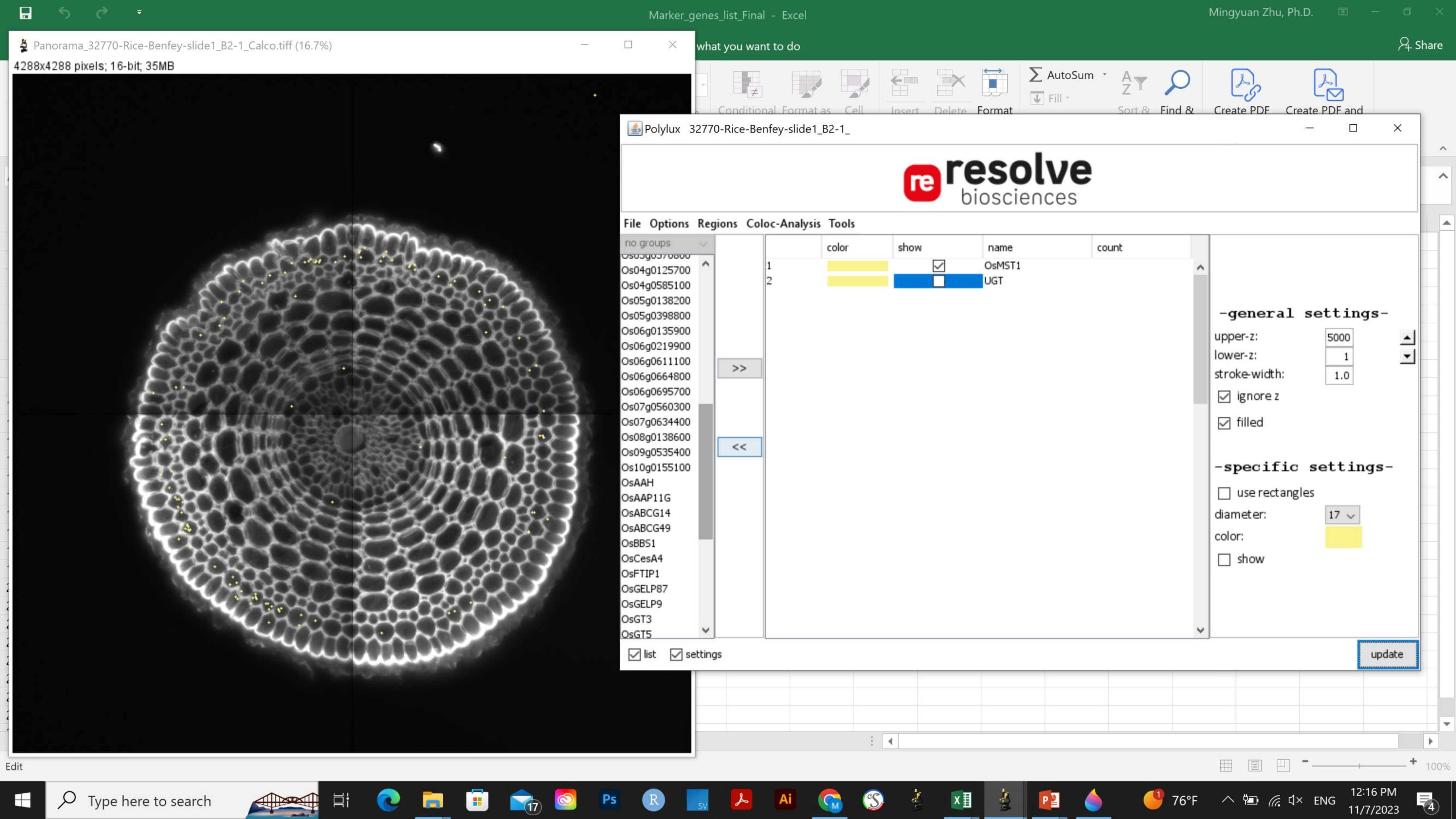

Marker_genes_list_Final - Excel
Mingyuan Zhu, Ph.D.
Panorama_32770-Rice-Benfey-slide1_B2-1_Calco.tiff (16.7%)
4288x4288 pixels; 16-bit; 35MB
Polylux 32770-Rice-Benfey-slide1_B2-1_
resolve biosciences
File Options Regions Coloc-Analysis Tools
no groups
Os04g0125700
Os04g0585100
Os05g0138200
Os05g0398800
Os06g0135900
Os06g0219900
Os06g0611100
Os06g0664800
Os06g0695700
Os07g0560300
Os07g0634400
Os08g0138600
Os09g0535400
Os10g0155100
OsAAH
OsAAP11G
OsABCG14
OsABCG49
OsBBS1
OsCesA4
OsFTIP1
OsGELP87
OsGELP9
OsGT3
OsGT5
color show name count
1 OsMST1
2 UGT
-general settings-
upper-z: 5000
lower-z: 1
stroke-width: 1.0
ignore z
filled
-specific settings-
use rectangles
diameter: 17
color:
show
list
settings
update
Type here to search
76°F
12:16 PM
11/7/2023

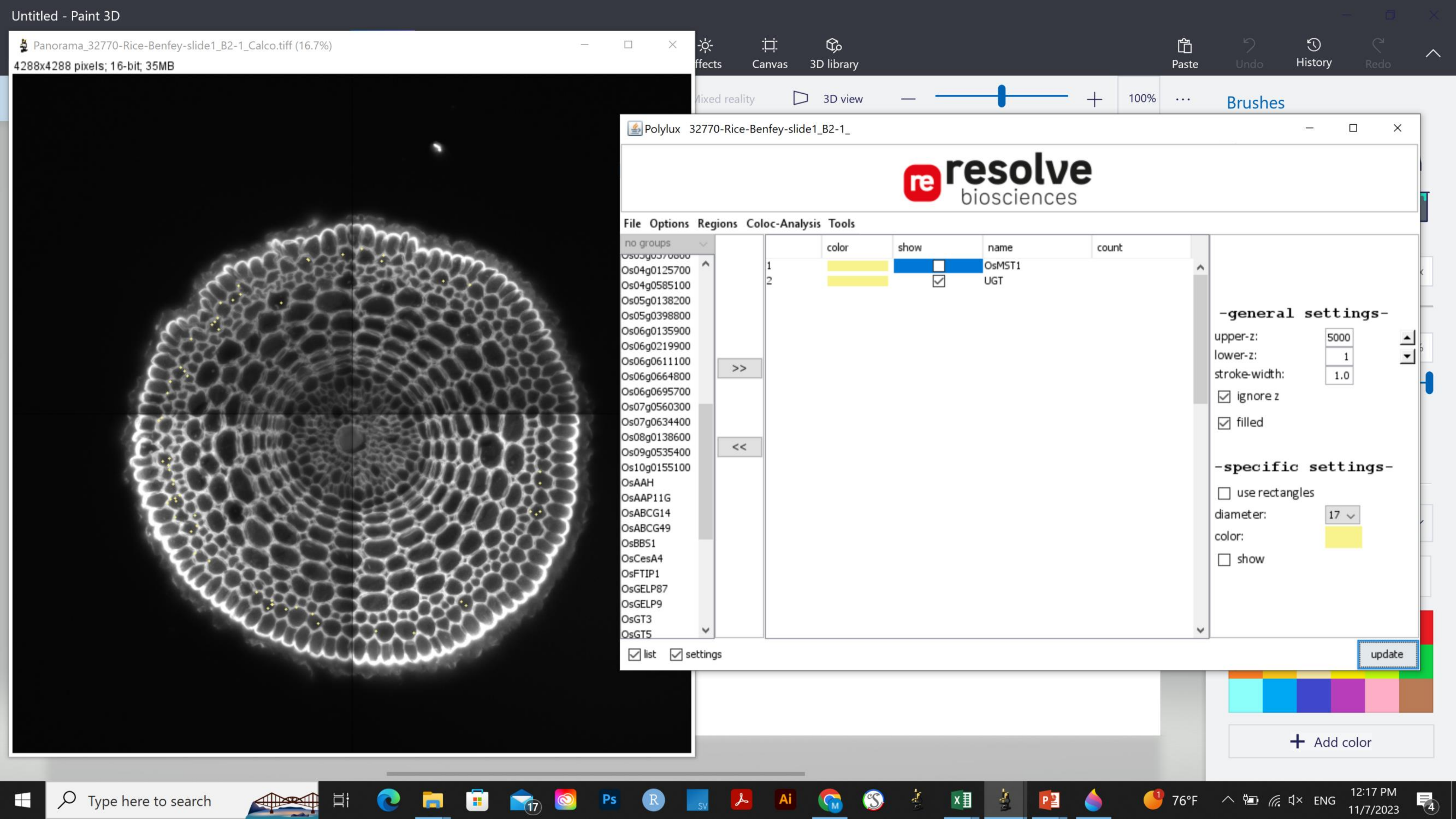

Untitled - Paint 3D
Panorama_32770-Rice-Benfey-slide1_B2-1_Calco.tiff (16.7%)
4288x4288 pixels; 16-bit; 35MB
Effects
Canvas
3D library
Paste
Undo
History
Redo
Mixed reality
3D view
100%
Brushes
Polylux 32770-Rice-Benfey-slide1_B2-1_
resolve biosciences
File Options Regions Coloc-Analysis Tools
no groups
Os04g0125700
Os04g0585100
Os05g0138200
Os05g0398800
Os06g0135900
Os06g0219900
Os06g0611100
Os06g0664800
Os06g0695700
Os07g0560300
Os07g0634400
Os08g0138600
Os09g0535400
Os10g0155100
OsAAH
OsAAP11G
OsABCG14
OsABCG49
OsBBS1
OsCesA4
OsFTIP1
OsGELP87
OsGELP9
OsGT3
OsGT5
>>
<<
color
show
name
count
1 OsMST1
2 UGT
-general settings-
upper-z: 5000
lower-z: 1
stroke-width: 1.0
ignore z
filled
-specific settings-
use rectangles
diameter: 17
color:
show
list
settings
update
Add color
Type here to search
76°F
ENG
12:17 PM
11/7/2023

Sclerenchyma

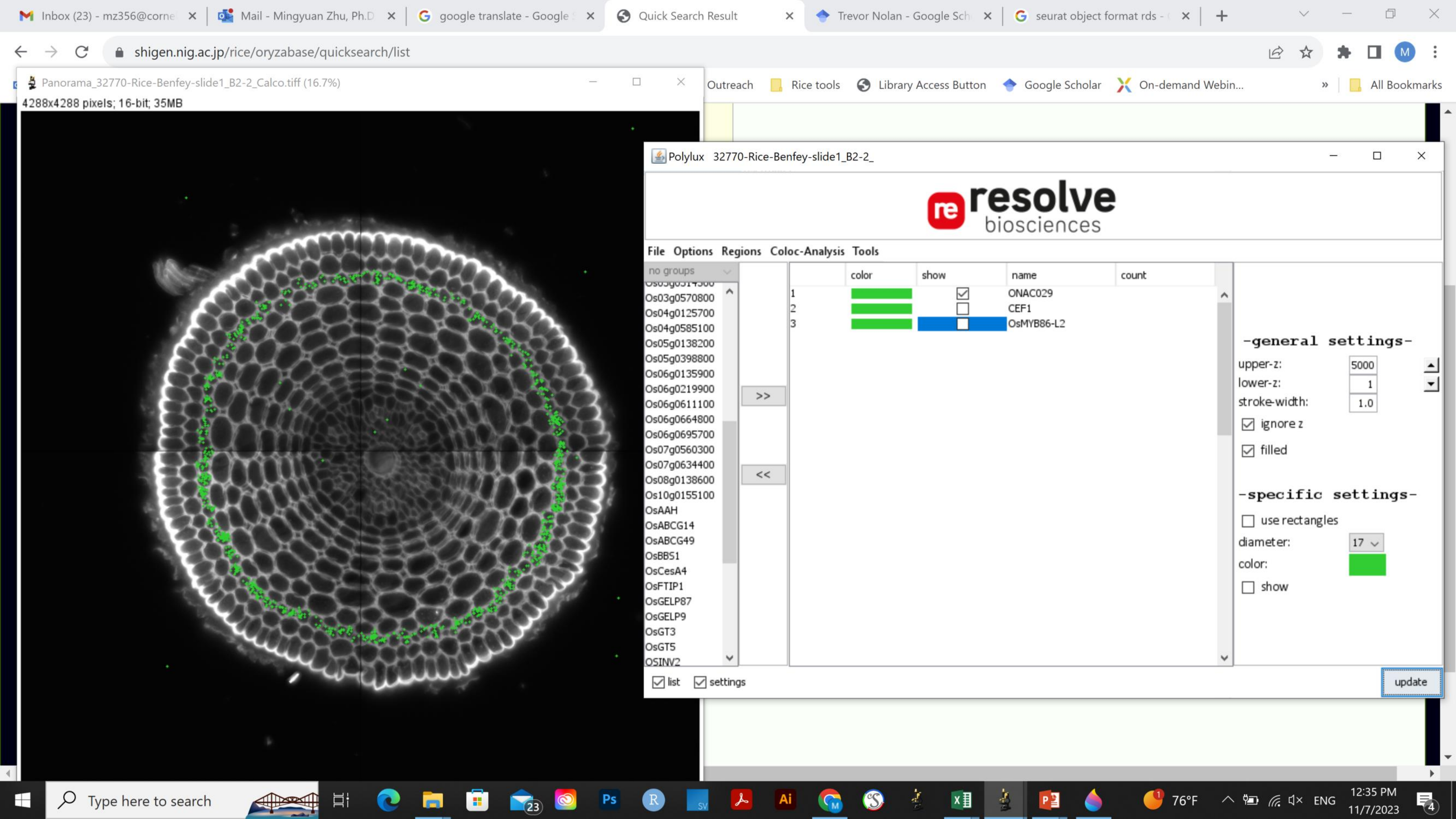
Inbox (23) - mz356@corne
Mail - Mingyuan Zhu, Ph.D
google translate - Google
Quick Search Result
Trevor Nolan - Google Sch
seurat object format rds -
shigen.nig.ac.jp/rice/oryzabase/quicksearch/list
Outreach
Rice tools
Library Access Button
Google Scholar
On-demand Webin...
All Bookmarks
Panorama_32770-Rice-Benfey-slide1_B2-2_Calco.tiff (16.7%)
4288x4288 pixels; 16-bit; 35MB
Polylux 32770-Rice-Benfey-slide1_B2-2_
resolve
biosciences
File Options Regions Coloc-Analysis Tools
no groups
Os03g0570800
Os04g0125700
Os04g0585100
Os05g0138200
Os05g0398800
Os06g0135900
Os06g0219900
Os06g0611100
Os06g0664800
Os06g0695700
Os07g0560300
Os07g0634400
Os08g0138600
Os10g0155100
OsAAH
OsABCG14
OsABCG49
OsBBS1
OsCesA4
OsFTIP1
OsGELP87
OsGELP9
OsGT3
OsGT5
OSINV2
>>
<<
color
show
name
count
1
ONAC029
2
CEF1
3
OsMYB86-L2
-general settings-
upper-z:
5000
lower-z:
1
stroke-width:
1.0
ignore z
filled
-specific settings-
use rectangles
diameter:
17
color:
show
list
settings
update
Type here to search
76°F
ENG
12:35 PM
11/7/2023

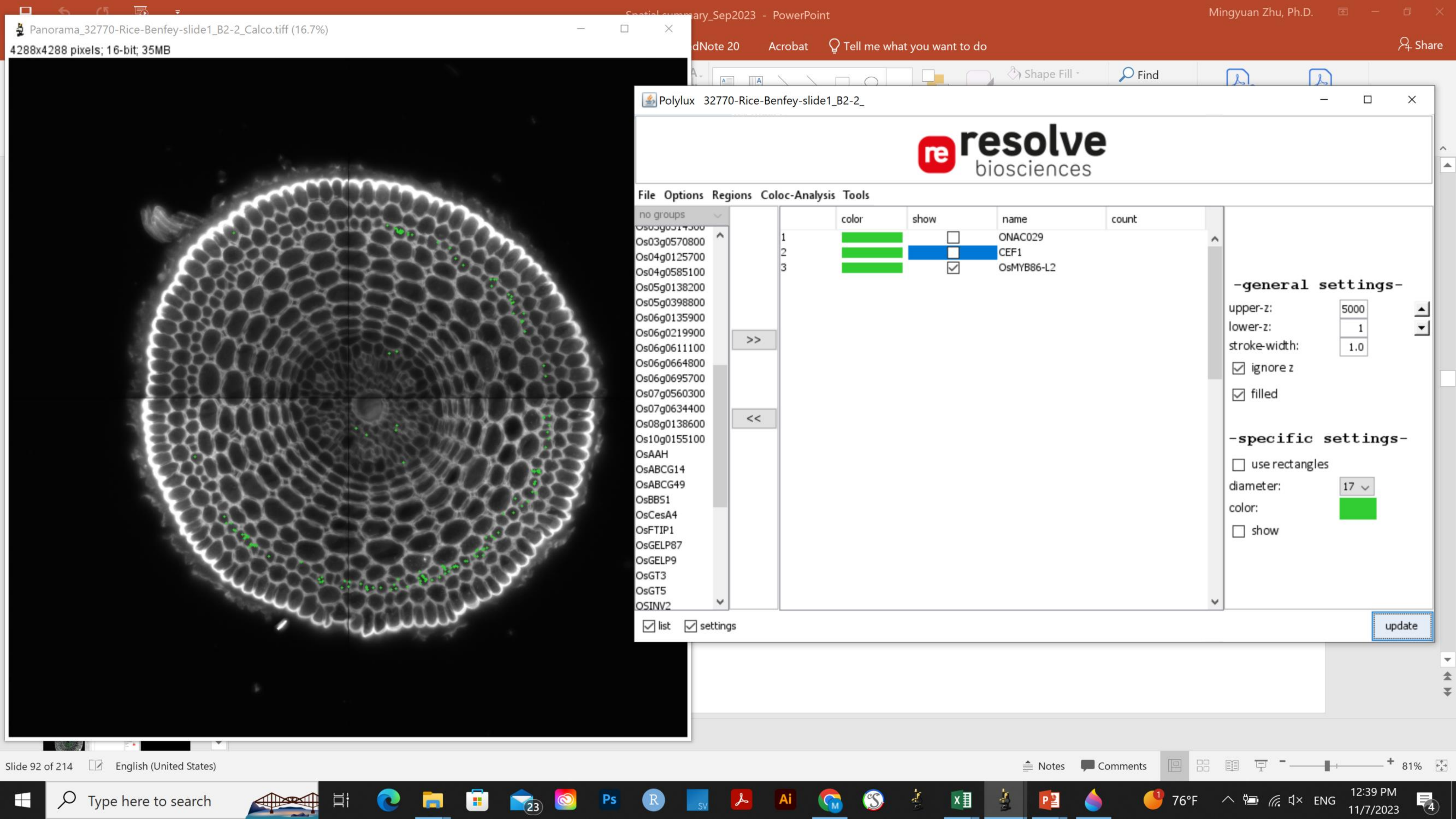

Spatial_summary_Sep2023 - PowerPoint
Mingyuan Zhu, Ph.D.
Panorama_32770-Rice-Benfey-slide1_B2-2_Calco.tiff (16.7%)
4288x4288 pixels; 16-bit; 35MB
Polylux 32770-Rice-Benfey-slide1_B2-2_
resolve biosciences
File Options Regions Coloc-Analysis Tools
no groups
Os03g0570800
Os04g0125700
Os04g0585100
Os05g0138200
Os05g0398800
Os06g0135900
Os06g0219900
Os06g0611100
Os06g0664800
Os06g0695700
Os07g0560300
Os07g0634400
Os08g0138600
Os10g0155100
OsAAH
OsABCG14
OsABCG49
OsBBS1
OsCesA4
OsFTIP1
OsGELP87
OsGELP9
OsGT3
OsGT5
OSINV2
>>
<<
color show name count
1 ONAC029
2 CEF1
3 OsMYB86-L2
-general settings-
upper-z: 5000
lower-z: 1
stroke-width: 1.0
ignore z
filled
-specific settings-
use rectangles
diameter: 17
color:
show
list settings
update
Slide 92 of 214
English (United States)
Notes Comments 81%
Type here to search
76°F ENG 12:39 PM 11/7/2023

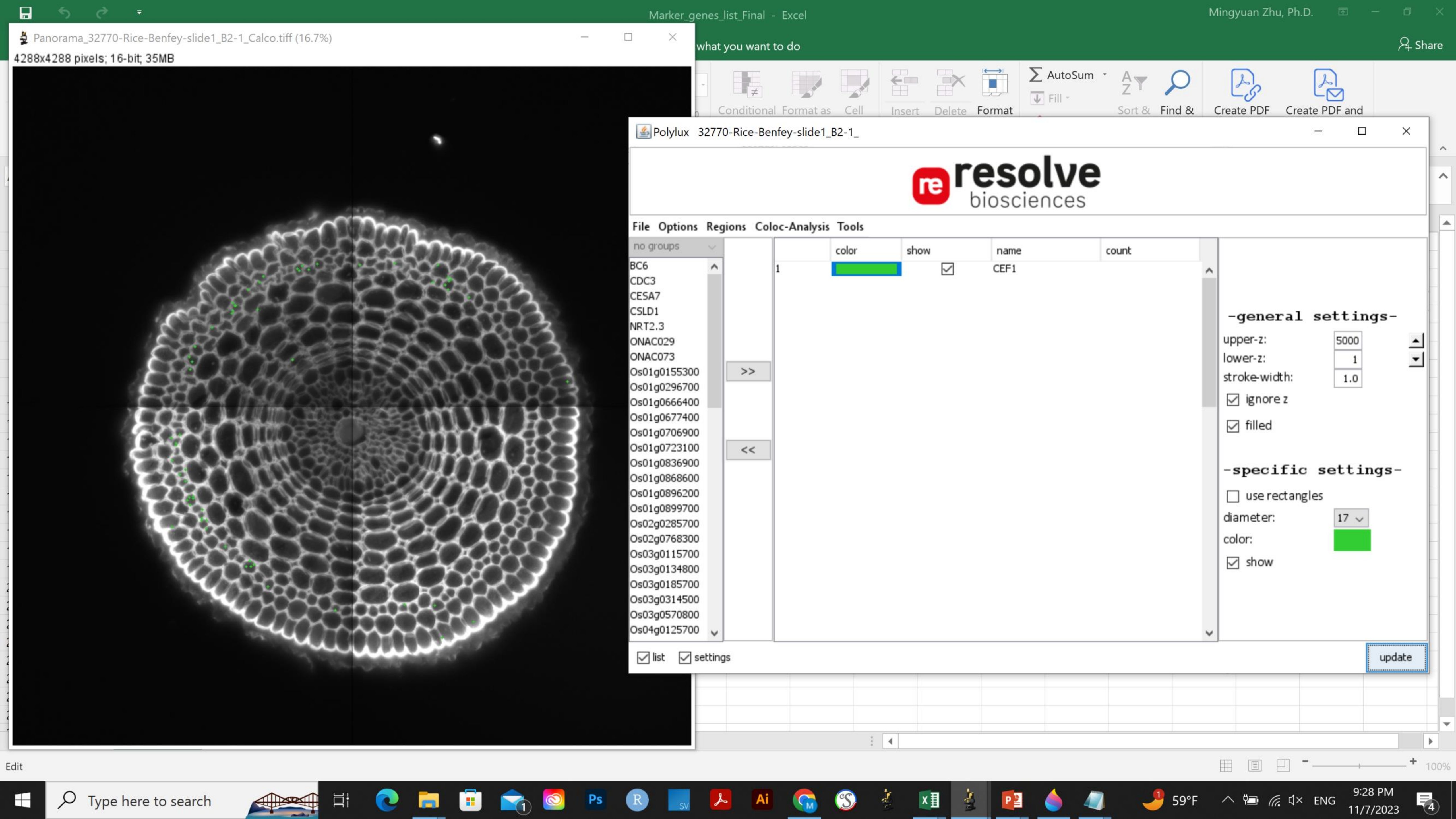
Marker_genes_list_Final - Excel
Mingyuan Zhu, Ph.D.
Panorama_32770-Rice-Benfey-slide1_B2-1_Calco.tiff (16.7%)
4288x4288 pixels; 16-bit; 35MB
Polylux 32770-Rice-Benfey-slide1_B2-1_
resolve biosciences
File Options Regions Coloc-Analysis Tools
no groups
BC6
CDC3
CESA7
CSLD1
NRT2.3
ONAC029
ONAC073
Os01g0155300
Os01g0296700
Os01g0666400
Os01g0677400
Os01g0706900
Os01g0723100
Os01g0836900
Os01g0868600
Os01g0896200
Os01g0899700
Os02g0285700
Os02g0768300
Os03g0115700
Os03g0134800
Os03g0185700
Os03g0314500
Os03g0570800
Os04g0125700
>>
<<
color
show
name
count
1
CEF1
-general settings-
upper-z: 5000
lower-z: 1
stroke-width: 1.0
ignore z
filled
-specific settings-
use rectangles
diameter: 17
color:
show
list
settings
update
Edit
Type here to search
59°F
ENG
9:28 PM
11/7/2023

Cortex

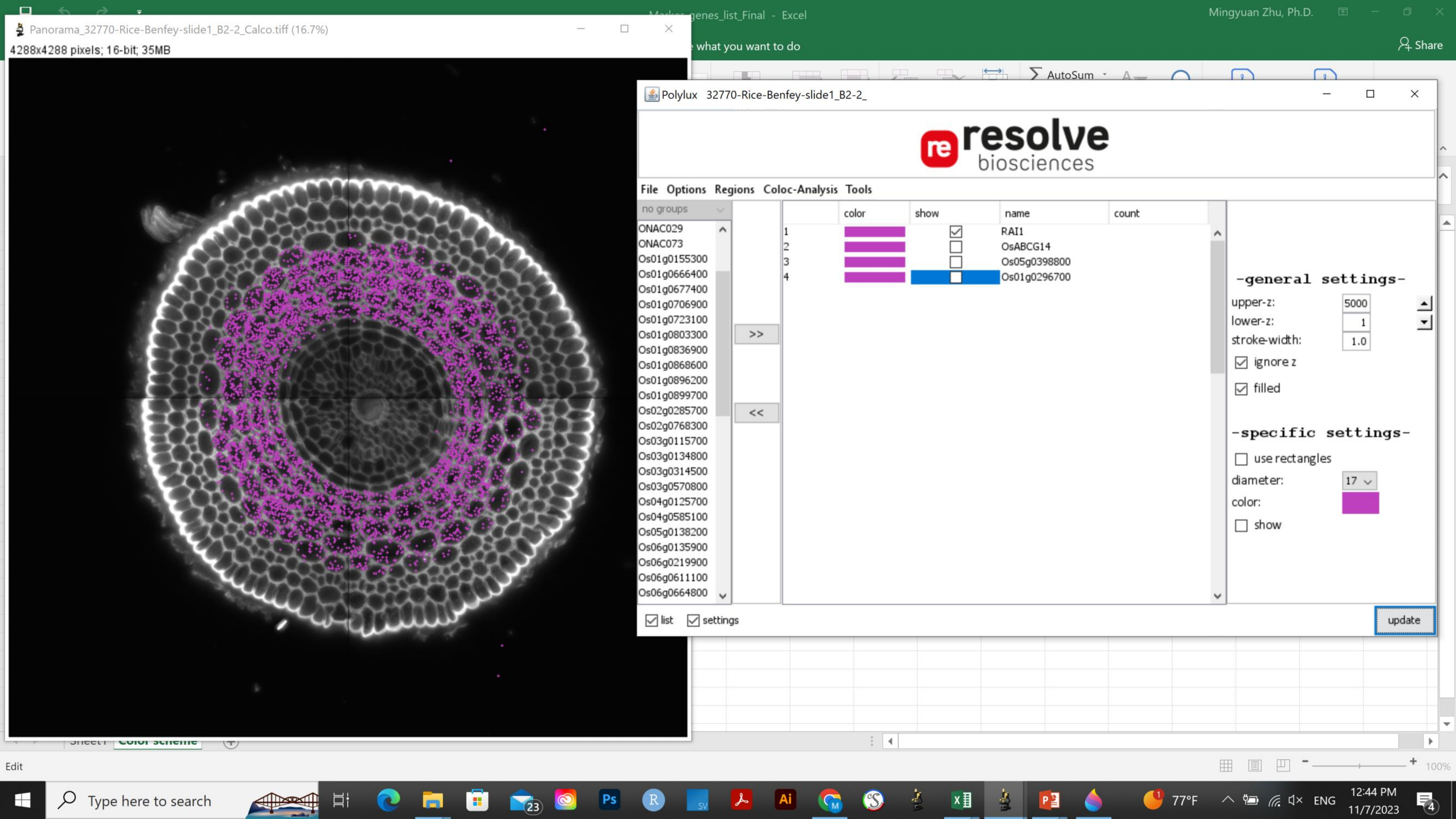
Panorama_32770-Rice-Benfey-slide1_B2-2_Calco.tiff (16.7%)
4288x4288 pixels; 16-bit; 35MB
genes_list_Final - Excel
Mingyuan Zhu, Ph.D.
what you want to do
Share
AutoSum
Polylux 32770-Rice-Benfey-slide1_B2-2_
resolve biosciences
File Options Regions Coloc-Analysis Tools
no groups
ONAC029
ONAC073
Os01g0155300
Os01g0666400
Os01g0677400
Os01g0706900
Os01g0723100
Os01g0803300
Os01g0836900
Os01g0868600
Os01g0896200
Os01g0899700
Os02g0285700
Os02g0768300
Os03g0115700
Os03g0134800
Os03g0314500
Os03g0570800
Os04g0125700
Os04g0585100
Os05g0138200
Os06g0135900
Os06g0219900
Os06g0611100
Os06g0664800
>>
<<
color
show
name
count
1 RAI1
2 OsABCG14
3 Os05g0398800
4 Os01g0296700
-general settings-
upper-z: 5000
lower-z: 1
stroke-width: 1.0
ignore z
filled
-specific settings-
use rectangles
diameter: 17
color:
show
list
settings
update
Edit
100%
Type here to search
77°F
ENG
12:44 PM
11/7/2023

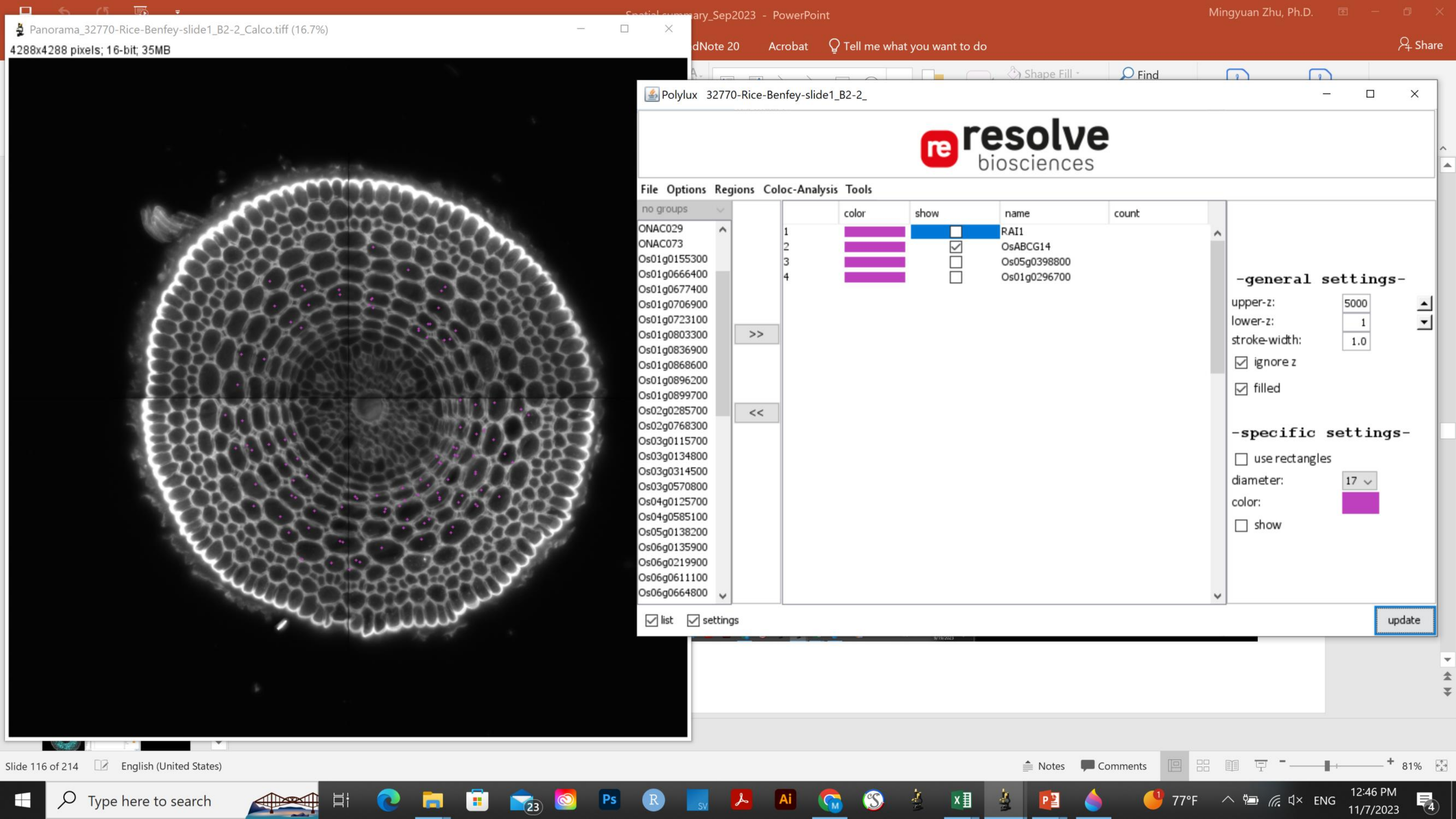
Spatial_summary_Sep2023 - PowerPoint
Mingyuan Zhu, Ph.D.
dNote 20
Acrobat
Tell me what you want to do
Share
Shape Fill
Find
Panorama_32770-Rice-Benfey-slide1_B2-2_Calco.tiff (16.7%)
4288x4288 pixels; 16-bit; 35MB
Polylux 32770-Rice-Benfey-slide1_B2-2_
resolve
biosciences
File
Options
Regions
Coloc-Analysis
Tools
no groups
ONAC029
ONAC073
Os01g0155300
Os01g0666400
Os01g0677400
Os01g0706900
Os01g0723100
Os01g0803300
Os01g0836900
Os01g0868600
Os01g0896200
Os01g0899700
Os02g0285700
Os02g0768300
Os03g0115700
Os03g0134800
Os03g0314500
Os03g0570800
Os04g0125700
Os04g0585100
Os05g0138200
Os06g0135900
Os06g0219900
Os06g0611100
Os06g0664800
>>
<<
color
show
name
count
1 RAI1
2 OsABCG14
3 Os05g0398800
4 Os01g0296700
-general settings-
upper-z: 5000
lower-z: 1
stroke-width: 1.0
ignore z
filled
-specific settings-
use rectangles
diameter: 17
color:
show
list
settings
update
Slide 116 of 214
English (United States)
Notes
Comments
81%
Type here to search
77°F
ENG
12:46 PM
11/7/2023

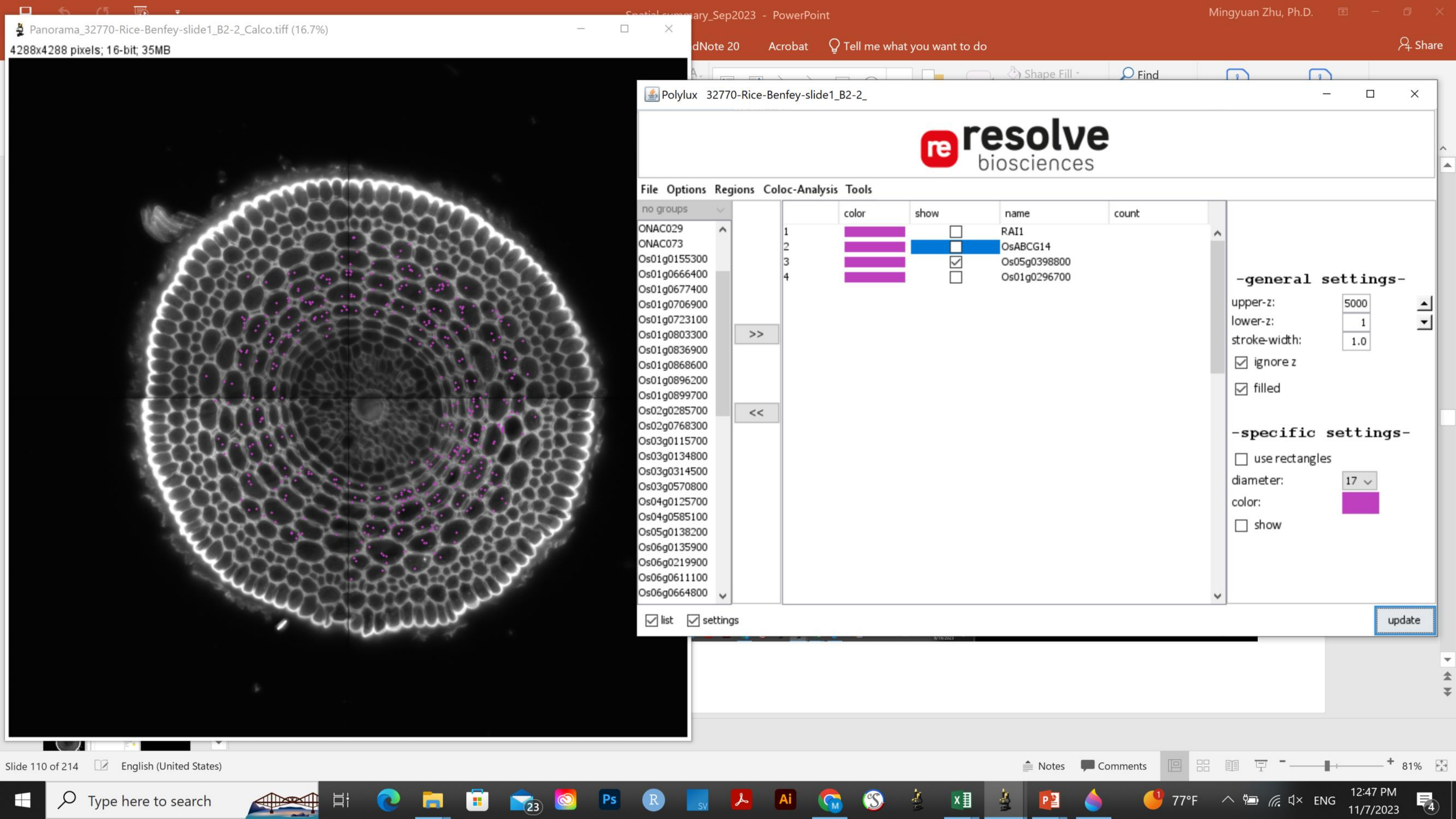
Spatial_summary_Sep2023 - PowerPoint
Mingyuan Zhu, Ph.D.
Panorama_32770-Rice-Benfey-slide1_B2-2_Calco.tiff (16.7%)
4288x4288 pixels; 16-bit; 35MB
Polylux 32770-Rice-Benfey-slide1_B2-2_
resolve biosciences
File Options Regions Coloc-Analysis Tools
no groups
ONAC029
ONAC073
Os01g0155300
Os01g0666400
Os01g0677400
Os01g0706900
Os01g0723100
Os01g0803300
Os01g0836900
Os01g0868600
Os01g0896200
Os01g0899700
Os02g0285700
Os02g0768300
Os03g0115700
Os03g0134800
Os03g0314500
Os03g0570800
Os04g0125700
Os04g0585100
Os05g0138200
Os06g0135900
Os06g0219900
Os06g0611100
Os06g0664800
>>
<<
color show name count
1 RAI1
2 OsABCG14
3 Os05g0398800
4 Os01g0296700
-general settings-
upper-z: 5000
lower-z: 1
stroke-width: 1.0
ignore z
filled
-specific settings-
use rectangles
diameter: 17
color:
show
list settings
update
Slide 110 of 214
English (United States)
Notes Comments 81%
Type here to search
77°F ENG 12:47 PM 11/7/2023

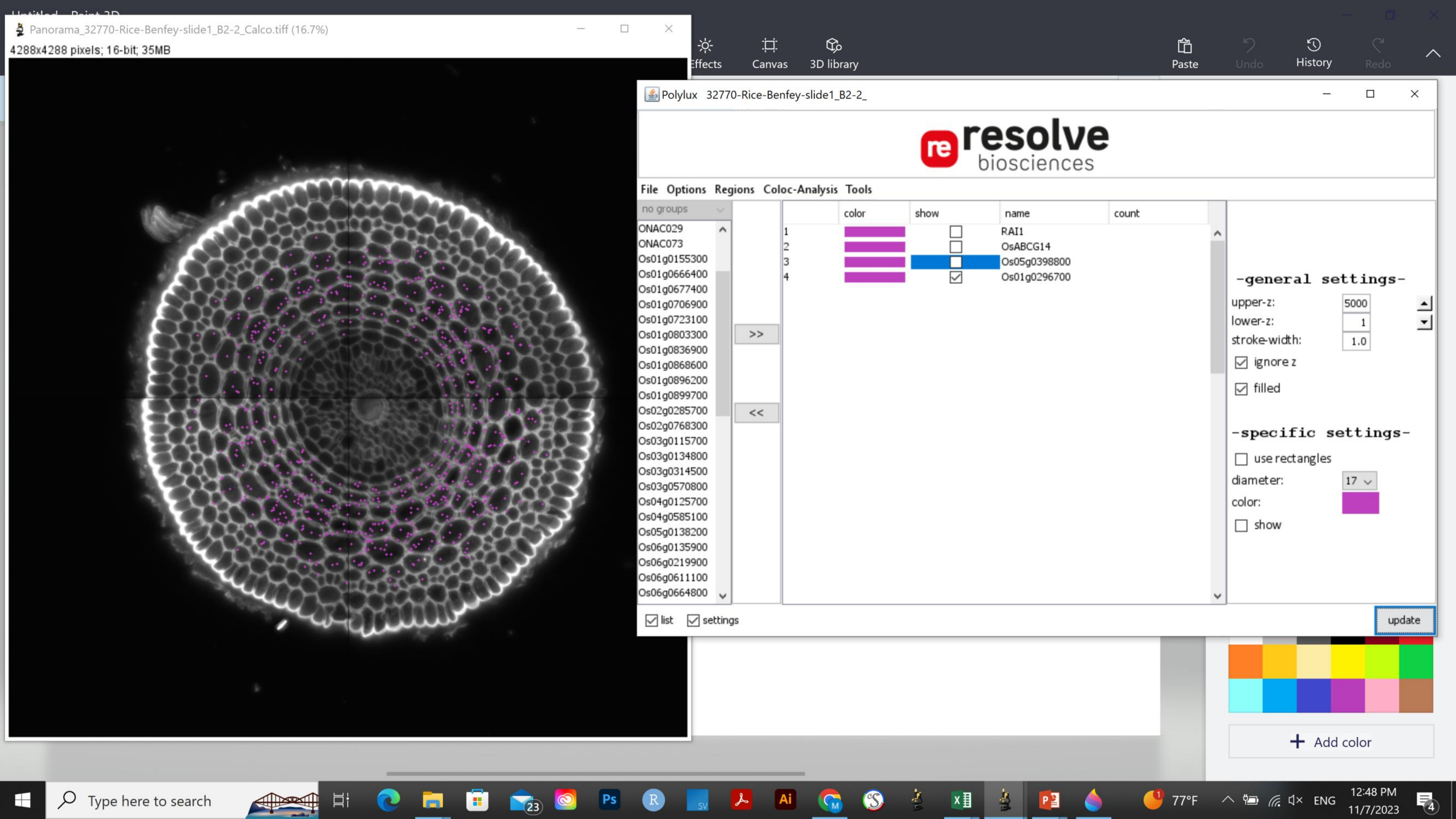

Panorama_32770-Rice-Benfey-slide1_B2-2_Calco.tiff (16.7%)
4288x4288 pixels; 16-bit; 35MB
Effects
Canvas
3D library
Paste
Undo
History
Redo
Polylux 32770-Rice-Benfey-slide1_B2-2_
resolve
biosciences
File Options Regions Coloc-Analysis Tools
no groups
ONAC029
ONAC073
Os01g0155300
Os01g0666400
Os01g0677400
Os01g0706900
Os01g0723100
Os01g0803300
Os01g0836900
Os01g0868600
Os01g0896200
Os01g0899700
Os02g0285700
Os02g0768300
Os03g0115700
Os03g0134800
Os03g0314500
Os03g0570800
Os04g0125700
Os04g0585100
Os05g0138200
Os06g0135900
Os06g0219900
Os06g0611100
Os06g0664800
>>
<<
color
show
name
count
1 RAI1
2 OsABCG14
3 Os05g0398800
4 Os01g0296700
-general settings-
upper-z: 5000
lower-z: 1
stroke-width: 1.0
ignore z
filled
-specific settings-
use rectangles
diameter: 17
color:
show
list
settings
update
Add color
Type here to search
77°F
ENG
12:48 PM
11/7/2023

Endodermis

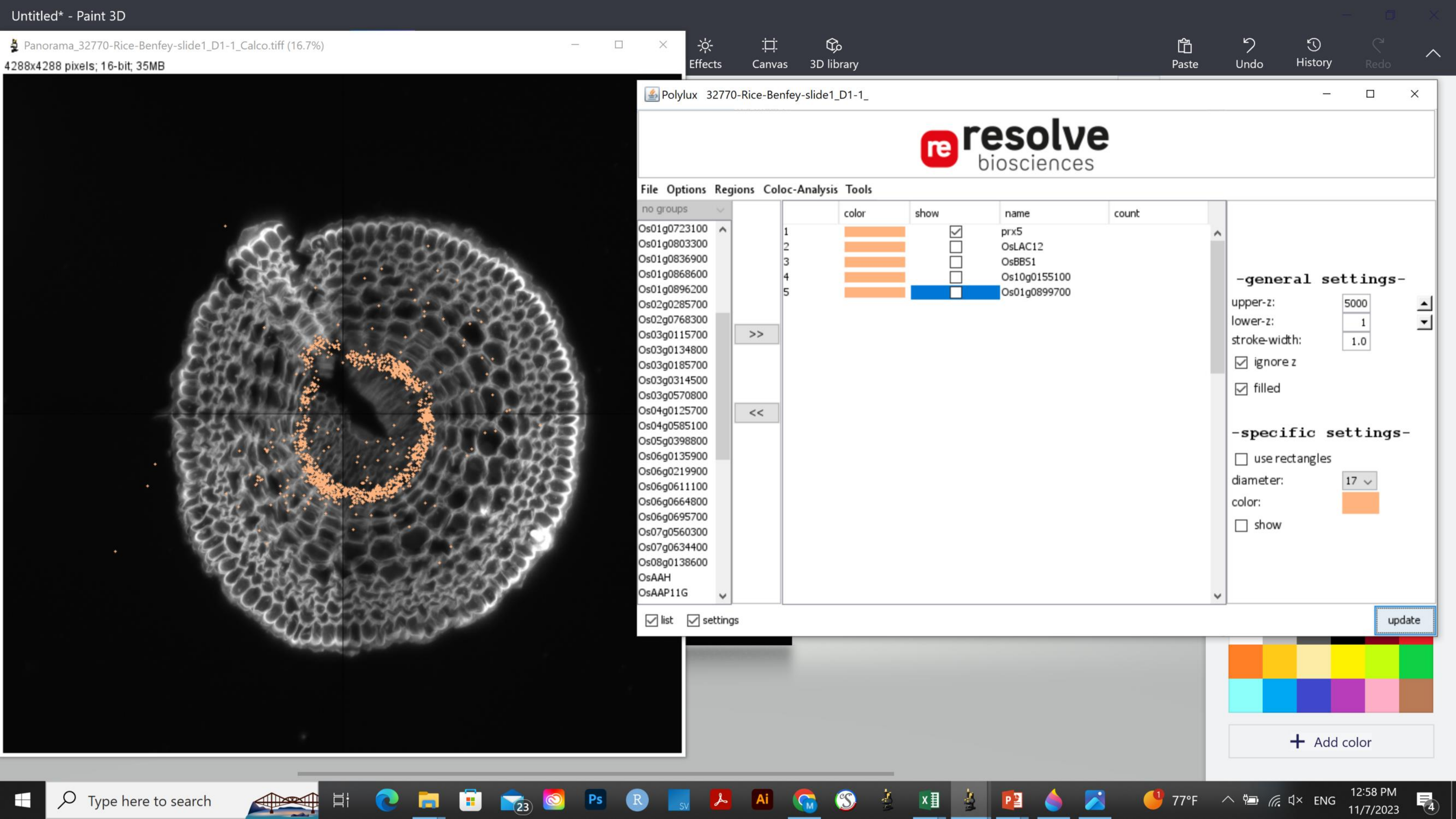
Untitled* - Paint 3D
Panorama_32770-Rice-Benfey-slide1_D1-1_Calco.tiff (16.7%)
4288x4288 pixels; 16-bit; 35MB
Effects
Canvas
3D library
Paste
Undo
History
Redo
Polylux 32770-Rice-Benfey-slide1_D1-1_
resolve biosciences
File Options Regions Coloc-Analysis Tools
no groups
Os01g0723100
Os01g0803300
Os01g0836900
Os01g0868600
Os01g0896200
Os02g0285700
Os02g0768300
Os03g0115700
Os03g0134800
Os03g0185700
Os03g0314500
Os03g0570800
Os04g0125700
Os04g0585100
Os05g0398800
Os06g0135900
Os06g0219900
Os06g0611100
Os06g0664800
Os06g0695700
Os07g0560300
Os07g0634400
Os08g0138600
OsAAH
OsAAP11G
>>
<<
color show name count
1 prx5
2 OsLAC12
3 OsBBS1
4 Os10g0155100
5 Os01g0899700
-general settings-
upper-z: 5000
lower-z: 1
stroke-width: 1.0
ignore z
filled
-specific settings-
use rectangles
diameter: 17
color:
show
list
settings
update
Add color
Type here to search
77°F
ENG
12:58 PM
11/7/2023

Inbox (25) - mz356@corne | Mail - Mingyuan Zhu, Ph.D | google translate - Google | Quick Search Result | Trevor Nolan - Google Sch | seurat object format rds -

Panorama_32770-Rice-Benfey-slide1_B2-1_Calco.tiff (16.7%)

4288x4288 pixels; 16-bit; 35MB

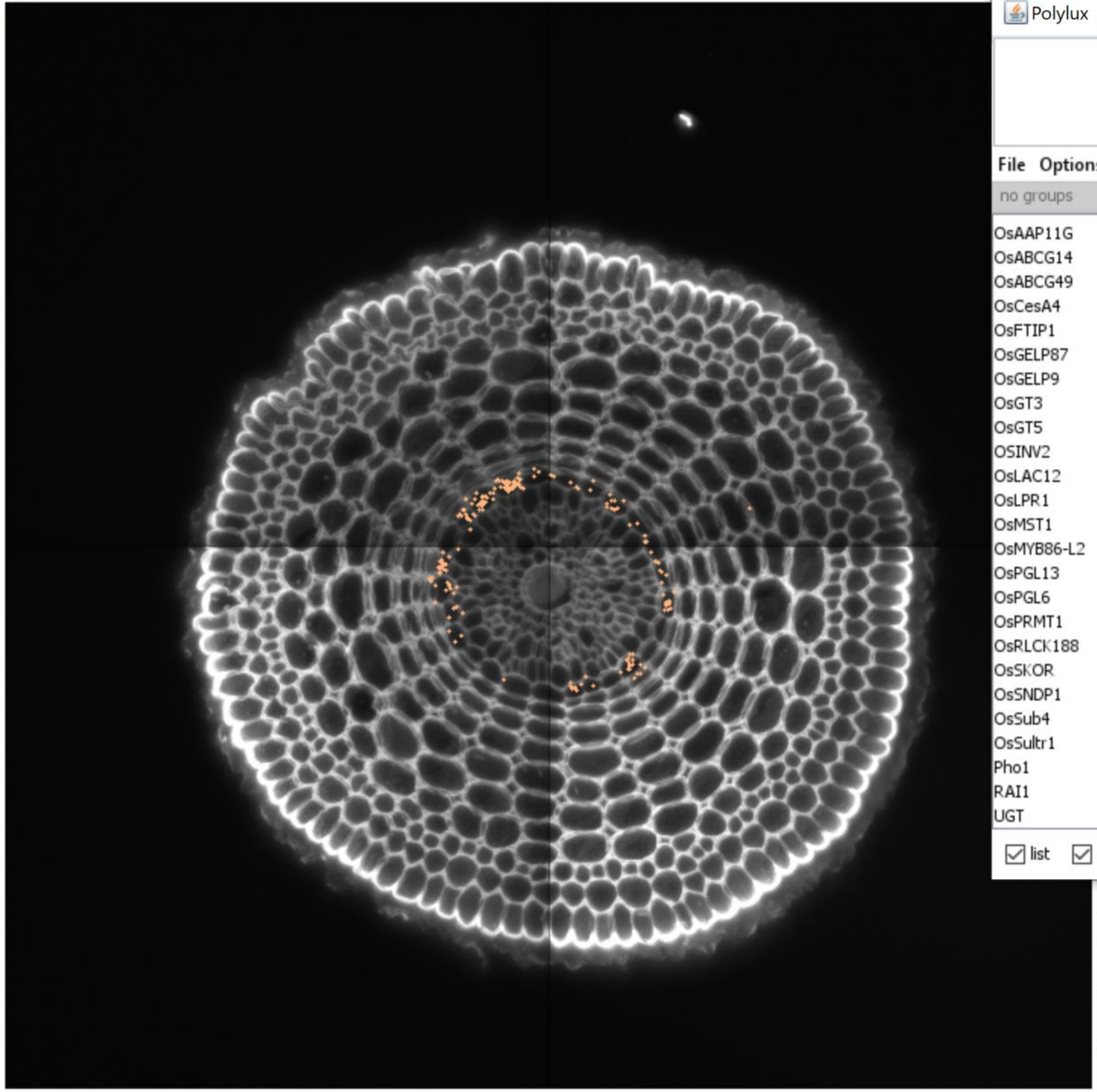

Polylux 32770-Rice-Benfey-slide1_B2-1_

re resolve biosciences

File Options Regions Coloc-Analysis Tools

no groups

OsAAP11G
OsABCG14
OsABCG49
OsCesA4
OsFTIP1
OsGELP87
OsGELP9
OsGT3
OsGT5
OSINV2
OsLAC12
OsLPR1
OsMST1
OsMYB86-L2
OsPGL13
OsPGL6
OsPRMT1
OsRLCK188
OsSKOR
OsSNDP1
OsSub4
OsSultr1
Pho1
RAI1
UGT

>>

<<

	color	show	name	count
1		☑	prx5	
2		☐	OsBBS1	

-general settings-

upper-z: 5000

lower-z: 1

stroke-width: 1.0

☑ ignore z

☑ filled

-specific settings-

☐ use rectangles

diameter: 17

color:

☐ show

☑ list ☑ settings

update

Os02g40500 and rought stress.	Ecotoxicol. Environ. Saf.	200	110721	2020	32464438	
tome sequencing reveal that two _10794 (LOC_Os03g14950) t.	Physiol Mol Biol Plants	23(4)	745-754	2017	29158625	_LEA7

Type here to search

79°F ENG 1:22 PM 11/7/2023

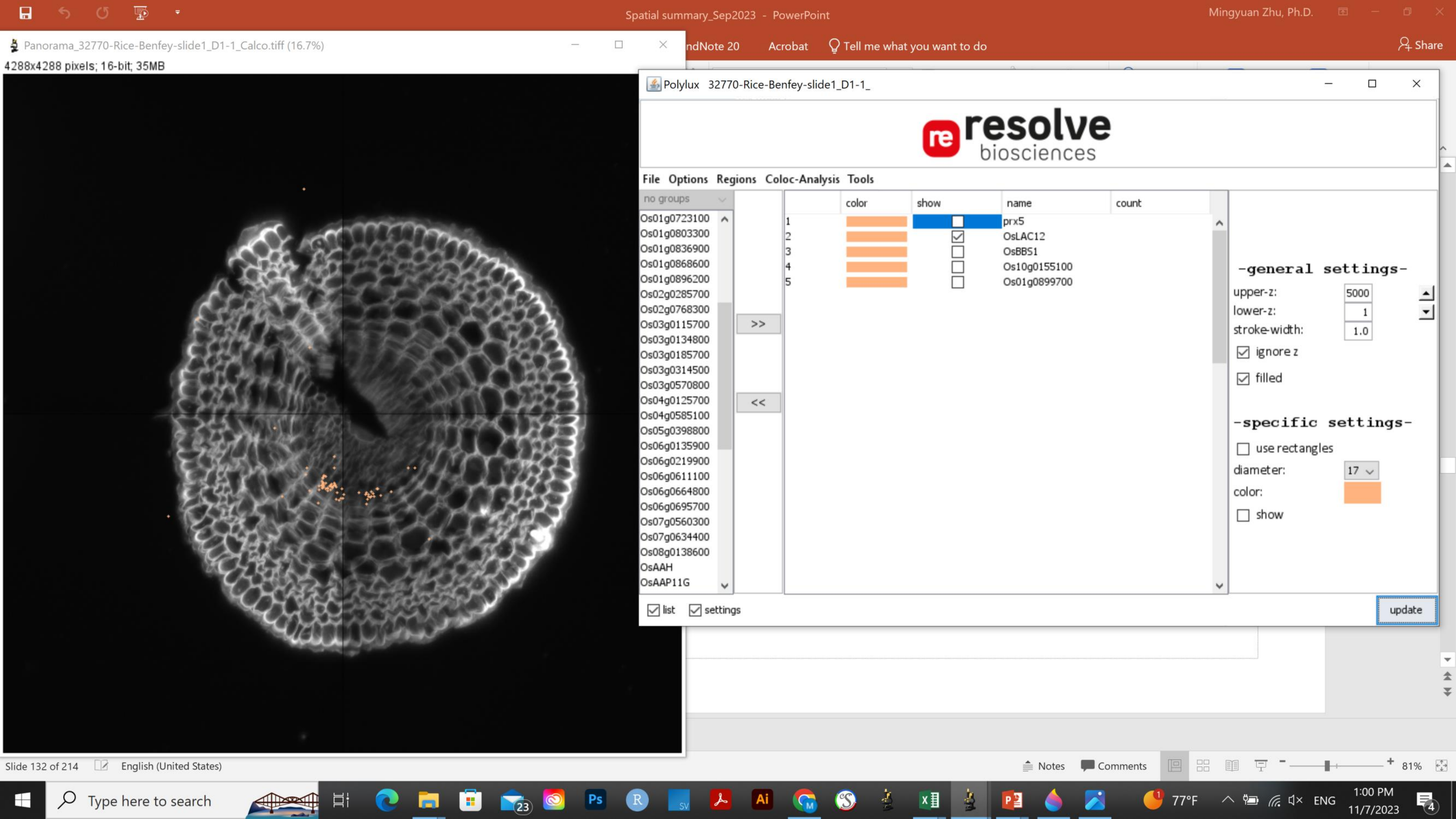
Spatial summary_Sep2023 - PowerPoint
Mingyuan Zhu, Ph.D.
Panorama_32770-Rice-Benfey-slide1_D1-1_Calco.tiff (16.7%)
4288x4288 pixels; 16-bit; 35MB
Polylux 32770-Rice-Benfey-slide1_D1-1_
resolve biosciences
File Options Regions Coloc-Analysis Tools
no groups
Os01g0723100
Os01g0803300
Os01g0836900
Os01g0868600
Os01g0896200
Os02g0285700
Os02g0768300
Os03g0115700
Os03g0134800
Os03g0185700
Os03g0314500
Os03g0570800
Os04g0125700
Os04g0585100
Os05g0398800
Os06g0135900
Os06g0219900
Os06g0611100
Os06g0664800
Os06g0695700
Os07g0560300
Os07g0634400
Os08g0138600
OsAAH
OsAAP11G
color
show
name
count
1 prx5
2 OsLAC12
3 OsBBS1
4 Os10g0155100
5 Os01g0899700
-general settings-
upper-z: 5000
lower-z: 1
stroke-width: 1.0
ignore z
filled
-specific settings-
use rectangles
diameter: 17
color:
show
list
settings
update
Slide 132 of 214
English (United States)
Notes
Comments
81%
Type here to search
1:00 PM
11/7/2023

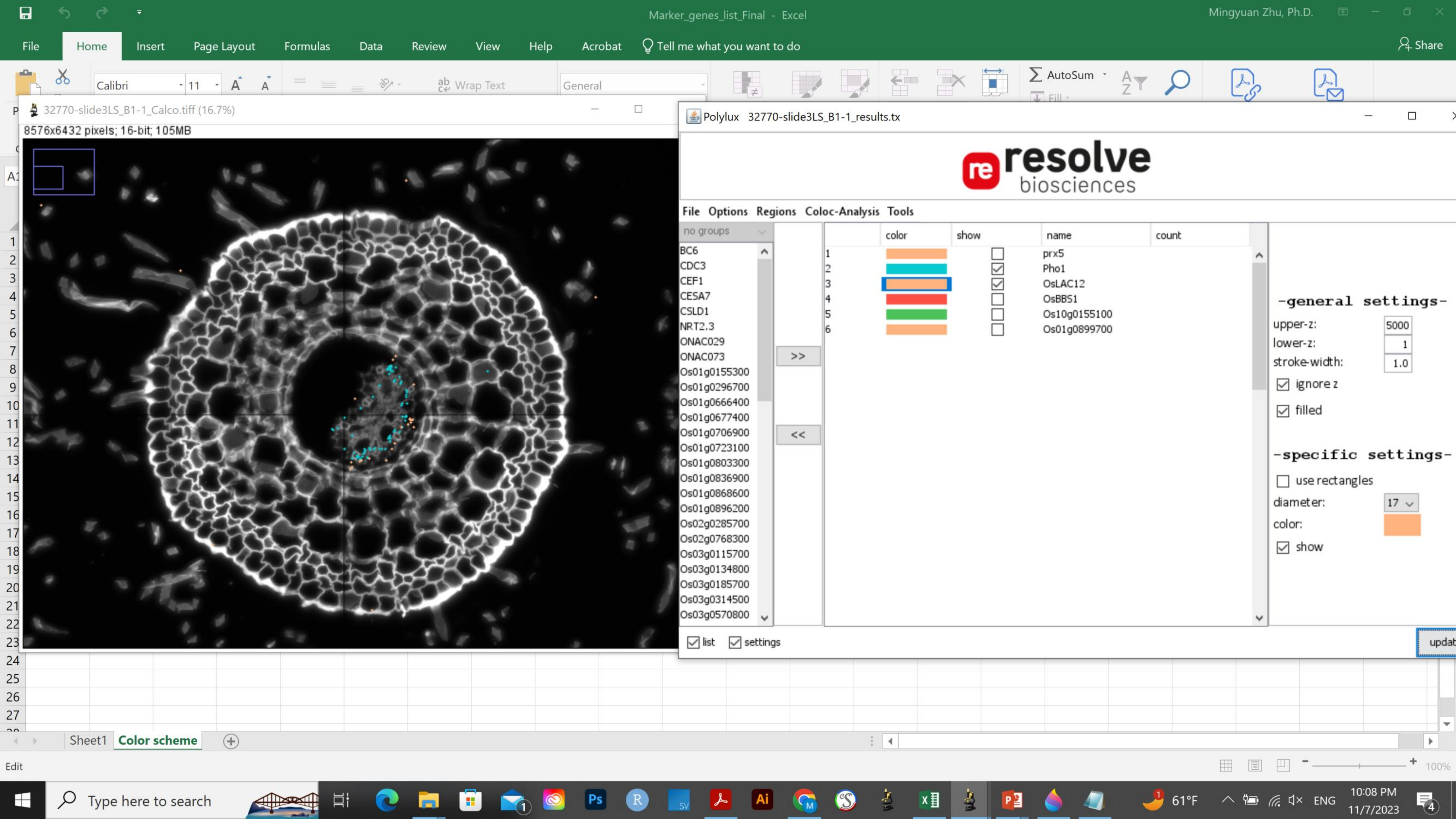
Marker_genes_list_Final - Excel
Mingyuan Zhu, Ph.D.
File Home Insert Page Layout Formulas Data Review View Help Acrobat Tell me what you want to do
Share
Calibri 11
Wrap Text
General
AutoSum
Fill
32770-slide3LS_B1-1_Calco.tiff (16.7%)
8576x6432 pixels; 16-bit; 105MB
Polylux 32770-slide3LS_B1-1_results.tx
resolve biosciences
File Options Regions Coloc-Analysis Tools
no groups
BC6
CDC3
CEF1
CESA7
CSLD1
NRT2.3
ONAC029
ONAC073
Os01g0155300
Os01g0296700
Os01g0666400
Os01g0677400
Os01g0706900
Os01g0723100
Os01g0803300
Os01g0836900
Os01g0868600
Os01g0896200
Os02g0285700
Os02g0768300
Os03g0115700
Os03g0134800
Os03g0185700
Os03g0314500
Os03g0570800
>>
<<
color show name count
1 prx5
2 Pho1
3 OsLAC12
4 OsBBS1
5 Os10g0155100
6 Os01g0899700
-general settings-
upper-z: 5000
lower-z: 1
stroke-width: 1.0
ignore z
filled
-specific settings-
use rectangles
diameter: 17
color:
show
list
settings
updat
Sheet1 Color scheme
Edit
100%
Type here to search
61°F
ENG
10:08 PM
11/7/2023

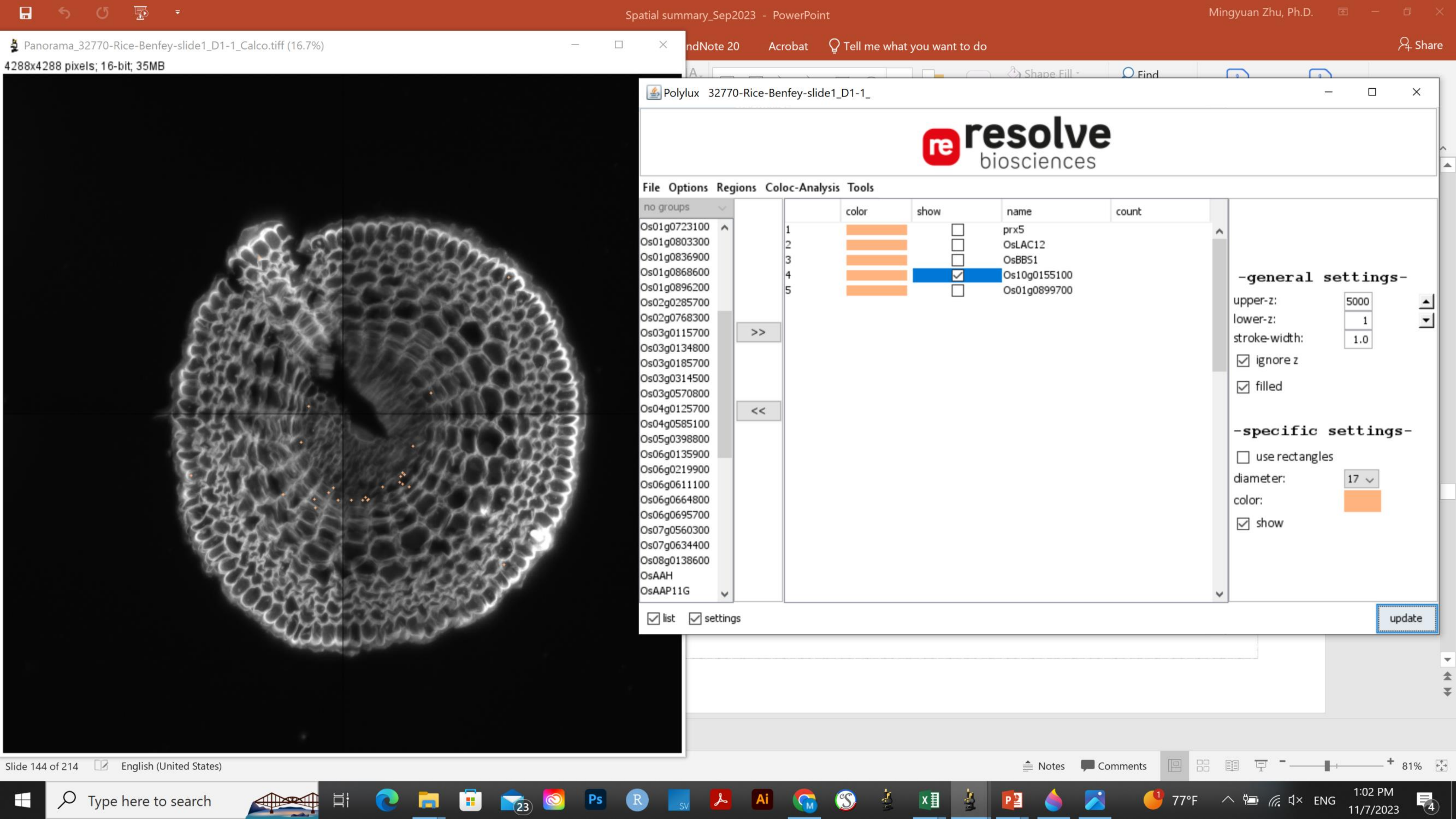
Spatial summary_Sep2023 - PowerPoint
Mingyuan Zhu, Ph.D.
Panorama_32770-Rice-Benfey-slide1_D1-1_Calco.tiff (16.7%)
4288x4288 pixels; 16-bit; 35MB
Polylux 32770-Rice-Benfey-slide1_D1-1_
resolve biosciences
File Options Regions Coloc-Analysis Tools
no groups
Os01g0723100
Os01g0803300
Os01g0836900
Os01g0868600
Os01g0896200
Os02g0285700
Os02g0768300
Os03g0115700
Os03g0134800
Os03g0185700
Os03g0314500
Os03g0570800
Os04g0125700
Os04g0585100
Os05g0398800
Os06g0135900
Os06g0219900
Os06g0611100
Os06g0664800
Os06g0695700
Os07g0560300
Os07g0634400
Os08g0138600
OsAAH
OsAAP11G
color show name count
1 prx5
2 OsLAC12
3 OsBBS1
4 Os10g0155100
5 Os01g0899700
-general settings-
upper-z: 5000
lower-z: 1
stroke-width: 1.0
ignore z
filled
-specific settings-
use rectangles
diameter: 17
color:
show
list settings
update
Slide 144 of 214 English (United States)
Notes Comments 81%
Type here to search
77°F ENG 1:02 PM 11/7/2023

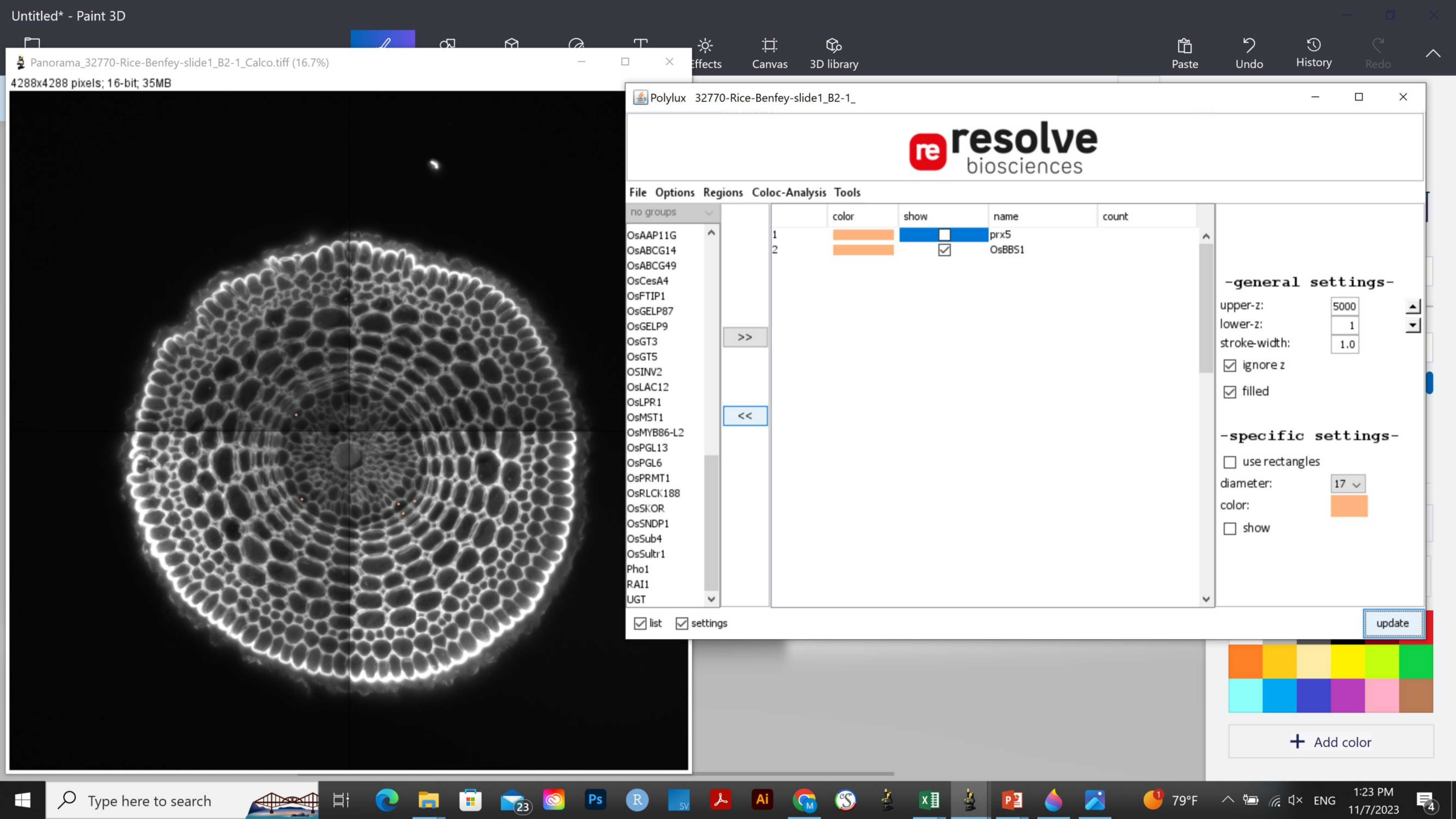
Untitled* - Paint 3D
Panorama_32770-Rice-Benfey-slide1_B2-1_Calco.tiff (16.7%)
4288x4288 pixels; 16-bit; 35MB
Effects
Canvas
3D library
Paste
Undo
History
Redo
Polylux 32770-Rice-Benfey-slide1_B2-1_
resolve biosciences
File Options Regions Coloc-Analysis Tools
no groups
OsAAP11G
OsABCG14
OsABCG49
OsCesA4
OsFTIP1
OsGELP87
OsGELP9
OsGT3
OsGT5
OSINV2
OsLAC12
OsLPR1
OsMST1
OsMYB86-L2
OsPGL13
OsPGL6
OsPRMT1
OsRLCK188
OsSKOR
OsSNDP1
OsSub4
OsSultr1
Pho1
RAI1
UGT
>>
<<
color
show
name
count
1
prx5
2
OsBBS1
-general settings-
upper-z:
5000
lower-z:
1
stroke-width:
1.0
ignore z
filled
-specific settings-
use rectangles
diameter:
17
color:
show
list
settings
update
Add color
Type here to search
79°F
ENG
1:23 PM
11/7/2023

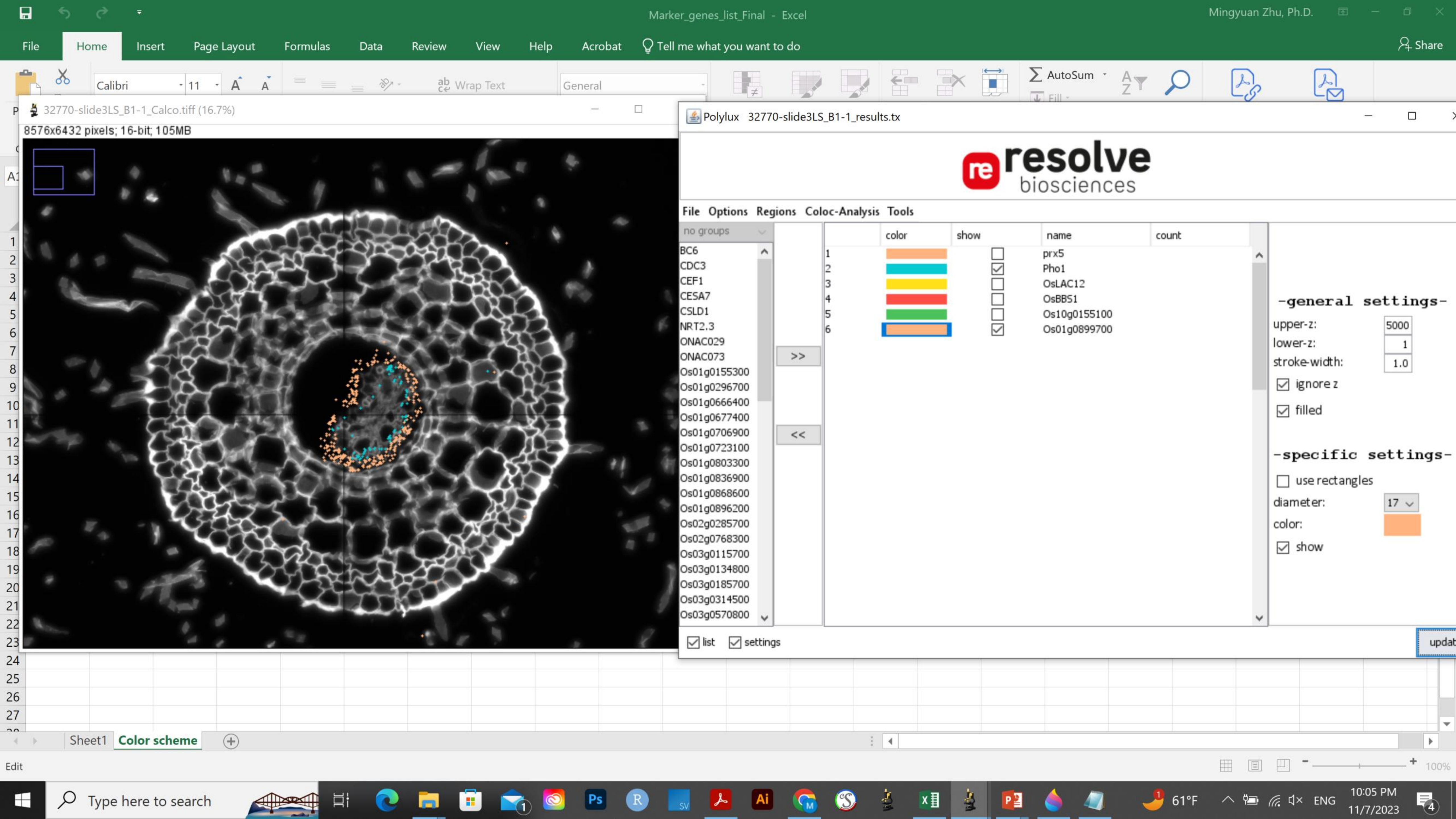
Marker_genes_list_Final - Excel
Mingyuan Zhu, Ph.D.
File Home Insert Page Layout Formulas Data Review View Help Acrobat Tell me what you want to do
Share
Calibri 11
Wrap Text
General
AutoSum
Fill
32770-slide3LS_B1-1_Calco.tiff (16.7%)
8576x6432 pixels; 16-bit; 105MB
Polylux 32770-slide3LS_B1-1_results.tx
resolve biosciences
File Options Regions Coloc-Analysis Tools
no groups
BC6
CDC3
CEF1
CESA7
CSLD1
NRT2.3
ONAC029
ONAC073
Os01g0155300
Os01g0296700
Os01g0666400
Os01g0677400
Os01g0706900
Os01g0723100
Os01g0803300
Os01g0836900
Os01g0868600
Os01g0896200
Os02g0285700
Os02g0768300
Os03g0115700
Os03g0134800
Os03g0185700
Os03g0314500
Os03g0570800
>>
<<
color show name count
1 prx5
2 Pho1
3 OsLAC12
4 OsBBS1
5 Os10g0155100
6 Os01g0899700
-general settings-
upper-z: 5000
lower-z: 1
stroke-width: 1.0
ignore z
filled
-specific settings-
use rectangles
diameter: 17
color:
show
list
settings
updat
Sheet1
Color scheme
Edit
100%
Type here to search
61°F
ENG
10:05 PM
11/7/2023

Pericycle/Procambium

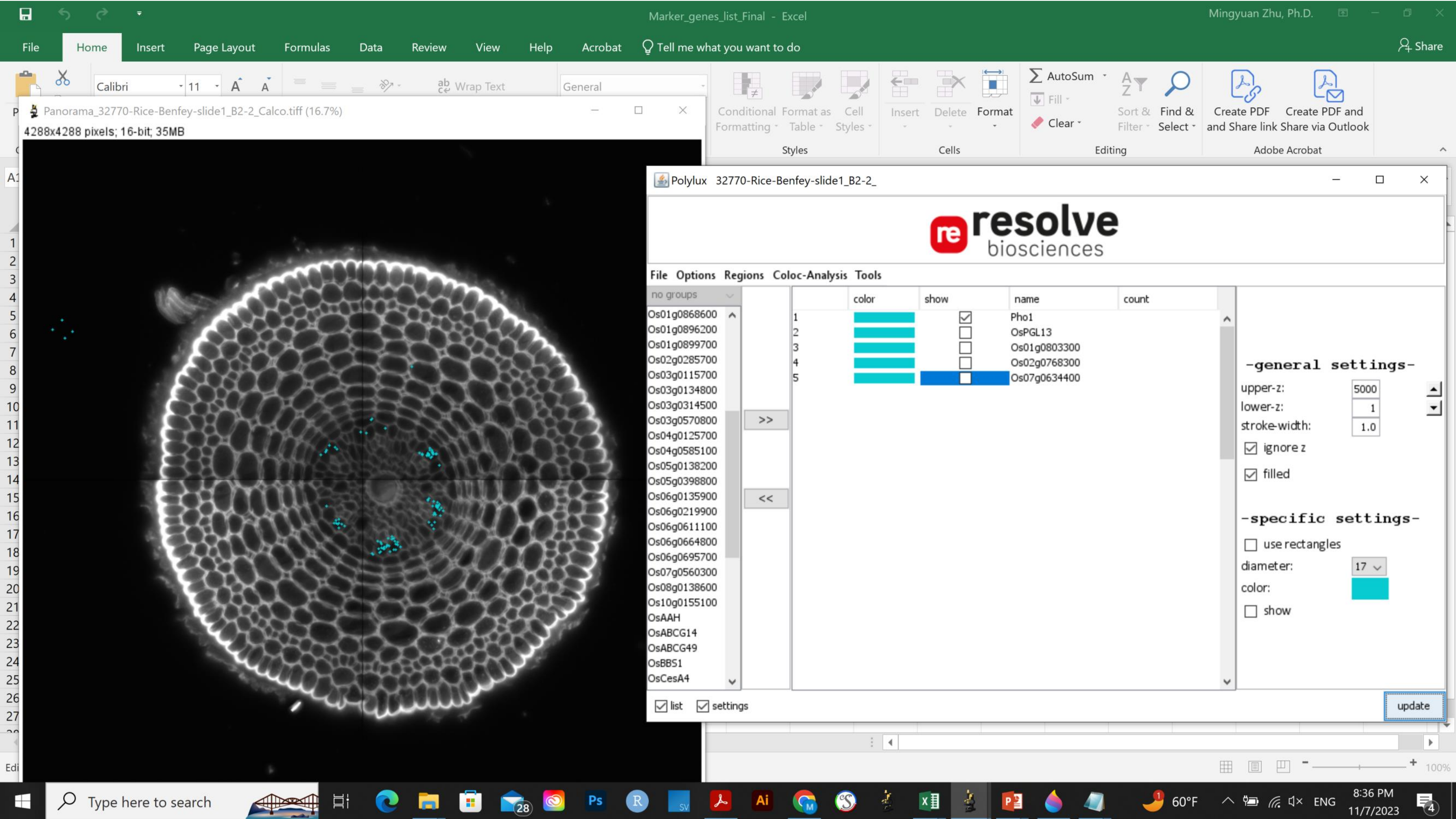
Marker_genes_list_Final - Excel
Mingyuan Zhu, Ph.D.
File
Home
Insert
Page Layout
Formulas
Data
Review
View
Help
Acrobat
Tell me what you want to do
Share
Calibri
11
Wrap Text
General
Conditional Formatting
Format as Table
Cell Styles
Insert
Delete
Format
AutoSum
Fill
Clear
Sort & Filter
Find & Select
Create PDF and Share link
Create PDF and Share via Outlook
Styles
Cells
Editing
Adobe Acrobat
Panorama_32770-Rice-Benfey-slide1_B2-2_Calco.tiff (16.7%)
4288x4288 pixels; 16-bit; 35MB
Polylux 32770-Rice-Benfey-slide1_B2-2_
resolve biosciences
File
Options
Regions
Coloc-Analysis
Tools
no groups
Os01g0868600
Os01g0896200
Os01g0899700
Os02g0285700
Os03g0115700
Os03g0134800
Os03g0314500
Os03g0570800
Os04g0125700
Os04g0585100
Os05g0138200
Os05g0398800
Os06g0135900
Os06g0219900
Os06g0611100
Os06g0664800
Os06g0695700
Os07g0560300
Os08g0138600
Os10g0155100
OsAAH
OsABCG14
OsABCG49
OsBBS1
OsCesA4
>>
<<
color
show
name
count
1 Pho1
2 OsPGL13
3 Os01g0803300
4 Os02g0768300
5 Os07g0634400
-general settings-
upper-z: 5000
lower-z: 1
stroke-width: 1.0
ignore z
filled
-specific settings-
use rectangles
diameter: 17
color:
show
list
settings
update
Type here to search
60°F
ENG
8:36 PM
11/7/2023

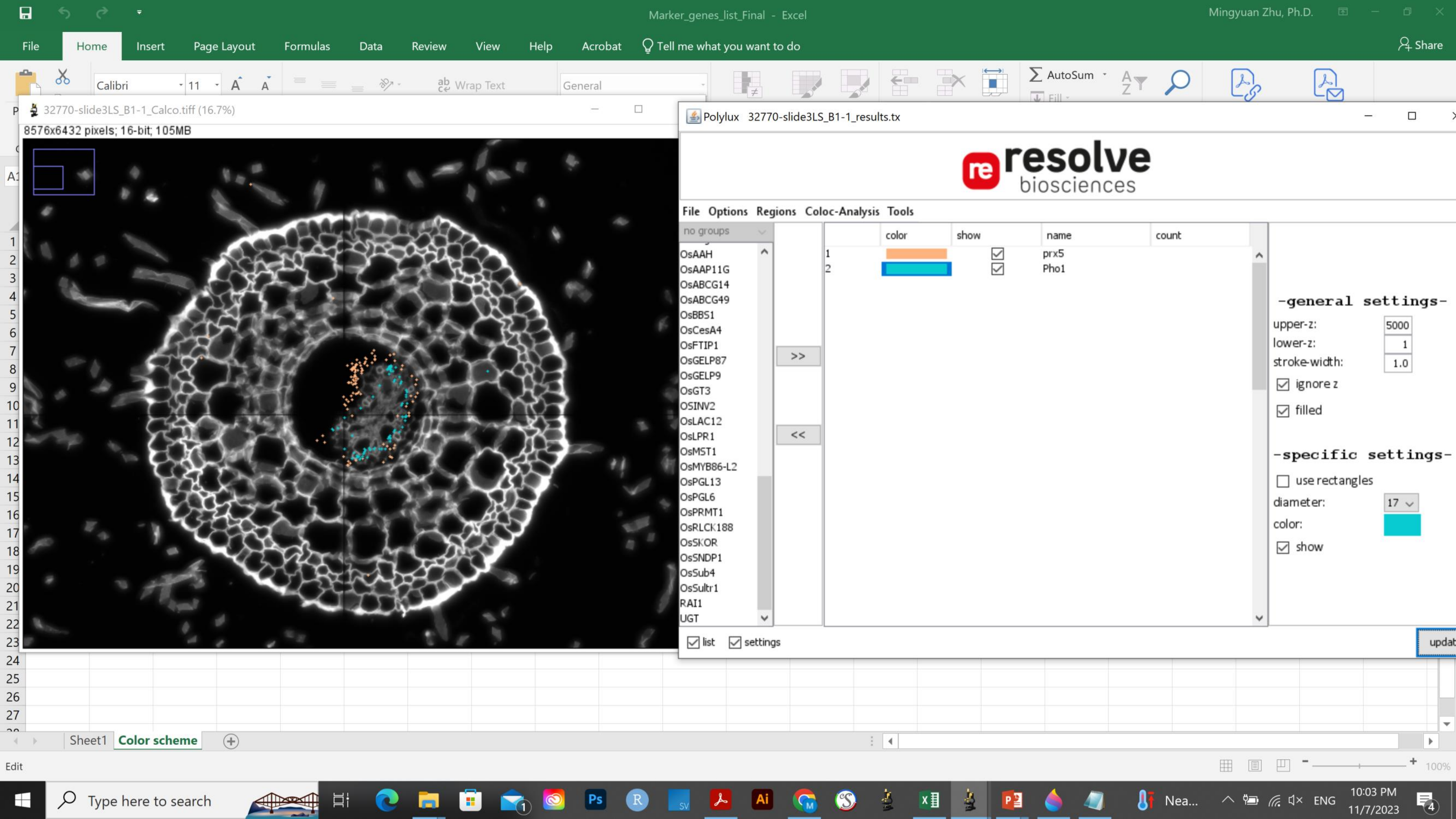
Marker_genes_list_Final - Excel
Mingyuan Zhu, Ph.D.
File
Home
Insert
Page Layout
Formulas
Data
Review
View
Help
Acrobat
Tell me what you want to do
Share
Calibri
11
Wrap Text
General
AutoSum
32770-slide3LS_B1-1_Calco.tiff (16.7%)
8576x6432 pixels; 16-bit; 105MB
Polylux 32770-slide3LS_B1-1_results.tx
resolve biosciences
File Options Regions Coloc-Analysis Tools
no groups
OsAAH
OsAAP11G
OsABCG14
OsABCG49
OsBBS1
OsCesA4
OsFTIP1
OsGELP87
OsGELP9
OsGT3
OSINV2
OsLAC12
OsLPR1
OsMST1
OsMYB86-L2
OsPGL13
OsPGL6
OsPRMT1
OsRLCK188
OsSKOR
OsSNDP1
OsSub4
OsSultr1
RAI1
UGT
>>
<<
color
show
name
count
1
prx5
2
Pho1
-general settings-
upper-z: 5000
lower-z: 1
stroke-width: 1.0
ignore z
filled
-specific settings-
use rectangles
diameter: 17
color:
show
list
settings
Sheet1
Color scheme
Edit
100%
Type here to search
ENG
10:03 PM
11/7/2023

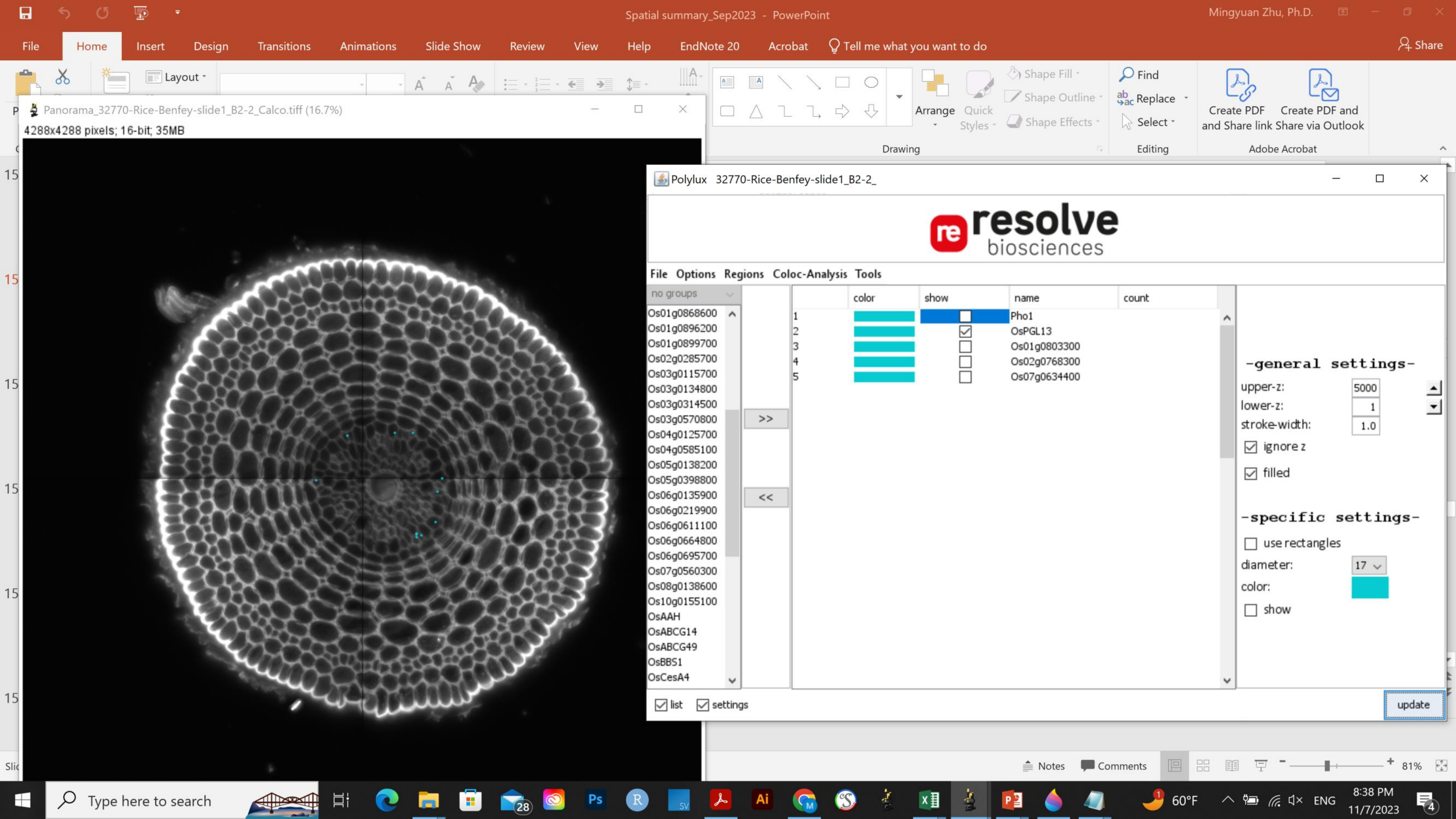
Spatial summary_Sep2023 - PowerPoint
Mingyuan Zhu, Ph.D.
File
Home
Insert
Design
Transitions
Animations
Slide Show
Review
View
Help
EndNote 20
Acrobat
Tell me what you want to do
Share
Layout
Shape Fill
Shape Outline
Shape Effects
Arrange
Quick Styles
Find
Replace
Select
Create PDF and Share link
Create PDF and Share via Outlook
Drawing
Editing
Adobe Acrobat
Panorama_32770-Rice-Benfey-slide1_B2-2_Calco.tiff (16.7%)
4288x4288 pixels; 16-bit; 35MB
Polylux 32770-Rice-Benfey-slide1_B2-2_
resolve biosciences
File Options Regions Coloc-Analysis Tools
no groups
Os01g0868600
Os01g0896200
Os01g0899700
Os02g0285700
Os03g0115700
Os03g0134800
Os03g0314500
Os03g0570800
Os04g0125700
Os04g0585100
Os05g0138200
Os05g0398800
Os06g0135900
Os06g0219900
Os06g0611100
Os06g0664800
Os06g0695700
Os07g0560300
Os08g0138600
Os10g0155100
OsAAH
OsABCG14
OsABCG49
OsBBS1
OsCesA4
>>
<<
color
show
name
count
1 Pho1
2 OsPGL13
3 Os01g0803300
4 Os02g0768300
5 Os07g0634400
-general settings-
upper-z: 5000
lower-z: 1
stroke-width: 1.0
ignore z
filled
-specific settings-
use rectangles
diameter: 17
color:
show
list
settings
update
Notes
Comments
81%
Type here to search
60°F
ENG
8:38 PM
11/7/2023

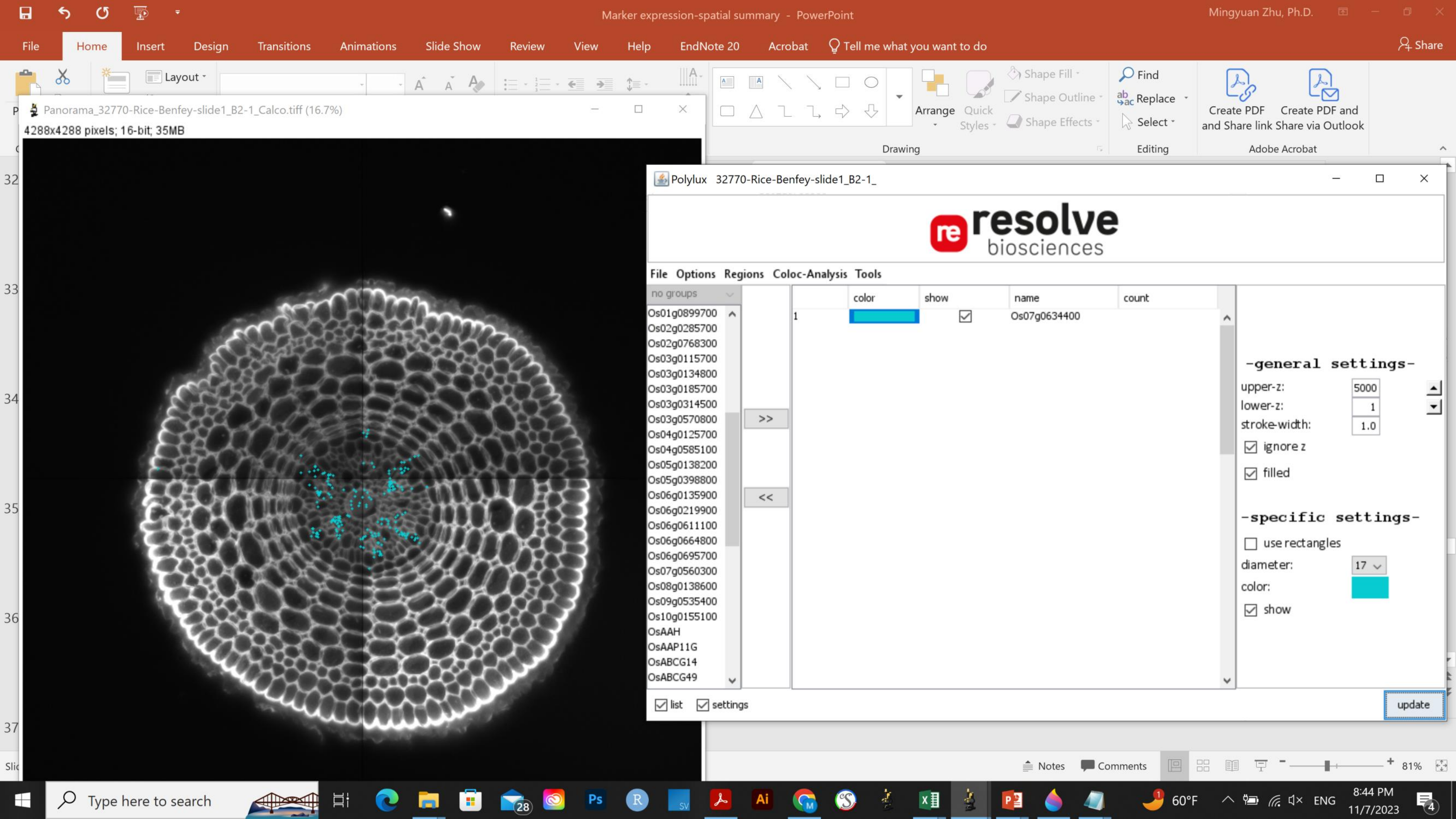
Marker expression-spatial summary - PowerPoint
Mingyuan Zhu, Ph.D.
File
Home
Insert
Design
Transitions
Animations
Slide Show
Review
View
Help
EndNote 20
Acrobat
Tell me what you want to do
Share
Layout
Arrange
Quick Styles
Shape Fill
Shape Outline
Shape Effects
Find
Replace
Select
Create PDF and Share link
Create PDF and Share via Outlook
Drawing
Editing
Adobe Acrobat
Panorama_32770-Rice-Benfey-slide1_B2-1_Calco.tiff (16.7%)
4288x4288 pixels; 16-bit; 35MB
Polylux 32770-Rice-Benfey-slide1_B2-1_
resolve biosciences
File Options Regions Coloc-Analysis Tools
no groups
Os01g0899700
Os02g0285700
Os02g0768300
Os03g0115700
Os03g0134800
Os03g0185700
Os03g0314500
Os03g0570800
Os04g0125700
Os04g0585100
Os05g0138200
Os05g0398800
Os06g0135900
Os06g0219900
Os06g0611100
Os06g0664800
Os06g0695700
Os07g0560300
Os08g0138600
Os09g0535400
Os10g0155100
OsAAH
OsAAP11G
OsABCG14
OsABCG49
>>
<<
color
show
name
count
1
Os07g0634400
-general settings-
upper-z: 5000
lower-z: 1
stroke-width: 1.0
ignore z
filled
-specific settings-
use rectangles
diameter: 17
color:
show
list
settings
update
Notes
Comments
81%
Type here to search
60°F
ENG
8:44 PM
11/7/2023

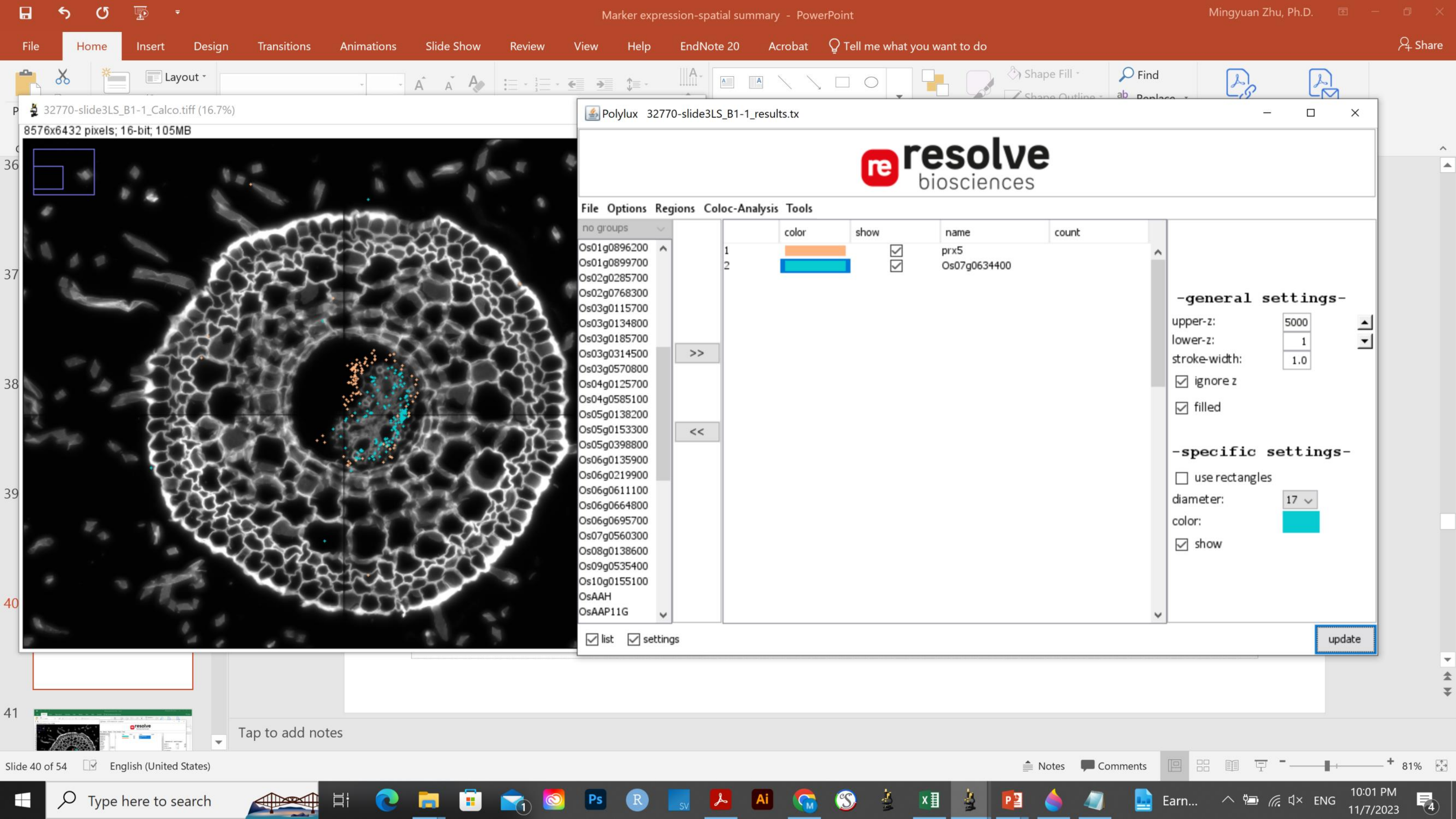
Marker expression-spatial summary - PowerPoint
Mingyuan Zhu, Ph.D.
File
Home
Insert
Design
Transitions
Animations
Slide Show
Review
View
Help
EndNote 20
Acrobat
Tell me what you want to do
Share
Layout
Shape Fill
Find
32770-slide3LS_B1-1_Calco.tiff (16.7%)
8576x6432 pixels; 16-bit; 105MB
Polylux 32770-slide3LS_B1-1_results.tx
resolve
biosciences
File
Options
Regions
Coloc-Analysis
Tools
no groups
Os01g0896200
Os01g0899700
Os02g0285700
Os02g0768300
Os03g0115700
Os03g0134800
Os03g0185700
Os03g0314500
Os03g0570800
Os04g0125700
Os04g0585100
Os05g0138200
Os05g0153300
Os05g0398800
Os06g0135900
Os06g0219900
Os06g0611100
Os06g0664800
Os06g0695700
Os07g0560300
Os08g0138600
Os09g0535400
Os10g0155100
OsAAH
OsAAP11G
>>
<<
color
show
name
count
1
prx5
2
Os07g0634400
-general settings-
upper-z:
5000
lower-z:
1
stroke-width:
1.0
ignore z
filled
-specific settings-
use rectangles
diameter:
17
color:
show
list
settings
update
Tap to add notes
Slide 40 of 54
English (United States)
Notes
Comments
81%
Type here to search
10:01 PM
11/7/2023

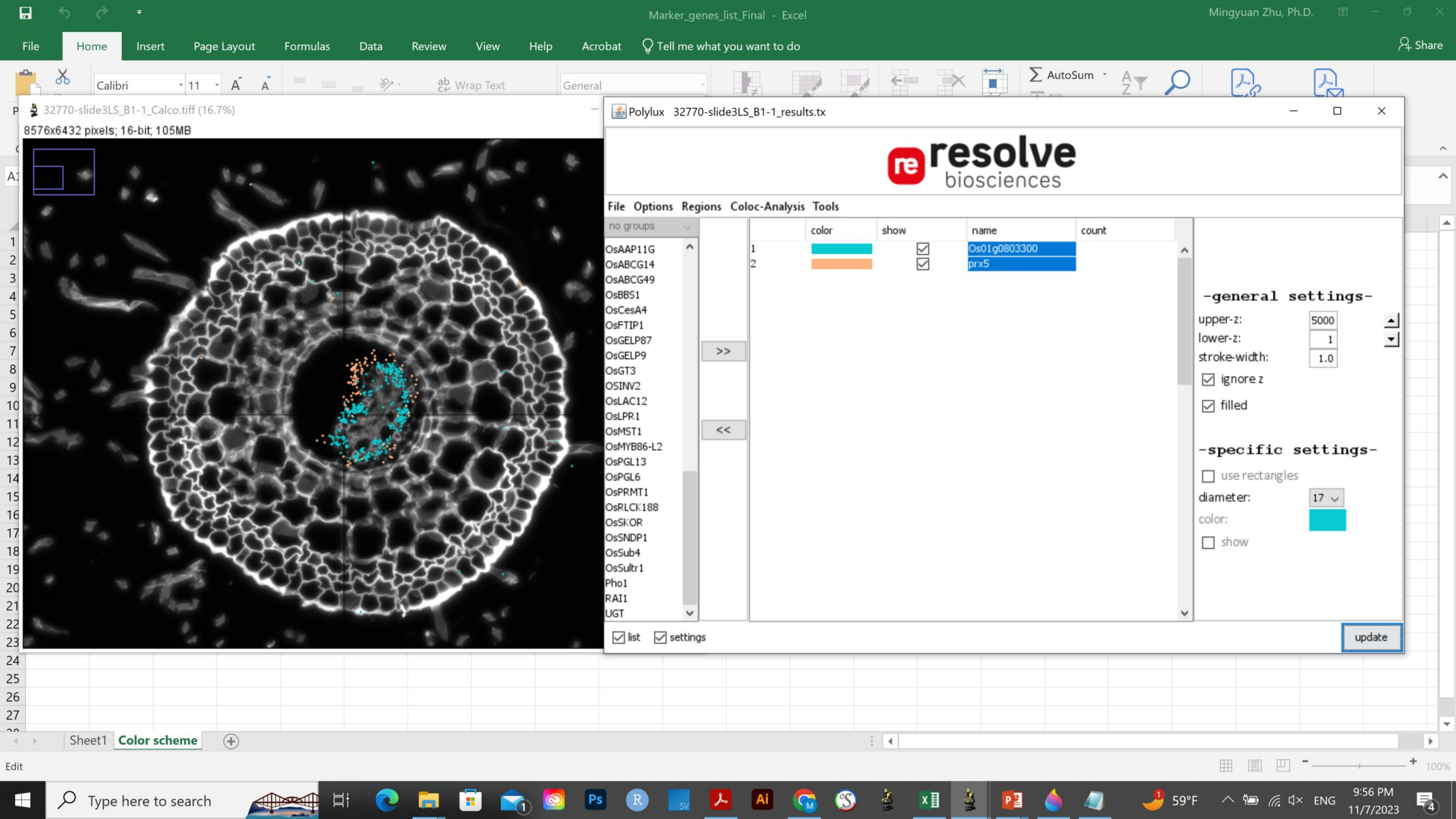
Marker_genes_list_Final - Excel
Mingyuan Zhu, Ph.D.
File
Home
Insert
Page Layout
Formulas
Data
Review
View
Help
Acrobat
Tell me what you want to do
Share
Calibri
11
Wrap Text
General
AutoSum
32770-slide3LS_B1-1_Calco.tiff (16.7%)
8576x6432 pixels; 16-bit; 105MB
Polylux 32770-slide3LS_B1-1_results.tx
resolve
biosciences
File
Options
Regions
Coloc-Analysis
Tools
no groups
OsAAP11G
OsABCG14
OsABCG49
OsBBS1
OsCesA4
OsFTIP1
OsGELP87
OsGELP9
OsGT3
OSINV2
OsLAC12
OsLPR1
OsMST1
OsMYB86-L2
OsPGL13
OsPGL6
OsPRMT1
OsRLCK188
OsSKOR
OsSNDP1
OsSub4
OsSultr1
Pho1
RAI1
UGT
>>
<<
color
show
name
count
1
Os01g0803300
2
prx5
-general settings-
upper-z:
5000
lower-z:
1
stroke-width:
1.0
ignore z
filled
-specific settings-
use rectangles
diameter:
17
color:
show
list
settings
update
Sheet1
Color scheme
Edit
100%
Type here to search
59°F
ENG
9:56 PM
11/7/2023

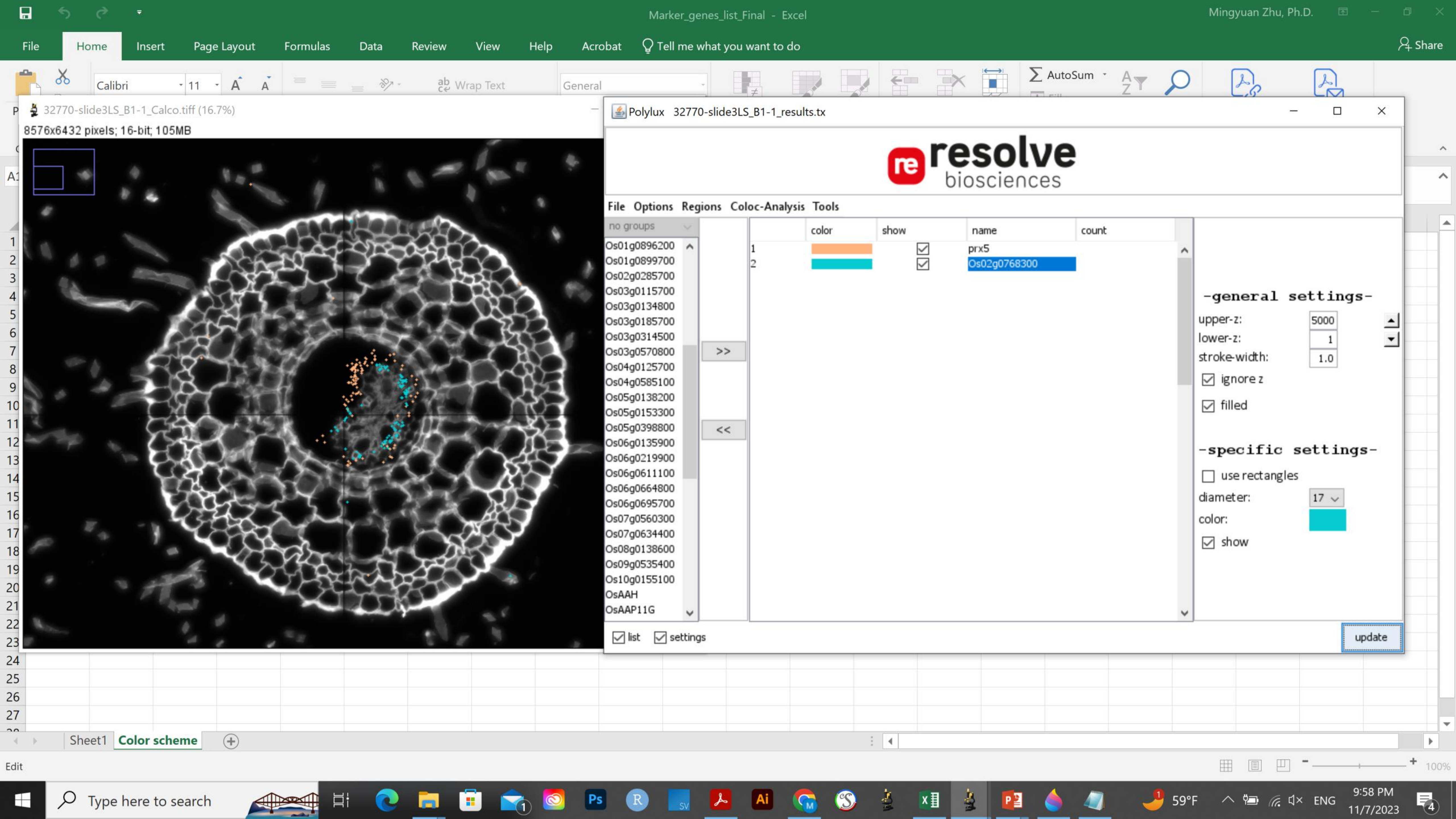

Marker_genes_list_Final - Excel
Mingyuan Zhu, Ph.D.
File Home Insert Page Layout Formulas Data Review View Help Acrobat Tell me what you want to do
Share
Calibri 11
Wrap Text
General
AutoSum
32770-slide3LS_B1-1_Calco.tiff (16.7%)
8576x6432 pixels; 16-bit; 105MB
Polylux 32770-slide3LS_B1-1_results.tx
resolve biosciences
File Options Regions Coloc-Analysis Tools
no groups
Os01g0896200
Os01g0899700
Os02g0285700
Os03g0115700
Os03g0134800
Os03g0185700
Os03g0314500
Os03g0570800
Os04g0125700
Os04g0585100
Os05g0138200
Os05g0153300
Os05g0398800
Os06g0135900
Os06g0219900
Os06g0611100
Os06g0664800
Os06g0695700
Os07g0560300
Os07g0634400
Os08g0138600
Os09g0535400
Os10g0155100
OsAAH
OsAAP11G
>>
<<
color show name count
1 prx5
2 Os02g0768300
-general settings-
upper-z: 5000
lower-z: 1
stroke-width: 1.0
ignore z
filled
-specific settings-
use rectangles
diameter: 17
color:
show
list settings
update
Sheet1 Color scheme
Edit
100%
Type here to search
59°F ENG
9:58 PM
11/7/2023

Phloem

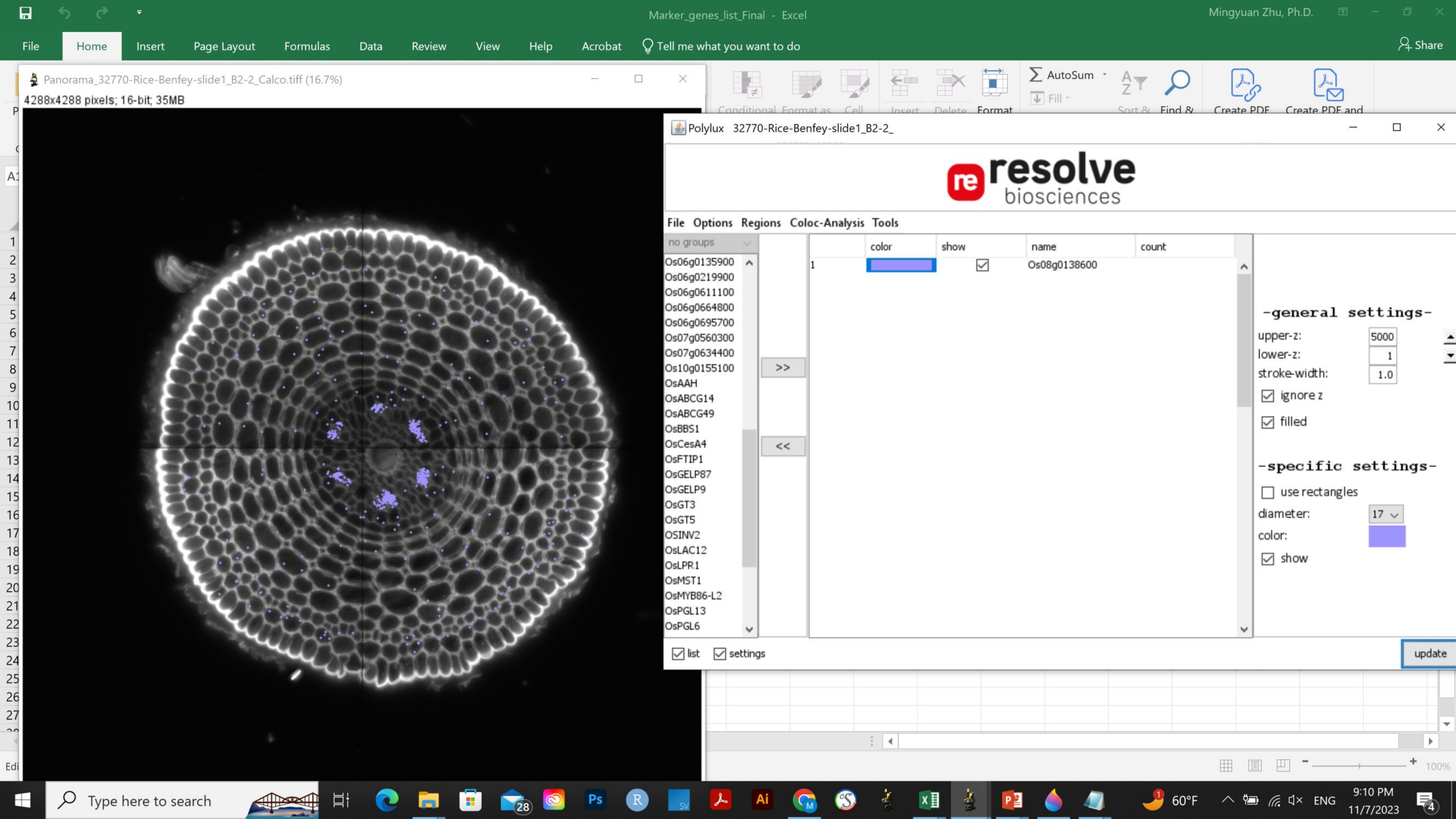
Marker_genes_list_Final - Excel
Mingyuan Zhu, Ph.D.
File
Home
Insert
Page Layout
Formulas
Data
Review
View
Help
Acrobat
Tell me what you want to do
Share
Panorama_32770-Rice-Benfey-slide1_B2-2_Calco.tiff (16.7%)
4288x4288 pixels; 16-bit; 35MB
AutoSum
Fill
Format
Find &
Create PDF
Create PDF and
Polylux 32770-Rice-Benfey-slide1_B2-2_
resolve
biosciences
File
Options
Regions
Coloc-Analysis
Tools
no groups
Os06g0135900
Os06g0219900
Os06g0611100
Os06g0664800
Os06g0695700
Os07g0560300
Os07g0634400
Os10g0155100
OsAAH
OsABCG14
OsABCG49
OsBBS1
OsCesA4
OsFTIP1
OsGELP87
OsGELP9
OsGT3
OsGT5
OSINV2
OsLAC12
OsLPR1
OsMST1
OsMYB86-L2
OsPGL13
OsPGL6
>>
<<
color
show
name
count
1
Os08g0138600
-general settings-
upper-z:
5000
lower-z:
1
stroke-width:
1.0
ignore z
filled
-specific settings-
use rectangles
diameter:
17
color:
show
list
settings
update
Type here to search
60°F
ENG
9:10 PM
11/7/2023

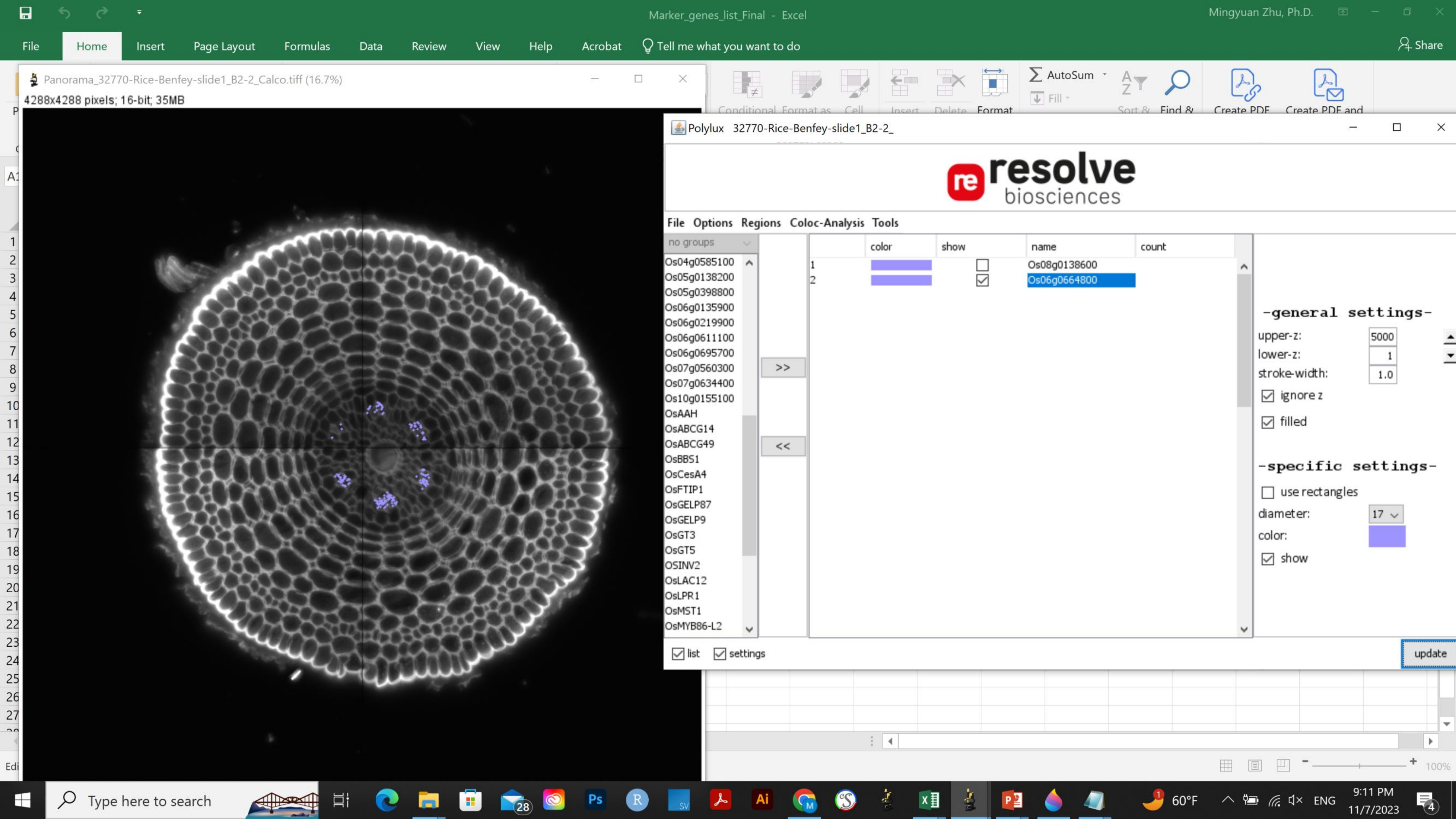
Marker_genes_list_Final - Excel
Mingyuan Zhu, Ph.D.
File
Home
Insert
Page Layout
Formulas
Data
Review
View
Help
Acrobat
Tell me what you want to do
Share
Panorama_32770-Rice-Benfey-slide1_B2-2_Calco.tiff (16.7%)
4288x4288 pixels; 16-bit; 35MB
Polylux 32770-Rice-Benfey-slide1_B2-2_
resolve biosciences
File
Options
Regions
Coloc-Analysis
Tools
no groups
Os04g0585100
Os05g0138200
Os05g0398800
Os06g0135900
Os06g0219900
Os06g0611100
Os06g0695700
Os07g0560300
Os07g0634400
Os10g0155100
OsAAH
OsABCG14
OsABCG49
OsBBS1
OsCesA4
OsFTIP1
OsGELP87
OsGELP9
OsGT3
OsGT5
OSINV2
OsLAC12
OsLPR1
OsMST1
OsMYB86-L2
color
show
name
count
1 Os08g0138600
2 Os06g0664800
-general settings-
upper-z: 5000
lower-z: 1
stroke-width: 1.0
ignore z
filled
-specific settings-
use rectangles
diameter: 17
color:
show
list
settings
update
Type here to search
60°F
ENG
9:11 PM
11/7/2023

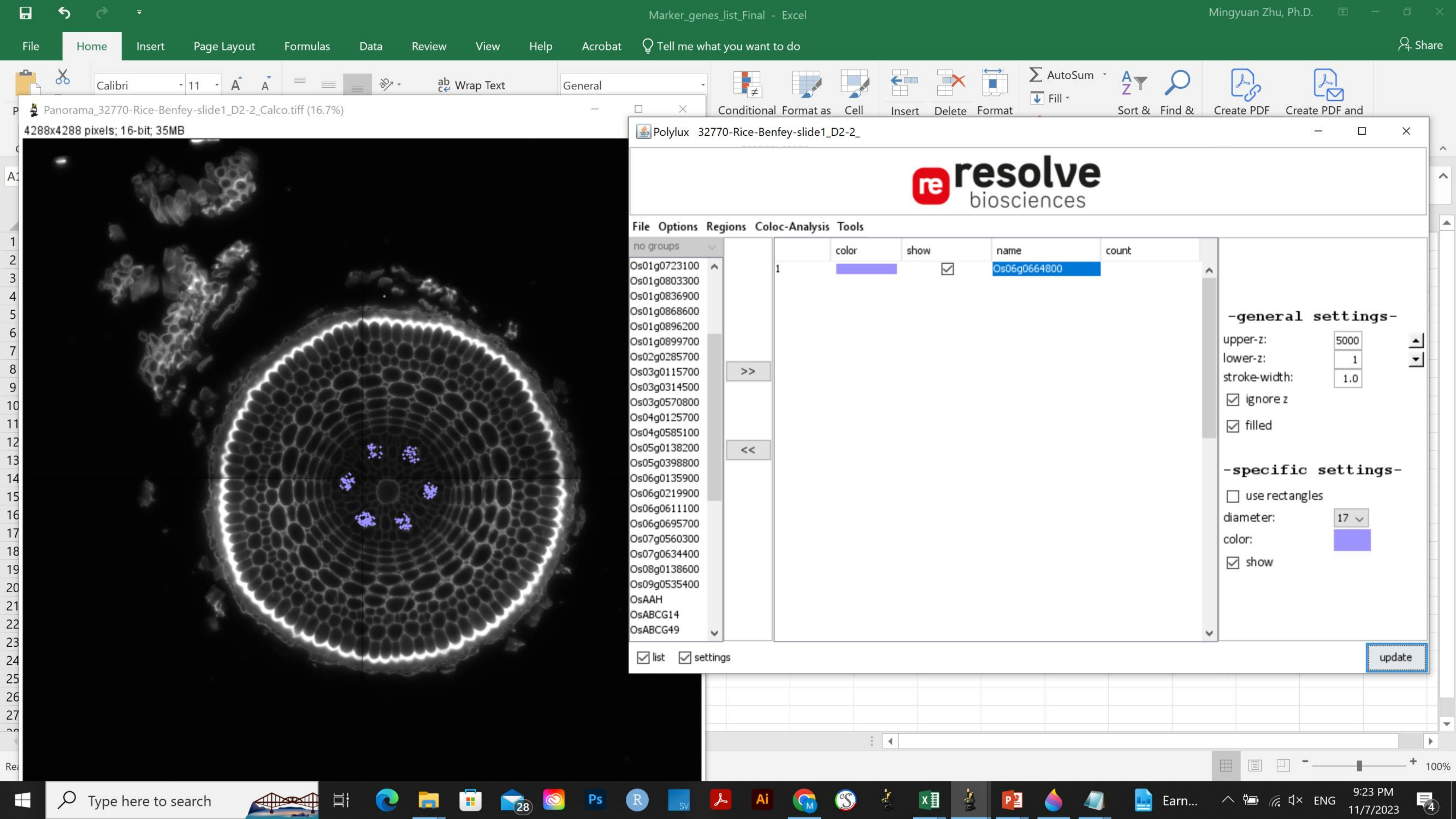

Marker_genes_list_Final - Excel
Mingyuan Zhu, Ph.D.
File Home Insert Page Layout Formulas Data Review View Help Acrobat Tell me what you want to do
Share
Calibri 11
Wrap Text
General
Conditional Format as Cell
Insert Delete Format
AutoSum
Fill
Sort & Find &
Create PDF Create PDF and
Panorama_32770-Rice-Benfey-slide1_D2-2_Calco.tiff (16.7%)
4288x4288 pixels; 16-bit; 35MB
Polylux 32770-Rice-Benfey-slide1_D2-2_
resolve biosciences
File Options Regions Coloc-Analysis Tools
no groups
Os01g0723100
Os01g0803300
Os01g0836900
Os01g0868600
Os01g0896200
Os01g0899700
Os02g0285700
Os03g0115700
Os03g0314500
Os03g0570800
Os04g0125700
Os04g0585100
Os05g0138200
Os05g0398800
Os06g0135900
Os06g0219900
Os06g0611100
Os06g0695700
Os07g0560300
Os07g0634400
Os08g0138600
Os09g0535400
OsAAH
OsABCG14
OsABCG49
>>
<<
color show name count
1 Os06g0664800
-general settings-
upper-z: 5000
lower-z: 1
stroke-width: 1.0
ignore z
filled
-specific settings-
use rectangles
diameter: 17
color:
show
list settings
update
100%
Type here to search
Earn...
ENG
9:23 PM
11/7/2023

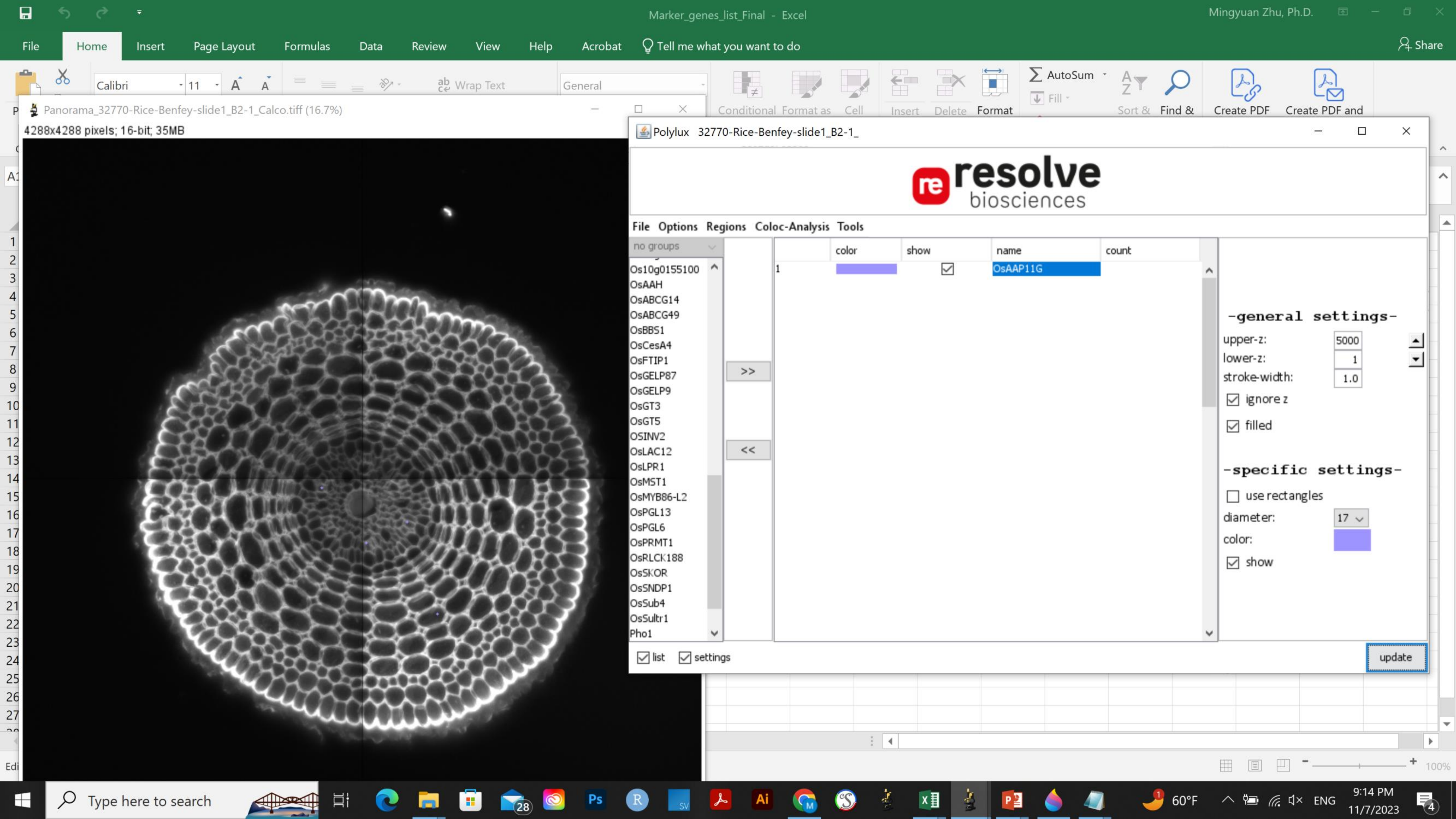
Marker_genes_list_Final - Excel
Mingyuan Zhu, Ph.D.
File
Home
Insert
Page Layout
Formulas
Data
Review
View
Help
Acrobat
Tell me what you want to do
Share
Calibri
11
Wrap Text
General
Conditional
Format as
Cell
Insert
Delete
Format
AutoSum
Fill
Sort &
Find &
Create PDF
Create PDF and
Panorama_32770-Rice-Benfey-slide1_B2-1_Calco.tiff (16.7%)
4288x4288 pixels; 16-bit; 35MB
Polylux 32770-Rice-Benfey-slide1_B2-1_
resolve
biosciences
File
Options
Regions
Coloc-Analysis
Tools
no groups
Os10g0155100
OsAAH
OsABCG14
OsABCG49
OsBBS1
OsCesA4
OsFTIP1
OsGELP87
OsGELP9
OsGT3
OsGT5
OSINV2
OsLAC12
OsLPR1
OsMST1
OsMYB86-L2
OsPGL13
OsPGL6
OsPRMT1
OsRLCK188
OsSKOR
OsSNDP1
OsSub4
OsSultr1
Pho1
>>
<<
color
show
name
count
1
OsAAP11G
-general settings-
upper-z:
5000
lower-z:
1
stroke-width:
1.0
ignore z
filled
-specific settings-
use rectangles
diameter:
17
color:
show
list
settings
update
Type here to search
60°F
ENG
9:14 PM
11/7/2023

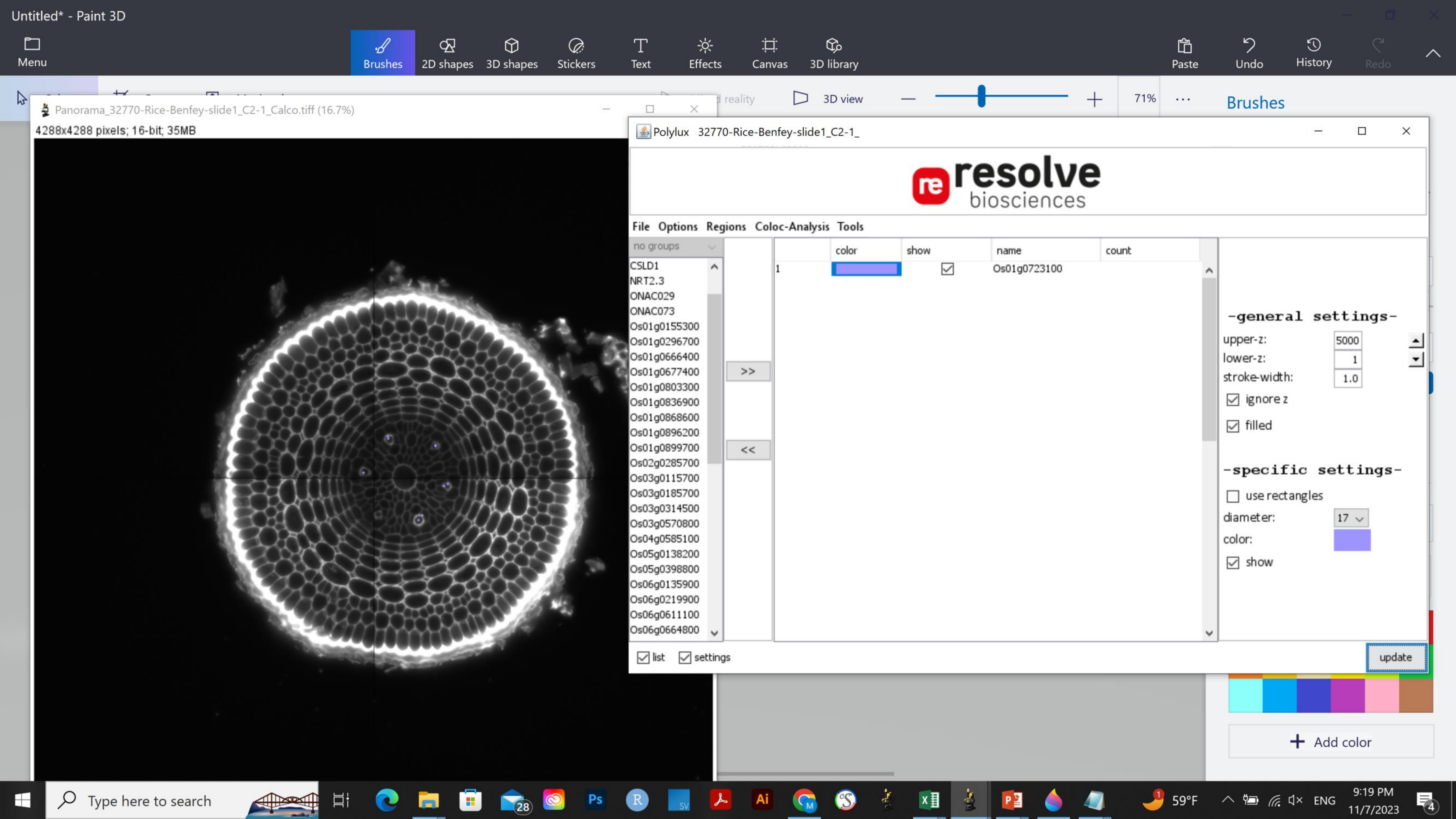

Untitled* - Paint 3D
Menu
Brushes
2D shapes
3D shapes
Stickers
Text
Effects
Canvas
3D library
Paste
Undo
History
Redo
3D view
71%
Brushes
Panorama_32770-Rice-Benfey-slide1_C2-1_Calco.tiff (16.7%)
4288x4288 pixels; 16-bit; 35MB
Polylux 32770-Rice-Benfey-slide1_C2-1_
resolve biosciences
File Options Regions Coloc-Analysis Tools
no groups
CSLD1
NRT2.3
ONAC029
ONAC073
Os01g0155300
Os01g0296700
Os01g0666400
Os01g0677400
Os01g0803300
Os01g0836900
Os01g0868600
Os01g0896200
Os01g0899700
Os02g0285700
Os03g0115700
Os03g0185700
Os03g0314500
Os03g0570800
Os04g0585100
Os05g0138200
Os05g0398800
Os06g0135900
Os06g0219900
Os06g0611100
Os06g0664800
>>
<<
color
show
name
count
1
Os01g0723100
-general settings-
upper-z: 5000
lower-z: 1
stroke-width: 1.0
ignore z
filled
-specific settings-
use rectangles
diameter: 17
color:
show
list
settings
update
Add color
Type here to search
59°F
ENG
9:19 PM
11/7/2023

Xylem

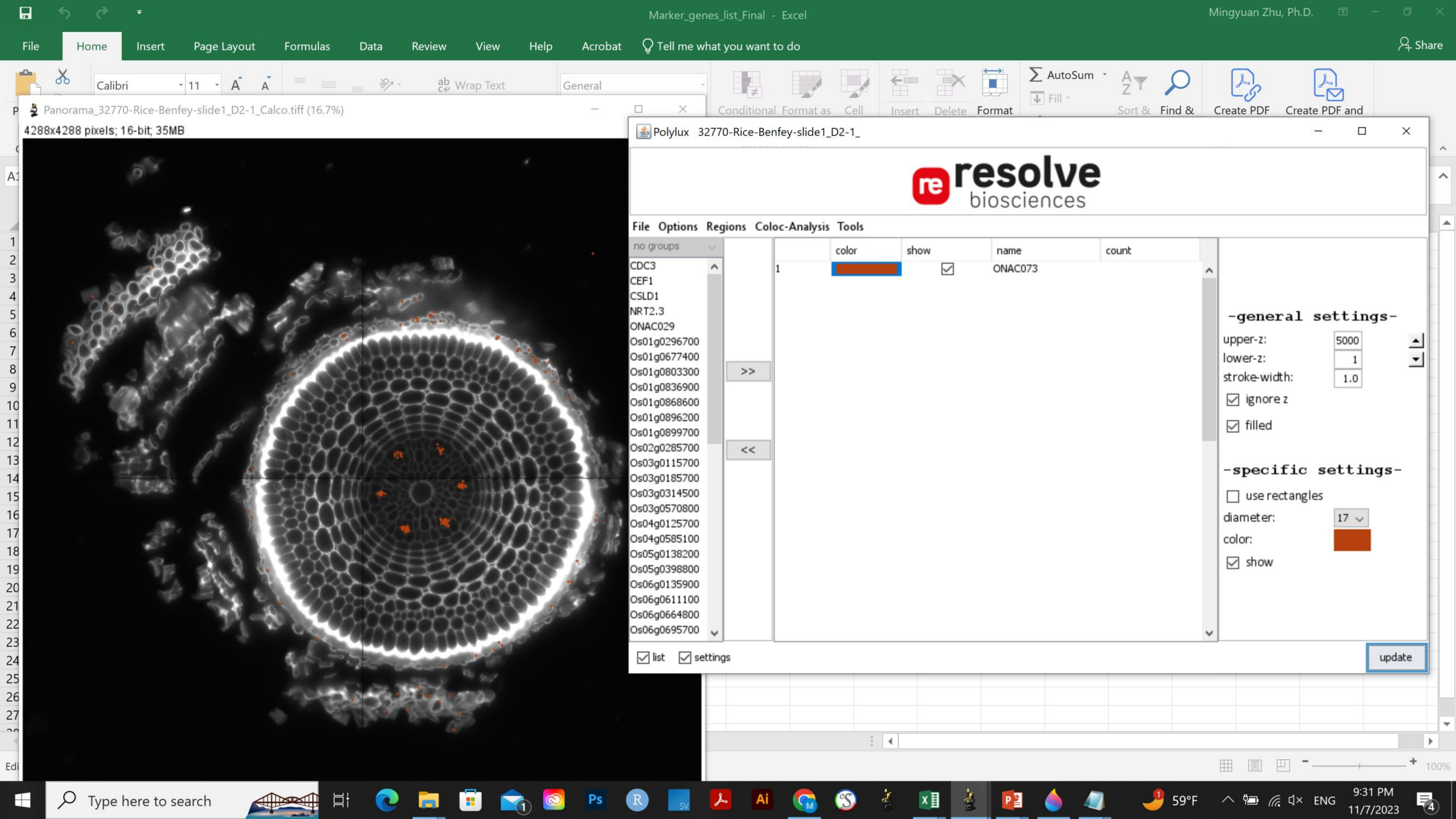

Marker_genes_list_Final - Excel
Mingyuan Zhu, Ph.D.
File
Home
Insert
Page Layout
Formulas
Data
Review
View
Help
Acrobat
Tell me what you want to do
Share
Panorama_32770-Rice-Benfey-slide1_D2-1_Calco.tiff (16.7%)
4288x4288 pixels; 16-bit; 35MB
Polylux 32770-Rice-Benfey-slide1_D2-1_
resolve biosciences
File Options Regions Coloc-Analysis Tools
no groups
CDC3
CEF1
CSLD1
NRT2.3
ONAC029
Os01g0296700
Os01g0677400
Os01g0803300
Os01g0836900
Os01g0868600
Os01g0896200
Os01g0899700
Os02g0285700
Os03g0115700
Os03g0185700
Os03g0314500
Os03g0570800
Os04g0125700
Os04g0585100
Os05g0138200
Os05g0398800
Os06g0135900
Os06g0611100
Os06g0664800
Os06g0695700
>>
<<
color
show
name
count
1
ONAC073
-general settings-
upper-z: 5000
lower-z: 1
stroke-width: 1.0
ignore z
filled
-specific settings-
use rectangles
diameter: 17
color:
show
list
settings
update
Type here to search
59°F
ENG
9:31 PM
11/7/2023

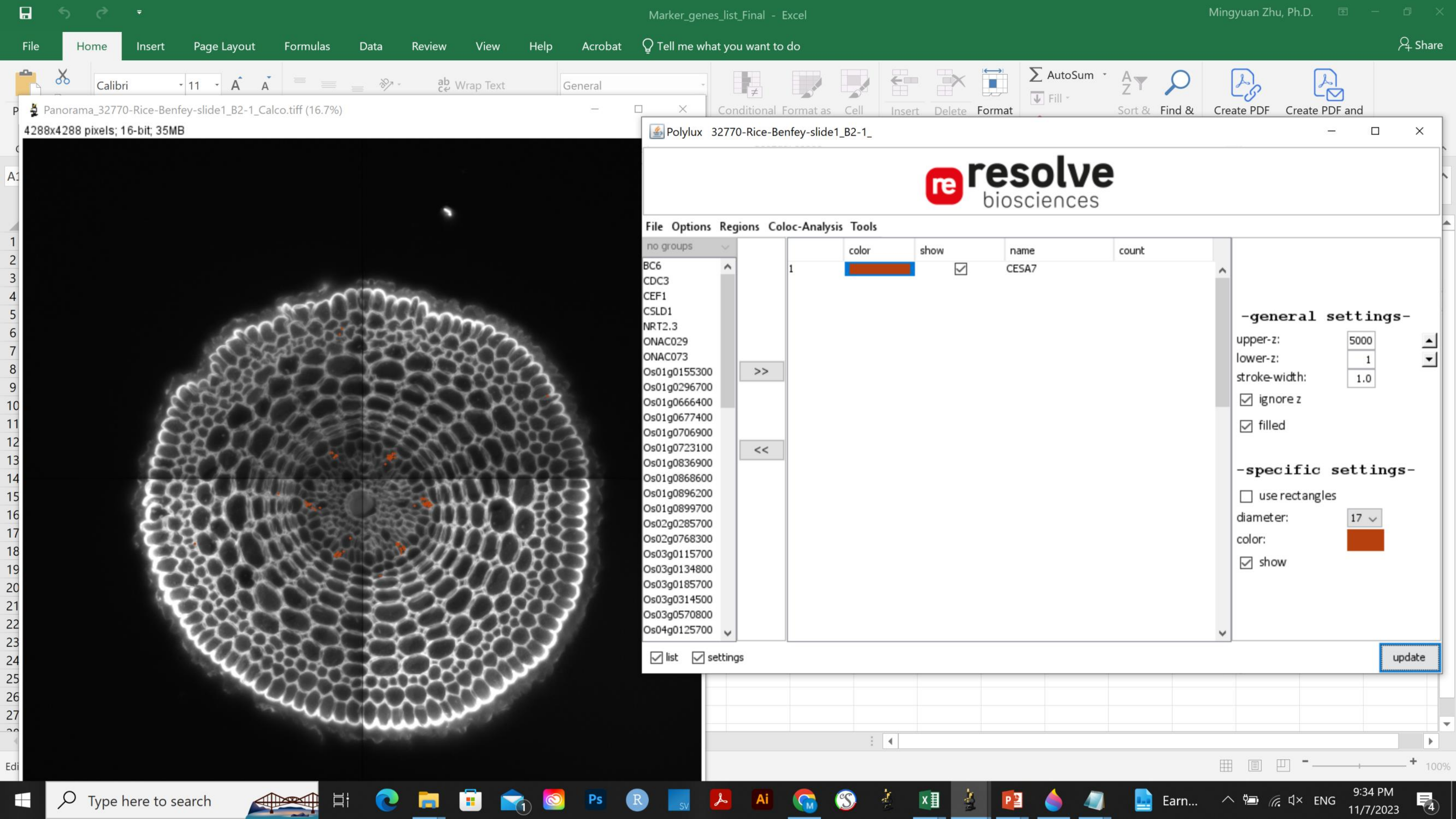
Marker_genes_list_Final - Excel
Mingyuan Zhu, Ph.D.
File Home Insert Page Layout Formulas Data Review View Help Acrobat Tell me what you want to do
Share
Calibri 11
Wrap Text
General
Conditional Format as Cell
Insert Delete Format
AutoSum
Fill
Sort & Find &
Create PDF
Create PDF and
Panorama_32770-Rice-Benfey-slide1_B2-1_Calco.tiff (16.7%)
4288x4288 pixels; 16-bit; 35MB
Polylux 32770-Rice-Benfey-slide1_B2-1_
resolve biosciences
File Options Regions Coloc-Analysis Tools
no groups
BC6
CDC3
CEF1
CSLD1
NRT2.3
ONAC029
ONAC073
Os01g0155300
Os01g0296700
Os01g0666400
Os01g0677400
Os01g0706900
Os01g0723100
Os01g0836900
Os01g0868600
Os01g0896200
Os01g0899700
Os02g0285700
Os02g0768300
Os03g0115700
Os03g0134800
Os03g0185700
Os03g0314500
Os03g0570800
Os04g0125700
>>
<<
color show name count
1 CESA7
-general settings-
upper-z: 5000
lower-z: 1
stroke-width: 1.0
ignore z
filled
-specific settings-
use rectangles
diameter: 17
color:
show
list settings
update
Type here to search
Earn...
ENG
9:34 PM
11/7/2023

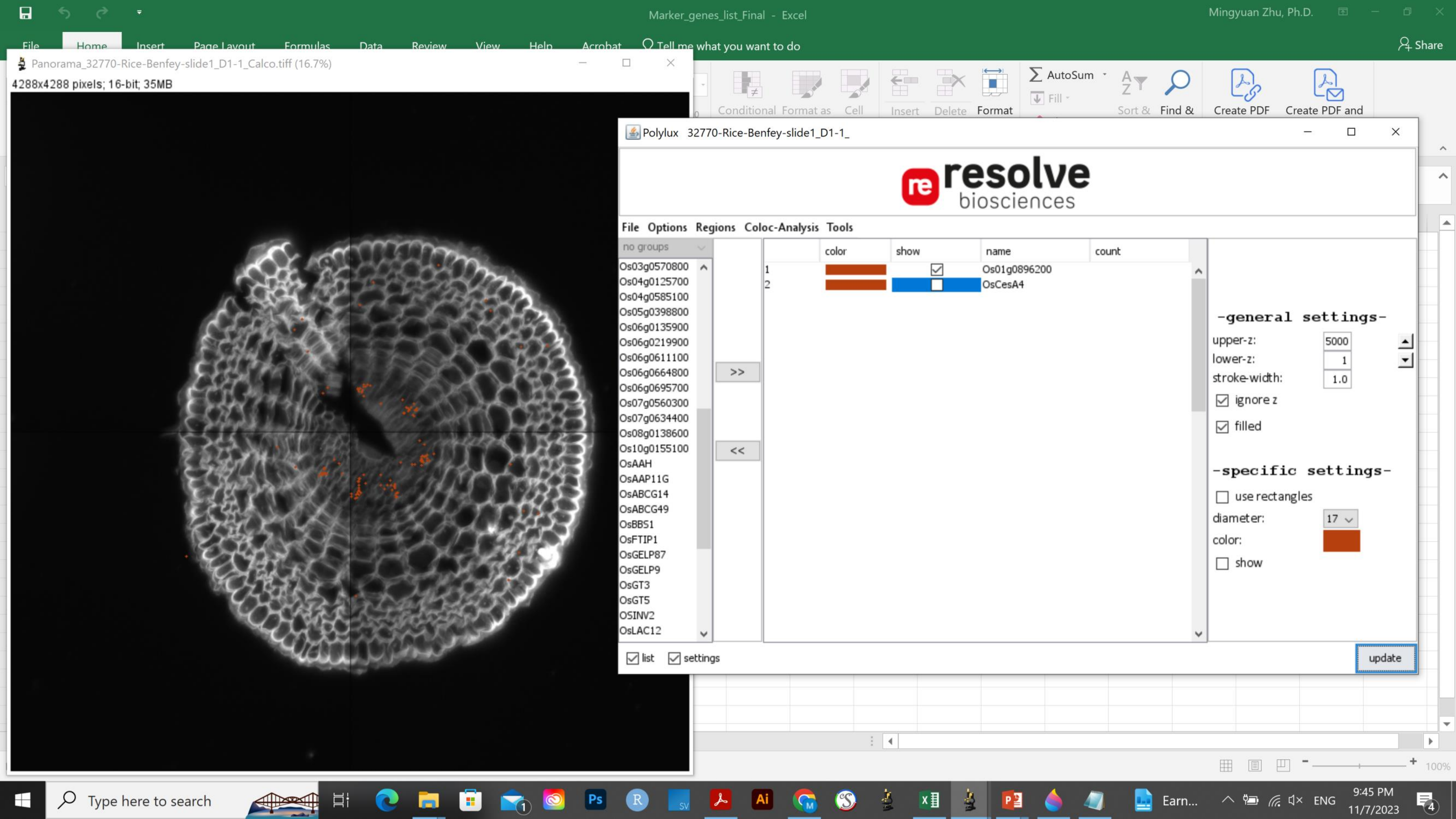
Marker_genes_list_Final - Excel
Mingyuan Zhu, Ph.D.
Panorama_32770-Rice-Benfey-slide1_D1-1_Calco.tiff (16.7%)
4288x4288 pixels; 16-bit; 35MB
Polylux 32770-Rice-Benfey-slide1_D1-1_
resolve biosciences
File Options Regions Coloc-Analysis Tools
no groups
Os03g0570800
Os04g0125700
Os04g0585100
Os05g0398800
Os06g0135900
Os06g0219900
Os06g0611100
Os06g0664800
Os06g0695700
Os07g0560300
Os07g0634400
Os08g0138600
Os10g0155100
OsAAH
OsAAP11G
OsABCG14
OsABCG49
OsBBS1
OsFTIP1
OsGELP87
OsGELP9
OsGT3
OsGT5
OSINV2
OsLAC12
color show name count
1 Os01g0896200
2 OsCesA4
-general settings-
upper-z: 5000
lower-z: 1
stroke-width: 1.0
ignore z
filled
-specific settings-
use rectangles
diameter: 17
color:
show
list settings
update
9:45 PM
11/7/2023

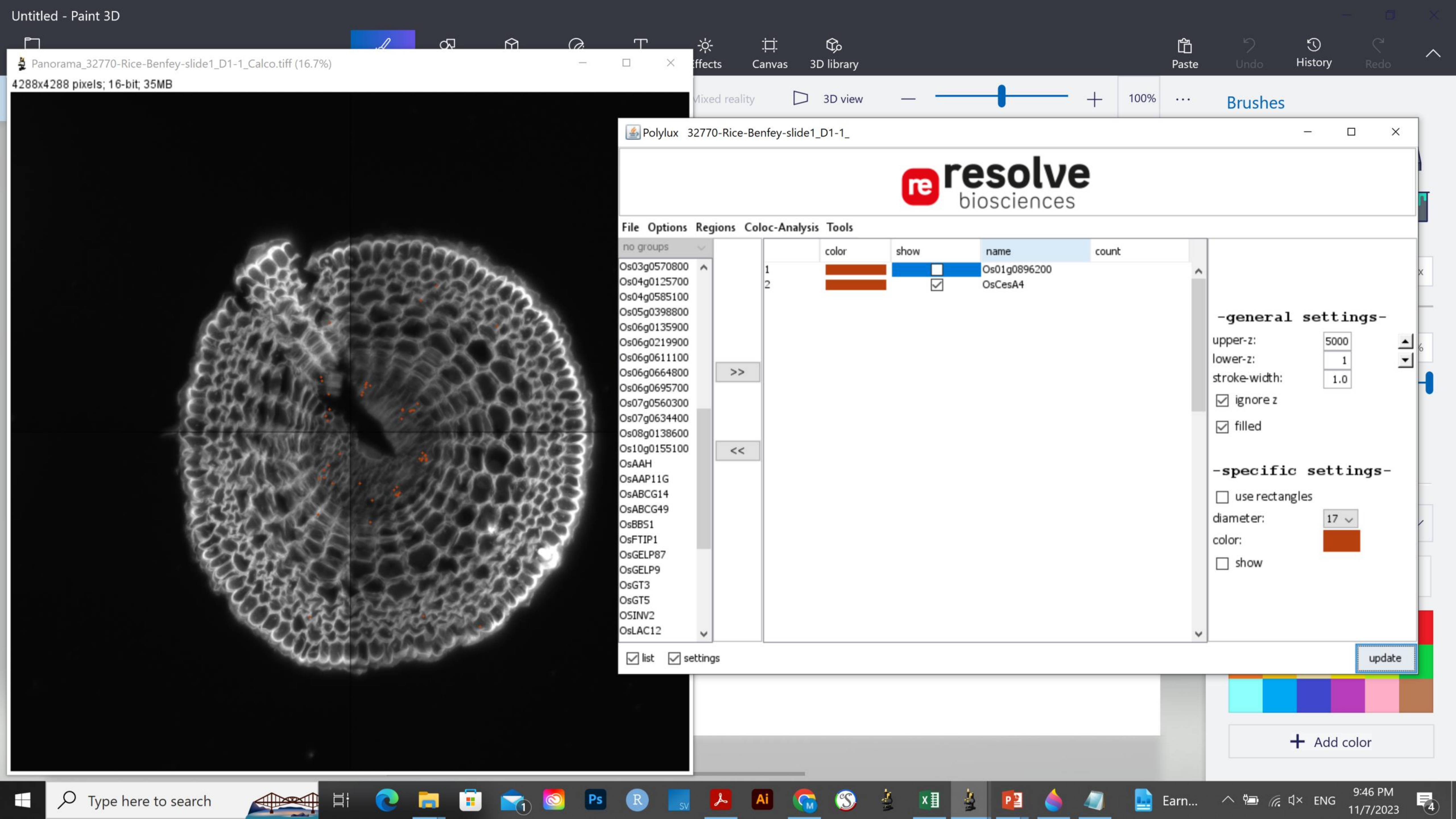
Untitled - Paint 3D
Effects
Canvas
3D library
Paste
Undo
History
Redo
Mixed reality
3D view
100%
Brushes
Panorama_32770-Rice-Benfey-slide1_D1-1_Calco.tiff (16.7%)
4288x4288 pixels; 16-bit; 35MB
Polylux 32770-Rice-Benfey-slide1_D1-1_
resolve biosciences
File Options Regions Coloc-Analysis Tools
no groups
Os03g0570800
Os04g0125700
Os04g0585100
Os05g0398800
Os06g0135900
Os06g0219900
Os06g0611100
Os06g0664800
Os06g0695700
Os07g0560300
Os07g0634400
Os08g0138600
Os10g0155100
OsAAH
OsAAP11G
OsABCG14
OsABCG49
OsBBS1
OsFTIP1
OsGELP87
OsGELP9
OsGT3
OsGT5
OSINV2
OsLAC12
>>
<<
color show name count
1 Os01g0896200
2 OsCesA4
-general settings-
upper-z: 5000
lower-z: 1
stroke-width: 1.0
ignore z
filled
-specific settings-
use rectangles
diameter: 17
color:
show
list
settings
update
Add color
Type here to search
Earn...
ENG
9:46 PM
11/7/2023

Marker_genes_list_Final - Excel

File | Home | Insert | Page Layout | Formulas | Data | Review | View | Help | Acrobat | Tell me what you want to do

32770-slide3LS_B1-1_Calco.tiff (16.7%)

8576x6432 pixels; 16-bit; 105MB

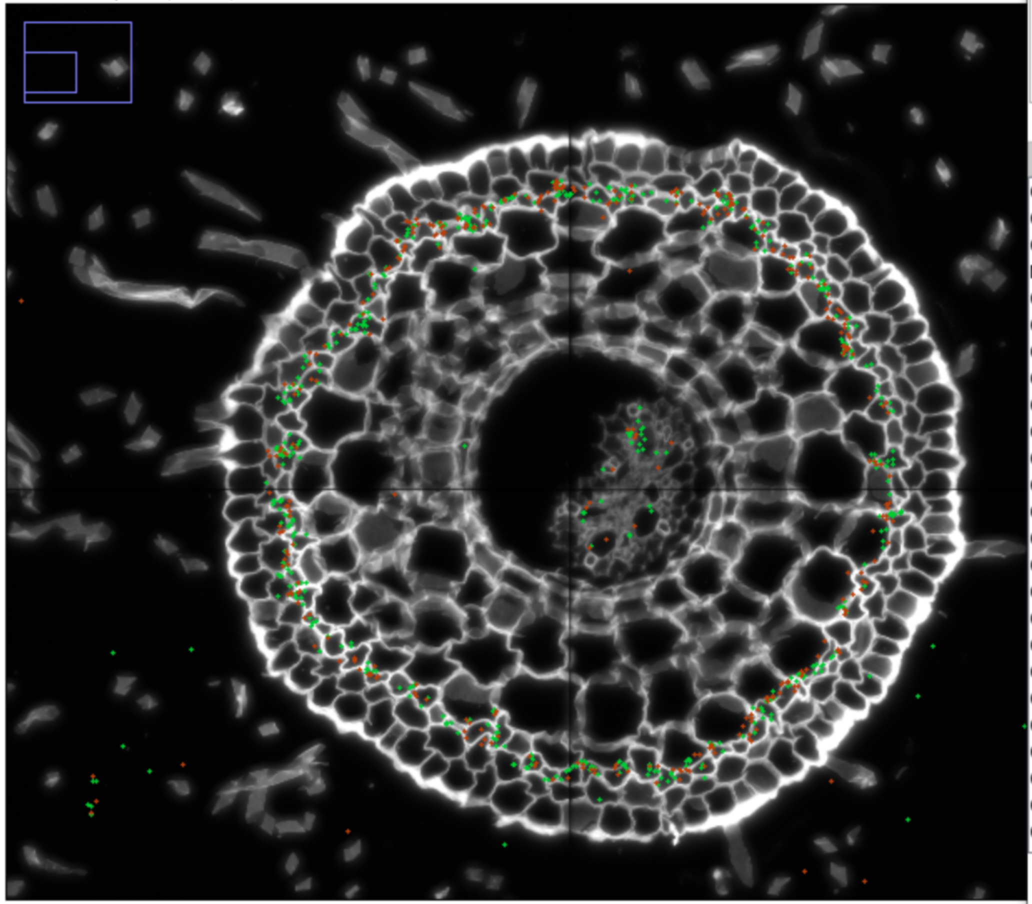

Polylux 32770-slide3LS_B1-1_results.tx

resolve biosciences

File Options Regions Coloc-Analysis Tools

no groups

CDC3
CEF1
CSLD1
NRT2.3
ONAC029
ONAC073
Os01g0155300
Os01g0296700
Os01g0666400
Os01g0677400
Os01g0706900
Os01g0723100
Os01g0803300
Os01g0836900
Os01g0868600
Os01g0896200
Os01g0899700
Os02g0285700
Os02g0768300
Os03g0115700
Os03g0134800
Os03g0185700
Os03g0314500
Os03g0570800
Os04g0125700

	color	show	name	count
1		☑	BC6	
2		☑	CESA7	

-general settings-

upper-z: 5000

lower-z: 1

stroke-width: 1.0

☑ ignore z

☑ filled

-specific settings-

☐ use rectangles

diameter: 17

color:

☑ show

☑ list ☑ settings

update

File Edit Image Process Analyze Plugins Window Help

ImageJ 2.0.0-rc-43/1.52n; Java 1.8.0_321 [64-bit];

Sheet1 | Color scheme

9:51 PM 11/7/2023
